# A scoping review and evidence map of radiofrequency field exposure and genotoxicity: assessing *in vivo, in vitro*, and epidemiological data

**DOI:** 10.3389/fpubh.2025.1613353

**Published:** 2025-07-30

**Authors:** Steven G. Weller, Julie E. McCredden, Victor Leach, Cordia Chu, Alfred King-yin Lam

**Affiliations:** ^1^Centre for Environment and Population Health, School of Medicine and Dentistry, Griffith University, Nathan, QLD, Australia; ^2^Oceania Radiofrequency Scientific Advisory Association Inc. (ORSAA), Scarborough, QLD, Australia; ^3^Department of Pathology, School of Medicine and Dentistry, Griffith University, Gold Coast Campus, Southport, QLD, Australia

**Keywords:** genotoxicity, radio frequencies, cancer, electromagnetic radiation, wireless technology, oxidative stress, apoptosis

## Abstract

**Background:**

Studies investigating genotoxic effects of radiofrequency electromagnetic field (RF-EMF) exposure (3 kHz−300 GHz) have used a wide variety of parameters, and results have been inconsistent. A systematic mapping of existing research is necessary to identify emerging patterns and to inform future research and policy.

**Methods:**

Evidence mapping was conducted using guidance from the Preferred Reporting Items for Systematic reviews and Meta-Analyses for Scoping Reviews (PRISMA-ScR). A comprehensive search strategy was applied across multiple research databases, using specific inclusion and exclusion criteria within each knowledge domain. Quantitative aggregation using tables, graphs and heat maps was used to synthesize data according to study type, organism type, exposure level and duration, biological markers (genotoxicity, cellular stress, apoptosis), RF-EMF signal characteristics, as well as funding source to further contextualize the evidence landscape. Quality criteria were applied as part of a focused analysis to explore potential biases and their effects on outcomes.

**Results:**

Over 500 pertinent studies were identified, categorized as *in vitro* (53%), *in vivo* (37%), and epidemiological (10%), and grouped according to type of DNA damage, organism, intensity, duration, signal characteristics, biological markers and funding source. *In vitro* studies predominantly showed proportionally fewer significant effects, while *in vivo* and epidemiological studies showed more. DNA base damage studies showed the highest proportion of effects, as did studies using GSM talk-mode, pulsed signals and real-world devices. A complex relationship was identified between exposure intensity and duration, with duration emerging as a critical determinant of outcomes. A complex U-shaped dose-response relationship was evident, suggesting adaptive cellular responses, with increased free radical production as a plausible mechanism. Higher-quality studies showed fewer significant effects; however, the funding source had a stronger influence on outcomes than study quality. Over half (58%) of studies observing DNA damage used exposures below the International Commission of Non-Ionizing Radiation Protection (ICNIRP) limits.

**Conclusion:**

The collective evidence reveals that RF-EMF exposures may be genotoxic and could pose a cancer risk. Exposure duration and real-world signals are the most important factors influencing genotoxicity, warranting further focused research. To address potential genotoxic risks, these findings support the adoption of precautionary measures alongside existing thermal-based exposure guidelines.

## Introduction

### Background and rationale

The world is facing a cancer pandemic, with exponential growth occurring in many cancers (International Agency for Research on Cancer (IARC) World Cancer Reports) (1–4). While population aging is contributing to this trend (5), it cannot fully explain the observed rise in certain cancers, suggesting that environmental and/or lifestyle factors are playing a role. One potential factor for consideration is the increasing global background levels of anthropogenic radiofrequency electromagnetic fields (RF-EMF) (6), which coincide with a general increase in cancer incidence rates over the last several decades. While this temporal overlap does not imply causation, it highlights the need for careful investigation into the potential role of RF-EMF exposure among the many environmental and lifestyle factors associated with cancer.

The current perspective held by IARC is that RF-EMF exposures, including exposure to mobile phones, are a group 2B possible carcinogen (IARC, May 2011) (7, 8). This classification was based on the available evidence at the time, which covered both epidemiological and animal experimental studies. While the evidence was deemed credible, bias and confounding could not be completely ruled out (8). A limited understanding of the underlying mechanisms, regarded by some as weak (9), also prevented a higher classification.

Genetic alteration is a well-established trigger for cancer development (10). Genotoxicity is the ability of a physical or chemical agent to induce genetic damage, which may result in genetic mutations (11), and represents a critical pathway to cancer. Therefore, if RF-EMF exposure is linked to genotoxicity, this would provide strong evidence for a plausible mechanism describing how RF-EMF may initiate carcinogenesis in humans and potentially in all living organisms. With the recommended prioritization for an IARC (12) assessment of the carcinogenic potential of radiofrequency exposures, an unbiased synthesis of the evidence on RF-induced genotoxicity is a crucial resource needed for such an investigation.

This review aims to determine whether RF-EMF exposure can damage DNA, thereby potentially contributing to the rising global incidence of cancer.

### Radiofrequency exposures

Radiofrequency electromagnetic fields are defined as non-ionizing electromagnetic frequencies in the range of 3 kilohertz (kHz) to 300 gigahertz (GHz) (13), which sit between extremely low-frequency fields (ELF), i.e., electrical power frequencies, and infrared light in the electromagnetic spectrum. RF-EMF is produced both naturally (as background cosmic radiation, lightning and other atmospheric activity) and from a wide range of man-made sources including radar, radio/TV broadcast antenna, satellite communications, mobile phone base stations, smart meters, smartphones, Bluetooth devices, game consoles, baby monitors, computers, Wi-Fi routers, microwave ovens, radiofrequency implanted devices, diathermy machines and wireless power transmission devices.

The primary source of natural, isotropic microwave background radiation on Earth is the Cosmic Microwave Background (14). This radiation falls to earth in a non-polarized and continuous manner, at very low intensity levels as low as 10^−18^ W/m^2^ (6) for frequencies used in the mobile phone RF-EMF spectrum. In contrast, man-made RF-EMF exposure is typically polarized and pulsed with intensities reaching up to 10 W/m^2^ for certain frequencies used in wireless communication, as permitted by the International Commission of Non-Ionizing Radiation Protection (ICNIRP) (15) for member of the public exposures. These important characteristics distinguish man-made from natural sources of radiation. Biological life has never before experienced this type of artificial radiation, which may have unique biological effects (16). Radiofrequency waves can penetrate human skin, depending on wave frequency and tissue properties (17). Lower-frequency RF waves penetrate deeper, reaching internal organs, while higher frequencies are more readily absorbed by the skin layers or reflected (18). Wireless communication technologies, such as mobile phones and Wi-Fi routers, rely on RF frequencies that can pass through barriers like walls, windows, and roofs so as to maintain signal coverage.

### DNA damage how it is measured

**Figure 1:**
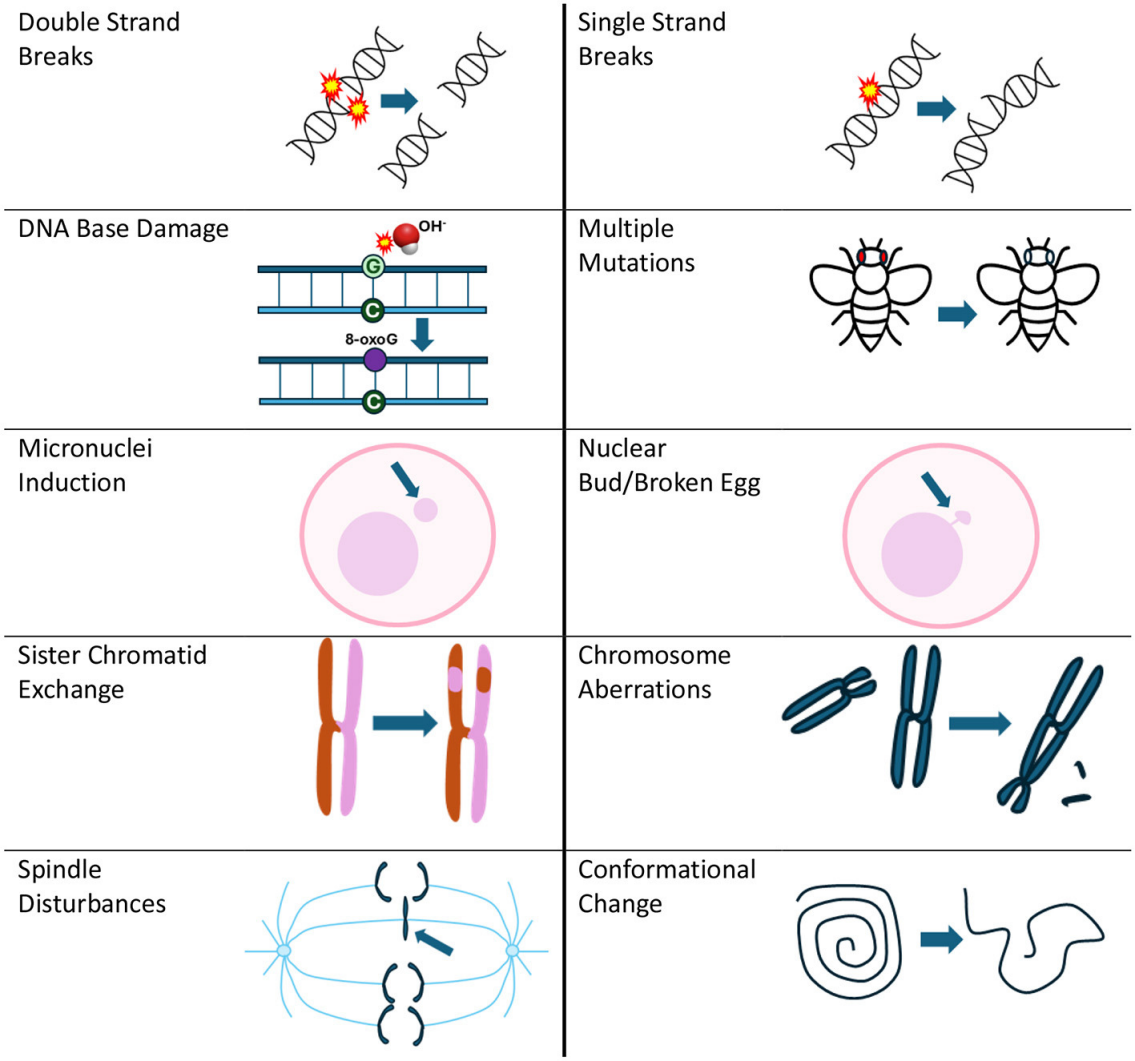
Types of DNA damage reported due to RF exposure that is covered in the evidence map. Mutations can be associated with many of the types of DNA damage presented in this figure, but for the evidence map, they reflect results from specific mutation assays listed in Supplementary Table 1.

DNA damage encompasses various forms (see Figure 1), including single-strand breaks (SSBs), double-strand breaks (DSBs), chromosome aberrations, sister chromatid exchanges and the presence of micronuclei (11), all of which can significantly impact cellular integrity.

Approximately 10,000 DNA modifications occur every hour per cell (19), and of these, SSBs are the most frequent types of DNA lesions (75%) (19). SSBs can arise from exposure to free radicals such as reactive oxygen and nitrogen species (ROS/RNS) (20). SSBs may also occur as intermediate products of the DNA repair process or as a result of abortive cellular enzyme activities (20). If they are not promptly and correctly repaired, SSBs can disrupt critical processes such as DNA replication and transcription, which ultimately compromises genome stability (21). DSBs, though less frequent, are particularly severe because they involve breaks in both DNA strands. DSBs pose a significant challenge to repair mechanisms and increase the risk of chromosomal aberrations and micronuclei, which serve as biomarkers for genome instability (22). The accumulation of unrepaired DNA damage, including SSBs, DSBs, and associated abnormalities, has been strongly implicated in the development of cancer (11), aging-related disorders (23), and neurodegenerative diseases (24).

Each type of DNA damage has specific assay methods (e.g., comet assay) to detect and evaluate the damage. The types of DNA damage and their associated assays that are investigated as part of the evidence map are detailed in Supplementary Table 1.^1^

DNA damage as measured via an assay represents the net result of three factors: (i) the damage that is induced by exposure to an external agent under test, (ii) the baseline level of endogenous DNA damage that occurs naturally under normal physiological conditions, and (iii) the extent of damage repaired by the cellular DNA repair mechanisms.

### The current state of knowledge and uncertainties

It has been well-established that the biological and health effects of microwaves depend on various biological and physical parameters that differ across studies, leading to variations in observed effect outcomes (25, 26).

Hundreds of experimental studies of varying quality have been conducted over many decades investigating whether RF-EMF exposure can damage DNA or result in genetic mutations. Several reviews have also been performed, each using a smaller subset of studies, with mixed results. The balance of evidence determined by these past reviews ranges from significant evidence of genetic damage or interference, Lai (27), to slightly favoring DNA damage, Ruediger (28), to claims that most studies show no significant effects, Vijayalaxmi and Prihoda (29, 30) and more recently, Romeo et al. (31).

Reasons for the large discrepancies in past reviews include:

1. The scope of the review, such as focusing only on *in vitro* studies or covering a more expansive set of *in vivo* and epidemiological studies;
2. The final pool of studies selected for review (allowing for selection bias);
3. Quality criteria used to exclude or downgrade relevant papers;
4. Review author (s) personal biases or affiliations.

Given the current incompleteness of available systematic reviews, inconsistencies in reporting and potential biases, an evidence map investigating the potential genotoxic effects of RF-EMF is both timely and essential.

## Aim, scope and objectives

The evidence map presented here aims to comprehensively assess the available research investigating potential genotoxic effects associated with radiofrequency (RF) electromagnetic fields (3 kHz to 300 GHz) exposure. The ultimate goal is to clarify whether RF exposure has a plausible role in damaging DNA, with subsequent implications for biological health and the induction of cancer.

The scope of this study encompasses all major forms of DNA damage as well as potential mechanistic pathways. The study examines experimental and observational research, including *in vitro, in vivo*, and epidemiological studies. It investigates all relevant past studies, including those published up until May 2023. The primary objectives are to catalog and synthesize this evidence, enabling the identification of potential biological mechanisms, patterns, gaps, and methodological limitations in the field. This study aims to:

- **Understand the diversity of evidence:** Provide a structured overview of the various forms of DNA damage studied in the context of different properties of RF exposure;
- **Discover potential biological mechanisms:** Investigate and categorize the main biological pathways and processes through which RF exposure might induce genetic damage, including direct and indirect effects;
- **Highlight quality issues and biases:** Identify the strengths and limitations of existing research, including methodological robustness, potential conflicts of interest, and funding sources;
- **Bridge data gaps for policy and research:** Offer insights into areas requiring further exploration to inform future experimental designs and public health guidelines;
- **Set the stage for quality-focused synthesis:** Lay the groundwork for a future systematic review and narrative analysis of high-quality studies;
- **Examine exposure levels in studies below the ICNIRP occupational limit for localized exposures:** Determine whether current international safety guidelines are effective in protecting all living entities from genetic damage associated with RF exposures.

### Primary question items

The primary question for this scoping review and evidence map is: What evidence exists regarding the genotoxic potential of anthropogenic RF-EMF exposures? A systematic mapping and synthesis of the following factors will help address this question:

1. location and history of research publications;
2. organisms and cell types for evaluating differential sensitivity to radiofrequency exposures;
3. balance of evidence across *in vitro, in vivo* and epidemiological studies for different types of DNA damage;
4. exposure signal characteristics (field intensity, duration, frequency, modulations, real or simulated) affecting the likelihood of detecting RF-EMF-induced genetic damage and its link to thermal or non-thermal interactions;
5. potential biological mechanisms that might explain RF-induced genotoxicity.

### Secondary question items

Further questions aim to explain the reasons for conflicting outcomes and inconsistencies found in previous reviews:

6. What does the evidence reveal about the risk of bias in study designs, as well as the influence of funding sources and researcher affiliations on study outcomes?
7. How does applying more stringent quality criteria alter the balance of evidence?
8. Is there sufficient homogeneity in the data to perform a meta-analysis in a future systematic review?
9. Does the existing evidence have gaps that require further exploration with future-focused research?

### PECO statement

**Populations:** all organisms and cell types, microorganisms or free DNA used in *in vitro, in vivo* and epidemiological studies.

**Exposures:** Anthropogenic radiofrequencies (3 kHz to 300 GHz) from real-world wireless transmitters and signal generators (or other methods used to simulate real-world device signals in a laboratory setting).

**Comparators:** Comparison populations exposed to lower levels, sham exposure, or no exposure (control).

**Outcomes:** DNA damage as depicted in Figure 1 as well as related potential biological mechanisms such as spindle disturbances, free radical production/oxidative stress, heat shock protein expression and apoptosis.

## Methods

The protocol used to generate this evidence map and associated data synthesis follows the Preferred Reporting Items for Systematic Reviews and Meta-Analyses extension for Scoping Reviews (PRISMA-ScR) (32) and Joanna Briggs Institute (JBI) Scoping Review guidelines (33). A full description of the method and protocol applied is available in Supplementary Data Sheet 1.

### Study quality assessment analysis

A quality assessment was performed on studies using specific quality criteria. The assessment adopted and extended the recommended quality attributes used by Vijayalaxmi and Prihoda (30). The specific quality attributes used for the selection of “higher quality” studies covered; waveform specified, exposure duration described, exposure intensity details provided, frequency of signal qualified, dosimetry calculated or measured, blinding/coding used, sham control used, and statistical methods described. A positive control was not judged to be critically important for quality determination, which is a deviation from Vijayalaxmi and Prihoda quality review protocol. While positive controls are desirable for evaluating an assay's sensitivity by comparing effects against a known genotoxic agent, they are not essential for determining cause and effect using experimental logic (34). Studies that incorporated all of these criteria were classified as higher quality (*n* = 130). For more details on quality assessment procedures refer to Supplementary Data Sheet 1.

## Review findings

### Study mapping and presentation

The main evidence for genotoxicity, potential biological mechanisms and funding influences are summarized within an evidence map using tables, graphs, a flow chart, heat maps, and network diagrams. The systematic map database containing the selected articles, including bibliographic information and extracted data, is summarized below, with full details provided in Supplementary Data Sheet 4.

In all graphs and tables, the main analysis presents the proportion of statistically significant damage effects (displayed in **orange**) vs. the proportion of no significant damage (displayed in **gray**). All trends in studies were classified as non-significant effects, and studies showing trends or protective effects were grouped under the “no effect” category, indicating no significant DNA damage.

### Results from search and screening (PRISMA)

**Figure 2:**
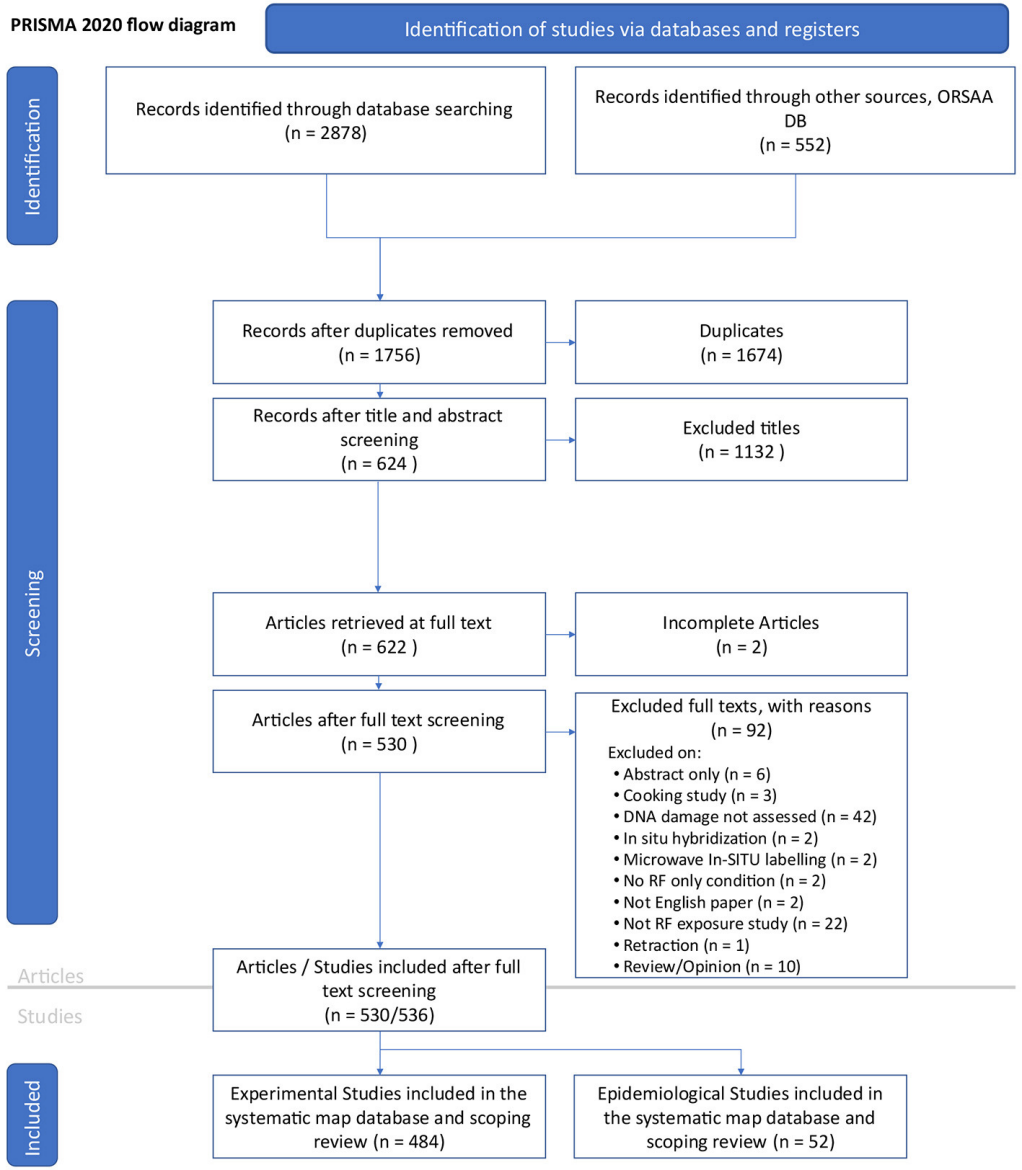
PRISMA 2020 flow diagram (35) for the RF-EMF genotoxicity systemic map. Note six of the 530 articles contained two studies resulting in 536 studies for review.

The PRISMA 2020 flow diagram (Figure 2) provides an overview of the search and screening process. An initial search was conducted on 12th April 2023, starting with the EMF-Portal database. A total of 3,430 candidate records were identified from all literature databases combined. After removing duplicates (*n* = 1674) and non-relevant or incomplete articles (*n* = 1,226), a total of 530 articles remained. Specific details for article exclusions are provided in Supplementary Data Sheet 4 (spreadsheet tab labeled “Review”, columns B:C).

Five articles reported on both *in vitro* and *in vivo* studies and one article covered an epidemiological and *in vitro* study (bringing the total number of studies to review *n* = 536). Of these, nineteen studies (6% of *in vitro* and 1% of *in vivo*) reported only “possible” DNA damage and were thus excluded from the main balance-of-evidence mapping process (leaving *n* = 517 studies for review).

## Geographical location and history of publications

**Figure 3:**
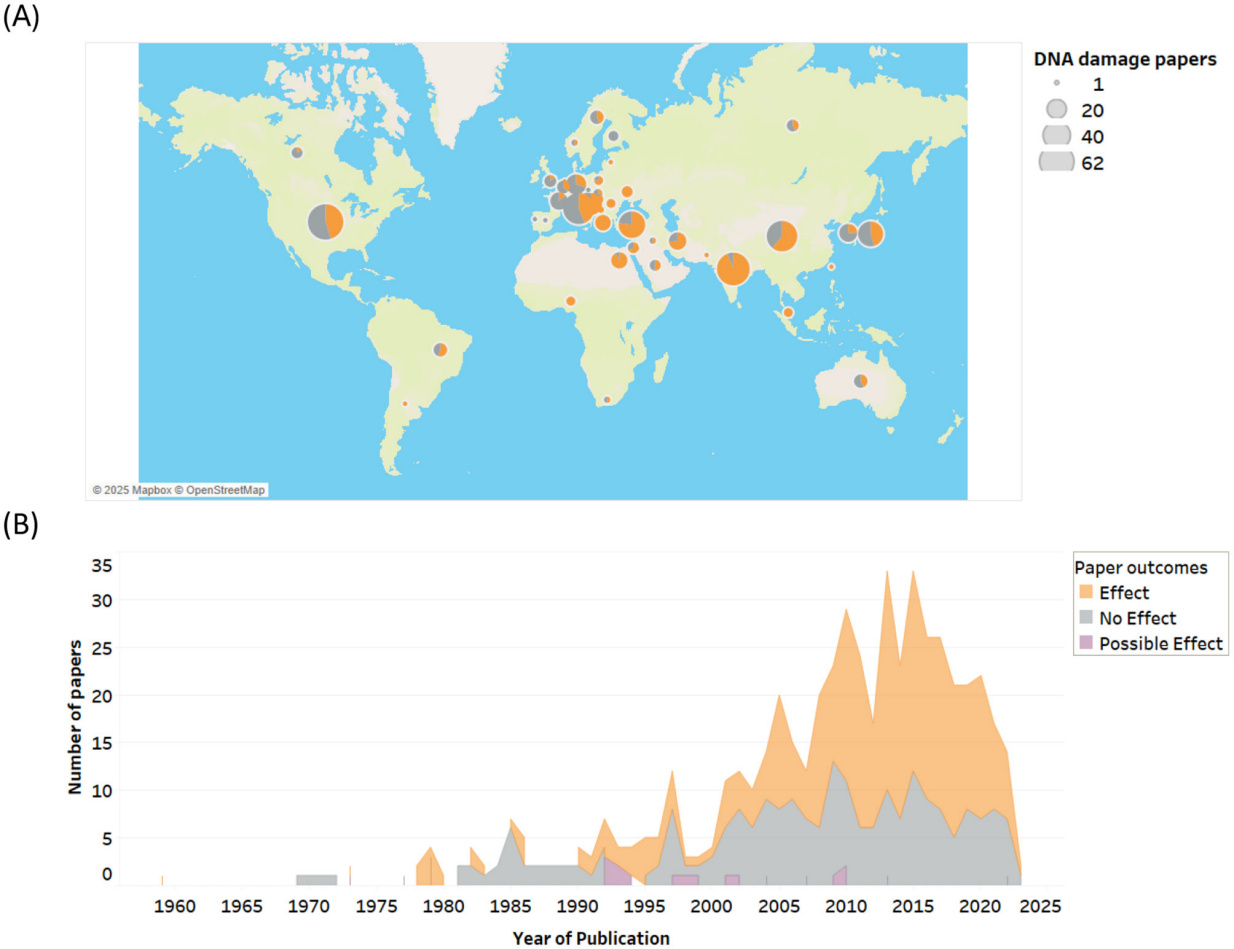
**(A)** RF-EMF genotoxicity research outcomes by country and **(B)** by date of publication.

Research on RF-EMF genotoxicity has been conducted globally, with the USA, China, India, Italy, Japan, and Turkey emerging as the leading countries in this area (Figure 3A). A more detailed breakdown by country and balance of evidence is provided in Supplementary Table 2.

Over 500 studies on RF-EMF induced genotoxicity, and to a lesser extent mutations, have been published since 1959 (Figure 3B). The rate of research publications remained relatively steady from the late 1970s to 2000, then surged over the next 15 years, coinciding with the increasing adoption of wireless devices, the development of supporting infrastructure, and rising public health concerns.

## Organism and cell types

Genotoxicity research has primarily focused on mammals, with non-mammalian organisms receiving less attention. Except for microbes, plants, and insects (which have over 10 studies for some DNA damage endpoints), many organisms are either understudied or not studied. Mammalian studies (448 combined) have primarily focused on human research (227 studies), with the majority being *in vitro* and epidemiological studies, with fewer *in vivo* studies. Rats are the next most prevalent (102 studies), then mice (78 studies) and other mammals (41 studies), including a small number of studies involving bovines, canines, felines, hamsters, and rabbits (see Supplementary Table 3). The research also covers a diverse range of microbe studies (*n* = 33), including bacteria, yeast and bacteriophage studies (all *in vitro*), which have shown mixed results (see Supplementary Table 4).

Human studies showed a near-even split (51 vs. 49%) in the balance of evidence, with *in vivo* and epidemiological research consistently reporting statistically significant DNA damage, compared to more null results for *in vitro* studies. Rats (75%) and other mammals (73%) showed similar trends. While the outcomes of mouse studies are generally aligned with those of human studies, a lower proportion of *in vitro* and *in vivo* mouse studies reported DNA damage. In contrast, all studies on plants, worms, birds, and amphibians reported statistically significant DNA damage. Similarly, 71% of insect studies identified statistically significant DNA damage effects.

### Organism vs. DNA damage type and mechanism

Approximately 80–100% of the non-mammalian studies demonstrated statistically significant effects, particularly DNA base damage and oxidative stress (Table 1). Spindle disturbances, a potential mechanism for DNA damage, were found in 100% of studies for both mammals and plants. Although smaller in number (so conclusions are less certain), studies with plants, insects, worms, and birds have high proportions of outcomes showing RF-induced DNA damage and oxidative stress. Given the limited number of studies on amphibians, worms and snails, robust conclusions are not possible.

**Table 1:** Organism vs. genotoxicity/mechanism studies: number of studies (% effect findings) – shading intensity set on # papers.

**DNA Damage End Point**	**Organism**							
	**Microbes**	**Plants**	**Molluscs**	**Worms**	**Insects**	**Mammals**	**Birds**	**Amphib's**
DNA breaks/ fragmentation	16 (69)	4 (100)	1 (0)	2 (100)	13 (92)	238 (55)	5 (100)	2 (100)
DNA base damage	1 (100)	0	0	1 (100)	0	38 (84)	2 (100)	0
Chromosome aberrations	1 (100)	16 (94)	0	0	2 (100)	81 (57)	0	0
Micronuclei induction	0	9 (100)	0	0	0	130 (50)	0	0
Sister chromatid exchange	0	0	0	0	0	25 (12)	0	0
Mutations	17 (29)	2 (100)	0	1 (100)	8 (38)	18 (39)	0	0
Spindle disturbances	0	5 (100)	0	0	0	5 (100)	0	0
DNA conformation changes	14 (93)	0	0	0	2 (100)	28 (93)	0	0
Free radicals/oxidative stress	2 (100)	4 (100)	0	2 (100)	7 (100)	101 (80)	2 (100)	1 (100)
Apoptosis	0	0	0	0	7 (100)	104 (56)	1 (100)	0
Heat shock proteins (HSPs)	0	0	0	1 (100)	1 (0)	25 (44)	0	0

### Damage by cell type

*In vitro* studies primarily focus on cellular damage across various organisms. Similarly, *in vivo* and certain epidemiological studies also examine specific cell types. When statistically significant DNA damage is observed in > 50% of studies for particular cell types, they are deemed more sensitive to RF-EMF exposures.

Genotoxicity findings for different cell types revealed varying sensitivities to RF exposure (see Table 2). Reproductive cells were highly sensitive, with statistically significant DNA damage effects in 80% of 20 ovarian, 80% of 30 testicular, and 74% of 27 spermatozoa studies. Insect larvae (75% of 4 studies) and embryos (55% of 20 studies) also showed differing levels of susceptibility. Normal brain cells were found to be very sensitive (76% of 54 studies), whereas neoplastic brain cells exhibited lower sensitivity (33% of 12 studies). A moderate proportion of liver and lung studies (56% of 16 studies) found damage, as well as studies of buccal (oral) mucosa cells (65% of 23 studies). This raises potential concerns due to mobile phone technology changes, which relocated the main antenna from the top of the phone to the bottom, nearer to the mouth (36).

**Table 2:** Proportion and type of cell studies finding DNA damage (shading intensity is set for % of effects).

**Cell type (tissue or organ)**	**Number of studies (% effects)**	**Cell type (tissue or organ)**	**Number of studies (% effects)**
All cells	503 (59)	Embryonic	20 (55)
Lymphocytes^*^	95 (47)	Eye (lens/cornea)	12 (33)
Leucocytes^*^	20 (30)	Liver	16 (56)
Erythrocytes (blood)	21 (67)	Lung	16 (56)
Erythrocytes (bone)	15 (53)	Ovary	20 (80)
Bone marrow	10 (30)	Skin	15 (33)
Brain neurons (cancer)	12 (33)	Spermatozoa	27 (74)
Brain neurons (normal)	54 (76)	Testicle	30 (80)
Buccal mucosa	23 (65)	Larvae	4 (75)
Cell line	119 (48)	Primary Cell	396 (62)

^*^Leukocytes (also called white blood cells) include lymphocytes, granulocytes, and monocytes. Studies that explicitly name lymphocytes have been captured on their own and not included in the Leukocyte study count (%).

Blood cells showed greater tolerance to RF-induced genotoxicity, particularly lymphocytes (47% of 95 studies) and leukocytes (30% of 20 studies), which were analyzed separately. However, this tolerance may be limited because longer exposures, particularly in real-world settings (e.g., epidemiological studies), showed statistically significant DNA damage across all blood cell types. On the other hand, 67% of 21 erythrocyte studies found evidence of genotoxicity. Some studies investigating genotoxicity on human blood (37–39) also demonstrate the existence of individual sensitivities and responses to radiofrequency exposures, which should not be confused with electromagnetic hypersensitivity (EHS). Some studies pooled their results, washing out potentially sensitive individual data (40–42).

Eye and skin tissues were studied less frequently, with only 33% of 12 and 15 studies, respectively, showing statistically significant DNA damage findings. Similarly, bone marrow (30% of 10 studies) showed less damage. Primary cells were more likely to present DNA damage than cell lines. These findings reveal the variability in cell sensitivity.

## Study type and DNA damage findings

### Evidence distribution by study type (*in vivo, in vitro*, epidemiological)

**Figure 4:**
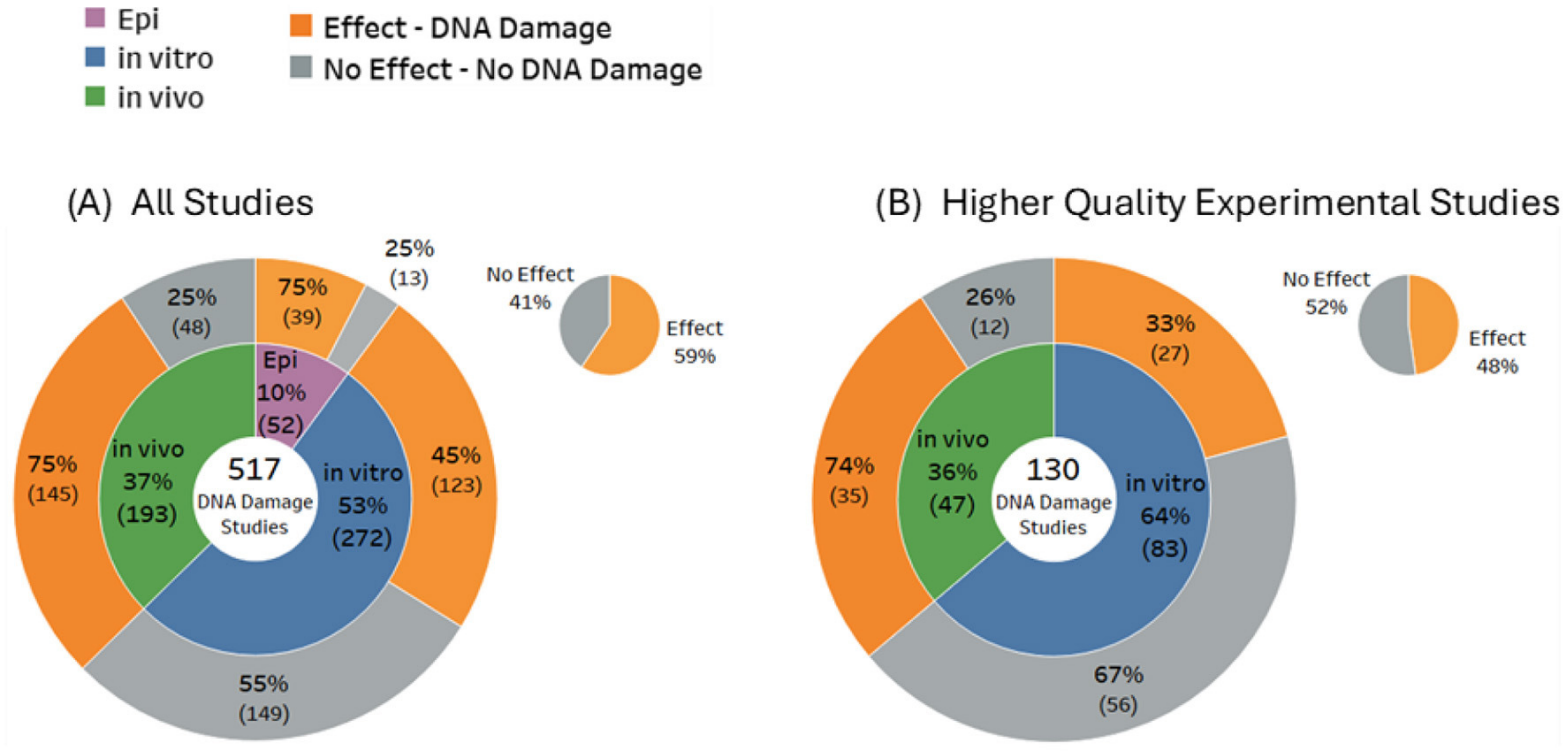
Overall balance of evidence for **(A)** DNA damage (all studies) and **(B)** higher quality experimental studies.

Of the 517 included studies that were confirmed to investigate actual DNA damage, the majority were *in vitro* studies, comprising 53% (*n* = 272) of all studies. In comparison, only 37% (*n* = 193) were *in vivo* studies, and a smaller 10% (*n* = 52) were observational (epidemiology) studies. There were five papers that investigated both *in vitro* and *in vivo* outcomes. The overall balance of evidence for DNA damage was 59% Effects compared to 41% No Effects (Figure 4). Outcomes for *in vitro* studies were slightly weighted toward No Effect (55%), whereas the majority of both the *in vivo* studies (75%) and the epidemiological studies (75%) reported statistically significant DNA damage. For higher quality studies, the balance of evidence for overall DNA damage shifted in favor of No Effects (52%) vs. Effects (48%).

These results reveal substantial variability in the potential for RF-EMF to induce genetic damage. *In vitro* studies, which dominate the evidence base, predominantly indicate no significant DNA damage. In contrast, an identical proportion of *in vivo* and epidemiological studies primarily indicate genotoxic effects. These opposing outcomes from studies of live organisms vs. isolated cells highlight the limitations of *in vitro* models for replicating the complexity of living organisms, particularly animals (44).

Animals are comprised of interconnected systems, including the nervous, endocrine, and immune systems, that can all influence cellular functions and responses (45). These systems mediate intricate feedback mechanisms, hormonal signaling, and immune responses (46). Furthermore, *in vivo* models include physiological processes and interactions such as blood flow, metabolic activity, and tissue-level interactions that can influence RF absorption and its biological effects (47, 48).

*In vitro* systems, however, are devoid of these regulatory influences, interacting systems and processes, providing a limited and potentially misleading perspective on how cells respond to RF exposure in a living organism. Additionally, the simplified environment of *in vitro* studies may lead to an underestimation of RF-induced genotoxic effects. To fully understand the biological impact of RF exposures, *in vitro* studies must be interpreted cautiously and validated through *in vivo* research.

Epidemiological research on the other hand, reflects real-world exposure conditions and indicates the consequences of long-term exposures. However, as the research map shows (Figure 4), epidemiological research is less common, potentially due to complexity, costs, and time requirements. Evaluating *in vivo* data and epidemiological data together results in a holistic view and resilient confirmation. Therefore, more weight should be given to these study types when assessing the health risks of RF exposure.

### Types of DNA damage

**Figure 5:**
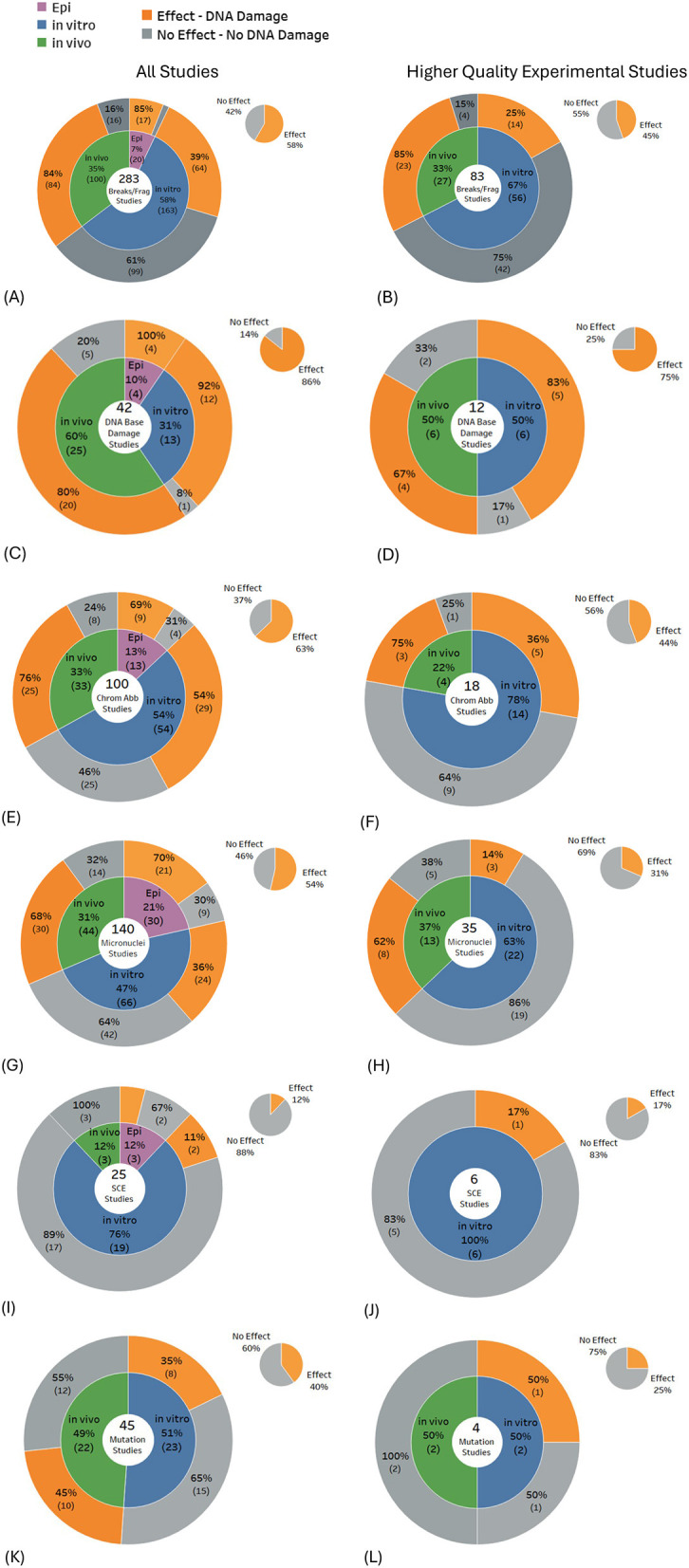
Results for types of DNA damage: **(A, B)** DNA breaks/fragmentation, **(C, D)** DNA base damage, **(E, F)** chromosome aberrations, **(G, H)** micronuclei, **(I, J)** sister chromatid exchange and **(K, L)** mutations.

Statistically significant DNA damage was prevalent in studies investigating DNA breaks (58% of 283 studies), DNA base damage (86% of 42 studies), chromosome aberrations (63% of 100 studies), and micronuclei induction (54% of 140 studies). DNA base damage was found in the majority of studies for all study types: 92% of 12 *in vitro* studies, 80% of 20 *in vivo* studies, and 100% of 4 epidemiological studies (Figure 5C), and DNA breaks/fragmentations were found in the majority of *in vivo* studies (Figure 5A). Conversely, the balance of evidence was weighted toward no significant effects for studies investigating mutations (60% of 45 studies) and sister chromatid exchange (88% of 25 studies).

When only the higher-quality studies were selected from the above DNA damage subtypes, results typically showed < 50% of studies reporting damage, except for DNA base damage, where 75% of the 12 higher quality studies produced statistically significant findings. The largest shift in the balance of evidence across most DNA damage subtypes occurred for *in vitro* experiments, where, for higher-quality studies, the proportion showing no effects increased markedly. In contrast, the balance of evidence for *in vivo* studies remained largely unchanged (see Figures 5A–L, gray regions of each pie chart).

### Further indicators of possible DNA damage

**Figure 6:**
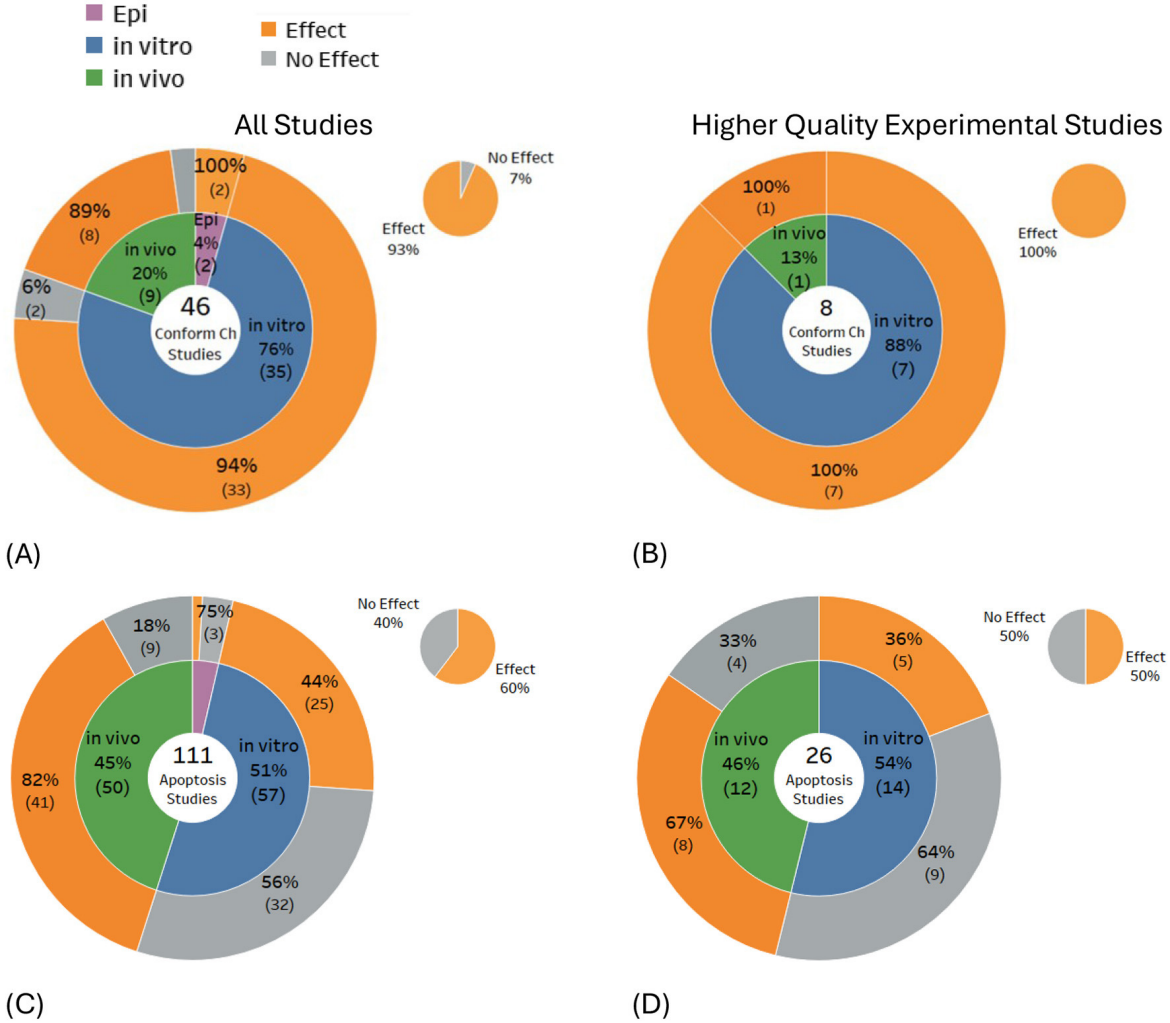
Results for indicators of potential DNA damage: **(A, B)** DNA conformational change and **(C, D)** apoptosis.

Some biological changes observed suggest DNA damage, but with no clear one-to-one correspondence between the occurrence of these biological changes and DNA damage. The evidence for these possible indicators was not included in the main systematic mapping detailed above, but instead, is presented in Figures 6A–D for completeness. Studies investigating these indicators predominantly found DNA conformational changes (93% of 46 studies) and cell apoptosis (60% of 111 studies). Selecting only the higher-quality studies had minimal impact on the balance of evidence for DNA conformational change, i.e., findings increased (100% of eight studies), but for apoptosis studies, findings decreased (50% of 26 studies). DNA conformational changes showed a consistently higher proportion of studies showing DNA alteration effects for both *in vitro* and *in vivo* higher quality studies, whereas for apoptosis, statistically significant findings decreased for both *in vitro* and *in vivo* studies.

### Synergistic, additive and protective effects

*Additive, synergistic, antagonistic, and potentiative effects can be observed and is dependent on the sequence of exposure (prior, follow, or simultaneously) and the initial functional state of the exposed biological system* [(49), p. 915].

Studies were conducted to investigate adaptive or protective cell responses to radiofrequency exposures in conjunction with known genotoxic agents. This was contrasted with studies showing synergistic effects. The presence of some agents (such as gamma rays) in both categories underscores the pivotal influence of the timing and duration of an RF exposure in determining whether the cellular response is likely to be protective or synergistic (damaging) (Supplementary Table 11).

In some cases, the published data indicates that exposures to RF-EMF can produce beneficial protective effects against other genotoxic agents, suggesting the induction of an adaptive response at both the cellular and organism level (50). Potential biological mechanisms underlying these protective effects include the activation of signaling pathways, upregulation of specific gene expression and protein synthesis (e.g., HSPs, antioxidant enzymes, cell repair), enhanced activity of the DNA repair system, and a reduction in free radical levels (51).

Although the pool of studies investigating combinative and synergistic effects was relatively small, it reveals how RF exposures can have potential therapeutic benefits (cancer treatments) or can enhance the harm caused by ionizing radiation and chemical agents, depending on exposure conditions. Unfortunately, current international RF Guidelines do not consider harm caused by synergistic interactions of RF with other genotoxic agents (52).

## Exposure signal characteristics

The relationships between exposure characteristics and genotoxicity findings are mapped out in the sections below.

### Exposure frequency

**Figure 7:**
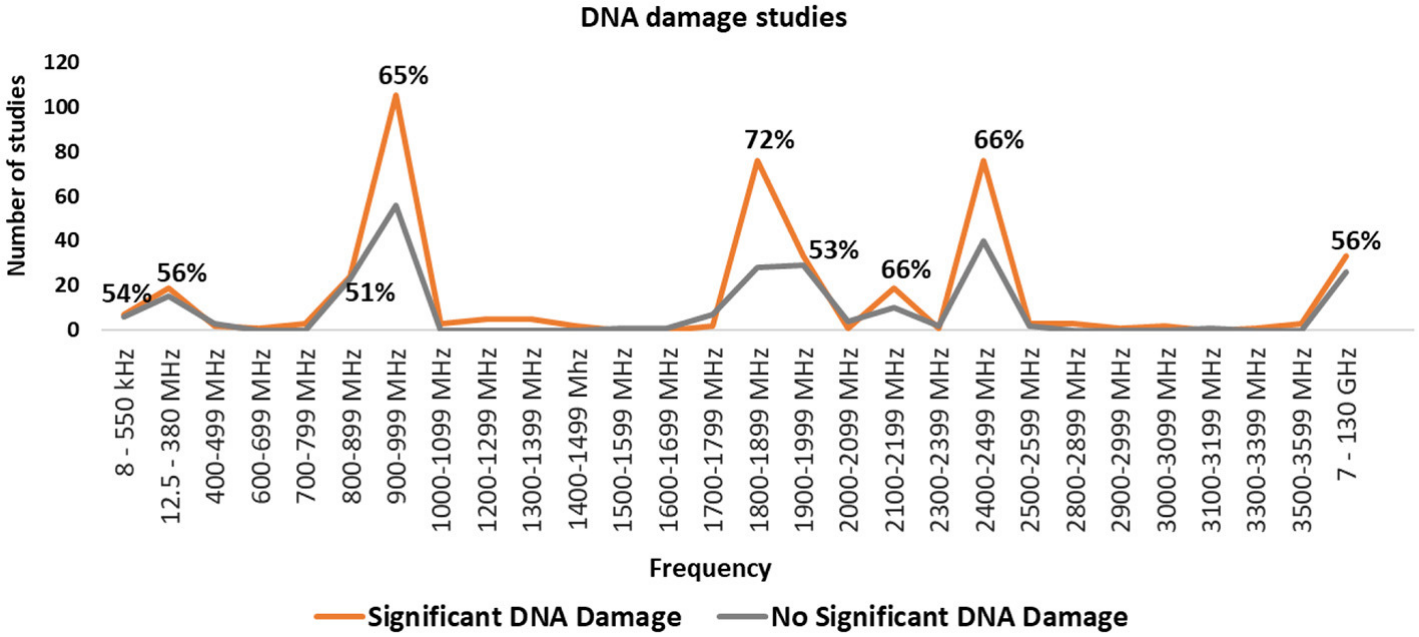
Number of studies in each frequency band and percentage of studies finding statistically significant DNA damage for the frequency bands where there were more than 10 studies.

Figure 7 summarizes research and genotoxicity findings by frequency band. The most studied bands include 900–999, 1800–1899, 1900–1999, and 2400–2499 MHz, which align with common frequencies used by mobile phones, base stations, microwave ovens, and Wi-Fi. The balance of evidence favors DNA damage effects for the 900, 1800 and 2450 MHz frequency bands. However, results for 1900 MHz, are equivocal.

Additional studies covering MHz and GHz ranges, corresponding to FM broadcasts, magnetic resonance imaging (MRI), radar, and satellite communications, are listed in Supplementary Figure 3. However, many wireless communication frequencies, especially those used by 5G new radio, remain under-researched. Consequently, novel frequencies and modulation schemes are being deployed without adequate testing for genotoxicity or broader health implications.

### Exposure duration

The data was investigated for the effects of exposure duration on study outcomes. The proportions of studies showing statistically significant DNA damage effects were graphed across a wide range of exposure durations (1 min to 1 year) for all exposure brackets containing five or more studies.

**Figure 8:**
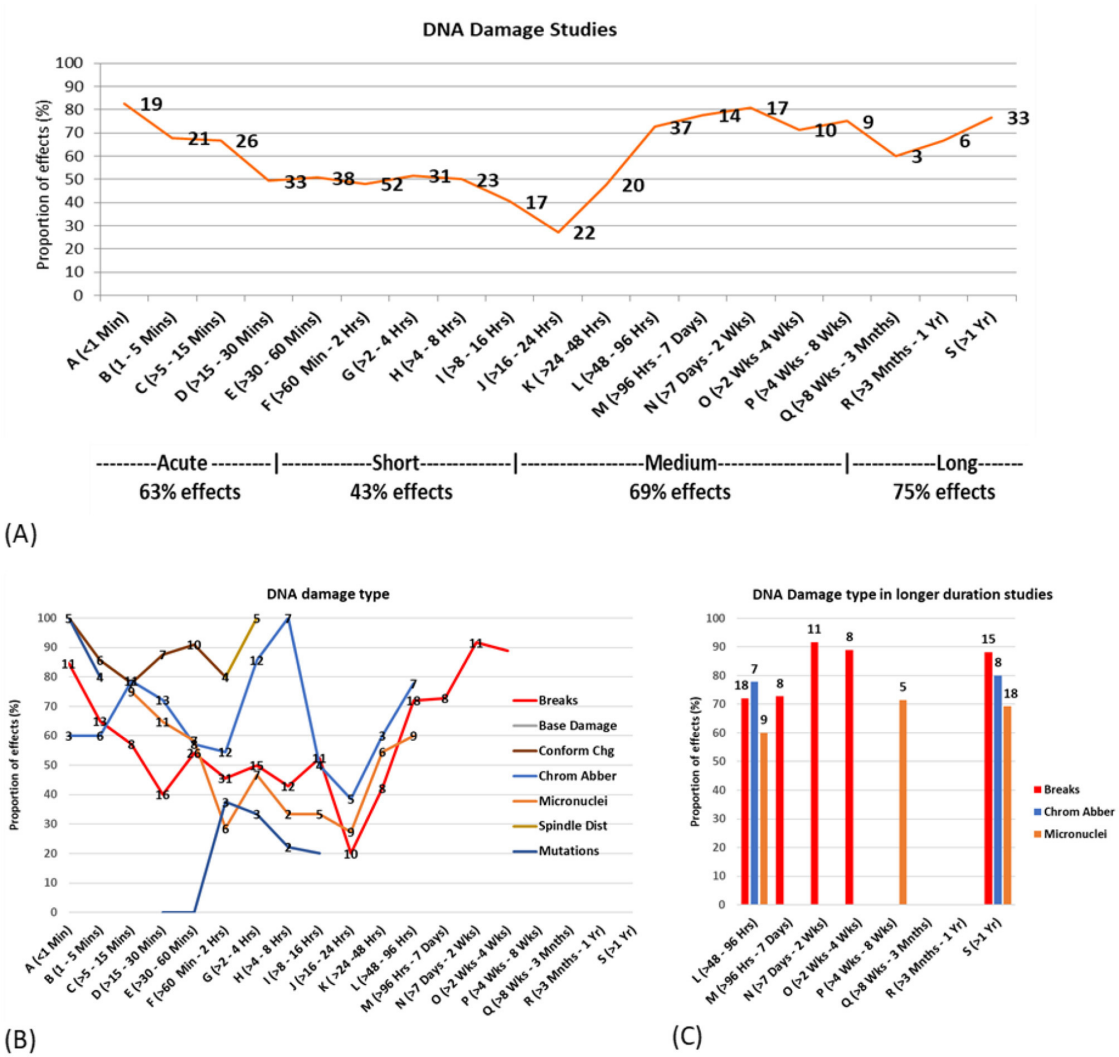
DNA damage vs. exposure time **(A)** proportion of statistically significant DNA damage studies for each time bracket, with the number of studies for each bracket overlayed on the line. **(B)** Proportion of studies showing effects by type of DNA damage **(C)** Proportion of studies showing effects and DNA damage type for study duration 2 days or more.

Figure 8A suggests a crude U-shaped dose response relationship, with over half of the studies in each time bracket showing effects for both short (less than half an hour) and longer exposures (more than 2 days), while less than half of the studies using exposure durations between half an hour and 2 days showed effects.

Both *in vitro* and *in vivo* studies showed the least amount of DNA damage in studies in the 16–24 h time bracket where < 30% of studies showed DNA damage. More than 50% of *in vitro* and *in vivo* studies showed statistically significant DNA damage for exposures < 15 min and >96 h. The range of exposure time where >50% *in vivo* studies showed damage is also lot broader than for *in vitro* studies (see Supplementary Figures 17a, 18a).

The exposure duration outcomes were then investigated according to specific DNA damage type. Figure 8B illustrates the percentage of studies reporting effects, each suggesting a bi- or tri- phasic response curve for most DNA damage types. This provides evidence for potential cellular adaptive responses, particularly in the 1–2 h and 16–24 h time bands, where a greater proportion of studies reported no effects. Figure 8C focuses on the existing studies using longer-term exposures (2 days or more), showing high proportions of statistically significant effects for DNA breaks, chromosome aberrations and micronuclei induction.

### Exposure duration categories

**Figure 9:**
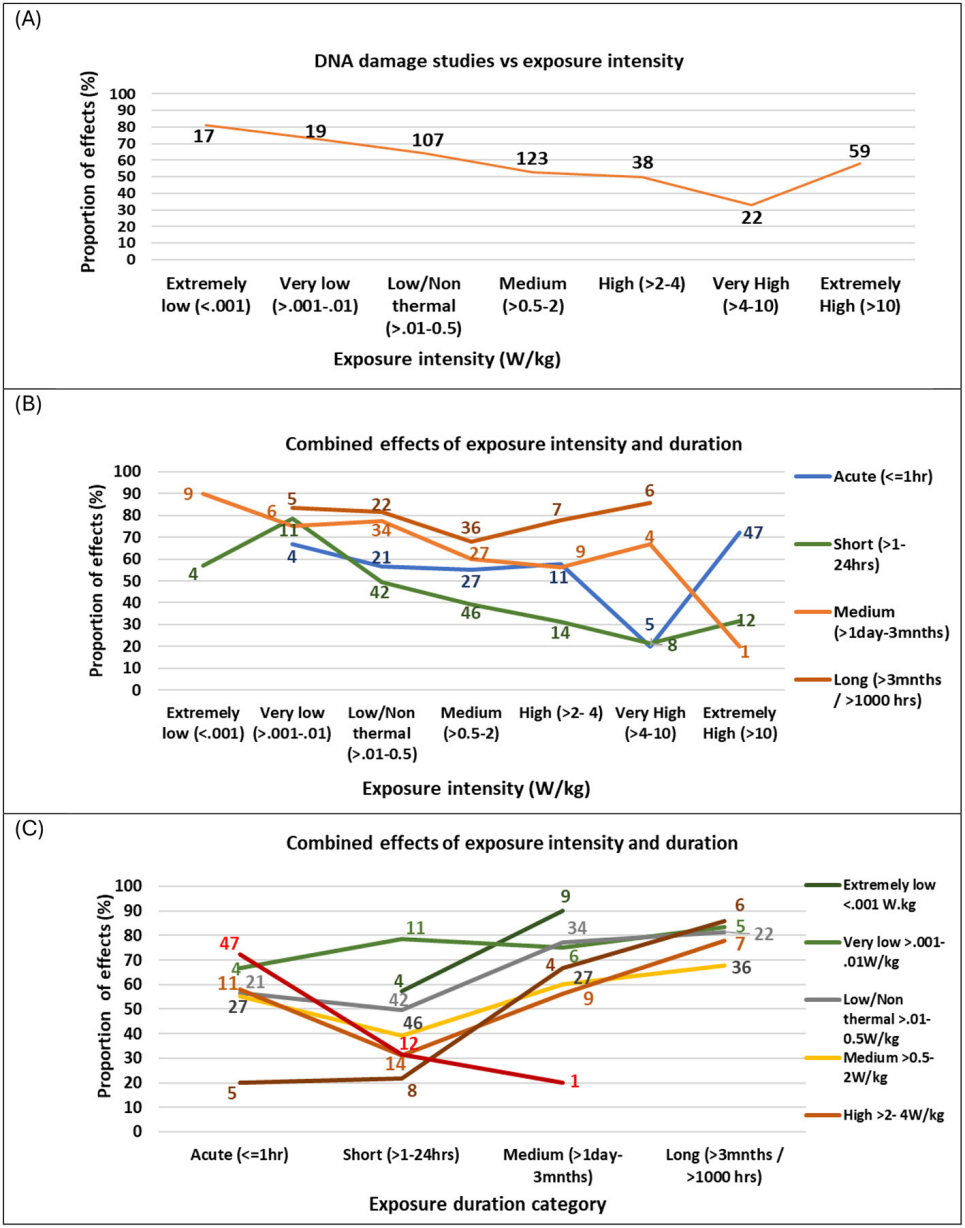
Proportion of studies showing **(A)** DNA damage effects vs. exposure intensity, overlaid with number of studies **(B)** exposure duration vs. exposure intensity and **(C)** exposure intensity vs. exposure duration. Graphs only show data where there were 5 or more studies in that category combination.

To simplify the analysis, exposure durations were categorized into four groups: acute (167 studies), short-term (243 studies), medium-term (108 studies), and long-term (70 studies) (see Figure 9C). Most experiments (70%) focused on short-term and acute exposures, with medium- and long-term exposures being less studied (30%). *In vitro* studies primarily focused on acute and short-term exposures, while *in vivo* studies encompassed all exposure durations, and epidemiological studies concentrated on long-term exposures. DNA damage was reported in 63% of acute, 43% of short-term, 69% of medium-term, and 76% of long-term studies (see Supplementary Table 12 for further details). These exposure duration categories were used for analyzing duration in combination with other factors, as described below.

### Exposure intensity and duration

The data was investigated for the effects of exposure intensity on study outcomes by plotting the proportion of studies showing effects for a range of exposure intensity windows (extremely low to extremely high). Figure 9A illustrates that the strength of evidence for DNA damage is moderated by intensity of exposure in a non-linear manner. Most studies have focused on near-field, low- to medium intensity exposure levels (>0.01–2.0 W/kg), typical of mobile phone use, where half or more of these studies found DNA damage effects. Fewer studies have examined higher exposure intensities (e.g., occupational settings) or extremely low intensities, which are relevant to far-field sources such as base stations, smart meters, radar, and Wi-Fi routers.

Studies investigating extremely low exposure levels (< 0.001 W/kg) reported the highest proportion of genotoxic effects (81% of 21 studies). Moreover, the proportion of statistically significant DNA damage findings decreased as intensity increased. However, at extremely high intensities (>10 W/kg), which exceed ICNIRP limits, the proportion of statistically significant DNA damage effects rose again (58% of 59 studies) (see Supplementary Table 13). These results suggest that DNA damage does not follow a linear dose-response pattern, and also suggesting that non-thermal mechanisms are likely to play a significant role in RF-induced DNA damage. Subsequently, exposure intensity was further explored by categorizing exposure relative to ICNIRP occupational limits. Surprisingly, studies at or below ICNIRP limits showed a slightly greater proportion of statistically significant genotoxic effects (58% of 439 studies) than those above the limit (54% of 92 studies) (see Supplementary Table 13).

Exposure intensity and duration were further explored to determine their combined effect on genotoxicity findings. Figure 9B shows the breakdown of Figure 9A into exposure duration categories where there are five or more studies investigating that exposure-duration combination.

The proportion of studies showing effects varied by exposure duration and intensity, following a pattern similar to Figure 9A. For studies with acute (< 1 h) and short (1–24 h) exposure durations, a high proportion showed effects at extremely low (< 0.001 W/kg) and very low (0.001–0.01 W/kg) intensities, but this proportion decreased as intensity increased. These studies reached a minimum at very high intensities (4–10 W/kg), with fewer studies showing effects, followed by an increased proportion of effects at extremely high intensities (>10 W/kg). For medium-duration studies (1 day−3 months), the proportion of studies showing effects reached an initial low point at high intensities (2–4 W/kg) but remained above 50%. At extremely high intensities (>10 W/kg), only one in five medium-duration studies showed effects. In contrast, long-duration studies (>3 months) exhibited a different trend: the proportion showing effects was lowest at medium intensities (0.5–2 W/kg) but increased at higher intensities. Notably, long-duration studies consistently showed effects in over 65% of cases across all intensity levels.

Altogether, the pattern of effects varied in a U-shape pattern across study duration, with the highest proportion of effects for long (>3 months) and then medium (< 1 day−3 months) duration studies, fewer effects for short duration studies (1–24 h) and a greater proportion of effects again for acute (< 1 h) durations. Figure 9C depicts this relationship more clearly, showing that for all exposure intensities, the proportion of studies showing effects was at a minimum for short exposure durations (1–24 h). Therefore, two U-shaped dose-response patterns emerged, one for intensity and one for duration, interacting with one another to give an overall non-linear and non-monotonic dose response pattern. This complex interaction confirms earlier indications by Lai and Levitt 2022 (43) of non-linear response patterns for both intensity and duration. It reveals that intensity alone is not the only important factor determining outcomes, and that the duration of exposure is a crucial, moderating factor. Moreover, linear models are not appropriate for describing results in this field.

These findings challenge the oversimplified ‘no effects' conclusions of some past reviews, which may have failed to account for the complex interplay between exposure intensity and duration. They also give direction for future research, to further explore these non-linear relationships.

### Modulations and simulated signals

Studies were investigated for the effects of different telecommunication signal modulations (see Supplementary Table 15). A greater proportion of studies using real mobile phone signals found DNA damage, except for GSM-Basic and CDMA communication protocols, where the small number of studies limits the ability to draw reliable conclusions. Studies using simulated signals were less consistent, with 50% or less showing evidence of genotoxicity across most modulation protocols. One exception, GSM-Talk, showed statistically significant DNA damage for 67% of 15 studies using simulated signals as well as 91% of 11 studies using a real signal. UMTS and Wi-Fi signals exhibited strong genotoxicity evidence from real signals (88% and 75% of 8 and 12 studies, respectively), but less evidence for effects (17% and 33% of 29 and 5 studies, respectively) for simulated signals. Overall, these results demonstrate that signal modulation can impact study outcomes and highlight the potential limitations of using simulated signals to evaluate genotoxic risks.

### Pulsed vs. continuous signals

Pulsed and continuous waves both showed a greater proportion of DNA damage for *in vivo* and epidemiological studies and a lower proportion for *in vitro* studies. Continuous waves produced a greater proportion of DNA damage than pulsed waves for *in vitro* experiments (but still < 50% of 116 studies). When all studies are combined, both pulsed and continuous wave studies tend toward greater DNA damage. Multiple exposures, which involve separate repeated exposures on a single day or over several days, were found to yield more positive findings than a single exposure.

Comparisons of exposure patterns i.e., continuous, intermittent (e.g., 5 min on, 10 min off), and variable (dynamically changing intensity, such as during mobile phone use) reveal a progressive increase in the proportion of DNA damage findings: 55% of 369 studies for continuous, 62% of 60 studies for intermittent, and 76% of 96 studies for variable exposures.

Further effects of signal characteristics on outcomes are presented in Supplementary Tables 15–17. Across all exposure characteristics, *in vivo* and epidemiological studies consistently report a higher proportion of DNA damage than *in vitro* studies.

### Patterns in exposure characteristics

Altogether, the results reveal that the relationship between exposure intensity and DNA damage varies non-linearly with exposure duration. The evidence is heavily weighted by short-term, *in vitro* studies, where adaptive responses appear to be occurring. Signal type, frequency, and exposure patterns showed significant influences on study outcomes. Commonly used bands like 900–999, 1800–1899 and 2400–2499 MHz, used by mobile phones, smart devices, and Wi-Fi showed greater proportions of effects. 5G bands, on the other hand, are under-researched. Pulsed waves produced a greater proportion of effects for *in vivo* and epidemiology studies. Real-world devices consistently showed a greater proportion of effects than signal generators.

Altogether, the exposure characteristics more likely to produce DNA damage effects are those that describe authentic exposure conditions. These findings underscore the importance of future studies to incorporate diverse frequencies, realistic exposure patterns, and emerging technologies.

## Potential biological mechanisms

### Mechanisms of DNA damage

**Figure 10:**
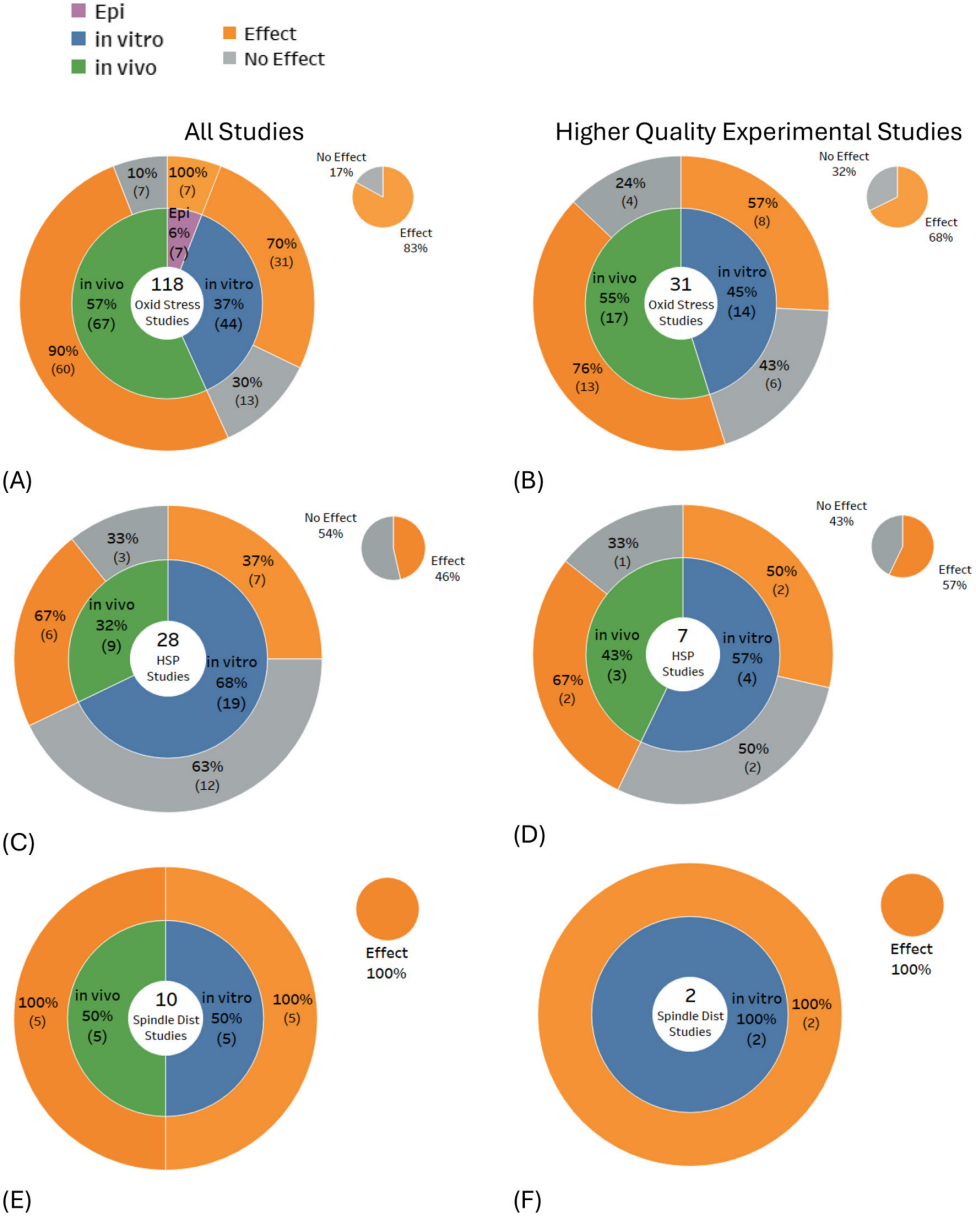
Results for potential biological mechanisms of DNA damage: **(A, B)** free radicals/oxidative stress, **(C, D)** heat shock protein expression/levels and **(E, F)** spindle disturbances.

A review of studies investigating potential biological mechanisms (Figures 10A–F) revealed that free radical production or oxidative stress was the most frequently studied endpoint, with most studies showing effects (83% of 118 studies). Heat shock proteins were found to be expressed in just under half of the studies (46% of 28 studies), and spindle disturbances were found in all the relevant studies (100% of 10 studies). For higher-quality studies, the balance of evidence remained weighted toward effects for free radical production/oxidative stress (68% of 31 studies) and tilted toward effects for heat shock protein expression (57% of seven studies) and remained unchanged for spindle disturbances (100% of two studies).

### Mechanisms and exposure duration

The proportion of studies showing effects for potential DNA damage mechanisms was compared for various exposure time intervals. Only those time intervals with five or more studies (for effects or no effects combined) were analyzed.

**Figure 11:**
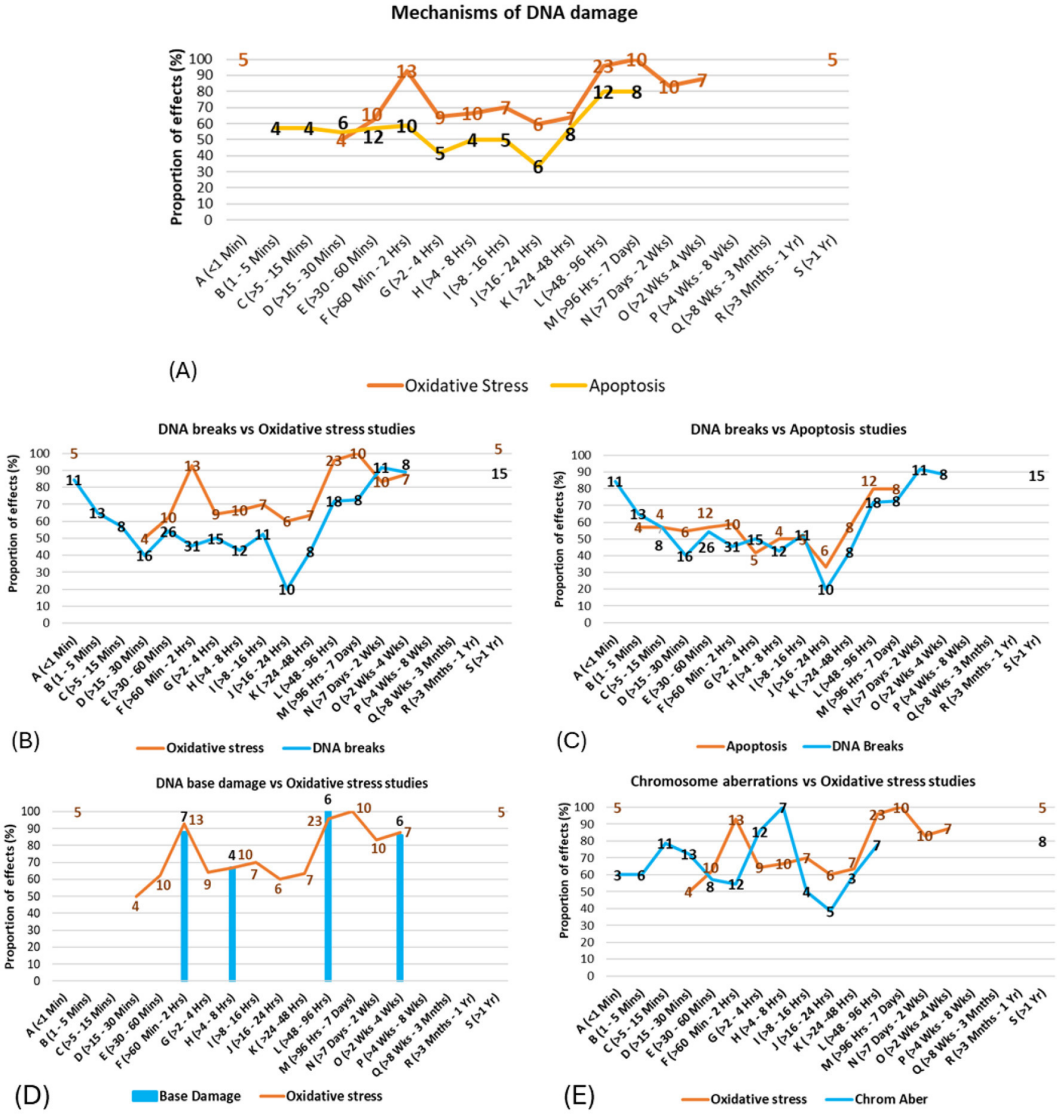
**(A)** Proportion of mechanisms showing damage vs. exposure time intervals (the numbers of studies showing effects are shown on the line); **(B–E)** Correspondence between patterns of evidence for mechanisms and patterns of evidence for DNA damage types across exposure time intervals.

When mechanisms were investigated across exposure time intervals, a bi- or tri- phasic response pattern was suggested (see Figure 11A), where free radical production/oxidative stress effects were dominant in studies using 30 min to 2-h exposures, and free radical production/oxidative stress and apoptosis were dominant in studies using exposure durations longer than 2 days. These results follow the same patterns of effects for DNA damage types shown in Figure 11 (also see Supplementary Figures 21–27 for more details). The possible relationships between the underlying mechanisms for DNA damage and the type of DNA damage were subsequently explored by overlaying the proportion of statistically significant evidence for each across the various exposure durations.

For example, studies on free radical production/oxidative stress and DNA breaks show similar patterns of results across the time intervals (Figure 11B), suggesting that free radical production/oxidative stress may be causally related to DNA breaks. Similarly, Figure 11 suggests strong correspondences between (C) apoptosis and DNA breaks/fragmentations, and correspondences between (D) free radical production/oxidative stress and DNA base damage.

Potential connections were also observed between (E) free radical production/oxidative stress and chromosome aberrations, with a time lag between the former and the latter; however, more evidence is needed to strengthen this conjecture. Possible further associations were also observed between spindle disturbances and chromosome aberrations, chromosome aberrations and micronuclei induction, and between free radical production/oxidative stress and both chromosome aberrations and micronuclei induction (see Supplementary Figures 23–26 for full details). The correspondences between the patterns of evidence for mechanisms and DNA damage effects in specific time bands support the finding from Figure 9 above, as well as existing theory suggesting that effects from EMF-RF exposures are stronger for certain time windows (53, 54).

### Free radical production as a potential mechanism

Figure 11B and Supplementary Figure 30 illustrates that a strong correspondence exists between observing free radicals/oxidative stress and finding DNA damage. Evidence of oxidative DNA base damage showed the highest proportion of statistically significant findings among all forms of DNA damage investigated (Figure 5C). Two critical markers for oxidative stress and carcinogenesis (55), 8-oxo-7,8-dihydro-2′-deoxyguanosine (8-oxo-dG) and 8-hydroxy-2′-deoxyguanosine (8-OHdG), were the most frequently measured biomarkers of oxidative DNA damage. Both of these oxidative DNA damage biomarkers are seen as risk factors for many diseases, including neurodegenerative disorders (56) and cancer (57). Failure to promptly remove 8-oxo-dG can result in a base transversion point mutation, where G:C is converted to T:A during DNA replication (56).

Reactive oxygen species (ROS) are free radicals commonly observed as an endpoint in numerous RF exposure studies (58), where they are often associated with oxidative stress. Elevated markers of oxidative stress have been found in individuals with neurological disorders such as Alzheimer's disease and Parkinson's disease, as well as diabetes, cardiovascular diseases, and cancer (59). Small increases in ROS have also been observed to provide therapeutic effects; however, these effects are only evident within narrow windows of exposure intensity (60) and duration.

**Figure 12:**
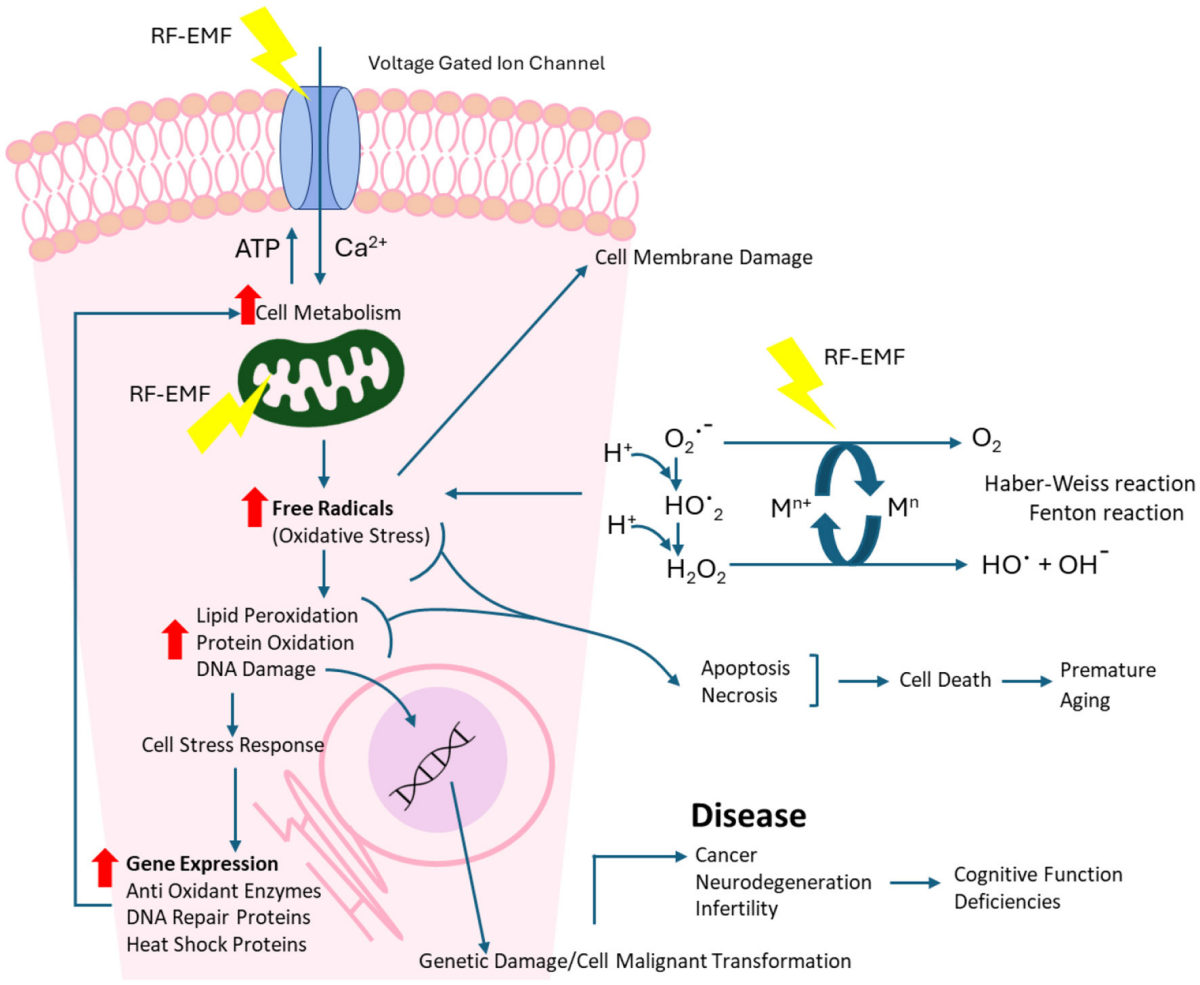
RF-EMF pathway for cellular and DNA damage.

There are several known and potential pathways identified by which RF-EMF exposure can lead to a free radical imbalance in cells (also see Figure 12):

- Mitochondrial dysfunction (61, 62);
- RF enhanced Haber-Weiss and Fenton reactions (H_2_O_2_, OH^−^, Fe^2+^ and Cu^+^ ions) (63–65);
- Microwave interactions with water molecules to form H_2_O_2_ (66, 67);
- Altered antioxidant gene expression (68).

Reactive oxygen species are also implicated in the activation of cellular signaling pathways, including the regulation of the main pathways of apoptosis, particularly the mitochondrial-mediated pathway (69) (see Figure 12). Increased levels of reactive oxygen species have been associated with DNA damage and linked to environmental stress, with anthropogenic radiofrequency exposures being a contributor (70).

### Bi-phasic relationships due to cellular adaptive responses

Cells employ various protective mechanisms when faced with cellular stress, including upregulating DNA repair genes, heat shock proteins, and enzymes that mitigate oxidative stress (71, 72). Gene expression is a sequential process requiring time, starting with signaling pathway activation and transcription factor binding, followed by mRNA processing and protein synthesis (73). The time-dependent activation of cellular stress responses may explain the U-shaped or bi-phasic dose response to RF-EMF exposure observed across increasing exposure durations (Figure 11). A recent study investigating free radical production has confirmed a bi-phasic cellular response to RF-EMF exposure (74).

Acute (very short) exposures to extremely high RF-EMF intensities often result in statistically significant DNA damage, possibly due to the inability of repair mechanisms to respond rapidly. In contrast, longer exposures to high intensities may lead to the activation of cellular defenses to repair damage and attempt to mitigate harm. Furthermore, prolonged or cumulative exposures could lead to accumulated DNA damage, genomic instability, and cellular dysfunction (75). Depending on the extent of the damage, cells may activate autophagy as a protective mechanism to remove damaged components and maintain cellular homeostasis (76, 77). While this may be effective in the short term, if damage persists or overwhelms repair mechanisms, cells may undergo apoptosis to remove severely compromised cells. However, should this survival process fail, there is a risk of malignant transformation (75).

Real-world RF exposures are typically chronic and variable, raising concerns about cumulative effects (78). While repair mechanisms appear to address much of the damage in the short term, prolonged or repeated exposures may overwhelm these defenses, leading to lasting genomic alterations. Given a large proportion of public exposures now occur cradle-to-grave and are often non-consensual, there is a need to address the potential long-term health risks of persistent RF exposure.

### DNA conformational changes

DNA conformational changes are a potential marker for DNA damage. Various methods have been employed in studies to identify changes in DNA conformation, including Raman spectroscopy, electron microscopy observations, circular dichroism, and dynamic light scattering techniques, as well as UV–vis spectroscopy. However, the most common method applied utilized Anomalous Viscosity Time Dependence (AVTD), which provides insights into structural dynamics and molecular interactions within biological systems. In particular, this method demonstrates how DNA-protein complexes (e.g., chromatin) affect the physical characteristics of the DNA, such as viscosity in a solution over time (79).

Chromatin is a DNA-protein complex found in the nucleus of eukaryotic cells. The structure of chromatin can undergo dynamic changes in response to various cellular processes, including DNA repair, transcription, and replication. Proteins such as histones and non-histone chromatin-associated proteins play crucial roles in organizing and regulating chromatin structure (80).

Results demonstrated that RF exposures can cause DNA conformational changes, with a high level of confidence (93% of studies), (see Supplementary Table 9). While the implications of such a change are not fully understood, one possible scenario is the situation where DNA damage becomes inaccessible to repair proteins or repair function is impeded by repair enzyme conformational changes (81).

While AVTD and similar methods provide valuable insights into DNA conformational changes, they cannot easily determine the underlying cause or whether it relates to DNA damage. Integrating AVTD with other experimental approaches, such as a comet assay, can help elucidate whether the observed changes are directly related to DNA damage or the result of normal cellular regulatory processes.

### Relationship between chromosome aberrations, micronuclei and spindle disturbances

Micronuclei, chromosome aberrations, and spindle disturbances are all interconnected indicators of genomic instability and cellular stress (82). The mitotic spindle apparatus is responsible for chromosome segregation during cell division. Spindle disturbances can lead to improper chromosome alignment and segregation, leading to lagging chromosomes or fragments that can become encapsulated and form micronuclei (82).

A positive correlation between micronuclei and particular chromosomal aberrations in human *in vitro* studies has previously been identified, specifically for acentric fragments and dicentric chromosomes (83). Furthermore, micronuclei formation may be induced by chromosomal breakage or inhibition of the spindles during cell division (84).

An examination of the graphs plotting the percentage of studies reporting micronuclei induction, chromosome aberrations and spindle disturbances over exposure time, when overlayed on one another, shows a close relationship validating statements made by past researchers (see Supplementary Figures 23, 25).

### Heat shock proteins

Heat shock proteins (HSPs) are chaperones that protect cell macromolecules. They play a crucial role in both DNA damage repair and the cellular response to oxidative stress (71, 72). Experimental evidence suggests that RF exposure can induce HSP expression, which protects cells, but may also paradoxically aid cancer cell survival by inhibiting apoptosis. RF exposure has been shown to upregulate HSPs in human endothelial cells, potentially contributing to tumor progression (85).

Only 27 studies investigated the relationship between RF-EMF exposure and the expression of heat shock proteins, which included HSP27, HSP70 and HSP90. Notably, apart from extremely high exposure levels, an inverse relationship appears to exist between exposure intensity and detected HSP levels (see Supplementary Table 20). The evidence follows a similar pattern to the exposure-intensity and DNA damage relationship (see Supplementary Figures 20A, B and Supplementary Table 13). However, the small number of studies at some exposure levels prevent reliable conclusions from being drawn.

### Apoptosis: a consequence or cause of DNA damage in RF-EMF studies?

Apoptosis is a normal, tightly regulated process used to eliminate superfluous cells and damaged cells, including cells with DNA damage, to reduce the risk of carcinogenesis (69). The apoptotic process can be initiated by three different signaling pathways (86):

1. intrinsic (facilitated by mitochondria),
2. extrinsic (involving death receptors on the cell surface) and
3. via the endoplasmic reticulum.

Caspase enzymes are crucial mediators of apoptosis, orchestrating the orderly dismantling of cellular components during programmed cell death. Their activation is a hallmark of apoptosis (87), with several studies in this evidence map using caspase assays for the detection of apoptotic events.

Analysis of the results reveals a strong correspondence between DNA damage and apoptosis, as evidenced in (Figure 11C), where numerous studies report both outcomes following RF-EMF exposure. This close relationship raises an important question: in these studies, was apoptosis triggered by excessive DNA damage from RF-EMF, resulting in controlled cell death or was the detected DNA damage the outcome of apoptosis? Both scenarios are plausible. Apoptosis is a cellular mechanism initiated when accumulated damage, including DNA damage, exceeds the cell's capacity to repair and recover (86). The apoptotic biochemical process can include DNA cleavage by activating endogenous endonucleases (88), leading to DNA fragmentation. However, many studies investigating apoptosis did not provide sufficient information to verify the principal initiation mechanism.

## Study quality and outcomes

A striking inconsistency among the reviewed studies was their quality, with numerous omissions of critical details such as complete study methodology, wave properties (e.g., pulsed or continuous waves, modulation applied), sham exposures, blinding, adequate dosimetry, or comprehensive statistical data.

Study outcomes were investigated as a function of study quality. Applying the quality criteria (see Methods and Supplementary Data Sheet 1) shifted the proportion of studies showing DNA damage from 59% of all 517 studies, down to 48% of 130 high quality studies (see Figure 4).

The most important quality criteria were further investigated separately to see how they influenced outcomes for the experimental studies. The results showed that the application of each of the quality criteria individually reduced the proportion of studies showing effects. There was a lower proportion of studies showing effects when studies were blinded (47%) vs. not blinded (64%), when sufficient dosimetry was incorporated (56%) vs. not incorporated (61%), when sham controls were used (50%) vs. only normal controls used (70%). Studies that incorporated all three of these criteria showed a lower proportion of DNA damage outcomes 48% (see Supplementary Table 21).

However, further investigation revealed that this shift toward fewer statistically significant outcomes when quality criteria were applied was only true for *in vitro* studies. There was very little change in outcomes when quality criteria were applied to *in vivo* studies. In contrast, when the waveform was specified, the proportion of studies showing effects increased slightly and the proportion of studies showing no effect decreased slightly (see Supplementary Figure 31).

The remaining quality criteria were applied in too few studies to allow for meaningful comparison i.e., exposure duration not described (*n* = 2), frequency of signal not qualified (*n* = 8). Higher-quality and lower-quality research within each research group (funding affiliation) yielded similar results. However, an opposing pattern emerged when comparing the balance of evidence findings between the vested interest research and the independent research (see Supplementary Table 27).

### All parameters and study quality

The factors above showing effects on study outcomes were collectively examined for any experimental parameters that may explain the lower proportion of DNA damage effects found in higher-quality studies compared to all studies. It was found that higher-quality studies have:

- a slightly greater proportion of *in vitro* studies, which tend to show null results (see Supplementary Tables 24a, b);
- a lower proportion of *in vivo* studies, which typically show statistically significant DNA damage (see Supplementary Tables 24a, b);
- a greater proportion of studies using simulated signals via a signal generator, which further skew findings toward showing no effect (see Supplementary Tables 24c, d);
- used more cell lines, which may contribute to less susceptibility to RF-induced DNA damage (due to uncertainty in cell heritage, phenotype or prior history of radiation exposure) (see Supplementary Tables 24g, h);
- focused less on acute exposures, where DNA damage is often found, and more on short-term exposures, where adaptive responses may exist, reducing the certainty of results (see Supplementary Tables 23a–d);
- significant differences in the number of studies conducted with medium and extremely high intensities, where higher-quality studies focused more on medium-intensity studies, where results for DNA damage are ambiguous, and fewer extremely high-intensity studies, where DNA damage is more likely to be found (see Supplementary Tables 22g, h, m, n);
- a higher proportion of research in this category that is potentially linked to vested interests while also having a stronger emphasis on *in vitro* studies (see Supplementary Figure 53 and Supplementary Table 24); and
- excluded epidemiological studies (due to dosimetry deficiencies), where DNA damage was predominantly found.

These extra parameters listed above may contribute to the lower balance of evidence for effects observed in higher-quality studies, just as much as the established quality criteria (30).

### Dosimetry concerns

Real-world wireless devices are expected to comply with public exposure limits recommended by ICNIRP (15). However, calculating dosimetry in studies utilizing these devices is complex due to the variable nature of the emitted signals (89). Many studies were found to lack adequate information on dosimetry or the methods used for its calculation or measurement.

Some researchers have criticized studies employing real-world devices for “poor dosimetry,” using this argument to question study quality and downplay findings (30, 90). However, minimal differences were observed in the balance of evidence when comparing so called studies with “poor dosimetry ” (which typically represent exposures under real-life conditions) to those with “sufficient dosimetry.” Furthermore, considering these unmodified real-world devices operate within, and often well below ICNIRP's public exposure limits, the critique of poor dosimetry becomes less pertinent when evaluating the validity of current public safety standards.

## Risk of bias and study outcomes

One of the most critical challenges when evaluating the genotoxic potential of RF-EMF is identifying the influence of funding source or author employment relationships, which has the potential to skew research outcomes and hinder the development of evidence-based policies. Funding affiliation(s) have an important influence on reported findings, as demonstrated in past reviews (91–94). A comprehensive analysis of potential biases, which examined authors' focus areas, funding sources, and journals used for publication, revealed a strong influence on reported outcomes as shown by the data patterns below.

### Study parameters vs. funding source

**Figure 13:**
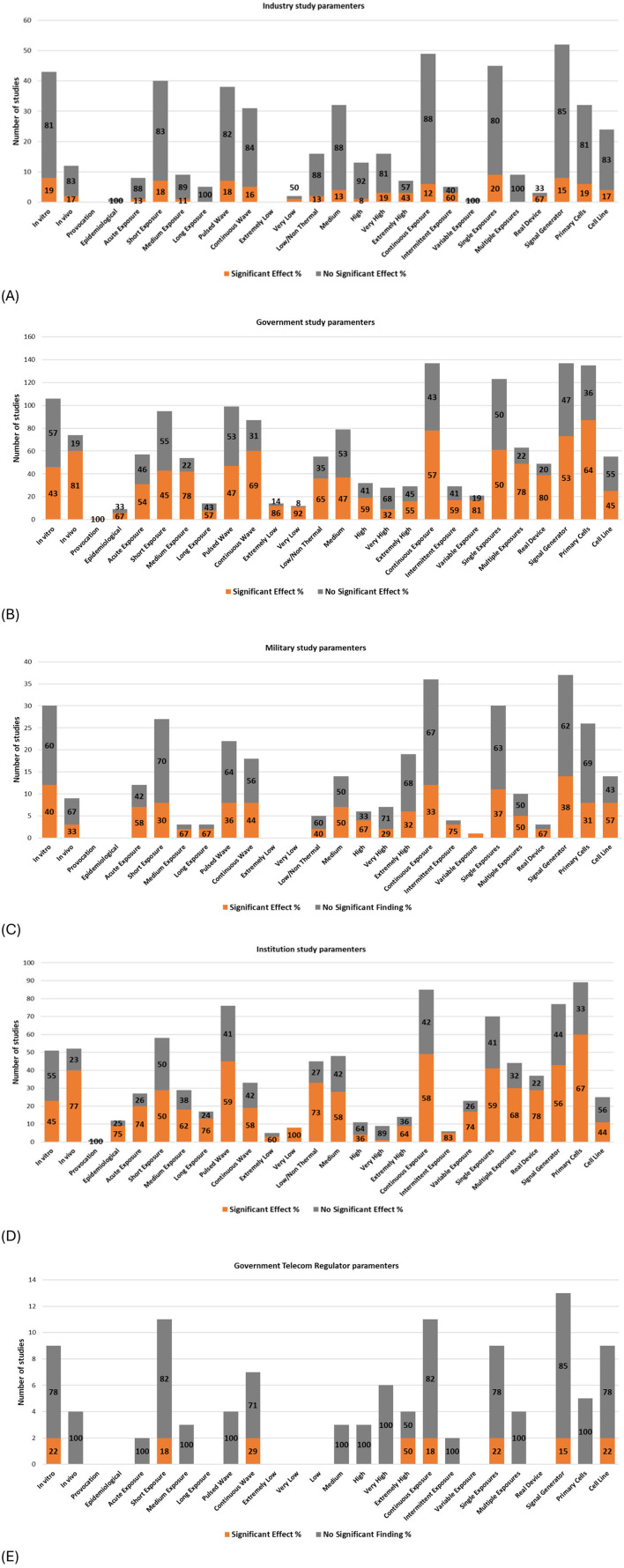
Study Parameters and balance of evidence by primary funding source; **(A)** Industry funded, **(B)** Government funded, **(C)** Military funded, **(D)** Institution funded and **(E)** Telecom regulator funded.

The charts presented in Figure 13 display the balance of evidence by parameter, grouped by primary funding source. (Note that studies may have multiple funding sources including institutional, industry, or government. Therefore, a filter was applied to focus on one funding source for each graph; Note that military and telecom regulator funding was explicitly excluded from government funding).

Research funded by vested interests (e.g., industry, government telecom regulators, and to a lesser extent, military—see section “Classification of potential vested interests”; Supplementary Data Sheet 1) shows a greater proportion of studies concluding “no DNA damage” (as illustrated by the amount of gray color) compared to studies funded by governments, institutions, or private/public sources (not shown). Notably, studies funded by the USAF resemble industry-funded research more than typical military-funded studies (see Supplementary Data Sheet 5, Supplementary Table 25 and Supplementary Figure 35 for full details).

Funding sources appear to have determined various experimental parameters, corresponding to research priorities and potential biases. Studies with potential conflicts of interest (COI), such as those funded by industry, military, or government telecom regulators, organizations who collectively have vested interests in RF technology, exhibit the following research trends:

1. A high number of *in vitro* studies, limited *in vivo* studies, and minimal epidemiological studies;
2. Predominance of short exposure durations (1–24 h), with limited studies on acute, medium, or long-term exposures;
3. Balanced investigation of pulsed and continuous wave exposures;
4. Avoidance of extremely low-intensity exposures, with a preference for medium to high-intensity exposures;
5. Emphasis on continuous exposures, with minimal focus on intermittent or variable exposures;
6. Preference for single exposures over multiple exposures;
7. Greater reliance on signal generators compared to real-world wireless devices; and
8. Balanced use of primary cells and cell lines.

Some of the above study design decisions are more likely to produce null results (as discussed in sections above).

In contrast, independent research i.e., research not funded by industry, telecom regulator or the military, demonstrates broader coverage of study parameters, characterized by:

1. Study designs making use of the entire range of study parameters, with near parity between *in vitro* and *in vivo* study quantity;
2. Most epidemiological studies were conducted as independent research;
3. Short-term exposures are common; however, acute, medium, and long-term exposures are better represented;
4. Slightly greater focus on pulsed wave exposures compared to continuous waves;
5. Heavy use of low/non-thermal and medium-intensity exposures, with good coverage of other intensities;
6. Continuous exposures are more dominant; however, intermittent and variable exposures have also been studied;
7. Single exposures are more common, but less dominant compared to studies funded by vested interests;
8. Signal generators are used more frequently than real-world wireless devices, but the imbalance is less pronounced than studies funded by vested interests; and
9. Primary cells are preferred over cell lines.

Industry, Telecom regulator and USAF-funded studies all have a similar balance of evidence profiles (Supplementary Table 25). In these cases, most studies (>80%) show no significant DNA damage. However, subtle differences are seen between them at the experimental level.

The impact of funding on study outcomes is further compounded by the lack of transparency and disclosure, with 173 studies (~33% of studies) missing a formal funding statement.

### Potential conflicts of interest

The designs and results of research studies that received funding from industry, telecom regulator or military were compared with research studies classified as independent. The two research groups (potential vested interest vs. independent) favored different study types and showed opposing proportions of effects in many cases (refer to Supplementary Table 27). Research funded by, or affiliated with industry or the military, primarily comprised *in vitro* studies, with 30% of 142 *in vitro* and 26% of 31 *in vivo* studies reporting statistically significant findings. Quality filtering did not significantly affect these results (25% of 56 *in vitro* studies and 25% of 12 *in vivo* studies respectively).

In contrast, 63% of the 465 experimental studies were classified as independent research, with a mix of 130 (44.5%) *in vitro* and 162 (55.5%) *in vivo* studies. Independent *in vitro* studies, regardless of quality, showed more null results than *in vivo* studies. Overall, study quality had little impact on independent research, with 74% of all independent studies (*n* = 292) showing statistically significant DNA damage compared to 73% of 62 higher-quality studies.

Higher-quality studies tended to report more conservative results (toward no significant effects), consistent with prior RF-EMF reviews by Wood et al. (95), Karipidis et al. (96), Vijayalaxmi and Prohida (30), and Simko et al. (97). However, these reviews did not assess the combined influence of potential vested interests (e.g., funding or author affiliations) and study quality on outcomes. The significant differences between research group findings (potential vested interests vs. independent) raise serious concerns about the reliability and validity of past review data (30, 95, 96), rendering their effect size comparisons as a potentially unreliable measure, particularly as funding source influence was not considered in their analysis. This finding aligns with previous related research that reported no significant effects of mobile phone use on brain tumors often had industry affiliations either through funding or influence on study design (93, 94). Together, these findings suggest that funding and author affiliation biases study outcomes more than study quality.

Studies funded by industry groups, specific military organizations (particularly the U.S. Air Force), or telecommunications regulators were skewed toward reporting null findings. This is likely due to the experimental methodologies employed by these studies, with a disproportionately large number being conducted as (i) *in vitro* experiments with (ii) short exposure durations (iii) with simple signal modulations from (iv) signal generators, where the likelihood of observing statistically significant DNA damage is much lower than when using real-world devices emitting signals carrying voice or data over longer exposures. These design decisions appear to bias results toward null outcomes. Supplementary Table 27 shows how studies funded by organizations with vested interests are biased toward *in vitro* experiments, resulting in a lower proportion of studies finding effects.

On the other hand, independent funded research has conducted a much broader range of experiments and primarily reported statistically significant DNA damage. These findings suggest that, compared to all studies overall, the more conservative results of higher-quality studies are likely due to the high proportion of higher-quality studies being performed by researchers who have received funding from vested interests, the greater prevalence of *in vitro* studies with short exposures from signal generators, and the exclusion of epidemiological studies due to dosimetry limitations.

The bibliographic network charts in Supplementary Figures 54–56 illustrate the connections between researchers and research groups in this area.

### Publication bias

Papers concluding that no DNA damage effects have been observed are primarily published in three journals: Radiation Research, Bioelectromagnetics, and the International Journal of Molecular Sciences. Papers concluding that DNA damage effects have been found appear in some of the same journals but are less concentrated. Overall, independent research has been published across a wide array of journals. In contrast, research associated with industry or the military through funding or employment, has to a larger degree been confined to a small group of journals (see Supplementary Table 29); e.g., *Radiation Research* (98). The clustering of studies funded by vested interests in select journals can distort the perceived balance of evidence, potentially misleading policymakers, radiation safety practitioners, and the public.

### Overall research bias

All the above demonstrate how methodological preferences and potential conflicts of interest inherently bias the results of studies (and reviews), reducing the robustness of the overall evidence base and thereby creating uncertainty.

This pattern of bias has been documented in other lucrative industries, such as pharmaceuticals (99), agriculture, and chemical manufacturing (100). Such biases must be acknowledged and accounted for to ensure the integrity and reliability of published findings, particularly when conducting future systematic reviews.

## Research limitations and gaps

### Limitations of this evidence map

#### Vote counting limitations

The choice to use vote counting for the syntheses of data introduces limitations which incur caution when interpreting the results of the mapping process.

Vote-counting of effect direction across primary studies is known to be underpowered, giving no indication of effects sizes, or range of effect sizes in primary studies (101, 102) and does not weigh studies by their precision or sample size. All studies are treated equally irrespective of in study size, design rigor, and statistical power (103). Consequently, the findings reported from a vote counting synthesis are based on simple counts of studies reporting proportions of effect vs. no effect outcomes in all dimensions and categories of interest, regardless of statistical power or study quality. The results should therefore be interpreted as informative descriptors of reported outcomes rather than proof of causation.

Because vote counting does not usually include a quality assessment of studies, the reliability of the evidence making up the outcome proportions is unknown, leading to uncertainly regarding the implication of findings. However, in the current evidence mapping process, studies were categorized according to the presence or not of established quality criteria, as extracted from the study text. In this way, vote counts could then be conducted for the higher quality studies and compared with vote counts for the lesser quality studies. This process enabled more confidence to be given to the final proportions allocated to each category that was investigated within the ‘higher quality' partition.

Overall, the results of the vote-count analysis used in the current evidence synthesis should be viewed as an exploratory mapping exercise that identifies broad patterns along an array of dimensions. The results cannot provide cause-and-effect explanations regarding any particular dimension or category, but rather, they give an indication of which dimensions and categories are most influential in showing effects of RF-EMF on DNA damage. Furthermore, the results indicate where the research gaps are located to inform future research.

#### Narrower subsets more suitable for meta-analysis

It could be argued that more precise quantitative synthesis might be feasible for narrowly defined subsets of the reviewed literature (for example: a meta-analysis of studies using the same cell line, exposure frequency, and comet assay). However, such homogenous subsets would be very small and only able to provide evidence for very narrow issues, which would be in contradiction to the aims of the scoping review, i.e., to map the breadth of evidence and to identify gaps.

#### Multiple different dose-response relationships

A U-shaped time dose-response relationship was postulated from pooling of all other dimensions across the designated time sub-categories (< 1 min–>1 yr). However, some studies investigating exposures across multiple time categories did not reproduce this response; i.e., some showed a linear dose-response relationship (104, 105), while others showed an inverse relationship (106) or no effects (107, 108). This inconsistency may stem from the limited number of studies employing multiple assays covering more than four-time bands, so looking at individual studies to validate the observed U-shaped pattern becomes problematic. To confirm the U-shaped duration dose-response relationship and better understand its implications, future research should incorporate multiple assays across a broader range of exposure intensities and time bands, using standardized protocols to improve comparability and reproducibility of findings.

### Limitations of past research

Many issues were identified in both experimental and epidemiological studies, separate from those required for “quality” assessment. Some of the more important issues are summarized below.

The purity of the signal-free sham or control environments could not be confirmed for most studies, because background measurements were either not taken or not reported. Verification of the test environment's EMF integrity and identification of potential stray fields acting as confounding factors was therefore not possible.

Many studies were found to be missing important methodological details. The absence of transparency in methods makes it difficult to evaluate study quality or to replicate studies. Such studies were classified as lower quality studies but were still included in the evaluation of DNA damage outcomes.

Some studies only presented pooled data, which can hide potentially “sensitive responders”; e.g., the blood of some individuals showed more damage from exposure from others (40–42), similar response have been seen in millimeter wavelength research with animals (109, 110).

Several epidemiological studies investigating buccal mucosa micronuclei from long-term mobile phone usage found no statistically significant difference between the left and right cheek. The authors did not consider nonlinear responses or the penetration capability of the RF-EMF from mobile phones. A more relevant comparison is between heavy and light mobile phone usage.

More specific concerns identified in studies are noted in the Systematic Evidence Database (see Supplementary Data Sheet 4, “Final Study List (Experimental)”: column CH and “Final Study List (Epidemiological)”: column CF).

### Heterogeneity of studies

An analysis of study parameters reveals significant variability across studies, which differ in exposure time, intensity, frequencies, modulations, assay methods, DNA assessment timings, equipment, organism and cell types, among others. Assays also use varying stains, preparation techniques, and timings, which can impact sensitivity. Even studies claiming to replicate others often have subtle differences, such as variations in animal species and comet assay parameters as described below.

#### Comet assays

An analysis of studies using the comet assay to measure DNA damage further exemplifies the heterogeneity across studies in assay parameters and the recording of results. Variations in these protocols, such as buffer formulations, temperature, electrophoresis voltages, runtimes, etc., can affect the sensitivity of the assay by influencing protein removal, DNA migration and the ability to detect damage (34). Additionally, reported outcomes use diverse metrics (e.g., tail moment, tail intensity, %tail DNA, damage index, etc.) which complicate data synthesis.

Overall, this pervasive heterogeneity, along with few studies in each category makes a quantitative meta-analysis for a systematic review unfeasible at present. Future research requires the use of agreed-upon standardized and established experimental protocols, so as to improve repeatability and comparability across studies, and to allow for effective evidence synthesis. This scoping review, which processed over 530 studies, highlights the critical need for such standardization.

### Research gaps

The evidence map and synthesis presented here have identified several important research gaps. Firstly, there were limited studies investigating the genotoxic effects of RF on non-mammalian species, including insects, birds, and trees. There is also a complete absence of studies on reptiles, and within mammals, there is scant research beyond human and rodent studies.

Most human experimental studies utilized conditions that did not reflect real-life exposure scenarios, typically involving *in vitro* experiments with short, single, continuous exposures to a simulated signal involving one specific frequency. In contrast, real-world exposures are characterized by chronic, simultaneous exposures to multiple signals overlayed with a variety of modulation patterns and intensities. Such conditions were not used in typical laboratory studies.

There were also few studies using higher frequencies, such as 5G technologies, despite their growing prevalence. The research is therefore lagging behind technological advancements, leaving potential risks largely unexplored.

Inconsistencies observed in past research stem from the complex interplay of numerous variables. For example, both therapeutic and potentially harmful effects have been identified, with this duality possibly tied to the combined effects of intensity and duration of exposures. To clarify these complex relationships, future studies should methodically address gaps in key experimental dimensions, such as exploring long durations and extremely low to very low exposure intensities (see Figure 9B). Additionally, few studies have investigated the combined effects of RF-EMF with other environmental stressors, which could amplify genotoxic outcomes in diverse settings and need further investigation (111).

## Implications for policy and practice

The evidence from the evidence map indicates that medium to long-term RF-EMF exposures, particularly at low intensities, can induce genetic damage through non-thermal mechanisms such as increased free radical production and oxidative stress. Genetic damage can have far-reaching, long-term, and potentially irreversible consequences for individual organisms and broader ecological and planetary health (112, 113).

Both *In vivo* and epidemiological RF-EMF studies provide credible evidence of genotoxicity, suggesting potential risks such as increased cancer susceptibility and reproductive harm. Studies on brain cells frequently reported positive findings for DNA damage, suggesting that brain cells may be particularly sensitive to RF-EMF, indicating a risk for neurological diseases and brain tumors, as observed in animal models (114–116).

Current RF-EMF exposure guidelines established by ICNIRP (15), prioritize the prevention of thermal effects by incorporating substantial safety margins (e.g., a 50-fold reduction from effect thresholds, setting a local SAR limit of 2 W/kg for the head and torso for the general public, averaged over 10 grams of tissue). However, the evidence mapping process found statistically significant DNA damage at extremely low intensities, with the lowest recorded effects occurring at a SAR of 0.000000319 W/kg in an epidemiological study (117) and at 0.000003 W/kg in several *in vivo* experiments (118, 119). These levels are substantially (>600,000 times) below the ICNIRP public exposure limits (15). This pattern suggests non-thermal genotoxic effects, because temperature changes at these intensities would be negligible and not measurable.

ICNIRP (2020) guidelines (15) set RF-EMF exposure limits to protect against thermal effects from acute exposures, with averaging times of 6 min for local exposure (head and torso) and 30 min for whole-body exposure. However, the above analysis revealed that medium (1 day−3 months) and long-term RF-EMF exposures (>3 months or 1,000 h) were most strongly linked to genotoxic effects, even at very low exposure intensities. ICNIRP (2020) guidelines (15) do not set specific limits for chronic, low-level RF-EMF exposures, particularly for non-thermal effects like genotoxicity, citing “*no substantiated evidence of health-relevant effects*” [(15), p. 522].

The mapping process also revealed that RF exposures are associated with genetic damage in a wide range of organisms, with an observed sensitivity of non-mammalian organisms, such as plants, insects, and possibly amphibians. Current guidelines neglect potential effects on wildlife or ecosystems (78, 96). Notably, a recent WHO-commissioned systematic review of animal studies suggested carcinogenic effects from RF-EMF exposures (116). Other studies suggest biological effects on non-human species (120, 121). Together, these results suggest that the environmental implications of RF-EMF exposure merit greater scrutiny (122), even though the current evidence remains limited and debated (96).

While these findings do not yet establish causation or a clear No Observed Adverse Effect Level (NOAEL), they indicate risks that ICNIRP's current framework discounts by prioritizing only effects with confirmed harm [(15), p. 487]. ICNIRP's review process and position is best described as a hazard-based assessment focused only on confirmed effects. This approach is overly restrictive, as it delays updating guidelines until absolute certainty is achieved (123), which may not align with the precautionary needs of public health or environmental protection.

Currently, there is a widespread (6) and often non-consensual nature to RF-EMF exposure (92) from mobile phones, base stations, and other wireless technologies. While acknowledging the permanence of this technology in modern society, policy adjustments are required that prioritize health and environmental protection over economic interests. This can be achieved by adopting a precautionary approach to RF-EMF (123) and addressing potential risks from non-thermal RF-EMF effects, despite scientific uncertainty. Strategies such as justification (assessing net benefits of RF-EMF applications), optimization (balancing protection with societal needs), and As low as Reasonably Achievable or As Low as Technically Achievable - ALARA/ALATA (avoiding deterministic effects and minimizing stochastic effects) per International Commission on Radiological Protection (ICRP) recommendations - ICRP103 (124) could be considered. Further development and deployment of wireless technologies should incorporate improved safety measures in their design (125), such as creating devices that emit lower levels of RF-EMF or using materials and antenna designs to direct emissions away from the body.

Additionally, public information regarding potential health risks and personal protective measures could be disseminated through public health campaigns, making use of existing advice such as the EUROPAEM EMF Guideline 2016 (126); e.g. minimizing the use of wireless devices, prioritizing wired connections, maintaining distance between RF-EMF sources and the body, use of air-tube headsets or handsfree calls, turning off wireless when not in use, and mitigation of oxidative stress by incorporating antioxidants into the diet.

While individual actions are valuable, they are not a substitute for robust regulatory standards and industry accountability. Ensuring the safety of wireless technologies requires a collective effort from manufacturers, policymakers, and consumers to develop comprehensive RF safety guidelines. Future regulatory guidelines could encompass workplace protection measures, including substitution, engineering, and administrative controls (127), integration of building biology standards (128), mandatory detailed product labeling to inform users of potential risks, and standardized safety hygiene practices.

## Recommended actions

To address these concerns and bridge existing gaps, the following actions are recommended:

1. **Standardization of Research Protocols**: Harmonizing methodologies across studies, particularly comet assay protocols, is critical for reducing heterogeneity and enabling robust meta-analyses.
2. **Focus on Long-Term and Low-Intensity Exposures**: Future research should prioritize investigating the cumulative effects of prolonged and low-intensity RF-EMF exposures, which are most relevant to real-world scenarios and devices.
3. **Inclusion of Emerging Frequencies**: Given the rapid deployment of 5G and other novel technologies, research focused on higher frequencies and new modulation schemes is urgently needed.
4. **Targeted Environmental and Health Studies**: Targeted research in both human health and ecological systems needs to be conducted independently of vested interest influences, ensuring methodological rigor in each domain.
5. **Independent Funding and Research Oversight**: To mitigate biases associated with industry funding, greater support for independent research is essential. Transparent disclosure of ALL funding sources and researcher affiliations should be mandatory.
6. **Re-evaluation of RF Standards**: Regulatory bodies must update exposure guidelines to reflect non-thermal mechanisms and the potential health effects from long-term chronic exposure settings by incorporating findings from independent, high-quality studies.

## Conclusions

The evidence map presented here reveals statistically significant DNA damage in humans and animals resulting from man-made RF-EMF exposures, particularly DNA base damage and DNA strand breaks. The evidence also suggests plausible mechanistic pathways for DNA damage, most notably through increased free radical production and oxidative stress. Sensitivity to damage varied by cell type, with reproductive cells (testicular, sperm and ovarian) along with brain cells appearing particularly vulnerable. A complex U-shaped dose-response relationship was observed for both exposure duration and intensity, with more DNA damage occurring in specific frequency and intensity combination windows. DNA damage was more likely to be found using *in vivo* studies, very weak or very strong signal intensities, very short or very long exposure durations, 900, 1,800 and 2,450 MHz frequencies, GSM-talk mode and pulsed modulations, particularly when using real-world devices. On the other hand, research funded by vested interests has tended to use different experimental design parameters, with a high proportion of studies using *in vitro*, short-term exposures, medium-high intensity signals and using signal generators. Funding source is also a stronger determinant of experimental outcomes than study quality.

Overall, there is a strong evidence base showing DNA damage and potential biological mechanisms operating at intensity levels much lower than the ICNIRP recommended exposure limits. Public policy could benefit from the implementation of precautionary measures such as ALARA or ALATA, along with public information campaigns to better safeguard human and environmental health and wellbeing.

## Data Availability

The original contributions presented in the study are included in the article/Supplementary material, further inquiries can be directed to the corresponding author.

## Footnotes

^1^All references to Supplementary Table or Supplementary Figure refer to details in Supplementary Results Map (Supplementary Data Sheet 5).
