# Supplementary material for: A scoping review and evidence map of radiofrequency field exposure and genotoxicity: assessing in vivo, in vitro, and epidemiological data
Source: Front Public Health. 2025 Jul 30;13:1613353. doi: 10.3389/fpubh.2025.1613353 (PMC12343714; doi:10.3389/fpubh.2025.1613353)
Supplement: Supplementary file 1 [file Data_Sheet_1.pdf]

Methods

The protocol used to generate this evidence map and conduct data synthesis follows the Preferred Reporting Items for Systematic Reviews and Meta-Analyses extension for Scoping Reviews (PRISMA-ScR)(1) and the Joanna Briggs Institute (JBI) Scoping Review guideline(2).

Searching for articles

Publication databases used for search

The following list of general research databases was used to conduct literature searches:

- National Institutes of Health (NIH) MEDLINE database using the PubMed search tool;
- SCOPUS multidisciplinary database;
- Web of Science bibliographic database;
- Elsevier's ScienceDirect platform for e-books and peer-reviewed journals.

In addition to the above general research databases, several specialised research databases that are specifically focused on non-ionising EMF research were also utilised and include:

- IEEE Xplore database;
- EMF-Portal database, which is maintained by the Research Centre for Bioelectromagnetic Interaction at RWTH Aachen University in Germany;
- Oceania Radiofrequency Scientific Advisory Association (ORSAA) Database on Electromagnetic Bioeffects (ODEB).

Search terms and strings

RF exposure studies use various terminologies and acronyms that encapsulate radiofrequency emissions and the technology that utilises them. Additionally, DNA damage can exist in many different forms. Searches with various parameters that incorporate the technology (2G, 3G, 4G, 5G, Wi-Fi, etc.) and devices (mobile phone, base station, microwave oven etc.) were initially tried and compared against more generic searches that focused on terms related to the physical definition of the electromagnetic fields under review (radiofrequency, microwave, millimeter waves, mmwave) etc.

Ultimately, the specificity of the search terms was kept relatively simple, avoiding the usage of explicit technology terminology or overly broad terms such as electromagnetic fields or Hz. This was done purposefully to prevent the returned pool of studies from being contaminated with large numbers of irrelevant papers focusing on other frequencies and types of radiation not in scope. A balanced approach between the research to be selected and the amount of effort to screen them was an important consideration. The use of search terms related to specific wireless technology or generations of technology was also intentionally avoided because specialised and focused databases, such as EMF-Portal and ODEB, already encompass wireless technology, thereby making such a requirement redundant.

Some research databases (Science Direct and EMF-Portal) restrict the number of keywords that can be used in a search operation and so customised search strings had to be developed. For EMF-Portal, it was necessary to create separate search criteria for each type of DNA damage independently. The output of each search result was combined and then sorted to identify and remove any duplicates. Search strings for each search operation are provided in Supplementary file-3 and are dated for transparency.

Search for grey literature

A supplementary search was performed through a bibliographic review of randomly selected studies from the established research collection identified in the initial database searches. Additional internet resources, including Google Scholar and ResearchGate, were searched manually. ResearchGate was further utilised to contact registered authors directly to request access to research papers behind paywalls or unavailable.

Search limitations

Searches were conducted exclusively using English keywords. Only studies published in English were included in this evidence map due to resource limitations for supporting other language translations. This means that relevant research sets of an indeterminate size from China, Russia, Eastern Europe and other non-English countries were not included. Articles were accepted without any restrictions on the publication timeframe.

Search results

Supplementary file-4 is a Microsoft Excel workbook “Systematic Evidence Database” containing all search results combined under the tab “Initial List”.

Assessing the comprehensiveness of the search

The combined search results were tested against the latest narrative review published by Lai in 2021(3), which provides a large collection of studies investigating genetic effects resulting from RF exposures. All the papers identified by Lai and many more were found by the search.

Article screening and study eligibility criteria

Screening process

Once the search for papers was completed, article screening was conducted in three phases. The first phase involved sorting the articles by title and identifying and tagging records as duplicates. The second phase involved screening the Title and Abstract of each paper for relevance. The third phase involved full-text analysis, required when the abstract or title provided insufficient information to make a full determination. Papers identified as not being relevant were tagged.

Primary reasons for exclusion

The reasons for exclusion were twofold. The primary reasons are listed below. The secondary reasons, which are numerous, are listed in Supplementary file-4 under the “Review” tab.

1. Ablation study
2. Abstract only - Secondary reason can be either a meeting abstract or a conference abstract (if they do not contain sufficient methodological and statistical information)
3. A comment or letter to the editor on others’ work
4. Cooking study
5. DNA damage not assessed
6. Duplicate
7. Incomplete Information
8. In Situ Hybridisation technique [using microwaves]
9. Is a Monograph [not an experimental or observational study]
10. Not an English paper
11. Not an RF exposure study
12. No RF only condition
13. Retracted article
14. Review or opinion piece

Phase three also included the tagging of epidemiological studies to identify them separately from experimental studies. All reasonable attempts were made to retrieve full papers for each article for this final phase of screening, including use of Griffith University Library resources, ResearchGate, the Internet and author contact.

SW and VL performed a full assessment of the combined search result for validity and inclusion. Decision-making consistency for eligibility (see below) was tested for all papers in the combined search results that were analysed by SW and VL. The decision-making test found 93% agreement and a kappa(κ) of 0.848. Where there were discrepancies, these were discussed and resolved within the review team comprising SW, VL and JM. Studies that passed through all three stages of review were deemed eligible for inclusion in the evidence map.

Eligibility criteria

Article eligibility was based on the following criteria:

- Populations or subjects: All organisms, cell types and free DNA;
- Exposure(s): RF-EMF in the frequency range of 3 kHz–300 GHz, either applied directly in experiments or from existing anthropogenic environmental sources (observational studies). All exposure intensities were included in the evidence map and quantitative synthesis. However, the quality synthesis only included studies with exposure intensities equal to or less than ICNIRP occupational limits;
- Comparator(s): Sham, or Control in the absence of a sham, lesser exposure in the case of epidemiological studies;
- Outcomes: DNA damage as the primary outcome, with secondary outcomes as DNA conformational change, spindle disturbances, free radical production/oxidative stress, heat shock protein expression and apoptosis (including caspase 3 or BCL/Bax expression);
- Study designs: Only experimental studies performed in a laboratory (*in vitro*/*in vivo* studies) and observational (epidemiological) studies.

Data coding strategy

The large range of methodological and outcome parameters for studies were coded and recorded into comprehensive data extraction sheets labelled “Final Study List (Experimental)”, and “Final Study List (Epidemiological)”. At least 85 key attributes were captured and coded from experimental and observational studies. A subset of studies meeting quality criteria is also included in the same excel workbook in a separate tab. The metadata coding definitions, which are self-explanatory, are elaborated in a “Data Coding Description” tab in Supplementary file-4. Secondary data relating to comet assay (23 parameters) were also captured in a separate tab labelled “Comet Assay”.

The analysis of the selected papers was conducted at the level of study (*in vitro*, *in vivo* or epidemiological) rather than individual papers, because five papers contained more than one study. Studies were evaluated to determine whether they reported at least one significant effect across a range of outcomes, including different types of DNA damage and other biological effects. Experimental parameters, including exposure intensities and durations, were recorded and colour-coded for clarity in the dataset.

No contact was made with authors if the study methodological data was found to be missing details in full papers to provide full transparency on reporting thoroughness. This was purposefully done to highlight how experimental details are not always fully disclosed when detailing study methodology, and because this study is not a formal systematic review with a meta-analysis component.

Assumptions

Several assumptions were made when processing or coding each research publication. It was assumed that the data contained within each paper was accurate and not fabricated. Published corrections were also taken into consideration, if available. It was also assumed that experimental results were not withheld, and that all relevant experimental data was published including null results and positive findings. Finally, funding sources were assumed to be accurate and fully disclosed when a declaration was made.

Quality assessment and Risk of Bias analysis

A separate quality assessment was performed on a subset of studies meeting quality criteria. The assessment adopted and extended the recommended quality attributes used by Vijayalaxmi and Prihoda(4). The specific quality attributes used for the selection of 'higher quality' studies were as follows:

- Waveform specified (pulsed wave or continuous wave) and modulation described (WQ=1);
- Exposure duration fully described (TQ=1);
- Exposure intensity details either as W/kg, V/m, or unit that can convert to W/m^2 (EQ=1);
- Frequency of signal well qualified (FQ=1);
- Dosimetry was sufficiently calculated and/or measured [Dosimetry Rating=Sufficient];
- Blinding/coding used [Blinded=Y or "Coded"];
- Sham control used [Sham=Y];
- Statistical methods described [Statistics≠"not specified"] and [Significance≠"NS"].

A positive control was not judged to be critically important for quality determination, which is a deviation from Vijayalaxmi and Prihoda quality review protocol. While positive controls are desirable for evaluating an assay's sensitivity, comparing effects against a known genotoxic agent, they are not essential for determining cause and effect using experimental logic(5). All experiments meeting the above quality criteria have sham and exposed assay data for comparison as a minimum, which is sufficient.

This evidence map did not include a formal risk of bias (ROB) assessment of each study due to the large volume of selected papers, the significant time/effort that would be required and because this is not necessary for producing an evidence map. However, a surrogate approach was applied, which involved reviewing the balance of evidence through various lenses and qualifying significant differences. The following study parameters were investigated:

- Blinding vs no blinding
- Sham exposure vs no sham (i.e., control only)
- Sufficient dosimetry vs poor dosimetry
- Potential vested interests vs no vested interests

Classification of potential vested interests

The identification of potential vested interests is a sensitive subject. The classification of studies was conducted objectively and neutrally using clear, verifiable evidence to minimise the influence of personal opinions or preconceived notions. The following rules were used, without exception, to designate a paper as potentially linked to vested interests:

1. If a paper received telecommunications/power industry funding, irrespective of whether firewalls were in place. Reasons: Wireless communication is a multi-trillion dollar industry

and with regards to firewalls, researchers are fully aware of where funding comes from, and future funding may depend on published results;

2. If a paper received military funding. Reason: radiofrequencies are a critical component of national defence and communications;
3. If a paper received Government telecommunication regulator funding. Reason: Telecom regulators make significant revenue from the sale of spectrum licenses;
4. If a paper had industry, ICNIRP or military co-authors. Reason: industry and military researchers have conflicts of interest, while many ICNIRP commissioners have been found to have industry connections(6, 7);
5. If a funding declaration was not provided (not to be confused with 'no funding received'), the author/co-author's funding history was reviewed as per steps 1-4 above and applied.

If none of the above elements were satisfied, then a paper was designated as "no-vested interest". In all instances, study outcomes were not used as a determining factor.

The balance of evidence for each main funding source was then mapped to identify relationships between funding source, experiment type and parameter focus versus outcomes.

Data synthesis

Vote counting is a simple quantitative aggregation method for synthesising evidence from multiple data viewpoints for evaluation, by comparing the number of positive studies with no effect studies.

This approach was taken due to the following reasons:

1) Experimental data (methodology, signal characteristics, organism/cell under study and reporting) was found to be extremely heterogeneous precluding more rigorous statistical methods (i.e. Meta-Analysis techniques used in Systematic Reviews incorporating quantitative analysis) where a degree of homogeneity is a requirement
2) Size of the database, time required and the small research team
3) This is an exploratory scoping study not a formal systematic review

This chosen method is consistent with the exploratory goal of a scoping review – which is to systematically map evidence and identify overall patterns across a broad topic(1). In vote counting, each study's outcome is classified (e.g., evidence of DNA damage vs no effect) and the number of studies in each category is tallied(8).

In other words, each study contributed one "vote" along each particular dimension under investigation, as a coarse indication of the distribution of evidence from all studies along a dimension or within a category of interest. It allows the examination of how outcomes are distributed by study feature.

All steps of the vote-count analysis are defined and fully reported below, in line with PRISMA-ScR and JBI scoping guidance to emphasise descriptive summarisation of study characteristics.

Conventional meta-analysis requires a degree of homogeneity in outcomes and study design, which was lacking in the comprehensive Systematic Evidence Database, making techniques like meta-regression or publication-bias plotting potentially unreliable. Standard meta-analytic methods are designed for linear relationships or consistent effect measures. While advanced methods exist to model non-linear effects, they were not feasible here due to data limitations, e.g., insufficient studies with homogeneity in methods or incompatible

outcome measures. Consequently, a simple vote-counting method was employed, despite its limitations, to summarise the findings.

On the other hand, a purely narrative summary of 530 studies would also likely obscure overall trends. Without a quantitative tally, it would have been easy to introduce bias by overemphasising certain results. The vote-counting strategy was therefore chosen so as to find a middle ground between these two problematic approaches, by providing a high-level, detailed map of the evidence while avoiding the pitfalls of selective narrative description.

Vote counting method

At the summary level, each study was given one vote ("Effect" or "No Effect") for DNA Damage and one vote for each of the biological endpoints (specific DNA damage type or potential mechanism) that were investigated separately.

DNA Damage summary voting data was set to "Effect" if any one of the studies' specific DNA damage endpoints (i.e. DNA base damage, micronuclei etc.) had a statistically significant finding for **increased** DNA damage, otherwise it was set to No Effect. Protective effects, even if significant were classified as "No Effect".

Secondary variables such as study type (in vitro, in vivo or epidemiological), organism or cell type, real or simulated signal, methods (blinding, sham, control, dosimetry rating) signal characteristics (frequency, pulsed, continuous or modulation patterns), exposure pattern (continuous, multiple, single, intermittent or variable), cell type (primary cell or cell line) and funding source (Industry, Military, Telecom Regulator, Government, Institution, Public Private, Vested Interest and Independent Researchers) were evaluated and mapped using a single vote per study.

When analysis included a time domain (coarse grained exposure duration categories - Acute, Short, Medium, Long or specific time bands i.e., A to S) or exposure intensity (Extremely Low – EL, Very Low – VL, Low/Non Thermal – L, Medium – M, High – H, Very High – VH or Extremely High – EH), a study may have been given multiple votes, depending on how many time domains or exposure intensities were investigated in that study (e.g., a study may have investigated 900 and 1800MHz specific intensities, for each of which the study would be given one vote).

Vote counting was performed to test specific methodological choices for bias analysis. Summary DNA Damage votes were compared for Control vs Sham, Blinding vs No Blinding, Poor Dosimetry vs Sufficient Dosimetry. These different bias tests were also compared with higher quality studies that used Blinding + Sham + Sufficient Dosimetry

Vote counting was used to compere lower quality studies with higher quality studies. This was conducted by comparing summary voting data for "DNA Damage" for lower and higher quality studies. Quality grading was determined by performing the "Quality assessment and Risk of Bias analysis" steps described above on each individual study.

Definitions for exposure time and exposure intensity labels can be found in supplementary document 4 and in the main article.

References

1. Tricco AC, Lillie E, Zarin W, O'Brien KK, Colquhoun H, Levac D, et al. PRISMA Extension for Scoping Reviews (PRISMA-ScR): Checklist and Explanation. Ann Intern Med 2018;169:467-73.
2. Peters MDJ, Godfrey C, McInerney P, Munn Z, Tricco AC, Khalil H. Scoping Reviews (2020). In: Aromataris E, et al., editors. JBI Manual for Evidence Synthesis: JBI; 2024
3. Lai H. Genetic effects of non-ionizing electromagnetic fields. Electromagn Biol Med 2021:1-10.
4. Vijayalaxmi, Prihoda TJ. Comprehensive Review of Quality of Publications and Meta-analysis of Genetic Damage in Mammalian Cells Exposed to Non-Ionizing Radiofrequency Fields. Radiat Res 2019;191:20-30.
5. Moller P, Azqueta A, Boutet-Robinet E, Koppen G, Bonassi S, Milic M, et al. Minimum Information for Reporting on the Comet Assay (MIRCA): recommendations for describing comet assay procedures and results. Nat Protoc 2020;15:3817-26.
6. McCredden JE, Weller S, Leach V. The assumption of safety is being used to justify the rollout of 5G technologies. Front Public Health 2023;11:1058454.
7. Weller S, McCredden JE. Understanding the public voices and researchers speaking into the 5G narrative. Front Public Health 2023;11:1339513.
8. Aromataris E, Lockwood C, Porritt K, Pilla B, Jordan Z. JBI Manual for Evidence Synthesis. Adelaide: JBI; 2024.
