# Supplementary material for: A scoping review and evidence map of radiofrequency field exposure and genotoxicity: assessing in vivo, in vitro, and epidemiological data
Source: Front Public Health. 2025 Jul 30;13:1613353. doi: 10.3389/fpubh.2025.1613353 (PMC12343714; doi:10.3389/fpubh.2025.1613353)
Supplement: Supplementary file 2 [file Data_Sheet_2.pdf]

## Preferred Reporting Items for Systematic reviews and Meta-Analyses extension for Scoping Reviews (PRISMA-ScR) Checklist

| SECTION | ITEM | PRISMA-ScR CHECKLIST ITEM | REPORTED ON PAGE # |
|---|---|---|---|
| **TITLE** | | | |
| Title | 1 | Identify the report as a scoping review. | A scoping review and evidence mapping of radiofrequency field exposure and genotoxicity: assessing in vivo, in vitro, and epidemiological data |
| **ABSTRACT** | | | |
| Structured summary | 2 | Provide a structured summary that includes (as applicable): background, objectives, eligibility criteria, sources of evidence, charting methods, results, and conclusions that relate to the review questions and objectives. | The abstract meets the stated requirements |
| **INTRODUCTION** | | | |
| Rationale | 3 | Describe the rationale for the review in the context of what is already known. Explain why the review questions/objectives lend themselves to a scoping review approach. | Rationale is provided in the manuscript under the subheading **Background and rationale.** |
| Objectives | 4 | Provide an explicit statement of the questions and objectives being addressed with reference to their key elements (e.g., population or participants, concepts, and context) or other relevant key elements used to conceptualise the review questions and/or objectives. | A PECO statement and questions are provided in the Manuscript. PECO is detailed under the sub-heading **PECO statement**. Questions are detailed under subheadings **Primary question items** and **Secondary question items.** |
| **METHODS** | | | |
| Protocol and registration | 5 | Indicate whether a review protocol exists; state if and where it can be accessed (e.g., a Web address); and if available, provide registration information, including the registration number. | The protocol has not been registered previously. However, the full protocol is included with the manuscript as a supplementary document for full transparency. |
| Eligibility criteria | 6 | Specify characteristics of the sources of evidence used as eligibility criteria (e.g., years considered, language, and publication status), and provide a rationale. | Page 2 and 3 of the supplementary document – Scoping Review Map Method and Protocol provide this detail along with Supplementary file 4 - Systematic Evidence Database.xslx |
| Information sources* | 7 | Describe all information sources in the search (e.g., databases with dates of coverage and contact with authors to identify additional sources), as well as the date the most recent search was executed. | Page 1 of supplementary document – Scoping Review Map Method and Protocol.pdf, as well as the section titled **Results from search and screening** in the manuscript. |

| SECTION | ITEM | PRISMA-ScR CHECKLIST ITEM | REPORTED ON PAGE # |
|---|---|---|---|
| Search | 8 | Present the full electronic search strategy for at least 1 database, including any limits used, such that it could be repeated. | Page 1 of supplementary document – Scoping Review Map Method and Protocol. All database search strings are included in supplementary file 3 - Search Data.zip |
| Selection of sources of evidence† | 9 | State the process for selecting sources of evidence (i.e., screening and eligibility) included in the scoping review. | Page 2 of supplementary document – Scoping Review Map Method and Protocol.pdf. Also, refer to PRISMA diagram in the manuscript. Supplementary file 4 - Systematic Evidence Database.xslx contains the detailed list of exclusion criteria. |
| Data charting process‡ | 10 | Describe the methods of charting data from the included sources of evidence (e.g., calibrated forms or forms that have been tested by the team before their use, and whether data charting was done independently or in duplicate) and any processes for obtaining and confirming data from investigators. | Page 4 of the supplementary document – Scoping Review Map Method.pdf and the section titled **Results from search and screening** in the manuscript. |
| Data items | 11 | List and define all variables for which data were sought and any assumptions and simplifications made. | Page 3 of the Supplementary file - 3 Scoping Review Map Method and protocol.pdf and Supplementary file 4 - Systematic Evidence Database.xslx. |
| Critical appraisal of individual sources of evidence§ | 12 | If done, provide a rationale for conducting a critical appraisal of included sources of evidence; describe the methods used and how this information was used in any data synthesis (if appropriate). | Quality assessment and Risk of bias assessment as described on page 4 of Supplementary file 3 - Scoping Review Map Method and protocol.pdf. |
| Synthesis of results | 13 | Describe the methods of handling and summarising the data that were charted. | This is described in 2 sections of Supplementary file 3 - Scoping Review Map Method and protocol.pdf<br>Firstly, **Data coding strategy** on p3 and secondly **Data synthesis** on p5. |
| **RESULTS** | | | |
| Selection of sources of evidence | 14 | Give numbers of sources of evidence screened, assessed for eligibility, and included in the review, with reasons for exclusions at each stage, ideally using a flow diagram. | PRISMA diagram is provided in the manuscript with reasons for exclusion. Supplementary file 4 – Systematic Evidence Database.xslx provides more detailed information for exclusions. File 5: Systematic Evidence Map and Manuscript |
| Characteristics of sources of evidence | 15 | For each source of evidence, present characteristics for which data were charted and provide the citations. | Supplementary file 4 - Systematic Evidence Database.xslx |

| SECTION | ITEM | PRISMA-ScR CHECKLIST ITEM | REPORTED ON PAGE # |
|---|---|---|---|
| Critical appraisal within sources of evidence | 16 | If done, present data on critical appraisal of included sources of evidence (see item 12). | Complete section under the title of **Risk of bias and study outcomes** in the manuscript and Supplementary file 5 – Supplementary Results Map.pdf |
| Results of individual sources of evidence | 17 | For each included source of evidence, present the relevant data that were charted that relate to the review questions and objectives. | Refer to results presented under the title **Review Findings** in the manuscript, as well as the Supplementary file 5 – Supplementary Results Map.pdf |
| Synthesis of results | 18 | Summarise and/or present the charting results as they relate to the review questions and objectives. | Refer to results presented under the title **Review Findings** in the manuscript, as well as the Supplementary file 5 – Supplementary Results Map.pdf |
| **DISCUSSION** | | | |
| Summary of evidence | 19 | Summarise the main results (including an overview of concepts, themes, and types of evidence available), link to the review questions and objectives, and consider the relevance to key groups. | The primary question and all the secondary questions are answered in the **Review Findings** section of the manuscript. |
| Limitations | 20 | Discuss the limitations of the scoping review process. | Discussed in the manuscript under the title **Research limitations and gaps** |
| Conclusions | 21 | Provide a general interpretation of the results with respect to the review questions and objectives, as well as potential implications and/or next steps. | Discussed in the manuscript under the title **Conclusions** |
| **FUNDING** | | | |
| Funding | 22 | Describe sources of funding for the included sources of evidence, as well as sources of funding for the scoping review. Describe the role of the funders of the scoping review. | Funding for papers reviewed has been identified (when declared) in Supplementary file 4 - Systematic Evidence Database.xslx<br>Funding declaration of the authors of this scoping review is also provided in the manuscript. |

JBI = Joanna Briggs Institute; PRISMA-ScR = Preferred Reporting Items for Systematic reviews and Meta-Analyses extension for Scoping Reviews.

* Where *sources of evidence* (see second footnote) are compiled from, such as bibliographic databases, social media platforms, and Web sites.

† A more inclusive/heterogeneous term used to account for the different types of evidence or data sources (e.g., quantitative and/or qualitative research, expert opinion, and policy documents) that may be eligible in a scoping review as opposed to only studies. This is not to be confused with *information sources* (see first footnote).

‡ The frameworks by Arksey and O'Malley (6) and Levac and colleagues (7) and the JBI guidance (4, 5) refer to the process of data extraction in a scoping review as data charting.

§ The process of systematically examining research evidence to assess its validity, results, and relevance before using it to inform a decision. This term is used for items 12 and 19 instead of "risk of bias" (which is more applicable to systematic reviews of interventions) to include and acknowledge the various sources of evidence that may be used in a scoping review (e.g., quantitative and/or qualitative research, expert opinion, and policy document).

*From:* Tricco AC, Lillie E, Zarin W, O'Brien KK, Colquhoun H, Levac D, et al. PRISMA Extension for Scoping Reviews (PRISMAScR): Checklist and Explanation. Ann Intern Med. 2018;169:467–473. doi: 10.7326/M18-0850.
