# Supplementary material for: A scoping review and evidence map of radiofrequency field exposure and genotoxicity: assessing in vivo, in vitro, and epidemiological data
Source: Front Public Health. 2025 Jul 30;13:1613353. doi: 10.3389/fpubh.2025.1613353 (PMC12343714; doi:10.3389/fpubh.2025.1613353)
Supplement: Supplementary file 3 [file Data_Sheet_3.zip › Search data/EMF Portal Search - Chromosome aberrations.docx]

EMF Portal search keywords

The following terms were included: "chromosome aberration", Chromosomenaberration, Chromosomenmutation, Aberration, 染色体異常, "chromosome exchange", "chromosome inversion", Chromosomeninversion, Inversion, 染色体逆位, 逆位, "chromosome deletion", Chromosomendeletion, 染色体欠失, "chromosome break", Chromosomenbruch, 染色体切断, "chromatid break", "dicentric chromosome", "dizentrisches Chromosom", 二動原体染色体, "acentric chromosome", "ring chromosome", Ringchromosom, 環状染色体

TY - JOUR

IS - 1

JO - Protoplasma

PY - 2023

SN - 0033-183X

VL - 260

AU - Sharma S

AU - Bahel S

AU - Kaur Katnoria J

DO - 10.1007/s00709-022-01768-9

LA - en

N1 - FEMU ID: 47420; EMF-Portal URL: https://www.emf-portal.org/en/article/47420

SP - 209-224

TI - Evaluation of oxidative stress and genotoxicity of 900 MHz electromagnetic radiations using Trigonella foenum-graecum test system

ER -

TY - JOUR

JA - Sci Rep

JO - Scientific Reports

PY - 2022

SN - 2045-2322

VL - 12

AU - Chae KS

AU - Kim SC

AU - Kwon HJ

AU - Kim Y

DO - 10.1038/s41598-022-12460-6

LA - en

N1 - FEMU ID: 47554; EMF-Portal URL: https://www.emf-portal.org/en/article/47554

SP - 8997

TI - Human magnetic sense is mediated by a light and magnetic field resonance-dependent mechanism

UR - https://www.nature.com/articles/s41598-022-12460-6.pdf

ER -

TY - JOUR

IS - 1

JA - J Indian Soc Pedod Prev Dent

JO - Journal of the Indian Society of Pedodontics and Preventive Dentistry

PY - 2022

SN - 0970-4388

VL - 40

AU - Srujana Aravinda VS

AU - Kandregula CR

AU - Muppa R

AU - Krishna MM

AU - Nikitha BS

AU - Yenni M

DO - 10.4103/jisppd.jisppd_28_22

LA - en

N1 - FEMU ID: 47334; EMF-Portal URL: https://www.emf-portal.org/en/article/47334

SP - 74-80

TI - A cross-sectional and histological analysis to understand the cytological effects of cell phone radiation on buccal mucosa of children

UR - https://www.jisppd.com/temp/JIndianSocPedodPrevDent40174-327673_090607.pdf

ER -

TY - JOUR

JA - Sci Rep

JO - Scientific Reports

PY - 2021

SN - 2045-2322

VL - 11

AU - Peltek S

AU - Meshcheryakova I

AU - Kiseleva E

AU - Oshchepkov D

AU - Rozanov A

AU - Serdyukov D

AU - Demidov E

AU - Vasiliev G

AU - Vinokurov N

AU - Bryanskaya A

AU - Bannikova S

AU - Popik V

AU - Goryachkovskaya T

DO - 10.1038/s41598-021-99665-3

LA - en

N1 - FEMU ID: 45803; EMF-Portal URL: https://www.emf-portal.org/en/article/45803

SP - 20464

TI - E. coli aggregation and impaired cell division after terahertz irradiation

UR - https://www.nature.com/articles/s41598-021-99665-3.pdf

ER -

TY - JOUR

IS - 6

JA - Gen Physiol Biophys

JO - General Physiology and Biophysics

PY - 2020

SN - 0231-5882

VL - 39

AU - Panagopoulos DJ

DO - 10.4149/gpb_2020036

LA - en

N1 - FEMU ID: 43746; EMF-Portal URL: https://www.emf-portal.org/en/article/43746

SP - 531-544

TI - Comparing chromosome damage induced by mobile telephony radiation and a high caffeine dose: Effect of combination and exposure duration

ER -

TY - JOUR

JA - Ecotoxicol Environ Saf

JO - Ecotoxicology and Environmental Safety

PY - 2020

SN - 0147-6513

VL - 188

AU - Kumar A

AU - Kaur S

AU - Chandel S

AU - Singh HP

AU - Batish DR

AU - Kohli RK

DO - 10.1016/j.ecoenv.2019.109786

LA - en

N1 - FEMU ID: 40396; EMF-Portal URL: https://www.emf-portal.org/en/article/40396

SP - 109786

TI - Comparative cyto- and genotoxicity of 900 MHz and 1800 MHz electromagnetic field radiations in root meristems of Allium cepa

ER -

TY - JOUR

IS - 5

JA - Gen Physiol Biophys

JO - General Physiology and Biophysics

PY - 2019

SN - 0231-5882

VL - 38

AU - Panagopoulos DJ

DO - 10.4149/gpb_2019032

LA - en

N1 - FEMU ID: 39526; EMF-Portal URL: https://www.emf-portal.org/en/article/39526

SP - 445-454

TI - Chromosome damage in human cells induced by UMTS mobile telephony radiation

ER -

TY - JOUR

IS - 1

JA - J Environ Health Sci Eng

JO - Journal of Environmental Health Science & Engineering

PY - 2019

VL - 17

AU - Chandel S

AU - Kaur S

AU - Issa M

AU - Singh HP

AU - Batish DR

AU - Kohli RK

DO - 10.1007/s40201-018-00330-1

LA - en

N1 - FEMU ID: 38862; EMF-Portal URL: https://www.emf-portal.org/en/article/38862

SP - 97-104

TI - Exposure to mobile phone radiations at 2350 MHz incites cyto- and genotoxic effects in root meristems of Allium cepa

UR - https://link.springer.com/content/pdf/10.1007/s40201-018-00330-1.pdf

ER -

TY - JOUR

IS - 5

JO - Protoplasma

PY - 2019

SN - 0033-183X

VL - 256

AU - Chandel S

AU - Kaur S

AU - Issa M

AU - Singh HP

AU - Batish DR

AU - Kohli RK

DO - 10.1007/s00709-019-01386-y

LA - en

N1 - FEMU ID: 38426; EMF-Portal URL: https://www.emf-portal.org/en/article/38426

SP - 1399-1407

TI - Appraisal of immediate and late effects of mobile phone radiations at 2100 MHz on mitotic activity and DNA integrity in root meristems of Allium cepa

ER -

TY - JOUR

JA - Environ Res

JO - Environmental Research

PY - 2019

SN - 0013-9351

VL - 174

AU - Jooyan N

AU - Goliaei B

AU - Bigdeli B

AU - Faraji-Dana R

AU - Zamani A

AU - Entezami M

AU - Mortazavi SMJ

DO - 10.1016/j.envres.2019.03.063

LA - en

N1 - FEMU ID: 38182; EMF-Portal URL: https://www.emf-portal.org/en/article/38182

SP - 176-187

TI - Direct and indirect effects of exposure to 900 MHz GSM radiofrequency electromagnetic fields on CHO cell line: Evidence of bystander effect by non-ionizing radiation

ER -

TY - JOUR

JA - Toxicol Appl Pharmacol

JO - Toxicology and Applied Pharmacology

PY - 2019

SN - 0041-008X

VL - 370

AU - Shahin NN

AU - El-Nabarawy NA

AU - Gouda AS

AU - Mégarbane B

DO - 10.1016/j.taap.2019.03.009

LA - en

N1 - FEMU ID: 37751; EMF-Portal URL: https://www.emf-portal.org/en/article/37751

SP - 117-130

TI - The protective role of spermine against male reproductive aberrations induced by exposure to electromagnetic field - An experimental investigation in the rat

ER -

TY - JOUR

IS - 6

JA - Int J Environ Sci Technol

JO - International Journal of Environmental Science and Technology

PY - 2018

SN - 1735-1472

VL - 15

AU - Răcuciu M

AU - Iftode C

AU - Miclăuş S

DO - 10.1007/s13762-017-1490-0

LA - en

N1 - FEMU ID: 49003; EMF-Portal URL: https://www.emf-portal.org/en/article/49003

SP - 1233-1242

TI - Influence of 1 GHz radiation at low specific absorption rate of energy deposition on plant mitotic division process

ER -

TY - JOUR

IS - 6

JO - Bioelectromagnetics

PY - 2017

SN - 0197-8462

VL - 38

AU - Suzuki S

AU - Okutsu M

AU - Suganuma R

AU - Komiya H

AU - Nakatani-Enomoto S

AU - Kobayashi S

AU - Ugawa Y

AU - Tateno H

AU - Fujimori K

DO - 10.1002/bem.22063

LA - en

N1 - FEMU ID: 32274; EMF-Portal URL: https://www.emf-portal.org/en/article/32274

SP - 466-473

TI - Influence of radiofrequency-electromagnetic waves from 3rd-generation cellular phones on fertilization and embryo development in mice

ER -

TY - JOUR

IS - 4

JA - Saudi J Biol Sci

JO - Saudi Journal of Biological Sciences

PY - 2017

SN - 1319-562X

VL - 24

AU - Qureshi ST

AU - Memon SA

AU - Abassi AR

AU - Sial MA

AU - Bughio FA

DO - 10.1016/j.sjbs.2016.02.011

LA - en

N1 - FEMU ID: 31864; EMF-Portal URL: https://www.emf-portal.org/en/article/31864

SP - 883-891

TI - Radiofrequency radiations induced genotoxic and carcinogenic effects on chickpea (Cicer arietinum L.) root tip cells

UR - https://www.sciencedirect.com/science/article/pii/S1319562X16000589/pdfft?md5=275ab70a0cf42609a2a27cd618810be3&pid=1-s2.0-S1319562X16000589-main.pdf

ER -

TY - JOUR

JA - Toxicol In Vitro

JO - Toxicology in Vitro

PY - 2017

SN - 0887-2333

VL - 40

AU - Al-Serori H

AU - Kundi M

AU - Ferk F

AU - Mišík M

AU - Nersesyan A

AU - Murbach M

AU - Lah TT

AU - Knasmüller S

DO - 10.1016/j.tiv.2017.01.012

LA - en

N1 - FEMU ID: 31101; EMF-Portal URL: https://www.emf-portal.org/en/article/31101

SP - 264-271

TI - Evaluation of the potential of mobile phone specific electromagnetic fields (UMTS) to produce micronuclei in human glioblastoma cell lines

ER -

TY - JOUR

IS - 12

JO - Environmental Engineering and Management Journal

PY - 2016

VL - 15

AU - Răcuciu M

AU - Iftode C

AU - Miclăuş S

DO - 10.30638/eemj.2016.282

LA - en

N1 - FEMU ID: 49001; EMF-Portal URL: https://www.emf-portal.org/en/article/49001

SP - 2561-2568

TI - Athermal Microwave Radiation Affects the Genetic of Vegetal Embryos

ER -

TY - JOUR

IS - 2

JA - Toxicol Res

JO - Toxicological Research

PY - 2016

SN - 1976-8257

VL - 32

AU - Nirwane A

AU - Sridhar V

AU - Majumdar A

DO - 10.5487/TR.2016.32.2.123

LA - en

N1 - FEMU ID: 29387; EMF-Portal URL: https://www.emf-portal.org/en/article/29387

SP - 123-132

TI - Neurobehavioural Changes and Brain Oxidative Stress Induced by Acute Exposure to GSM900 Mobile Phone Radiations in Zebrafish (Danio rerio)

UR - https://www.ncbi.nlm.nih.gov/pmc/articles/PMC4843974/pdf/tr-32-123.pdf

ER -

TY - JOUR

JA - Mutat Res Genet Toxicol Environ Mutagen

JO - Mutation Research - Genetic Toxicology and Environmental Mutagenesis

PY - 2015

VL - 793

AU - Amicis A

AU - Sanctis S

AU - Cristofaro SD

AU - Franchini V

AU - Lista F

AU - Regalbuto E

AU - Giovenale E

AU - Gallerano GP

AU - Nenzi P

AU - Bei R

AU - Fantini M

AU - Benvenuto M

AU - Masuelli L

AU - Coluzzi E

AU - Cicia C

AU - Sgura A

DO - 10.1016/j.mrgentox.2015.06.003

LA - en

N1 - FEMU ID: 28170; EMF-Portal URL: https://www.emf-portal.org/en/article/28170

SP - 150-160

TI - Biological effects of in vitro THz radiation exposure in human foetal fibroblasts

ER -

TY - JOUR

IS - 8

JA - Int J Radiat Biol

JO - International Journal of Radiation Biology

PY - 2015

SN - 0955-3002

VL - 91

AU - Kumar G

AU - McIntosh RL

AU - Anderson V

AU - McKenzie RJ

AU - Wood AW

DO - 10.3109/09553002.2015.1047988

LA - en

N1 - FEMU ID: 27083; EMF-Portal URL: https://www.emf-portal.org/en/article/27083

SP - 664-672

TI - A genotoxic analysis of the hematopoietic system after mobile phone type radiation exposure in rats

ER -

TY - JOUR

JA - Sci Rep

JO - Scientific Reports

PY - 2015

SN - 2045-2322

VL - 5

AU - Bogomazova AN

AU - Vassina EM

AU - Goryachkovskaya TN

AU - Popik VM

AU - Sokolov AS

AU - Kolchanov NA

AU - Lagarkova MA

AU - Kiselev SL

AU - Peltek SE

DO - 10.1038/srep07749

LA - en

N1 - FEMU ID: 26377; EMF-Portal URL: https://www.emf-portal.org/en/article/26377

SP - 7749

TI - No DNA damage response and negligible genome-wide transcriptional changes in human embryonic stem cells exposed to terahertz radiation

UR - http://www.nature.com/srep/2015/150113/srep07749/pdf/srep07749.pdf

ER -

TY - JOUR

IS - 11

JA - Int J Radiat Biol

JO - International Journal of Radiation Biology

PY - 2013

SN - 0955-3002

VL - 89

AU - Atli Sekeroglu Z

AU - Akar A

AU - Sekeroglu V

DO - 10.3109/09553002.2013.809170

LA - en

N1 - FEMU ID: 22646; EMF-Portal URL: https://www.emf-portal.org/en/article/22646

SP - 985-992

TI - Evaluation of the cytogenotoxic damage in immature and mature rats exposed to 900 MHz radiofrequency electromagnetic fields

ER -

TY - JOUR

IS - 10

JA - Int J Radiat Biol

JO - International Journal of Radiation Biology

PY - 2013

SN - 0955-3002

VL - 89

AU - Szerencsi A

AU - Kubinyi G

AU - Valiczko E

AU - Juhasz P

AU - Rudas G

AU - Mester A

AU - Janossy G

AU - Bakos J

AU - Thuroczy G

DO - 10.3109/09553002.2013.804962

LA - en

N1 - FEMU ID: 22464; EMF-Portal URL: https://www.emf-portal.org/en/article/22464

SP - 870-876

TI - DNA Integrity of Human Leukocytes after Magnetic Resonance Imaging

ER -

TY - JOUR

IS - 2

JA - Radiat Res

JO - Radiation Research

PY - 2013

SN - 0033-7587

VL - 179

AU - Waldmann P

AU - Bohnenberger S

AU - Greinert R

AU - Hermann-Then B

AU - Heselich A

AU - Klug SJ

AU - Koenig J

AU - Kuhr K

AU - Kuster N

AU - Merker M

AU - Murbach M

AU - Pollet D

AU - Schadenboeck W

AU - Scheidemann-Wesp U

AU - Schwab B

AU - Volkmer B

AU - Weyer V

AU - Blettner M

DO - 10.1667/RR2914.1

LA - en

N1 - FEMU ID: 21641; EMF-Portal URL: https://www.emf-portal.org/en/article/21641

SP - 243-253

TI - Influence of GSM Signals on Human Peripheral Lymphocytes: Study of Genotoxicity

ER -

TY - JOUR

IS - 3

JA - Arch Microbiol

JO - Archives of Microbiology

PY - 2013

SN - 0302-8933

VL - 195

AU - Berzin V

AU - Kiriukhin M

AU - Tyurin M

DO - 10.1007/s00203-012-0862-6

LA - en

N1 - FEMU ID: 21588; EMF-Portal URL: https://www.emf-portal.org/en/article/21588

SP - 181-188

TI - "Curing" of plasmid DNA in acetogen using microwave or applying an electric pulse improves cell growth and metabolite production as compared to the plasmid-harboring strain

ER -

TY - JOUR

IS - 3

JA - Int J Radiat Biol

JO - International Journal of Radiation Biology

PY - 2013

SN - 0955-3002

VL - 89

AU - Kumar S

AU - Behari J

AU - Sisodia R

DO - 10.3109/09553002.2013.741282

LA - en

N1 - FEMU ID: 21367; EMF-Portal URL: https://www.emf-portal.org/en/article/21367

SP - 147-154

TI - Influence of electromagnetic fields on reproductive system of male rats

ER -

TY - JOUR

IS - 1-2

JA - Mutat Res Genet Toxicol Environ Mutagen

JO - Mutation Research - Genetic Toxicology and Environmental Mutagenesis

PY - 2013

VL - 750

AU - Pesnya DS

AU - Romanovsky AV

DO - 10.1016/j.mrgentox.2012.08.010

LA - en

N1 - FEMU ID: 21320; EMF-Portal URL: https://www.emf-portal.org/en/article/21320

SP - 27-33

TI - Comparison of cytotoxic and genotoxic effects of plutonium-239 alpha particles and mobile phone GSM 900 radiation in the Allium cepa test

ER -

TY - JOUR

IS - 2

JA - J Appl Pharm Sci

JO - Journal of Applied Pharmaceutical Science

PY - 2012

SN - 2231-3354

VL - 2

AU - El-Abd SF

AU - Eltoweissy MY

LA - en

N1 - FEMU ID: 47219; EMF-Portal URL: https://www.emf-portal.org/en/article/47219

SP - 16-20

TI - Cytogenetic alterations in human lymphocyte culture following exposure to radiofrequency field of mobile phone

UR - https://www.japsonline.com/admin/php/uploads/372_pdf.pdf

ER -

TY - JOUR

JA - Ecotoxicol Environ Saf

JO - Ecotoxicology and Environmental Safety

PY - 2012

SN - 0147-6513

VL - 80

AU - Sekeroglu V

AU - Akar A

AU - Sekeroglu ZA

DO - 10.1016/j.ecoenv.2012.02.028

LA - en

N1 - FEMU ID: 20362; EMF-Portal URL: https://www.emf-portal.org/en/article/20362

SP - 140-144

TI - Cytotoxic and genotoxic effects of high-frequency electromagnetic fields (GSM 1800MHz) on immature and mature rats

ER -

TY - JOUR

IS - 1

JA - Int J Hyg Environ Health

JO - International Journal of Hygiene and Environmental Health

PY - 2011

SN - 1438-4639

VL - 214

AU - Garaj-Vrhovac V

AU - Gajski G

AU - Pazanin S

AU - Sarolic A

AU - Domijan AM

AU - Flajs D

AU - Peraica M

DO - 10.1016/j.ijheh.2010.08.003

LA - en

N1 - FEMU ID: 18617; EMF-Portal URL: https://www.emf-portal.org/en/article/18617

SP - 59-65

TI - Assessment of cytogenetic damage and oxidative stress in personnel occupationally exposed to the pulsed microwave radiation of marine radar equipment

ER -

TY - JOUR

IS - 6

JA - Radiat Res

JO - Radiation Research

PY - 2010

SN - 0033-7587

VL - 174

AU - Bourthoumieu S

AU - Joubert V

AU - Marin B

AU - Collin A

AU - Leveque P

AU - Terro F

AU - Yardin C

LA - en

N1 - FEMU ID: 18635; EMF-Portal URL: https://www.emf-portal.org/en/article/18635

SP - 712-718

TI - Cytogenetic Studies in Human Cells Exposed In Vitro to GSM-900 MHz Radiofrequency Radiation Using R-Banded Karyotyping

ER -

TY - JOUR

IS - 2

JA - Genet Couns

JO - Genetic Counseling

PY - 2010

SN - 1015-8146

VL - 21

AU - Yildirim MS

AU - Yildirim A

AU - Zamani AG

AU - Okudan N

LA - en

N1 - FEMU ID: 18526; EMF-Portal URL: https://www.emf-portal.org/en/article/18526

SP - 243-251

TI - Effect of mobile phone station on micronucleus frequency and chromosomal aberrations in human blood cells

ER -

TY - JOUR

IS - 11

JA - Anticancer Res

JO - Anticancer Research

PY - 2009

SN - 0250-7005

VL - 29

AU - Hansteen IL

AU - Clausen KO

AU - Haugan V

AU - Svendsen M

AU - Svendsen MV

AU - Eriksen JG

AU - Skiaker R

AU - Hauger E

AU - Lageide L

AU - Vistnes AI

AU - Kure EH

LA - en

N1 - FEMU ID: 17824; EMF-Portal URL: https://www.emf-portal.org/en/article/17824

SP - 4323-4330

TI - Cytogenetic effects of exposure to 2.3 GHz radiofrequency radiation on human lymphocytes in vitro

UR - http://ar.iiarjournals.org/content/29/11/4323.full.pdf+html

ER -

TY - JOUR

IS - 8

JA - Anticancer Res

JO - Anticancer Research

PY - 2009

SN - 0250-7005

VL - 29

AU - Hansteen IL

AU - Lageide L

AU - Clausen KO

AU - Haugan V

AU - Svendsen M

AU - Eriksen JG

AU - Skiaker R

AU - Hauger E

AU - Vistnes AI

AU - Kure EH

LA - en

N1 - FEMU ID: 17426; EMF-Portal URL: https://www.emf-portal.org/en/article/17426

SP - 2885-2892

TI - Cytogenetic effects of 18.0 and 16.5 GHz microwave radiation on human lymphocytes in vitro

UR - http://ar.iiarjournals.org/content/29/8/2885.full.pdf+html

ER -

TY - JOUR

IS - 2

JA - Mutat Res Genet Toxicol Environ Mutagen

JO - Mutation Research - Genetic Toxicology and Environmental Mutagenesis

PY - 2009

VL - 672

AU - Tkalec M

AU - Malaric K

AU - Pavlica M

AU - Pevalek-Kozlina B

AU - Vidakovic-Cifrek Z

DO - 10.1016/j.mrgentox.2008.09.022

LA - en

N1 - FEMU ID: 16578; EMF-Portal URL: https://www.emf-portal.org/en/article/16578

SP - 76-81

TI - Effects of radiofrequency electromagnetic fields on seed germination and root meristematic cells of Allium cepa L

ER -

TY - JOUR

IS - 8

JO - Bioelectromagnetics

PY - 2008

SN - 0197-8462

VL - 29

AU - Schrader T

AU - Münter K

AU - Kleine-Ostmann T

AU - Schmid E

DO - 10.1002/bem.20428

LA - en

N1 - FEMU ID: 16004; EMF-Portal URL: https://www.emf-portal.org/en/article/16004

SP - 626-639

TI - Spindle disturbances in human-hamster hybrid (AL) cells induced by mobile communication frequency range signals

ER -

TY - JOUR

IS - 5

JA - Radiat Res

JO - Radiation Research

PY - 2008

SN - 0033-7587

VL - 169

AU - Manti L

AU - Braselmann H

AU - Calabrese ML

AU - Massa R

AU - Pugliese M

AU - Scampoli P

AU - Sicignano G

AU - Grossi G

DO - 10.1667/RR1044.1

LA - en

N1 - FEMU ID: 15911; EMF-Portal URL: https://www.emf-portal.org/en/article/15911

SP - 575-583

TI - Effects of modulated microwave radiation at cellular telephone frequency (1.95 GHz) on X-ray-induced chromosome aberrations in human lymphocytes in vitro

ER -

TY - JOUR

IS - 3

JA - Environ Toxicol

JO - Environmental Toxicology

PY - 2008

SN - 1520-4081

VL - 23

AU - Kim JY

AU - Hong SY

AU - Lee YM

AU - Yu SA

AU - Koh WS

AU - Hong JR

AU - Son T

AU - Chang SK

AU - Lee M

DO - 10.1002/tox.20347

LA - en

N1 - FEMU ID: 15600; EMF-Portal URL: https://www.emf-portal.org/en/article/15600

SP - 319-327

TI - In vitro assessment of clastogenicity of mobile-phone radiation (835 MHz) using the alkaline comet assay and chromosomal aberration test

ER -

TY - JOUR

IS - 1

JA - Radiat Res

JO - Radiation Research

PY - 2008

SN - 0033-7587

VL - 169

AU - Mazor R

AU - Korenstein-Ilan A

AU - Barbul A

AU - Eshet Y

AU - Shahadi A

AU - Jerby E

AU - Korenstein R

DO - 10.1667/RR0872.1

LA - en

N1 - FEMU ID: 15530; EMF-Portal URL: https://www.emf-portal.org/en/article/15530

SP - 28-37

TI - Increased levels of numerical chromosome aberrations after in vitro exposure of human peripheral blood lymphocytes to radiofrequency electromagnetic fields for 72 hours

ER -

TY - JOUR

IS - 1-2

JA - Mutat Res Genet Toxicol Environ Mutagen

JO - Mutation Research - Genetic Toxicology and Environmental Mutagenesis

PY - 2007

VL - 626

AU - Speit G

AU - Schütz P

AU - Hoffmann H

DO - 10.1016/j.mrgentox.2006.08.003

LA - en

N1 - FEMU ID: 14202; EMF-Portal URL: https://www.emf-portal.org/en/article/14202

SP - 42-47

TI - Genotoxic effects of exposure to radiofrequency electromagnetic fields (RF-EMF) in cultured mammalian cells are not independently reproducible

ER -

TY - JOUR

IS - 3

JA - Radiat Res

JO - Radiation Research

PY - 2006

SN - 0033-7587

VL - 166

AU - Vijayalaxmi

DO - 10.1667/RR0643.1

LA - en

N1 - FEMU ID: 14138; EMF-Portal URL: https://www.emf-portal.org/en/article/14138

SP - 532-538

TI - Cytogenetic studies in human blood lymphocytes exposed in vitro to 2.45 GHz or 8.2 GHz radiofrequency radiation

ER -

TY - JOUR

IS - 5

JA - Int J Radiat Biol

JO - International Journal of Radiation Biology

PY - 2006

SN - 0955-3002

VL - 82

AU - Stronati L

AU - Testa A

AU - Moquet J

AU - Edwards A

AU - Cordelli E

AU - Villani P

AU - Marino C

AU - Fresegna AM

AU - Appolloni M

AU - Lloyd D

DO - 10.1080/09553000600739173

LA - en

N1 - FEMU ID: 13927; EMF-Portal URL: https://www.emf-portal.org/en/article/13927

SP - 339-346

TI - 935 MHz cellular phone radiation. An in vitro study of genotoxicity in human lymphocytes

ER -

TY - JOUR

IS - 2

JO - Mutagenesis

PY - 2006

SN - 0267-8357

VL - 21

AU - Maes A

AU - Van Gorp U

AU - Verschaeve L

DO - 10.1093/mutage/gel008

LA - en

N1 - FEMU ID: 13387; EMF-Portal URL: https://www.emf-portal.org/en/article/13387

SP - 139-142

TI - Cytogenetic investigation of subjects professionally exposed to radiofrequency radiation

UR - https://academic.oup.com/mutage/article-pdf/21/2/139/3906515/gel008.pdf

ER -

TY - JOUR

IS - 4

JA - Int J Hum Genet

JO - International Journal of Human Genetics

PY - 2005

SN - 0972-3757

VL - 5

AU - Gandhi G

AU - Singh P

LA - en

N1 - FEMU ID: 16802; EMF-Portal URL: https://www.emf-portal.org/en/article/16802

SP - 259-265

TI - Cytogenetic damage in mobile phone users: preliminary data

UR - http://www.krepublishers.com/02-Journals/IJHG/IJHG-05-0-000-000-2005-Web/IJHG-05-4-225-288-2005-Abst-PDF/IJHG-05-4-259-265-2005-210-Gandhi-G/IJHG-05-4-259-265-2005-210-Gandhi-G.pdf

ER -

TY - JOUR

IS - 2

JA - Indian J Hum Genet

JO - Indian Journal of Human Genetics

PY - 2005

SN - 1998-362X

VL - 11

AU - Gandhi G

AU - Anita

DO - 10.4103/0971-6866.16810

LA - en

N1 - FEMU ID: 16801; EMF-Portal URL: https://www.emf-portal.org/en/article/16801

SP - 99-104

TI - Genetic damage in mobile phone users: some preliminary findings

UR - http://www.bioline.org.br/pdf?hg05022

ER -

TY - JOUR

IS - 1-2

JA - Mutat Res Genet Toxicol Environ Mutagen

JO - Mutation Research - Genetic Toxicology and Environmental Mutagenesis

PY - 2005

VL - 587

AU - Komatsubara Y

AU - Hirose H

AU - Sakurai T

AU - Koyama S

AU - Suzuki Y

AU - Taki M

AU - Miyakoshi J

DO - 10.1016/j.mrgentox.2005.08.010

LA - en

N1 - FEMU ID: 12641; EMF-Portal URL: https://www.emf-portal.org/en/article/12641

SP - 114-119

TI - Effect of high-frequency electromagnetic fields with a wide range of SARs on chromosomal aberrations in murine m5S cells

ER -

TY - JOUR

IS - 12

JA - FASEB J

JO - The FASEB Journal

PY - 2005

SN - 0892-6638

VL - 19

AU - Nikolova T

AU - Czyz J

AU - Rolletschek A

AU - Blyszczuk P

AU - Fuchs J

AU - Jovtchev G

AU - Schuderer J

AU - Kuster N

AU - Wobus AM

DO - 10.1096/fj.04-3549fje

LA - en

N1 - FEMU ID: 12365; EMF-Portal URL: https://www.emf-portal.org/en/article/12365

SP - 1686-1688

TI - Electromagnetic fields affect transcript levels of apoptosis-related genes in embryonic stem cell-derived neural progenitor cells

ER -

TY - JOUR

IS - 4

JO - Bioelectromagnetics

PY - 2005

SN - 0197-8462

VL - 26

AU - Zeni O

AU - Romano M

AU - Perrotta A

AU - Lioi MB

AU - Barbieri R

AU - d'Ambrosio G

AU - Massa R

AU - Scarfi MR

DO - 10.1002/bem.20078

LA - en

N1 - FEMU ID: 11836; EMF-Portal URL: https://www.emf-portal.org/en/article/11836

SP - 258-265

TI - Evaluation of genotoxic effects in human peripheral blood leukocytes following an acute in vitro exposure to 900 MHz radiofrequency fields

ER -

TY - JOUR

IS - 3

JA - Genet Mol Biol

JO - Genetics and Molecular Biology

PY - 2004

SN - 1415-4757

VL - 27

AU - Figueiredo ABS

AU - Alves RN

AU - Ramalho AT

LA - en

N1 - FEMU ID: 14002; EMF-Portal URL: https://www.emf-portal.org/en/article/14002

SP - 460-466

TI - Cytogenetic analysis of the effects of 2.5 and 10.5 GHz microwaves on human lymphocytes

UR - http://www.scielo.br/pdf/gmb/v27n3/a24v27n3.pdf

ER -

TY - JOUR

JA - ScientificWorldJournal

JO - The Scientific World Journal

PY - 2004

SN - 1537-744X

VL - 4

AU - Koyama S

AU - Isozumi Y

AU - Suzuki Y

AU - Taki M

AU - Miyakoshi J

DO - 10.1100/tsw.2004.176

LA - en

N1 - FEMU ID: 11393; EMF-Portal URL: https://www.emf-portal.org/en/article/11393

SP - 29-40

TI - Effects of 2.45-GHz electromagnetic fields with a wide range of SARs on micronucleus formation in CHO-K1 cells

UR - http://downloads.hindawi.com/journals/tswj/2004/743762.pdf

ER -

TY - JOUR

IS - 3

JO - Bioelectromagnetics

PY - 2004

SN - 0197-8462

VL - 25

AU - Pyrpasopoulou A

AU - Kotoula V

AU - Cheva A

AU - Hytiroglou P

AU - Nikolakaki E

AU - Magras IN

AU - Xenos TD

AU - Tsiboukis TD

AU - Karkavelas G

DO - 10.1002/bem.10185

LA - en

N1 - FEMU ID: 10631; EMF-Portal URL: https://www.emf-portal.org/en/article/10631

SP - 216-227

TI - Bone morphogenetic protein expression in newborn rat kidneys after prenatal exposure to radiofrequency radiation

ER -

TY - JOUR

IS - 2-3

JA - Electromagn Biol Med

JO - Electromagnetic Biology and Medicine

PY - 2003

SN - 1536-8386

VL - 22

AU - Gadhia PK

AU - Shah T

AU - Mistry A

AU - Pithawala M

AU - Tamakuvala D

DO - 10.1081/JBC-120024624

LA - en

N1 - FEMU ID: 10674; EMF-Portal URL: https://www.emf-portal.org/en/article/10674

SP - 149-159

TI - A Preliminary Study to Assess Possible Chromosomal Damage Among Users of Digital Mobile Phones

ER -

TY - JOUR

IS - 1-2

JA - Mutat Res Genet Toxicol Environ Mutagen

JO - Mutation Research - Genetic Toxicology and Environmental Mutagenesis

PY - 2003

VL - 542

AU - Stacey M

AU - Stickley J

AU - Fox P

AU - Statler V

AU - Schoenbach K

AU - Beebe SJ

AU - Buescher S

DO - 10.1016/j.mrgentox.2003.08.006

LA - en

N1 - FEMU ID: 10503; EMF-Portal URL: https://www.emf-portal.org/en/article/10503

SP - 65-75

TI - Differential effects in cells exposed to ultra-short, high intensity electric fields: cell survival, DNA damage, and cell cycle analysis

ER -

TY - JOUR

IS - 1-2

JA - Mutat Res Genet Toxicol Environ Mutagen

JO - Mutation Research - Genetic Toxicology and Environmental Mutagenesis

PY - 2003

VL - 541

AU - Koyama S

AU - Nakahara T

AU - Wake K

AU - Taki M

AU - Isozumi Y

AU - Miyakoshi J

DO - 10.1016/j.mrgentox.2003.07.009

LA - en

N1 - FEMU ID: 10341; EMF-Portal URL: https://www.emf-portal.org/en/article/10341

SP - 81-89

TI - Effects of high frequency electromagnetic fields on micronucleus formation in CHO-K1 cells

ER -

TY - JOUR

IS - 2

JO - Bioelectromagnetics

PY - 2003

SN - 0197-8462

VL - 24

AU - Mashevich M

AU - Folkman D

AU - Kesar A

AU - Barbul A

AU - Korenstein R

AU - Jerby E

AU - Avivi L

DO - 10.1002/bem.10086

LA - en

N1 - FEMU ID: 9413; EMF-Portal URL: https://www.emf-portal.org/en/article/9413

SP - 82-90

TI - Exposure of human peripheral blood lymphocytes to electromagnetic fields associated with cellular phones leads to chromosomal instability

ER -

TY - JOUR

IS - 4

JA - Biomed Environ Sci

JO - Biomedical and Environmental Sciences

PY - 2002

SN - 0895-3988

VL - 15

AU - Zhang MB

AU - He JL

AU - Jin LF

AU - Lu DQ

LA - en

N1 - FEMU ID: 9988; EMF-Portal URL: https://www.emf-portal.org/en/article/9988

SP - 283-290

TI - Study of low-intensity 2450-MHz microwave exposure enhancing the genotoxic effects of mitomycin C using micronucleus test and comet assay in vitro

ER -

TY - JOUR

IS - 2

JO - Cytologia

PY - 2001

SN - 0011-4545

VL - 66

AU - Othman EO

AU - Aly MS

AU - El Nahas SM

DO - 10.1508/cytologia.66.117

LA - en

N1 - FEMU ID: 36938; EMF-Portal URL: https://www.emf-portal.org/en/article/36938

SP - 117-125

TI - Aneuploidy in workers occupationally exposed to electromagnetic field detected by FISH

UR - https://www.jstage.jst.go.jp/article/cytologia1929/66/2/66_2_117/_pdf/-char/en

ER -

TY - JOUR

IS - 2

JA - Acta Med Okayama

JO - Acta Medica Okayama

PY - 2001

SN - 0386-300X

VL - 55

AU - Lalic H

AU - Lekic A

AU - Radosevic-Stasic B

DO - 10.18926/AMO/32005

LA - en

N1 - FEMU ID: 8555; EMF-Portal URL: https://www.emf-portal.org/en/article/8555

SP - 117-127

TI - Comparison of chromosome aberrations in peripheral blood lymphocytes from people occupationally exposed to ionizing and radiofrequency radiation

UR - http://www.lib.okayama-u.ac.jp/www/acta/pdf/55_2_117.pdf

ER -

TY - JOUR

IS - 4

JA - Radiat Res

JO - Radiation Research

PY - 2001

SN - 0033-7587

VL - 156

AU - Vijayalaxmi

AU - Bisht KS

AU - Pickard WF

AU - Meltz ML

AU - Roti Roti JL

AU - Moros EG

DO - 10.1667/0033-7587(2001)156[0430:cdamfi]2.0.co;2

LA - en

N1 - FEMU ID: 7827; EMF-Portal URL: https://www.emf-portal.org/en/article/7827

SP - 430-433

TI - Chromosome damage and micronucleus formation in human blood lymphocytes exposed in vitro to radiofrequency radiation at a cellular telephone frequency (847.74 MHz, CDMA)

ER -

TY - JOUR

IS - 5

JA - Radiat Res

JO - Radiation Research

PY - 2001

SN - 0033-7587

VL - 156

AU - Sykes PJ

AU - McCallum BD

AU - Bangay MJ

AU - Hooker AM

AU - Morley AA

DO - 10.1667/0033-7587(2001)156[0495:eoetmr]2.0.co;2

LA - en

N1 - FEMU ID: 7646; EMF-Portal URL: https://www.emf-portal.org/en/article/7646

SP - 495-502

TI - Effect of exposure to 900 MHz radiofrequency radiation on intrachromosomal recombination in pKZ1 mice

ER -

TY - JOUR

IS - 2

JO - Bioelectromagnetics

PY - 2001

SN - 0197-8462

VL - 22

AU - Maes A

AU - Collier M

AU - Verschaeve L

LA - en

N1 - FEMU ID: 5625; EMF-Portal URL: https://www.emf-portal.org/en/article/5625

SP - 91-96

TI - Cytogenetic effects of 900 MHz (GSM) microwaves on human lymphocytes

ER -

TY - JOUR

IS - 1

JA - Radiat Res

JO - Radiation Research

PY - 2001

SN - 0033-7587

VL - 155

AU - Vijayalaxmi

AU - Leal BZ

AU - Meltz ML

AU - Pickard WF

AU - Bisht KS

AU - Roti Roti JL

AU - Straube WL

AU - Moros EG

DO - 10.1667/0033-7587(2001)155[0113:csihbl]2.0.co;2

LA - en

N1 - FEMU ID: 5187; EMF-Portal URL: https://www.emf-portal.org/en/article/5187

SP - 113-121

TI - Cytogenetic studies in human blood lymphocytes exposed in vitro to radiofrequency radiation at a cellular telephone frequency (835.62 MHz, FDMA)

ER -

TY - JOUR

IS - 5

JA - Folia Biol

JO - Folia Biologica

PY - 2000

SN - 0015-5500

VL - 46

AU - Maes A

AU - Collier M

AU - Verschaeve L

LA - en

N1 - FEMU ID: 4988; EMF-Portal URL: https://www.emf-portal.org/en/article/4988

SP - 175-180

TI - Cytogenetic investigations on microwaves emitted by a 455.7 MHz car phone

ER -

TY - JOUR

IS - 2

JO - Bioelectromagnetics

PY - 1998

SN - 0197-8462

VL - 19

AU - Pakhomova ON

AU - Belt ML

AU - Mathur SP

AU - Lee JC

AU - Akyel Y

DO - 10.1002/(sici)1521-186x(1998)19:2<128::aid-bem12>3.0.co;2-m

LA - en

N1 - FEMU ID: 2082; EMF-Portal URL: https://www.emf-portal.org/en/article/2082

SP - 128-130

TI - Ultra-wide band electromagnetic radiation does not affect UV-induced recombination and mutagenesis in yeast

ER -

TY - JOUR

IS - 6

JA - Int J Radiat Biol

JO - International Journal of Radiation Biology

PY - 1997

SN - 0955-3002

VL - 72

AU - Vijayalaxmi

AU - Mohan N

AU - Meltz ML

AU - Wittler MA

DO - 10.1080/095530097142915

LA - en

N1 - FEMU ID: 2359; EMF-Portal URL: https://www.emf-portal.org/en/article/2359

SP - 751-757

TI - Proliferation and cytogenetic studies in human blood lymphocytes exposed in vitro to 2450 MHz radiofrequency radiation

ER -

TY - JOUR

IS - 1-2

JA - Mutat Res Genet Toxicol Environ Mutagen

JO - Mutation Research - Genetic Toxicology and Environmental Mutagenesis

PY - 1997

VL - 393

AU - Maes A

AU - Collier M

AU - Van Gorp U

AU - Vandoninck S

AU - Verschaeve L

DO - 10.1016/s1383-5718(97)00100-9

LA - en

N1 - FEMU ID: 948; EMF-Portal URL: https://www.emf-portal.org/en/article/948

SP - 151-156

TI - Cytogenetic effects of 935.2-MHz (GSM) microwaves alone and in combination with mitomycin C

ER -

TY - JOUR

JO - Edition Wissenschaft

PY - 1996

VL - 4

AU - Eberle P

AU - Erdtmann-Vourliotis M

AU - Diener S

AU - Finke HG

AU - Löffelholz B

AU - Schnor A

AU - Schräder M

LA - de

N1 - FEMU ID: 9467; EMF-Portal URL: https://www.emf-portal.org/en/article/9467

SP - 2-15

TI - Zellproliferation, Schwesterchromatidaustausche, Chromosomenaberrationen, Mikrokerne und Mutationsrate des HGPRT-Locus

UR - https://d-nb.info/974863475/34

ER -

TY - JOUR

IS - 2

JA - Electro Magnetobiol

JO - Electro- and Magnetobiology

PY - 1995

SN - 1061-9526

VL - 14

AU - Maes A

AU - Collier M

AU - Slaets D

AU - Verschaeve L

LA - en

N1 - FEMU ID: 891; EMF-Portal URL: https://www.emf-portal.org/en/article/891

SP - 91-98

TI - Cytogenetic Effects of Microwaves from Mobile Communication Frequencies (954 MHz)

ER -

TY - JOUR

IS - 1-2

JO - Mutation Research - Letters

PY - 1994

VL - 328

AU - Haider T

AU - Knasmueller S

AU - Kundi M

AU - Haider M

DO - 10.1016/0165-7992(94)90069-8

LA - en

N1 - FEMU ID: 873; EMF-Portal URL: https://www.emf-portal.org/en/article/873

SP - 65-68

TI - Clastogenic effects of radiofrequency radiations on chromosomes of Tradescantia

ER -

TY - JOUR

JA - Bioelectrochem Bioenerg

JO - Bioelectrochemistry and Bioenergetics

PY - 1993

SN - 0302-4598

VL - 30

AU - Garaj-Vrhovac V

AU - Fucic A

DO - 10.1016/0302-4598(93)80091-8

LA - en

N1 - FEMU ID: 1874; EMF-Portal URL: https://www.emf-portal.org/en/article/1874

SP - 319-325

TI - The rate of elimination of chromosomal aberrations after accidental exposure to microwave radiation

ER -

TY - JOUR

IS - 6

JO - Bioelectromagnetics

PY - 1993

SN - 0197-8462

VL - 14

AU - Maes A

AU - Verschaeve L

AU - Arroyo A

AU - De Wagter C

AU - Vercruyssen L

DO - 10.1002/bem.2250140602

LA - en

N1 - FEMU ID: 889; EMF-Portal URL: https://www.emf-portal.org/en/article/889

SP - 495-501

TI - In vitro cytogenetic effects of 2450 MHz waves on human peripheral blood lymphocytes

ER -

TY - JOUR

IS - 3

JO - Mutation Research - Letters

PY - 1992

VL - 281

AU - Garaj-Vrhovac V

AU - Fucic A

AU - Horvat D

DO - 10.1016/0165-7992(92)90006-4

LA - en

N1 - FEMU ID: 876; EMF-Portal URL: https://www.emf-portal.org/en/article/876

SP - 181-186

TI - The correlation between the frequency of micronuclei and specific chromosome aberrations in human lymphocytes exposed to microwave radiation in vitro

ER -

TY - JOUR

IS - 4

JO - Mutation Research - Letters

PY - 1992

VL - 282

AU - Fucic A

AU - Garaj-Vrhovac V

AU - Skara M

AU - Dimitrovic B

DO - 10.1016/0165-7992(92)90133-3

LA - en

N1 - FEMU ID: 875; EMF-Portal URL: https://www.emf-portal.org/en/article/875

SP - 265-271

TI - X-rays, microwaves and vinyl chloride monomer: their clastogenic and aneugenic activity, using the micronucleus assay on human lymphocytes

ER -

TY - JOUR

IS - 5

JA - Med J Aust

JO - The Medical Journal of Australia

PY - 1991

SN - 0025-729X

VL - 155

AU - Garson OM

AU - McRobert TL

AU - Campbell LJ

AU - Hocking BA

AU - Gordon I

DO - 10.5694/j.1326-5377.1991.tb142282.x

LA - en

N1 - FEMU ID: 1407; EMF-Portal URL: https://www.emf-portal.org/en/article/1407

SP - 289-292

TI - A chromosomal study of workers with long-term exposure to radio-frequency radiation

ER -

TY - JOUR

IS - 3

JO - Mutation Research - Letters

PY - 1991

VL - 263

AU - Garaj-Vrhovac V

AU - Horvat D

AU - Koren Z

DO - 10.1016/0165-7992(91)90054-8

LA - en

N1 - FEMU ID: 877; EMF-Portal URL: https://www.emf-portal.org/en/article/877

SP - 143-149

TI - The relationship between colony-forming ability, chromosome aberrations and incidence of micronuclei in V79 Chinese hamster cells exposed to microwave radiation

ER -

TY - JOUR

IS - 4

JA - Period Biol

JO - Periodicum Biologorum

PY - 1990

SN - 0031-5362

VL - 92

AU - Garaj-Vrhovac V

AU - Fucic A

AU - Horvat D

LA - en

N1 - FEMU ID: 9585; EMF-Portal URL: https://www.emf-portal.org/en/article/9585

SP - 411-416

TI - Comparison of chromosome aberration and micronucleus induction in human lymphocytes after occupational exposure to vinyl chloride monomer and microwave radiation

ER -

TY - JOUR

IS - 3

JA - Radiat Res

JO - Radiation Research

PY - 1990

SN - 0033-7587

VL - 123

AU - Kerbacher JJ

AU - Meltz ML

AU - Erwin DN

LA - en

N1 - FEMU ID: 3403; EMF-Portal URL: https://www.emf-portal.org/en/article/3403

SP - 311-319

TI - Influence of radiofrequency radiation on chromosome aberrations in CHO cells and its interaction with DNA-damaging agents

ER -

TY - JOUR

IS - 2

JO - Bioelectromagnetics

PY - 1990

SN - 0197-8462

VL - 11

AU - Meltz ML

AU - Eagan P

AU - Erwin DN

DO - 10.1002/bem.2250110206

LA - en

N1 - FEMU ID: 888; EMF-Portal URL: https://www.emf-portal.org/en/article/888

SP - 149-157

TI - Proflavin and microwave radiation: absence of a mutagenic interaction

ER -

TY - JOUR

IS - 3

JO - Mutation Research - Letters

PY - 1990

VL - 243

AU - Garaj-Vrhovac V

AU - Horvat D

AU - Koren Z

DO - 10.1016/0165-7992(90)90028-i

LA - en

N1 - FEMU ID: 878; EMF-Portal URL: https://www.emf-portal.org/en/article/878

SP - 87-93

TI - The effect of microwave radiation on the cell genome

ER -

TY - JOUR

IS - 3

JA - Int J Biometeorol

JO - International Journal of Biometeorology

PY - 1987

SN - 0020-7128

VL - 31

AU - Levengood WC

DO - 10.1007/BF02188921

LA - en

N1 - FEMU ID: 2114; EMF-Portal URL: https://www.emf-portal.org/en/article/2114

SP - 185-190

TI - Non-disjunction mutations in Drosophila exposed to magnetic fields

ER -

TY - JOUR

IS - 5

JA - Int J Radiat Biol Relat Stud Phys Chem Med

JO - International Journal of Radiation Biology and Related Studies in Physics, Chemistry and Medicine

PY - 1986

SN - 0020-7616

VL - 50

AU - Beechey CV

AU - Brooker D

AU - Kowalczuk CI

AU - Saunders RD

AU - Searle AG

DO - 10.1080/09553008614551321

LA - en

N1 - FEMU ID: 1388; EMF-Portal URL: https://www.emf-portal.org/en/article/1388

SP - 909-918

TI - Cytogenetic effects of microwave irradiation on male germ cells of the mouse

ER -

TY - JOUR

IS - 2

JO - Bioelectromagnetics

PY - 1986

SN - 0197-8462

VL - 7

AU - Lloyd DC

AU - Saunders RD

AU - Moquet JE

AU - Kowalczuk CI

DO - 10.1002/bem.2250070212

LA - en

N1 - FEMU ID: 893; EMF-Portal URL: https://www.emf-portal.org/en/article/893

SP - 235-237

TI - Absence of chromosomal damage in human lymphocytes exposed to microwave radiation with hyperthermia

ER -

TY - JOUR

IS - 1

JO - Radiology

PY - 1985

SN - 0033-8419

VL - 155

AU - Wolff S

AU - James TL

AU - Young GB

AU - Margulis AR

AU - Bodycote J

AU - Afzal V

DO - 10.1148/radiology.155.1.4038809

LA - en

N1 - FEMU ID: 22347; EMF-Portal URL: https://www.emf-portal.org/en/article/22347

SP - 163-165

TI - Magnetic resonance imaging: absence of in vitro cytogenetic damage

ER -

TY - JOUR

IS - 1

JA - J Hered

JO - The Journal of Heredity

PY - 1985

SN - 0022-1503

VL - 76

AU - Manikowska-Czerska E

AU - Czerski P

AU - Leach WM

DO - 10.1093/oxfordjournals.jhered.a110027

LA - en

N1 - FEMU ID: 922; EMF-Portal URL: https://www.emf-portal.org/en/article/922

SP - 71-73

TI - Effects of 2.45 GHz microwaves on meiotic chromosomes of male CBA/CAY mice

ER -

TY - JOUR

IS - 2

JA - Int J Radiat Biol Relat Stud Phys Chem Med

JO - International Journal of Radiation Biology and Related Studies in Physics, Chemistry and Medicine

PY - 1984

SN - 0020-7616

VL - 46

AU - Lloyd DC

AU - Saunders RD

AU - Finnon P

AU - Kowalczuk CI

DO - 10.1080/09553008414551211

LA - en

N1 - FEMU ID: 13126; EMF-Portal URL: https://www.emf-portal.org/en/article/13126

SP - 135-141

TI - No clastogenic effect from in vitro microwave irradiation of G0 human lymphocytes

ER -

TY - JOUR

IS - 3

JA - J Natl Cancer Inst

JO - Journal of the National Cancer Institute

PY - 1983

SN - 0027-8874

VL - 70

AU - Banerjee R

AU - Goldfeder A

AU - Mitra J

LA - en

N1 - FEMU ID: 9033; EMF-Portal URL: https://www.emf-portal.org/en/article/9033

SP - 517-521

TI - Sister chromatid exchanges and chromosome aberrations induced by radiosensitizing agents in bone marrow cells of treated tumor-bearing mice

ER -

TY - JOUR

IS - 2

JA - J Hered

JO - The Journal of Heredity

PY - 1982

SN - 0022-1503

VL - 73

AU - Yao KT

DO - 10.1093/oxfordjournals.jhered.a109596

LA - en

N1 - FEMU ID: 927; EMF-Portal URL: https://www.emf-portal.org/en/article/927

SP - 133-138

TI - Cytogenetic consequences of microwave irradiation on mammalian cells incubated in vitro

ER -

TY - JOUR

IS - 3

JO - Experientia

PY - 1979

SN - 0014-4754

VL - 35

AU - Manikowska E

AU - Luciani JM

AU - Servantie B

AU - Czerski P

AU - Obrenovitch J

AU - Stahl A

DO - 10.1007/BF01964370

LA - en

N1 - FEMU ID: 9731; EMF-Portal URL: https://www.emf-portal.org/en/article/9731

SP - 388-390

TI - Effects of 9.4 GHz microwave exposure on meiosis in mice

ER -

TY - JOUR

IS - 6

JA - J Hered

JO - The Journal of Heredity

PY - 1978

SN - 0022-1503

VL - 69

AU - Yao KT

DO - 10.1093/oxfordjournals.jhered.a108983

LA - en

N1 - FEMU ID: 13540; EMF-Portal URL: https://www.emf-portal.org/en/article/13540

SP - 409-412

TI - Microwave radiation-induced chromosomal aberrations in corneal epithelium of Chinese hamsters

ER -

TY - JOUR

IS - 1

JA - Can J Genet Cytol

JO - Canadian Journal of Genetics and Cytology

PY - 1978

SN - 0008-4093

VL - 20

AU - Alam MT

AU - Barthakur N

AU - Lambert NG

AU - Kasatiya SS

DO - 10.1139/g78-004

LA - en

N1 - FEMU ID: 933; EMF-Portal URL: https://www.emf-portal.org/en/article/933

SP - 23-30

TI - Cytological effects of microwave radiation in Chinese hamster cells in vitro

ER -

TY - JOUR

IS - 6

JA - Radio Sci

JO - Radio Science

PY - 1977

SN - 0048-6604

VL - 12

AU - Huang AT

AU - Engle ME

AU - Elder JA

AU - Kinn JB

AU - Ward TR

DO - 10.1029/RS012i06Sp00173

LA - en

N1 - FEMU ID: 31069; EMF-Portal URL: https://www.emf-portal.org/en/article/31069

SP - 173 - 177

TI - The effect of microwave radiation (2450 MHz) on the morphology and chromosomes of lymphocytes

ER -

TY - JOUR

IS - 3

JA - Environ Res

JO - Environmental Research

PY - 1976

SN - 0013-9351

VL - 11

AU - Mittler S

DO - 10.1016/0013-9351(76)90094-3

LA - en

N1 - FEMU ID: 1410; EMF-Portal URL: https://www.emf-portal.org/en/article/1410

SP - 326-330

TI - Failure of 2- and 10-meter radio waves to induce genetic damage in Drosophila melanogaster

ER -

TY - JOUR

IS - 1

JA - Environ Lett

JO - Environmental Letters

PY - 1974

SN - 0013-9300

VL - 6

AU - Chen KM

AU - Samuel A

AU - Hoopingarner R

DO - 10.1080/00139307409437344

LA - en

N1 - FEMU ID: 7715; EMF-Portal URL: https://www.emf-portal.org/en/article/7715

SP - 37-46

TI - Chromosomal aberrations of living cells induced by microwave radiation

ER -

TY - JOUR

IS - 1

JA - J Appl Physiol

JO - Journal of Applied Physiology

PY - 1972

SN - 0021-8987

VL - 32

AU - McLees BD

AU - Finch ED

AU - Albright ML

DO - 10.1152/jappl.1972.32.1.78

LA - en

N1 - FEMU ID: 12703; EMF-Portal URL: https://www.emf-portal.org/en/article/12703

SP - 78-85

TI - An examination of regenerating hepatic tissue subjected to radio-frequency irradiation

ER -
