# Supplementary material for: A scoping review and evidence map of radiofrequency field exposure and genotoxicity: assessing in vivo, in vitro, and epidemiological data
Source: Front Public Health. 2025 Jul 30;13:1613353. doi: 10.3389/fpubh.2025.1613353 (PMC12343714; doi:10.3389/fpubh.2025.1613353)
Supplement: Supplementary file 3 [file Data_Sheet_3.zip › Search data/EMF Portal Search - Complex DNA damage search.docx]

EMF Portal search key words

The following terms were included: "DNA damage", "chromosome aberration", Chromosomenaberration, Chromosomenmutation, Aberration, 染色体異常, Mikronukleus, Mikrokern, MN, micronucleus, 小核, "sister chromatid exchange", Schwesterchromatid-Austausch, SCE, 姉妹染色分体交換, "DNA breaks", "double strand break", "single strand break", "DNA fragmentation", "DNA base damage"

TY - JOUR

JA - Front Cell Dev Biol

JO - Frontiers in Cell and Developmental Biology

PY - 2023

SN - 2296-634X

VL - 11

AU - Rana JN

AU - Mumtaz S

AU - Choi EH

AU - Han I

DO - 10.3389/fcell.2023.1067861

LA - en

N1 - FEMU ID: 50183; EMF-Portal URL: https://www.emf-portal.org/en/article/50183

SP - 1067861

TI - ROS production in response to high-power microwave pulses induces p53 activation and DNA damage in brain cells: Radiosensitivity and biological dosimetry evaluation

UR - https://www.frontiersin.org/articles/10.3389/fcell.2023.1067861/pdf

ER -

TY - JOUR

JO - Neurotoxicology

PY - 2023

SN - 0161-813X

VL - 94

AU - Singh KV

AU - Prakash C

AU - Nirala JP

AU - Nanda RK

AU - Rajamani P

DO - 10.1016/j.neuro.2022.11.001

LA - en

N1 - FEMU ID: 48916; EMF-Portal URL: https://www.emf-portal.org/en/article/48916

SP - 46-58

TI - Acute radiofrequency electromagnetic radiation exposure impairs neurogenesis and causes neuronal DNA damage in the young rat brain

ER -

TY - JOUR

IS - 1

JA - J Biophotonics

JO - Journal of Biophotonics

PY - 2023

SN - 1864-063X

VL - 16

AU - Sitnikov DS

AU - Revkova VA

AU - Ilina IV

AU - Gurova SA

AU - Komarov PS

AU - Struleva EV

AU - Konoplyannikov MA

AU - Kalsin VA

AU - Baklaushev VP

DO - 10.1002/jbio.202200212

LA - en

N1 - FEMU ID: 48706; EMF-Portal URL: https://www.emf-portal.org/en/article/48706

SP - e202200212

TI - Studying the genotoxic effects of high intensity terahertz radiation on fibroblasts and CNS tumor cells

ER -

TY - JOUR

IS - 1

JO - Protoplasma

PY - 2023

SN - 0033-183X

VL - 260

AU - Sharma S

AU - Bahel S

AU - Kaur Katnoria J

DO - 10.1007/s00709-022-01768-9

LA - en

N1 - FEMU ID: 47420; EMF-Portal URL: https://www.emf-portal.org/en/article/47420

SP - 209-224

TI - Evaluation of oxidative stress and genotoxicity of 900 MHz electromagnetic radiations using Trigonella foenum-graecum test system

ER -

TY - JOUR

JA - Biomed Pharmacother

JO - Biomedicine & Pharmacotherapy

PY - 2022

SN - 0753-3322

VL - 155

AU - Zhang Y

AU - Li Q

AU - Wang C

AU - Zhu L

AU - Wang F

AU - Jiao W

AU - Zhuang X

AU - Xie F

AU - Du L

AU - Jin Y

DO - 10.1016/j.biopha.2022.113779

LA - en

N1 - FEMU ID: 50195; EMF-Portal URL: https://www.emf-portal.org/en/article/50195

SP - 113779

TI - Cinnarizine dissolving microneedles against microwave-induced brain injury

UR - https://www.sciencedirect.com/science/article/pii/S0753332222011684/pdfft?md5=f26c25755710d42ca38db2d0ae065622&pid=1-s2.0-S0753332222011684-main.pdf

ER -

TY - GEN

ET - 1

PB - IEEE

PY - 2022

SN - 9781665471114

T2 - 2022 Microwave Mediterranean Symposium (MMS)

AU - Sannino A

AU - Scarfi MR

AU - Romeo S

AU - Priault M

AU - Dufossee M

AU - Poeta L

AU - Prouzet-Mauleon V

AU - Zeni O

DO - 10.1109/MMS55062.2022.9825588

LA - en

N1 - FEMU ID: 48629; EMF-Portal URL: https://www.emf-portal.org/en/article/48629

SP - 1-5

TI - Possible role of autophagy in in vitro radiofrequency-induced adaptive response

ER -

TY - GEN

ET - 1

PB - IEEE

PY - 2022

SN - 9781728194288

T2 - 2022 47th International Conference on Infrared, Millimeter and Terahertz Waves (IRMMW-THz)

AU - Lawler NB

AU - Evans CW

AU - Romanenko S

AU - Chaudhari N

AU - Fear M

AU - Wood F

AU - Smith NM

AU - Iyer KS

AU - Wallace VP

DO - 10.1109/IRMMW-THz50927.2022.9896043

LA - en

N1 - FEMU ID: 48491; EMF-Portal URL: https://www.emf-portal.org/en/article/48491

SP - 1-2

TI - Millimeter Waves Alter the Genomic Architecture and Transcriptome of Primary Human Fibroblasts

ER -

TY - JOUR

IS - 4

JA - Electromagn Biol Med

JO - Electromagnetic Biology and Medicine

PY - 2022

SN - 1536-8386

VL - 41

AU - Kucukbagriacik Y

AU - Dastouri M

AU - Ozgur-Buyukatalay E

AU - Akarca Dizakar O

AU - Yegin K

DO - 10.1080/15368378.2022.2117187

LA - en

N1 - FEMU ID: 48288; EMF-Portal URL: https://www.emf-portal.org/en/article/48288

SP - 389-401

TI - Investigation of oxidative damage, antioxidant balance, DNA repair genes, and apoptosis due to radiofrequency-induced adaptive response in mice

ER -

TY - JOUR

IS - 15

JA - Int J Mol Sci

JO - International Journal of Molecular Sciences

PY - 2022

SN - 1422-0067

VL - 23

AU - Sannino A

AU - Scarfì MR

AU - Dufossée M

AU - Romeo S

AU - Poeta L

AU - Prouzet-Mauléon V

AU - Priault M

AU - Zeni O

DO - 10.3390/ijms23158414

LA - en

N1 - FEMU ID: 48101; EMF-Portal URL: https://www.emf-portal.org/en/article/48101

SP - 8414

TI - Inhibition of Autophagy Negates Radiofrequency-Induced Adaptive Response in SH-SY5Y Neuroblastoma Cells

UR - https://www.mdpi.com/1422-0067/23/15/8414/pdf?version=1659342995

ER -

TY - JOUR

IS - 2

JA - J Popul Ther Clin Pharmacol

JO - Journal of Population Therapeutics and Clinical Pharmacology

PY - 2022

SN - 1198-581X

VL - 29

AU - Mahmood MN

AU - Shaker AH

AU - Mohammed HE

DO - 10.47750/jptcp.2022.934

LA - en

N1 - FEMU ID: 47880; EMF-Portal URL: https://www.emf-portal.org/en/article/47880

SP - E79-E87

TI - Estimation of some antioxidants in people exposed to electromagnetic waves from Internet towers in Samarra

UR - https://www.jptcp.com/index.php/jptcp/article/view/934/895

ER -

TY - JOUR

IS - 5

JA - Biomed Opt Express

JO - Biomedical Optics Express

PY - 2022

SN - 2156-7085

VL - 13

AU - Lawler NB

AU - Evans CW

AU - Romanenko S

AU - Chaudhari N

AU - Fear M

AU - Wood F

AU - Smith NM

AU - Wallace VP

AU - Swaminathan Iyer K

DO - 10.1364/BOE.458478

LA - en

N1 - FEMU ID: 47810; EMF-Portal URL: https://www.emf-portal.org/en/article/47810

SP - 3131-3144

TI - Millimeter waves alter DNA secondary structures and modulate the transcriptome in human fibroblasts

UR - https://opg.optica.org/boe/viewmedia.cfm?uri=boe-13-5-3131&seq=0

ER -

TY - JOUR

IS - 1

JA - J Indian Soc Pedod Prev Dent

JO - Journal of the Indian Society of Pedodontics and Preventive Dentistry

PY - 2022

SN - 0970-4388

VL - 40

AU - Srujana Aravinda VS

AU - Kandregula CR

AU - Muppa R

AU - Krishna MM

AU - Nikitha BS

AU - Yenni M

DO - 10.4103/jisppd.jisppd_28_22

LA - en

N1 - FEMU ID: 47334; EMF-Portal URL: https://www.emf-portal.org/en/article/47334

SP - 74-80

TI - A cross-sectional and histological analysis to understand the cytological effects of cell phone radiation on buccal mucosa of children

UR - https://www.jisppd.com/temp/JIndianSocPedodPrevDent40174-327673_090607.pdf

ER -

TY - JOUR

IS - 9

JA - Reprod Fertil Dev

JO - Reproduction, Fertility, and Development

PY - 2022

SN - 1031-3613

VL - 34

AU - Zhang S

AU - Mo F

AU - Chang Y

AU - Wu S

AU - Ma Q

AU - Jin F

AU - Xing L

DO - 10.1071/RD21234

LA - en

N1 - FEMU ID: 47240; EMF-Portal URL: https://www.emf-portal.org/en/article/47240

SP - 669-678

TI - Effects of mobile phone use on semen parameters: a cross-sectional study of 1634 men in China

UR - https://www.publish.csiro.au/rd/pdf/RD21234

ER -

TY - JOUR

JA - Biochem Biophys Rep

JO - Biochemistry and Biophysics Reports

PY - 2022

SN - 2405-5808

VL - 30

AU - Khodamoradi E

AU - Afrashi S

AU - Khoshgard K

AU - Fathi F

AU - Shahasavari S

AU - Azmoonfar R

AU - Najafi M

DO - 10.1016/j.bbrep.2022.101232

LA - en

N1 - FEMU ID: 46940; EMF-Portal URL: https://www.emf-portal.org/en/article/46940

SP - 101232

TI - Simultaneous effect of gamma and Wi-Fi radiation on gamma-H2Ax expression in peripheral blood of rat: A radio-protection note

UR - https://www.sciencedirect.com/science/article/pii/S2405580822000334/pdfft?md5=accbc2c516212300e3b839966dbd4e82&pid=1-s2.0-S2405580822000334-main.pdf

ER -

TY - JOUR

IS - 1

JA - Egypt J Med Hum Genet

JO - Egyptian Journal of Medical Human Genetics

PY - 2022

SN - 1110-8630

VL - 23

AU - Khalil AM

AU - Al-Qaoud KM

AU - Alemam IF

AU - Okour MA

DO - 10.1186/s43042-022-00231-x

LA - en

N1 - FEMU ID: 46753; EMF-Portal URL: https://www.emf-portal.org/en/article/46753

SP - 12

TI - Mobile phone radiation might alter gene expression in the oral squamous epithelial cells

UR - https://jmhg.springeropen.com/track/pdf/10.1186/s43042-022-00231-x.pdf

ER -

TY - JOUR

IS - 1

JA - Saudi J Biol Sci

JO - Saudi Journal of Biological Sciences

PY - 2022

SN - 1319-562X

VL - 29

AU - Hasan I

AU - Jahan MR

AU - Islam MN

AU - Islam MR

DO - 10.1016/j.sjbs.2021.08.063

LA - en

N1 - FEMU ID: 46443; EMF-Portal URL: https://www.emf-portal.org/en/article/46443

SP - 102-110

TI - Effect of 2400 MHz mobile phone radiation exposure on the behavior and hippocampus morphology in Swiss mouse model

UR - https://www.sciencedirect.com/science/article/pii/S1319562X21007518/pdfft?md5=faa25562f821496cb73104fa28106e6f&pid=1-s2.0-S1319562X21007518-main.pdf

ER -

TY - JOUR

IS - 7

JA - Chin J Integr Med

JO - Chinese Journal of Integrative Medicine

PY - 2022

SN - 1672-0415

VL - 28

AU - Yin L

AU - Fan SJ

AU - Zhang MN

DO - 10.1007/s11655-021-3527-y

LA - en

N1 - FEMU ID: 46037; EMF-Portal URL: https://www.emf-portal.org/en/article/46037

SP - 620-626

TI - Protective Effects of Anthocyanins Extracted from Vaccinium Uliginosum on 661W Cells Against Microwave-Induced Retinal Damage

ER -

TY - JOUR

IS - 1

JO - Andrologia

PY - 2022

SN - 0303-4569

VL - 54

AU - Hassanzadeh-Taheri M

AU - Khalili MA

AU - Hosseininejad Mohebati A

AU - Zardast M

AU - Hosseini M

AU - Palmerini MG

AU - Doostabadi MR

DO - 10.1111/and.14257

LA - en

N1 - FEMU ID: 45775; EMF-Portal URL: https://www.emf-portal.org/en/article/45775

SP - e14257

TI - The detrimental effect of cell phone radiation on sperm biological characteristics in normozoospermic

ER -

TY - JOUR

IS - 4

JA - J Int Oral Health

JO - Journal of International Oral Health

PY - 2021

SN - 0976-1799

VL - 13

AU - Thamilselvan S

AU - Behera A

AU - Nair SK

AU - Chandru CSL

AU - Krishnakumar M

AU - Ramani P

DO - 10.4103/JIOH.JIOH_358_20

LA - en

N1 - FEMU ID: 48844; EMF-Portal URL: https://www.emf-portal.org/en/article/48844

SP - 350-355

TI - Micronuclei analysis in people residing within 25 m of radiation-exposed areas around mobile towers in Chennai, India: An observational study

ER -

TY - JOUR

IS - 1

JA - J Clin of Diagn Res

JO - Journal of Clinical and Diagnostic Research

PY - 2021

SN - 0973-709X

VL - 15

AU - D'Silva MH

AU - Swer RT

AU - Anbalagan J

AU - Bhargavan R

DO - 10.7860/JCDR/2021/47115.14441

LA - en

N1 - FEMU ID: 48839; EMF-Portal URL: https://www.emf-portal.org/en/article/48839

SP - AC01-AC04

TI - Assessment of DNA Damage in Chick Embryo Brains Exposed to 2G and 3G Cell Phone Radiation using Alkaline Comet Assay Technique

UR - https://jcdr.net/article_fulltext.asp?issn=0973-709x&year=2021&volume=15&issue=1&page=AC01&issn=0973-709x&id=14441

ER -

TY - JOUR

JA - Int J Prev Med

JO - International Journal of Preventive Medicine

PY - 2021

SN - 2008-7802

VL - 12

AU - Ghandehari M

AU - Sadri D

AU - Farhadi S

DO - 10.4103/ijpvm.IJPVM_489_19

LA - en

N1 - FEMU ID: 46095; EMF-Portal URL: https://www.emf-portal.org/en/article/46095

SP - 125

TI - Micronucleus Assay in Cell Phone Users: Importance of Oral Mucosa Screening

UR - https://www.ijpvmjournal.net/temp/IntJPrevMed121125-3231313_085833.pdf

ER -

TY - JOUR

IS - 11

JA - Biomed Opt Express

JO - Biomedical Optics Express

PY - 2021

SN - 2156-7085

VL - 12

AU - Sitnikov DS

AU - Ilina IV

AU - Revkova VA

AU - Rodionov SA

AU - Gurova SA

AU - Shatalova RO

AU - Kovalev AV

AU - Ovchinnikov AV

AU - Chefonov OV

AU - Konoplyannikov MA

AU - Kalsin VA

AU - Baklaushev VP

DO - 10.1364/BOE.440460

LA - en

N1 - FEMU ID: 46080; EMF-Portal URL: https://www.emf-portal.org/en/article/46080

SP - 7122-7138

TI - Effects of high intensity non-ionizing terahertz radiation on human skin fibroblasts

UR - https://www.osapublishing.org/boe/viewmedia.cfm?uri=boe-12-11-7122&seq=0

ER -

TY - JOUR

JA - Sci Rep

JO - Scientific Reports

PY - 2021

SN - 2045-2322

VL - 11

AU - Peltek S

AU - Meshcheryakova I

AU - Kiseleva E

AU - Oshchepkov D

AU - Rozanov A

AU - Serdyukov D

AU - Demidov E

AU - Vasiliev G

AU - Vinokurov N

AU - Bryanskaya A

AU - Bannikova S

AU - Popik V

AU - Goryachkovskaya T

DO - 10.1038/s41598-021-99665-3

LA - en

N1 - FEMU ID: 45803; EMF-Portal URL: https://www.emf-portal.org/en/article/45803

SP - 20464

TI - E. coli aggregation and impaired cell division after terahertz irradiation

UR - https://www.nature.com/articles/s41598-021-99665-3.pdf

ER -

TY - JOUR

IS - 4

JA - Vet Arhiv

JO - Veterinarski Arhiv

PY - 2021

SN - 0372-5480

VL - 91

AU - Vilić M

AU - Žura Žaja I

AU - Tkalec M

AU - Štambuk A

AU - Šrut M

AU - Klobučar G

AU - Malarić K

AU - Tucak P

AU - Pašić S

AU - Tlak Gajger I

DO - 10.24099/vet.arhiv.1321

LA - en

N1 - FEMU ID: 45784; EMF-Portal URL: https://www.emf-portal.org/en/article/45784

SP - 427-435

TI - Effects of a radio frequency electromagnetic field on honey bee larvae (Apis mellifera) differ in relation to the experimental study design

UR - http://vetarhiv.vef.unizg.hr/papers/2021-91-4-9.pdf

ER -

TY - JOUR

JA - Front Public Health

JO - Frontiers in Public Health

PY - 2021

SN - 2296-2565

VL - 9

AU - Sieroń K

AU - Knapik K

AU - Onik G

AU - Romuk E

AU - Birkner E

AU - Kwiatek S

AU - Sieroń A

DO - 10.3389/fpubh.2021.710484

LA - en

N1 - FEMU ID: 45739; EMF-Portal URL: https://www.emf-portal.org/en/article/45739

SP - 710484

TI - Electromagnetic Fields Modify Redox Balance in the Rat Gastrointestinal Tract

UR - https://www.frontiersin.org/articles/10.3389/fpubh.2021.710484/pdf

ER -

TY - JOUR

IS - 12

JA - Chronobiol Int

JO - Chronobiology International

PY - 2021

SN - 0742-0528

VL - 38

AU - Qin F

AU - Cao H

AU - Feng C

AU - Zhu T

AU - Zhu B

AU - Zhang J

AU - Tong J

AU - Pei H

DO - 10.1080/07420528.2021.1962902

LA - en

N1 - FEMU ID: 45430; EMF-Portal URL: https://www.emf-portal.org/en/article/45430

SP - 1745-1760

TI - Microarray profiling of LncRNA expression in the testis of pubertal mice following morning and evening exposure to 1800 MHz radiofrequency fields

ER -

TY - JOUR

JA - Sci Rep

JO - Scientific Reports

PY - 2021

SN - 2045-2322

VL - 11

AU - Shaw P

AU - Kumar N

AU - Mumtaz S

AU - Lim JS

AU - Jang JH

AU - Kim D

AU - Sahu BD

AU - Bogaerts A

AU - Choi EH

DO - 10.1038/s41598-021-93274-w

LA - en

N1 - FEMU ID: 45231; EMF-Portal URL: https://www.emf-portal.org/en/article/45231

SP - 14003

TI - Evaluation of non-thermal effect of microwave radiation and its mode of action in bacterial cell inactivation

UR - https://www.nature.com/articles/s41598-021-93274-w.pdf

ER -

TY - JOUR

IS - 20

JA - J Toxicol Environ Health A

JO - Journal of Toxicology and Environmental Health, Part A

PY - 2021

SN - 0098-4108

VL - 84

AU - Kim JH

AU - Jeon S

AU - Choi HD

AU - Lee JH

AU - Bae JS

AU - Kim N

AU - Kim HG

AU - Kim KB

AU - Kim HR

DO - 10.1080/15287394.2021.1944944

LA - en

N1 - FEMU ID: 45194; EMF-Portal URL: https://www.emf-portal.org/en/article/45194

SP - 846-857

TI - Exposure to long-term evolution radiofrequency electromagnetic fields decreases neuroblastoma cell proliferation via Akt/mTOR-mediated cellular senescence

ER -

TY - JOUR

JA - Sci Rep

JO - Scientific Reports

PY - 2021

SN - 2045-2322

VL - 11

AU - Ioniţă E

AU - Marcu A

AU - Temelie M

AU - Savu D

AU - Şerbănescu M

AU - Ciubotaru M

DO - 10.1038/s41598-021-91790-3

LA - en

N1 - FEMU ID: 45135; EMF-Portal URL: https://www.emf-portal.org/en/article/45135

SP - 12651

TI - Radiofrequency EMF irradiation effects on pre-B lymphocytes undergoing somatic recombination

UR - https://www.nature.com/articles/s41598-021-91790-3.pdf

ER -

TY - JOUR

IS - 10

JA - Int J Mol Sci

JO - International Journal of Molecular Sciences

PY - 2021

SN - 1422-0067

VL - 22

AU - Jin H

AU - Kim K

AU - Park GY

AU - Kim M

AU - Lee HJ

AU - Jeon S

AU - Kim JH

AU - Kim HR

AU - Lim KM

AU - Lee YS

DO - 10.3390/ijms22105134

LA - en

N1 - FEMU ID: 45005; EMF-Portal URL: https://www.emf-portal.org/en/article/45005

SP - 5134

TI - The Protective Effects of EMF-LTE against DNA Double-Strand Break Damage In Vitro and In Vivo

UR - https://www.mdpi.com/1422-0067/22/10/5134/pdf

ER -

TY - JOUR

JA - Environ Res

JO - Environmental Research

PY - 2021

SN - 0013-9351

VL - 196

AU - Zeni O

AU - Romeo S

AU - Sannino A

AU - Palumbo R

AU - Scarfì MR

DO - 10.1016/j.envres.2021.110935

LA - en

N1 - FEMU ID: 44355; EMF-Portal URL: https://www.emf-portal.org/en/article/44355

SP - 110935

TI - Evidence of bystander effect induced by radiofrequency radiation in a human neuroblastoma cell line

ER -

TY - JOUR

IS - 2

JA - Electromagn Biol Med

JO - Electromagnetic Biology and Medicine

PY - 2021

SN - 1536-8386

VL - 40

AU - Gunes M

AU - Ates K

AU - Yalcin B

AU - Akkurt S

AU - Ozen S

AU - Kaya B

DO - 10.1080/15368378.2021.1878210

LA - en

N1 - FEMU ID: 44328; EMF-Portal URL: https://www.emf-portal.org/en/article/44328

SP - 254-263

TI - An Evaluation of the Genotoxic Effects of Electromagnetic Radiation at 900 MHz, 1800 MHz, and 2100 MHz Frequencies with a SMART Assay in Drosophila melanogaster

ER -

TY - JOUR

JA - Sci Rep

JO - Scientific Reports

PY - 2021

SN - 2045-2322

VL - 11

AU - Liu YC

AU - Ke L

AU - Yang SWQ

AU - Nan Z

AU - Teo EPW

AU - Lwin NC

AU - Lin MT

AU - Lee IXY

AU - Chan AS

AU - Schmetterer L

AU - Mehta JS

DO - 10.1038/s41598-021-82103-9

LA - en

N1 - FEMU ID: 44105; EMF-Portal URL: https://www.emf-portal.org/en/article/44105

SP - 2448

TI - Safety profiles of terahertz scanning in ophthalmology

UR - https://www.nature.com/articles/s41598-021-82103-9.pdf

ER -

TY - JOUR

IS - 1

JO - Bioelectromagnetics

PY - 2021

SN - 0197-8462

VL - 42

AU - Alkis ME

AU - Akdag MZ

AU - Dasdag S

DO - 10.1002/bem.22315

LA - en

N1 - FEMU ID: 43930; EMF-Portal URL: https://www.emf-portal.org/en/article/43930

SP - 76-85

TI - Effects of Low-Intensity Microwave Radiation on Oxidant-Antioxidant Parameters and DNA Damage in the Liver of Rats

ER -

TY - JOUR

IS - 1

JO - All Life

PY - 2020

VL - 13

AU - Zhu S

AU - Zhu Y

AU - Li H

AU - Zhang D

AU - Zhang D

DO - 10.1080/26895293.2020.1763481

LA - en

N1 - FEMU ID: 46015; EMF-Portal URL: https://www.emf-portal.org/en/article/46015

SP - 252-258

TI - The toxic effect of mobile phone radiation on rabbit organs

UR - https://www.tandfonline.com/doi/pdf/10.1080/26895293.2020.1763481

ER -

TY - GEN

ET - 1

PB - IEEE

PY - 2020

SN - 9781728158679

T2 - 2020 IEEE MTT-S International Microwave Biomedical Conference (IMBioC), Toulouse, France

AU - Kolosnjaj-Tabi J

AU - Alberola G

AU - Augé S

AU - Tamra A

AU - Dubuc D

AU - Grenier K

AU - Rols MP

DO - 10.1109/IMBIoC47321.2020.9385011

LA - en

N1 - FEMU ID: 44633; EMF-Portal URL: https://www.emf-portal.org/en/article/44633

SP - 1-3

TI - Evaluation of Cell Membrane Effects After 3D Multicellular Spheroids RF Exposure

ER -

TY - JOUR

IS - 5

JA - Indian J Dent Res

JO - Indian Journal of Dental Research

PY - 2020

SN - 0970-9290

VL - 31

AU - Rashmi B

AU - Chinna SK

AU - Rodrigues C

AU - Anjaly D

AU - Bankur PK

AU - Kannaiyan K

DO - 10.4103/ijdr.IJDR_634_18

LA - en

N1 - FEMU ID: 44017; EMF-Portal URL: https://www.emf-portal.org/en/article/44017

SP - 734-737

TI - Occurrence of micronuclei in exfoliated buccal mucosal cells in mobile phone users: A case-control study

UR - https://www.ijdr.in/article.asp?issn=0970-9290;year=2020;volume=31;issue=5;spage=734;epage=737;aulast=Rashmi

ER -

TY - JOUR

JA - Environ Pollut

JO - Environmental Pollution

PY - 2020

SN - 0269-7491

VL - 267

AU - Gulati S

AU - Kosik P

AU - Durdik M

AU - Skorvaga M

AU - Jakl L

AU - Markova E

AU - Belyaev I

DO - 10.1016/j.envpol.2020.115632

LA - en

N1 - FEMU ID: 43792; EMF-Portal URL: https://www.emf-portal.org/en/article/43792

SP - 115632

TI - Effects of different mobile phone UMTS signals on DNA, apoptosis and oxidative stress in human lymphocytes

ER -

TY - JOUR

IS - 6

JA - Gen Physiol Biophys

JO - General Physiology and Biophysics

PY - 2020

SN - 0231-5882

VL - 39

AU - Panagopoulos DJ

DO - 10.4149/gpb_2020036

LA - en

N1 - FEMU ID: 43746; EMF-Portal URL: https://www.emf-portal.org/en/article/43746

SP - 531-544

TI - Comparing chromosome damage induced by mobile telephony radiation and a high caffeine dose: Effect of combination and exposure duration

ER -

TY - JOUR

IS - 5

JA - Neurol India

JO - Neurology India

PY - 2020

SN - 0028-3886

VL - 68

AU - Sharma A

AU - Shrivastava S

AU - Shukla S

DO - 10.4103/0028-3886.294554

LA - en

N1 - FEMU ID: 43574; EMF-Portal URL: https://www.emf-portal.org/en/article/43574

SP - 1092-1100

TI - Exposure of Radiofrequency Electromagnetic Radiation on Biochemical and Pathological Alterations

UR - https://www.neurologyindia.com/article.asp?issn=0028-3886;year=2020;volume=68;issue=5;spage=1092;epage=1100;aulast=Sharma

ER -

TY - JOUR

IS - 8

JO - Bioelectromagnetics

PY - 2020

SN - 0197-8462

VL - 41

AU - Lerchl A

AU - Klose M

AU - Drees (née Grote) K

DO - 10.1002/bem.22301

LA - en

N1 - FEMU ID: 43440; EMF-Portal URL: https://www.emf-portal.org/en/article/43440

SP - 611-616

TI - No Increased DNA Damage Observed in the Brain, Liver, and Lung of Fetal Mice Treated With Ethylnitrosourea and Exposed to UMTS Radiofrequency Electromagnetic Fields

ER -

TY - JOUR

IS - 1

JA - Biotechnol Biotechnol Equip

JO - Biotechnology & Biotechnological Equipment

PY - 2020

SN - 1310-2818

VL - 34

AU - Bektas H

AU - Dasdag S

AU - Bektas MS

DO - 10.1080/13102818.2020.1725639

LA - en

N1 - FEMU ID: 43422; EMF-Portal URL: https://www.emf-portal.org/en/article/43422

SP - 154-162

TI - Comparison of effects of 2.4 GHz Wi-Fi and mobile phone exposure on human placenta and cord blood

UR - https://www.tandfonline.com/doi/abs/10.1080/13102818.2020.1725639?needAccess=true#aHR0cHM6Ly93d3cudGFuZGZvbmxpbmUuY29tL2RvaS9wZGYvMTAuMTA4MC8xMzEwMjgxOC4yMDIwLjE3MjU2Mzk/bmVlZEFjY2Vzcz10cnVlQEBAMA==

ER -

TY - JOUR

JA - Sci Rep

JO - Scientific Reports

PY - 2020

SN - 2045-2322

VL - 10

AU - Choi J

AU - Min K

AU - Jeon S

AU - Kim N

AU - Pack JK

AU - Song K

DO - 10.1038/s41598-020-65732-4

LA - en

N1 - FEMU ID: 42704; EMF-Portal URL: https://www.emf-portal.org/en/article/42704

SP - 9238

TI - Continuous Exposure to 1.7 GHz LTE Electromagnetic Fields Increases Intracellular Reactive Oxygen Species to Decrease Human Cell Proliferation and Induce Senescence

UR - https://www.nature.com/articles/s41598-020-65732-4.pdf

ER -

TY - JOUR

IS - 5

JA - J Anim Physiol Anim Nutr (Berl)

JO - Journal of Animal Physiology and Animal Nutrition

PY - 2020

SN - 0931-2439

VL - 104

AU - Azimzadeh M

AU - Jelodar G

DO - 10.1111/jpn.13360

LA - en

N1 - FEMU ID: 42238; EMF-Portal URL: https://www.emf-portal.org/en/article/42238

SP - 1568-1574

TI - Trace elements homeostasis in brain exposed to 900 MHz RFW emitted from a BTS-antenna model and the protective role of vitamin E

ER -

TY - JOUR

IS - 4

JO - Genes

PY - 2020

SN - 2073-4425

VL - 11

AU - Schuermann D

AU - Ziemann C

AU - Barekati Z

AU - Capstick M

AU - Oertel A

AU - Focke F

AU - Murbach M

AU - Kuster N

AU - Dasenbrock C

AU - Schär P

DO - 10.3390/genes11040347

LA - en

N1 - FEMU ID: 42121; EMF-Portal URL: https://www.emf-portal.org/en/article/42121

SP - E347

TI - Assessment of Genotoxicity in Human Cells Exposed to Modulated Electromagnetic Fields of Wireless Communication Devices

UR - https://www.mdpi.com/2073-4425/11/4/347/pdf

ER -

TY - JOUR

JA - J Chem Neuroanat

JO - Journal of Chemical Neuroanatomy

PY - 2020

SN - 0891-0618

VL - 106

AU - Sharma S

AU - Shukla S

DO - 10.1016/j.jchemneu.2020.101784

LA - en

N1 - FEMU ID: 42061; EMF-Portal URL: https://www.emf-portal.org/en/article/42061

SP - 101784

TI - Effect of electromagnetic radiation on redox status, acetylcholine esterase activity and cellular damage contributing to the diminution of the brain working memory in rats

ER -

TY - JOUR

IS - 3

JO - Bioelectromagnetics

PY - 2020

SN - 0197-8462

VL - 41

AU - Yang H

AU - Zhang Y

AU - Wang Z

AU - Zhong S

AU - Hu G

AU - Zuo W

DO - 10.1002/bem.22255

LA - en

N1 - FEMU ID: 41784; EMF-Portal URL: https://www.emf-portal.org/en/article/41784

SP - 219-229

TI - The Effects of Mobile Phone Radiofrequency Radiation on Cochlear Stria Marginal Cells in Sprague-Dawley Rats

ER -

TY - JOUR

JA - Ecotoxicol Environ Saf

JO - Ecotoxicology and Environmental Safety

PY - 2020

SN - 0147-6513

VL - 188

AU - Kumar A

AU - Kaur S

AU - Chandel S

AU - Singh HP

AU - Batish DR

AU - Kohli RK

DO - 10.1016/j.ecoenv.2019.109786

LA - en

N1 - FEMU ID: 40396; EMF-Portal URL: https://www.emf-portal.org/en/article/40396

SP - 109786

TI - Comparative cyto- and genotoxicity of 900 MHz and 1800 MHz electromagnetic field radiations in root meristems of Allium cepa

ER -

TY - JOUR

IS - 2

JA - Environ Mol Mutagen

JO - Environmental and Molecular Mutagenesis

PY - 2020

SN - 0893-6692

VL - 61

AU - Smith-Roe SL

AU - Wyde ME

AU - Stout MD

AU - Winters JW

AU - Hobbs CA

AU - Shepard KG

AU - Green AS

AU - Kissling GE

AU - Shockley KR

AU - Tice RR

AU - Bucher JR

AU - Witt KL

DO - 10.1002/em.22343

LA - en

N1 - FEMU ID: 39949; EMF-Portal URL: https://www.emf-portal.org/en/article/39949

SP - 276-290

TI - Evaluation of the genotoxicity of cell phone radiofrequency radiation in male and female rats and mice following subchronic exposure

ER -

TY - JOUR

IS - 1

JA - IEEE J Electromagn RF Microw Med Biol

JO - IEEE Journal of Electromagnetics, RF and Microwaves in Medicine and Biology

PY - 2020

VL - 4

AU - Romeo S

AU - Sannino A

AU - Zeni O

AU - Angrisani L

AU - Massa R

AU - Scarfi MR

DO - 10.1109/JERM.2019.2918023

LA - en

N1 - FEMU ID: 38441; EMF-Portal URL: https://www.emf-portal.org/en/article/38441

SP - 17-23

TI - Effects of Radiofrequency Exposure and Co-Exposure on Human Lymphocytes: the Influence of Signal Modulation and Bandwidth

ER -

TY - JOUR

IS - 1

JA - Egypt J Rad Sci Applic

JO - Egyptian Journal of Radiation Sciences and Applications

PY - 2019

SN - 1110-0303

VL - 32

AU - Shedid SM

AU - El-Tawill GA

AU - Algeda FR

AU - El-Fatih NN

AU - Eltahawy NA

DO - 10.21608/EJRSA.2019.7814.1063

LA - en

N1 - FEMU ID: 46380; EMF-Portal URL: https://www.emf-portal.org/en/article/46380

SP - 51-60

TI - The Impact of 950MHz Electromagnetic Radiation on the Brain and Liver of Rats and the Role of Garlic Treatment

UR - https://ejrsa.journals.ekb.eg/article_31561_d52dbf6882859df535be3cb2874ce239.pdf

ER -

TY - JOUR

IS - 4

JA - Histol Histopathol

JO - Histology and Histopathology

PY - 2019

SN - 0213-3911

VL - 34

AU - Šimaiová V

AU - Almášiová V

AU - Holovská K

AU - Kisková T

AU - Horváthová F

AU - Ševčíková Z

AU - Tóth Š

AU - Raček A

AU - Račeková E

AU - Beňová K

AU - Dvořák P

AU - Cigánková V

DO - 10.14670/HH-18-049

LA - en

N1 - FEMU ID: 45874; EMF-Portal URL: https://www.emf-portal.org/en/article/45874

SP - 391-403

TI - The effect of 2.45 GHz non-ionizing radiation on the structure and ultrastructure of the testis in juvenile rats

ER -

TY - JOUR

IS - 1

JA - Biotechnol Biotechnol Equip

JO - Biotechnology & Biotechnological Equipment

PY - 2019

SN - 1310-2818

VL - 33

AU - Alkis ME

AU - Akdag MZ

AU - Dasdag S

AU - Yegin K

AU - Akpolat V

DO - 10.1080/13102818.2019.1696702

LA - en

N1 - FEMU ID: 43430; EMF-Portal URL: https://www.emf-portal.org/en/article/43430

SP - 1733-1740

TI - Single-strand DNA breaks and oxidative changes in rat testes exposed to radiofrequency radiation emitted from cellular phones

UR - https://www.tandfonline.com/doi/abs/10.1080/13102818.2019.1696702?needAccess=true#aHR0cHM6Ly93d3cudGFuZGZvbmxpbmUuY29tL2RvaS9wZGYvMTAuMTA4MC8xMzEwMjgxOC4yMDE5LjE2OTY3MDI/bmVlZEFjY2Vzcz10cnVlQEBAMA==

ER -

TY - JOUR

JA - Sci Rep

JO - Scientific Reports

PY - 2019

SN - 2045-2322

VL - 9

AU - Houston BJ

AU - Nixon B

AU - McEwan KE

AU - Martin JH

AU - King BV

AU - Aitken RJ

AU - De Iuliis GN

DO - 10.1038/s41598-019-53983-9

LA - en

N1 - FEMU ID: 40773; EMF-Portal URL: https://www.emf-portal.org/en/article/40773

SP - 17478

TI - Whole-body exposures to radiofrequency-electromagnetic energy can cause DNA damage in mouse spermatozoa via an oxidative mechanism

UR - https://www.nature.com/articles/s41598-019-53983-9.pdf

ER -

TY - JOUR

JA - Sci Rep

JO - Scientific Reports

PY - 2019

SN - 2045-2322

VL - 9

AU - Durdik M

AU - Kosik P

AU - Markova E

AU - Somsedikova A

AU - Gajdosechova B

AU - Nikitina E

AU - Horvathova E

AU - Kozics K

AU - Davis D

AU - Belyaev I

DO - 10.1038/s41598-019-52389-x

LA - en

N1 - FEMU ID: 40421; EMF-Portal URL: https://www.emf-portal.org/en/article/40421

SP - 16182

TI - Microwaves from mobile phone induce reactive oxygen species but not DNA damage, preleukemic fusion genes and apoptosis in hematopoietic stem/progenitor cells

UR - https://www.nature.com/articles/s41598-019-52389-x.pdf

ER -

TY - JOUR

IS - 10

JA - Biomed Opt Express

JO - Biomedical Optics Express

PY - 2019

SN - 2156-7085

VL - 10

AU - Cheon H

AU - Yang HJ

AU - Choi M

AU - Son JH

DO - 10.1364/BOE.10.004931

LA - en

N1 - FEMU ID: 40027; EMF-Portal URL: https://www.emf-portal.org/en/article/40027

SP - 4931-4941

TI - Effective demethylation of melanoma cells using terahertz radiation

UR - https://www.osapublishing.org/boe/viewmedia.cfm?uri=boe-10-10-4931&seq=0

ER -

TY - JOUR

IS - 5

JA - Gen Physiol Biophys

JO - General Physiology and Biophysics

PY - 2019

SN - 0231-5882

VL - 38

AU - Panagopoulos DJ

DO - 10.4149/gpb_2019032

LA - en

N1 - FEMU ID: 39526; EMF-Portal URL: https://www.emf-portal.org/en/article/39526

SP - 445-454

TI - Chromosome damage in human cells induced by UMTS mobile telephony radiation

ER -

TY - JOUR

IS - 1

JA - J Environ Health Sci Eng

JO - Journal of Environmental Health Science & Engineering

PY - 2019

VL - 17

AU - Chandel S

AU - Kaur S

AU - Issa M

AU - Singh HP

AU - Batish DR

AU - Kohli RK

DO - 10.1007/s40201-018-00330-1

LA - en

N1 - FEMU ID: 38862; EMF-Portal URL: https://www.emf-portal.org/en/article/38862

SP - 97-104

TI - Exposure to mobile phone radiations at 2350 MHz incites cyto- and genotoxic effects in root meristems of Allium cepa

UR - https://link.springer.com/content/pdf/10.1007/s40201-018-00330-1.pdf

ER -

TY - JOUR

IS - 4

JA - J Radiat Res

JO - Journal of Radiation Research

PY - 2019

SN - 0449-3060

VL - 60

AU - Koyama S

AU - Narita E

AU - Suzuki Y

AU - Shiina T

AU - Taki M

AU - Shinohara N

AU - Miyakoshi J

DO - 10.1093/jrr/rrz017

LA - en

N1 - FEMU ID: 38589; EMF-Portal URL: https://www.emf-portal.org/en/article/38589

SP - 417-423

TI - Long-term exposure to a 40-GHz electromagnetic field does not affect genotoxicity or heat shock protein expression in HCE-T or SRA01/04 cells

UR - https://academic.oup.com/jrr/article-pdf/60/4/417/28964321/rrz017.pdf

ER -

TY - JOUR

IS - 5

JO - Protoplasma

PY - 2019

SN - 0033-183X

VL - 256

AU - Chandel S

AU - Kaur S

AU - Issa M

AU - Singh HP

AU - Batish DR

AU - Kohli RK

DO - 10.1007/s00709-019-01386-y

LA - en

N1 - FEMU ID: 38426; EMF-Portal URL: https://www.emf-portal.org/en/article/38426

SP - 1399-1407

TI - Appraisal of immediate and late effects of mobile phone radiations at 2100 MHz on mitotic activity and DNA integrity in root meristems of Allium cepa

ER -

TY - JOUR

JA - Environ Res

JO - Environmental Research

PY - 2019

SN - 0013-9351

VL - 174

AU - Jooyan N

AU - Goliaei B

AU - Bigdeli B

AU - Faraji-Dana R

AU - Zamani A

AU - Entezami M

AU - Mortazavi SMJ

DO - 10.1016/j.envres.2019.03.063

LA - en

N1 - FEMU ID: 38182; EMF-Portal URL: https://www.emf-portal.org/en/article/38182

SP - 176-187

TI - Direct and indirect effects of exposure to 900 MHz GSM radiofrequency electromagnetic fields on CHO cell line: Evidence of bystander effect by non-ionizing radiation

ER -

TY - JOUR

IS - 2

JA - IEEE Trans Nanobioscience

JO - IEEE Transactions on Nanobioscience

PY - 2019

SN - 1536-1241

VL - 18

AU - Miyakoshi J

AU - Tonomura H

AU - Koyama S

AU - Narita E

AU - Shinohara N

DO - 10.1109/TNB.2019.2905491

LA - en

N1 - FEMU ID: 37795; EMF-Portal URL: https://www.emf-portal.org/en/article/37795

SP - 257-260

TI - Effects of Exposure to 5.8 GHz Electromagnetic Field on Micronucleus Formation, DNA Strand Breaks, and Heat Shock Protein Expressions in Cells Derived From Human Eye

ER -

TY - JOUR

JA - Toxicol Appl Pharmacol

JO - Toxicology and Applied Pharmacology

PY - 2019

SN - 0041-008X

VL - 370

AU - Shahin NN

AU - El-Nabarawy NA

AU - Gouda AS

AU - Mégarbane B

DO - 10.1016/j.taap.2019.03.009

LA - en

N1 - FEMU ID: 37751; EMF-Portal URL: https://www.emf-portal.org/en/article/37751

SP - 117-130

TI - The protective role of spermine against male reproductive aberrations induced by exposure to electromagnetic field - An experimental investigation in the rat

ER -

TY - JOUR

IS - 1

JA - Electromagn Biol Med

JO - Electromagnetic Biology and Medicine

PY - 2019

SN - 1536-8386

VL - 38

AU - Alkis ME

AU - Bilgin HM

AU - Akpolat V

AU - Dasdag S

AU - Yegin K

AU - Yavas MC

AU - Akdag MZ

DO - 10.1080/15368378.2019.1567526

LA - en

N1 - FEMU ID: 37161; EMF-Portal URL: https://www.emf-portal.org/en/article/37161

SP - 32-47

TI - Effect of 900-, 1800-, and 2100-MHz radiofrequency radiation on DNA and oxidative stress in brain

ER -

TY - JOUR

IS - 2

JA - Int J Radiat Biol

JO - International Journal of Radiation Biology

PY - 2019

SN - 0955-3002

VL - 95

AU - Mahdavi SR

AU - Janati Esfahani A

AU - Khoei S

AU - Bakhshandeh M

AU - Rajabi A

DO - 10.1080/09553002.2019.1532608

LA - en

N1 - FEMU ID: 36423; EMF-Portal URL: https://www.emf-portal.org/en/article/36423

SP - 193-200

TI - Capacitive hyperthermia as an alternative to brachytherapy in DNA damages of human prostate cancer cell line (DU-145)

ER -

TY - JOUR

IS - 4

JA - Chin Med J

JO - Chinese Medical Journal

PY - 2018

SN - 0366-6999

VL - 131

AU - Ding SS

AU - Sun P

AU - Zhang Z

AU - Liu X

AU - Tian H

AU - Huo YW

AU - Wang LR

AU - Han Y

AU - Xing JP

DO - 10.4103/0366-6999.225045

LA - en

N1 - FEMU ID: 49146; EMF-Portal URL: https://www.emf-portal.org/en/article/49146

SP - 402-412

TI - Moderate Dose of Trolox Preventing the Deleterious Effects of Wi-Fi Radiation on Spermatozoa In vitro through Reduction of Oxidative Stress Damage

UR - https://journals.lww.com/cmj/Fulltext/2018/02200/Moderate_Dose_of_Trolox_Preventing_the_Deleterious.4.aspx

ER -

TY - JOUR

IS - 6

JA - Int J Environ Sci Technol

JO - International Journal of Environmental Science and Technology

PY - 2018

SN - 1735-1472

VL - 15

AU - Răcuciu M

AU - Iftode C

AU - Miclăuş S

DO - 10.1007/s13762-017-1490-0

LA - en

N1 - FEMU ID: 49003; EMF-Portal URL: https://www.emf-portal.org/en/article/49003

SP - 1233-1242

TI - Influence of 1 GHz radiation at low specific absorption rate of energy deposition on plant mitotic division process

ER -

TY - JOUR

IS - 3

JA - J Oral Maxillofac Pathol

JO - Journal of Oral and Maxillofacial Pathology: JOMFP

PY - 2018

SN - 0973-029X

VL - 22

AU - Vanishree M

AU - Manvikar V

AU - Rudraraju A

AU - Reddy KMP

AU - Kumar NHP

AU - Quadri SJM

DO - 10.4103/jomfp.JOMFP_201_18

LA - en

N1 - FEMU ID: 37117; EMF-Portal URL: https://www.emf-portal.org/en/article/37117

SP - 448

TI - Significance of micronuclei in buccal smears of mobile phone users: A comparative study

UR - http://www.jomfp.in/article.asp?issn=0973-029X;year=2018;volume=22;issue=3;spage=448;epage=448;aulast=Vanishree

ER -

TY - JOUR

JA - Front Public Health

JO - Frontiers in Public Health

PY - 2018

SN - 2296-2565

VL - 6

AU - Houston BJ

AU - Nixon B

AU - King BV

AU - Aitken RJ

AU - De Iuliis GN

DO - 10.3389/fpubh.2018.00270

LA - en

N1 - FEMU ID: 36249; EMF-Portal URL: https://www.emf-portal.org/en/article/36249

SP - 270

TI - Probing the Origins of 1,800 MHz Radio Frequency Electromagnetic Radiation Induced Damage in Mouse Immortalized Germ Cells and Spermatozoa in vitro

UR - https://www.frontiersin.org/articles/10.3389/fpubh.2018.00270/pdf

ER -

TY - JOUR

IS - 9

JA - Bratisl Lek Listy

JO - Bratislavské Lekárske Listy

PY - 2018

SN - 0006-9248

VL - 119

AU - Eker ED

AU - Arslan B

AU - Yildirim M

AU - Akar A

AU - Aras N

DO - 10.4149/BLL_2018_106

LA - en

N1 - FEMU ID: 36061; EMF-Portal URL: https://www.emf-portal.org/en/article/36061

SP - 588-592

TI - The effect of exposure to 1800 MHz radiofrequency radiation on epidermal growth factor, caspase-3, Hsp27 and p38MAPK gene expressions in the rat eye

UR - http://www.elis.sk/download_file.php?product_id=5838&session_id=17juffpe9loofcmnusa9dq6q13

ER -

TY - JOUR

JA - Sci Rep

JO - Scientific Reports

PY - 2018

SN - 2045-2322

VL - 8

AU - Falone S

AU - Sannino A

AU - Romeo S

AU - Zeni O

AU - Santini SJ

AU - Rispoli R

AU - Amicarelli F

AU - Scarfì MR

DO - 10.1038/s41598-018-31636-7

LA - en

N1 - FEMU ID: 35984; EMF-Portal URL: https://www.emf-portal.org/en/article/35984

SP - 13234

TI - Protective effect of 1950 MHz electromagnetic field in human neuroblastoma cells challenged with menadione

UR - https://www.nature.com/articles/s41598-018-31636-7.pdf

ER -

TY - JOUR

IS - 5

JA - Syst Biol Reprod Med

JO - Systems Biology in Reproductive Medicine

PY - 2018

SN - 1939-6368

VL - 64

AU - Shi X

AU - Chan CPS

AU - Waters T

AU - Chi L

AU - Chan DYL

AU - Li TC

DO - 10.1080/19396368.2018.1491074

LA - en

N1 - FEMU ID: 35596; EMF-Portal URL: https://www.emf-portal.org/en/article/35596

SP - 358-367

TI - Lifestyle and demographic factors associated with human semen quality and sperm function

ER -

TY - JOUR

IS - 7

JA - Int J Mol Sci

JO - International Journal of Molecular Sciences

PY - 2018

SN - 1422-0067

VL - 19

AU - Jeong YJ

AU - Son Y

AU - Han NK

AU - Choi HD

AU - Pack JK

AU - Kim N

AU - Lee YS

AU - Lee HJ

DO - 10.3390/ijms19072103

LA - en

N1 - FEMU ID: 35546; EMF-Portal URL: https://www.emf-portal.org/en/article/35546

TI - Impact of Long-Term RF-EMF on Oxidative Stress and Neuroinflammation in Aging Brains of C57BL/6 Mice

UR - http://www.mdpi.com/1422-0067/19/7/2103/pdf

ER -

TY - JOUR

IS - 1

JA - Cell Physiol Biochem

JO - Cellular Physiology and Biochemistry

PY - 2018

SN - 1015-8987

VL - 48

AU - Li R

AU - Ma M

AU - Li L

AU - Zhao L

AU - Zhang T

AU - Gao X

AU - Zhang D

AU - Zhu Y

AU - Peng Q

AU - Luo X

AU - Wang M

DO - 10.1159/000491660

LA - en

N1 - FEMU ID: 35470; EMF-Portal URL: https://www.emf-portal.org/en/article/35470

SP - 29-41

TI - The Protective Effect of Autophagy on DNA Damage in Mouse Spermatocyte-Derived Cells Exposed to 1800 MHz Radiofrequency Electromagnetic Fields

UR - https://www.karger.com/Article/Pdf/491660

ER -

TY - JOUR

IS - 1

JA - Health Phys

JO - Health Physics

PY - 2018

SN - 0017-9078

VL - 115

AU - Franchini V

AU - Regalbuto E

AU - De Amicis A

AU - De Sanctis S

AU - Di Cristofaro S

AU - Coluzzi E

AU - Marinaccio J

AU - Sgura A

AU - Ceccuzzi S

AU - Doria A

AU - Gallerano GP

AU - Giovenale E

AU - Ravera GL

AU - Bei R

AU - Benvenuto M

AU - Modesti A

AU - Masuelli L

AU - Lista F

DO - 10.1097/HP.0000000000000871

LA - en

N1 - FEMU ID: 35185; EMF-Portal URL: https://www.emf-portal.org/en/article/35185

SP - 126-139

TI - Genotoxic Effects in Human Fibroblasts Exposed to Microwave Radiation

ER -

TY - JOUR

IS - 2

JA - Electromagn Biol Med

JO - Electromagnetic Biology and Medicine

PY - 2018

SN - 1536-8386

VL - 37

AU - Akdag M

AU - Dasdag S

AU - Canturk F

AU - Akdag MZ

DO - 10.1080/15368378.2018.1463246

LA - en

N1 - FEMU ID: 34966; EMF-Portal URL: https://www.emf-portal.org/en/article/34966

SP - 66-75

TI - Exposure to non-ionizing electromagnetic fields emitted from mobile phones induced DNA damage in human ear canal hair follicle cells

ER -

TY - JOUR

IS - 4

JO - PLoS One

PY - 2018

SN - 1932-6203

VL - 13

AU - Al-Serori H

AU - Ferk F

AU - Kundi M

AU - Bileck A

AU - Gerner C

AU - Mišík M

AU - Nersesyan A

AU - Waldherr M

AU - Murbach M

AU - Lah TT

AU - Herold-Mende C

AU - Collins AR

AU - Knasmüller S

DO - 10.1371/journal.pone.0193677

LA - en

N1 - FEMU ID: 34934; EMF-Portal URL: https://www.emf-portal.org/en/article/34934

SP - e0193677

TI - Mobile phone specific electromagnetic fields induce transient DNA damage and nucleotide excision repair in serum-deprived human glioblastoma cells

UR - http://journals.plos.org/plosone/article/file?id=10.1371/journal.pone.0193677&type=printable

ER -

TY - JOUR

IS - 6

JA - Environ Mol Mutagen

JO - Environmental and Molecular Mutagenesis

PY - 2018

SN - 0893-6692

VL - 59

AU - Franchini V

AU - De Sanctis S

AU - Marinaccio J

AU - De Amicis A

AU - Coluzzi E

AU - Di Cristofaro S

AU - Lista F

AU - Regalbuto E

AU - Doria A

AU - Giovenale E

AU - Gallerano GP

AU - Bei R

AU - Benvenuto M

AU - Masuelli L

AU - Udroiu I

AU - Sgura A

DO - 10.1002/em.22192

LA - en

N1 - FEMU ID: 34868; EMF-Portal URL: https://www.emf-portal.org/en/article/34868

SP - 476-487

TI - Study of the effects of 0.15 terahertz radiation on genome integrity of adult fibroblasts

UR - https://onlinelibrary.wiley.com/doi/epdf/10.1002/em.22192

ER -

TY - JOUR

IS - 5

JA - Toxicol Ind Health

JO - Toxicology and Industrial Health

PY - 2018

SN - 0748-2337

VL - 34

AU - Pandey N

AU - Giri S

DO - 10.1177/0748233718758092

LA - en

N1 - FEMU ID: 34795; EMF-Portal URL: https://www.emf-portal.org/en/article/34795

SP - 315-327

TI - Melatonin attenuates radiofrequency radiation (900 MHz)-induced oxidative stress, DNA damage and cell cycle arrest in germ cells of male Swiss albino mice

ER -

TY - JOUR

IS - 10

JA - Int J Radiat Biol

JO - International Journal of Radiation Biology

PY - 2018

SN - 0955-3002

VL - 94

AU - Herrala M

AU - Mustafa E

AU - Naarala J

AU - Juutilainen J

DO - 10.1080/09553002.2018.1450534

LA - en

N1 - FEMU ID: 34733; EMF-Portal URL: https://www.emf-portal.org/en/article/34733

SP - 883-889

TI - Assessment of genotoxicity and genomic instability in rat primary astrocytes exposed to 872 MHz radiofrequency radiation and chemicals

ER -

TY - JOUR

IS - 3

JA - Int J Radiat Biol

JO - International Journal of Radiation Biology

PY - 2018

SN - 0955-3002

VL - 94

AU - Su L

AU - Yimaer A

AU - Xu Z

AU - Chen G

DO - 10.1080/09553002.2018.1432913

LA - en

N1 - FEMU ID: 34481; EMF-Portal URL: https://www.emf-portal.org/en/article/34481

SP - 295-305

TI - Effects of 1800 MHz RF-EMF exposure on DNA damage and cellular functions in primary cultured neurogenic cells

ER -

TY - JOUR

IS - 9

JA - Sains Malays

JO - Sains Malaysiana

PY - 2017

SN - 0126-6039

VL - 46

AU - Jamaludin N

AU - Razak SSA

AU - Jaffar FHF

AU - Osman K

AU - Ibrahim SF

DO - 10.17576/jsm-2017-4609-31

LA - en

N1 - FEMU ID: 46571; EMF-Portal URL: https://www.emf-portal.org/en/article/46571

SP - 1597-1602

TI - The Effect of Smartphone's Radiation Frequency and Exposure Duration on NADPH Oxidase 5 (NOX5) Level in Sperm Parameters

UR - http://www.ukm.my/jsm/pdf_files/SM-PDF-46-9-2017/31%20Norazurashima.pdf

ER -

TY - JOUR

IS - 1

JA - Int J Radiat Res

JO - International Journal of Radiation Research

PY - 2017

SN - 2322-3243

VL - 15

AU - Parsanezhad ME

AU - Mortazavi SMJ

AU - Doohandeh T

AU - Jahromi BN

AU - Mozdarani H

AU - Zarei A

AU - Davari M

AU - Amjadi S

AU - Soleimani A

AU - Haghani M

DO - 10.18869/acadpub.ijrr.15.1.63

LA - en

N1 - FEMU ID: 46205; EMF-Portal URL: https://www.emf-portal.org/en/article/46205

SP - 63-70

TI - Exposure to radiofrequency radiation emitted from mobile phone jammers adversely affects the quality of human sperm

UR - http://ijrr.com/article-1-1887-en.pdf

ER -

TY - JOUR

IS - 4

JA - J Apic Res

JO - Journal of Apicultural Research

PY - 2017

VL - 56

AU - Vilic M

AU - Gajger IT

AU - Tucak P

AU - Stambuk A

AU - Srut M

AU - Klobucar G

AU - Malaric K

AU - Zajaa IZ

AU - Pavelic A

AU - Manger M

AU - Tkalec M

DO - 10.1080/00218839.2017.1329798

LA - en

N1 - FEMU ID: 46075; EMF-Portal URL: https://www.emf-portal.org/en/article/46075

SP - 430-438

TI - Effects of short-term exposure to mobile phone radiofrequency (900 MHz) on the oxidative response and genotoxicity in honey bee larvae

ER -

TY - JOUR

IS - 1

JA - Pol J Environ Stud

JO - Polish Journal of Environmental Studies

PY - 2017

SN - 1230-1485

VL - 26

AU - Sieron-Stoltny K

AU - Pasek J

AU - Cieslar G

AU - Sieron A

DO - 10.15244/pjoes/64747

LA - en

N1 - FEMU ID: 46062; EMF-Portal URL: https://www.emf-portal.org/en/article/46062

SP - 279-285

TI - Influence of Electromagnetic Fields on Prooxidant/Antioxidant Balance in Rat Liver

UR - http://www.pjoes.com/pdf-64746-23951?filename=Increasing%20Area%20of.pdf

ER -

TY - JOUR

JA - Data Brief

JO - Data in Brief

PY - 2017

SN - 2352-3409

VL - 15

AU - de Oliveira FM

AU - Carmona AM

AU - Ladeira C

DO - 10.1016/j.dib.2017.09.048

LA - en

N1 - FEMU ID: 34141; EMF-Portal URL: https://www.emf-portal.org/en/article/34141

SP - 344-347

TI - Genotoxicity assessment data for exfoliated buccal cells exposed to mobile phone radiation

UR - https://www.sciencedirect.com/science/article/pii/S2352340917304791/pdfft?md5=cf92857ee630b0a186210e76b366ce6d&pid=1-s2.0-S2352340917304791-main.pdf

ER -

TY - JOUR

JA - Biomed Res Int

JO - BioMed Research International

PY - 2017

VL - 2017

AU - Ibitayo AO

AU - Afolabi OB

AU - Akinyemi AJ

AU - Ojiezeh TI

AU - Adekoya KO

AU - Ojewunmi OO

DO - 10.1155/2017/8653286

LA - en

N1 - FEMU ID: 33141; EMF-Portal URL: https://www.emf-portal.org/en/article/33141

SP - 8653286

TI - RAPD Profiling, DNA Fragmentation, and Histomorphometric Examination in Brains of Wistar Rats Exposed to Indoor 2.5 Ghz Wi-Fi Devices Radiation

UR - http://downloads.hindawi.com/journals/bmri/2017/8653286.pdf

ER -

TY - JOUR

IS - 7

JA - J Clin of Diagn Res

JO - Journal of Clinical and Diagnostic Research

PY - 2017

SN - 0973-709X

VL - 11

AU - D'Silva MH

AU - Swer RT

AU - Anbalagan J

AU - Rajesh B

DO - 10.7860/JCDR/2017/26360.10275

LA - en

N1 - FEMU ID: 33100; EMF-Portal URL: https://www.emf-portal.org/en/article/33100

SP - AC05-AC09

TI - Effect of Radiofrequency Radiation Emitted from 2G and 3G Cell Phone on Developing Liver of Chick Embryo - A Comparative Study

UR - https://www.ncbi.nlm.nih.gov/pmc/articles/PMC5583901/pdf/jcdr-11-AC05.pdf

ER -

TY - JOUR

JA - Mutat Res Genet Toxicol Environ Mutagen

JO - Mutation Research - Genetic Toxicology and Environmental Mutagenesis

PY - 2017

VL - 822

AU - de Oliveira FM

AU - Carmona AM

AU - Ladeira C

DO - 10.1016/j.mrgentox.2017.08.001

LA - en

N1 - FEMU ID: 32942; EMF-Portal URL: https://www.emf-portal.org/en/article/32942

SP - 41-46

TI - Is mobile phone radiation genotoxic? An analysis of micronucleus frequency in exfoliated buccal cells

ER -

TY - JOUR

IS - 3

JA - Electromagn Biol Med

JO - Electromagnetic Biology and Medicine

PY - 2017

SN - 1536-8386

VL - 36

AU - Zothansiama

AU - Zosangzuali M

AU - Lalramdinpuii M

AU - Jagetia GC

DO - 10.1080/15368378.2017.1350584

LA - en

N1 - FEMU ID: 32665; EMF-Portal URL: https://www.emf-portal.org/en/article/32665

SP - 295-305

TI - Impact of radiofrequency radiation on DNA damage and antioxidants in peripheral blood lymphocytes of humans residing in the vicinity of mobile phone base stations

ER -

TY - JOUR

IS - 13

JA - Ann Transl Med

JO - Annals of Translational Medicine

PY - 2017

SN - 2305-5839

VL - 5

AU - Danese E

AU - Lippi G

AU - Buonocore R

AU - Benati M

AU - Bovo C

AU - Bonaguri C

AU - Salvagno GL

AU - Brocco G

AU - Roggenbuck D

AU - Montagnana M

DO - 10.21037/atm.2017.04.35

LA - en

N1 - FEMU ID: 32623; EMF-Portal URL: https://www.emf-portal.org/en/article/32623

SP - 272

TI - Mobile phone radiofrequency exposure has no effect on DNA double strand breaks (DSB) in human lymphocytes

UR - https://www.ncbi.nlm.nih.gov/pmc/articles/PMC5515807/pdf/atm-05-13-272.pdf

ER -

TY - JOUR

JA - Mutat Res Genet Toxicol Environ Mutagen

JO - Mutation Research - Genetic Toxicology and Environmental Mutagenesis

PY - 2017

VL - 820

AU - He Q

AU - Zong L

AU - Sun Y

AU - Vijayalaxmi

AU - Prihoda TJ

AU - Tong J

AU - Cao Y

DO - 10.1016/j.mrgentox.2017.05.007

LA - en

N1 - FEMU ID: 32408; EMF-Portal URL: https://www.emf-portal.org/en/article/32408

SP - 19-25

TI - Adaptive response in mouse bone marrow stromal cells exposed to 900MHz radiofrequency fields: Impact of poly (ADP-ribose) polymerase (PARP)

ER -

TY - JOUR

IS - 2

JA - Arh Hig Rada Toksikol

JO - Arhiv za Higijenu Rada i Toksikologiju (Archives of Industrial Hygiene and Toxicology)

PY - 2017

SN - 0004-1254

VL - 68

AU - Bourdineaud JP

AU - Šrut M

AU - Štambuk A

AU - Tkalec M

AU - Brèthes D

AU - Malarić K

AU - Klobučar GIV

DO - 10.1515/aiht-2017-68-2928

LA - en

N1 - FEMU ID: 32367; EMF-Portal URL: https://www.emf-portal.org/en/article/32367

SP - 142-152

TI - Electromagnetic fields at a mobile phone frequency (900 MHz) trigger the onset of general stress response along with DNA modifications in Eisenia fetida earthworms

UR - https://sciendo.com/article/10.1515/aiht-2017-68-2928

ER -

TY - JOUR

IS - 6

JO - Bioelectromagnetics

PY - 2017

SN - 0197-8462

VL - 38

AU - Suzuki S

AU - Okutsu M

AU - Suganuma R

AU - Komiya H

AU - Nakatani-Enomoto S

AU - Kobayashi S

AU - Ugawa Y

AU - Tateno H

AU - Fujimori K

DO - 10.1002/bem.22063

LA - en

N1 - FEMU ID: 32274; EMF-Portal URL: https://www.emf-portal.org/en/article/32274

SP - 466-473

TI - Influence of radiofrequency-electromagnetic waves from 3rd-generation cellular phones on fertilization and embryo development in mice

ER -

TY - JOUR

IS - 4

JA - Saudi J Biol Sci

JO - Saudi Journal of Biological Sciences

PY - 2017

SN - 1319-562X

VL - 24

AU - Qureshi ST

AU - Memon SA

AU - Abassi AR

AU - Sial MA

AU - Bughio FA

DO - 10.1016/j.sjbs.2016.02.011

LA - en

N1 - FEMU ID: 31864; EMF-Portal URL: https://www.emf-portal.org/en/article/31864

SP - 883-891

TI - Radiofrequency radiations induced genotoxic and carcinogenic effects on chickpea (Cicer arietinum L.) root tip cells

UR - https://www.sciencedirect.com/science/article/pii/S1319562X16000589/pdfft?md5=275ab70a0cf42609a2a27cd618810be3&pid=1-s2.0-S1319562X16000589-main.pdf

ER -

TY - JOUR

JO - Mutation Research - Fundamental and Molecular Mechanism of Mutagenesis

PY - 2017

SN - 0027-5107

VL - 797-799

AU - Sun Y

AU - Zong L

AU - Gao Z

AU - Zhu S

AU - Tong J

AU - Cao Y

DO - 10.1016/j.mrfmmm.2017.03.001

LA - en

N1 - FEMU ID: 31583; EMF-Portal URL: https://www.emf-portal.org/en/article/31583

SP - 7-14

TI - Mitochondrial DNA damage and oxidative damage in HL-60 cells exposed to 900MHz radiofrequency fields

ER -

TY - JOUR

JA - Toxicol In Vitro

JO - Toxicology in Vitro

PY - 2017

SN - 0887-2333

VL - 40

AU - Al-Serori H

AU - Kundi M

AU - Ferk F

AU - Mišík M

AU - Nersesyan A

AU - Murbach M

AU - Lah TT

AU - Knasmüller S

DO - 10.1016/j.tiv.2017.01.012

LA - en

N1 - FEMU ID: 31101; EMF-Portal URL: https://www.emf-portal.org/en/article/31101

SP - 264-271

TI - Evaluation of the potential of mobile phone specific electromagnetic fields (UMTS) to produce micronuclei in human glioblastoma cell lines

ER -

TY - JOUR

IS - 4

JO - Bioelectromagnetics

PY - 2017

SN - 0197-8462

VL - 38

AU - Sannino A

AU - Zeni O

AU - Romeo S

AU - Massa R

AU - Scarfi MR

DO - 10.1002/bem.22034

LA - en

N1 - FEMU ID: 30897; EMF-Portal URL: https://www.emf-portal.org/en/article/30897

SP - 245-254

TI - Adverse and beneficial effects in Chinese hamster lung fibroblast cells following radiofrequency exposure

ER -

TY - JOUR

IS - 3

JO - Bioelectromagnetics

PY - 2017

SN - 0197-8462

VL - 38

AU - Su L

AU - Wei X

AU - Xu Z

AU - Chen G

DO - 10.1002/bem.22032

LA - en

N1 - FEMU ID: 30787; EMF-Portal URL: https://www.emf-portal.org/en/article/30787

SP - 175-185

TI - RF-EMF exposure at 1800 MHz did not elicit DNA damage or abnormal cellular behaviors in different neurogenic cells

ER -

TY - JOUR

IS - 4

JA - Toxicol Ind Health

JO - Toxicology and Industrial Health

PY - 2017

SN - 0748-2337

VL - 33

AU - Pandey N

AU - Giri S

AU - Das S

AU - Upadhaya P

DO - 10.1177/0748233716671206

LA - en

N1 - FEMU ID: 30481; EMF-Portal URL: https://www.emf-portal.org/en/article/30481

SP - 373-384

TI - Radiofrequency radiation (900 MHz)-induced DNA damage and cell cycle arrest in testicular germ cells in swiss albino mice

ER -

TY - JOUR

IS - 12

JO - Environmental Engineering and Management Journal

PY - 2016

VL - 15

AU - Răcuciu M

AU - Iftode C

AU - Miclăuş S

DO - 10.30638/eemj.2016.282

LA - en

N1 - FEMU ID: 49001; EMF-Portal URL: https://www.emf-portal.org/en/article/49001

SP - 2561-2568

TI - Athermal Microwave Radiation Affects the Genetic of Vegetal Embryos

ER -

TY - JOUR

IS - 8

JA - Int J Environ Res Public Health

JO - International Journal of Environmental Research and Public Health

PY - 2016

SN - 1660-4601

VL - 13

AU - Koyama S

AU - Narita E

AU - Shimizu Y

AU - Suzuki Y

AU - Shiina T

AU - Taki M

AU - Shinohara N

AU - Miyakoshi J

DO - 10.3390/ijerph13080802

LA - en

N1 - FEMU ID: 32667; EMF-Portal URL: https://www.emf-portal.org/en/article/32667

SP - E802

TI - Effects of Long-Term Exposure to 60 GHz Millimeter-Wavelength Radiation on the Genotoxicity and Heat Shock Protein (Hsp) Expression of Cells Derived from Human Eye

UR - https://www.ncbi.nlm.nih.gov/pmc/articles/PMC4997488/pdf/ijerph-13-00802.pdf

ER -

TY - JOUR

IS - 7

JA - J Stroke Cerebrovasc Dis

JO - Journal of Stroke and Cerebrovascular Diseases

PY - 2016

SN - 1052-3057

VL - 25

AU - Fan Y

AU - Zhang C

AU - Li T

AU - Peng W

AU - Yin J

AU - Li X

AU - Kong Y

AU - Lan C

AU - Wang R

AU - Hu Z

DO - 10.1016/j.jstrokecerebrovasdis.2016.03.033

LA - en

N1 - FEMU ID: 31826; EMF-Portal URL: https://www.emf-portal.org/en/article/31826

SP - 1813-1822

TI - A New Approach of Short Wave Protection against Middle Cerebral Artery Occlusion/Reperfusion Injury via Attenuation of Golgi Apparatus Stress by Inhibition of Downregulation of Secretory Pathway Ca(2+)-ATPase Isoform 1 in Rats

ER -

TY - JOUR

IS - 12

JA - Biomed Environ Sci

JO - Biomedical and Environmental Sciences

PY - 2016

SN - 0895-3988

VL - 29

AU - Deshmukh PS

AU - Megha K

AU - Nasare N

AU - Banerjee BD

AU - Ahmed RS

AU - Abegaonkar MP

AU - Tripathi AK

AU - Mediratta PK

DO - 10.3967/bes2016.115

LA - en

N1 - FEMU ID: 30925; EMF-Portal URL: https://www.emf-portal.org/en/article/30925

SP - 858-867

TI - Effect of Low Level Subchronic Microwave Radiation on Rat Brain

UR - https://www.sciencedirect.com/science/article/pii/S089539881730003X/pdf?md5=0d903e08b2bdd4758787ca791d1624bf&pid=1-s2.0-S089539881730003X-main.pdf

ER -

TY - JOUR

JA - Sci Rep

JO - Scientific Reports

PY - 2016

SN - 2045-2322

VL - 6

AU - Sun C

AU - Wei X

AU - Fei Y

AU - Su L

AU - Zhao X

AU - Chen G

AU - Xu Z

DO - 10.1038/srep37423

LA - en

N1 - FEMU ID: 30619; EMF-Portal URL: https://www.emf-portal.org/en/article/30619

SP - 37423

TI - Mobile phone signal exposure triggers a hormesis-like effect in Atm+/+ and Atm-/- mouse embryonic fibroblasts

UR - http://www.nature.com/articles/srep37423.pdf

ER -

TY - JOUR

IS - 5

JA - Radiat Res

JO - Radiation Research

PY - 2016

SN - 0033-7587

VL - 186

AU - Gläser K

AU - Rohland M

AU - Kleine-Ostmann T

AU - Schrader T

AU - Stopper H

AU - Hintzsche H

DO - 10.1667/RR14405.1

LA - en

N1 - FEMU ID: 30442; EMF-Portal URL: https://www.emf-portal.org/en/article/30442

SP - 455-465

TI - Effect of Radiofrequency Radiation on Human Hematopoietic Stem Cells

ER -

TY - JOUR

IS - 9

JO - PLoS One

PY - 2016

SN - 1932-6203

VL - 11

AU - Xing F

AU - Zhan Q

AU - He Y

AU - Cui J

AU - He S

AU - Wang G

DO - 10.1371/journal.pone.0163935

LA - en

N1 - FEMU ID: 30422; EMF-Portal URL: https://www.emf-portal.org/en/article/30422

SP - e0163935-

TI - 1800 MHz microwave induces p53 and p53-mediated caspase-3 activation leading to cell apoptosis in vitro

UR - https://journals.plos.org/plosone/article/file?id=10.1371/journal.pone.0163935&type=printable

ER -

TY - JOUR

IS - 40

JO - Oncotarget

PY - 2016

SN - 1949-2553

VL - 7

AU - López-Furelos A

AU - Leiro-Vidal JM

AU - Salas-Sánchez AÁ

AU - Ares-Pena FJ

AU - López-Martín ME

DO - 10.18632/oncotarget.11753

LA - en

N1 - FEMU ID: 30264; EMF-Portal URL: https://www.emf-portal.org/en/article/30264

SP - 64674-

TI - Evidence of cellular stress and caspase-3 resulting from a combined two-frequency signal in the cerebrum and cerebellum of Sprague-Dawley rats

UR - https://www.oncotarget.com/article/11753/pdf/

ER -

TY - JOUR

IS - 8

JA - Int J Environ Res Public Health

JO - International Journal of Environmental Research and Public Health

PY - 2016

SN - 1660-4601

VL - 13

AU - Koyama S

AU - Narita E

AU - Shimizu Y

AU - Shiina T

AU - Taki M

AU - Shinohara N

AU - Miyakoshi J

DO - 10.3390/ijerph13080793

LA - en

N1 - FEMU ID: 30171; EMF-Portal URL: https://www.emf-portal.org/en/article/30171

SP - E793-

TI - Twenty four-hour exposure to a 0.12 THz electromagnetic field does not affect the genotoxicity, morphological changes, or expression of heat shock protein in HCE-T cells

UR - http://www.mdpi.com/1660-4601/13/8/793/pdf

ER -

TY - JOUR

JA - J Chem Neuroanat

JO - Journal of Chemical Neuroanatomy

PY - 2016

SN - 0891-0618

VL - 78

AU - Hussein S

AU - El-Saba AA

AU - Galal MK

DO - 10.1016/j.jchemneu.2016.07.009

LA - en

N1 - FEMU ID: 30004; EMF-Portal URL: https://www.emf-portal.org/en/article/30004

SP - 10-19

TI - Biochemical and histological studies on adverse effects of mobile phone radiation on rat's brain

ER -

TY - JOUR

IS - 6

JA - J Radiat Res

JO - Journal of Radiation Research

PY - 2016

SN - 0449-3060

VL - 57

AU - Lee JS

AU - Kim JY

AU - Kim HJ

AU - Kim JC

AU - Lee JS

AU - Kim N

AU - Park MJ

DO - 10.1093/jrr/rrw040

LA - en

N1 - FEMU ID: 29719; EMF-Portal URL: https://www.emf-portal.org/en/article/29719

SP - 620-626

TI - Effects of combined radiofrequency field exposure on amyloid-beta-induced cytotoxicity in HT22 mouse hippocampal neurones

UR - https://academic.oup.com/jrr/article/57/6/620/2605885

ER -

TY - JOUR

IS - 9-10

JA - J Toxicol Environ Health A

JO - Journal of Toxicology and Environmental Health, Part A

PY - 2016

SN - 0098-4108

VL - 79

AU - Ji Y

AU - He Q

AU - Sun Y

AU - Tong J

AU - Cao Y

DO - 10.1080/15287394.2016.1176618

LA - en

N1 - FEMU ID: 29624; EMF-Portal URL: https://www.emf-portal.org/en/article/29624

SP - 419-426

TI - Adaptive response in mouse bone-marrow stromal cells exposed to 900-MHz radiofrequency fields: Gamma-radiation-induced DNA strand breaks and repair

ER -

TY - JOUR

IS - 2

JA - Cell Biochem Biophys

JO - Cell Biochemistry and Biophysics

PY - 2016

SN - 1085-9195

VL - 74

AU - Kayhan H

AU - Esmekaya MA

AU - Saglam AS

AU - Tuysuz MZ

AU - Canseven AG

AU - Yagci AM

AU - Seyhan N

DO - 10.1007/s12013-016-0734-9

LA - en

N1 - FEMU ID: 29618; EMF-Portal URL: https://www.emf-portal.org/en/article/29618

SP - 99-107

TI - Does MW Radiation Affect Gene Expression, Apoptotic Level, and Cell Cycle Progression of Human SH-SY5Y Neuroblastoma Cells?

ER -

TY - JOUR

JA - Biomed Res Int

JO - BioMed Research International

PY - 2016

AU - He Q

AU - Sun Y

AU - Zong L

AU - Tong J

AU - Cao Y

DO - 10.1155/2016/4918691

LA - en

N1 - FEMU ID: 29497; EMF-Portal URL: https://www.emf-portal.org/en/article/29497

SP - 4918691-

TI - Induction of poly(ADP-ribose) polymerase in mouse bone marrow stromal cells exposed to 900 MHz radiofrequency fields: preliminary observations

UR - https://www.ncbi.nlm.nih.gov/pmc/articles/PMC4848421/pdf/BMRI2016-4918691.pdf

ER -

TY - JOUR

IS - 3

JA - J Clin of Diagn Res

JO - Journal of Clinical and Diagnostic Research

PY - 2016

SN - 0973-709X

VL - 10

AU - Banerjee S

AU - Singh NN

AU - Sreedhar G

AU - Mukherjee S

DO - 10.7860/JCDR/2016/17592.7505

LA - en

N1 - FEMU ID: 29426; EMF-Portal URL: https://www.emf-portal.org/en/article/29426

SP - ZC82-ZC85

TI - Analysis of the Genotoxic Effects of Mobile Phone Radiation using Buccal Micronucleus Assay: A Comparative Evaluation

UR - https://www.ncbi.nlm.nih.gov/pmc/articles/PMC4843394/pdf/jcdr-10-ZC82.pdf

ER -

TY - JOUR

IS - 2

JA - Toxicol Res

JO - Toxicological Research

PY - 2016

SN - 1976-8257

VL - 32

AU - Nirwane A

AU - Sridhar V

AU - Majumdar A

DO - 10.5487/TR.2016.32.2.123

LA - en

N1 - FEMU ID: 29387; EMF-Portal URL: https://www.emf-portal.org/en/article/29387

SP - 123-132

TI - Neurobehavioural Changes and Brain Oxidative Stress Induced by Acute Exposure to GSM900 Mobile Phone Radiations in Zebrafish (Danio rerio)

UR - https://www.ncbi.nlm.nih.gov/pmc/articles/PMC4843974/pdf/tr-32-123.pdf

ER -

TY - JOUR

IS - 4

JA - Int J Impot Res

JO - International Journal of Impotence Research

PY - 2016

SN - 0955-9930

VL - 28

AU - Radwan M

AU - Jurewicz J

AU - Merecz-Kot D

AU - Sobala W

AU - Radwan P

AU - Bochenek M

AU - Hanke W

DO - 10.1038/ijir.2016.15

LA - en

N1 - FEMU ID: 29287; EMF-Portal URL: https://www.emf-portal.org/en/article/29287

SP - 148-154

TI - Sperm DNA damage - the effect of stress and everyday life factors

UR - https://www.nature.com/articles/ijir201615.pdf

ER -

TY - JOUR

JA - J Chem Neuroanat

JO - Journal of Chemical Neuroanatomy

PY - 2016

SN - 0891-0618

VL - 75

AU - Akdag MZ

AU - Dasdag S

AU - Canturk F

AU - Karabulut D

AU - Caner Y

AU - Adalier N

DO - 10.1016/j.jchemneu.2016.01.003

LA - en

N1 - FEMU ID: 28659; EMF-Portal URL: https://www.emf-portal.org/en/article/28659

SP - 116-122

TI - Does prolonged radiofrequency radiation emitted from Wi-Fi devices induce DNA damage in various tissues of rats?

ER -

TY - JOUR

JA - J Chem Neuroanat

JO - Journal of Chemical Neuroanatomy

PY - 2016

SN - 0891-0618

VL - 75

AU - Sahin D

AU - Ozgur E

AU - Guler G

AU - Tomruk A

AU - Unlu I

AU - Sepici-Dincel A

AU - Seyhan N

DO - 10.1016/j.jchemneu.2016.01.002

LA - en

N1 - FEMU ID: 28658; EMF-Portal URL: https://www.emf-portal.org/en/article/28658

SP - 94-98

TI - The 2100 MHz radiofrequency radiation of a 3G-mobile phone and the DNA oxidative damage in brain

ER -

TY - JOUR

JA - J Chem Neuroanat

JO - Journal of Chemical Neuroanatomy

PY - 2016

SN - 0891-0618

VL - 75

AU - Guler G

AU - Ozgur E

AU - Keles H

AU - Tomruk A

AU - Vural SA

AU - Seyhan N

DO - 10.1016/j.jchemneu.2015.10.006

LA - en

N1 - FEMU ID: 28169; EMF-Portal URL: https://www.emf-portal.org/en/article/28169

SP - 128-133

TI - Neurodegenerative changes and apoptosis induced by intrauterine and extrauterine exposure of radiofrequency radiation

ER -

TY - JOUR

IS - 2

JO - Mutagenesis

PY - 2016

SN - 0267-8357

VL - 31

AU - Gustavino B

AU - Carboni G

AU - Petrillo R

AU - Paoluzzi G

AU - Santovetti E

AU - Rizzoni M

DO - 10.1093/mutage/gev071

LA - en

N1 - FEMU ID: 28074; EMF-Portal URL: https://www.emf-portal.org/en/article/28074

SP - 187-192

TI - Exposure to 915 MHz radiation induces micronuclei in Vicia faba root tips

UR - https://academic.oup.com/mutage/article-pdf/31/2/187/8178338/gev071.pdf

ER -

TY - JOUR

IS - 3

JA - Arch Environ Contam Toxicol

JO - Archives of Environmental Contamination and Toxicology

PY - 2016

SN - 0090-4341

VL - 70

AU - Gulati S

AU - Yadav A

AU - Kumar N

AU - Kanupriya

AU - Aggarwal NK

AU - Kumar R

AU - Gupta R

DO - 10.1007/s00244-015-0195-y

LA - en

N1 - FEMU ID: 27671; EMF-Portal URL: https://www.emf-portal.org/en/article/27671

SP - 615-625

TI - Effect of GSTM1 and GSTT1 polymorphisms on genetic damage in humans populations exposed to radiation from mobile towers

ER -

TY - JOUR

IS - 3

JA - Anat Histol Embryol

JO - Anatomia, Histologia, Embryologia

PY - 2016

SN - 0340-2096

VL - 45

AU - Ye W

AU - Wang F

AU - Zhang W

AU - Fang N

AU - Zhao W

AU - Wang J

DO - 10.1111/ahe.12188

LA - en

N1 - FEMU ID: 27547; EMF-Portal URL: https://www.emf-portal.org/en/article/27547

SP - 197-208

TI - Effect of Mobile Phone Radiation on Cardiovascular Development of Chick Embryo

ER -

TY - JOUR

IS - 4

JA - Int J Hum Genet

JO - International Journal of Human Genetics

PY - 2015

SN - 0972-3757

VL - 15

AU - Gandhi G

AU - Singh P

AU - Kaur G

DO - 10.1080/09723757.2015.11886265

LA - en

N1 - FEMU ID: 47619; EMF-Portal URL: https://www.emf-portal.org/en/article/47619

SP - 173-182

TI - Perspectives Revisited - The Buccal Cytome Assay in Mobile Phone Users

UR - http://krepublishers.com/02-Journals/IJHG/IJHG-15-0-000-15-Web/IJHG-15-4-000-15-Abst-PDF/IJHG-15-4-173-15-606-Gandhi-G/IJHG-15-4-173-15-606-Gandhi-G-Tx[2].pdf

ER -

TY - JOUR

IS - 4

JA - Int J Radiat Res

JO - International Journal of Radiation Research

PY - 2015

SN - 2322-3243

VL - 13

AU - Mahmoudi R

AU - Mortazavi SMJ

AU - Safari S

AU - Nikseresht M

AU - Mozdarani H

AU - Jafari M

AU - Zamani A

AU - Haghani M

AU - Davari M

AU - Tabatabaie A

AU - Soleimani A

DO - 10.7508/ijrr.2015.04.010

LA - en

N1 - FEMU ID: 46193; EMF-Portal URL: https://www.emf-portal.org/en/article/46193

SP - 363-368

TI - Effects of microwave electromagnetic radiations emitted from common Wi-Fi routers on rats' sperm count and motility

ER -

TY - JOUR

IS - 5

JO - Biophysics

PY - 2015

SN - 0006-3509

VL - 60

AU - Gapeyev AB

AU - Lukyanova NA

DO - 10.1134/S0006350915050061

LA - en

N1 - FEMU ID: 29340; EMF-Portal URL: https://www.emf-portal.org/en/article/29340

SP - 732-738

TI - Pulse-modulated extremely high-frequency electromagnetic radiation protects cellular DNA from the damaging effects of physical and chemical factors in vitro

ER -

TY - GEN

ET - 1

PB - IEEE

PY - 2015

SN - 9781424492718

T2 - 2015 37th Annual International Conference of the IEEE Engineering in Medicine and Biology Society (EMBC), Milan, Italy

AU - Moraitis N

AU - Christopoulou M

AU - Nikita KS

AU - Voulgaridou GP

AU - Anestopoulos I

AU - Panagiotidis MI

AU - Pappa A

DO - 10.1109/EMBC.2015.7318922

LA - en

N1 - FEMU ID: 28545; EMF-Portal URL: https://www.emf-portal.org/en/article/28545

SP - 2592-2595

TI - In-vitro assessment of Jurkat T-cells response to 1966 MHz electromagnetic fields in a GTEM cell

ER -

TY - JOUR

JA - Mutat Res Genet Toxicol Environ Mutagen

JO - Mutation Research - Genetic Toxicology and Environmental Mutagenesis

PY - 2015

VL - 793

AU - Amicis A

AU - Sanctis S

AU - Cristofaro SD

AU - Franchini V

AU - Lista F

AU - Regalbuto E

AU - Giovenale E

AU - Gallerano GP

AU - Nenzi P

AU - Bei R

AU - Fantini M

AU - Benvenuto M

AU - Masuelli L

AU - Coluzzi E

AU - Cicia C

AU - Sgura A

DO - 10.1016/j.mrgentox.2015.06.003

LA - en

N1 - FEMU ID: 28170; EMF-Portal URL: https://www.emf-portal.org/en/article/28170

SP - 150-160

TI - Biological effects of in vitro THz radiation exposure in human foetal fibroblasts

ER -

TY - JOUR

JO - Neurotoxicology

PY - 2015

SN - 0161-813X

VL - 51

AU - Megha K

AU - Deshmukh PS

AU - Banerjee BD

AU - Tripathi AK

AU - Ahmed R

AU - Abegaonkar MP

DO - 10.1016/j.neuro.2015.10.009

LA - en

N1 - FEMU ID: 28149; EMF-Portal URL: https://www.emf-portal.org/en/article/28149

SP - 158-165

TI - Low intensity microwave radiation induced oxidative stress, inflammatory response and DNA damage in rat brain

ER -

TY - JOUR

IS - 1

JA - Braz Oral Res

JO - Brazilian Oral Research

PY - 2015

SN - 1806-8324

VL - 29

AU - Daroit NB

AU - Visioli F

AU - Magnusson AS

AU - Vieira GR

AU - Rados PV

DO - 10.1590/1807-3107BOR-2015.vol29.0114

LA - en

N1 - FEMU ID: 28094; EMF-Portal URL: https://www.emf-portal.org/en/article/28094

SP - 1-8

TI - Cell phone radiation effects on cytogenetic abnormalities of oral mucosal cells

UR - http://www.scielo.br/pdf/bor/v29n1/1807-3107-bor-29-1-1807-3107BOR-2015vol290114.pdf

ER -

TY - JOUR

IS - 3

JA - Adv Clin Exp Med

JO - Advances in Clinical and Experimental Medicine

PY - 2015

SN - 1899-5276

VL - 24

AU - Sokolovic D

AU - Djordjevic B

AU - Kocic G

AU - Stoimenov TJ

AU - Stanojkovic Z

AU - Sokolovic DM

AU - Veljkovic A

AU - Ristic G

AU - Despotovic M

AU - Milisavljevic D

AU - Jankovic R

AU - Binic I

DO - 10.17219/acem/43888

LA - en

N1 - FEMU ID: 28061; EMF-Portal URL: https://www.emf-portal.org/en/article/28061

SP - 429-436

TI - The Effects of Melatonin on Oxidative Stress Parameters and DNA Fragmentation in Testicular Tissue of Rats Exposed to Microwave Radiation

UR - http://www.advances.umed.wroc.pl/pdf/2015/24/3/429.pdf

ER -

TY - JOUR

IS - 3

JA - Cell Physiol Biochem

JO - Cellular Physiology and Biochemistry

PY - 2015

SN - 1015-8987

VL - 37

AU - Wang X

AU - Liu C

AU - Ma Q

AU - Feng W

AU - Yang L

AU - Lu Y

AU - Zhou Z

AU - Yu Z

AU - Li W

AU - Zhang L

DO - 10.1159/000430233

LA - en

N1 - FEMU ID: 27920; EMF-Portal URL: https://www.emf-portal.org/en/article/27920

SP - 1075-1088

TI - 8-oxoG DNA Glycosylase-1 Inhibition Sensitizes Neuro-2a Cells to Oxidative DNA Base Damage Induced by 900 MHz Radiofrequency Electromagnetic Radiation

UR - https://www.karger.com/Article/Pdf/430233

ER -

TY - JOUR

IS - 11

JA - Int J Radiat Biol

JO - International Journal of Radiation Biology

PY - 2015

SN - 0955-3002

VL - 91

AU - Furtado-Filho OV

AU - Borba JB

AU - Maraschin T

AU - Souza LM

AU - Henriques JA

AU - Moreira JC

AU - Saffi J

DO - 10.3109/09553002.2015.1083629

LA - en

N1 - FEMU ID: 27729; EMF-Portal URL: https://www.emf-portal.org/en/article/27729

SP - 891-897

TI - Effects of chronic exposure to 950 MHz ultra-high-frequency electromagnetic radiation on reactive oxygen species metabolism in the right and left cerebral cortex of young rats of different ages

ER -

TY - JOUR

IS - 3

JA - Endocr Regul

JO - Endocrine Regulations

PY - 2015

SN - 1210-0668

VL - 49

AU - Gurbuz N

AU - Sirav B

AU - Kuzay D

AU - Ozer C

AU - Seyhan N

DO - 10.4149/endo_2015_03_126

LA - en

N1 - FEMU ID: 27666; EMF-Portal URL: https://www.emf-portal.org/en/article/27666

SP - 126-130

TI - Does radio frequency radiation induce micronuclei frequency in exfoliated bladder cells of diabetic rats?

ER -

TY - JOUR

JA - J Neuroinflammation

JO - Journal of Neuroinflammation

PY - 2015

SN - 1742-2094

VL - 12

AU - Zuo WQ

AU - Hu YJ

AU - Yang Y

AU - Zhao XY

AU - Zhang YY

AU - Kong W

AU - Kong WJ

DO - 10.1186/s12974-015-0300-1

LA - en

N1 - FEMU ID: 27276; EMF-Portal URL: https://www.emf-portal.org/en/article/27276

SP - 105

TI - Sensitivity of spiral ganglion neurons to damage caused by mobile phone electromagnetic radiation will increase in lipopolysaccharide-induced inflammation in vitro model

UR - https://www.ncbi.nlm.nih.gov/pmc/articles/PMC4458026/pdf/12974_2015_Article_300.pdf

ER -

TY - JOUR

IS - 8

JA - Int J Radiat Biol

JO - International Journal of Radiation Biology

PY - 2015

SN - 0955-3002

VL - 91

AU - Kumar G

AU - McIntosh RL

AU - Anderson V

AU - McKenzie RJ

AU - Wood AW

DO - 10.3109/09553002.2015.1047988

LA - en

N1 - FEMU ID: 27083; EMF-Portal URL: https://www.emf-portal.org/en/article/27083

SP - 664-672

TI - A genotoxic analysis of the hematopoietic system after mobile phone type radiation exposure in rats

ER -

TY - JOUR

IS - 1

JA - Int J Fertil Steril

JO - International Journal of Fertility & Sterility

PY - 2015

SN - 2008-0778

VL - 9

AU - Zalata A

AU - El-Samanoudy AZ

AU - Shaalan D

AU - El-Baiomy Y

AU - Mostafa T

DO - 10.22074/ijfs.2015.4217

LA - en

N1 - FEMU ID: 26969; EMF-Portal URL: https://www.emf-portal.org/en/article/26969

SP - 129-136

TI - In vitro effect of cell phone radiation on motility, DNA fragmentation and clusterin gene expression in human sperm

UR - https://www.ncbi.nlm.nih.gov/pmc/articles/PMC4410031/pdf/Int-J-Fertil-Steril-9-129.pdf

ER -

TY - JOUR

IS - 4

JA - Int J Environ Res Public Health

JO - International Journal of Environmental Research and Public Health

PY - 2015

SN - 1660-4601

VL - 12

AU - Mizuno K

AU - Shinohara N

AU - Miyakoshi J

DO - 10.3390/ijerph120403853

LA - en

N1 - FEMU ID: 26850; EMF-Portal URL: https://www.emf-portal.org/en/article/26850

SP - 3853-3863

TI - In Vitro Evaluation of Genotoxic Effects under Magnetic Resonant Coupling Wireless Power Transfer

UR - http://www.mdpi.com/1660-4601/12/4/3853

ER -

TY - JOUR

IS - 3

JA - Int J Toxicol

JO - International Journal of Toxicology

PY - 2015

SN - 1091-5818

VL - 34

AU - Deshmukh PS

AU - Nasare N

AU - Megha K

AU - Banerjee BD

AU - Ahmed RS

AU - Singh D

AU - Abegaonkar MP

AU - Tripathi AK

AU - Mediratta PK

DO - 10.1177/1091581815574348

LA - en

N1 - FEMU ID: 26650; EMF-Portal URL: https://www.emf-portal.org/en/article/26650

SP - 284-290

TI - Cognitive impairment and neurogenotoxic effects in rats exposed to low-intensity microwave radiation

ER -

TY - JOUR

IS - 3

JA - Radiat Res

JO - Radiation Research

PY - 2015

SN - 0033-7587

VL - 183

AU - Duan W

AU - Liu C

AU - Zhang L

AU - He M

AU - Xu S

AU - Chen C

AU - Pi H

AU - Gao P

AU - Zhang Y

AU - Zhong M

AU - Yu Z

AU - Zhou Z

DO - 10.1667/RR13851.1

LA - en

N1 - FEMU ID: 26541; EMF-Portal URL: https://www.emf-portal.org/en/article/26541

SP - 305-314

TI - Comparison of the genotoxic effects induced by 50 Hz extremely low-frequency electromagnetic fields and 1800 MHz radiofrequency electromagnetic fields in GC-2 cells

ER -

TY - JOUR

IS - 2

JO - Bioelectromagnetics

PY - 2015

SN - 0197-8462

VL - 36

AU - Halgamuge MN

AU - Yak SK

AU - Eberhardt JL

DO - 10.1002/BEM.21890

LA - en

N1 - FEMU ID: 26476; EMF-Portal URL: https://www.emf-portal.org/en/article/26476

SP - 87-95

TI - Reduced growth of soybean seedlings after exposure to weak microwave radiation from GSM 900 mobile phone and base station

ER -

TY - JOUR

JA - Sci Rep

JO - Scientific Reports

PY - 2015

SN - 2045-2322

VL - 5

AU - Bogomazova AN

AU - Vassina EM

AU - Goryachkovskaya TN

AU - Popik VM

AU - Sokolov AS

AU - Kolchanov NA

AU - Lagarkova MA

AU - Kiselev SL

AU - Peltek SE

DO - 10.1038/srep07749

LA - en

N1 - FEMU ID: 26377; EMF-Portal URL: https://www.emf-portal.org/en/article/26377

SP - 7749

TI - No DNA damage response and negligible genome-wide transcriptional changes in human embryonic stem cells exposed to terahertz radiation

UR - http://www.nature.com/srep/2015/150113/srep07749/pdf/srep07749.pdf

ER -

TY - JOUR

IS - 3

JA - Int J Radiat Biol

JO - International Journal of Radiation Biology

PY - 2015

SN - 0955-3002

VL - 91

AU - Zong C

AU - Ji Y

AU - He Q

AU - Zhu S

AU - Qin F

AU - Tong J

AU - Cao Y

DO - 10.3109/09553002.2014.980465

LA - en

N1 - FEMU ID: 25895; EMF-Portal URL: https://www.emf-portal.org/en/article/25895

SP - 270-276

TI - Adaptive response in mice exposed to 900 MHz radiofrequency fields: Bleomycin-induced DNA and oxidative damage/repair

ER -

TY - JOUR

IS - 3

JA - Mol Neurobiol

JO - Molecular Neurobiology

PY - 2015

SN - 0893-7648

VL - 51

AU - Zuo H

AU - Lin T

AU - Wang D

AU - Peng R

AU - Wang S

AU - Gao Y

AU - Xu X

AU - Zhao L

AU - Wang S

AU - Su Z

DO - 10.1007/s12035-014-8831-5

LA - en

N1 - FEMU ID: 25610; EMF-Portal URL: https://www.emf-portal.org/en/article/25610

SP - 1520-1529

TI - RKIP Regulates Neural Cell Apoptosis Induced by Exposure to Microwave Radiation Partly Through the MEK/ERK/CREB Pathway

ER -

TY - JOUR

IS - 4

JA - Electromagn Biol Med

JO - Electromagnetic Biology and Medicine

PY - 2015

SN - 1536-8386

VL - 34

AU - Gandhi G

AU - Kaur G

AU - Nisar U

DO - 10.3109/15368378.2014.933349

LA - en

N1 - FEMU ID: 25331; EMF-Portal URL: https://www.emf-portal.org/en/article/25331

SP - 344-354

TI - A cross-sectional case control study on genetic damage in individuals residing in the vicinity of a mobile phone base station

ER -

TY - JOUR

IS - 1

JA - Electromagn Biol Med

JO - Electromagnetic Biology and Medicine

PY - 2015

SN - 1536-8386

VL - 34

AU - Hou Q

AU - Wang M

AU - Wu S

AU - Ma X

AU - An G

AU - Liu H

AU - Xie F

DO - 10.3109/15368378.2014.900507

LA - en

N1 - FEMU ID: 24552; EMF-Portal URL: https://www.emf-portal.org/en/article/24552

SP - 85-92

TI - Oxidative changes and apoptosis induced by 1800-MHz electromagnetic radiation in NIH/3T3 cells

ER -

TY - GEN

ET - 1

PB - IEEE

PY - 2014

SN - 9781479956913

T2 - 2014 IEEE 5th Control and System Graduate Research Colloquium, Shah Alam, Malaysia

AU - Ma YP

AU - Haron MH

AU - Taib MN

AU - Jailani R

DO - 10.1109/ICSGRC.2014.6908738

LA - en

N1 - FEMU ID: 49747; EMF-Portal URL: https://www.emf-portal.org/en/article/49747

SP - 285-289

TI - Emotional identification during mobile RF Radiation

ER -

TY - JOUR

IS - 10

JA - Cent Eur J Biol

JO - Central European Journal of Biology

PY - 2014

SN - 1895-104X

VL - 9

AU - Gapeyev AB

AU - Lukyanova NA

AU - Gudkov SV

DO - 10.2478/s11535-014-0326-x

LA - en

N1 - FEMU ID: 49139; EMF-Portal URL: https://www.emf-portal.org/en/article/49139

SP - 915-921

TI - Hydrogen peroxide induced by modulated electromagnetic radiation protects the cells from DNA damage

UR - https://www.degruyter.com/document/doi/10.2478/s11535-014-0326-x/pdf

ER -

TY - JOUR

IS - 4

JO - Journal of Applied Science and Environmental Management: JASEM

PY - 2014

SN - 1119-8362

VL - 18

AU - Adebayo EA

AU - Adeeyo AO

AU - Ayandele AA

AU - Omomowo IO

DO - 10.4314/jasem.v18i4.16

LA - en

N1 - FEMU ID: 38207; EMF-Portal URL: https://www.emf-portal.org/en/article/38207

SP - 669-674

TI - Effect of Radiofrequency Radiation from Telecommunication Base Stations on Microbial Diversity and Antibiotic Resistance

UR - https://www.ajol.info/index.php/jasem/article/download/112366/102119

ER -

TY - JOUR

IS - 4

JO - Trace Elements and Electrolytes

PY - 2014

SN - 0946-2104

VL - 31

AU - Zhang Y

AU - An K

AU - Yin MS

AU - Yin LL

AU - Guo DM

AU - Cui X

DO - 10.5414/TEX01352

LA - en

N1 - FEMU ID: 31253; EMF-Portal URL: https://www.emf-portal.org/en/article/31253

SP - 174-180

TI - Effect of radiofrequency electromagnetic radiation emitted by mobile phone on trace elements levels in fetal rats

ER -

TY - JOUR

IS - 9

JA - Indian J Exp Biol

JO - Indian Journal of Experimental Biology

PY - 2014

SN - 0019-5189

VL - 52

AU - Kumar S

AU - Nirala JP

AU - Behari J

AU - Paulraj R

LA - en

N1 - FEMU ID: 25773; EMF-Portal URL: https://www.emf-portal.org/en/article/25773

SP - 890-897

TI - Effect of electromagnetic irradiation produced by 3G mobile phone on male rat reproductive system in a simulated scenario

UR - http://nopr.niscpr.res.in/bitstream/123456789/29335/1/IJEB%2052%289%29%20890-897.pdf

ER -

TY - JOUR

IS - 1

JA - Cent European J Urol

JO - Central European Journal of Urology

PY - 2014

SN - 2080-4806

VL - 67

AU - Gorpinchenko I

AU - Nikitin O

AU - Banyra O

AU - Shulyak A

DO - 10.5173/ceju.2014.01.art14

LA - en

N1 - FEMU ID: 25254; EMF-Portal URL: https://www.emf-portal.org/en/article/25254

SP - 65-71

TI - The influence of direct mobile phone radiation on sperm quality

UR - http://ceju.online/journal/2014/commenting-on-gorpinchenko-et-al-the-influence-of-direct-mobile-phone-radiation-on-sperm-439.php

ER -

TY - JOUR

IS - 10

JA - Int J Radiat Biol

JO - International Journal of Radiation Biology

PY - 2014

SN - 0955-3002

VL - 90

AU - Gürler HS

AU - Bilgici B

AU - Akar AK

AU - Tomak L

AU - Bedir A

DO - 10.3109/09553002.2014.922717

LA - en

N1 - FEMU ID: 24955; EMF-Portal URL: https://www.emf-portal.org/en/article/24955

SP - 892-896

TI - Increased DNA oxidation (8-OHdG) and protein oxidation (AOPP) by Low level electromagnetic field (2.45 GHz) in rat brain and protective effect of garlic

ER -

TY - JOUR

IS - 2

JA - Cell Biochem Biophys

JO - Cell Biochemistry and Biophysics

PY - 2014

SN - 1085-9195

VL - 70

AU - Ozgur E

AU - Guler G

AU - Kismali G

AU - Seyhan N

DO - 10.1007/s12013-014-0007-4

LA - en

N1 - FEMU ID: 24865; EMF-Portal URL: https://www.emf-portal.org/en/article/24865

SP - 983-991

TI - Mobile phone radiation alters proliferation of hepatocarcinoma cells

ER -

TY - JOUR

IS - 2

JA - Cell Biochem Biophys

JO - Cell Biochemistry and Biophysics

PY - 2014

SN - 1085-9195

VL - 70

AU - Motawi TK

AU - Darwish HA

AU - Moustafa YM

AU - Labib MM

DO - 10.1007/s12013-014-9990-8

LA - en

N1 - FEMU ID: 24796; EMF-Portal URL: https://www.emf-portal.org/en/article/24796

SP - 845-855

TI - Biochemical modifications and neuronal damage in brain of young and adult rats after long-term exposure to mobile phone radiations

ER -

TY - JOUR

IS - 5

JA - Int J Med Sci

JO - International Journal of Medical Sciences

PY - 2014

SN - 1449-1907

VL - 11

AU - Zuo H

AU - Lin T

AU - Wang D

AU - Peng R

AU - Wang S

AU - Gao Y

AU - Xu X

AU - Li Y

AU - Wang S

AU - Zhao L

AU - Wang L

AU - Zhou H

DO - 10.7150/ijms.6540

LA - en

N1 - FEMU ID: 24567; EMF-Portal URL: https://www.emf-portal.org/en/article/24567

SP - 426-435

TI - Neural cell apoptosis induced by microwave exposure through mitochondria-dependent caspase-3 pathway

UR - https://www.ncbi.nlm.nih.gov/pmc/articles/PMC3970093/pdf/ijmsv11p0426.pdf

ER -

TY - JOUR

IS - 4

JA - Electromagn Biol Med

JO - Electromagnetic Biology and Medicine

PY - 2014

SN - 1536-8386

VL - 33

AU - Gurbuz N

AU - Sirav B

AU - Colbay M

AU - Yetkin I

AU - Seyhan N

DO - 10.3109/15368378.2013.831354

LA - en

N1 - FEMU ID: 23927; EMF-Portal URL: https://www.emf-portal.org/en/article/23927

SP - 296-301

TI - No genotoxic effect in exfoliated bladder cells of rat under the exposure of 1800 and 2100 MHz radio frequency radiation

ER -

TY - JOUR

IS - 2

JA - J Radiat Res

JO - Journal of Radiation Research

PY - 2014

SN - 0449-3060

VL - 55

AU - Kang KA

AU - Lee HC

AU - Lee JJ

AU - Hong MN

AU - Park MJ

AU - Lee YS

AU - Choi HD

AU - Kim N

AU - Ko YK

AU - Lee JS

DO - 10.1093/jrr/rrt116

LA - en

N1 - FEMU ID: 23673; EMF-Portal URL: https://www.emf-portal.org/en/article/23673

SP - 265-276

TI - Effects of combined radiofrequency radiation exposure on levels of reactive oxygen species in neuronal cells

UR - https://academic.oup.com/jrr/article-pdf/55/2/265/2803067/rrt116.pdf

ER -

TY - JOUR

IS - 1

JA - Syst Biol Reprod Med

JO - Systems Biology in Reproductive Medicine

PY - 2014

SN - 1939-6368

VL - 60

AU - Jurewicz J

AU - Radwan M

AU - Sobala W

AU - Ligocka D

AU - Radwan P

AU - Bochenek M

AU - Hanke W

DO - 10.3109/19396368.2013.840687

LA - en

N1 - FEMU ID: 23596; EMF-Portal URL: https://www.emf-portal.org/en/article/23596

SP - 43-51

TI - Lifestyle and semen quality: role of modifiable risk factors

ER -

TY - JOUR

IS - 2

JA - J Radiat Res

JO - Journal of Radiation Research

PY - 2014

SN - 0449-3060

VL - 55

AU - Sannino A

AU - Zeni O

AU - Romeo S

AU - Massa R

AU - Gialanella G

AU - Grossi G

AU - Manti L

AU - Vijayalaxmi

AU - Scarfi MR

DO - 10.1093/jrr/rrt106

LA - en

N1 - FEMU ID: 23403; EMF-Portal URL: https://www.emf-portal.org/en/article/23403

SP - 210-217

TI - Adaptive response in human blood lymphocytes exposed to non-ionizing radiofrequency fields: resistance to ionizing radiation-induced damage

UR - https://academic.oup.com/jrr/article-pdf/55/2/210/2797100/rrt106.pdf

ER -

TY - JOUR

IS - 2

JA - Cell Biochem Biophys

JO - Cell Biochemistry and Biophysics

PY - 2014

SN - 1085-9195

VL - 68

AU - Kesari KK

AU - Meena R

AU - Nirala J

AU - Kumar J

AU - Verma HN

DO - 10.1007/s12013-013-9715-4

LA - en

N1 - FEMU ID: 23361; EMF-Portal URL: https://www.emf-portal.org/en/article/23361

SP - 347-358

TI - Effect of 3G Cell Phone Exposure with Computer Controlled 2-D Stepper Motor on Non-thermal Activation of the hsp27/p38MAPK Stress Pathway in Rat Brain

ER -

TY - JOUR

IS - 2

JA - Int J Radiat Biol

JO - International Journal of Radiation Biology

PY - 2014

SN - 0955-3002

VL - 90

AU - Furtado-Filho OV

AU - Borba JB

AU - Dallegrave A

AU - Pizzolato TM

AU - Henriques JA

AU - Moreira JC

AU - Saffi J

DO - 10.3109/09553002.2013.817697

LA - en

N1 - FEMU ID: 22969; EMF-Portal URL: https://www.emf-portal.org/en/article/22969

SP - 159-168

TI - Effect of 950 MHz UHF Electromagnetic radiation on biomarkers of oxidative damage, metabolism of UFA and antioxidants in the liver of young rats of different ages

ER -

TY - JOUR

IS - 2

JA - Electromagn Biol Med

JO - Electromagnetic Biology and Medicine

PY - 2014

SN - 1536-8386

VL - 33

AU - Souza LCM

AU - Cerqueira Ede M

AU - Meireles JR

DO - 10.3109/15368378.2013.783856

LA - en

N1 - FEMU ID: 22645; EMF-Portal URL: https://www.emf-portal.org/en/article/22645

SP - 98-102

TI - Assessment of nuclear abnormalities in exfoliated cells from the oral epithelium of mobile phone users

ER -

TY - JOUR

IS - 2

JA - Electromagn Biol Med

JO - Electromagnetic Biology and Medicine

PY - 2014

SN - 1536-8386

VL - 33

AU - Meena R

AU - Kumari K

AU - Kumar J

AU - Rajamani P

AU - Verma HN

AU - Kesari KK

DO - 10.3109/15368378.2013.781035

LA - en

N1 - FEMU ID: 22452; EMF-Portal URL: https://www.emf-portal.org/en/article/22452

SP - 81-91

TI - Therapeutic approaches of melatonin in microwave radiations-induced oxidative stress-mediated toxicity on male fertility pattern of Wistar rats

ER -

TY - JOUR

IS - 11

JO - Zahedan Journal of Research in Medical Sciences

PY - 2013

SN - 2383-2894

VL - 15

AU - Zahedifar Z

AU - Baharara J

LA - en

N1 - FEMU ID: 39150; EMF-Portal URL: https://www.emf-portal.org/en/article/39150

SP - 39-44

TI - Effect of Green Tea Extract in Reducing Genotoxic Injuries of Cell Phone Microwaves on Bone Marrow

UR - https://admin.kowsarpub.com/cdn/dl/28fbf5fa-6ccf-11e9-b7d0-8329d5be8d6a

ER -

TY - JOUR

IS - 11

JA - J Endocrinol Invest

JO - Journal of Endocrinological Investigation

PY - 2013

SN - 0391-4097

VL - 36

AU - Rago R

AU - Salacone P

AU - Caponecchia L

AU - Sebastianelli A

AU - Marcucci I

AU - Calogero AE

AU - Condorelli R

AU - Vicari E

AU - Morgia G

AU - Favilla V

AU - Cimino S

AU - Arcoria AF

AU - La Vignera S

DO - 10.3275/8996

LA - en

N1 - FEMU ID: 25784; EMF-Portal URL: https://www.emf-portal.org/en/article/25784

SP - 970-974

TI - The semen quality of the mobile phone users

ER -

TY - JOUR

JA - Reprod Toxicol

JO - Reproductive Toxicology

PY - 2013

SN - 0890-6238

VL - 42

AU - Hanci H

AU - Odaci E

AU - Kaya H

AU - Aliyazicioglu Y

AU - Turan I

AU - Demir S

AU - Colakoglu S

DO - 10.1016/j.reprotox.2013.09.006

LA - en

N1 - FEMU ID: 23648; EMF-Portal URL: https://www.emf-portal.org/en/article/23648

SP - 203-209

TI - The effect of prenatal exposure to 900-megahertz electromagnetic field on the 21-old-day rat testicle

ER -

TY - JOUR

IS - 3

JA - Exp Oncol

JO - Experimental Oncology

PY - 2013

SN - 1812-9269

VL - 35

AU - Burlaka A

AU - Tsybulin O

AU - Sidorik E

AU - Lukin S

AU - Polishuk V

AU - Tsehmistrenko S

AU - Yakymenko I

LA - en

N1 - FEMU ID: 23628; EMF-Portal URL: https://www.emf-portal.org/en/article/23628

SP - 219-225

TI - Overproduction of free radical species in embryonal cells exposed to low intensity radiofrequency radiation

UR - https://exp-oncology.com.ua/article/6079

ER -

TY - JOUR

IS - 11

JA - Int J Radiat Biol

JO - International Journal of Radiation Biology

PY - 2013

SN - 0955-3002

VL - 89

AU - Liu C

AU - Gao P

AU - Xu SC

AU - Wang Y

AU - Chen CH

AU - He MD

AU - Yu ZP

AU - Zhang L

AU - Zhou Z

DO - 10.3109/09553002.2013.811309

LA - en

N1 - FEMU ID: 23360; EMF-Portal URL: https://www.emf-portal.org/en/article/23360

SP - 993-1001

TI - Mobile phone radiation induces mode-dependent DNA damage in a mouse spermatocyte-derived cell line: a protective role of melatonin

ER -

TY - JOUR

IS - 1

JA - Toxicol Int

JO - Toxicology International

PY - 2013

SN - 0971-6580

VL - 20

AU - Deshmukh PS

AU - Megha K

AU - Banerjee BD

AU - Ahmed RS

AU - Chandna S

AU - Abegaonkar MP

AU - Tripathi AK

DO - 10.4103/0971-6580.111549

LA - en

N1 - FEMU ID: 22984; EMF-Portal URL: https://www.emf-portal.org/en/article/22984

SP - 19-24

TI - Detection of Low Level Microwave Radiation Induced Deoxyribonucleic Acid Damage Vis-a-vis Genotoxicity in Brain of Fischer Rats

UR - https://www.ncbi.nlm.nih.gov/pmc/articles/PMC3702122/?report=printable

ER -

TY - JOUR

IS - 2

JA - Mutat Res Genet Toxicol Environ Mutagen

JO - Mutation Research - Genetic Toxicology and Environmental Mutagenesis

PY - 2013

VL - 755

AU - Speit G

AU - Gminski R

AU - Tauber R

DO - 10.1016/j.mrgentox.2013.06.014

LA - en

N1 - FEMU ID: 22960; EMF-Portal URL: https://www.emf-portal.org/en/article/22960

SP - 163-166

TI - Genotoxic effects of exposure to radiofrequency electromagnetic fields (RF-EMF) in HL-60 cells are not reproducible

ER -

TY - JOUR

IS - 7

JO - Bioelectromagnetics

PY - 2013

SN - 0197-8462

VL - 34

AU - Vijayalaxmi

AU - Reddy AB

AU - McKenzie RJ

AU - McIntosh RL

AU - Prihoda TJ

AU - Wood AW

DO - 10.1002/bem.21798

LA - en

N1 - FEMU ID: 22648; EMF-Portal URL: https://www.emf-portal.org/en/article/22648

SP - 542-548

TI - Incidence of micronuclei in human peripheral blood lymphocytes exposed to modulated and unmodulated 2450 MHz radiofrequency fields

ER -

TY - JOUR

IS - 11

JA - Int J Radiat Biol

JO - International Journal of Radiation Biology

PY - 2013

SN - 0955-3002

VL - 89

AU - Atli Sekeroglu Z

AU - Akar A

AU - Sekeroglu V

DO - 10.3109/09553002.2013.809170

LA - en

N1 - FEMU ID: 22646; EMF-Portal URL: https://www.emf-portal.org/en/article/22646

SP - 985-992

TI - Evaluation of the cytogenotoxic damage in immature and mature rats exposed to 900 MHz radiofrequency electromagnetic fields

ER -

TY - JOUR

IS - 10

JA - Int J Radiat Biol

JO - International Journal of Radiation Biology

PY - 2013

SN - 0955-3002

VL - 89

AU - Szerencsi A

AU - Kubinyi G

AU - Valiczko E

AU - Juhasz P

AU - Rudas G

AU - Mester A

AU - Janossy G

AU - Bakos J

AU - Thuroczy G

DO - 10.3109/09553002.2013.804962

LA - en

N1 - FEMU ID: 22464; EMF-Portal URL: https://www.emf-portal.org/en/article/22464

SP - 870-876

TI - DNA Integrity of Human Leukocytes after Magnetic Resonance Imaging

ER -

TY - JOUR

IS - 9

JA - Int J Radiat Biol

JO - International Journal of Radiation Biology

PY - 2013

SN - 0955-3002

VL - 89

AU - Tsybulin O

AU - Sidorik E

AU - Brieieva O

AU - Buchynska L

AU - Kyrylenko S

AU - Henshel D

AU - Yakymenko I

DO - 10.3109/09553002.2013.791408

LA - en

N1 - FEMU ID: 22134; EMF-Portal URL: https://www.emf-portal.org/en/article/22134

SP - 756-763

TI - GSM 900 MHz cellular phone radiation can either stimulate or depress early embryogenesis in Japanese quails depending on the duration of exposure

ER -

TY - JOUR

IS - 4

JA - Biomed Opt Express

JO - Biomedical Optics Express

PY - 2013

SN - 2156-7085

VL - 4

AU - Titova LV

AU - Ayesheshim AK

AU - Golubov A

AU - Fogen D

AU - Rodriguez-Juarez R

AU - Hegmann FA

AU - Kovalchuk O

DO - 10.1364/BOE.4.000559

LA - en

N1 - FEMU ID: 22051; EMF-Portal URL: https://www.emf-portal.org/en/article/22051

SP - 559-568

TI - Intense THz pulses cause H2AX phosphorylation and activate DNA damage response in human skin tissue

UR - https://opg.optica.org/boe/viewmedia.cfm?uri=boe-4-4-559&seq=0

ER -

TY - JOUR

IS - 1

JA - Gen Physiol Biophys

JO - General Physiology and Biophysics

PY - 2013

SN - 0231-5882

VL - 32

AU - Sokolovic D

AU - Djordjevic B

AU - Kocic G

AU - Veljkovic A

AU - Marinkovic M

AU - Basic J

AU - Jevtovic-Stoimenov T

AU - Stanojkovic Z

AU - Sokolovic DM

AU - Pavlovic V

AU - Djindjic B

AU - Krstic D

DO - 10.4149/gpb_2013002

LA - en

N1 - FEMU ID: 21991; EMF-Portal URL: https://www.emf-portal.org/en/article/21991

SP - 79-90

TI - Melatonin protects rat thymus against oxidative stress caused by exposure to microwaves and modulates proliferation/apoptosis of thymocytes

ER -

TY - JOUR

IS - 1

JA - Biochem Biophys Res Commun

JO - Biochemical and Biophysical Research Communications

PY - 2013

SN - 0006-291X

VL - 433

AU - Zhijian C

AU - Xiaoxue L

AU - Wei Z

AU - Yezhen L

AU - Jianlin L

AU - Deqiang L

AU - Shijie C

AU - Lifen J

AU - Jiliang H

DO - 10.1016/j.bbrc.2013.02.071

LA - en

N1 - FEMU ID: 21865; EMF-Portal URL: https://www.emf-portal.org/en/article/21865

SP - 36-39

TI - Studying the protein expression in human B lymphoblastoid cells exposed to 1.8-GHz (GSM) radiofrequency radiation (RFR) with protein microarray

ER -

TY - JOUR

JA - Ecotoxicol Environ Saf

JO - Ecotoxicology and Environmental Safety

PY - 2013

SN - 0147-6513

VL - 90

AU - Tkalec M

AU - Stambuk A

AU - Srut M

AU - Malaric K

AU - Klobucar GI

DO - 10.1016/j.ecoenv.2012.12.005

LA - en

N1 - FEMU ID: 21702; EMF-Portal URL: https://www.emf-portal.org/en/article/21702

SP - 7-12

TI - Oxidative and genotoxic effects of 900 MHz electromagnetic fields in the earthworm Eisenia fetida

ER -

TY - JOUR

IS - 1

JO - PLoS One

PY - 2013

SN - 1932-6203

VL - 8

AU - Xu S

AU - Chen G

AU - Chen C

AU - Sun C

AU - Zhang D

AU - Murbach M

AU - Kuster N

AU - Zeng Q

AU - Xu Z

DO - 10.1371/journal.pone.0054906

LA - en

N1 - FEMU ID: 21701; EMF-Portal URL: https://www.emf-portal.org/en/article/21701

SP - e54906

TI - Cell Type-Dependent Induction of DNA Damage by 1800 MHz Radiofrequency Electromagnetic Fields Does Not Result in Significant Cellular Dysfunctions

UR - http://journals.plos.org/plosone/article?id=10.1371/journal.pone.0054906

ER -

TY - JOUR

IS - 1

JA - Toxicol Lett

JO - Toxicology Letters

PY - 2013

SN - 0378-4274

VL - 218

AU - Liu C

AU - Duan W

AU - Xu S

AU - Chen C

AU - He M

AU - Zhang L

AU - Yu Z

AU - Zhou Z

DO - 10.1016/j.toxlet.2013.01.003

LA - en

N1 - FEMU ID: 21674; EMF-Portal URL: https://www.emf-portal.org/en/article/21674

SP - 2-9

TI - Exposure to 1800 MHz radiofrequency electromagnetic radiation induces oxidative DNA base damage in a mouse spermatocyte-derived cell line

ER -

TY - JOUR

IS - 5

JA - Appl Biochem Biotechnol

JO - Applied Biochemistry and Biotechnology

PY - 2013

SN - 0273-2289

VL - 169

AU - Shahin S

AU - Singh VP

AU - Shukla RK

AU - Dhawan A

AU - Gangwar RK

AU - Singh SP

AU - Chaturvedi CM

DO - 10.1007/s12010-012-0079-9

LA - en

N1 - FEMU ID: 21672; EMF-Portal URL: https://www.emf-portal.org/en/article/21672

SP - 1727-1751

TI - 2.45 GHz microwave irradiation-induced oxidative stress affects implantation or pregnancy in mice, Mus musculus

ER -

TY - JOUR

IS - 2

JA - Radiat Res

JO - Radiation Research

PY - 2013

SN - 0033-7587

VL - 179

AU - Waldmann P

AU - Bohnenberger S

AU - Greinert R

AU - Hermann-Then B

AU - Heselich A

AU - Klug SJ

AU - Koenig J

AU - Kuhr K

AU - Kuster N

AU - Merker M

AU - Murbach M

AU - Pollet D

AU - Schadenboeck W

AU - Scheidemann-Wesp U

AU - Schwab B

AU - Volkmer B

AU - Weyer V

AU - Blettner M

DO - 10.1667/RR2914.1

LA - en

N1 - FEMU ID: 21641; EMF-Portal URL: https://www.emf-portal.org/en/article/21641

SP - 243-253

TI - Influence of GSM Signals on Human Peripheral Lymphocytes: Study of Genotoxicity

ER -

TY - JOUR

IS - 2

JA - Mutat Res Genet Toxicol Environ Mutagen

JO - Mutation Research - Genetic Toxicology and Environmental Mutagenesis

PY - 2013

VL - 751

AU - Jiang B

AU - Zong C

AU - Zhao H

AU - Ji Y

AU - Tong J

AU - Cao Y

DO - 10.1016/j.mrgentox.2012.12.003

LA - en

N1 - FEMU ID: 21616; EMF-Portal URL: https://www.emf-portal.org/en/article/21616

SP - 127-129

TI - Induction of adaptive response in mice exposed to 900MHz radiofrequency fields: application of micronucleus assay

ER -

TY - JOUR

IS - 3

JA - Arch Microbiol

JO - Archives of Microbiology

PY - 2013

SN - 0302-8933

VL - 195

AU - Berzin V

AU - Kiriukhin M

AU - Tyurin M

DO - 10.1007/s00203-012-0862-6

LA - en

N1 - FEMU ID: 21588; EMF-Portal URL: https://www.emf-portal.org/en/article/21588

SP - 181-188

TI - "Curing" of plasmid DNA in acetogen using microwave or applying an electric pulse improves cell growth and metabolite production as compared to the plasmid-harboring strain

ER -

TY - JOUR

IS - 1

JA - Radiat Res

JO - Radiation Research

PY - 2013

SN - 0033-7587

VL - 179

AU - Hintzsche H

AU - Jastrow C

AU - Heinen B

AU - Baaske K

AU - Kleine-Ostmann T

AU - Schwerdtfeger M

AU - Shakfa MK

AU - Karst U

AU - Koch M

AU - Schrader T

AU - Stopper H

DO - 10.1667/RR3077.1

LA - en

N1 - FEMU ID: 21496; EMF-Portal URL: https://www.emf-portal.org/en/article/21496

SP - 38-45

TI - Terahertz Radiation at 0.380 THz and 2.520 THz Does Not Lead to DNA Damage in Skin Cells In Vitro

ER -

TY - JOUR

IS - 3

JA - Int J Radiat Biol

JO - International Journal of Radiation Biology

PY - 2013

SN - 0955-3002

VL - 89

AU - Kumar S

AU - Behari J

AU - Sisodia R

DO - 10.3109/09553002.2013.741282

LA - en

N1 - FEMU ID: 21367; EMF-Portal URL: https://www.emf-portal.org/en/article/21367

SP - 147-154

TI - Influence of electromagnetic fields on reproductive system of male rats

ER -

TY - JOUR

IS - 1-2

JA - Mutat Res Genet Toxicol Environ Mutagen

JO - Mutation Research - Genetic Toxicology and Environmental Mutagenesis

PY - 2013

VL - 750

AU - Pesnya DS

AU - Romanovsky AV

DO - 10.1016/j.mrgentox.2012.08.010

LA - en

N1 - FEMU ID: 21320; EMF-Portal URL: https://www.emf-portal.org/en/article/21320

SP - 27-33

TI - Comparison of cytotoxic and genotoxic effects of plutonium-239 alpha particles and mobile phone GSM 900 radiation in the Allium cepa test

ER -

TY - JOUR

IS - 1

JO - Bioelectromagnetics

PY - 2013

SN - 0197-8462

VL - 34

AU - Bourthoumieu S

AU - Magnaudeix A

AU - Terro F

AU - Leveque P

AU - Collin A

AU - Yardin C

DO - 10.1002/bem.21744

LA - en

N1 - FEMU ID: 20967; EMF-Portal URL: https://www.emf-portal.org/en/article/20967

SP - 52-60

TI - Study of p53 expression and post-transcriptional modifications after GSM-900 radiofrequency exposure of human amniotic cells

ER -

TY - JOUR

IS - 2

JA - J Pediatr Urol

JO - Journal of Pediatric Urology

PY - 2013

SN - 1477-5131

VL - 9

AU - Atasoy HI

AU - Gunal MY

AU - Atasoy P

AU - Elgun S

AU - Bugdayci G

DO - 10.1016/j.jpurol.2012.02.015

LA - en

N1 - FEMU ID: 20439; EMF-Portal URL: https://www.emf-portal.org/en/article/20439

SP - 223-229

TI - Immunohistopathologic demonstration of deleterious effects on growing rat testes of radiofrequency waves emitted from conventional Wi-Fi devices

ER -

TY - JOUR

IS - 2

JA - J Appl Pharm Sci

JO - Journal of Applied Pharmaceutical Science

PY - 2012

SN - 2231-3354

VL - 2

AU - El-Abd SF

AU - Eltoweissy MY

LA - en

N1 - FEMU ID: 47219; EMF-Portal URL: https://www.emf-portal.org/en/article/47219

SP - 16-20

TI - Cytogenetic alterations in human lymphocyte culture following exposure to radiofrequency field of mobile phone

UR - https://www.japsonline.com/admin/php/uploads/372_pdf.pdf

ER -

TY - JOUR

IS - 9

JO - PLoS One

PY - 2012

SN - 1932-6203

VL - 7

AU - Hintzsche H

AU - Jastrow C

AU - Kleine-Ostmann T

AU - Karst U

AU - Schrader T

AU - Stopper H

DO - 10.1371/journal.pone.0046397

LA - en

N1 - FEMU ID: 21295; EMF-Portal URL: https://www.emf-portal.org/en/article/21295

SP - e46397

TI - Terahertz electromagnetic fields (0.106 THz) do not induce manifest genomic damage in vitro

UR - https://www.ncbi.nlm.nih.gov/pmc/articles/PMC3459899/pdf/pone.0046397.pdf

ER -

TY - JOUR

JA - Oxid Med Cell Longev

JO - Oxidative Medicine and Cellular Longevity

PY - 2012

SN - 1942-0994

AU - Lu YS

AU - Huang BT

AU - Huang YX

DO - 10.1155/2012/740280

LA - en

N1 - FEMU ID: 20976; EMF-Portal URL: https://www.emf-portal.org/en/article/20976

SP - 740280

TI - Reactive Oxygen Species Formation and Apoptosis in Human Peripheral Blood Mononuclear Cell Induced by 900 MHz Mobile Phone Radiation

UR - https://www.hindawi.com/journals/oximed/2012/740280/

ER -

TY - JOUR

IS - 8

JA - Oral Dis

JO - Oral Diseases

PY - 2012

SN - 1354-523X

VL - 18

AU - Ros-Llor I

AU - Sanchez-Siles M

AU - Camacho-Alonso F

AU - Lopez-Jornet P

DO - 10.1111/j.1601-0825.2012.01946.x

LA - en

N1 - FEMU ID: 20877; EMF-Portal URL: https://www.emf-portal.org/en/article/20877

SP - 786-792

TI - Effect of mobile phones on micronucleus frequency in human exfoliated oral mucosal cells

ER -

TY - JOUR

IS - 7

JO - Bioelectromagnetics

PY - 2012

SN - 0197-8462

VL - 33

AU - Hong MN

AU - Kim BC

AU - Ko YG

AU - Lee YS

AU - Hong SC

AU - Kim T

AU - Pack JK

AU - Choi HD

AU - Kim N

AU - Lee JS

DO - 10.1002/bem.21731

LA - en

N1 - FEMU ID: 20693; EMF-Portal URL: https://www.emf-portal.org/en/article/20693

SP - 604-611

TI - Effects of 837 and 1950 MHz radiofrequency radiation exposure alone or combined on oxidative stress in MCF10A cells

ER -

TY - JOUR

IS - 1

JA - Mutat Res Genet Toxicol Environ Mutagen

JO - Mutation Research - Genetic Toxicology and Environmental Mutagenesis

PY - 2012

VL - 747

AU - Zeni O

AU - Sannino A

AU - Romeo S

AU - Massa R

AU - Sarti M

AU - Reddy AB

AU - Prihoda TJ

AU - Vijayalaxmi

AU - Scarfi MR

DO - 10.1016/j.mrgentox.2012.03.013

LA - en

N1 - FEMU ID: 20662; EMF-Portal URL: https://www.emf-portal.org/en/article/20662

SP - 29-35

TI - Induction of an adaptive response in human blood lymphocytes exposed to radiofrequency fields: Influence of the universal mobile telecommunication system (UMTS) signal and the specific absorption rate

ER -

TY - JOUR

IS - 2

JA - J Radiat Res

JO - Journal of Radiation Research

PY - 2012

SN - 0449-3060

VL - 53

AU - Kim HN

AU - Han NK

AU - Hong MN

AU - Chi SG

AU - Lee YS

AU - Kim T

AU - Pack JK

AU - Choi HD

AU - Kim N

AU - Lee JS

DO - 10.1269/jrr.11048

LA - en

N1 - FEMU ID: 20543; EMF-Portal URL: https://www.emf-portal.org/en/article/20543

SP - 176-183

TI - Analysis of the cellular stress response in MCF10A cells exposed to combined radio frequency radiation

UR - https://www.jstage.jst.go.jp/article/jrr/53/2/53_11048/_pdf

ER -

TY - JOUR

IS - 14

JA - J Agric Food Chem

JO - Journal of Agricultural and Food Chemistry

PY - 2012

SN - 0021-8561

VL - 60

AU - Pande R

AU - Mishra HN

AU - Singh MN

DO - 10.1021/jf204540n

LA - en

N1 - FEMU ID: 20386; EMF-Portal URL: https://www.emf-portal.org/en/article/20386

SP - 3809-3816

TI - Microwave drying for safe storage and improved nutritional quality of green gram seed (Vigna radiata)

ER -

TY - JOUR

IS - 4

JO - Mutagenesis

PY - 2012

SN - 0267-8357

VL - 27

AU - Hintzsche H

AU - Jastrow C

AU - Kleine-Ostmann T

AU - Schrader T

AU - Stopper H

DO - 10.1093/mutage/ges007

LA - en

N1 - FEMU ID: 20370; EMF-Portal URL: https://www.emf-portal.org/en/article/20370

SP - 477-483

TI - 900 MHz radiation does not induce micronucleus formation in different cell types

UR - https://academic.oup.com/mutage/article-pdf/27/4/477/3889278/ges007.pdf

ER -

TY - JOUR

JA - Ecotoxicol Environ Saf

JO - Ecotoxicology and Environmental Safety

PY - 2012

SN - 0147-6513

VL - 80

AU - Sekeroglu V

AU - Akar A

AU - Sekeroglu ZA

DO - 10.1016/j.ecoenv.2012.02.028

LA - en

N1 - FEMU ID: 20362; EMF-Portal URL: https://www.emf-portal.org/en/article/20362

SP - 140-144

TI - Cytotoxic and genotoxic effects of high-frequency electromagnetic fields (GSM 1800MHz) on immature and mature rats

ER -

TY - JOUR

IS - 2

JO - PLoS One

PY - 2012

SN - 1932-6203

VL - 7

AU - Jiang B

AU - Nie J

AU - Zhou Z

AU - Zhang J

AU - Tong J

AU - Cao Y

DO - 10.1371/journal.pone.0032040

LA - en

N1 - FEMU ID: 20338; EMF-Portal URL: https://www.emf-portal.org/en/article/20338

SP - e32040

TI - Adaptive Response in Mice Exposed to 900 MHz Radiofrequency Fields: Primary DNA Damage

UR - http://journals.plos.org/plosone/article?id=10.1371/journal.pone.0032040

ER -

TY - JOUR

IS - 2

JA - Cell Biochem Biophys

JO - Cell Biochemistry and Biophysics

PY - 2012

SN - 1085-9195

VL - 63

AU - Panagopoulos DJ

DO - 10.1007/s12013-012-9347-0

LA - en

N1 - FEMU ID: 20327; EMF-Portal URL: https://www.emf-portal.org/en/article/20327

SP - 121-132

TI - Effect of microwave exposure on the ovarian development of Drosophila melanogaster

ER -

TY - JOUR

IS - 6

JO - Bioelectromagnetics

PY - 2012

SN - 0197-8462

VL - 33

AU - Zeni O

AU - Sannino A

AU - Sarti M

AU - Romeo S

AU - Massa R

AU - Scarfi MR

DO - 10.1002/bem.21712

LA - en

N1 - FEMU ID: 20288; EMF-Portal URL: https://www.emf-portal.org/en/article/20288

SP - 497-507

TI - Radiofrequency radiation at 1950 MHz (UMTS) does not affect key cellular endpoints in neuron-like PC12 cells

ER -

TY - JOUR

IS - 5

JA - Int J Radiat Biol

JO - International Journal of Radiation Biology

PY - 2012

SN - 0955-3002

VL - 88

AU - Cam ST

AU - Seyhan N

DO - 10.3109/09553002.2012.666005

LA - en

N1 - FEMU ID: 20278; EMF-Portal URL: https://www.emf-portal.org/en/article/20278

SP - 420-424

TI - Single-strand DNA breaks in human hair root cells exposed to mobile phone radiation

ER -

TY - JOUR

IS - 1

JA - Electromagn Biol Med

JO - Electromagnetic Biology and Medicine

PY - 2012

SN - 1536-8386

VL - 31

AU - Trivino Pardo JC

AU - Grimaldi S

AU - Taranta M

AU - Naldi I

AU - Cinti C

DO - 10.3109/15368378.2011.596251

LA - en

N1 - FEMU ID: 20263; EMF-Portal URL: https://www.emf-portal.org/en/article/20263

SP - 1-18

TI - Microwave electromagnetic field regulates gene expression in T-lymphoblastoid leukemia CCRF-CEM cell line exposed to 900 MHz

ER -

TY - JOUR

IS - 7

JA - Hum Exp Toxicol

JO - Human & Experimental Toxicology

PY - 2012

SN - 0960-3271

VL - 31

AU - Khalil AM

AU - Gagaa MH

AU - Alshamali AM

DO - 10.1177/0960327111433184

LA - en

N1 - FEMU ID: 20105; EMF-Portal URL: https://www.emf-portal.org/en/article/20105

SP - 734-740

TI - 8-Oxo-7, 8-dihydro-2'-deoxyguanosine as a biomarker of DNA damage by mobile phone radiation

ER -

TY - JOUR

IS - 4

JA - Int J Radiat Biol

JO - International Journal of Radiation Biology

PY - 2012

SN - 0955-3002

VL - 88

AU - Guler G

AU - Tomruk A

AU - Ozgur E

AU - Sahin D

AU - Sepici A

AU - Altan N

AU - Seyhan N

DO - 10.3109/09553002.2012.646349

LA - en

N1 - FEMU ID: 19950; EMF-Portal URL: https://www.emf-portal.org/en/article/19950

SP - 367-373

TI - The effect of radiofrequency radiation on DNA and lipid damage in female and male infant rabbits

ER -

TY - JOUR

IS - 1

JA - Fertil Steril

JO - Fertility and Sterility

PY - 2012

SN - 0015-0282

VL - 97

AU - Avendano C

AU - Mata A

AU - Sanchez Sarmiento CA

AU - Doncel GF

DO - 10.1016/j.fertnstert.2011.10.012

LA - en

N1 - FEMU ID: 19930; EMF-Portal URL: https://www.emf-portal.org/en/article/19930

SP - 39-45.e2

TI - Use of laptop computers connected to internet through Wi-Fi decreases human sperm motility and increases sperm DNA fragmentation

ER -

TY - JOUR

IS - 4

JO - Bioelectromagnetics

PY - 2012

SN - 0197-8462

VL - 33

AU - Lee HJ

AU - Jin YB

AU - Kim TH

AU - Pack JK

AU - Kim N

AU - Choi HD

AU - Lee JS

AU - Lee YS

DO - 10.1002/bem.20715

LA - en

N1 - FEMU ID: 19804; EMF-Portal URL: https://www.emf-portal.org/en/article/19804

SP - 356-364

TI - The effects of simultaneous combined exposure to CDMA and WCDMA electromagnetic fields on rat testicular function

ER -

TY - JOUR

IS - 1

JA - J Neurooncol

JO - Journal of Neuro-Oncology

PY - 2012

SN - 0167-594X

VL - 106

AU - Karaca E

AU - Durmaz B

AU - Aktug H

AU - Yildiz T

AU - Guducu C

AU - Irgi M

AU - Koksal MG

AU - Ozkinay F

AU - Gunduz C

AU - Cogulu O

DO - 10.1007/s11060-011-0644-z

LA - en

N1 - FEMU ID: 19410; EMF-Portal URL: https://www.emf-portal.org/en/article/19410

SP - 53-58

TI - The genotoxic effect of radiofrequency waves on mouse brain

ER -

TY - JOUR

IS - 4

JO - Biophysics

PY - 2011

SN - 0006-3509

VL - 56

AU - Gapeyev AB

AU - Romanova NA

AU - Chemeris NK

DO - 10.1134/S0006350911040087

LA - en

N1 - FEMU ID: 22828; EMF-Portal URL: https://www.emf-portal.org/en/article/22828

SP - 672-678

TI - Changes in the chromatin structure of lymphoid cells under the influence of low-intensity extremely high-frequency electromagnetic radiation against the background of inflammatory process

ER -

TY - JOUR

IS - 4

JA - Coll Antropol

JO - Collegium Antropologicum

PY - 2011

SN - 0350-6134

VL - 35

AU - Trosic I

AU - Pavicic I

AU - Milkovic-Kraus S

AU - Mladinic M

AU - Zeljezic D

LA - en

N1 - FEMU ID: 20359; EMF-Portal URL: https://www.emf-portal.org/en/article/20359

SP - 1259-1264

TI - Effect of electromagnetic radiofrequency radiation on the rats' brain, liver and kidney cells measured by comet assay

UR - https://hrcak.srce.hr/file/112380

ER -

TY - JOUR

JA - World Acad Sci Eng Technol

JO - World Academy of Science, Engineering and Technology

PY - 2011

VL - 76

AU - Khalil AM

AU - Alshamali AM

AU - Gagaa MH

LA - en

N1 - FEMU ID: 20110; EMF-Portal URL: https://www.emf-portal.org/en/article/20110

SP - 657-622

TI - Detection of oxidative stress induced by mobile phone radiation in tissues of mice using 8-oxo-7, 8-dihydro-20-deoxyguanosine as a biomarker

UR - http://publications.waset.org/7054/pdf

ER -

TY - JOUR

IS - 4

JA - Electromagn Biol Med

JO - Electromagnetic Biology and Medicine

PY - 2011

SN - 1536-8386

VL - 30

AU - Zeng L

AU - Ji X

AU - Zhang Y

AU - Miao X

AU - Zou C

AU - Lang H

AU - Zhang J

AU - Li Y

AU - Wang X

AU - Qi H

AU - Ren D

AU - Guo G

DO - 10.3109/15368378.2011.587929

LA - en

N1 - FEMU ID: 19856; EMF-Portal URL: https://www.emf-portal.org/en/article/19856

SP - 205-218

TI - MnSOD expression inhibited by electromagnetic pulse radiation in the rat testis

ER -

TY - JOUR

JA - Sci Total Environ

JO - Science of the Total Evironment

PY - 2011

SN - 0048-9697

VL - 410

AU - Esmekaya MA

AU - Aytekin E

AU - Ozgur E

AU - Güler G

AU - Ergun MA

AU - Omeroglu S

AU - Seyhan N

DO - 10.1016/j.scitotenv.2011.09.036

LA - en

N1 - FEMU ID: 19800; EMF-Portal URL: https://www.emf-portal.org/en/article/19800

SP - 59-64

TI - Mutagenic and morphologic impacts of 1.8 GHz radiofrequency radiation on human peripheral blood lymphocytes (hPBLs) and possible protective role of pre-treatment with Ginkgo biloba (EGb 761)

ER -

TY - JOUR

JA - Progr Electromagn Res B (PIER B)

JO - Progress in Electromagnetics Research B

PY - 2011

SN - 1937-6472

VL - 29

AU - Chaturvedi CM

AU - Singh VP

AU - Singh P

AU - Basu P

AU - Singaravel M

AU - Shukla RK

AU - Dhawan A

AU - Pati AK

AU - Gangwar RK

AU - Singh SP

DO - 10.2528/PIERB11011205

LA - en

N1 - FEMU ID: 19608; EMF-Portal URL: https://www.emf-portal.org/en/article/19608

SP - 23-42

TI - 2.45 GHz (CW) microwave irradiation alters circadian organization, spatial memory, DNA structure in the brain cells and blood cell counts of male mice, Mus musculus

UR - https://www.jpier.org/ac_api/download.php?id=11011205

ER -

TY - JOUR

IS - 7

JO - Clinics

PY - 2011

SN - 1807-5932

VL - 66

AU - Kumar S

AU - Kesari KK

AU - Behari J

DO - 10.1590/s1807-59322011000700020

LA - en

N1 - FEMU ID: 19575; EMF-Portal URL: https://www.emf-portal.org/en/article/19575

SP - 1237-1245

TI - The therapeutic effect of a pulsed electromagnetic field on the reproductive patterns of male Wistar rats exposed to a 2.45-GHz microwave field

UR - http://www.scielo.br/pdf/clin/v66n7/v66n7a20.pdf

ER -

TY - JOUR

IS - 9

JA - Int J Radiat Biol

JO - International Journal of Radiation Biology

PY - 2011

SN - 0955-3002

VL - 87

AU - Sannino A

AU - Zeni O

AU - Sarti M

AU - Romeo S

AU - Reddy SB

AU - Belisario MA

AU - Prihoda TJ

AU - Vijayalaxmi

AU - Scarfi MR

DO - 10.3109/09553002.2011.574779

LA - en

N1 - FEMU ID: 19265; EMF-Portal URL: https://www.emf-portal.org/en/article/19265

SP - 993-999

TI - Induction of adaptive response in human blood lymphocytes exposed to 900 MHz radiofrequency fields: Influence of cell cycle

ER -

TY - JOUR

IS - 4

JA - Appl Biochem Biotechnol

JO - Applied Biochemistry and Biotechnology

PY - 2011

SN - 0273-2289

VL - 164

AU - Kesari KK

AU - Kumar S

AU - Behari J

DO - 10.1007/s12010-010-9156-0

LA - en

N1 - FEMU ID: 18931; EMF-Portal URL: https://www.emf-portal.org/en/article/18931

SP - 546-559

TI - Effects of radiofrequency electromagnetic wave exposure from cellular phones on the reproductive pattern in male wistar rats

ER -

TY - JOUR

IS - 2

JA - Int J Radiat Biol

JO - International Journal of Radiation Biology

PY - 2011

SN - 0955-3002

VL - 87

AU - Kumar G

AU - Wood AW

AU - Anderson V

AU - McIntosh RL

AU - Chen YY

AU - McKenzie RJ

DO - 10.3109/09553002.2010.518212

LA - en

N1 - FEMU ID: 18740; EMF-Portal URL: https://www.emf-portal.org/en/article/18740

SP - 231-240

TI - Evaluation of hematopoietic system effects after in vitro radiofrequency radiation exposure in rats

ER -

TY - JOUR

IS - 1

JA - Int J Hyg Environ Health

JO - International Journal of Hygiene and Environmental Health

PY - 2011

SN - 1438-4639

VL - 214

AU - Garaj-Vrhovac V

AU - Gajski G

AU - Pazanin S

AU - Sarolic A

AU - Domijan AM

AU - Flajs D

AU - Peraica M

DO - 10.1016/j.ijheh.2010.08.003

LA - en

N1 - FEMU ID: 18617; EMF-Portal URL: https://www.emf-portal.org/en/article/18617

SP - 59-65

TI - Assessment of cytogenetic damage and oxidative stress in personnel occupationally exposed to the pulsed microwave radiation of marine radar equipment

ER -

TY - JOUR

IS - 2

JA - Lasers Surg Med

JO - Lasers in Surgery and Medicine

PY - 2011

SN - 0196-8092

VL - 43

AU - Wilmink GJ

AU - Rivest BD

AU - Roth CC

AU - Ibey BL

AU - Payne JA

AU - Cundin LX

AU - Grundt JE

AU - Peralta X

AU - Mixon DG

AU - Roach WP

DO - 10.1002/lsm.20960

LA - en

N1 - FEMU ID: 18578; EMF-Portal URL: https://www.emf-portal.org/en/article/18578

SP - 152-163

TI - In vitro investigation of the biological effects associated with human dermal fibroblasts exposed to 2.52 THz radiation

ER -

TY - JOUR

IS - 6

JA - Toxicol Environ Chem

JO - Toxicological and Environmental Chemistry

PY - 2010

SN - 0092-9867

VL - 92

AU - Kesari KK

AU - Behari J

DO - 10.1080/02772240903233637

LA - en

N1 - FEMU ID: 28577; EMF-Portal URL: https://www.emf-portal.org/en/article/28577

SP - 1135-1147

TI - Effects of microwave at 2.45 GHz radiations on reproductive system of male rats

ER -

TY - JOUR

IS - 9-10

JA - CR physique

JO - Comptes Rendus Physique

PY - 2010

SN - 1631-0705

VL - 11

AU - Perrin A

AU - Freire M

AU - Bachelet C

AU - Collin A

AU - Leveque P

AU - Pla S

AU - Debouzy JC

DO - 10.1016/j.crhy.2010.10.006

LA - en

N1 - FEMU ID: 19405; EMF-Portal URL: https://www.emf-portal.org/en/article/19405

SP - 613-621

TI - Evaluation of the co-genotoxic effects of 1800 MHz GSM radiofrequency exposure and a chemical mutagen in cultured human cells

ER -

TY - JOUR

IS - 10

JA - J Zhejiang Univ Sci B

JO - Journal of Zhejiang University Science B

PY - 2010

SN - 1673-1581

VL - 11

AU - Shckorbatov YG

AU - Pasiuga VN

AU - Goncharuk EI

AU - Petrenko TP

AU - Grabina VA

AU - Kolchigin NN

AU - Ivanchenko DD

AU - Bykov VN

AU - Dumin OM

DO - 10.1631/jzus.B1000051

LA - en

N1 - FEMU ID: 18648; EMF-Portal URL: https://www.emf-portal.org/en/article/18648

SP - 801-805

TI - Effects of differently polarized microwave radiation on the microscopic structure of the nuclei in human fibroblasts

UR - https://www.ncbi.nlm.nih.gov/pmc/articles/PMC2950243/pdf/JZUSB11-0801.pdf

ER -

TY - JOUR

IS - 6

JA - Radiat Res

JO - Radiation Research

PY - 2010

SN - 0033-7587

VL - 174

AU - Bourthoumieu S

AU - Joubert V

AU - Marin B

AU - Collin A

AU - Leveque P

AU - Terro F

AU - Yardin C

LA - en

N1 - FEMU ID: 18635; EMF-Portal URL: https://www.emf-portal.org/en/article/18635

SP - 712-718

TI - Cytogenetic Studies in Human Cells Exposed In Vitro to GSM-900 MHz Radiofrequency Radiation Using R-Banded Karyotyping

ER -

TY - JOUR

IS - 6

JA - Indian J Exp Biol

JO - Indian Journal of Experimental Biology

PY - 2010

SN - 0019-5189

VL - 48

AU - Kumar S

AU - Kesari KK

AU - Behari J

LA - en

N1 - FEMU ID: 18567; EMF-Portal URL: https://www.emf-portal.org/en/article/18567

SP - 586-592

TI - Evaluation of genotoxic effects in male Wistar rats following microwave exposure

UR - http://nopr.niscpr.res.in/bitstream/123456789/9081/1/IJEB%2048%286%29%20586-592.pdf

ER -

TY - JOUR

IS - 3

JA - Electromagn Biol Med

JO - Electromagnetic Biology and Medicine

PY - 2010

SN - 1536-8386

VL - 29

AU - Gurbuz N

AU - Sirav B

AU - Yuvaci HU

AU - Turhan N

AU - Coskun ZK

AU - Seyhan N

DO - 10.3109/15368378.2010.482498

LA - en

N1 - FEMU ID: 18549; EMF-Portal URL: https://www.emf-portal.org/en/article/18549

SP - 98-104

TI - Is There Any Possible Genotoxic Effect in Exfoliated Bladder Cells of Rat Under the Exposure of 1800 MHz GSM-Like Modulated Radio Frequency Radiation (RFR)?

ER -

TY - JOUR

IS - 2

JA - Genet Couns

JO - Genetic Counseling

PY - 2010

SN - 1015-8146

VL - 21

AU - Yildirim MS

AU - Yildirim A

AU - Zamani AG

AU - Okudan N

LA - en

N1 - FEMU ID: 18526; EMF-Portal URL: https://www.emf-portal.org/en/article/18526

SP - 243-251

TI - Effect of mobile phone station on micronucleus frequency and chromosomal aberrations in human blood cells

ER -

TY - JOUR

IS - 3

JA - Oncol Rep

JO - Oncology Reports

PY - 2010

SN - 1021-335X

VL - 24

AU - Motomura T

AU - Ueda K

AU - Ohtani S

AU - Hansen E

AU - Ji L

AU - Ito K

AU - Saito K

AU - Sugita Y

AU - Nose Y

DO - 10.3892/or_00000896

LA - en

N1 - FEMU ID: 18489; EMF-Portal URL: https://www.emf-portal.org/en/article/18489

SP - 591-598

TI - Evaluation of systemic external microwave hyperthermia for treatment of pleural metastasis in orthotopic lung cancer model

UR - https://www.spandidos-publications.com/or/24/3/591/download

ER -

TY - JOUR

IS - 1-2

JA - Mutat Res Genet Toxicol Environ Mutagen

JO - Mutation Research - Genetic Toxicology and Environmental Mutagenesis

PY - 2010

VL - 700

AU - Chavdoula ED

AU - Panagopoulos DJ

AU - Margaritis LH

DO - 10.1016/j.mrgentox.2010.05.008

LA - en

N1 - FEMU ID: 18222; EMF-Portal URL: https://www.emf-portal.org/en/article/18222

SP - 51-61

TI - Comparison of biological effects between continuous and intermittent exposure to GSM-900-MHz mobile phone radiation: Detection of apoptotic cell-death features

ER -

TY - JOUR

IS - 2

JA - Radiat Res

JO - Radiation Research

PY - 2010

SN - 0033-7587

VL - 174

AU - Falzone N

AU - Huyser C

AU - Franken DR

AU - Leszczynski D

DO - 10.1667/RR2091.1

LA - en

N1 - FEMU ID: 18217; EMF-Portal URL: https://www.emf-portal.org/en/article/18217

SP - 169-176

TI - Mobile Phone Radiation Does Not Induce Pro-apoptosis Effects in Human Spermatozoa

ER -

TY - JOUR

IS - 6

JO - Bioelectromagnetics

PY - 2010

SN - 0197-8462

VL - 31

AU - Luukkonen J

AU - Juutilainen J

AU - Naarala J

DO - 10.1002/bem.20580

LA - en

N1 - FEMU ID: 18142; EMF-Portal URL: https://www.emf-portal.org/en/article/18142

SP - 417-424

TI - Combined effects of 872 MHz radiofrequency radiation and ferrous chloride on reactive oxygen species production and DNA damage in human SH-SY5Y neuroblastoma cells

ER -

TY - JOUR

IS - 5

JA - Int J Radiat Biol

JO - International Journal of Radiation Biology

PY - 2010

SN - 0955-3002

VL - 86

AU - Panagopoulos DJ

AU - Chavdoula ED

AU - Margaritis LH

DO - 10.3109/09553000903567961

LA - en

N1 - FEMU ID: 18122; EMF-Portal URL: https://www.emf-portal.org/en/article/18122

SP - 345-357

TI - Bioeffects of mobile telephony radiation in relation to its intensity or distance from the antenna

ER -

TY - JOUR

IS - 1

JA - Gen Physiol Biophys

JO - General Physiology and Biophysics

PY - 2010

SN - 0231-5882

VL - 29

AU - Güler G

AU - Tomruk A

AU - Ozgur E

AU - Seyhan N

DO - 10.4149/gpb_2010_01_59

LA - en

N1 - FEMU ID: 18104; EMF-Portal URL: https://www.emf-portal.org/en/article/18104

SP - 59-66

TI - The effect of radiofrequency radiation on DNA and lipid damage in non-pregnant and pregnant rabbits and their newborns

ER -

TY - JOUR

IS - 4

JA - Int J Radiat Biol

JO - International Journal of Radiation Biology

PY - 2010

SN - 0955-3002

VL - 86

AU - Kesari KK

AU - Behari J

AU - Kumar S

DO - 10.3109/09553000903564059

LA - en

N1 - FEMU ID: 18089; EMF-Portal URL: https://www.emf-portal.org/en/article/18089

SP - 334-343

TI - Mutagenic response of 2.45 GHz radiation exposure on rat brain

ER -

TY - JOUR

IS - 1

JA - Neurosci Lett

JO - Neuroscience Letters

PY - 2010

SN - 0304-3940

VL - 473

AU - Campisi A

AU - Gulino M

AU - Acquaviva R

AU - Bellia P

AU - Raciti G

AU - Grasso R

AU - Musumeci F

AU - Vanella A

AU - Triglia A

DO - 10.1016/j.neulet.2010.02.018

LA - en

N1 - FEMU ID: 17968; EMF-Portal URL: https://www.emf-portal.org/en/article/17968

SP - 52-55

TI - Reactive oxygen species levels and DNA fragmentation on astrocytes in primary culture after acute exposure to low intensity microwave electromagnetic field

ER -

TY - JOUR

IS - 6

JA - Int Arch Occup Environ Health

JO - International Archives of Occupational and Environmental Health

PY - 2010

SN - 0340-0131

VL - 83

AU - Gerner C

AU - Haudek V

AU - Schandl U

AU - Bayer E

AU - Gundacker N

AU - Hutter HP

AU - Mosgoeller W

DO - 10.1007/s00420-010-0513-7

LA - en

N1 - FEMU ID: 17958; EMF-Portal URL: https://www.emf-portal.org/en/article/17958

SP - 691-702

TI - Increased protein synthesis by cells exposed to a 1,800-MHz radio-frequency mobile phone electromagnetic field, detected by proteome profiling

ER -

TY - JOUR

IS - 3

JA - Environ Health Perspect

JO - Environmental Health Perspectives

PY - 2010

SN - 0091-6765

VL - 118

AU - Belyaev I

AU - Markova E

AU - Malmgren L

DO - 10.1289/ehp.0900781

LA - en

N1 - FEMU ID: 17858; EMF-Portal URL: https://www.emf-portal.org/en/article/17858

SP - 394-399

TI - Microwaves from Mobile Phones Inhibit 53BP1 Focus Formation in Human Stem Cells Stronger than in Differentiated Cells: Possible Mechanistic Link to Cancer Risk

UR - https://www.ncbi.nlm.nih.gov/pmc/articles/PMC2854769/pdf/ehp-118-394.pdf

ER -

TY - JOUR

IS - 1

JA - Toxicol Lett

JO - Toxicology Letters

PY - 2010

SN - 0378-4274

VL - 193

AU - Hintzsche H

AU - Stopper H

DO - 10.1016/j.toxlet.2009.12.016

LA - en

N1 - FEMU ID: 17832; EMF-Portal URL: https://www.emf-portal.org/en/article/17832

SP - 124-130

TI - Micronucleus frequency in buccal mucosa cells of mobile phone users

ER -

TY - JOUR

JA - Brain Res

JO - Brain Research

PY - 2010

SN - 0006-8993

VL - 1311

AU - Xu S

AU - Zhou Z

AU - Zhang L

AU - Yu Z

AU - Zhang W

AU - Wang Y

AU - Wang X

AU - Li M

AU - Chen Y

AU - Chen C

AU - He M

AU - Zhang G

AU - Zhong M

DO - 10.1016/j.brainres.2009.10.062

LA - en

N1 - FEMU ID: 17674; EMF-Portal URL: https://www.emf-portal.org/en/article/17674

SP - 189-196

TI - Exposure to 1800 MHz radiofrequency radiation induces oxidative damage to mitochondrial DNA in primary cultured neurons

ER -

TY - JOUR

IS - 1

JA - Cell Biochem Biophys

JO - Cell Biochemistry and Biophysics

PY - 2010

SN - 1085-9195

VL - 56

AU - Tomruk A

AU - Güler G

AU - Dincel AS

DO - 10.1007/s12013-009-9068-1

LA - en

N1 - FEMU ID: 17640; EMF-Portal URL: https://www.emf-portal.org/en/article/17640

SP - 39-47

TI - The influence of 1800 MHz GSM-like signals on hepatic oxidative DNA and lipid damage in nonpregnant, pregnant, and newly born rabbits

ER -

TY - JOUR

IS - 1-2

JA - Mutat Res Genet Toxicol Environ Mutagen

JO - Mutation Research - Genetic Toxicology and Environmental Mutagenesis

PY - 2010

VL - 695

AU - Zhijian C

AU - Xiaoxue L

AU - Yezhen L

AU - Shijie C

AU - Lifen J

AU - Jianlin L

AU - Deqiang L

AU - Jiliang H

DO - 10.1016/j.mrgentox.2009.10.001

LA - en

N1 - FEMU ID: 17623; EMF-Portal URL: https://www.emf-portal.org/en/article/17623

SP - 16-21

TI - Impact of 1.8-GHz radiofrequency radiation (RFR) on DNA damage and repair induced by doxorubicin in human B-cell lymphoblastoid cells

ER -

TY - JOUR

IS - 1-2

JO - Mutation Research - Fundamental and Molecular Mechanism of Mutagenesis

PY - 2010

SN - 0027-5107

VL - 683

AU - Franzellitti S

AU - Valbonesi P

AU - Ciancaglini N

AU - Biondi C

AU - Contin A

AU - Bersani F

AU - Fabbri E

DO - 10.1016/j.mrfmmm.2009.10.004

LA - en

N1 - FEMU ID: 17612; EMF-Portal URL: https://www.emf-portal.org/en/article/17612

SP - 35-42

TI - Transient DNA damage induced by high-frequency electromagnetic fields (GSM 1.8GHz) in the human trophoblast HTR-8/SVneo cell line evaluated with the alkaline comet assay

ER -

TY - JOUR

IS - 1-2

JA - Int J Med Biol Front

JO - International Journal of Medical and Biological Frontiers

PY - 2009

SN - 1081-3829

VL - 15

AU - Panagopoulos DJ

AU - Margaritis LH

LA - en

N1 - FEMU ID: 18335; EMF-Portal URL: https://www.emf-portal.org/en/article/18335

SP - 33-76

TI - Biological and Health Effects of Mobile Telephone Radiations

ER -

TY - JOUR

IS - 11

JA - Anticancer Res

JO - Anticancer Research

PY - 2009

SN - 0250-7005

VL - 29

AU - Hansteen IL

AU - Clausen KO

AU - Haugan V

AU - Svendsen M

AU - Svendsen MV

AU - Eriksen JG

AU - Skiaker R

AU - Hauger E

AU - Lageide L

AU - Vistnes AI

AU - Kure EH

LA - en

N1 - FEMU ID: 17824; EMF-Portal URL: https://www.emf-portal.org/en/article/17824

SP - 4323-4330

TI - Cytogenetic effects of exposure to 2.3 GHz radiofrequency radiation on human lymphocytes in vitro

UR - http://ar.iiarjournals.org/content/29/11/4323.full.pdf+html

ER -

TY - JOUR

IS - 2

JA - Int J Toxicol

JO - International Journal of Toxicology

PY - 2009

SN - 1091-5818

VL - 28

AU - Gajski G

AU - Garaj-Vrhovac V

DO - 10.1177/1091581809335051

LA - en

N1 - FEMU ID: 17452; EMF-Portal URL: https://www.emf-portal.org/en/article/17452

SP - 88-98

TI - Radioprotective effects of honeybee venom (Apis mellifera) against 915-MHz microwave radiation-induced DNA damage in wistar rat lymphocytes: in vitro study

ER -

TY - JOUR

IS - 8

JA - Anticancer Res

JO - Anticancer Research

PY - 2009

SN - 0250-7005

VL - 29

AU - Hansteen IL

AU - Lageide L

AU - Clausen KO

AU - Haugan V

AU - Svendsen M

AU - Eriksen JG

AU - Skiaker R

AU - Hauger E

AU - Vistnes AI

AU - Kure EH

LA - en

N1 - FEMU ID: 17426; EMF-Portal URL: https://www.emf-portal.org/en/article/17426

SP - 2885-2892

TI - Cytogenetic effects of 18.0 and 16.5 GHz microwave radiation on human lymphocytes in vitro

UR - http://ar.iiarjournals.org/content/29/8/2885.full.pdf+html

ER -

TY - JOUR

IS - 7

JO - PLoS One

PY - 2009

SN - 1932-6203

VL - 4

AU - De Iuliis GN

AU - Newey RJ

AU - King BV

AU - Aitken RJ

DO - 10.1371/journal.pone.0006446

LA - en

N1 - FEMU ID: 17394; EMF-Portal URL: https://www.emf-portal.org/en/article/17394

SP - e6446

TI - Mobile phone radiation induces reactive oxygen species production and DNA damage in human spermatozoa in vitro

UR - https://journals.plos.org/plosone/article/file?id=10.1371/journal.pone.0006446&type=printable

ER -

TY - JOUR

IS - 1-2

JA - Mutat Res Genet Toxicol Environ Mutagen

JO - Mutation Research - Genetic Toxicology and Environmental Mutagenesis

PY - 2009

VL - 677

AU - Zhijian C

AU - Xiaoxue L

AU - Yezhen L

AU - Deqiang L

AU - Shijie C

AU - Lifen J

AU - Jianlin L

AU - Jiliang H

DO - 10.1016/j.mrgentox.2009.05.015

LA - en

N1 - FEMU ID: 17178; EMF-Portal URL: https://www.emf-portal.org/en/article/17178

SP - 100-104

TI - Influence of 1.8-GHz (GSM) radiofrequency radiation (RFR) on DNA damage and repair induced by X-rays in human leukocytes in vitro

ER -

TY - JOUR

IS - 6

JA - Radiat Res

JO - Radiation Research

PY - 2009

SN - 0033-7587

VL - 171

AU - Sannino A

AU - Di Costanzo G

AU - Brescia F

AU - Sarti M

AU - Zeni O

AU - Juutilainen J

AU - Scarfi MR

DO - 10.1667/RR1642.1

LA - en

N1 - FEMU ID: 17175; EMF-Portal URL: https://www.emf-portal.org/en/article/17175

SP - 743-751

TI - Human fibroblasts and 900 MHz radiofrequency radiation: evaluation of DNA damage after exposure and co-exposure to 3-chloro-4-(dichloromethyl)-5-hydroxy-2(5h)-furanone (MX)

ER -

TY - JOUR

IS - 6

JA - Radiat Res

JO - Radiation Research

PY - 2009

SN - 0033-7587

VL - 171

AU - Sannino A

AU - Sarti M

AU - Reddy SB

AU - Prihoda TJ

AU - Vijayalaxmi

AU - Scarfi MR

DO - 10.1667/RR1687.1

LA - en

N1 - FEMU ID: 17174; EMF-Portal URL: https://www.emf-portal.org/en/article/17174

SP - 735-742

TI - Induction of adaptive response in human blood lymphocytes exposed to radiofrequency radiation

ER -

TY - JOUR

IS - 5

JA - Int J Radiat Biol

JO - International Journal of Radiation Biology

PY - 2009

SN - 0955-3002

VL - 85

AU - Ziemann C

AU - Brockmeyer H

AU - Reddy SB

AU - Vijayalaxmi

AU - Prihoda TJ

AU - Kuster N

AU - Tillmann T

AU - Dasenbrock C

DO - 10.1080/09553000902818907

LA - en

N1 - FEMU ID: 17010; EMF-Portal URL: https://www.emf-portal.org/en/article/17010

SP - 454-464

TI - Absence of genotoxic potential of 902 MHz (GSM) and 1747 MHz (DCS) wireless communication signals: In vivo two-year bioassay in B6C3F1 mice

ER -

TY - JOUR

IS - 1-2

JO - Mutation Research - Fundamental and Molecular Mechanism of Mutagenesis

PY - 2009

SN - 0027-5107

VL - 662

AU - Luukkonen J

AU - Hakulinen P

AU - Maki-Paakkanen J

AU - Juutilainen J

AU - Naarala J

DO - 10.1016/j.mrfmmm.2008.12.005

LA - en

N1 - FEMU ID: 16700; EMF-Portal URL: https://www.emf-portal.org/en/article/16700

SP - 54-58

TI - Enhancement of chemically induced reactive oxygen species production and DNA damage in human SH-SY5Y neuroblastoma cells by 872 MHz radiofrequency radiation

ER -

TY - JOUR

IS - 1

JA - Appl Biochem Biotechnol

JO - Applied Biochemistry and Biotechnology

PY - 2009

SN - 0273-2289

VL - 158

AU - Kesari KK

AU - Behari J

DO - 10.1007/s12010-008-8469-8

LA - en

N1 - FEMU ID: 16653; EMF-Portal URL: https://www.emf-portal.org/en/article/16653

SP - 126-139

TI - Fifty-gigahertz microwave exposure effect of radiations on rat brain

ER -

TY - JOUR

IS - 2

JA - Mutat Res Genet Toxicol Environ Mutagen

JO - Mutation Research - Genetic Toxicology and Environmental Mutagenesis

PY - 2009

VL - 672

AU - Tkalec M

AU - Malaric K

AU - Pavlica M

AU - Pevalek-Kozlina B

AU - Vidakovic-Cifrek Z

DO - 10.1016/j.mrgentox.2008.09.022

LA - en

N1 - FEMU ID: 16578; EMF-Portal URL: https://www.emf-portal.org/en/article/16578

SP - 76-81

TI - Effects of radiofrequency electromagnetic fields on seed germination and root meristematic cells of Allium cepa L

ER -

TY - JOUR

IS - 2

JO - Bioelectromagnetics

PY - 2009

SN - 0197-8462

VL - 30

AU - Belyaev IY

AU - Markova E

AU - Hillert L

AU - Malmgren LO

AU - Persson BR

DO - 10.1002/bem.20445

LA - en

N1 - FEMU ID: 16451; EMF-Portal URL: https://www.emf-portal.org/en/article/16451

SP - 129-141

TI - Microwaves from UMTS/GSM mobile phones induce long-lasting inhibition of 53BP1/gamma-H2AX DNA repair foci in human lymphocytes

ER -

TY - JOUR

IS - 4

JA - Fertil Steril

JO - Fertility and Sterility

PY - 2009

SN - 0015-0282

VL - 92

AU - Agarwal A

AU - Desai NR

AU - Makker K

AU - Varghese A

AU - Mouradi R

AU - Sabanegh E

AU - Sharma R

DO - 10.1016/j.fertnstert.2008.08.022

LA - en

N1 - FEMU ID: 16397; EMF-Portal URL: https://www.emf-portal.org/en/article/16397

SP - 1318-1325

TI - Effects of radiofrequency electromagnetic waves (RF-EMW) from cellular phones on human ejaculated semen: an in vitro pilot study

ER -

TY - GEN

ET - 1

PB - IEEE

PY - 2008

SN - 9781424428670

T2 - 2008 IEEE International RF and Microwave Conference, Kuala Lumpur, Malaysia

AU - Rusnani A

AU - Norhayati MN

AU - Siti Noraini S

AU - Marina M

DO - 10.1109/RFM.2008.4897448

LA - en

N1 - FEMU ID: 49987; EMF-Portal URL: https://www.emf-portal.org/en/article/49987

SP - 262-267

TI - Microwave radiation effect - a test on white mice

ER -

TY - JOUR

IS - 4

JA - Electromagn Biol Med

JO - Electromagnetic Biology and Medicine

PY - 2008

SN - 1536-8386

VL - 27

AU - Tiwari R

AU - Lakshmi NK

AU - Surender V

AU - Rajesh AD

AU - Bhargava SC

AU - Ahuja YR

DO - 10.1080/15368370802473554

LA - en

N1 - FEMU ID: 16591; EMF-Portal URL: https://www.emf-portal.org/en/article/16591

SP - 418-425

TI - Combinative Exposure Effect of Radio Frequency Signals from CDMA Mobile Phones and Aphidicolin on DNA Integrity

ER -

TY - JOUR

IS - 11

JA - Int J Radiat Biol

JO - International Journal of Radiation Biology

PY - 2008

SN - 0955-3002

VL - 84

AU - Huang TQ

AU - Lee MS

AU - Oh EH

AU - Kalinec F

AU - Zhang BT

AU - Seo JS

AU - Park WY

DO - 10.1080/09553000802460123

LA - en

N1 - FEMU ID: 16546; EMF-Portal URL: https://www.emf-portal.org/en/article/16546

SP - 909-915

TI - Characterization of biological effect of 1763 MHz radiofrequency exposure on auditory hair cells

ER -

TY - JOUR

IS - 9

JA - Int J Radiat Biol

JO - International Journal of Radiation Biology

PY - 2008

SN - 0955-3002

VL - 84

AU - Huang TQ

AU - Lee MS

AU - Oh E

AU - Zhang BT

AU - Seo JS

AU - Park WY

DO - 10.1080/09553000802317760

LA - en

N1 - FEMU ID: 16424; EMF-Portal URL: https://www.emf-portal.org/en/article/16424

SP - 734-741

TI - Molecular responses of Jurkat T-cells to 1763 MHz radiofrequency radiation

ER -

TY - JOUR

IS - 2

JA - Radiat Res

JO - Radiation Research

PY - 2008

SN - 0033-7587

VL - 170

AU - Hoyto A

AU - Luukkonen J

AU - Juutilainen J

AU - Naarala J

DO - 10.1667/RR1322.1

LA - en

N1 - FEMU ID: 16193; EMF-Portal URL: https://www.emf-portal.org/en/article/16193

SP - 235-243

TI - Proliferation, Oxidative Stress and Cell Death in Cells Exposed to 872 MHz Radiofrequency Radiation and Oxidants

ER -

TY - JOUR

IS - 8

JO - Bioelectromagnetics

PY - 2008

SN - 0197-8462

VL - 29

AU - Schrader T

AU - Münter K

AU - Kleine-Ostmann T

AU - Schmid E

DO - 10.1002/bem.20428

LA - en

N1 - FEMU ID: 16004; EMF-Portal URL: https://www.emf-portal.org/en/article/16004

SP - 626-639

TI - Spindle disturbances in human-hamster hybrid (AL) cells induced by mobile communication frequency range signals

ER -

TY - JOUR

JA - Mol Vis

JO - Molecular Vision

PY - 2008

SN - 1090-0535

VL - 14

AU - Yao K

AU - Wu W

AU - Wang K

AU - Ni S

AU - Ye P

AU - Yu Y

AU - Ye J

AU - Sun L

LA - en

N1 - FEMU ID: 15998; EMF-Portal URL: https://www.emf-portal.org/en/article/15998

SP - 964-969

TI - Electromagnetic noise inhibits radiofrequency radiation-induced DNA damage and reactive oxygen species increase in human lens epithelial cells

UR - https://www.ncbi.nlm.nih.gov/pmc/articles/PMC2391079/pdf/mv-v14-964.pdf

ER -

TY - JOUR

IS - 5

JA - Radiat Res

JO - Radiation Research

PY - 2008

SN - 0033-7587

VL - 169

AU - Manti L

AU - Braselmann H

AU - Calabrese ML

AU - Massa R

AU - Pugliese M

AU - Scampoli P

AU - Sicignano G

AU - Grossi G

DO - 10.1667/RR1044.1

LA - en

N1 - FEMU ID: 15911; EMF-Portal URL: https://www.emf-portal.org/en/article/15911

SP - 575-583

TI - Effects of modulated microwave radiation at cellular telephone frequency (1.95 GHz) on X-ray-induced chromosome aberrations in human lymphocytes in vitro

ER -

TY - JOUR

IS - 3

JA - Radiat Res

JO - Radiation Research

PY - 2008

SN - 0033-7587

VL - 169

AU - Valbonesi P

AU - Franzellitti S

AU - Piano A

AU - Contin A

AU - Biondi C

AU - Fabbri E

DO - 10.1667/RR1061.1

LA - en

N1 - FEMU ID: 15699; EMF-Portal URL: https://www.emf-portal.org/en/article/15699

SP - 270-279

TI - Evaluation of HSP70 expression and DNA damage in cells of a human trophoblast cell line exposed to 1.8 GHz amplitude-modulated radiofrequency fields

ER -

TY - JOUR

IS - 6

JA - Int Arch Occup Environ Health

JO - International Archives of Occupational and Environmental Health

PY - 2008

SN - 0340-0131

VL - 81

AU - Schwarz C

AU - Kratochvil E

AU - Pilger A

AU - Kuster N

AU - Adlkofer F

AU - Rudiger HW

DO - 10.1007/s00420-008-0305-5

LA - en

N1 - FEMU ID: 15682; EMF-Portal URL: https://www.emf-portal.org/en/article/15682

SP - 755-767

TI - Radiofrequency electromagnetic fields (UMTS, 1,950 MHz) induce genotoxic effects in vitro in human fibroblasts but not in lymphocytes

ER -

TY - JOUR

IS - 3

JA - Environ Toxicol

JO - Environmental Toxicology

PY - 2008

SN - 1520-4081

VL - 23

AU - Kim JY

AU - Hong SY

AU - Lee YM

AU - Yu SA

AU - Koh WS

AU - Hong JR

AU - Son T

AU - Chang SK

AU - Lee M

DO - 10.1002/tox.20347

LA - en

N1 - FEMU ID: 15600; EMF-Portal URL: https://www.emf-portal.org/en/article/15600

SP - 319-327

TI - In vitro assessment of clastogenicity of mobile-phone radiation (835 MHz) using the alkaline comet assay and chromosomal aberration test

ER -

TY - JOUR

IS - 2

JA - Mutat Res Genet Toxicol Environ Mutagen

JO - Mutation Research - Genetic Toxicology and Environmental Mutagenesis

PY - 2008

VL - 650

AU - Yadav AS

AU - Sharma MK

DO - 10.1016/j.mrgentox.2007.11.005

LA - en

N1 - FEMU ID: 15588; EMF-Portal URL: https://www.emf-portal.org/en/article/15588

SP - 175-180

TI - Increased frequency of micronucleated exfoliated cells among humans exposed in vivo to mobile telephone radiations

ER -

TY - JOUR

IS - 1

JA - Radiat Res

JO - Radiation Research

PY - 2008

SN - 0033-7587

VL - 169

AU - Mazor R

AU - Korenstein-Ilan A

AU - Barbul A

AU - Eshet Y

AU - Shahadi A

AU - Jerby E

AU - Korenstein R

DO - 10.1667/RR0872.1

LA - en

N1 - FEMU ID: 15530; EMF-Portal URL: https://www.emf-portal.org/en/article/15530

SP - 28-37

TI - Increased levels of numerical chromosome aberrations after in vitro exposure of human peripheral blood lymphocytes to radiofrequency electromagnetic fields for 72 hours

ER -

TY - JOUR

IS - 1

JA - Radiat Res

JO - Radiation Research

PY - 2008

SN - 0033-7587

VL - 169

AU - Joubert V

AU - Bourthoumieu S

AU - Leveque P

AU - Yardin C

DO - 10.1667/RR1077.1

LA - en

N1 - FEMU ID: 15528; EMF-Portal URL: https://www.emf-portal.org/en/article/15528

SP - 38-45

TI - Apoptosis is Induced by Radiofrequency Fields through the Caspase-Independent Mitochondrial Pathway in Cortical Neurons

ER -

TY - JOUR

IS - 3

JO - Bioelectromagnetics

PY - 2008

SN - 0197-8462

VL - 29

AU - Zeni O

AU - Schiavoni A

AU - Perrotta A

AU - Forigo D

AU - Deplano M

AU - Scarfi MR

DO - 10.1002/bem.20378

LA - en

N1 - FEMU ID: 15373; EMF-Portal URL: https://www.emf-portal.org/en/article/15373

SP - 177-184

TI - Evaluation of genotoxic effects in human leukocytes after in vitro exposure to 1950 MHz UMTS radiofrequency field

ER -

TY - GEN

ET - 1

PB - IEEE

PY - 2007

SN - 9781424413720

T2 - 2007 International Symposium on Electromagnetic Compatibility

AU - Ren DQ

AU - Yang WQ

AU - Zhao T

AU - Li XJ

AU - Zeng GY

AU - Zhang J

AU - Li YR

DO - 10.1109/ELMAGC.2007.4413535

LA - en

N1 - FEMU ID: 49696; EMF-Portal URL: https://www.emf-portal.org/en/article/49696

SP - 479-481

TI - Changes of microwave radiation on the level of trace elements in mice bone tissues

ER -

TY - JOUR

IS - 4

JA - Int J Radiat Biol

JO - International Journal of Radiation Biology

PY - 2007

SN - 0955-3002

VL - 83

AU - Juutilainen J

AU - Heikkinen P

AU - Soikkeli H

AU - Maki-Paakkanen J

DO - 10.1080/09553000601169800

LA - en

N1 - FEMU ID: 14790; EMF-Portal URL: https://www.emf-portal.org/en/article/14790

SP - 213-220

TI - Micronucleus frequency in erythrocytes of mice after long-term exposure to radiofrequency radiation

ER -

TY - JOUR

IS - 4

JA - Health Phys

JO - Health Physics

PY - 2007

SN - 0017-9078

VL - 92

AU - Zeni O

AU - Gallerano GP

AU - Perrotta A

AU - Romano M

AU - Sannino A

AU - Sarti M

AU - D'Arienzo M

AU - Doria A

AU - Giovenale E

AU - Lai A

AU - Messina G

AU - Scarfi MR

DO - 10.1097/01.HP.0000251248.23991.35

LA - en

N1 - FEMU ID: 14603; EMF-Portal URL: https://www.emf-portal.org/en/article/14603

SP - 349-357

TI - Cytogenetic Observations In Human Peripheral Blood Leukocytes Following In Vitro Exposure To THz Radiation: A Pilot Study

ER -

TY - JOUR

IS - 3

JO - Toxicology

PY - 2007

SN - 0300-483X

VL - 232

AU - Baohong W

AU - Lifen J

AU - Lanjuan L

AU - Jianlin L

AU - Deqiang L

AU - Wei Z

AU - Jiliang H

DO - 10.1016/j.tox.2007.01.019

LA - en

N1 - FEMU ID: 14600; EMF-Portal URL: https://www.emf-portal.org/en/article/14600

SP - 311-316

TI - Evaluating the combinative effects on human lymphocyte DNA damage induced by ultraviolet ray C plus 1.8 GHz microwaves using comet assay in vitro

ER -

TY - JOUR

IS - 1

JA - J Radiat Res

JO - Journal of Radiation Research

PY - 2007

SN - 0449-3060

VL - 48

AU - Koyama S

AU - Takashima Y

AU - Sakurai T

AU - Suzuki Y

AU - Taki M

AU - Miyakoshi J

DO - 10.1269/jrr.06085

LA - en

N1 - FEMU ID: 14407; EMF-Portal URL: https://www.emf-portal.org/en/article/14407

SP - 69-75

TI - Effects of 2.45 GHz electromagnetic fields with a wide range of SARs on bacterial and HPRT gene mutations

UR - https://www.jstage.jst.go.jp/article/jrr/48/1/48_1_69/_article

ER -

TY - JOUR

IS - 1-2

JA - Mutat Res Genet Toxicol Environ Mutagen

JO - Mutation Research - Genetic Toxicology and Environmental Mutagenesis

PY - 2007

VL - 626

AU - Panagopoulos DJ

AU - Chavdoula ED

AU - Nezis IP

AU - Margaritis LH

DO - 10.1016/j.mrgentox.2006.08.008

LA - en

N1 - FEMU ID: 14278; EMF-Portal URL: https://www.emf-portal.org/en/article/14278

SP - 69-78

TI - Cell death induced by GSM 900-MHz and DCS 1800-MHz mobile telephony radiation

ER -

TY - JOUR

IS - 1-2

JA - Mutat Res Genet Toxicol Environ Mutagen

JO - Mutation Research - Genetic Toxicology and Environmental Mutagenesis

PY - 2007

VL - 626

AU - Speit G

AU - Schütz P

AU - Hoffmann H

DO - 10.1016/j.mrgentox.2006.08.003

LA - en

N1 - FEMU ID: 14202; EMF-Portal URL: https://www.emf-portal.org/en/article/14202

SP - 42-47

TI - Genotoxic effects of exposure to radiofrequency electromagnetic fields (RF-EMF) in cultured mammalian cells are not independently reproducible

ER -

TY - JOUR

IS - 1-2

JO - Mutation Research - Fundamental and Molecular Mechanism of Mutagenesis

PY - 2006

SN - 0027-5107

VL - 596

AU - Paulraj R

AU - Behari J

DO - 10.1016/j.mrfmmm.2005.12.006

LA - en

N1 - FEMU ID: 18097; EMF-Portal URL: https://www.emf-portal.org/en/article/18097

SP - 76-80

TI - Single strand DNA breaks in rat brain cells exposed to microwave radiation

ER -

TY - JOUR

IS - 6

JA - IEEE Trans Microw Theory Tech

JO - IEEE Transactions on Microwave Theory and Techniques

PY - 2006

SN - 0018-9480

VL - 54

AU - Zhadobov M

AU - Sauleau R

AU - Vie V

AU - Himd M

AU - Le Coq L

AU - Thouroude D

DO - 10.1109/TMTT.2006.875811

LA - en

N1 - FEMU ID: 16235; EMF-Portal URL: https://www.emf-portal.org/en/article/16235

SP - 2534-2542

TI - Interactions between 60-GHz millimeter waves and artificial biological membranes: dependence on radiation parameters

ER -

TY - JOUR

IS - 3

JA - Exp Toxicol Pathol

JO - Experimental and Toxicologic Pathology

PY - 2006

SN - 0940-2993

VL - 57

AU - Trosic I

AU - Busljeta I

DO - 10.1016/j.etp.2005.08.002

LA - en

N1 - FEMU ID: 15461; EMF-Portal URL: https://www.emf-portal.org/en/article/15461

SP - 247-251

TI - Erythropoietic dynamic equilibrium in rats maintained after microwave irradiation

ER -

TY - JOUR

IS - 4

JA - IEEE Trans Plasma Sci

JO - IEEE Transactions on Plasma Science

PY - 2006

SN - 0093-3813

VL - 34

AU - Sannino A

AU - Calabrese ML

AU - d'Ambrosio G

AU - Massa R

AU - Petraglia G

AU - Mita P

AU - Sarti M

AU - Scarfi MR

DO - 10.1109/TPS.2006.878379

LA - en

N1 - FEMU ID: 15411; EMF-Portal URL: https://www.emf-portal.org/en/article/15411

SP - 1441-1448

TI - Evaluation of Cytotoxic and Genotoxic Effects in Human Peripheral Blood Leukocytes Following Exposure to 1950-MHz Modulated Signal

ER -

TY - JOUR

IS - 1-2

JO - Mutation Research - Fundamental and Molecular Mechanism of Mutagenesis

PY - 2006

SN - 0027-5107

VL - 602

AU - Lixia S

AU - Yao K

AU - Kaijun W

AU - Deqiang L

AU - Huajun H

AU - Xiangwei G

AU - Baohong W

AU - Wei Z

AU - Jianling L

AU - Wei W

DO - 10.1016/j.mrfmmm.2006.08.010

LA - en

N1 - FEMU ID: 14262; EMF-Portal URL: https://www.emf-portal.org/en/article/14262

SP - 135-142

TI - Effects of 1.8 GHz radiofrequency field on DNA damage and expression of heat shock protein 70 in human lens epithelial cells

ER -

TY - JOUR

IS - 1

JA - Life Sci

JO - Life Sciences

PY - 2006

SN - 0024-3205

VL - 80

AU - Ferreira AR

AU - Knakievicz T

AU - Pasquali MA

AU - Gelain DP

AU - Dal-Pizzol F

AU - Fernandez CE

AU - de Salles AA

AU - Ferreira HB

AU - Moreira JC

DO - 10.1016/j.lfs.2006.08.018

LA - en

N1 - FEMU ID: 14184; EMF-Portal URL: https://www.emf-portal.org/en/article/14184

SP - 43-50

TI - Ultra high frequency-electromagnetic field irradiation during pregnancy leads to an increase in erythrocytes micronuclei incidence in rat offspring

ER -

TY - JOUR

IS - 3

JA - Radiat Res

JO - Radiation Research

PY - 2006

SN - 0033-7587

VL - 166

AU - Vijayalaxmi

DO - 10.1667/RR0643.1

LA - en

N1 - FEMU ID: 14138; EMF-Portal URL: https://www.emf-portal.org/en/article/14138

SP - 532-538

TI - Cytogenetic studies in human blood lymphocytes exposed in vitro to 2.45 GHz or 8.2 GHz radiofrequency radiation

ER -

TY - JOUR

IS - 5

JA - Int J Radiat Biol

JO - International Journal of Radiation Biology

PY - 2006

SN - 0955-3002

VL - 82

AU - Stronati L

AU - Testa A

AU - Moquet J

AU - Edwards A

AU - Cordelli E

AU - Villani P

AU - Marino C

AU - Fresegna AM

AU - Appolloni M

AU - Lloyd D

DO - 10.1080/09553000600739173

LA - en

N1 - FEMU ID: 13927; EMF-Portal URL: https://www.emf-portal.org/en/article/13927

SP - 339-346

TI - 935 MHz cellular phone radiation. An in vitro study of genotoxicity in human lymphocytes

ER -

TY - JOUR

IS - 6

JA - Radiat Res

JO - Radiation Research

PY - 2006

SN - 0033-7587

VL - 165

AU - Scarfi MR

AU - Fresegna AM

AU - Villani P

AU - Pinto R

AU - Marino C

AU - Sarti M

AU - Altavista P

AU - Sannino A

AU - Lovisolo GA

DO - 10.1667/RR3570.1

LA - en

N1 - FEMU ID: 13901; EMF-Portal URL: https://www.emf-portal.org/en/article/13901

SP - 655-663

TI - Exposure to radiofrequency radiation (900 MHz, GSM signal) does not affect micronucleus frequency and cell proliferation in human peripheral blood lymphocytes: an interlaboratory study

ER -

TY - JOUR

IS - 6

JO - Bioelectromagnetics

PY - 2006

SN - 0197-8462

VL - 27

AU - Hirose H

AU - Sakuma N

AU - Kaji N

AU - Suhara T

AU - Sekijima M

AU - Nojima T

AU - Miyakoshi J

DO - 10.1002/bem.20238

LA - en

N1 - FEMU ID: 13863; EMF-Portal URL: https://www.emf-portal.org/en/article/13863

SP - 494-504

TI - Phosphorylation and gene expression of p53 are not affected in human cells exposed to 2.1425 GHz band CW or W-CDMA modulated radiation allocated to mobile radio base stations

ER -

TY - JOUR

IS - 5

JA - Radiat Res

JO - Radiation Research

PY - 2006

SN - 0033-7587

VL - 165

AU - Verschaeve L

AU - Heikkinen P

AU - Verheyen G

AU - Van Gorp U

AU - Boonen F

AU - Vander Plaetse F

AU - Maes A

AU - Kumlin T

AU - Maki-Paakkanen J

AU - Puranen L

AU - Juutilainen J

DO - 10.1667/RR3559.1

LA - en

N1 - FEMU ID: 13792; EMF-Portal URL: https://www.emf-portal.org/en/article/13792

SP - 598-607

TI - Investigation of co-genotoxic effects of radiofrequency electromagnetic fields in vivo

ER -

TY - JOUR

IS - 4

JO - Bioelectromagnetics

PY - 2006

SN - 0197-8462

VL - 27

AU - Belyaev IY

AU - Koch CB

AU - Terenius O

AU - Röxstrom-Lindquist K

AU - Malmgren LO

AU - Sommer WH

AU - Salford LG

AU - Persson BR

DO - 10.1002/bem.20216

LA - en

N1 - FEMU ID: 13430; EMF-Portal URL: https://www.emf-portal.org/en/article/13430

SP - 295-306

TI - Exposure of rat brain to 915 MHz GSM microwaves induces changes in gene expression but not double stranded DNA breaks or effects on chromatin conformation

ER -

TY - JOUR

IS - 2

JO - Mutagenesis

PY - 2006

SN - 0267-8357

VL - 21

AU - Maes A

AU - Van Gorp U

AU - Verschaeve L

DO - 10.1093/mutage/gel008

LA - en

N1 - FEMU ID: 13387; EMF-Portal URL: https://www.emf-portal.org/en/article/13387

SP - 139-142

TI - Cytogenetic investigation of subjects professionally exposed to radiofrequency radiation

UR - https://academic.oup.com/mutage/article-pdf/21/2/139/3906515/gel008.pdf

ER -

TY - JOUR

IS - 3

JO - Bioelectromagnetics

PY - 2006

SN - 0197-8462

VL - 27

AU - Chemeris NK

AU - Gapeyev AB

AU - Sirota NP

AU - Gudkova OY

AU - Tankanag AV

AU - Konovalov IV

AU - Buzoverya ME

AU - Suvorov VG

AU - Logunov VA

DO - 10.1002/bem.20196

LA - en

N1 - FEMU ID: 12886; EMF-Portal URL: https://www.emf-portal.org/en/article/12886

SP - 197-203

TI - Lack of direct DNA damage in human blood leukocytes and lymphocytes after in vitro exposure to high power microwave pulses

ER -

TY - JOUR

IS - 1

JO - Bioelectromagnetics

PY - 2006

SN - 0197-8462

VL - 27

AU - Sakuma N

AU - Komatsubara Y

AU - Takeda H

AU - Hirose H

AU - Sekijima M

AU - Nojima T

AU - Miyakoshi J

DO - 10.1002/bem.20179

LA - en

N1 - FEMU ID: 12875; EMF-Portal URL: https://www.emf-portal.org/en/article/12875

SP - 51-57

TI - DNA strand breaks are not induced in human cells exposed to 2.1425 GHz band CW and W-CDMA modulated radiofrequency fields allocated to mobile radio base stations

ER -

TY - JOUR

JA - Phys Scr

JO - Physica Scripta

PY - 2005

SN - 0031-8949

VL - 118

AU - Trosic I

AU - Busljeta I

DO - 10.1238/Physica.Topical.118a00168

LA - en

N1 - FEMU ID: 19665; EMF-Portal URL: https://www.emf-portal.org/en/article/19665

SP - 168-170

TI - Frequency of micronucleated erythrocytes in rat bone marrow exposed to 2.45 GHz radiation

ER -

TY - JOUR

IS - 4

JA - Int J Hum Genet

JO - International Journal of Human Genetics

PY - 2005

SN - 0972-3757

VL - 5

AU - Gandhi G

AU - Singh P

LA - en

N1 - FEMU ID: 16802; EMF-Portal URL: https://www.emf-portal.org/en/article/16802

SP - 259-265

TI - Cytogenetic damage in mobile phone users: preliminary data

UR - http://www.krepublishers.com/02-Journals/IJHG/IJHG-05-0-000-000-2005-Web/IJHG-05-4-225-288-2005-Abst-PDF/IJHG-05-4-259-265-2005-210-Gandhi-G/IJHG-05-4-259-265-2005-210-Gandhi-G.pdf

ER -

TY - JOUR

IS - 2

JA - Indian J Hum Genet

JO - Indian Journal of Human Genetics

PY - 2005

SN - 1998-362X

VL - 11

AU - Gandhi G

AU - Anita

DO - 10.4103/0971-6866.16810

LA - en

N1 - FEMU ID: 16801; EMF-Portal URL: https://www.emf-portal.org/en/article/16801

SP - 99-104

TI - Genetic damage in mobile phone users: some preliminary findings

UR - http://www.bioline.org.br/pdf?hg05022

ER -

TY - JOUR

IS - 3

JA - Electromagn Biol Med

JO - Electromagnetic Biology and Medicine

PY - 2005

SN - 1536-8386

VL - 24

AU - Vojtisek M

AU - Knotkova J

AU - Kasparova L

AU - Hornychova M

AU - Frantik M

AU - Svandova E

DO - 10.1080/15368370500381695

LA - en

N1 - FEMU ID: 13487; EMF-Portal URL: https://www.emf-portal.org/en/article/13487

SP - 355-358

TI - Potential Impact of Simulated Mobile Phone Radiation on Blood-Brain Barrier

ER -

TY - JOUR

IS - 1-2

JA - Mutat Res Genet Toxicol Environ Mutagen

JO - Mutation Research - Genetic Toxicology and Environmental Mutagenesis

PY - 2005

VL - 587

AU - Komatsubara Y

AU - Hirose H

AU - Sakurai T

AU - Koyama S

AU - Suzuki Y

AU - Taki M

AU - Miyakoshi J

DO - 10.1016/j.mrgentox.2005.08.010

LA - en

N1 - FEMU ID: 12641; EMF-Portal URL: https://www.emf-portal.org/en/article/12641

SP - 114-119

TI - Effect of high-frequency electromagnetic fields with a wide range of SARs on chromosomal aberrations in murine m5S cells

ER -

TY - JOUR

IS - 4

JA - Radiat Res

JO - Radiation Research

PY - 2005

SN - 0033-7587

VL - 164

AU - Gorlitz BD

AU - Muller M

AU - Ebert S

AU - Hecker H

AU - Kuster N

AU - Dasenbrock C

DO - 10.1667/rr3440.1

LA - en

N1 - FEMU ID: 12605; EMF-Portal URL: https://www.emf-portal.org/en/article/12605

SP - 431-439

TI - Effects of 1-week and 6-week exposure to GSM/DCS radiofrequency radiation on micronucleus formation in B6C3F1 mice

ER -

TY - JOUR

IS - 9

JA - Environ Health Perspect

JO - Environmental Health Perspectives

PY - 2005

SN - 0091-6765

VL - 113

AU - Markova E

AU - Hillert L

AU - Malmgren L

AU - Persson BR

AU - Belyaev IY

DO - 10.1289/ehp.7561

LA - en

N1 - FEMU ID: 12441; EMF-Portal URL: https://www.emf-portal.org/en/article/12441

SP - 1172-1177

TI - Microwaves from GSM mobile telephones affect 53BP1 and gamma-H2AX foci in human lymphocytes from hypersensitive and healthy persons

UR - https://www.ncbi.nlm.nih.gov/pmc/articles/PMC1280397/pdf/ehp0113-001172.pdf

ER -

TY - JOUR

IS - 12

JA - FASEB J

JO - The FASEB Journal

PY - 2005

SN - 0892-6638

VL - 19

AU - Nikolova T

AU - Czyz J

AU - Rolletschek A

AU - Blyszczuk P

AU - Fuchs J

AU - Jovtchev G

AU - Schuderer J

AU - Kuster N

AU - Wobus AM

DO - 10.1096/fj.04-3549fje

LA - en

N1 - FEMU ID: 12365; EMF-Portal URL: https://www.emf-portal.org/en/article/12365

SP - 1686-1688

TI - Electromagnetic fields affect transcript levels of apoptosis-related genes in embryonic stem cell-derived neural progenitor cells

ER -

TY - JOUR

IS - 1-2

JA - Mutat Res Genet Toxicol Environ Mutagen

JO - Mutation Research - Genetic Toxicology and Environmental Mutagenesis

PY - 2005

VL - 582

AU - Zotti-Martelli L

AU - Peccatori M

AU - Maggini V

AU - Ballardin M

AU - Barale R

DO - 10.1016/j.mrgentox.2004.12.014

LA - en

N1 - FEMU ID: 12152; EMF-Portal URL: https://www.emf-portal.org/en/article/12152

SP - 42-52

TI - Individual responsiveness to induction of micronuclei in human lymphocytes after exposure in vitro to 1800 MHz microwave radiation

ER -

TY - JOUR

IS - 1

JA - Electromagn Biol Med

JO - Electromagnetic Biology and Medicine

PY - 2005

SN - 1536-8386

VL - 24

AU - Lai H

AU - Singh NP

LA - en

N1 - FEMU ID: 12077; EMF-Portal URL: https://www.emf-portal.org/en/article/12077

SP - 23-29

TI - Interaction of Microwaves and a Temporally Incoherent Magnetic Field on Single and Double DNA Strand Breaks in Rat Brain Cells

ER -

TY - JOUR

IS - 1-2

JO - Mutation Research - Fundamental and Molecular Mechanism of Mutagenesis

PY - 2005

SN - 0027-5107

VL - 578

AU - Baohong W

AU - Jiliang H

AU - Lifen J

AU - Deqiang L

AU - Wei Z

AU - Jianlin L

AU - Hongping D

DO - 10.1016/j.mrfmmm.2005.05.001

LA - en

N1 - FEMU ID: 12063; EMF-Portal URL: https://www.emf-portal.org/en/article/12063

SP - 149-157

TI - Studying the synergistic damage effects induced by 1.8 GHz radiofrequency field radiation (RFR) with four chemical mutagens on human lymphocyte DNA using comet assay in vitro

ER -

TY - JOUR

IS - 3

JA - Int J Androl

JO - International Journal of Andrology

PY - 2005

SN - 0105-6263

VL - 28

AU - Aitken RJ

AU - Bennetts LE

AU - Sawyer D

AU - Wiklendt AM

AU - King BV

DO - 10.1111/j.1365-2605.2005.00531.x

LA - en

N1 - FEMU ID: 11992; EMF-Portal URL: https://www.emf-portal.org/en/article/11992

SP - 171-179

TI - Impact of radio frequency electromagnetic radiation on DNA integrity in the male germline

ER -

TY - JOUR

IS - 2

JA - Mutat Res Genet Toxicol Environ Mutagen

JO - Mutation Research - Genetic Toxicology and Environmental Mutagenesis

PY - 2005

VL - 583

AU - Diem E

AU - Schwarz C

AU - Adlkofer F

AU - Jahn O

AU - Rüdiger H

DO - 10.1016/j.mrgentox.2005.03.006

LA - en

N1 - FEMU ID: 11910; EMF-Portal URL: https://www.emf-portal.org/en/article/11910

SP - 178-183

TI - Non-thermal DNA breakage by mobile-phone radiation (1800 MHz) in human fibroblasts and in transformed GFSH-R17 rat granulosa cells in vitro

ER -

TY - JOUR

IS - 4

JO - Bioelectromagnetics

PY - 2005

SN - 0197-8462

VL - 26

AU - Zeni O

AU - Romano M

AU - Perrotta A

AU - Lioi MB

AU - Barbieri R

AU - d'Ambrosio G

AU - Massa R

AU - Scarfi MR

DO - 10.1002/bem.20078

LA - en

N1 - FEMU ID: 11836; EMF-Portal URL: https://www.emf-portal.org/en/article/11836

SP - 258-265

TI - Evaluation of genotoxic effects in human peripheral blood leukocytes following an acute in vitro exposure to 900 MHz radiofrequency fields

ER -

TY - JOUR

IS - 2

JA - Eur J Cancer Prev

JO - European Journal of Cancer Prevention

PY - 2005

SN - 0959-8278

VL - 14

AU - Chang SK

AU - Choi JS

AU - Gil HW

AU - Yang JO

AU - Lee EY

AU - Jeon YS

AU - Lee ZW

AU - Lee M

AU - Hong MY

AU - Ho Son T

AU - Hong SY

DO - 10.1097/00008469-200504000-00014

LA - en

N1 - FEMU ID: 11767; EMF-Portal URL: https://www.emf-portal.org/en/article/11767

SP - 175-179

TI - Genotoxicity evaluation of electromagnetic fields generated by 835-MHz mobile phone frequency band

ER -

TY - JOUR

IS - 3

JO - Bioelectromagnetics

PY - 2005

SN - 0197-8462

VL - 26

AU - Belyaev IY

AU - Hillert L

AU - Protopopova M

AU - Tamm C

AU - Malmgren LO

AU - Persson BR

AU - Selivanova G

AU - Harms-Ringdahl M

DO - 10.1002/bem.20103

LA - en

N1 - FEMU ID: 11713; EMF-Portal URL: https://www.emf-portal.org/en/article/11713

SP - 173-184

TI - 915 MHz microwaves and 50 Hz magnetic field affect chromatin conformation and 53BP1 foci in human lymphocytes from hypersensitive and healthy persons

ER -

TY - JOUR

IS - 2

JA - J Cell Physiol

JO - Journal of Cellular Physiology

PY - 2005

SN - 0021-9541

VL - 204

AU - Caraglia M

AU - Marra M

AU - Mancinelli F

AU - d'Ambrosio G

AU - Massa R

AU - Giordano A

AU - Budillon A

AU - Abbruzzese A

AU - Bismuto E

DO - 10.1002/jcp.20327

LA - en

N1 - FEMU ID: 11646; EMF-Portal URL: https://www.emf-portal.org/en/article/11646

SP - 539-548

TI - Electromagnetic fields at mobile phone frequency induce apoptosis and inactivation of the multi-chaperone complex in human epidermoid cancer cells

ER -

TY - JOUR

IS - 4

JA - Oncol Rep

JO - Oncology Reports

PY - 2004

SN - 1021-335X

VL - 11

AU - Maeda K

AU - Maeda T

AU - Qi Y

DO - 10.3892/or.11.4.771

LA - en

N1 - FEMU ID: 14042; EMF-Portal URL: https://www.emf-portal.org/en/article/14042

SP - 771-775

TI - In vitro and in vivo induction of human LoVo cells into apoptotic process by non-invasive microwave treatment: a potentially novel approach for physical therapy of human colorectal cancer

ER -

TY - JOUR

IS - 3

JA - Genet Mol Biol

JO - Genetics and Molecular Biology

PY - 2004

SN - 1415-4757

VL - 27

AU - Figueiredo ABS

AU - Alves RN

AU - Ramalho AT

LA - en

N1 - FEMU ID: 14002; EMF-Portal URL: https://www.emf-portal.org/en/article/14002

SP - 460-466

TI - Cytogenetic analysis of the effects of 2.5 and 10.5 GHz microwaves on human lymphocytes

UR - http://www.scielo.br/pdf/gmb/v27n3/a24v27n3.pdf

ER -

TY - JOUR

IS - 6

JA - Int J Hyg Environ Health

JO - International Journal of Hygiene and Environmental Health

PY - 2004

SN - 1438-4639

VL - 207

AU - Busljeta I

AU - Trosic I

AU - Milkovic-Kraus S

DO - 10.1078/1438-4639-00326

LA - en

N1 - FEMU ID: 11630; EMF-Portal URL: https://www.emf-portal.org/en/article/11630

SP - 549-554

TI - Erythropoietic changes in rats after 2.45 GHz nonthermal irradiation

ER -

TY - JOUR

IS - 5

JO - Mutagenesis

PY - 2004

SN - 0267-8357

VL - 19

AU - Trosic I

AU - Busljeta I

AU - Modlic B

DO - 10.1093/mutage/geh042

LA - en

N1 - FEMU ID: 11628; EMF-Portal URL: https://www.emf-portal.org/en/article/11628

SP - 361-364

TI - Investigation of the genotoxic effect of microwave irradiation in rat bone marrow cells: in vivo exposure

UR - https://academic.oup.com/mutage/article-pdf/19/5/361/4067612/geh042.pdf

ER -

TY - JOUR

JA - ScientificWorldJournal

JO - The Scientific World Journal

PY - 2004

SN - 1537-744X

VL - 4

AU - Demsia G

AU - Vlastos D

AU - Matthopoulos DP

DO - 10.1100/tsw.2004.178

LA - en

N1 - FEMU ID: 11395; EMF-Portal URL: https://www.emf-portal.org/en/article/11395

SP - 48-54

TI - Effect of 910-MHz electromagnetic field on rat bone marrow

UR - https://www.hindawi.com/journals/tswj/2004/591712/abs/

ER -

TY - JOUR

JA - ScientificWorldJournal

JO - The Scientific World Journal

PY - 2004

SN - 1537-744X

VL - 4

AU - Koyama S

AU - Isozumi Y

AU - Suzuki Y

AU - Taki M

AU - Miyakoshi J

DO - 10.1100/tsw.2004.176

LA - en

N1 - FEMU ID: 11393; EMF-Portal URL: https://www.emf-portal.org/en/article/11393

SP - 29-40

TI - Effects of 2.45-GHz electromagnetic fields with a wide range of SARs on micronucleus formation in CHO-K1 cells

UR - http://downloads.hindawi.com/journals/tswj/2004/743762.pdf

ER -

TY - JOUR

IS - 1

JA - Int J Radiat Biol

JO - International Journal of Radiation Biology

PY - 2004

SN - 0955-3002

VL - 80

AU - Lagroye I

AU - Anane R

AU - Wettring BA

AU - Moros EG

AU - Straube WL

AU - LaRegina MC

AU - Niehoff M

AU - Pickard WF

AU - Baty J

AU - Roti Roti JL

DO - 10.1080/09553000310001642911

LA - en

N1 - FEMU ID: 11161; EMF-Portal URL: https://www.emf-portal.org/en/article/11161

SP - 11-20

TI - Measurement of DNA damage after acute exposure to pulsed-wave 2450 MHz microwaves in rat brain cells by two alkaline comet assay methods

ER -

TY - JOUR

IS - 1-2

JA - Mutat Res Genet Toxicol Environ Mutagen

JO - Mutation Research - Genetic Toxicology and Environmental Mutagenesis

PY - 2004

VL - 558

AU - Chemeris NK

AU - Gapeyev AB

AU - Sirota NP

AU - Gudkova OY

AU - Kornienko NV

AU - Tankanag AV

AU - Konovalov IV

AU - Buzoverya ME

AU - Suvorov VG

AU - Logunov VA

DO - 10.1016/j.mrgentox.2003.10.017

LA - en

N1 - FEMU ID: 10650; EMF-Portal URL: https://www.emf-portal.org/en/article/10650

SP - 27-34

TI - DNA damage in frog erythrocytes after in vitro exposure to a high peak-power pulsed electromagnetic field

ER -

TY - JOUR

IS - 2

JA - Radiat Res

JO - Radiation Research

PY - 2004

SN - 0033-7587

VL - 161

AU - Hook GJ

AU - Zhang P

AU - Lagroye I

AU - Li L

AU - Higashikubo R

AU - Moros EG

AU - Straube WL

AU - Pickard WF

AU - Baty JD

AU - Roti Roti JL

DO - 10.1667/rr3127

LA - en

N1 - FEMU ID: 10635; EMF-Portal URL: https://www.emf-portal.org/en/article/10635

SP - 193-200

TI - Measurement of DNA damage and apoptosis in Molt-4 cells after in vitro exposure to radiofrequency radiation

ER -

TY - JOUR

IS - 2

JA - Radiat Res

JO - Radiation Research

PY - 2004

SN - 0033-7587

VL - 161

AU - Lagroye I

AU - Hook GJ

AU - Wettring BA

AU - Baty JD

AU - Moros EG

AU - Straube WL

AU - Roti Roti JL

DO - 10.1667/rr3122

LA - en

N1 - FEMU ID: 10634; EMF-Portal URL: https://www.emf-portal.org/en/article/10634

SP - 201-214

TI - Measurements of alkali-labile DNA damage and protein-DNA crosslinks after 2450 MHz microwave and low-dose gamma irradiation in vitro

ER -

TY - JOUR

IS - 3

JO - Bioelectromagnetics

PY - 2004

SN - 0197-8462

VL - 25

AU - Pyrpasopoulou A

AU - Kotoula V

AU - Cheva A

AU - Hytiroglou P

AU - Nikolakaki E

AU - Magras IN

AU - Xenos TD

AU - Tsiboukis TD

AU - Karkavelas G

DO - 10.1002/bem.10185

LA - en

N1 - FEMU ID: 10631; EMF-Portal URL: https://www.emf-portal.org/en/article/10631

SP - 216-227

TI - Bone morphogenetic protein expression in newborn rat kidneys after prenatal exposure to radiofrequency radiation

ER -

TY - JOUR

IS - 3

JA - Radiat Res

JO - Radiation Research

PY - 2004

SN - 0033-7587

VL - 161

AU - Vijayalaxmi

AU - Logani MK

AU - Bhanushali A

AU - Ziskin MC

AU - Prihoda TJ

DO - 10.1667/rr3121

LA - en

N1 - FEMU ID: 10560; EMF-Portal URL: https://www.emf-portal.org/en/article/10560

SP - 341-345

TI - Micronuclei in peripheral blood and bone marrow cells of mice exposed to 42 GHz electromagnetic millimeter waves

ER -

TY - JOUR

IS - 2

JA - J Cell Physiol

JO - Journal of Cellular Physiology

PY - 2004

SN - 0021-9541

VL - 198

AU - Marinelli F

AU - La Sala D

AU - Cicciotti G

AU - Cattini L

AU - Trimarchi C

AU - Putti S

AU - Zamparelli A

AU - Giuliani L

AU - Tomassetti G

AU - Cinti C

DO - 10.1002/jcp.10425

LA - en

N1 - FEMU ID: 10381; EMF-Portal URL: https://www.emf-portal.org/en/article/10381

SP - 324-332

TI - Exposure to 900 MHz electromagnetic field induces an unbalance between pro-apoptotic and pro-survival signals in T-lymphoblastoid leukemia CCRF-CEM cells

ER -

TY - JOUR

IS - 2-3

JA - J Biol Phys

JO - Journal of Biological Physics

PY - 2003

SN - 0092-0606

VL - 29

AU - Scarfi MR

AU - Romano M

AU - Di Pietro R

AU - Zeni O

AU - Doria A

AU - Gallerano GP

AU - Giovenale E

AU - Messina G

AU - Lai A

AU - Campurra G

AU - Coniglio D

AU - Arienzo D

DO - 10.1023/A:1024440708943

LA - en

N1 - FEMU ID: 14927; EMF-Portal URL: https://www.emf-portal.org/en/article/14927

SP - 171-177

TI - THz Exposure of Whole Blood for the Study of Biological Effects on Human Lymphocytes

UR - https://www.ncbi.nlm.nih.gov/pmc/articles/PMC3456423/pdf/10867_2004_Article_5121580.pdf

ER -

TY - JOUR

IS - 9

JA - Int J Radiat Biol

JO - International Journal of Radiation Biology

PY - 2003

SN - 0955-3002

VL - 79

AU - Port M

AU - Abend M

AU - Romer B

AU - Van Beuningen D

DO - 10.1080/09553000310001606803

LA - en

N1 - FEMU ID: 10792; EMF-Portal URL: https://www.emf-portal.org/en/article/10792

SP - 701-708

TI - Influence of high-frequency electromagnetic fields on different modes of cell death and gene expression

ER -

TY - JOUR

IS - 2-3

JA - Electromagn Biol Med

JO - Electromagnetic Biology and Medicine

PY - 2003

SN - 1536-8386

VL - 22

AU - Gadhia PK

AU - Shah T

AU - Mistry A

AU - Pithawala M

AU - Tamakuvala D

DO - 10.1081/JBC-120024624

LA - en

N1 - FEMU ID: 10674; EMF-Portal URL: https://www.emf-portal.org/en/article/10674

SP - 149-159

TI - A Preliminary Study to Assess Possible Chromosomal Damage Among Users of Digital Mobile Phones

ER -

TY - JOUR

IS - 1-2

JA - Mutat Res Genet Toxicol Environ Mutagen

JO - Mutation Research - Genetic Toxicology and Environmental Mutagenesis

PY - 2003

VL - 542

AU - Stacey M

AU - Stickley J

AU - Fox P

AU - Statler V

AU - Schoenbach K

AU - Beebe SJ

AU - Buescher S

DO - 10.1016/j.mrgentox.2003.08.006

LA - en

N1 - FEMU ID: 10503; EMF-Portal URL: https://www.emf-portal.org/en/article/10503

SP - 65-75

TI - Differential effects in cells exposed to ultra-short, high intensity electric fields: cell survival, DNA damage, and cell cycle analysis

ER -

TY - JOUR

IS - 12

JA - DNA Cell Biol

JO - DNA and Cell Biology

PY - 2003

SN - 1044-5498

VL - 22

AU - Beebe SJ

AU - White J

AU - Blackmore PF

AU - Deng Y

AU - Somers K

AU - Schoenbach KH

DO - 10.1089/104454903322624993

LA - en

N1 - FEMU ID: 10502; EMF-Portal URL: https://www.emf-portal.org/en/article/10502

SP - 785-796

TI - Diverse effects of nanosecond pulsed electric fields on cells and tissues

ER -

TY - JOUR

IS - 1-2

JA - Mutat Res Genet Toxicol Environ Mutagen

JO - Mutation Research - Genetic Toxicology and Environmental Mutagenesis

PY - 2003

VL - 541

AU - Koyama S

AU - Nakahara T

AU - Wake K

AU - Taki M

AU - Isozumi Y

AU - Miyakoshi J

DO - 10.1016/j.mrgentox.2003.07.009

LA - en

N1 - FEMU ID: 10341; EMF-Portal URL: https://www.emf-portal.org/en/article/10341

SP - 81-89

TI - Effects of high frequency electromagnetic fields on micronucleus formation in CHO-K1 cells

ER -

TY - JOUR

IS - 2

JA - Radiat Res

JO - Radiation Research

PY - 2003

SN - 0033-7587

VL - 160

AU - Zeni O

AU - Chiavoni AS

AU - Sannino A

AU - Antolini A

AU - Forigo D

AU - Bersani F

AU - Scarfi MR

DO - 10.1667/rr3014

LA - en

N1 - FEMU ID: 10036; EMF-Portal URL: https://www.emf-portal.org/en/article/10036

SP - 152-158

TI - Lack of genotoxic effects (micronucleus induction) in human lymphocytes exposed in vitro to 900 MHz electromagnetic fields

ER -

TY - JOUR

IS - 4

JA - Radiat Res

JO - Radiation Research

PY - 2003

SN - 0033-7587

VL - 159

AU - Vijayalaxmi

AU - Sasser LB

AU - Morris JE

AU - Wilson BW

AU - Anderson LE

DO - 10.1667/0033-7587(2003)159[0558:gpogwc]2.0.co;2

LA - en

N1 - FEMU ID: 9893; EMF-Portal URL: https://www.emf-portal.org/en/article/9893

SP - 558-564

TI - Genotoxic potential of 1.6 GHz wireless communication signal: in vivo two-year bioassay

UR - https://meridian.allenpress.com/radiation-research/article-pdf/159/4/558/2194600/0033-7587(2003)159%5b0558_gpogwc%5d2_0_co_2.pdf

ER -

TY - JOUR

IS - 5

JA - Radiat Res

JO - Radiation Research

PY - 2003

SN - 0033-7587

VL - 159

AU - McNamee JP

AU - Bellier PV

AU - Gajda GB

AU - Lavallee BF

AU - Marro L

AU - Lemay E

AU - Thansandote A

DO - 10.1667/0033-7587(2003)159[0693:nefgef]2.0.co;2

LA - en

N1 - FEMU ID: 9883; EMF-Portal URL: https://www.emf-portal.org/en/article/9883

SP - 693-697

TI - No evidence for genotoxic effects from 24 h exposure of human leukocytes to 1.9 GHz radiofrequency fields

ER -

TY - JOUR

IS - 2

JO - Bioelectromagnetics

PY - 2003

SN - 0197-8462

VL - 24

AU - Mashevich M

AU - Folkman D

AU - Kesar A

AU - Barbul A

AU - Korenstein R

AU - Jerby E

AU - Avivi L

DO - 10.1002/bem.10086

LA - en

N1 - FEMU ID: 9413; EMF-Portal URL: https://www.emf-portal.org/en/article/9413

SP - 82-90

TI - Exposure of human peripheral blood lymphocytes to electromagnetic fields associated with cellular phones leads to chromosomal instability

ER -

TY - JOUR

IS - 1-2

JA - Mutat Res Genet Toxicol Environ Mutagen

JO - Mutation Research - Genetic Toxicology and Environmental Mutagenesis

PY - 2002

VL - 521

AU - Trosic I

AU - Busljeta I

AU - Kasuba V

AU - Rozgaj R

DO - 10.1016/s1383-5718(02)00214-0

LA - en

N1 - FEMU ID: 11629; EMF-Portal URL: https://www.emf-portal.org/en/article/11629

SP - 73-79

TI - Micronucleus induction after whole-body microwave irradiation of rats

ER -

TY - JOUR

IS - 4

JA - Biomed Environ Sci

JO - Biomedical and Environmental Sciences

PY - 2002

SN - 0895-3988

VL - 15

AU - Zhang MB

AU - He JL

AU - Jin LF

AU - Lu DQ

LA - en

N1 - FEMU ID: 9988; EMF-Portal URL: https://www.emf-portal.org/en/article/9988

SP - 283-290

TI - Study of low-intensity 2450-MHz microwave exposure enhancing the genotoxic effects of mitomycin C using micronucleus test and comet assay in vitro

ER -

TY - JOUR

IS - 4

JA - Radiat Res

JO - Radiation Research

PY - 2002

SN - 0033-7587

VL - 158

AU - McNamee JP

AU - Bellier PV

AU - Gajda GB

AU - Miller SM

AU - Lemay EP

AU - Lavallee BF

AU - Marro L

AU - Thansandote A

DO - 10.1667/0033-7587(2002)158[0523:ddamii]2.0.co;2

LA - en

N1 - FEMU ID: 9741; EMF-Portal URL: https://www.emf-portal.org/en/article/9741

SP - 523-533

TI - DNA damage and micronucleus induction in human leukocytes after acute in vitro exposure to a 1.9 GHz continuous-wave radiofrequency field

ER -

TY - JOUR

IS - 4

JA - Radiat Res

JO - Radiation Research

PY - 2002

SN - 0033-7587

VL - 158

AU - McNamee JP

AU - Bellier PV

AU - Gajda GB

AU - Lavallee BF

AU - Lemay EP

AU - Marro L

AU - Thansandote A

DO - 10.1667/0033-7587(2002)158[0534:ddihla]2.0.co;2

LA - en

N1 - FEMU ID: 9740; EMF-Portal URL: https://www.emf-portal.org/en/article/9740

SP - 534-537

TI - DNA damage in human leukocytes after acute in vitro exposure to a 1.9 GHz pulse-modulated radiofrequency field

ER -

TY - JOUR

IS - 5

JA - Radiat Res

JO - Radiation Research

PY - 2002

SN - 0033-7587

VL - 157

AU - Bisht KS

AU - Moros EG

AU - Straube WL

AU - Baty JD

AU - Roti Roti JL

DO - 10.1667/0033-7587(2002)157[0506:teomfo]2.0.co;2

LA - en

N1 - FEMU ID: 8831; EMF-Portal URL: https://www.emf-portal.org/en/article/8831

SP - 506-515

TI - The effect of 835.62 MHz FDMA or 847.74 MHz CDMA modulated radiofrequency radiation on the induction of micronuclei in C3H 10T(1/2) cells

ER -

TY - JOUR

IS - 2

JO - Bioelectromagnetics

PY - 2002

SN - 0197-8462

VL - 23

AU - Tice RR

AU - Hook GG

AU - Donner M

AU - McRee DI

AU - Guy AW

DO - 10.1002/bem.104

LA - en

N1 - FEMU ID: 8518; EMF-Portal URL: https://www.emf-portal.org/en/article/8518

SP - 113-126

TI - Genotoxicity of radiofrequency signals. I. Investigation of DNA damage and micronuclei induction in cultured human blood cells

ER -

TY - JOUR

IS - 1

JO - Bioelectromagnetics

PY - 2002

SN - 0197-8462

VL - 23

AU - d'Ambrosio G

AU - Massa R

AU - Scarfi MR

AU - Zeni O

DO - 10.1002/bem.93

LA - en

N1 - FEMU ID: 8130; EMF-Portal URL: https://www.emf-portal.org/en/article/8130

SP - 7-13

TI - Cytogenetic damage in human lymphocytes following GMSK phase modulated microwave exposure

ER -

TY - GEN

ET - 1

PB - Springer

PP - Boston

PY - 2001

SN - 9780306469015

T2 - Wireless Phones and Health II: State of the Science

AU - Hook GJ

DO - 10.1007/0-306-46901-4_11

LA - en

N1 - FEMU ID: 49553; EMF-Portal URL: https://www.emf-portal.org/en/article/49553

SP - 143-146

TI - Evaluation of potential genotoxicity of radiofrequency radiation technologies using the single cell gel electrophoresis and micronucleus assays

ER -

TY - JOUR

IS - 2

JO - Cytologia

PY - 2001

SN - 0011-4545

VL - 66

AU - Othman EO

AU - Aly MS

AU - El Nahas SM

DO - 10.1508/cytologia.66.117

LA - en

N1 - FEMU ID: 36938; EMF-Portal URL: https://www.emf-portal.org/en/article/36938

SP - 117-125

TI - Aneuploidy in workers occupationally exposed to electromagnetic field detected by FISH

UR - https://www.jstage.jst.go.jp/article/cytologia1929/66/2/66_2_117/_pdf/-char/en

ER -

TY - JOUR

IS - 2

JA - Acta Med Okayama

JO - Acta Medica Okayama

PY - 2001

SN - 0386-300X

VL - 55

AU - Lalic H

AU - Lekic A

AU - Radosevic-Stasic B

DO - 10.18926/AMO/32005

LA - en

N1 - FEMU ID: 8555; EMF-Portal URL: https://www.emf-portal.org/en/article/8555

SP - 117-127

TI - Comparison of chromosome aberrations in peripheral blood lymphocytes from people occupationally exposed to ionizing and radiofrequency radiation

UR - http://www.lib.okayama-u.ac.jp/www/acta/pdf/55_2_117.pdf

ER -

TY - JOUR

IS - 3

JA - Radiat Res

JO - Radiation Research

PY - 2001

SN - 0033-7587

VL - 156

AU - Li L

AU - Bisht KS

AU - Lagroye I

AU - Zhang P

AU - Straube WL

AU - Moros EG

AU - Roti Roti JL

DO - 10.1667/0033-7587(2001)156[0328:moddim]2.0.co;2

LA - en

N1 - FEMU ID: 8548; EMF-Portal URL: https://www.emf-portal.org/en/article/8548

SP - 328-332

TI - Measurement of DNA damage in mammalian cells exposed in vitro to radiofrequency fields at SARs of 3-5 W/kg

ER -

TY - JOUR

IS - 4

JA - Radiat Res

JO - Radiation Research

PY - 2001

SN - 0033-7587

VL - 156

AU - Vijayalaxmi

AU - Bisht KS

AU - Pickard WF

AU - Meltz ML

AU - Roti Roti JL

AU - Moros EG

DO - 10.1667/0033-7587(2001)156[0430:cdamfi]2.0.co;2

LA - en

N1 - FEMU ID: 7827; EMF-Portal URL: https://www.emf-portal.org/en/article/7827

SP - 430-433

TI - Chromosome damage and micronucleus formation in human blood lymphocytes exposed in vitro to radiofrequency radiation at a cellular telephone frequency (847.74 MHz, CDMA)

ER -

TY - JOUR

IS - 11

JA - Int J Radiat Biol

JO - International Journal of Radiation Biology

PY - 2001

SN - 0955-3002

VL - 77

AU - Vijayalaxmi

AU - Pickard WF

AU - Bisht KS

AU - Prihoda TJ

AU - Meltz ML

AU - LaRegina MC

AU - Roti Roti JL

AU - Straube WL

AU - Moros EG

DO - 10.1080/09553000110069100

LA - en

N1 - FEMU ID: 7826; EMF-Portal URL: https://www.emf-portal.org/en/article/7826

SP - 1109-1115

TI - Micronuclei in the peripheral blood and bone marrow cells of rats exposed to 2450 MHz radiofrequency radiation

ER -

TY - JOUR

IS - 5

JA - Radiat Res

JO - Radiation Research

PY - 2001

SN - 0033-7587

VL - 156

AU - Sykes PJ

AU - McCallum BD

AU - Bangay MJ

AU - Hooker AM

AU - Morley AA

DO - 10.1667/0033-7587(2001)156[0495:eoetmr]2.0.co;2

LA - en

N1 - FEMU ID: 7646; EMF-Portal URL: https://www.emf-portal.org/en/article/7646

SP - 495-502

TI - Effect of exposure to 900 MHz radiofrequency radiation on intrachromosomal recombination in pKZ1 mice

ER -

TY - JOUR

IS - 2

JO - Bioelectromagnetics

PY - 2001

SN - 0197-8462

VL - 22

AU - Maes A

AU - Collier M

AU - Verschaeve L

LA - en

N1 - FEMU ID: 5625; EMF-Portal URL: https://www.emf-portal.org/en/article/5625

SP - 91-96

TI - Cytogenetic effects of 900 MHz (GSM) microwaves on human lymphocytes

ER -

TY - JOUR

IS - 1

JA - Radiat Res

JO - Radiation Research

PY - 2001

SN - 0033-7587

VL - 155

AU - Vijayalaxmi

AU - Leal BZ

AU - Meltz ML

AU - Pickard WF

AU - Bisht KS

AU - Roti Roti JL

AU - Straube WL

AU - Moros EG

DO - 10.1667/0033-7587(2001)155[0113:csihbl]2.0.co;2

LA - en

N1 - FEMU ID: 5187; EMF-Portal URL: https://www.emf-portal.org/en/article/5187

SP - 113-121

TI - Cytogenetic studies in human blood lymphocytes exposed in vitro to radiofrequency radiation at a cellular telephone frequency (835.62 MHz, FDMA)

ER -

TY - JOUR

IS - 1-2

JA - Mutat Res Genet Toxicol Environ Mutagen

JO - Mutation Research - Genetic Toxicology and Environmental Mutagenesis

PY - 2000

VL - 471

AU - Zotti-Martelli L

AU - Peccatori M

AU - Scarpato R

AU - Migliore L

DO - 10.1016/s1383-5718(00)00112-1

LA - en

N1 - FEMU ID: 5088; EMF-Portal URL: https://www.emf-portal.org/en/article/5088

SP - 51-58

TI - Induction of micronuclei in human lymphocytes exposed in vitro to microwave radiation

ER -

TY - JOUR

IS - 5

JA - Folia Biol

JO - Folia Biologica

PY - 2000

SN - 0015-5500

VL - 46

AU - Maes A

AU - Collier M

AU - Verschaeve L

LA - en

N1 - FEMU ID: 4988; EMF-Portal URL: https://www.emf-portal.org/en/article/4988

SP - 175-180

TI - Cytogenetic investigations on microwaves emitted by a 455.7 MHz car phone

ER -

TY - JOUR

IS - 4

JA - Radiat Res

JO - Radiation Research

PY - 2000

SN - 0033-7587

VL - 153

AU - Vijayalaxmi

AU - Leal BZ

AU - Szilagyi M

AU - Prihoda TJ

AU - Meltz ML

DO - 10.1667/0033-7587(2000)153[0479:pddihb]2.0.co;2

LA - en

N1 - FEMU ID: 4299; EMF-Portal URL: https://www.emf-portal.org/en/article/4299

SP - 479-486

TI - Primary DNA damage in human blood lymphocytes exposed in vitro to 2450 MHz radiofrequency radiation

ER -

TY - JOUR

IS - 13

JO - Chemosphere

PY - 1999

SN - 0045-6535

VL - 39

AU - Garaj-Vrhovac V

DO - 10.1016/s0045-6535(99)00139-3

LA - en

N1 - FEMU ID: 5642; EMF-Portal URL: https://www.emf-portal.org/en/article/5642

SP - 2301-2312

TI - Micronucleus assay and lymphocyte mitotic activity in risk assessment of occupational exposure to microwave radiation

ER -

TY - JOUR

IS - 1

JA - Int J Radiat Biol

JO - International Journal of Radiation Biology

PY - 1999

SN - 0955-3002

VL - 75

AU - Vijayalaxmi

AU - Seaman RL

AU - Belt ML

AU - Doyle JM

AU - Mathur SP

AU - Prihoda TJ

DO - 10.1080/095530099140870

LA - en

N1 - FEMU ID: 2207; EMF-Portal URL: https://www.emf-portal.org/en/article/2207

SP - 115-120

TI - Frequency of micronuclei in the blood and bone marrow cells of mice exposed to ultra-wideband electromagnetic radiation

ER -

TY - JOUR

IS - 2

JA - J Microw Power Electromagn Energy

JO - Journal of Microwave Power and Electromagnetic Energy

PY - 1998

SN - 0832-7823

VL - 33

AU - Kuchma T

DO - 10.1080/08327823.1998.11688363

LA - en

N1 - FEMU ID: 11053; EMF-Portal URL: https://www.emf-portal.org/en/article/11053

SP - 77-87

TI - Synergistic effect of microwave heating and hydrogen peroxide on inactivation of microorganisms

ER -

TY - JOUR

JO - Edition Wissenschaft

PY - 1998

VL - 14

AU - Antonopoulos A

AU - Obe G

AU - Brinkmann K

AU - Eisenbrandt H

AU - Grigat JP

AU - Elsner R

AU - Storbeck W

AU - Dehmel G

LA - de

N1 - FEMU ID: 9446; EMF-Portal URL: https://www.emf-portal.org/en/article/9446

SP - 3-13

TI - Der Einfluß von hochfrequenten elektromagnetischen Feldern auf den Zellzyklus und auf die Frequenz von Schwesterchromatidaustauschen: Analysen an menschlichen Lymphozyten in Kultur

UR - https://d-nb.info/974863599/34

ER -

TY - JOUR

IS - 1

JA - Bioelectrochem Bioenerg

JO - Bioelectrochemistry and Bioenergetics

PY - 1998

SN - 0302-4598

VL - 45

AU - Phillips JL

AU - Ivaschuk O

AU - Ishida-Jones T

AU - Jones RA

AU - Campbell-Beachler M

AU - Haggren W

LA - en

N1 - FEMU ID: 2112; EMF-Portal URL: https://www.emf-portal.org/en/article/2112

SP - 103-110

TI - DNA damage in Molt-4 T-lymphoblastoid cells exposed to cellular telephone radiofrequency fields in vitro

ER -

TY - JOUR

IS - 2

JO - Bioelectromagnetics

PY - 1998

SN - 0197-8462

VL - 19

AU - Pakhomova ON

AU - Belt ML

AU - Mathur SP

AU - Lee JC

AU - Akyel Y

DO - 10.1002/(sici)1521-186x(1998)19:2<128::aid-bem12>3.0.co;2-m

LA - en

N1 - FEMU ID: 2082; EMF-Portal URL: https://www.emf-portal.org/en/article/2082

SP - 128-130

TI - Ultra-wide band electromagnetic radiation does not affect UV-induced recombination and mutagenesis in yeast

ER -

TY - JOUR

IS - 6

JA - Radiat Res

JO - Radiation Research

PY - 1998

SN - 0033-7587

VL - 149

AU - Malyapa RS

AU - Ahern EW

AU - Bi C

AU - Straube WL

AU - LaRegina MC

AU - Pickard WF

AU - Roti Roti JL

LA - en

N1 - FEMU ID: 1374; EMF-Portal URL: https://www.emf-portal.org/en/article/1374

SP - 637-645

TI - DNA damage in rat brain cells after in vivo exposure to 2450 MHz electromagnetic radiation and various methods of euthanasia

ER -

TY - JOUR

IS - 6

JA - Wirel Netw

JO - Wireless Networks

PY - 1997

VL - 3

AU - Lai H

AU - Carino M

AU - Singh N

DO - 10.1023/A:1019154611749

LA - en

N1 - FEMU ID: 10681; EMF-Portal URL: https://www.emf-portal.org/en/article/10681

SP - 471-476

TI - Naltrexone blocks RFR-induced DNA double strand breaks in rat brain cells

UR - https://dl.acm.org/doi/pdf/10.1023/A%3A1019154611749

ER -

TY - JOUR

IS - 3

JA - Electro Magnetobiol

JO - Electro- and Magnetobiology

PY - 1997

SN - 1061-9526

VL - 16

AU - Pakhomova ON

AU - Belt MN

AU - Mathur SP

AU - Lee JC

AU - Akyel Y

DO - 10.3109/15368379709015652

LA - en

N1 - FEMU ID: 8582; EMF-Portal URL: https://www.emf-portal.org/en/article/8582

SP - 195-201

TI - Lack of genetic effects of ultra-wide band electromagnetic radiation in yeast

ER -

TY - JOUR

IS - 6

JA - Int J Radiat Biol

JO - International Journal of Radiation Biology

PY - 1997

SN - 0955-3002

VL - 72

AU - Vijayalaxmi

AU - Mohan N

AU - Meltz ML

AU - Wittler MA

DO - 10.1080/095530097142915

LA - en

N1 - FEMU ID: 2359; EMF-Portal URL: https://www.emf-portal.org/en/article/2359

SP - 751-757

TI - Proliferation and cytogenetic studies in human blood lymphocytes exposed in vitro to 2450 MHz radiofrequency radiation

ER -

TY - JOUR

IS - 2-3

JA - Mutat Res Genet Toxicol Environ Mutagen

JO - Mutation Research - Genetic Toxicology and Environmental Mutagenesis

PY - 1997

VL - 395

AU - Antonopoulos A

AU - Eisenbrandt H

AU - Obe G

DO - 10.1016/s1383-5718(97)00173-3

LA - en

N1 - FEMU ID: 2200; EMF-Portal URL: https://www.emf-portal.org/en/article/2200

SP - 209-214

TI - Effects of high-frequency electromagnetic fields on human lymphocytes in vitro

ER -

TY - JOUR

IS - 6

JA - Radiat Res

JO - Radiation Research

PY - 1997

SN - 0033-7587

VL - 148

AU - Malyapa RS

AU - Ahern EW

AU - Straube WL

AU - Moros EG

AU - Pickard WF

AU - Roti Roti JL

LA - en

N1 - FEMU ID: 2070; EMF-Portal URL: https://www.emf-portal.org/en/article/2070

SP - 608-617

TI - Measurement of DNA damage after exposure to 2450 MHz electromagnetic radiation

ER -

TY - JOUR

IS - 4

JA - Radiat Res

JO - Radiation Research

PY - 1997

SN - 0033-7587

VL - 147

AU - Vijayalaxmi

AU - Frei MR

AU - Dusch SJ

AU - Guel V

AU - Meltz ML

AU - Jauchem JR

LA - en

N1 - FEMU ID: 1413; EMF-Portal URL: https://www.emf-portal.org/en/article/1413

SP - 495-500

TI - Frequency of micronuclei in the peripheral blood and bone marrow of cancer-prone mice chronically exposed to 2450 MHz radiofrequency radiation

ER -

TY - JOUR

IS - 6

JO - Bioelectromagnetics

PY - 1997

SN - 0197-8462

VL - 18

AU - Lai H

AU - Singh NP

DO - 10.1002/(sici)1521-186x(1997)18:6<446::aid-bem7>3.0.co;2-2

LA - en

N1 - FEMU ID: 1257; EMF-Portal URL: https://www.emf-portal.org/en/article/1257

SP - 446-454

TI - Melatonin and a spin-trap compound block radiofrequency electromagnetic radiation-induced DNA strand breaks in rat brain cells

ER -

TY - JOUR

IS - 1-2

JA - Mutat Res Genet Toxicol Environ Mutagen

JO - Mutation Research - Genetic Toxicology and Environmental Mutagenesis

PY - 1997

VL - 393

AU - Maes A

AU - Collier M

AU - Van Gorp U

AU - Vandoninck S

AU - Verschaeve L

DO - 10.1016/s1383-5718(97)00100-9

LA - en

N1 - FEMU ID: 948; EMF-Portal URL: https://www.emf-portal.org/en/article/948

SP - 151-156

TI - Cytogenetic effects of 935.2-MHz (GSM) microwaves alone and in combination with mitomycin C

ER -

TY - JOUR

IS - 6

JA - Radiat Res

JO - Radiation Research

PY - 1997

SN - 0033-7587

VL - 148

AU - Malyapa RS

AU - Ahern EW

AU - Straube WL

AU - Moros EG

AU - Pickard WF

AU - Roti Roti JL

LA - en

N1 - FEMU ID: 947; EMF-Portal URL: https://www.emf-portal.org/en/article/947

SP - 618-627

TI - Measurement of DNA damage after exposure to electromagnetic radiation in the cellular phone communication frequency band (835.62 and 847.74 MHz)

ER -

TY - JOUR

JO - Edition Wissenschaft

PY - 1996

VL - 4

AU - Eberle P

AU - Erdtmann-Vourliotis M

AU - Diener S

AU - Finke HG

AU - Löffelholz B

AU - Schnor A

AU - Schräder M

LA - de

N1 - FEMU ID: 9467; EMF-Portal URL: https://www.emf-portal.org/en/article/9467

SP - 2-15

TI - Zellproliferation, Schwesterchromatidaustausche, Chromosomenaberrationen, Mikrokerne und Mutationsrate des HGPRT-Locus

UR - https://d-nb.info/974863475/34

ER -

TY - JOUR

JO - Edition Wissenschaft

PY - 1996

VL - 8

AU - Hansen V

AU - Rüger W

LA - de

N1 - FEMU ID: 9463; EMF-Portal URL: https://www.emf-portal.org/en/article/9463

SP - 3-38

TI - Wirkung hochfrequenter elektromagnetischer Felder auf DNA, Proteine und DNA-Protein-Komplexe

UR - https://d-nb.info/974870188/34

ER -

TY - JOUR

IS - 1

JA - Sci Total Environ

JO - Science of the Total Evironment

PY - 1996

SN - 0048-9697

VL - 180

AU - Balode Z

DO - 10.1016/0048-9697(95)04923-1

LA - en

N1 - FEMU ID: 2132; EMF-Portal URL: https://www.emf-portal.org/en/article/2132

SP - 81-85

TI - Assessment of radio-frequency electromagnetic radiation by the micronucleus test in bovine peripheral erythrocytes

ER -

TY - JOUR

IS - 4

JA - Int J Radiat Biol

JO - International Journal of Radiation Biology

PY - 1996

SN - 0955-3002

VL - 69

AU - Lai H

AU - Singh NP

DO - 10.1080/095530096145814

LA - en

N1 - FEMU ID: 1389; EMF-Portal URL: https://www.emf-portal.org/en/article/1389

SP - 513-521

TI - Single- and double-strand DNA breaks in rat brain cells after acute exposure to radiofrequency electromagnetic radiation

ER -

TY - JOUR

IS - 2

JA - Electro Magnetobiol

JO - Electro- and Magnetobiology

PY - 1996

SN - 1061-9526

VL - 15

AU - Scarfi MR

AU - Lioi MB

AU - d'Ambrosio G

AU - Massa R

AU - Zeni O

AU - Di Pietto R

AU - Di Berardino D

DO - 10.3109/15368379609009826

LA - en

N1 - FEMU ID: 959; EMF-Portal URL: https://www.emf-portal.org/en/article/959

SP - 99-107

TI - Genotoxic effects of mitomycin-c and microwave radiation on bovine lymphocytes

ER -

TY - JOUR

IS - 1

JA - Environ Mol Mutagen

JO - Environmental and Molecular Mutagenesis

PY - 1996

SN - 0893-6692

VL - 28

AU - Maes A

AU - Collier M

AU - Slaets D

AU - Verschaeve L

DO - 10.1002/(SICI)1098-2280(1996)28:1<26::AID-EM6>3.0.CO;2-C

LA - en

N1 - FEMU ID: 934; EMF-Portal URL: https://www.emf-portal.org/en/article/934

SP - 26-30

TI - 954 MHz microwaves enhance the mutagenic properties of mitomycin C

ER -

TY - JOUR

IS - 3

JO - Bioelectromagnetics

PY - 1995

SN - 0197-8462

VL - 16

AU - Lai H

AU - Singh NP

DO - 10.1002/bem.2250160309

LA - en

N1 - FEMU ID: 1385; EMF-Portal URL: https://www.emf-portal.org/en/article/1385

SP - 207-210

TI - Acute low-intensity microwave exposure increases DNA single-strand breaks in rat brain cells

ER -

TY - JOUR

IS - 3

JA - Electro Magnetobiol

JO - Electro- and Magnetobiology

PY - 1995

SN - 1061-9526

VL - 14

AU - d'Ambrosio G

AU - Lioi MB

AU - Massa R

AU - Scarfi MR

AU - Zeni O

DO - 10.3109/15368379509030726

LA - en

N1 - FEMU ID: 905; EMF-Portal URL: https://www.emf-portal.org/en/article/905

SP - 157-164

TI - Genotoxic Effects of Amplitude-Modulated Microwaves on Human Lymphocytes Exposed in Vitro under Controlled Conditions

ER -

TY - JOUR

IS - 2

JA - Electro Magnetobiol

JO - Electro- and Magnetobiology

PY - 1995

SN - 1061-9526

VL - 14

AU - Maes A

AU - Collier M

AU - Slaets D

AU - Verschaeve L

LA - en

N1 - FEMU ID: 891; EMF-Portal URL: https://www.emf-portal.org/en/article/891

SP - 91-98

TI - Cytogenetic Effects of Microwaves from Mobile Communication Frequencies (954 MHz)

ER -

TY - JOUR

IS - 1-2

JO - Mutation Research - Letters

PY - 1994

VL - 328

AU - Haider T

AU - Knasmueller S

AU - Kundi M

AU - Haider M

DO - 10.1016/0165-7992(94)90069-8

LA - en

N1 - FEMU ID: 873; EMF-Portal URL: https://www.emf-portal.org/en/article/873

SP - 65-68

TI - Clastogenic effects of radiofrequency radiations on chromosomes of Tradescantia

ER -

TY - JOUR

IS - 6

JA - Acta Radiol

JO - Acta Radiologica

PY - 1993

SN - 0284-1851

VL - 34

AU - Yamazaki E

AU - Matsubara S

AU - Yamada I

LA - en

N1 - FEMU ID: 22349; EMF-Portal URL: https://www.emf-portal.org/en/article/22349

SP - 607-611

TI - Effect of Gd-DTPA and/or magnetic field and radiofrequency exposure on sister chromatid exchange in human peripheral lymphocytes

ER -

TY - JOUR

JA - Bioelectrochem Bioenerg

JO - Bioelectrochemistry and Bioenergetics

PY - 1993

SN - 0302-4598

VL - 30

AU - Garaj-Vrhovac V

AU - Fucic A

DO - 10.1016/0302-4598(93)80091-8

LA - en

N1 - FEMU ID: 1874; EMF-Portal URL: https://www.emf-portal.org/en/article/1874

SP - 319-325

TI - The rate of elimination of chromosomal aberrations after accidental exposure to microwave radiation

ER -

TY - JOUR

IS - 6

JO - Bioelectromagnetics

PY - 1993

SN - 0197-8462

VL - 14

AU - Maes A

AU - Verschaeve L

AU - Arroyo A

AU - De Wagter C

AU - Vercruyssen L

DO - 10.1002/bem.2250140602

LA - en

N1 - FEMU ID: 889; EMF-Portal URL: https://www.emf-portal.org/en/article/889

SP - 495-501

TI - In vitro cytogenetic effects of 2450 MHz waves on human peripheral blood lymphocytes

ER -

TY - JOUR

IS - 2

JA - Electro Magnetobiol

JO - Electro- and Magnetobiology

PY - 1992

SN - 1061-9526

VL - 11

AU - Belyaev IY

AU - Alipov YD

AU - Shcheglov VS

DO - 10.3109/15368379209009820

LA - en

N1 - FEMU ID: 2115; EMF-Portal URL: https://www.emf-portal.org/en/article/2115

SP - 97-108

TI - Chromosome DNA as a target of resonant interaction between Escherichia coli cells and low-intensity millimeter waves

ER -

TY - JOUR

IS - 3

JO - Mutation Research - Letters

PY - 1992

VL - 281

AU - Garaj-Vrhovac V

AU - Fucic A

AU - Horvat D

DO - 10.1016/0165-7992(92)90006-4

LA - en

N1 - FEMU ID: 876; EMF-Portal URL: https://www.emf-portal.org/en/article/876

SP - 181-186

TI - The correlation between the frequency of micronuclei and specific chromosome aberrations in human lymphocytes exposed to microwave radiation in vitro

ER -

TY - JOUR

IS - 4

JO - Mutation Research - Letters

PY - 1992

VL - 282

AU - Fucic A

AU - Garaj-Vrhovac V

AU - Skara M

AU - Dimitrovic B

DO - 10.1016/0165-7992(92)90133-3

LA - en

N1 - FEMU ID: 875; EMF-Portal URL: https://www.emf-portal.org/en/article/875

SP - 265-271

TI - X-rays, microwaves and vinyl chloride monomer: their clastogenic and aneugenic activity, using the micronucleus assay on human lymphocytes

ER -

TY - JOUR

IS - 2

JA - Biochem Int

JO - Biochemistry International

PY - 1991

SN - 0158-5231

VL - 25

AU - Narasimhan V

AU - Huh WK

LA - en

N1 - FEMU ID: 8617; EMF-Portal URL: https://www.emf-portal.org/en/article/8617

SP - 363-370

TI - Altered restriction patterns of microwave irradiated lambda-phage DNA

ER -

TY - JOUR

IS - 5

JO - Bioelectromagnetics

PY - 1991

SN - 0197-8462

VL - 12

AU - Ciaravino V

AU - Meltz ML

AU - Erwin DN

DO - 10.1002/bem.2250120504

LA - en

N1 - FEMU ID: 1770; EMF-Portal URL: https://www.emf-portal.org/en/article/1770

SP - 289-298

TI - Absence of a synergistic effect between moderate-power radio-frequency electromagnetic radiation and adriamycin on cell-cycle progression and sister-chromatid exchange

ER -

TY - JOUR

IS - 5

JA - Med J Aust

JO - The Medical Journal of Australia

PY - 1991

SN - 0025-729X

VL - 155

AU - Garson OM

AU - McRobert TL

AU - Campbell LJ

AU - Hocking BA

AU - Gordon I

DO - 10.5694/j.1326-5377.1991.tb142282.x

LA - en

N1 - FEMU ID: 1407; EMF-Portal URL: https://www.emf-portal.org/en/article/1407

SP - 289-292

TI - A chromosomal study of workers with long-term exposure to radio-frequency radiation

ER -

TY - JOUR

IS - 3

JO - Mutation Research - Letters

PY - 1991

VL - 263

AU - Garaj-Vrhovac V

AU - Horvat D

AU - Koren Z

DO - 10.1016/0165-7992(91)90054-8

LA - en

N1 - FEMU ID: 877; EMF-Portal URL: https://www.emf-portal.org/en/article/877

SP - 143-149

TI - The relationship between colony-forming ability, chromosome aberrations and incidence of micronuclei in V79 Chinese hamster cells exposed to microwave radiation

ER -

TY - JOUR

IS - 4

JA - Period Biol

JO - Periodicum Biologorum

PY - 1990

SN - 0031-5362

VL - 92

AU - Garaj-Vrhovac V

AU - Fucic A

AU - Horvat D

LA - en

N1 - FEMU ID: 9585; EMF-Portal URL: https://www.emf-portal.org/en/article/9585

SP - 411-416

TI - Comparison of chromosome aberration and micronucleus induction in human lymphocytes after occupational exposure to vinyl chloride monomer and microwave radiation

ER -

TY - JOUR

IS - 3

JA - Radiat Res

JO - Radiation Research

PY - 1990

SN - 0033-7587

VL - 123

AU - Kerbacher JJ

AU - Meltz ML

AU - Erwin DN

LA - en

N1 - FEMU ID: 3403; EMF-Portal URL: https://www.emf-portal.org/en/article/3403

SP - 311-319

TI - Influence of radiofrequency radiation on chromosome aberrations in CHO cells and its interaction with DNA-damaging agents

ER -

TY - JOUR

IS - 2

JO - Bioelectromagnetics

PY - 1990

SN - 0197-8462

VL - 11

AU - Meltz ML

AU - Eagan P

AU - Erwin DN

DO - 10.1002/bem.2250110206

LA - en

N1 - FEMU ID: 888; EMF-Portal URL: https://www.emf-portal.org/en/article/888

SP - 149-157

TI - Proflavin and microwave radiation: absence of a mutagenic interaction

ER -

TY - JOUR

IS - 3

JO - Mutation Research - Letters

PY - 1990

VL - 243

AU - Garaj-Vrhovac V

AU - Horvat D

AU - Koren Z

DO - 10.1016/0165-7992(90)90028-i

LA - en

N1 - FEMU ID: 878; EMF-Portal URL: https://www.emf-portal.org/en/article/878

SP - 87-93

TI - The effect of microwave radiation on the cell genome

ER -

TY - JOUR

IS - 4

JA - Environ Mol Mutagen

JO - Environmental and Molecular Mutagenesis

PY - 1989

SN - 0893-6692

VL - 13

AU - Meltz ML

AU - Eagan P

AU - Erwin DN

DO - 10.1002/em.2850130404

LA - en

N1 - FEMU ID: 932; EMF-Portal URL: https://www.emf-portal.org/en/article/932

SP - 294-303

TI - Absence of mutagenic interaction between microwaves and mitomycin C in mammalian cells

ER -

TY - JOUR

IS - 3

JA - Int J Biometeorol

JO - International Journal of Biometeorology

PY - 1987

SN - 0020-7128

VL - 31

AU - Levengood WC

DO - 10.1007/BF02188921

LA - en

N1 - FEMU ID: 2114; EMF-Portal URL: https://www.emf-portal.org/en/article/2114

SP - 185-190

TI - Non-disjunction mutations in Drosophila exposed to magnetic fields

ER -

TY - JOUR

IS - 2

JA - Radiat Res

JO - Radiation Research

PY - 1987

SN - 0033-7587

VL - 110

AU - Sagripanti JL

AU - Swicord ML

AU - Davis CC

DO - 10.2307/3576900

LA - en

N1 - FEMU ID: 2077; EMF-Portal URL: https://www.emf-portal.org/en/article/2077

SP - 219-231

TI - Microwave effects on plasmid DNA

ER -

TY - JOUR

IS - 4

JA - Environ Mutagen

JO - Environmental Mutagenesis

PY - 1987

SN - 0192-2521

VL - 9

AU - Ciaravino V

AU - Meltz ML

AU - Erwin DN

DO - 10.1002/em.2860090405

LA - en

N1 - FEMU ID: 2060; EMF-Portal URL: https://www.emf-portal.org/en/article/2060

SP - 393-399

TI - Effects of radiofrequency radiation and simultaneous exposure with mitomycin C on the frequency of sister chromatid exchanges in Chinese hamster ovary cells

ER -

TY - JOUR

IS - 2

JA - Radiat Res

JO - Radiation Research

PY - 1987

SN - 0033-7587

VL - 110

AU - Meltz ML

AU - Walker KA

AU - Erwin DN

LA - en

N1 - FEMU ID: 871; EMF-Portal URL: https://www.emf-portal.org/en/article/871

SP - 255-266

TI - Radiofrequency (microwave) radiation exposure of mammalian cells during UV-induced DNA repair synthesis

ER -

TY - JOUR

IS - 3

JA - Radiat Res

JO - Radiation Research

PY - 1986

SN - 0033-7587

VL - 108

AU - Roux C

AU - Elefant E

AU - Gaboriaud G

AU - Jaullery C

AU - Gardette J

AU - Dupuis R

AU - Lambert D

LA - en

N1 - FEMU ID: 8762; EMF-Portal URL: https://www.emf-portal.org/en/article/8762

SP - 317-326

TI - Association of microwaves and ionizing radiation: potentiation of teratogenic effects in the rat

ER -

TY - JOUR

IS - 1

JA - Int J Radiat Biol Relat Stud Phys Chem Med

JO - International Journal of Radiation Biology and Related Studies in Physics, Chemistry and Medicine

PY - 1986

SN - 0020-7616

VL - 50

AU - Sagripanti JL

AU - Swicord ML

DO - 10.1080/09553008614550431

LA - en

N1 - FEMU ID: 7147; EMF-Portal URL: https://www.emf-portal.org/en/article/7147

SP - 47-50

TI - DNA structural changes caused by microwave radiation

ER -

TY - JOUR

IS - 5

JA - Int J Radiat Biol Relat Stud Phys Chem Med

JO - International Journal of Radiation Biology and Related Studies in Physics, Chemistry and Medicine

PY - 1986

SN - 0020-7616

VL - 50

AU - Beechey CV

AU - Brooker D

AU - Kowalczuk CI

AU - Saunders RD

AU - Searle AG

DO - 10.1080/09553008614551321

LA - en

N1 - FEMU ID: 1388; EMF-Portal URL: https://www.emf-portal.org/en/article/1388

SP - 909-918

TI - Cytogenetic effects of microwave irradiation on male germ cells of the mouse

ER -

TY - JOUR

IS - 2

JO - Bioelectromagnetics

PY - 1986

SN - 0197-8462

VL - 7

AU - Lloyd DC

AU - Saunders RD

AU - Moquet JE

AU - Kowalczuk CI

DO - 10.1002/bem.2250070212

LA - en

N1 - FEMU ID: 893; EMF-Portal URL: https://www.emf-portal.org/en/article/893

SP - 235-237

TI - Absence of chromosomal damage in human lymphocytes exposed to microwave radiation with hyperthermia

ER -

TY - JOUR

IS - 1

JO - Radiology

PY - 1985

SN - 0033-8419

VL - 155

AU - Wolff S

AU - James TL

AU - Young GB

AU - Margulis AR

AU - Bodycote J

AU - Afzal V

DO - 10.1148/radiology.155.1.4038809

LA - en

N1 - FEMU ID: 22347; EMF-Portal URL: https://www.emf-portal.org/en/article/22347

SP - 163-165

TI - Magnetic resonance imaging: absence of in vitro cytogenetic damage

ER -

TY - JOUR

IS - 1

JA - J Hered

JO - The Journal of Heredity

PY - 1985

SN - 0022-1503

VL - 76

AU - Manikowska-Czerska E

AU - Czerski P

AU - Leach WM

DO - 10.1093/oxfordjournals.jhered.a110027

LA - en

N1 - FEMU ID: 922; EMF-Portal URL: https://www.emf-portal.org/en/article/922

SP - 71-73

TI - Effects of 2.45 GHz microwaves on meiotic chromosomes of male CBA/CAY mice

ER -

TY - JOUR

IS - 2

JA - Int J Radiat Biol Relat Stud Phys Chem Med

JO - International Journal of Radiation Biology and Related Studies in Physics, Chemistry and Medicine

PY - 1984

SN - 0020-7616

VL - 46

AU - Lloyd DC

AU - Saunders RD

AU - Finnon P

AU - Kowalczuk CI

DO - 10.1080/09553008414551211

LA - en

N1 - FEMU ID: 13126; EMF-Portal URL: https://www.emf-portal.org/en/article/13126

SP - 135-141

TI - No clastogenic effect from in vitro microwave irradiation of G0 human lymphocytes

ER -

TY - JOUR

IS - 3

JA - J Natl Cancer Inst

JO - Journal of the National Cancer Institute

PY - 1983

SN - 0027-8874

VL - 70

AU - Banerjee R

AU - Goldfeder A

AU - Mitra J

LA - en

N1 - FEMU ID: 9033; EMF-Portal URL: https://www.emf-portal.org/en/article/9033

SP - 517-521

TI - Sister chromatid exchanges and chromosome aberrations induced by radiosensitizing agents in bone marrow cells of treated tumor-bearing mice

ER -

TY - JOUR

IS - 2

JA - J Hered

JO - The Journal of Heredity

PY - 1982

SN - 0022-1503

VL - 73

AU - Yao KT

DO - 10.1093/oxfordjournals.jhered.a109596

LA - en

N1 - FEMU ID: 927; EMF-Portal URL: https://www.emf-portal.org/en/article/927

SP - 133-138

TI - Cytogenetic consequences of microwave irradiation on mammalian cells incubated in vitro

ER -

TY - JOUR

IS - 2

JA - Radiat Res

JO - Radiation Research

PY - 1981

SN - 0033-7587

VL - 85

AU - McRee DI

AU - MacNichols G

LA - en

N1 - FEMU ID: 1399; EMF-Portal URL: https://www.emf-portal.org/en/article/1399

SP - 340-348

TI - Incidence of sister chromatid exchange in bone marrow cells of the mouse following microwave exposure

ER -

TY - JOUR

IS - 3

JO - Experientia

PY - 1979

SN - 0014-4754

VL - 35

AU - Manikowska E

AU - Luciani JM

AU - Servantie B

AU - Czerski P

AU - Obrenovitch J

AU - Stahl A

DO - 10.1007/BF01964370

LA - en

N1 - FEMU ID: 9731; EMF-Portal URL: https://www.emf-portal.org/en/article/9731

SP - 388-390

TI - Effects of 9.4 GHz microwave exposure on meiosis in mice

ER -

TY - JOUR

IS - 6

JA - J Hered

JO - The Journal of Heredity

PY - 1978

SN - 0022-1503

VL - 69

AU - Yao KT

DO - 10.1093/oxfordjournals.jhered.a108983

LA - en

N1 - FEMU ID: 13540; EMF-Portal URL: https://www.emf-portal.org/en/article/13540

SP - 409-412

TI - Microwave radiation-induced chromosomal aberrations in corneal epithelium of Chinese hamsters

ER -

TY - JOUR

IS - 1

JA - Can J Genet Cytol

JO - Canadian Journal of Genetics and Cytology

PY - 1978

SN - 0008-4093

VL - 20

AU - Alam MT

AU - Barthakur N

AU - Lambert NG

AU - Kasatiya SS

DO - 10.1139/g78-004

LA - en

N1 - FEMU ID: 933; EMF-Portal URL: https://www.emf-portal.org/en/article/933

SP - 23-30

TI - Cytological effects of microwave radiation in Chinese hamster cells in vitro

ER -

TY - JOUR

IS - 6

JA - Radio Sci

JO - Radio Science

PY - 1977

SN - 0048-6604

VL - 12

AU - Huang AT

AU - Engle ME

AU - Elder JA

AU - Kinn JB

AU - Ward TR

DO - 10.1029/RS012i06Sp00173

LA - en

N1 - FEMU ID: 31069; EMF-Portal URL: https://www.emf-portal.org/en/article/31069

SP - 173 - 177

TI - The effect of microwave radiation (2450 MHz) on the morphology and chromosomes of lymphocytes

ER -

TY - JOUR

IS - 3

JA - Environ Res

JO - Environmental Research

PY - 1976

SN - 0013-9351

VL - 11

AU - Mittler S

DO - 10.1016/0013-9351(76)90094-3

LA - en

N1 - FEMU ID: 1410; EMF-Portal URL: https://www.emf-portal.org/en/article/1410

SP - 326-330

TI - Failure of 2- and 10-meter radio waves to induce genetic damage in Drosophila melanogaster

ER -

TY - JOUR

IS - 1

JA - Environ Lett

JO - Environmental Letters

PY - 1974

SN - 0013-9300

VL - 6

AU - Chen KM

AU - Samuel A

AU - Hoopingarner R

DO - 10.1080/00139307409437344

LA - en

N1 - FEMU ID: 7715; EMF-Portal URL: https://www.emf-portal.org/en/article/7715

SP - 37-46

TI - Chromosomal aberrations of living cells induced by microwave radiation

ER -

TY - JOUR

IS - 1

JA - J Appl Physiol

JO - Journal of Applied Physiology

PY - 1972

SN - 0021-8987

VL - 32

AU - McLees BD

AU - Finch ED

AU - Albright ML

DO - 10.1152/jappl.1972.32.1.78

LA - en

N1 - FEMU ID: 12703; EMF-Portal URL: https://www.emf-portal.org/en/article/12703

SP - 78-85

TI - An examination of regenerating hepatic tissue subjected to radio-frequency irradiation

ER -
