# Supplementary material for: A scoping review and evidence map of radiofrequency field exposure and genotoxicity: assessing in vivo, in vitro, and epidemiological data
Source: Front Public Health. 2025 Jul 30;13:1613353. doi: 10.3389/fpubh.2025.1613353 (PMC12343714; doi:10.3389/fpubh.2025.1613353)
Supplement: Supplementary file 3 [file Data_Sheet_3.zip › Search data/EMF Portal Search - Genotoxicity.docx]

EMF Portal key word search

The following terms were included: Genotoxizität, genotoxicity, 遺伝毒性

TY - JOUR

IS - 3

JA - Int J Mol Sci

JO - International Journal of Molecular Sciences

PY - 2023

SN - 1422-0067

VL - 24

AU - Szilágyi Z

AU - Németh Z

AU - Bakos J

AU - Kubinyi G

AU - Necz PP

AU - Szabó E

AU - Thuróczy G

AU - Pinto R

AU - Selmaoui B

DO - 10.3390/ijms24032853

LA - en

N1 - FEMU ID: 49895; EMF-Portal URL: https://www.emf-portal.org/en/article/49895

SP - 2853

TI - Assessment of Inflammation in 3D Reconstructed Human Skin Exposed to Combined Exposure to Ultraviolet and Wi-Fi Radiation

UR - https://www.mdpi.com/1422-0067/24/3/2853/pdf?version=1675316287

ER -

TY - JOUR

JO - Neurotoxicology

PY - 2023

SN - 0161-813X

VL - 94

AU - Singh KV

AU - Prakash C

AU - Nirala JP

AU - Nanda RK

AU - Rajamani P

DO - 10.1016/j.neuro.2022.11.001

LA - en

N1 - FEMU ID: 48916; EMF-Portal URL: https://www.emf-portal.org/en/article/48916

SP - 46-58

TI - Acute radiofrequency electromagnetic radiation exposure impairs neurogenesis and causes neuronal DNA damage in the young rat brain

ER -

TY - JOUR

IS - 1

JA - J Biophotonics

JO - Journal of Biophotonics

PY - 2023

SN - 1864-063X

VL - 16

AU - Sitnikov DS

AU - Revkova VA

AU - Ilina IV

AU - Gurova SA

AU - Komarov PS

AU - Struleva EV

AU - Konoplyannikov MA

AU - Kalsin VA

AU - Baklaushev VP

DO - 10.1002/jbio.202200212

LA - en

N1 - FEMU ID: 48706; EMF-Portal URL: https://www.emf-portal.org/en/article/48706

SP - e202200212

TI - Studying the genotoxic effects of high intensity terahertz radiation on fibroblasts and CNS tumor cells

ER -

TY - JOUR

IS - 1

JO - Protoplasma

PY - 2023

SN - 0033-183X

VL - 260

AU - Sharma S

AU - Bahel S

AU - Kaur Katnoria J

DO - 10.1007/s00709-022-01768-9

LA - en

N1 - FEMU ID: 47420; EMF-Portal URL: https://www.emf-portal.org/en/article/47420

SP - 209-224

TI - Evaluation of oxidative stress and genotoxicity of 900 MHz electromagnetic radiations using Trigonella foenum-graecum test system

ER -

TY - JOUR

IS - 24

JO - Cells

PY - 2022

SN - 2073-4409

VL - 11

AU - Cappucci U

AU - Casale AM

AU - Proietti M

AU - Marinelli F

AU - Giuliani L

AU - Piacentini L

DO - 10.3390/cells11244036

LA - en

N1 - FEMU ID: 49389; EMF-Portal URL: https://www.emf-portal.org/en/article/49389

SP - 4036

TI - WiFi Related Radiofrequency Electromagnetic Fields Promote Transposable Element Dysregulation and Genomic Instability in Drosophila melanogaster

UR - https://www.mdpi.com/2073-4409/11/24/4036/pdf?version=1671612228

ER -

TY - GEN

ET - 1

PB - IEEE

PY - 2022

SN - 9781665471114

T2 - 2022 Microwave Mediterranean Symposium (MMS)

AU - Sannino A

AU - Scarfi MR

AU - Romeo S

AU - Priault M

AU - Dufossee M

AU - Poeta L

AU - Prouzet-Mauleon V

AU - Zeni O

DO - 10.1109/MMS55062.2022.9825588

LA - en

N1 - FEMU ID: 48629; EMF-Portal URL: https://www.emf-portal.org/en/article/48629

SP - 1-5

TI - Possible role of autophagy in in vitro radiofrequency-induced adaptive response

ER -

TY - JOUR

IS - 15

JA - Int J Mol Sci

JO - International Journal of Molecular Sciences

PY - 2022

SN - 1422-0067

VL - 23

AU - Sannino A

AU - Scarfì MR

AU - Dufossée M

AU - Romeo S

AU - Poeta L

AU - Prouzet-Mauléon V

AU - Priault M

AU - Zeni O

DO - 10.3390/ijms23158414

LA - en

N1 - FEMU ID: 48101; EMF-Portal URL: https://www.emf-portal.org/en/article/48101

SP - 8414

TI - Inhibition of Autophagy Negates Radiofrequency-Induced Adaptive Response in SH-SY5Y Neuroblastoma Cells

UR - https://www.mdpi.com/1422-0067/23/15/8414/pdf?version=1659342995

ER -

TY - JOUR

IS - 4

JA - J Int Oral Health

JO - Journal of International Oral Health

PY - 2021

SN - 0976-1799

VL - 13

AU - Thamilselvan S

AU - Behera A

AU - Nair SK

AU - Chandru CSL

AU - Krishnakumar M

AU - Ramani P

DO - 10.4103/JIOH.JIOH_358_20

LA - en

N1 - FEMU ID: 48844; EMF-Portal URL: https://www.emf-portal.org/en/article/48844

SP - 350-355

TI - Micronuclei analysis in people residing within 25 m of radiation-exposed areas around mobile towers in Chennai, India: An observational study

ER -

TY - JOUR

IS - 1

JA - J Clin of Diagn Res

JO - Journal of Clinical and Diagnostic Research

PY - 2021

SN - 0973-709X

VL - 15

AU - D'Silva MH

AU - Swer RT

AU - Anbalagan J

AU - Bhargavan R

DO - 10.7860/JCDR/2021/47115.14441

LA - en

N1 - FEMU ID: 48839; EMF-Portal URL: https://www.emf-portal.org/en/article/48839

SP - AC01-AC04

TI - Assessment of DNA Damage in Chick Embryo Brains Exposed to 2G and 3G Cell Phone Radiation using Alkaline Comet Assay Technique

UR - https://jcdr.net/article_fulltext.asp?issn=0973-709x&year=2021&volume=15&issue=1&page=AC01&issn=0973-709x&id=14441

ER -

TY - JOUR

IS - 4

JA - Vet Arhiv

JO - Veterinarski Arhiv

PY - 2021

SN - 0372-5480

VL - 91

AU - Vilić M

AU - Žura Žaja I

AU - Tkalec M

AU - Štambuk A

AU - Šrut M

AU - Klobučar G

AU - Malarić K

AU - Tucak P

AU - Pašić S

AU - Tlak Gajger I

DO - 10.24099/vet.arhiv.1321

LA - en

N1 - FEMU ID: 45784; EMF-Portal URL: https://www.emf-portal.org/en/article/45784

SP - 427-435

TI - Effects of a radio frequency electromagnetic field on honey bee larvae (Apis mellifera) differ in relation to the experimental study design

UR - http://vetarhiv.vef.unizg.hr/papers/2021-91-4-9.pdf

ER -

TY - JOUR

IS - 10

JA - Int J Mol Sci

JO - International Journal of Molecular Sciences

PY - 2021

SN - 1422-0067

VL - 22

AU - Jin H

AU - Kim K

AU - Park GY

AU - Kim M

AU - Lee HJ

AU - Jeon S

AU - Kim JH

AU - Kim HR

AU - Lim KM

AU - Lee YS

DO - 10.3390/ijms22105134

LA - en

N1 - FEMU ID: 45005; EMF-Portal URL: https://www.emf-portal.org/en/article/45005

SP - 5134

TI - The Protective Effects of EMF-LTE against DNA Double-Strand Break Damage In Vitro and In Vivo

UR - https://www.mdpi.com/1422-0067/22/10/5134/pdf

ER -

TY - JOUR

JA - Environ Res

JO - Environmental Research

PY - 2021

SN - 0013-9351

VL - 196

AU - Zeni O

AU - Romeo S

AU - Sannino A

AU - Palumbo R

AU - Scarfì MR

DO - 10.1016/j.envres.2021.110935

LA - en

N1 - FEMU ID: 44355; EMF-Portal URL: https://www.emf-portal.org/en/article/44355

SP - 110935

TI - Evidence of bystander effect induced by radiofrequency radiation in a human neuroblastoma cell line

ER -

TY - JOUR

IS - 2

JA - Electromagn Biol Med

JO - Electromagnetic Biology and Medicine

PY - 2021

SN - 1536-8386

VL - 40

AU - Gunes M

AU - Ates K

AU - Yalcin B

AU - Akkurt S

AU - Ozen S

AU - Kaya B

DO - 10.1080/15368378.2021.1878210

LA - en

N1 - FEMU ID: 44328; EMF-Portal URL: https://www.emf-portal.org/en/article/44328

SP - 254-263

TI - An Evaluation of the Genotoxic Effects of Electromagnetic Radiation at 900 MHz, 1800 MHz, and 2100 MHz Frequencies with a SMART Assay in Drosophila melanogaster

ER -

TY - JOUR

JA - Environ Pollut

JO - Environmental Pollution

PY - 2020

SN - 0269-7491

VL - 267

AU - Gulati S

AU - Kosik P

AU - Durdik M

AU - Skorvaga M

AU - Jakl L

AU - Markova E

AU - Belyaev I

DO - 10.1016/j.envpol.2020.115632

LA - en

N1 - FEMU ID: 43792; EMF-Portal URL: https://www.emf-portal.org/en/article/43792

SP - 115632

TI - Effects of different mobile phone UMTS signals on DNA, apoptosis and oxidative stress in human lymphocytes

ER -

TY - JOUR

IS - 6

JA - Gen Physiol Biophys

JO - General Physiology and Biophysics

PY - 2020

SN - 0231-5882

VL - 39

AU - Panagopoulos DJ

DO - 10.4149/gpb_2020036

LA - en

N1 - FEMU ID: 43746; EMF-Portal URL: https://www.emf-portal.org/en/article/43746

SP - 531-544

TI - Comparing chromosome damage induced by mobile telephony radiation and a high caffeine dose: Effect of combination and exposure duration

ER -

TY - JOUR

IS - 5

JA - Neurol India

JO - Neurology India

PY - 2020

SN - 0028-3886

VL - 68

AU - Sharma A

AU - Shrivastava S

AU - Shukla S

DO - 10.4103/0028-3886.294554

LA - en

N1 - FEMU ID: 43574; EMF-Portal URL: https://www.emf-portal.org/en/article/43574

SP - 1092-1100

TI - Exposure of Radiofrequency Electromagnetic Radiation on Biochemical and Pathological Alterations

UR - https://www.neurologyindia.com/article.asp?issn=0028-3886;year=2020;volume=68;issue=5;spage=1092;epage=1100;aulast=Sharma

ER -

TY - JOUR

IS - 8

JO - Bioelectromagnetics

PY - 2020

SN - 0197-8462

VL - 41

AU - Lerchl A

AU - Klose M

AU - Drees (née Grote) K

DO - 10.1002/bem.22301

LA - en

N1 - FEMU ID: 43440; EMF-Portal URL: https://www.emf-portal.org/en/article/43440

SP - 611-616

TI - No Increased DNA Damage Observed in the Brain, Liver, and Lung of Fetal Mice Treated With Ethylnitrosourea and Exposed to UMTS Radiofrequency Electromagnetic Fields

ER -

TY - JOUR

IS - 19

JA - Int J Mol Sci

JO - International Journal of Molecular Sciences

PY - 2020

SN - 1422-0067

VL - 21

AU - Regalbuto E

AU - Anselmo A

AU - De Sanctis S

AU - Franchini V

AU - Lista F

AU - Benvenuto M

AU - Bei R

AU - Masuelli L

AU - D'Inzeo G

AU - Paffi A

AU - Trodella E

AU - Sgura A

DO - 10.3390/ijms21197069

LA - en

N1 - FEMU ID: 43389; EMF-Portal URL: https://www.emf-portal.org/en/article/43389

SP - E7069

TI - Human Fibroblasts In Vitro Exposed to 2.45 GHz Continuous and Pulsed Wave Signals: Evaluation of Biological Effects with a Multimethodological Approach

UR - https://www.mdpi.com/1422-0067/21/19/7069/pdf

ER -

TY - JOUR

JA - Sci Rep

JO - Scientific Reports

PY - 2020

SN - 2045-2322

VL - 10

AU - Choi J

AU - Min K

AU - Jeon S

AU - Kim N

AU - Pack JK

AU - Song K

DO - 10.1038/s41598-020-65732-4

LA - en

N1 - FEMU ID: 42704; EMF-Portal URL: https://www.emf-portal.org/en/article/42704

SP - 9238

TI - Continuous Exposure to 1.7 GHz LTE Electromagnetic Fields Increases Intracellular Reactive Oxygen Species to Decrease Human Cell Proliferation and Induce Senescence

UR - https://www.nature.com/articles/s41598-020-65732-4.pdf

ER -

TY - JOUR

IS - 4

JO - Genes

PY - 2020

SN - 2073-4425

VL - 11

AU - Schuermann D

AU - Ziemann C

AU - Barekati Z

AU - Capstick M

AU - Oertel A

AU - Focke F

AU - Murbach M

AU - Kuster N

AU - Dasenbrock C

AU - Schär P

DO - 10.3390/genes11040347

LA - en

N1 - FEMU ID: 42121; EMF-Portal URL: https://www.emf-portal.org/en/article/42121

SP - E347

TI - Assessment of Genotoxicity in Human Cells Exposed to Modulated Electromagnetic Fields of Wireless Communication Devices

UR - https://www.mdpi.com/2073-4425/11/4/347/pdf

ER -

TY - JOUR

JA - J Chem Neuroanat

JO - Journal of Chemical Neuroanatomy

PY - 2020

SN - 0891-0618

VL - 106

AU - Sharma S

AU - Shukla S

DO - 10.1016/j.jchemneu.2020.101784

LA - en

N1 - FEMU ID: 42061; EMF-Portal URL: https://www.emf-portal.org/en/article/42061

SP - 101784

TI - Effect of electromagnetic radiation on redox status, acetylcholine esterase activity and cellular damage contributing to the diminution of the brain working memory in rats

ER -

TY - JOUR

IS - 3

JO - Bioelectromagnetics

PY - 2020

SN - 0197-8462

VL - 41

AU - Yang H

AU - Zhang Y

AU - Wang Z

AU - Zhong S

AU - Hu G

AU - Zuo W

DO - 10.1002/bem.22255

LA - en

N1 - FEMU ID: 41784; EMF-Portal URL: https://www.emf-portal.org/en/article/41784

SP - 219-229

TI - The Effects of Mobile Phone Radiofrequency Radiation on Cochlear Stria Marginal Cells in Sprague-Dawley Rats

ER -

TY - JOUR

JA - Ecotoxicol Environ Saf

JO - Ecotoxicology and Environmental Safety

PY - 2020

SN - 0147-6513

VL - 188

AU - Kumar A

AU - Kaur S

AU - Chandel S

AU - Singh HP

AU - Batish DR

AU - Kohli RK

DO - 10.1016/j.ecoenv.2019.109786

LA - en

N1 - FEMU ID: 40396; EMF-Portal URL: https://www.emf-portal.org/en/article/40396

SP - 109786

TI - Comparative cyto- and genotoxicity of 900 MHz and 1800 MHz electromagnetic field radiations in root meristems of Allium cepa

ER -

TY - JOUR

IS - 2

JA - Environ Mol Mutagen

JO - Environmental and Molecular Mutagenesis

PY - 2020

SN - 0893-6692

VL - 61

AU - Smith-Roe SL

AU - Wyde ME

AU - Stout MD

AU - Winters JW

AU - Hobbs CA

AU - Shepard KG

AU - Green AS

AU - Kissling GE

AU - Shockley KR

AU - Tice RR

AU - Bucher JR

AU - Witt KL

DO - 10.1002/em.22343

LA - en

N1 - FEMU ID: 39949; EMF-Portal URL: https://www.emf-portal.org/en/article/39949

SP - 276-290

TI - Evaluation of the genotoxicity of cell phone radiofrequency radiation in male and female rats and mice following subchronic exposure

ER -

TY - JOUR

IS - 1

JA - IEEE J Electromagn RF Microw Med Biol

JO - IEEE Journal of Electromagnetics, RF and Microwaves in Medicine and Biology

PY - 2020

VL - 4

AU - Romeo S

AU - Sannino A

AU - Zeni O

AU - Angrisani L

AU - Massa R

AU - Scarfi MR

DO - 10.1109/JERM.2019.2918023

LA - en

N1 - FEMU ID: 38441; EMF-Portal URL: https://www.emf-portal.org/en/article/38441

SP - 17-23

TI - Effects of Radiofrequency Exposure and Co-Exposure on Human Lymphocytes: the Influence of Signal Modulation and Bandwidth

ER -

TY - JOUR

IS - 1

JA - Biotechnol Biotechnol Equip

JO - Biotechnology & Biotechnological Equipment

PY - 2019

SN - 1310-2818

VL - 33

AU - Alkis ME

AU - Akdag MZ

AU - Dasdag S

AU - Yegin K

AU - Akpolat V

DO - 10.1080/13102818.2019.1696702

LA - en

N1 - FEMU ID: 43430; EMF-Portal URL: https://www.emf-portal.org/en/article/43430

SP - 1733-1740

TI - Single-strand DNA breaks and oxidative changes in rat testes exposed to radiofrequency radiation emitted from cellular phones

UR - https://www.tandfonline.com/doi/abs/10.1080/13102818.2019.1696702?needAccess=true#aHR0cHM6Ly93d3cudGFuZGZvbmxpbmUuY29tL2RvaS9wZGYvMTAuMTA4MC8xMzEwMjgxOC4yMDE5LjE2OTY3MDI/bmVlZEFjY2Vzcz10cnVlQEBAMA==

ER -

TY - JOUR

JA - Sci Rep

JO - Scientific Reports

PY - 2019

SN - 2045-2322

VL - 9

AU - Houston BJ

AU - Nixon B

AU - McEwan KE

AU - Martin JH

AU - King BV

AU - Aitken RJ

AU - De Iuliis GN

DO - 10.1038/s41598-019-53983-9

LA - en

N1 - FEMU ID: 40773; EMF-Portal URL: https://www.emf-portal.org/en/article/40773

SP - 17478

TI - Whole-body exposures to radiofrequency-electromagnetic energy can cause DNA damage in mouse spermatozoa via an oxidative mechanism

UR - https://www.nature.com/articles/s41598-019-53983-9.pdf

ER -

TY - JOUR

JA - Sci Rep

JO - Scientific Reports

PY - 2019

SN - 2045-2322

VL - 9

AU - Durdik M

AU - Kosik P

AU - Markova E

AU - Somsedikova A

AU - Gajdosechova B

AU - Nikitina E

AU - Horvathova E

AU - Kozics K

AU - Davis D

AU - Belyaev I

DO - 10.1038/s41598-019-52389-x

LA - en

N1 - FEMU ID: 40421; EMF-Portal URL: https://www.emf-portal.org/en/article/40421

SP - 16182

TI - Microwaves from mobile phone induce reactive oxygen species but not DNA damage, preleukemic fusion genes and apoptosis in hematopoietic stem/progenitor cells

UR - https://www.nature.com/articles/s41598-019-52389-x.pdf

ER -

TY - JOUR

IS - 5

JA - Gen Physiol Biophys

JO - General Physiology and Biophysics

PY - 2019

SN - 0231-5882

VL - 38

AU - Panagopoulos DJ

DO - 10.4149/gpb_2019032

LA - en

N1 - FEMU ID: 39526; EMF-Portal URL: https://www.emf-portal.org/en/article/39526

SP - 445-454

TI - Chromosome damage in human cells induced by UMTS mobile telephony radiation

ER -

TY - JOUR

IS - 1

JA - J Environ Health Sci Eng

JO - Journal of Environmental Health Science & Engineering

PY - 2019

VL - 17

AU - Chandel S

AU - Kaur S

AU - Issa M

AU - Singh HP

AU - Batish DR

AU - Kohli RK

DO - 10.1007/s40201-018-00330-1

LA - en

N1 - FEMU ID: 38862; EMF-Portal URL: https://www.emf-portal.org/en/article/38862

SP - 97-104

TI - Exposure to mobile phone radiations at 2350 MHz incites cyto- and genotoxic effects in root meristems of Allium cepa

UR - https://link.springer.com/content/pdf/10.1007/s40201-018-00330-1.pdf

ER -

TY - JOUR

IS - 4

JA - J Radiat Res

JO - Journal of Radiation Research

PY - 2019

SN - 0449-3060

VL - 60

AU - Koyama S

AU - Narita E

AU - Suzuki Y

AU - Shiina T

AU - Taki M

AU - Shinohara N

AU - Miyakoshi J

DO - 10.1093/jrr/rrz017

LA - en

N1 - FEMU ID: 38589; EMF-Portal URL: https://www.emf-portal.org/en/article/38589

SP - 417-423

TI - Long-term exposure to a 40-GHz electromagnetic field does not affect genotoxicity or heat shock protein expression in HCE-T or SRA01/04 cells

UR - https://academic.oup.com/jrr/article-pdf/60/4/417/28964321/rrz017.pdf

ER -

TY - JOUR

IS - 5

JO - Protoplasma

PY - 2019

SN - 0033-183X

VL - 256

AU - Chandel S

AU - Kaur S

AU - Issa M

AU - Singh HP

AU - Batish DR

AU - Kohli RK

DO - 10.1007/s00709-019-01386-y

LA - en

N1 - FEMU ID: 38426; EMF-Portal URL: https://www.emf-portal.org/en/article/38426

SP - 1399-1407

TI - Appraisal of immediate and late effects of mobile phone radiations at 2100 MHz on mitotic activity and DNA integrity in root meristems of Allium cepa

ER -

TY - JOUR

JA - Environ Res

JO - Environmental Research

PY - 2019

SN - 0013-9351

VL - 174

AU - Jooyan N

AU - Goliaei B

AU - Bigdeli B

AU - Faraji-Dana R

AU - Zamani A

AU - Entezami M

AU - Mortazavi SMJ

DO - 10.1016/j.envres.2019.03.063

LA - en

N1 - FEMU ID: 38182; EMF-Portal URL: https://www.emf-portal.org/en/article/38182

SP - 176-187

TI - Direct and indirect effects of exposure to 900 MHz GSM radiofrequency electromagnetic fields on CHO cell line: Evidence of bystander effect by non-ionizing radiation

ER -

TY - JOUR

IS - 1

JA - Electromagn Biol Med

JO - Electromagnetic Biology and Medicine

PY - 2019

SN - 1536-8386

VL - 38

AU - Alkis ME

AU - Bilgin HM

AU - Akpolat V

AU - Dasdag S

AU - Yegin K

AU - Yavas MC

AU - Akdag MZ

DO - 10.1080/15368378.2019.1567526

LA - en

N1 - FEMU ID: 37161; EMF-Portal URL: https://www.emf-portal.org/en/article/37161

SP - 32-47

TI - Effect of 900-, 1800-, and 2100-MHz radiofrequency radiation on DNA and oxidative stress in brain

ER -

TY - JOUR

IS - 3

JA - Int J Clin Exp Med

JO - International Journal of Clinical and Experimental Medicine

PY - 2018

SN - 1940-5901

VL - 11

AU - Ding SS

AU - Sun P

AU - Tian H

AU - Huo YW

AU - Wang LR

AU - Han Y

AU - Zhang Z

AU - Liu X

AU - Xing JP

LA - en

N1 - FEMU ID: 49145; EMF-Portal URL: https://www.emf-portal.org/en/article/49145

SP - 2821-2830

TI - Association between daily exposure to electromagnetic radiation from 4G smartphone and 2.45-GHz wi-fi and oxidative damage to semen of males attending a genetics clinic: a primary study

UR - https://e-century.us/files/ijcem/11/3/ijcem0063001.pdf

ER -

TY - JOUR

IS - 6

JA - Int J Environ Sci Technol

JO - International Journal of Environmental Science and Technology

PY - 2018

SN - 1735-1472

VL - 15

AU - Răcuciu M

AU - Iftode C

AU - Miclăuş S

DO - 10.1007/s13762-017-1490-0

LA - en

N1 - FEMU ID: 49003; EMF-Portal URL: https://www.emf-portal.org/en/article/49003

SP - 1233-1242

TI - Influence of 1 GHz radiation at low specific absorption rate of energy deposition on plant mitotic division process

ER -

TY - JOUR

IS - 3

JA - J Entomol Sci

JO - Journal of Entomological Science

PY - 2018

SN - 0749-8004

VL - 53

AU - Shakina LA

AU - Kolchigin NN

AU - Shckorbatov YG

DO - 10.18474/JES17-80.1

LA - en

N1 - FEMU ID: 48572; EMF-Portal URL: https://www.emf-portal.org/en/article/48572

SP - 295-306

TI - Changes in Puffing Pattern of Drosophila melanogaster (Diptera: Drosophilidae) Polytene Chromosomes after Egg Exposure to Microwave Radiation and Magnetic Field

ER -

TY - JOUR

IS - 3

JA - J Oral Maxillofac Pathol

JO - Journal of Oral and Maxillofacial Pathology: JOMFP

PY - 2018

SN - 0973-029X

VL - 22

AU - Vanishree M

AU - Manvikar V

AU - Rudraraju A

AU - Reddy KMP

AU - Kumar NHP

AU - Quadri SJM

DO - 10.4103/jomfp.JOMFP_201_18

LA - en

N1 - FEMU ID: 37117; EMF-Portal URL: https://www.emf-portal.org/en/article/37117

SP - 448

TI - Significance of micronuclei in buccal smears of mobile phone users: A comparative study

UR - http://www.jomfp.in/article.asp?issn=0973-029X;year=2018;volume=22;issue=3;spage=448;epage=448;aulast=Vanishree

ER -

TY - JOUR

IS - 4

JA - Exp Oncol

JO - Experimental Oncology

PY - 2018

SN - 1812-9269

VL - 40

AU - Yakymenko I

AU - Burlaka A

AU - Tsybulin I

AU - Brieieva I

AU - Buchynska L

AU - Tsehmistrenko I

AU - Chekhun F

LA - en

N1 - FEMU ID: 36998; EMF-Portal URL: https://www.emf-portal.org/en/article/36998

SP - 282-287

TI - Oxidative and mutagenic effects of low intensity GSM 1800 MHz microwave radiation

UR - https://exp-oncology.com.ua/wp/wp-content/uploads/2018/12/2458.pdf?upload=

ER -

TY - GEN

ET - 11

PB - IEEE

PY - 2018

SN - 9781538638101

T2 - 2018 43rd International Conference on Infrared, Millimeter, and Terahertz Waves (IRMMW-THz), Nagoya, Japan

AU - Yamazaki S

AU - Harata M

AU - Idehara T

AU - Konagaya K

AU - Yokoyama G

AU - Hoshina H

AU - Ogawa Y

DO - 10.1109/IRMMW-THz.2018.8510110

LA - en

N1 - FEMU ID: 36567; EMF-Portal URL: https://www.emf-portal.org/en/article/36567

SP - 1-2

TI - Terahertz irradiation stimulates actin polymerization

ER -

TY - JOUR

JA - Front Public Health

JO - Frontiers in Public Health

PY - 2018

SN - 2296-2565

VL - 6

AU - Houston BJ

AU - Nixon B

AU - King BV

AU - Aitken RJ

AU - De Iuliis GN

DO - 10.3389/fpubh.2018.00270

LA - en

N1 - FEMU ID: 36249; EMF-Portal URL: https://www.emf-portal.org/en/article/36249

SP - 270

TI - Probing the Origins of 1,800 MHz Radio Frequency Electromagnetic Radiation Induced Damage in Mouse Immortalized Germ Cells and Spermatozoa in vitro

UR - https://www.frontiersin.org/articles/10.3389/fpubh.2018.00270/pdf

ER -

TY - JOUR

JA - Sci Rep

JO - Scientific Reports

PY - 2018

SN - 2045-2322

VL - 8

AU - Falone S

AU - Sannino A

AU - Romeo S

AU - Zeni O

AU - Santini SJ

AU - Rispoli R

AU - Amicarelli F

AU - Scarfì MR

DO - 10.1038/s41598-018-31636-7

LA - en

N1 - FEMU ID: 35984; EMF-Portal URL: https://www.emf-portal.org/en/article/35984

SP - 13234

TI - Protective effect of 1950 MHz electromagnetic field in human neuroblastoma cells challenged with menadione

UR - https://www.nature.com/articles/s41598-018-31636-7.pdf

ER -

TY - JOUR

IS - 1

JA - Cell Physiol Biochem

JO - Cellular Physiology and Biochemistry

PY - 2018

SN - 1015-8987

VL - 48

AU - Li R

AU - Ma M

AU - Li L

AU - Zhao L

AU - Zhang T

AU - Gao X

AU - Zhang D

AU - Zhu Y

AU - Peng Q

AU - Luo X

AU - Wang M

DO - 10.1159/000491660

LA - en

N1 - FEMU ID: 35470; EMF-Portal URL: https://www.emf-portal.org/en/article/35470

SP - 29-41

TI - The Protective Effect of Autophagy on DNA Damage in Mouse Spermatocyte-Derived Cells Exposed to 1800 MHz Radiofrequency Electromagnetic Fields

UR - https://www.karger.com/Article/Pdf/491660

ER -

TY - JOUR

IS - 1

JA - Health Phys

JO - Health Physics

PY - 2018

SN - 0017-9078

VL - 115

AU - Franchini V

AU - Regalbuto E

AU - De Amicis A

AU - De Sanctis S

AU - Di Cristofaro S

AU - Coluzzi E

AU - Marinaccio J

AU - Sgura A

AU - Ceccuzzi S

AU - Doria A

AU - Gallerano GP

AU - Giovenale E

AU - Ravera GL

AU - Bei R

AU - Benvenuto M

AU - Modesti A

AU - Masuelli L

AU - Lista F

DO - 10.1097/HP.0000000000000871

LA - en

N1 - FEMU ID: 35185; EMF-Portal URL: https://www.emf-portal.org/en/article/35185

SP - 126-139

TI - Genotoxic Effects in Human Fibroblasts Exposed to Microwave Radiation

ER -

TY - JOUR

IS - 2

JA - Electromagn Biol Med

JO - Electromagnetic Biology and Medicine

PY - 2018

SN - 1536-8386

VL - 37

AU - Akdag M

AU - Dasdag S

AU - Canturk F

AU - Akdag MZ

DO - 10.1080/15368378.2018.1463246

LA - en

N1 - FEMU ID: 34966; EMF-Portal URL: https://www.emf-portal.org/en/article/34966

SP - 66-75

TI - Exposure to non-ionizing electromagnetic fields emitted from mobile phones induced DNA damage in human ear canal hair follicle cells

ER -

TY - JOUR

IS - 4

JO - PLoS One

PY - 2018

SN - 1932-6203

VL - 13

AU - Al-Serori H

AU - Ferk F

AU - Kundi M

AU - Bileck A

AU - Gerner C

AU - Mišík M

AU - Nersesyan A

AU - Waldherr M

AU - Murbach M

AU - Lah TT

AU - Herold-Mende C

AU - Collins AR

AU - Knasmüller S

DO - 10.1371/journal.pone.0193677

LA - en

N1 - FEMU ID: 34934; EMF-Portal URL: https://www.emf-portal.org/en/article/34934

SP - e0193677

TI - Mobile phone specific electromagnetic fields induce transient DNA damage and nucleotide excision repair in serum-deprived human glioblastoma cells

UR - http://journals.plos.org/plosone/article/file?id=10.1371/journal.pone.0193677&type=printable

ER -

TY - JOUR

IS - 10

JA - Int J Radiat Biol

JO - International Journal of Radiation Biology

PY - 2018

SN - 0955-3002

VL - 94

AU - Herrala M

AU - Mustafa E

AU - Naarala J

AU - Juutilainen J

DO - 10.1080/09553002.2018.1450534

LA - en

N1 - FEMU ID: 34733; EMF-Portal URL: https://www.emf-portal.org/en/article/34733

SP - 883-889

TI - Assessment of genotoxicity and genomic instability in rat primary astrocytes exposed to 872 MHz radiofrequency radiation and chemicals

ER -

TY - JOUR

IS - 1

JA - Saudi J Biol Sci

JO - Saudi Journal of Biological Sciences

PY - 2018

SN - 1319-562X

VL - 25

AU - Salmen SH

AU - Alharbi SA

AU - Faden AA

AU - Wainwright M

DO - 10.1016/j.sjbs.2017.07.006

LA - en

N1 - FEMU ID: 34507; EMF-Portal URL: https://www.emf-portal.org/en/article/34507

SP - 105-110

TI - Evaluation of effect of high frequency electromagnetic field on growth and antibiotic sensitivity of bacteria

UR - https://www.sciencedirect.com/science/article/pii/S1319562X17301857/pdfft?md5=87c11cde1f2068ed16e842bed52ab9f1&pid=1-s2.0-S1319562X17301857-main.pdf

ER -

TY - JOUR

IS - 4

JA - J Apic Res

JO - Journal of Apicultural Research

PY - 2017

VL - 56

AU - Vilic M

AU - Gajger IT

AU - Tucak P

AU - Stambuk A

AU - Srut M

AU - Klobucar G

AU - Malaric K

AU - Zajaa IZ

AU - Pavelic A

AU - Manger M

AU - Tkalec M

DO - 10.1080/00218839.2017.1329798

LA - en

N1 - FEMU ID: 46075; EMF-Portal URL: https://www.emf-portal.org/en/article/46075

SP - 430-438

TI - Effects of short-term exposure to mobile phone radiofrequency (900 MHz) on the oxidative response and genotoxicity in honey bee larvae

ER -

TY - JOUR

JA - Data Brief

JO - Data in Brief

PY - 2017

SN - 2352-3409

VL - 15

AU - de Oliveira FM

AU - Carmona AM

AU - Ladeira C

DO - 10.1016/j.dib.2017.09.048

LA - en

N1 - FEMU ID: 34141; EMF-Portal URL: https://www.emf-portal.org/en/article/34141

SP - 344-347

TI - Genotoxicity assessment data for exfoliated buccal cells exposed to mobile phone radiation

UR - https://www.sciencedirect.com/science/article/pii/S2352340917304791/pdfft?md5=cf92857ee630b0a186210e76b366ce6d&pid=1-s2.0-S2352340917304791-main.pdf

ER -

TY - JOUR

IS - 7

JA - J Clin of Diagn Res

JO - Journal of Clinical and Diagnostic Research

PY - 2017

SN - 0973-709X

VL - 11

AU - D'Silva MH

AU - Swer RT

AU - Anbalagan J

AU - Rajesh B

DO - 10.7860/JCDR/2017/26360.10275

LA - en

N1 - FEMU ID: 33100; EMF-Portal URL: https://www.emf-portal.org/en/article/33100

SP - AC05-AC09

TI - Effect of Radiofrequency Radiation Emitted from 2G and 3G Cell Phone on Developing Liver of Chick Embryo - A Comparative Study

UR - https://www.ncbi.nlm.nih.gov/pmc/articles/PMC5583901/pdf/jcdr-11-AC05.pdf

ER -

TY - JOUR

JA - Mutat Res Genet Toxicol Environ Mutagen

JO - Mutation Research - Genetic Toxicology and Environmental Mutagenesis

PY - 2017

VL - 822

AU - de Oliveira FM

AU - Carmona AM

AU - Ladeira C

DO - 10.1016/j.mrgentox.2017.08.001

LA - en

N1 - FEMU ID: 32942; EMF-Portal URL: https://www.emf-portal.org/en/article/32942

SP - 41-46

TI - Is mobile phone radiation genotoxic? An analysis of micronucleus frequency in exfoliated buccal cells

ER -

TY - JOUR

IS - 13

JA - Ann Transl Med

JO - Annals of Translational Medicine

PY - 2017

SN - 2305-5839

VL - 5

AU - Danese E

AU - Lippi G

AU - Buonocore R

AU - Benati M

AU - Bovo C

AU - Bonaguri C

AU - Salvagno GL

AU - Brocco G

AU - Roggenbuck D

AU - Montagnana M

DO - 10.21037/atm.2017.04.35

LA - en

N1 - FEMU ID: 32623; EMF-Portal URL: https://www.emf-portal.org/en/article/32623

SP - 272

TI - Mobile phone radiofrequency exposure has no effect on DNA double strand breaks (DSB) in human lymphocytes

UR - https://www.ncbi.nlm.nih.gov/pmc/articles/PMC5515807/pdf/atm-05-13-272.pdf

ER -

TY - JOUR

JA - Mutat Res Genet Toxicol Environ Mutagen

JO - Mutation Research - Genetic Toxicology and Environmental Mutagenesis

PY - 2017

VL - 820

AU - He Q

AU - Zong L

AU - Sun Y

AU - Vijayalaxmi

AU - Prihoda TJ

AU - Tong J

AU - Cao Y

DO - 10.1016/j.mrgentox.2017.05.007

LA - en

N1 - FEMU ID: 32408; EMF-Portal URL: https://www.emf-portal.org/en/article/32408

SP - 19-25

TI - Adaptive response in mouse bone marrow stromal cells exposed to 900MHz radiofrequency fields: Impact of poly (ADP-ribose) polymerase (PARP)

ER -

TY - JOUR

IS - 2

JA - Arh Hig Rada Toksikol

JO - Arhiv za Higijenu Rada i Toksikologiju (Archives of Industrial Hygiene and Toxicology)

PY - 2017

SN - 0004-1254

VL - 68

AU - Bourdineaud JP

AU - Šrut M

AU - Štambuk A

AU - Tkalec M

AU - Brèthes D

AU - Malarić K

AU - Klobučar GIV

DO - 10.1515/aiht-2017-68-2928

LA - en

N1 - FEMU ID: 32367; EMF-Portal URL: https://www.emf-portal.org/en/article/32367

SP - 142-152

TI - Electromagnetic fields at a mobile phone frequency (900 MHz) trigger the onset of general stress response along with DNA modifications in Eisenia fetida earthworms

UR - https://sciendo.com/article/10.1515/aiht-2017-68-2928

ER -

TY - JOUR

IS - 6

JO - Bioelectromagnetics

PY - 2017

SN - 0197-8462

VL - 38

AU - Suzuki S

AU - Okutsu M

AU - Suganuma R

AU - Komiya H

AU - Nakatani-Enomoto S

AU - Kobayashi S

AU - Ugawa Y

AU - Tateno H

AU - Fujimori K

DO - 10.1002/bem.22063

LA - en

N1 - FEMU ID: 32274; EMF-Portal URL: https://www.emf-portal.org/en/article/32274

SP - 466-473

TI - Influence of radiofrequency-electromagnetic waves from 3rd-generation cellular phones on fertilization and embryo development in mice

ER -

TY - JOUR

IS - 4

JA - Saudi J Biol Sci

JO - Saudi Journal of Biological Sciences

PY - 2017

SN - 1319-562X

VL - 24

AU - Qureshi ST

AU - Memon SA

AU - Abassi AR

AU - Sial MA

AU - Bughio FA

DO - 10.1016/j.sjbs.2016.02.011

LA - en

N1 - FEMU ID: 31864; EMF-Portal URL: https://www.emf-portal.org/en/article/31864

SP - 883-891

TI - Radiofrequency radiations induced genotoxic and carcinogenic effects on chickpea (Cicer arietinum L.) root tip cells

UR - https://www.sciencedirect.com/science/article/pii/S1319562X16000589/pdfft?md5=275ab70a0cf42609a2a27cd618810be3&pid=1-s2.0-S1319562X16000589-main.pdf

ER -

TY - JOUR

IS - 1

JA - J Biomed Phys Eng

JO - Journal of Biomedical Physics & Engineering

PY - 2017

SN - 2251-7200

VL - 7

AU - Mokarram P

AU - Sheikhi M

AU - Mortazavi SMJ

AU - Saeb S

AU - Shokrpour N

LA - en

N1 - FEMU ID: 31805; EMF-Portal URL: https://www.emf-portal.org/en/article/31805

SP - 79-86

TI - Effect of Exposure to 900 MHz GSM Mobile Phone Radiofrequency Radiation on Estrogen Receptor Methylation Status in Colon Cells of Male Sprague Dawley Rats

UR - https://www.ncbi.nlm.nih.gov/pmc/articles/PMC5401136/pdf/JBPE-7-79.pdf

ER -

TY - JOUR

JO - Mutation Research - Fundamental and Molecular Mechanism of Mutagenesis

PY - 2017

SN - 0027-5107

VL - 797-799

AU - Sun Y

AU - Zong L

AU - Gao Z

AU - Zhu S

AU - Tong J

AU - Cao Y

DO - 10.1016/j.mrfmmm.2017.03.001

LA - en

N1 - FEMU ID: 31583; EMF-Portal URL: https://www.emf-portal.org/en/article/31583

SP - 7-14

TI - Mitochondrial DNA damage and oxidative damage in HL-60 cells exposed to 900MHz radiofrequency fields

ER -

TY - JOUR

JA - Toxicol In Vitro

JO - Toxicology in Vitro

PY - 2017

SN - 0887-2333

VL - 40

AU - Al-Serori H

AU - Kundi M

AU - Ferk F

AU - Mišík M

AU - Nersesyan A

AU - Murbach M

AU - Lah TT

AU - Knasmüller S

DO - 10.1016/j.tiv.2017.01.012

LA - en

N1 - FEMU ID: 31101; EMF-Portal URL: https://www.emf-portal.org/en/article/31101

SP - 264-271

TI - Evaluation of the potential of mobile phone specific electromagnetic fields (UMTS) to produce micronuclei in human glioblastoma cell lines

ER -

TY - JOUR

IS - 4

JO - Bioelectromagnetics

PY - 2017

SN - 0197-8462

VL - 38

AU - Sannino A

AU - Zeni O

AU - Romeo S

AU - Massa R

AU - Scarfi MR

DO - 10.1002/bem.22034

LA - en

N1 - FEMU ID: 30897; EMF-Portal URL: https://www.emf-portal.org/en/article/30897

SP - 245-254

TI - Adverse and beneficial effects in Chinese hamster lung fibroblast cells following radiofrequency exposure

ER -

TY - JOUR

IS - 3

JO - Bioelectromagnetics

PY - 2017

SN - 0197-8462

VL - 38

AU - Su L

AU - Wei X

AU - Xu Z

AU - Chen G

DO - 10.1002/bem.22032

LA - en

N1 - FEMU ID: 30787; EMF-Portal URL: https://www.emf-portal.org/en/article/30787

SP - 175-185

TI - RF-EMF exposure at 1800 MHz did not elicit DNA damage or abnormal cellular behaviors in different neurogenic cells

ER -

TY - JOUR

IS - 4

JA - Toxicol Ind Health

JO - Toxicology and Industrial Health

PY - 2017

SN - 0748-2337

VL - 33

AU - Pandey N

AU - Giri S

AU - Das S

AU - Upadhaya P

DO - 10.1177/0748233716671206

LA - en

N1 - FEMU ID: 30481; EMF-Portal URL: https://www.emf-portal.org/en/article/30481

SP - 373-384

TI - Radiofrequency radiation (900 MHz)-induced DNA damage and cell cycle arrest in testicular germ cells in swiss albino mice

ER -

TY - JOUR

IS - 8

JA - Int J Environ Res Public Health

JO - International Journal of Environmental Research and Public Health

PY - 2016

SN - 1660-4601

VL - 13

AU - Koyama S

AU - Narita E

AU - Shimizu Y

AU - Suzuki Y

AU - Shiina T

AU - Taki M

AU - Shinohara N

AU - Miyakoshi J

DO - 10.3390/ijerph13080802

LA - en

N1 - FEMU ID: 32667; EMF-Portal URL: https://www.emf-portal.org/en/article/32667

SP - E802

TI - Effects of Long-Term Exposure to 60 GHz Millimeter-Wavelength Radiation on the Genotoxicity and Heat Shock Protein (Hsp) Expression of Cells Derived from Human Eye

UR - https://www.ncbi.nlm.nih.gov/pmc/articles/PMC4997488/pdf/ijerph-13-00802.pdf

ER -

TY - JOUR

IS - 12

JA - Biomed Environ Sci

JO - Biomedical and Environmental Sciences

PY - 2016

SN - 0895-3988

VL - 29

AU - Deshmukh PS

AU - Megha K

AU - Nasare N

AU - Banerjee BD

AU - Ahmed RS

AU - Abegaonkar MP

AU - Tripathi AK

AU - Mediratta PK

DO - 10.3967/bes2016.115

LA - en

N1 - FEMU ID: 30925; EMF-Portal URL: https://www.emf-portal.org/en/article/30925

SP - 858-867

TI - Effect of Low Level Subchronic Microwave Radiation on Rat Brain

UR - https://www.sciencedirect.com/science/article/pii/S089539881730003X/pdf?md5=0d903e08b2bdd4758787ca791d1624bf&pid=1-s2.0-S089539881730003X-main.pdf

ER -

TY - JOUR

JA - Sci Rep

JO - Scientific Reports

PY - 2016

SN - 2045-2322

VL - 6

AU - Sun C

AU - Wei X

AU - Fei Y

AU - Su L

AU - Zhao X

AU - Chen G

AU - Xu Z

DO - 10.1038/srep37423

LA - en

N1 - FEMU ID: 30619; EMF-Portal URL: https://www.emf-portal.org/en/article/30619

SP - 37423

TI - Mobile phone signal exposure triggers a hormesis-like effect in Atm+/+ and Atm-/- mouse embryonic fibroblasts

UR - http://www.nature.com/articles/srep37423.pdf

ER -

TY - JOUR

IS - 5

JA - Radiat Res

JO - Radiation Research

PY - 2016

SN - 0033-7587

VL - 186

AU - Gläser K

AU - Rohland M

AU - Kleine-Ostmann T

AU - Schrader T

AU - Stopper H

AU - Hintzsche H

DO - 10.1667/RR14405.1

LA - en

N1 - FEMU ID: 30442; EMF-Portal URL: https://www.emf-portal.org/en/article/30442

SP - 455-465

TI - Effect of Radiofrequency Radiation on Human Hematopoietic Stem Cells

ER -

TY - JOUR

IS - 9

JO - PLoS One

PY - 2016

SN - 1932-6203

VL - 11

AU - Xing F

AU - Zhan Q

AU - He Y

AU - Cui J

AU - He S

AU - Wang G

DO - 10.1371/journal.pone.0163935

LA - en

N1 - FEMU ID: 30422; EMF-Portal URL: https://www.emf-portal.org/en/article/30422

SP - e0163935-

TI - 1800 MHz microwave induces p53 and p53-mediated caspase-3 activation leading to cell apoptosis in vitro

UR - https://journals.plos.org/plosone/article/file?id=10.1371/journal.pone.0163935&type=printable

ER -

TY - JOUR

IS - 8

JA - Int J Environ Res Public Health

JO - International Journal of Environmental Research and Public Health

PY - 2016

SN - 1660-4601

VL - 13

AU - Koyama S

AU - Narita E

AU - Shimizu Y

AU - Shiina T

AU - Taki M

AU - Shinohara N

AU - Miyakoshi J

DO - 10.3390/ijerph13080793

LA - en

N1 - FEMU ID: 30171; EMF-Portal URL: https://www.emf-portal.org/en/article/30171

SP - E793-

TI - Twenty four-hour exposure to a 0.12 THz electromagnetic field does not affect the genotoxicity, morphological changes, or expression of heat shock protein in HCE-T cells

UR - http://www.mdpi.com/1660-4601/13/8/793/pdf

ER -

TY - GEN

ET - 1

PB - IEEE

PY - 2016

SN - 9781509022687

T2 - 2016 9th International Kharkiv Symposium on Physics and Engineering of Microwaves, Millimeter and Submillimeter Waves (MSMW), Kharkiv

AU - Kuznetsov KA

AU - Miroshnik DB

AU - Shckorbatov YG

AU - Nikolov OT

AU - Kolchigin NN

DO - 10.1109/MSMW.2016.7538012

LA - en

N1 - FEMU ID: 30144; EMF-Portal URL: https://www.emf-portal.org/en/article/30144

SP - 1-3

TI - Modification of cellular effects of exposure to gamma-radiation by microwaves and magnetic field

ER -

TY - JOUR

JA - J Chem Neuroanat

JO - Journal of Chemical Neuroanatomy

PY - 2016

SN - 0891-0618

VL - 78

AU - Hussein S

AU - El-Saba AA

AU - Galal MK

DO - 10.1016/j.jchemneu.2016.07.009

LA - en

N1 - FEMU ID: 30004; EMF-Portal URL: https://www.emf-portal.org/en/article/30004

SP - 10-19

TI - Biochemical and histological studies on adverse effects of mobile phone radiation on rat's brain

ER -

TY - GEN

ET - 1

PB - IEEE

PY - 2016

SN - 9781467379861

T2 - 2016 IEEE Wireless Power Transfer Conference (WPTC), Aveiro, Portugal

AU - Toromura H

AU - Huang Y

AU - Koyama S

AU - Miyakoshi J

AU - Shinohara N

DO - 10.1109/WPT.2016.7498870

LA - en

N1 - FEMU ID: 29817; EMF-Portal URL: https://www.emf-portal.org/en/article/29817

SP - 1-3

TI - Biological effects of high-power microwave power transfer for electric vehicle

ER -

TY - JOUR

IS - 9-10

JA - J Toxicol Environ Health A

JO - Journal of Toxicology and Environmental Health, Part A

PY - 2016

SN - 0098-4108

VL - 79

AU - Ji Y

AU - He Q

AU - Sun Y

AU - Tong J

AU - Cao Y

DO - 10.1080/15287394.2016.1176618

LA - en

N1 - FEMU ID: 29624; EMF-Portal URL: https://www.emf-portal.org/en/article/29624

SP - 419-426

TI - Adaptive response in mouse bone-marrow stromal cells exposed to 900-MHz radiofrequency fields: Gamma-radiation-induced DNA strand breaks and repair

ER -

TY - JOUR

JA - Mutat Res Genet Toxicol Environ Mutagen

JO - Mutation Research - Genetic Toxicology and Environmental Mutagenesis

PY - 2016

VL - 803

AU - Sergeeva S

AU - Demidova E

AU - Sinitsyna O

AU - Goryachkovskaya T

AU - Bryanskaya A

AU - Semenov A

AU - Meshcheryakova I

AU - Dianov G

AU - Popik V

AU - Peltek S

DO - 10.1016/j.mrgentox.2016.05.005

LA - en

N1 - FEMU ID: 29619; EMF-Portal URL: https://www.emf-portal.org/en/article/29619

SP - 34-38

TI - 2.3THz radiation: Absence of genotoxicity/mutagenicity in Escherichia coli and Salmonella typhimurium

ER -

TY - JOUR

IS - 2

JA - Cell Biochem Biophys

JO - Cell Biochemistry and Biophysics

PY - 2016

SN - 1085-9195

VL - 74

AU - Kayhan H

AU - Esmekaya MA

AU - Saglam AS

AU - Tuysuz MZ

AU - Canseven AG

AU - Yagci AM

AU - Seyhan N

DO - 10.1007/s12013-016-0734-9

LA - en

N1 - FEMU ID: 29618; EMF-Portal URL: https://www.emf-portal.org/en/article/29618

SP - 99-107

TI - Does MW Radiation Affect Gene Expression, Apoptotic Level, and Cell Cycle Progression of Human SH-SY5Y Neuroblastoma Cells?

ER -

TY - JOUR

IS - 3

JA - J Clin of Diagn Res

JO - Journal of Clinical and Diagnostic Research

PY - 2016

SN - 0973-709X

VL - 10

AU - Banerjee S

AU - Singh NN

AU - Sreedhar G

AU - Mukherjee S

DO - 10.7860/JCDR/2016/17592.7505

LA - en

N1 - FEMU ID: 29426; EMF-Portal URL: https://www.emf-portal.org/en/article/29426

SP - ZC82-ZC85

TI - Analysis of the Genotoxic Effects of Mobile Phone Radiation using Buccal Micronucleus Assay: A Comparative Evaluation

UR - https://www.ncbi.nlm.nih.gov/pmc/articles/PMC4843394/pdf/jcdr-10-ZC82.pdf

ER -

TY - JOUR

IS - 3

JA - Saudi J Biol Sci

JO - Saudi Journal of Biological Sciences

PY - 2016

SN - 1319-562X

VL - 23

AU - Shahin-Jafari A

AU - Bayat M

AU - Shahhosseiny MH

AU - Tajik P

AU - Roudbar-Mohammadi S

DO - 10.1016/j.sjbs.2015.05.001

LA - en

N1 - FEMU ID: 29289; EMF-Portal URL: https://www.emf-portal.org/en/article/29289

SP - 426-433

TI - Effect of long-term exposure to mobile phone radiation on alpha-Int1 gene sequence of Candida albicans

UR - https://www.sciencedirect.com/science/article/pii/S1319562X15001023/pdfft?md5=d115cdd86917cb8a9bcc6e2d5c318361&pid=1-s2.0-S1319562X15001023-main.pdf

ER -

TY - JOUR

IS - 4

JA - Int J Radiat Biol

JO - International Journal of Radiation Biology

PY - 2016

SN - 0955-3002

VL - 92

AU - Dyka LD

AU - Shakina LA

AU - Strashnyuk VY

AU - Shckorbatov YG

DO - 10.3109/09553002.2016.1137105

LA - en

N1 - FEMU ID: 28868; EMF-Portal URL: https://www.emf-portal.org/en/article/28868

SP - 222-227

TI - Effects of 36.6 GHz and static magnetic field on degree of endoreduplication in Drosophila melanogaster polytene chromosomes

ER -

TY - JOUR

JA - J Chem Neuroanat

JO - Journal of Chemical Neuroanatomy

PY - 2016

SN - 0891-0618

VL - 75

AU - Akdag MZ

AU - Dasdag S

AU - Canturk F

AU - Karabulut D

AU - Caner Y

AU - Adalier N

DO - 10.1016/j.jchemneu.2016.01.003

LA - en

N1 - FEMU ID: 28659; EMF-Portal URL: https://www.emf-portal.org/en/article/28659

SP - 116-122

TI - Does prolonged radiofrequency radiation emitted from Wi-Fi devices induce DNA damage in various tissues of rats?

ER -

TY - JOUR

JA - J Chem Neuroanat

JO - Journal of Chemical Neuroanatomy

PY - 2016

SN - 0891-0618

VL - 75

AU - Sahin D

AU - Ozgur E

AU - Guler G

AU - Tomruk A

AU - Unlu I

AU - Sepici-Dincel A

AU - Seyhan N

DO - 10.1016/j.jchemneu.2016.01.002

LA - en

N1 - FEMU ID: 28658; EMF-Portal URL: https://www.emf-portal.org/en/article/28658

SP - 94-98

TI - The 2100 MHz radiofrequency radiation of a 3G-mobile phone and the DNA oxidative damage in brain

ER -

TY - JOUR

IS - 2

JA - Int J Radiat Biol

JO - International Journal of Radiation Biology

PY - 2016

SN - 0955-3002

VL - 92

AU - Silva V

AU - Hilly O

AU - Strenov Y

AU - Tzabari C

AU - Hauptman Y

AU - Feinmesser R

DO - 10.3109/09553002.2016.1117678

LA - en

N1 - FEMU ID: 28505; EMF-Portal URL: https://www.emf-portal.org/en/article/28505

SP - 107-115

TI - Effect of cell phone-like electromagnetic radiation on primary human thyroid cells

ER -

TY - JOUR

IS - 2

JO - Mutagenesis

PY - 2016

SN - 0267-8357

VL - 31

AU - Gustavino B

AU - Carboni G

AU - Petrillo R

AU - Paoluzzi G

AU - Santovetti E

AU - Rizzoni M

DO - 10.1093/mutage/gev071

LA - en

N1 - FEMU ID: 28074; EMF-Portal URL: https://www.emf-portal.org/en/article/28074

SP - 187-192

TI - Exposure to 915 MHz radiation induces micronuclei in Vicia faba root tips

UR - https://academic.oup.com/mutage/article-pdf/31/2/187/8178338/gev071.pdf

ER -

TY - JOUR

IS - 3

JA - Arch Environ Contam Toxicol

JO - Archives of Environmental Contamination and Toxicology

PY - 2016

SN - 0090-4341

VL - 70

AU - Gulati S

AU - Yadav A

AU - Kumar N

AU - Kanupriya

AU - Aggarwal NK

AU - Kumar R

AU - Gupta R

DO - 10.1007/s00244-015-0195-y

LA - en

N1 - FEMU ID: 27671; EMF-Portal URL: https://www.emf-portal.org/en/article/27671

SP - 615-625

TI - Effect of GSTM1 and GSTT1 polymorphisms on genetic damage in humans populations exposed to radiation from mobile towers

ER -

TY - JOUR

IS - 4

JA - Int J Hum Genet

JO - International Journal of Human Genetics

PY - 2015

SN - 0972-3757

VL - 15

AU - Gandhi G

AU - Singh P

AU - Kaur G

DO - 10.1080/09723757.2015.11886265

LA - en

N1 - FEMU ID: 47619; EMF-Portal URL: https://www.emf-portal.org/en/article/47619

SP - 173-182

TI - Perspectives Revisited - The Buccal Cytome Assay in Mobile Phone Users

UR - http://krepublishers.com/02-Journals/IJHG/IJHG-15-0-000-15-Web/IJHG-15-4-000-15-Abst-PDF/IJHG-15-4-173-15-606-Gandhi-G/IJHG-15-4-173-15-606-Gandhi-G-Tx[2].pdf

ER -

TY - JOUR

IS - 5

JO - Biophysics

PY - 2015

SN - 0006-3509

VL - 60

AU - Gapeyev AB

AU - Lukyanova NA

DO - 10.1134/S0006350915050061

LA - en

N1 - FEMU ID: 29340; EMF-Portal URL: https://www.emf-portal.org/en/article/29340

SP - 732-738

TI - Pulse-modulated extremely high-frequency electromagnetic radiation protects cellular DNA from the damaging effects of physical and chemical factors in vitro

ER -

TY - GEN

ET - 1

PB - IEEE

PY - 2015

SN - 9781424492718

T2 - 2015 37th Annual International Conference of the IEEE Engineering in Medicine and Biology Society (EMBC), Milan, Italy

AU - Moraitis N

AU - Christopoulou M

AU - Nikita KS

AU - Voulgaridou GP

AU - Anestopoulos I

AU - Panagiotidis MI

AU - Pappa A

DO - 10.1109/EMBC.2015.7318922

LA - en

N1 - FEMU ID: 28545; EMF-Portal URL: https://www.emf-portal.org/en/article/28545

SP - 2592-2595

TI - In-vitro assessment of Jurkat T-cells response to 1966 MHz electromagnetic fields in a GTEM cell

ER -

TY - JOUR

JA - Mutat Res Genet Toxicol Environ Mutagen

JO - Mutation Research - Genetic Toxicology and Environmental Mutagenesis

PY - 2015

VL - 793

AU - Amicis A

AU - Sanctis S

AU - Cristofaro SD

AU - Franchini V

AU - Lista F

AU - Regalbuto E

AU - Giovenale E

AU - Gallerano GP

AU - Nenzi P

AU - Bei R

AU - Fantini M

AU - Benvenuto M

AU - Masuelli L

AU - Coluzzi E

AU - Cicia C

AU - Sgura A

DO - 10.1016/j.mrgentox.2015.06.003

LA - en

N1 - FEMU ID: 28170; EMF-Portal URL: https://www.emf-portal.org/en/article/28170

SP - 150-160

TI - Biological effects of in vitro THz radiation exposure in human foetal fibroblasts

ER -

TY - JOUR

JO - Neurotoxicology

PY - 2015

SN - 0161-813X

VL - 51

AU - Megha K

AU - Deshmukh PS

AU - Banerjee BD

AU - Tripathi AK

AU - Ahmed R

AU - Abegaonkar MP

DO - 10.1016/j.neuro.2015.10.009

LA - en

N1 - FEMU ID: 28149; EMF-Portal URL: https://www.emf-portal.org/en/article/28149

SP - 158-165

TI - Low intensity microwave radiation induced oxidative stress, inflammatory response and DNA damage in rat brain

ER -

TY - JOUR

IS - 1

JA - Braz Oral Res

JO - Brazilian Oral Research

PY - 2015

SN - 1806-8324

VL - 29

AU - Daroit NB

AU - Visioli F

AU - Magnusson AS

AU - Vieira GR

AU - Rados PV

DO - 10.1590/1807-3107BOR-2015.vol29.0114

LA - en

N1 - FEMU ID: 28094; EMF-Portal URL: https://www.emf-portal.org/en/article/28094

SP - 1-8

TI - Cell phone radiation effects on cytogenetic abnormalities of oral mucosal cells

UR - http://www.scielo.br/pdf/bor/v29n1/1807-3107-bor-29-1-1807-3107BOR-2015vol290114.pdf

ER -

TY - JOUR

IS - 3

JA - Cell Physiol Biochem

JO - Cellular Physiology and Biochemistry

PY - 2015

SN - 1015-8987

VL - 37

AU - Wang X

AU - Liu C

AU - Ma Q

AU - Feng W

AU - Yang L

AU - Lu Y

AU - Zhou Z

AU - Yu Z

AU - Li W

AU - Zhang L

DO - 10.1159/000430233

LA - en

N1 - FEMU ID: 27920; EMF-Portal URL: https://www.emf-portal.org/en/article/27920

SP - 1075-1088

TI - 8-oxoG DNA Glycosylase-1 Inhibition Sensitizes Neuro-2a Cells to Oxidative DNA Base Damage Induced by 900 MHz Radiofrequency Electromagnetic Radiation

UR - https://www.karger.com/Article/Pdf/430233

ER -

TY - JOUR

IS - 11

JA - Int J Radiat Biol

JO - International Journal of Radiation Biology

PY - 2015

SN - 0955-3002

VL - 91

AU - Furtado-Filho OV

AU - Borba JB

AU - Maraschin T

AU - Souza LM

AU - Henriques JA

AU - Moreira JC

AU - Saffi J

DO - 10.3109/09553002.2015.1083629

LA - en

N1 - FEMU ID: 27729; EMF-Portal URL: https://www.emf-portal.org/en/article/27729

SP - 891-897

TI - Effects of chronic exposure to 950 MHz ultra-high-frequency electromagnetic radiation on reactive oxygen species metabolism in the right and left cerebral cortex of young rats of different ages

ER -

TY - JOUR

IS - 3

JA - Endocr Regul

JO - Endocrine Regulations

PY - 2015

SN - 1210-0668

VL - 49

AU - Gurbuz N

AU - Sirav B

AU - Kuzay D

AU - Ozer C

AU - Seyhan N

DO - 10.4149/endo_2015_03_126

LA - en

N1 - FEMU ID: 27666; EMF-Portal URL: https://www.emf-portal.org/en/article/27666

SP - 126-130

TI - Does radio frequency radiation induce micronuclei frequency in exfoliated bladder cells of diabetic rats?

ER -

TY - JOUR

JA - J Neuroinflammation

JO - Journal of Neuroinflammation

PY - 2015

SN - 1742-2094

VL - 12

AU - Zuo WQ

AU - Hu YJ

AU - Yang Y

AU - Zhao XY

AU - Zhang YY

AU - Kong W

AU - Kong WJ

DO - 10.1186/s12974-015-0300-1

LA - en

N1 - FEMU ID: 27276; EMF-Portal URL: https://www.emf-portal.org/en/article/27276

SP - 105

TI - Sensitivity of spiral ganglion neurons to damage caused by mobile phone electromagnetic radiation will increase in lipopolysaccharide-induced inflammation in vitro model

UR - https://www.ncbi.nlm.nih.gov/pmc/articles/PMC4458026/pdf/12974_2015_Article_300.pdf

ER -

TY - JOUR

IS - 8

JA - Int J Radiat Biol

JO - International Journal of Radiation Biology

PY - 2015

SN - 0955-3002

VL - 91

AU - Kumar G

AU - McIntosh RL

AU - Anderson V

AU - McKenzie RJ

AU - Wood AW

DO - 10.3109/09553002.2015.1047988

LA - en

N1 - FEMU ID: 27083; EMF-Portal URL: https://www.emf-portal.org/en/article/27083

SP - 664-672

TI - A genotoxic analysis of the hematopoietic system after mobile phone type radiation exposure in rats

ER -

TY - JOUR

IS - 1

JA - Int J Fertil Steril

JO - International Journal of Fertility & Sterility

PY - 2015

SN - 2008-0778

VL - 9

AU - Zalata A

AU - El-Samanoudy AZ

AU - Shaalan D

AU - El-Baiomy Y

AU - Mostafa T

DO - 10.22074/ijfs.2015.4217

LA - en

N1 - FEMU ID: 26969; EMF-Portal URL: https://www.emf-portal.org/en/article/26969

SP - 129-136

TI - In vitro effect of cell phone radiation on motility, DNA fragmentation and clusterin gene expression in human sperm

UR - https://www.ncbi.nlm.nih.gov/pmc/articles/PMC4410031/pdf/Int-J-Fertil-Steril-9-129.pdf

ER -

TY - JOUR

IS - 4

JA - Int J Environ Res Public Health

JO - International Journal of Environmental Research and Public Health

PY - 2015

SN - 1660-4601

VL - 12

AU - Mizuno K

AU - Shinohara N

AU - Miyakoshi J

DO - 10.3390/ijerph120403853

LA - en

N1 - FEMU ID: 26850; EMF-Portal URL: https://www.emf-portal.org/en/article/26850

SP - 3853-3863

TI - In Vitro Evaluation of Genotoxic Effects under Magnetic Resonant Coupling Wireless Power Transfer

UR - http://www.mdpi.com/1660-4601/12/4/3853

ER -

TY - JOUR

IS - 3

JA - Int J Toxicol

JO - International Journal of Toxicology

PY - 2015

SN - 1091-5818

VL - 34

AU - Deshmukh PS

AU - Nasare N

AU - Megha K

AU - Banerjee BD

AU - Ahmed RS

AU - Singh D

AU - Abegaonkar MP

AU - Tripathi AK

AU - Mediratta PK

DO - 10.1177/1091581815574348

LA - en

N1 - FEMU ID: 26650; EMF-Portal URL: https://www.emf-portal.org/en/article/26650

SP - 284-290

TI - Cognitive impairment and neurogenotoxic effects in rats exposed to low-intensity microwave radiation

ER -

TY - JOUR

IS - 3

JA - Radiat Res

JO - Radiation Research

PY - 2015

SN - 0033-7587

VL - 183

AU - Duan W

AU - Liu C

AU - Zhang L

AU - He M

AU - Xu S

AU - Chen C

AU - Pi H

AU - Gao P

AU - Zhang Y

AU - Zhong M

AU - Yu Z

AU - Zhou Z

DO - 10.1667/RR13851.1

LA - en

N1 - FEMU ID: 26541; EMF-Portal URL: https://www.emf-portal.org/en/article/26541

SP - 305-314

TI - Comparison of the genotoxic effects induced by 50 Hz extremely low-frequency electromagnetic fields and 1800 MHz radiofrequency electromagnetic fields in GC-2 cells

ER -

TY - JOUR

IS - 3

JA - Int J Radiat Biol

JO - International Journal of Radiation Biology

PY - 2015

SN - 0955-3002

VL - 91

AU - Zong C

AU - Ji Y

AU - He Q

AU - Zhu S

AU - Qin F

AU - Tong J

AU - Cao Y

DO - 10.3109/09553002.2014.980465

LA - en

N1 - FEMU ID: 25895; EMF-Portal URL: https://www.emf-portal.org/en/article/25895

SP - 270-276

TI - Adaptive response in mice exposed to 900 MHz radiofrequency fields: Bleomycin-induced DNA and oxidative damage/repair

ER -

TY - JOUR

IS - 1

JA - Electromagn Biol Med

JO - Electromagnetic Biology and Medicine

PY - 2015

SN - 1536-8386

VL - 34

AU - Hou Q

AU - Wang M

AU - Wu S

AU - Ma X

AU - An G

AU - Liu H

AU - Xie F

DO - 10.3109/15368378.2014.900507

LA - en

N1 - FEMU ID: 24552; EMF-Portal URL: https://www.emf-portal.org/en/article/24552

SP - 85-92

TI - Oxidative changes and apoptosis induced by 1800-MHz electromagnetic radiation in NIH/3T3 cells

ER -

TY - JOUR

IS - 3

JO - Rare Tumors

PY - 2014

SN - 2036-3605

VL - 6

AU - Akhavan-Sigari R

AU - Baf MM

AU - Ariabod V

AU - Rohde V

AU - Rahighi S

DO - 10.4081/rt.2014.5350

LA - en

N1 - FEMU ID: 25830; EMF-Portal URL: https://www.emf-portal.org/en/article/25830

SP - 5350

TI - Connection between Cell Phone use, p53 Gene Expression in Different Zones of Glioblastoma Multiforme and Survival Prognoses

UR - https://www.ncbi.nlm.nih.gov/pmc/articles/PMC4178273/pdf/rt-2014-3-5350.pdf

ER -

TY - JOUR

IS - 9

JA - Indian J Exp Biol

JO - Indian Journal of Experimental Biology

PY - 2014

SN - 0019-5189

VL - 52

AU - Kumar S

AU - Nirala JP

AU - Behari J

AU - Paulraj R

LA - en

N1 - FEMU ID: 25773; EMF-Portal URL: https://www.emf-portal.org/en/article/25773

SP - 890-897

TI - Effect of electromagnetic irradiation produced by 3G mobile phone on male rat reproductive system in a simulated scenario

UR - http://nopr.niscpr.res.in/bitstream/123456789/29335/1/IJEB%2052%289%29%20890-897.pdf

ER -

TY - JOUR

IS - 1

JA - Cent European J Urol

JO - Central European Journal of Urology

PY - 2014

SN - 2080-4806

VL - 67

AU - Gorpinchenko I

AU - Nikitin O

AU - Banyra O

AU - Shulyak A

DO - 10.5173/ceju.2014.01.art14

LA - en

N1 - FEMU ID: 25254; EMF-Portal URL: https://www.emf-portal.org/en/article/25254

SP - 65-71

TI - The influence of direct mobile phone radiation on sperm quality

UR - http://ceju.online/journal/2014/commenting-on-gorpinchenko-et-al-the-influence-of-direct-mobile-phone-radiation-on-sperm-439.php

ER -

TY - JOUR

IS - 10

JA - Int J Radiat Biol

JO - International Journal of Radiation Biology

PY - 2014

SN - 0955-3002

VL - 90

AU - Gürler HS

AU - Bilgici B

AU - Akar AK

AU - Tomak L

AU - Bedir A

DO - 10.3109/09553002.2014.922717

LA - en

N1 - FEMU ID: 24955; EMF-Portal URL: https://www.emf-portal.org/en/article/24955

SP - 892-896

TI - Increased DNA oxidation (8-OHdG) and protein oxidation (AOPP) by Low level electromagnetic field (2.45 GHz) in rat brain and protective effect of garlic

ER -

TY - JOUR

IS - 4

JA - Electromagn Biol Med

JO - Electromagnetic Biology and Medicine

PY - 2014

SN - 1536-8386

VL - 33

AU - Gurbuz N

AU - Sirav B

AU - Colbay M

AU - Yetkin I

AU - Seyhan N

DO - 10.3109/15368378.2013.831354

LA - en

N1 - FEMU ID: 23927; EMF-Portal URL: https://www.emf-portal.org/en/article/23927

SP - 296-301

TI - No genotoxic effect in exfoliated bladder cells of rat under the exposure of 1800 and 2100 MHz radio frequency radiation

ER -

TY - JOUR

IS - 2

JA - J Radiat Res

JO - Journal of Radiation Research

PY - 2014

SN - 0449-3060

VL - 55

AU - Sannino A

AU - Zeni O

AU - Romeo S

AU - Massa R

AU - Gialanella G

AU - Grossi G

AU - Manti L

AU - Vijayalaxmi

AU - Scarfi MR

DO - 10.1093/jrr/rrt106

LA - en

N1 - FEMU ID: 23403; EMF-Portal URL: https://www.emf-portal.org/en/article/23403

SP - 210-217

TI - Adaptive response in human blood lymphocytes exposed to non-ionizing radiofrequency fields: resistance to ionizing radiation-induced damage

UR - https://academic.oup.com/jrr/article-pdf/55/2/210/2797100/rrt106.pdf

ER -

TY - JOUR

IS - 2

JA - Cell Biochem Biophys

JO - Cell Biochemistry and Biophysics

PY - 2014

SN - 1085-9195

VL - 68

AU - Kesari KK

AU - Meena R

AU - Nirala J

AU - Kumar J

AU - Verma HN

DO - 10.1007/s12013-013-9715-4

LA - en

N1 - FEMU ID: 23361; EMF-Portal URL: https://www.emf-portal.org/en/article/23361

SP - 347-358

TI - Effect of 3G Cell Phone Exposure with Computer Controlled 2-D Stepper Motor on Non-thermal Activation of the hsp27/p38MAPK Stress Pathway in Rat Brain

ER -

TY - JOUR

IS - 2

JA - Int J Radiat Biol

JO - International Journal of Radiation Biology

PY - 2014

SN - 0955-3002

VL - 90

AU - Furtado-Filho OV

AU - Borba JB

AU - Dallegrave A

AU - Pizzolato TM

AU - Henriques JA

AU - Moreira JC

AU - Saffi J

DO - 10.3109/09553002.2013.817697

LA - en

N1 - FEMU ID: 22969; EMF-Portal URL: https://www.emf-portal.org/en/article/22969

SP - 159-168

TI - Effect of 950 MHz UHF Electromagnetic radiation on biomarkers of oxidative damage, metabolism of UFA and antioxidants in the liver of young rats of different ages

ER -

TY - JOUR

IS - 2

JA - Electromagn Biol Med

JO - Electromagnetic Biology and Medicine

PY - 2014

SN - 1536-8386

VL - 33

AU - Souza LCM

AU - Cerqueira Ede M

AU - Meireles JR

DO - 10.3109/15368378.2013.783856

LA - en

N1 - FEMU ID: 22645; EMF-Portal URL: https://www.emf-portal.org/en/article/22645

SP - 98-102

TI - Assessment of nuclear abnormalities in exfoliated cells from the oral epithelium of mobile phone users

ER -

TY - JOUR

IS - 2

JA - Electromagn Biol Med

JO - Electromagnetic Biology and Medicine

PY - 2014

SN - 1536-8386

VL - 33

AU - Meena R

AU - Kumari K

AU - Kumar J

AU - Rajamani P

AU - Verma HN

AU - Kesari KK

DO - 10.3109/15368378.2013.781035

LA - en

N1 - FEMU ID: 22452; EMF-Portal URL: https://www.emf-portal.org/en/article/22452

SP - 81-91

TI - Therapeutic approaches of melatonin in microwave radiations-induced oxidative stress-mediated toxicity on male fertility pattern of Wistar rats

ER -

TY - JOUR

IS - 11

JO - Zahedan Journal of Research in Medical Sciences

PY - 2013

SN - 2383-2894

VL - 15

AU - Zahedifar Z

AU - Baharara J

LA - en

N1 - FEMU ID: 39150; EMF-Portal URL: https://www.emf-portal.org/en/article/39150

SP - 39-44

TI - Effect of Green Tea Extract in Reducing Genotoxic Injuries of Cell Phone Microwaves on Bone Marrow

UR - https://admin.kowsarpub.com/cdn/dl/28fbf5fa-6ccf-11e9-b7d0-8329d5be8d6a

ER -

TY - JOUR

IS - 11

JA - J Endocrinol Invest

JO - Journal of Endocrinological Investigation

PY - 2013

SN - 0391-4097

VL - 36

AU - Rago R

AU - Salacone P

AU - Caponecchia L

AU - Sebastianelli A

AU - Marcucci I

AU - Calogero AE

AU - Condorelli R

AU - Vicari E

AU - Morgia G

AU - Favilla V

AU - Cimino S

AU - Arcoria AF

AU - La Vignera S

DO - 10.3275/8996

LA - en

N1 - FEMU ID: 25784; EMF-Portal URL: https://www.emf-portal.org/en/article/25784

SP - 970-974

TI - The semen quality of the mobile phone users

ER -

TY - JOUR

JA - Reprod Toxicol

JO - Reproductive Toxicology

PY - 2013

SN - 0890-6238

VL - 42

AU - Hanci H

AU - Odaci E

AU - Kaya H

AU - Aliyazicioglu Y

AU - Turan I

AU - Demir S

AU - Colakoglu S

DO - 10.1016/j.reprotox.2013.09.006

LA - en

N1 - FEMU ID: 23648; EMF-Portal URL: https://www.emf-portal.org/en/article/23648

SP - 203-209

TI - The effect of prenatal exposure to 900-megahertz electromagnetic field on the 21-old-day rat testicle

ER -

TY - JOUR

IS - 3

JA - Exp Oncol

JO - Experimental Oncology

PY - 2013

SN - 1812-9269

VL - 35

AU - Burlaka A

AU - Tsybulin O

AU - Sidorik E

AU - Lukin S

AU - Polishuk V

AU - Tsehmistrenko S

AU - Yakymenko I

LA - en

N1 - FEMU ID: 23628; EMF-Portal URL: https://www.emf-portal.org/en/article/23628

SP - 219-225

TI - Overproduction of free radical species in embryonal cells exposed to low intensity radiofrequency radiation

UR - https://exp-oncology.com.ua/article/6079

ER -

TY - JOUR

IS - 11

JA - Int J Radiat Biol

JO - International Journal of Radiation Biology

PY - 2013

SN - 0955-3002

VL - 89

AU - Liu C

AU - Gao P

AU - Xu SC

AU - Wang Y

AU - Chen CH

AU - He MD

AU - Yu ZP

AU - Zhang L

AU - Zhou Z

DO - 10.3109/09553002.2013.811309

LA - en

N1 - FEMU ID: 23360; EMF-Portal URL: https://www.emf-portal.org/en/article/23360

SP - 993-1001

TI - Mobile phone radiation induces mode-dependent DNA damage in a mouse spermatocyte-derived cell line: a protective role of melatonin

ER -

TY - JOUR

IS - 2

JO - Dose Response

PY - 2013

SN - 1559-3258

VL - 11

AU - Mortazavi S

AU - Mosleh-Shirazi M

AU - Tavassoli A

AU - Taheri M

AU - Mehdizadeh A

AU - Namazi S

AU - Jamali A

AU - Ghalandari R

AU - Bonyadi S

AU - Haghani M

AU - Shafie M

DO - 10.2203/dose-response.12-010.Mortazavi

LA - en

N1 - FEMU ID: 23318; EMF-Portal URL: https://www.emf-portal.org/en/article/23318

SP - 281-292

TI - Increased Radioresistance to Lethal Doses of Gamma Rays in Mice and Rats after Exposure to Microwave Radiation Emitted by a GSM Mobile Phone Simulator

UR - https://www.ncbi.nlm.nih.gov/pmc/articles/PMC3682203/pdf/drp-11-281.pdf

ER -

TY - JOUR

IS - 1

JA - Toxicol Int

JO - Toxicology International

PY - 2013

SN - 0971-6580

VL - 20

AU - Deshmukh PS

AU - Megha K

AU - Banerjee BD

AU - Ahmed RS

AU - Chandna S

AU - Abegaonkar MP

AU - Tripathi AK

DO - 10.4103/0971-6580.111549

LA - en

N1 - FEMU ID: 22984; EMF-Portal URL: https://www.emf-portal.org/en/article/22984

SP - 19-24

TI - Detection of Low Level Microwave Radiation Induced Deoxyribonucleic Acid Damage Vis-a-vis Genotoxicity in Brain of Fischer Rats

UR - https://www.ncbi.nlm.nih.gov/pmc/articles/PMC3702122/?report=printable

ER -

TY - JOUR

IS - 2

JA - Mutat Res Genet Toxicol Environ Mutagen

JO - Mutation Research - Genetic Toxicology and Environmental Mutagenesis

PY - 2013

VL - 755

AU - Speit G

AU - Gminski R

AU - Tauber R

DO - 10.1016/j.mrgentox.2013.06.014

LA - en

N1 - FEMU ID: 22960; EMF-Portal URL: https://www.emf-portal.org/en/article/22960

SP - 163-166

TI - Genotoxic effects of exposure to radiofrequency electromagnetic fields (RF-EMF) in HL-60 cells are not reproducible

ER -

TY - JOUR

IS - 7

JO - Bioelectromagnetics

PY - 2013

SN - 0197-8462

VL - 34

AU - Vijayalaxmi

AU - Reddy AB

AU - McKenzie RJ

AU - McIntosh RL

AU - Prihoda TJ

AU - Wood AW

DO - 10.1002/bem.21798

LA - en

N1 - FEMU ID: 22648; EMF-Portal URL: https://www.emf-portal.org/en/article/22648

SP - 542-548

TI - Incidence of micronuclei in human peripheral blood lymphocytes exposed to modulated and unmodulated 2450 MHz radiofrequency fields

ER -

TY - JOUR

IS - 11

JA - Int J Radiat Biol

JO - International Journal of Radiation Biology

PY - 2013

SN - 0955-3002

VL - 89

AU - Atli Sekeroglu Z

AU - Akar A

AU - Sekeroglu V

DO - 10.3109/09553002.2013.809170

LA - en

N1 - FEMU ID: 22646; EMF-Portal URL: https://www.emf-portal.org/en/article/22646

SP - 985-992

TI - Evaluation of the cytogenotoxic damage in immature and mature rats exposed to 900 MHz radiofrequency electromagnetic fields

ER -

TY - JOUR

IS - 10

JA - Int J Radiat Biol

JO - International Journal of Radiation Biology

PY - 2013

SN - 0955-3002

VL - 89

AU - Szerencsi A

AU - Kubinyi G

AU - Valiczko E

AU - Juhasz P

AU - Rudas G

AU - Mester A

AU - Janossy G

AU - Bakos J

AU - Thuroczy G

DO - 10.3109/09553002.2013.804962

LA - en

N1 - FEMU ID: 22464; EMF-Portal URL: https://www.emf-portal.org/en/article/22464

SP - 870-876

TI - DNA Integrity of Human Leukocytes after Magnetic Resonance Imaging

ER -

TY - JOUR

IS - 9

JA - Int J Radiat Biol

JO - International Journal of Radiation Biology

PY - 2013

SN - 0955-3002

VL - 89

AU - Tsybulin O

AU - Sidorik E

AU - Brieieva O

AU - Buchynska L

AU - Kyrylenko S

AU - Henshel D

AU - Yakymenko I

DO - 10.3109/09553002.2013.791408

LA - en

N1 - FEMU ID: 22134; EMF-Portal URL: https://www.emf-portal.org/en/article/22134

SP - 756-763

TI - GSM 900 MHz cellular phone radiation can either stimulate or depress early embryogenesis in Japanese quails depending on the duration of exposure

ER -

TY - JOUR

IS - 1

JA - Gen Physiol Biophys

JO - General Physiology and Biophysics

PY - 2013

SN - 0231-5882

VL - 32

AU - Sokolovic D

AU - Djordjevic B

AU - Kocic G

AU - Veljkovic A

AU - Marinkovic M

AU - Basic J

AU - Jevtovic-Stoimenov T

AU - Stanojkovic Z

AU - Sokolovic DM

AU - Pavlovic V

AU - Djindjic B

AU - Krstic D

DO - 10.4149/gpb_2013002

LA - en

N1 - FEMU ID: 21991; EMF-Portal URL: https://www.emf-portal.org/en/article/21991

SP - 79-90

TI - Melatonin protects rat thymus against oxidative stress caused by exposure to microwaves and modulates proliferation/apoptosis of thymocytes

ER -

TY - JOUR

JA - Ecotoxicol Environ Saf

JO - Ecotoxicology and Environmental Safety

PY - 2013

SN - 0147-6513

VL - 90

AU - Tkalec M

AU - Stambuk A

AU - Srut M

AU - Malaric K

AU - Klobucar GI

DO - 10.1016/j.ecoenv.2012.12.005

LA - en

N1 - FEMU ID: 21702; EMF-Portal URL: https://www.emf-portal.org/en/article/21702

SP - 7-12

TI - Oxidative and genotoxic effects of 900 MHz electromagnetic fields in the earthworm Eisenia fetida

ER -

TY - JOUR

IS - 1

JO - PLoS One

PY - 2013

SN - 1932-6203

VL - 8

AU - Xu S

AU - Chen G

AU - Chen C

AU - Sun C

AU - Zhang D

AU - Murbach M

AU - Kuster N

AU - Zeng Q

AU - Xu Z

DO - 10.1371/journal.pone.0054906

LA - en

N1 - FEMU ID: 21701; EMF-Portal URL: https://www.emf-portal.org/en/article/21701

SP - e54906

TI - Cell Type-Dependent Induction of DNA Damage by 1800 MHz Radiofrequency Electromagnetic Fields Does Not Result in Significant Cellular Dysfunctions

UR - http://journals.plos.org/plosone/article?id=10.1371/journal.pone.0054906

ER -

TY - JOUR

IS - 1

JA - Toxicol Lett

JO - Toxicology Letters

PY - 2013

SN - 0378-4274

VL - 218

AU - Liu C

AU - Duan W

AU - Xu S

AU - Chen C

AU - He M

AU - Zhang L

AU - Yu Z

AU - Zhou Z

DO - 10.1016/j.toxlet.2013.01.003

LA - en

N1 - FEMU ID: 21674; EMF-Portal URL: https://www.emf-portal.org/en/article/21674

SP - 2-9

TI - Exposure to 1800 MHz radiofrequency electromagnetic radiation induces oxidative DNA base damage in a mouse spermatocyte-derived cell line

ER -

TY - JOUR

IS - 2

JA - Radiat Res

JO - Radiation Research

PY - 2013

SN - 0033-7587

VL - 179

AU - Waldmann P

AU - Bohnenberger S

AU - Greinert R

AU - Hermann-Then B

AU - Heselich A

AU - Klug SJ

AU - Koenig J

AU - Kuhr K

AU - Kuster N

AU - Merker M

AU - Murbach M

AU - Pollet D

AU - Schadenboeck W

AU - Scheidemann-Wesp U

AU - Schwab B

AU - Volkmer B

AU - Weyer V

AU - Blettner M

DO - 10.1667/RR2914.1

LA - en

N1 - FEMU ID: 21641; EMF-Portal URL: https://www.emf-portal.org/en/article/21641

SP - 243-253

TI - Influence of GSM Signals on Human Peripheral Lymphocytes: Study of Genotoxicity

ER -

TY - JOUR

IS - 2

JA - Mutat Res Genet Toxicol Environ Mutagen

JO - Mutation Research - Genetic Toxicology and Environmental Mutagenesis

PY - 2013

VL - 751

AU - Jiang B

AU - Zong C

AU - Zhao H

AU - Ji Y

AU - Tong J

AU - Cao Y

DO - 10.1016/j.mrgentox.2012.12.003

LA - en

N1 - FEMU ID: 21616; EMF-Portal URL: https://www.emf-portal.org/en/article/21616

SP - 127-129

TI - Induction of adaptive response in mice exposed to 900MHz radiofrequency fields: application of micronucleus assay

ER -

TY - JOUR

JA - Ecotoxicol Environ Saf

JO - Ecotoxicology and Environmental Safety

PY - 2013

SN - 0147-6513

VL - 88

AU - Hekmat A

AU - Saboury AA

AU - Moosavi-Movahedi AA

DO - 10.1016/j.ecoenv.2012.10.016

LA - en

N1 - FEMU ID: 21475; EMF-Portal URL: https://www.emf-portal.org/en/article/21475

SP - 35-41

TI - The toxic effects of mobile phone radiofrequency (940 MHz) on the structure of calf thymus DNA

ER -

TY - JOUR

IS - 3

JA - Int J Radiat Biol

JO - International Journal of Radiation Biology

PY - 2013

SN - 0955-3002

VL - 89

AU - Kumar S

AU - Behari J

AU - Sisodia R

DO - 10.3109/09553002.2013.741282

LA - en

N1 - FEMU ID: 21367; EMF-Portal URL: https://www.emf-portal.org/en/article/21367

SP - 147-154

TI - Influence of electromagnetic fields on reproductive system of male rats

ER -

TY - JOUR

IS - 1-2

JA - Mutat Res Genet Toxicol Environ Mutagen

JO - Mutation Research - Genetic Toxicology and Environmental Mutagenesis

PY - 2013

VL - 750

AU - Pesnya DS

AU - Romanovsky AV

DO - 10.1016/j.mrgentox.2012.08.010

LA - en

N1 - FEMU ID: 21320; EMF-Portal URL: https://www.emf-portal.org/en/article/21320

SP - 27-33

TI - Comparison of cytotoxic and genotoxic effects of plutonium-239 alpha particles and mobile phone GSM 900 radiation in the Allium cepa test

ER -

TY - JOUR

IS - 2

JA - J Pediatr Urol

JO - Journal of Pediatric Urology

PY - 2013

SN - 1477-5131

VL - 9

AU - Atasoy HI

AU - Gunal MY

AU - Atasoy P

AU - Elgun S

AU - Bugdayci G

DO - 10.1016/j.jpurol.2012.02.015

LA - en

N1 - FEMU ID: 20439; EMF-Portal URL: https://www.emf-portal.org/en/article/20439

SP - 223-229

TI - Immunohistopathologic demonstration of deleterious effects on growing rat testes of radiofrequency waves emitted from conventional Wi-Fi devices

ER -

TY - JOUR

IS - 4

JA - Nepal Med Coll J

JO - Nepal Medical College Journal

PY - 2012

SN - 2676-1319

VL - 14

AU - Ingole IV

AU - Ghosh SK

LA - en

N1 - FEMU ID: 49582; EMF-Portal URL: https://www.emf-portal.org/en/article/49582

SP - 337-341

TI - Effect of exposure to radio frequency radiation emitted by cell phone on the developing dorsal root ganglion of chick embryo: a light microscopic study

UR - https://nmcth.edu/images/gallery/Original%20Articles/KM56PIV%20Ingole.pdf

ER -

TY - JOUR

IS - 2

JA - J Appl Pharm Sci

JO - Journal of Applied Pharmaceutical Science

PY - 2012

SN - 2231-3354

VL - 2

AU - El-Abd SF

AU - Eltoweissy MY

LA - en

N1 - FEMU ID: 47219; EMF-Portal URL: https://www.emf-portal.org/en/article/47219

SP - 16-20

TI - Cytogenetic alterations in human lymphocyte culture following exposure to radiofrequency field of mobile phone

UR - https://www.japsonline.com/admin/php/uploads/372_pdf.pdf

ER -

TY - JOUR

JA - Oxid Med Cell Longev

JO - Oxidative Medicine and Cellular Longevity

PY - 2012

SN - 1942-0994

AU - Lu YS

AU - Huang BT

AU - Huang YX

DO - 10.1155/2012/740280

LA - en

N1 - FEMU ID: 20976; EMF-Portal URL: https://www.emf-portal.org/en/article/20976

SP - 740280

TI - Reactive Oxygen Species Formation and Apoptosis in Human Peripheral Blood Mononuclear Cell Induced by 900 MHz Mobile Phone Radiation

UR - https://www.hindawi.com/journals/oximed/2012/740280/

ER -

TY - JOUR

IS - 8

JA - Oral Dis

JO - Oral Diseases

PY - 2012

SN - 1354-523X

VL - 18

AU - Ros-Llor I

AU - Sanchez-Siles M

AU - Camacho-Alonso F

AU - Lopez-Jornet P

DO - 10.1111/j.1601-0825.2012.01946.x

LA - en

N1 - FEMU ID: 20877; EMF-Portal URL: https://www.emf-portal.org/en/article/20877

SP - 786-792

TI - Effect of mobile phones on micronucleus frequency in human exfoliated oral mucosal cells

ER -

TY - JOUR

IS - 1

JA - Mutat Res Genet Toxicol Environ Mutagen

JO - Mutation Research - Genetic Toxicology and Environmental Mutagenesis

PY - 2012

VL - 747

AU - Zeni O

AU - Sannino A

AU - Romeo S

AU - Massa R

AU - Sarti M

AU - Reddy AB

AU - Prihoda TJ

AU - Vijayalaxmi

AU - Scarfi MR

DO - 10.1016/j.mrgentox.2012.03.013

LA - en

N1 - FEMU ID: 20662; EMF-Portal URL: https://www.emf-portal.org/en/article/20662

SP - 29-35

TI - Induction of an adaptive response in human blood lymphocytes exposed to radiofrequency fields: Influence of the universal mobile telecommunication system (UMTS) signal and the specific absorption rate

ER -

TY - JOUR

IS - 4

JO - Mutagenesis

PY - 2012

SN - 0267-8357

VL - 27

AU - Hintzsche H

AU - Jastrow C

AU - Kleine-Ostmann T

AU - Schrader T

AU - Stopper H

DO - 10.1093/mutage/ges007

LA - en

N1 - FEMU ID: 20370; EMF-Portal URL: https://www.emf-portal.org/en/article/20370

SP - 477-483

TI - 900 MHz radiation does not induce micronucleus formation in different cell types

UR - https://academic.oup.com/mutage/article-pdf/27/4/477/3889278/ges007.pdf

ER -

TY - JOUR

JA - Ecotoxicol Environ Saf

JO - Ecotoxicology and Environmental Safety

PY - 2012

SN - 0147-6513

VL - 80

AU - Sekeroglu V

AU - Akar A

AU - Sekeroglu ZA

DO - 10.1016/j.ecoenv.2012.02.028

LA - en

N1 - FEMU ID: 20362; EMF-Portal URL: https://www.emf-portal.org/en/article/20362

SP - 140-144

TI - Cytotoxic and genotoxic effects of high-frequency electromagnetic fields (GSM 1800MHz) on immature and mature rats

ER -

TY - JOUR

IS - 2

JO - PLoS One

PY - 2012

SN - 1932-6203

VL - 7

AU - Jiang B

AU - Nie J

AU - Zhou Z

AU - Zhang J

AU - Tong J

AU - Cao Y

DO - 10.1371/journal.pone.0032040

LA - en

N1 - FEMU ID: 20338; EMF-Portal URL: https://www.emf-portal.org/en/article/20338

SP - e32040

TI - Adaptive Response in Mice Exposed to 900 MHz Radiofrequency Fields: Primary DNA Damage

UR - http://journals.plos.org/plosone/article?id=10.1371/journal.pone.0032040

ER -

TY - JOUR

IS - 2

JA - Cell Biochem Biophys

JO - Cell Biochemistry and Biophysics

PY - 2012

SN - 1085-9195

VL - 63

AU - Panagopoulos DJ

DO - 10.1007/s12013-012-9347-0

LA - en

N1 - FEMU ID: 20327; EMF-Portal URL: https://www.emf-portal.org/en/article/20327

SP - 121-132

TI - Effect of microwave exposure on the ovarian development of Drosophila melanogaster

ER -

TY - JOUR

IS - 6

JO - Bioelectromagnetics

PY - 2012

SN - 0197-8462

VL - 33

AU - Zeni O

AU - Sannino A

AU - Sarti M

AU - Romeo S

AU - Massa R

AU - Scarfi MR

DO - 10.1002/bem.21712

LA - en

N1 - FEMU ID: 20288; EMF-Portal URL: https://www.emf-portal.org/en/article/20288

SP - 497-507

TI - Radiofrequency radiation at 1950 MHz (UMTS) does not affect key cellular endpoints in neuron-like PC12 cells

ER -

TY - JOUR

IS - 5

JA - Int J Radiat Biol

JO - International Journal of Radiation Biology

PY - 2012

SN - 0955-3002

VL - 88

AU - Cam ST

AU - Seyhan N

DO - 10.3109/09553002.2012.666005

LA - en

N1 - FEMU ID: 20278; EMF-Portal URL: https://www.emf-portal.org/en/article/20278

SP - 420-424

TI - Single-strand DNA breaks in human hair root cells exposed to mobile phone radiation

ER -

TY - JOUR

IS - 7

JA - Hum Exp Toxicol

JO - Human & Experimental Toxicology

PY - 2012

SN - 0960-3271

VL - 31

AU - Khalil AM

AU - Gagaa MH

AU - Alshamali AM

DO - 10.1177/0960327111433184

LA - en

N1 - FEMU ID: 20105; EMF-Portal URL: https://www.emf-portal.org/en/article/20105

SP - 734-740

TI - 8-Oxo-7, 8-dihydro-2'-deoxyguanosine as a biomarker of DNA damage by mobile phone radiation

ER -

TY - JOUR

IS - 4

JA - Int J Radiat Biol

JO - International Journal of Radiation Biology

PY - 2012

SN - 0955-3002

VL - 88

AU - Guler G

AU - Tomruk A

AU - Ozgur E

AU - Sahin D

AU - Sepici A

AU - Altan N

AU - Seyhan N

DO - 10.3109/09553002.2012.646349

LA - en

N1 - FEMU ID: 19950; EMF-Portal URL: https://www.emf-portal.org/en/article/19950

SP - 367-373

TI - The effect of radiofrequency radiation on DNA and lipid damage in female and male infant rabbits

ER -

TY - JOUR

IS - 2

JA - Br J Cancer

JO - British Journal of Cancer

PY - 2012

SN - 0007-0920

VL - 106

AU - Zimmerman JW

AU - Pennison MJ

AU - Brezovich I

AU - Yi N

AU - Yang CT

AU - Ramaker R

AU - Absher D

AU - Myers RM

AU - Kuster N

AU - Costa FP

AU - Barbault A

AU - Pasche B

DO - 10.1038/bjc.2011.523

LA - en

N1 - FEMU ID: 19937; EMF-Portal URL: https://www.emf-portal.org/en/article/19937

SP - 307-313

TI - Cancer cell proliferation is inhibited by specific modulation frequencies

UR - https://stopsmartmetersirvine.files.wordpress.com/2011/12/zimmerman-et-al-advance-online-publication-1201111.pdf

ER -

TY - JOUR

IS - 1

JA - Fertil Steril

JO - Fertility and Sterility

PY - 2012

SN - 0015-0282

VL - 97

AU - Avendano C

AU - Mata A

AU - Sanchez Sarmiento CA

AU - Doncel GF

DO - 10.1016/j.fertnstert.2011.10.012

LA - en

N1 - FEMU ID: 19930; EMF-Portal URL: https://www.emf-portal.org/en/article/19930

SP - 39-45.e2

TI - Use of laptop computers connected to internet through Wi-Fi decreases human sperm motility and increases sperm DNA fragmentation

ER -

TY - JOUR

IS - 1

JA - J Neurooncol

JO - Journal of Neuro-Oncology

PY - 2012

SN - 0167-594X

VL - 106

AU - Karaca E

AU - Durmaz B

AU - Aktug H

AU - Yildiz T

AU - Guducu C

AU - Irgi M

AU - Koksal MG

AU - Ozkinay F

AU - Gunduz C

AU - Cogulu O

DO - 10.1007/s11060-011-0644-z

LA - en

N1 - FEMU ID: 19410; EMF-Portal URL: https://www.emf-portal.org/en/article/19410

SP - 53-58

TI - The genotoxic effect of radiofrequency waves on mouse brain

ER -

TY - JOUR

IS - 4

JA - Coll Antropol

JO - Collegium Antropologicum

PY - 2011

SN - 0350-6134

VL - 35

AU - Trosic I

AU - Pavicic I

AU - Milkovic-Kraus S

AU - Mladinic M

AU - Zeljezic D

LA - en

N1 - FEMU ID: 20359; EMF-Portal URL: https://www.emf-portal.org/en/article/20359

SP - 1259-1264

TI - Effect of electromagnetic radiofrequency radiation on the rats' brain, liver and kidney cells measured by comet assay

UR - https://hrcak.srce.hr/file/112380

ER -

TY - JOUR

JA - World Acad Sci Eng Technol

JO - World Academy of Science, Engineering and Technology

PY - 2011

VL - 76

AU - Khalil AM

AU - Alshamali AM

AU - Gagaa MH

LA - en

N1 - FEMU ID: 20110; EMF-Portal URL: https://www.emf-portal.org/en/article/20110

SP - 657-622

TI - Detection of oxidative stress induced by mobile phone radiation in tissues of mice using 8-oxo-7, 8-dihydro-20-deoxyguanosine as a biomarker

UR - http://publications.waset.org/7054/pdf

ER -

TY - JOUR

IS - 4

JA - Electromagn Biol Med

JO - Electromagnetic Biology and Medicine

PY - 2011

SN - 1536-8386

VL - 30

AU - Zeng L

AU - Ji X

AU - Zhang Y

AU - Miao X

AU - Zou C

AU - Lang H

AU - Zhang J

AU - Li Y

AU - Wang X

AU - Qi H

AU - Ren D

AU - Guo G

DO - 10.3109/15368378.2011.587929

LA - en

N1 - FEMU ID: 19856; EMF-Portal URL: https://www.emf-portal.org/en/article/19856

SP - 205-218

TI - MnSOD expression inhibited by electromagnetic pulse radiation in the rat testis

ER -

TY - JOUR

JA - Sci Total Environ

JO - Science of the Total Evironment

PY - 2011

SN - 0048-9697

VL - 410

AU - Esmekaya MA

AU - Aytekin E

AU - Ozgur E

AU - Güler G

AU - Ergun MA

AU - Omeroglu S

AU - Seyhan N

DO - 10.1016/j.scitotenv.2011.09.036

LA - en

N1 - FEMU ID: 19800; EMF-Portal URL: https://www.emf-portal.org/en/article/19800

SP - 59-64

TI - Mutagenic and morphologic impacts of 1.8 GHz radiofrequency radiation on human peripheral blood lymphocytes (hPBLs) and possible protective role of pre-treatment with Ginkgo biloba (EGb 761)

ER -

TY - JOUR

JA - Progr Electromagn Res B (PIER B)

JO - Progress in Electromagnetics Research B

PY - 2011

SN - 1937-6472

VL - 29

AU - Chaturvedi CM

AU - Singh VP

AU - Singh P

AU - Basu P

AU - Singaravel M

AU - Shukla RK

AU - Dhawan A

AU - Pati AK

AU - Gangwar RK

AU - Singh SP

DO - 10.2528/PIERB11011205

LA - en

N1 - FEMU ID: 19608; EMF-Portal URL: https://www.emf-portal.org/en/article/19608

SP - 23-42

TI - 2.45 GHz (CW) microwave irradiation alters circadian organization, spatial memory, DNA structure in the brain cells and blood cell counts of male mice, Mus musculus

UR - https://www.jpier.org/ac_api/download.php?id=11011205

ER -

TY - JOUR

JO - Mutation Research - Fundamental and Molecular Mechanism of Mutagenesis

PY - 2011

SN - 0027-5107

VL - 716

AU - Ballardin M

AU - Tusa I

AU - Fontana N

AU - Monorchio A

AU - Pelletti C

AU - Rogovich A

AU - Barale R

AU - Scarpato R

DO - 10.1016/j.mrfmmm.2011.07.009

LA - en

N1 - FEMU ID: 19499; EMF-Portal URL: https://www.emf-portal.org/en/article/19499

SP - 1-9

TI - Non-thermal effects of 2.45GHz microwaves on spindle assembly, mitotic cells and viability of Chinese hamster V-79 cells

ER -

TY - JOUR

IS - 9

JA - Int J Radiat Biol

JO - International Journal of Radiation Biology

PY - 2011

SN - 0955-3002

VL - 87

AU - Sannino A

AU - Zeni O

AU - Sarti M

AU - Romeo S

AU - Reddy SB

AU - Belisario MA

AU - Prihoda TJ

AU - Vijayalaxmi

AU - Scarfi MR

DO - 10.3109/09553002.2011.574779

LA - en

N1 - FEMU ID: 19265; EMF-Portal URL: https://www.emf-portal.org/en/article/19265

SP - 993-999

TI - Induction of adaptive response in human blood lymphocytes exposed to 900 MHz radiofrequency fields: Influence of cell cycle

ER -

TY - JOUR

IS - 5

JA - Radiat Res

JO - Radiation Research

PY - 2011

SN - 0033-7587

VL - 175

AU - Hintzsche H

AU - Jastrow C

AU - Kleine-Ostmann T

AU - Stopper H

AU - Schmid E

AU - Schrader T

DO - 10.1667/RR2406.1

LA - en

N1 - FEMU ID: 19081; EMF-Portal URL: https://www.emf-portal.org/en/article/19081

SP - 569-574

TI - Terahertz radiation induces spindle disturbances in human-hamster hybrid cells

ER -

TY - JOUR

IS - 4

JA - Int J Radiat Biol

JO - International Journal of Radiation Biology

PY - 2011

SN - 0955-3002

VL - 87

AU - Bourthoumieu S

AU - Terro F

AU - Leveque P

AU - Collin A

AU - Joubert V

AU - Yardin C

DO - 10.3109/09553002.2011.542543

LA - en

N1 - FEMU ID: 18946; EMF-Portal URL: https://www.emf-portal.org/en/article/18946

SP - 400-408

TI - Aneuploidy studies in human cells exposed in vitro to GSM-900 MHz radiofrequency radiation using FISH

ER -

TY - JOUR

IS - 4

JA - Appl Biochem Biotechnol

JO - Applied Biochemistry and Biotechnology

PY - 2011

SN - 0273-2289

VL - 164

AU - Kesari KK

AU - Kumar S

AU - Behari J

DO - 10.1007/s12010-010-9156-0

LA - en

N1 - FEMU ID: 18931; EMF-Portal URL: https://www.emf-portal.org/en/article/18931

SP - 546-559

TI - Effects of radiofrequency electromagnetic wave exposure from cellular phones on the reproductive pattern in male wistar rats

ER -

TY - JOUR

IS - 4

JO - Bioelectromagnetics

PY - 2011

SN - 0197-8462

VL - 32

AU - Schrader T

AU - Kleine-Ostmann T

AU - Münter K

AU - Jastrow C

AU - Schmid E

DO - 10.1002/bem.20634

LA - en

N1 - FEMU ID: 18854; EMF-Portal URL: https://www.emf-portal.org/en/article/18854

SP - 291-301

TI - Spindle disturbances in human-hamster hybrid (A(L)) cells induced by the electrical component of the mobile communication frequency range signal

ER -

TY - JOUR

IS - 2

JA - Int J Radiat Biol

JO - International Journal of Radiation Biology

PY - 2011

SN - 0955-3002

VL - 87

AU - Kumar G

AU - Wood AW

AU - Anderson V

AU - McIntosh RL

AU - Chen YY

AU - McKenzie RJ

DO - 10.3109/09553002.2010.518212

LA - en

N1 - FEMU ID: 18740; EMF-Portal URL: https://www.emf-portal.org/en/article/18740

SP - 231-240

TI - Evaluation of hematopoietic system effects after in vitro radiofrequency radiation exposure in rats

ER -

TY - JOUR

IS - 1

JA - Int J Hyg Environ Health

JO - International Journal of Hygiene and Environmental Health

PY - 2011

SN - 1438-4639

VL - 214

AU - Garaj-Vrhovac V

AU - Gajski G

AU - Pazanin S

AU - Sarolic A

AU - Domijan AM

AU - Flajs D

AU - Peraica M

DO - 10.1016/j.ijheh.2010.08.003

LA - en

N1 - FEMU ID: 18617; EMF-Portal URL: https://www.emf-portal.org/en/article/18617

SP - 59-65

TI - Assessment of cytogenetic damage and oxidative stress in personnel occupationally exposed to the pulsed microwave radiation of marine radar equipment

ER -

TY - JOUR

IS - 2

JA - Lasers Surg Med

JO - Lasers in Surgery and Medicine

PY - 2011

SN - 0196-8092

VL - 43

AU - Wilmink GJ

AU - Rivest BD

AU - Roth CC

AU - Ibey BL

AU - Payne JA

AU - Cundin LX

AU - Grundt JE

AU - Peralta X

AU - Mixon DG

AU - Roach WP

DO - 10.1002/lsm.20960

LA - en

N1 - FEMU ID: 18578; EMF-Portal URL: https://www.emf-portal.org/en/article/18578

SP - 152-163

TI - In vitro investigation of the biological effects associated with human dermal fibroblasts exposed to 2.52 THz radiation

ER -

TY - JOUR

IS - 9-10

JA - CR physique

JO - Comptes Rendus Physique

PY - 2010

SN - 1631-0705

VL - 11

AU - Perrin A

AU - Freire M

AU - Bachelet C

AU - Collin A

AU - Leveque P

AU - Pla S

AU - Debouzy JC

DO - 10.1016/j.crhy.2010.10.006

LA - en

N1 - FEMU ID: 19405; EMF-Portal URL: https://www.emf-portal.org/en/article/19405

SP - 613-621

TI - Evaluation of the co-genotoxic effects of 1800 MHz GSM radiofrequency exposure and a chemical mutagen in cultured human cells

ER -

TY - JOUR

IS - 10

JA - J Zhejiang Univ Sci B

JO - Journal of Zhejiang University Science B

PY - 2010

SN - 1673-1581

VL - 11

AU - Shckorbatov YG

AU - Pasiuga VN

AU - Goncharuk EI

AU - Petrenko TP

AU - Grabina VA

AU - Kolchigin NN

AU - Ivanchenko DD

AU - Bykov VN

AU - Dumin OM

DO - 10.1631/jzus.B1000051

LA - en

N1 - FEMU ID: 18648; EMF-Portal URL: https://www.emf-portal.org/en/article/18648

SP - 801-805

TI - Effects of differently polarized microwave radiation on the microscopic structure of the nuclei in human fibroblasts

UR - https://www.ncbi.nlm.nih.gov/pmc/articles/PMC2950243/pdf/JZUSB11-0801.pdf

ER -

TY - JOUR

IS - 6

JA - Radiat Res

JO - Radiation Research

PY - 2010

SN - 0033-7587

VL - 174

AU - Bourthoumieu S

AU - Joubert V

AU - Marin B

AU - Collin A

AU - Leveque P

AU - Terro F

AU - Yardin C

LA - en

N1 - FEMU ID: 18635; EMF-Portal URL: https://www.emf-portal.org/en/article/18635

SP - 712-718

TI - Cytogenetic Studies in Human Cells Exposed In Vitro to GSM-900 MHz Radiofrequency Radiation Using R-Banded Karyotyping

ER -

TY - JOUR

IS - 6

JA - Indian J Exp Biol

JO - Indian Journal of Experimental Biology

PY - 2010

SN - 0019-5189

VL - 48

AU - Kumar S

AU - Kesari KK

AU - Behari J

LA - en

N1 - FEMU ID: 18567; EMF-Portal URL: https://www.emf-portal.org/en/article/18567

SP - 586-592

TI - Evaluation of genotoxic effects in male Wistar rats following microwave exposure

UR - http://nopr.niscpr.res.in/bitstream/123456789/9081/1/IJEB%2048%286%29%20586-592.pdf

ER -

TY - JOUR

IS - 3

JA - Electromagn Biol Med

JO - Electromagnetic Biology and Medicine

PY - 2010

SN - 1536-8386

VL - 29

AU - Gurbuz N

AU - Sirav B

AU - Yuvaci HU

AU - Turhan N

AU - Coskun ZK

AU - Seyhan N

DO - 10.3109/15368378.2010.482498

LA - en

N1 - FEMU ID: 18549; EMF-Portal URL: https://www.emf-portal.org/en/article/18549

SP - 98-104

TI - Is There Any Possible Genotoxic Effect in Exfoliated Bladder Cells of Rat Under the Exposure of 1800 MHz GSM-Like Modulated Radio Frequency Radiation (RFR)?

ER -

TY - JOUR

IS - 2

JA - Genet Couns

JO - Genetic Counseling

PY - 2010

SN - 1015-8146

VL - 21

AU - Yildirim MS

AU - Yildirim A

AU - Zamani AG

AU - Okudan N

LA - en

N1 - FEMU ID: 18526; EMF-Portal URL: https://www.emf-portal.org/en/article/18526

SP - 243-251

TI - Effect of mobile phone station on micronucleus frequency and chromosomal aberrations in human blood cells

ER -

TY - JOUR

IS - 6

JO - Bioelectromagnetics

PY - 2010

SN - 0197-8462

VL - 31

AU - Luukkonen J

AU - Juutilainen J

AU - Naarala J

DO - 10.1002/bem.20580

LA - en

N1 - FEMU ID: 18142; EMF-Portal URL: https://www.emf-portal.org/en/article/18142

SP - 417-424

TI - Combined effects of 872 MHz radiofrequency radiation and ferrous chloride on reactive oxygen species production and DNA damage in human SH-SY5Y neuroblastoma cells

ER -

TY - JOUR

IS - 1

JA - Gen Physiol Biophys

JO - General Physiology and Biophysics

PY - 2010

SN - 0231-5882

VL - 29

AU - Güler G

AU - Tomruk A

AU - Ozgur E

AU - Seyhan N

DO - 10.4149/gpb_2010_01_59

LA - en

N1 - FEMU ID: 18104; EMF-Portal URL: https://www.emf-portal.org/en/article/18104

SP - 59-66

TI - The effect of radiofrequency radiation on DNA and lipid damage in non-pregnant and pregnant rabbits and their newborns

ER -

TY - JOUR

IS - 4

JA - Int J Radiat Biol

JO - International Journal of Radiation Biology

PY - 2010

SN - 0955-3002

VL - 86

AU - Kesari KK

AU - Behari J

AU - Kumar S

DO - 10.3109/09553000903564059

LA - en

N1 - FEMU ID: 18089; EMF-Portal URL: https://www.emf-portal.org/en/article/18089

SP - 334-343

TI - Mutagenic response of 2.45 GHz radiation exposure on rat brain

ER -

TY - JOUR

IS - 1

JA - Neurosci Lett

JO - Neuroscience Letters

PY - 2010

SN - 0304-3940

VL - 473

AU - Campisi A

AU - Gulino M

AU - Acquaviva R

AU - Bellia P

AU - Raciti G

AU - Grasso R

AU - Musumeci F

AU - Vanella A

AU - Triglia A

DO - 10.1016/j.neulet.2010.02.018

LA - en

N1 - FEMU ID: 17968; EMF-Portal URL: https://www.emf-portal.org/en/article/17968

SP - 52-55

TI - Reactive oxygen species levels and DNA fragmentation on astrocytes in primary culture after acute exposure to low intensity microwave electromagnetic field

ER -

TY - JOUR

IS - 3

JA - Environ Health Perspect

JO - Environmental Health Perspectives

PY - 2010

SN - 0091-6765

VL - 118

AU - Belyaev I

AU - Markova E

AU - Malmgren L

DO - 10.1289/ehp.0900781

LA - en

N1 - FEMU ID: 17858; EMF-Portal URL: https://www.emf-portal.org/en/article/17858

SP - 394-399

TI - Microwaves from Mobile Phones Inhibit 53BP1 Focus Formation in Human Stem Cells Stronger than in Differentiated Cells: Possible Mechanistic Link to Cancer Risk

UR - https://www.ncbi.nlm.nih.gov/pmc/articles/PMC2854769/pdf/ehp-118-394.pdf

ER -

TY - JOUR

IS - 1

JA - Toxicol Lett

JO - Toxicology Letters

PY - 2010

SN - 0378-4274

VL - 193

AU - Hintzsche H

AU - Stopper H

DO - 10.1016/j.toxlet.2009.12.016

LA - en

N1 - FEMU ID: 17832; EMF-Portal URL: https://www.emf-portal.org/en/article/17832

SP - 124-130

TI - Micronucleus frequency in buccal mucosa cells of mobile phone users

ER -

TY - JOUR

JA - Brain Res

JO - Brain Research

PY - 2010

SN - 0006-8993

VL - 1311

AU - Xu S

AU - Zhou Z

AU - Zhang L

AU - Yu Z

AU - Zhang W

AU - Wang Y

AU - Wang X

AU - Li M

AU - Chen Y

AU - Chen C

AU - He M

AU - Zhang G

AU - Zhong M

DO - 10.1016/j.brainres.2009.10.062

LA - en

N1 - FEMU ID: 17674; EMF-Portal URL: https://www.emf-portal.org/en/article/17674

SP - 189-196

TI - Exposure to 1800 MHz radiofrequency radiation induces oxidative damage to mitochondrial DNA in primary cultured neurons

ER -

TY - JOUR

IS - 1

JA - Cell Biochem Biophys

JO - Cell Biochemistry and Biophysics

PY - 2010

SN - 1085-9195

VL - 56

AU - Tomruk A

AU - Güler G

AU - Dincel AS

DO - 10.1007/s12013-009-9068-1

LA - en

N1 - FEMU ID: 17640; EMF-Portal URL: https://www.emf-portal.org/en/article/17640

SP - 39-47

TI - The influence of 1800 MHz GSM-like signals on hepatic oxidative DNA and lipid damage in nonpregnant, pregnant, and newly born rabbits

ER -

TY - JOUR

IS - 1-2

JA - Mutat Res Genet Toxicol Environ Mutagen

JO - Mutation Research - Genetic Toxicology and Environmental Mutagenesis

PY - 2010

VL - 695

AU - Zhijian C

AU - Xiaoxue L

AU - Yezhen L

AU - Shijie C

AU - Lifen J

AU - Jianlin L

AU - Deqiang L

AU - Jiliang H

DO - 10.1016/j.mrgentox.2009.10.001

LA - en

N1 - FEMU ID: 17623; EMF-Portal URL: https://www.emf-portal.org/en/article/17623

SP - 16-21

TI - Impact of 1.8-GHz radiofrequency radiation (RFR) on DNA damage and repair induced by doxorubicin in human B-cell lymphoblastoid cells

ER -

TY - JOUR

IS - 1-2

JO - Mutation Research - Fundamental and Molecular Mechanism of Mutagenesis

PY - 2010

SN - 0027-5107

VL - 683

AU - Franzellitti S

AU - Valbonesi P

AU - Ciancaglini N

AU - Biondi C

AU - Contin A

AU - Bersani F

AU - Fabbri E

DO - 10.1016/j.mrfmmm.2009.10.004

LA - en

N1 - FEMU ID: 17612; EMF-Portal URL: https://www.emf-portal.org/en/article/17612

SP - 35-42

TI - Transient DNA damage induced by high-frequency electromagnetic fields (GSM 1.8GHz) in the human trophoblast HTR-8/SVneo cell line evaluated with the alkaline comet assay

ER -

TY - JOUR

IS - 11

JA - Anticancer Res

JO - Anticancer Research

PY - 2009

SN - 0250-7005

VL - 29

AU - Hansteen IL

AU - Clausen KO

AU - Haugan V

AU - Svendsen M

AU - Svendsen MV

AU - Eriksen JG

AU - Skiaker R

AU - Hauger E

AU - Lageide L

AU - Vistnes AI

AU - Kure EH

LA - en

N1 - FEMU ID: 17824; EMF-Portal URL: https://www.emf-portal.org/en/article/17824

SP - 4323-4330

TI - Cytogenetic effects of exposure to 2.3 GHz radiofrequency radiation on human lymphocytes in vitro

UR - http://ar.iiarjournals.org/content/29/11/4323.full.pdf+html

ER -

TY - JOUR

IS - 2

JA - Int J Toxicol

JO - International Journal of Toxicology

PY - 2009

SN - 1091-5818

VL - 28

AU - Gajski G

AU - Garaj-Vrhovac V

DO - 10.1177/1091581809335051

LA - en

N1 - FEMU ID: 17452; EMF-Portal URL: https://www.emf-portal.org/en/article/17452

SP - 88-98

TI - Radioprotective effects of honeybee venom (Apis mellifera) against 915-MHz microwave radiation-induced DNA damage in wistar rat lymphocytes: in vitro study

ER -

TY - JOUR

IS - 8

JA - Anticancer Res

JO - Anticancer Research

PY - 2009

SN - 0250-7005

VL - 29

AU - Hansteen IL

AU - Lageide L

AU - Clausen KO

AU - Haugan V

AU - Svendsen M

AU - Eriksen JG

AU - Skiaker R

AU - Hauger E

AU - Vistnes AI

AU - Kure EH

LA - en

N1 - FEMU ID: 17426; EMF-Portal URL: https://www.emf-portal.org/en/article/17426

SP - 2885-2892

TI - Cytogenetic effects of 18.0 and 16.5 GHz microwave radiation on human lymphocytes in vitro

UR - http://ar.iiarjournals.org/content/29/8/2885.full.pdf+html

ER -

TY - JOUR

IS - 7

JO - PLoS One

PY - 2009

SN - 1932-6203

VL - 4

AU - De Iuliis GN

AU - Newey RJ

AU - King BV

AU - Aitken RJ

DO - 10.1371/journal.pone.0006446

LA - en

N1 - FEMU ID: 17394; EMF-Portal URL: https://www.emf-portal.org/en/article/17394

SP - e6446

TI - Mobile phone radiation induces reactive oxygen species production and DNA damage in human spermatozoa in vitro

UR - https://journals.plos.org/plosone/article/file?id=10.1371/journal.pone.0006446&type=printable

ER -

TY - JOUR

IS - 1-2

JA - Mutat Res Genet Toxicol Environ Mutagen

JO - Mutation Research - Genetic Toxicology and Environmental Mutagenesis

PY - 2009

VL - 677

AU - Zhijian C

AU - Xiaoxue L

AU - Yezhen L

AU - Deqiang L

AU - Shijie C

AU - Lifen J

AU - Jianlin L

AU - Jiliang H

DO - 10.1016/j.mrgentox.2009.05.015

LA - en

N1 - FEMU ID: 17178; EMF-Portal URL: https://www.emf-portal.org/en/article/17178

SP - 100-104

TI - Influence of 1.8-GHz (GSM) radiofrequency radiation (RFR) on DNA damage and repair induced by X-rays in human leukocytes in vitro

ER -

TY - JOUR

IS - 6

JA - Radiat Res

JO - Radiation Research

PY - 2009

SN - 0033-7587

VL - 171

AU - Sannino A

AU - Di Costanzo G

AU - Brescia F

AU - Sarti M

AU - Zeni O

AU - Juutilainen J

AU - Scarfi MR

DO - 10.1667/RR1642.1

LA - en

N1 - FEMU ID: 17175; EMF-Portal URL: https://www.emf-portal.org/en/article/17175

SP - 743-751

TI - Human fibroblasts and 900 MHz radiofrequency radiation: evaluation of DNA damage after exposure and co-exposure to 3-chloro-4-(dichloromethyl)-5-hydroxy-2(5h)-furanone (MX)

ER -

TY - JOUR

IS - 6

JA - Radiat Res

JO - Radiation Research

PY - 2009

SN - 0033-7587

VL - 171

AU - Sannino A

AU - Sarti M

AU - Reddy SB

AU - Prihoda TJ

AU - Vijayalaxmi

AU - Scarfi MR

DO - 10.1667/RR1687.1

LA - en

N1 - FEMU ID: 17174; EMF-Portal URL: https://www.emf-portal.org/en/article/17174

SP - 735-742

TI - Induction of adaptive response in human blood lymphocytes exposed to radiofrequency radiation

ER -

TY - JOUR

IS - 4

JA - Int J Radiat Biol

JO - International Journal of Radiation Biology

PY - 2009

SN - 0955-3002

VL - 85

AU - Shckorbatov YG

AU - Pasiuga VN

AU - Kolchigin NN

AU - Grabina VA

AU - Batrakov DO

AU - Kalashnikov VV

AU - Ivanchenko DD

AU - Bykov VN

DO - 10.1080/09553000902781113

LA - en

N1 - FEMU ID: 17050; EMF-Portal URL: https://www.emf-portal.org/en/article/17050

SP - 322-329

TI - The influence of differently polarised microwave radiation on chromatin in human cells

ER -

TY - JOUR

IS - 5

JA - Int J Radiat Biol

JO - International Journal of Radiation Biology

PY - 2009

SN - 0955-3002

VL - 85

AU - Ziemann C

AU - Brockmeyer H

AU - Reddy SB

AU - Vijayalaxmi

AU - Prihoda TJ

AU - Kuster N

AU - Tillmann T

AU - Dasenbrock C

DO - 10.1080/09553000902818907

LA - en

N1 - FEMU ID: 17010; EMF-Portal URL: https://www.emf-portal.org/en/article/17010

SP - 454-464

TI - Absence of genotoxic potential of 902 MHz (GSM) and 1747 MHz (DCS) wireless communication signals: In vivo two-year bioassay in B6C3F1 mice

ER -

TY - JOUR

IS - 1-2

JO - Mutation Research - Fundamental and Molecular Mechanism of Mutagenesis

PY - 2009

SN - 0027-5107

VL - 662

AU - Luukkonen J

AU - Hakulinen P

AU - Maki-Paakkanen J

AU - Juutilainen J

AU - Naarala J

DO - 10.1016/j.mrfmmm.2008.12.005

LA - en

N1 - FEMU ID: 16700; EMF-Portal URL: https://www.emf-portal.org/en/article/16700

SP - 54-58

TI - Enhancement of chemically induced reactive oxygen species production and DNA damage in human SH-SY5Y neuroblastoma cells by 872 MHz radiofrequency radiation

ER -

TY - JOUR

IS - 1

JA - Appl Biochem Biotechnol

JO - Applied Biochemistry and Biotechnology

PY - 2009

SN - 0273-2289

VL - 158

AU - Kesari KK

AU - Behari J

DO - 10.1007/s12010-008-8469-8

LA - en

N1 - FEMU ID: 16653; EMF-Portal URL: https://www.emf-portal.org/en/article/16653

SP - 126-139

TI - Fifty-gigahertz microwave exposure effect of radiations on rat brain

ER -

TY - JOUR

IS - 2

JA - Mutat Res Genet Toxicol Environ Mutagen

JO - Mutation Research - Genetic Toxicology and Environmental Mutagenesis

PY - 2009

VL - 672

AU - Tkalec M

AU - Malaric K

AU - Pavlica M

AU - Pevalek-Kozlina B

AU - Vidakovic-Cifrek Z

DO - 10.1016/j.mrgentox.2008.09.022

LA - en

N1 - FEMU ID: 16578; EMF-Portal URL: https://www.emf-portal.org/en/article/16578

SP - 76-81

TI - Effects of radiofrequency electromagnetic fields on seed germination and root meristematic cells of Allium cepa L

ER -

TY - JOUR

IS - 2

JO - Bioelectromagnetics

PY - 2009

SN - 0197-8462

VL - 30

AU - Belyaev IY

AU - Markova E

AU - Hillert L

AU - Malmgren LO

AU - Persson BR

DO - 10.1002/bem.20445

LA - en

N1 - FEMU ID: 16451; EMF-Portal URL: https://www.emf-portal.org/en/article/16451

SP - 129-141

TI - Microwaves from UMTS/GSM mobile phones induce long-lasting inhibition of 53BP1/gamma-H2AX DNA repair foci in human lymphocytes

ER -

TY - JOUR

IS - 4

JA - Fertil Steril

JO - Fertility and Sterility

PY - 2009

SN - 0015-0282

VL - 92

AU - Agarwal A

AU - Desai NR

AU - Makker K

AU - Varghese A

AU - Mouradi R

AU - Sabanegh E

AU - Sharma R

DO - 10.1016/j.fertnstert.2008.08.022

LA - en

N1 - FEMU ID: 16397; EMF-Portal URL: https://www.emf-portal.org/en/article/16397

SP - 1318-1325

TI - Effects of radiofrequency electromagnetic waves (RF-EMW) from cellular phones on human ejaculated semen: an in vitro pilot study

ER -

TY - JOUR

IS - 4

JA - Electromagn Biol Med

JO - Electromagnetic Biology and Medicine

PY - 2008

SN - 1536-8386

VL - 27

AU - Tiwari R

AU - Lakshmi NK

AU - Surender V

AU - Rajesh AD

AU - Bhargava SC

AU - Ahuja YR

DO - 10.1080/15368370802473554

LA - en

N1 - FEMU ID: 16591; EMF-Portal URL: https://www.emf-portal.org/en/article/16591

SP - 418-425

TI - Combinative Exposure Effect of Radio Frequency Signals from CDMA Mobile Phones and Aphidicolin on DNA Integrity

ER -

TY - JOUR

IS - 11

JA - Int J Radiat Biol

JO - International Journal of Radiation Biology

PY - 2008

SN - 0955-3002

VL - 84

AU - Huang TQ

AU - Lee MS

AU - Oh EH

AU - Kalinec F

AU - Zhang BT

AU - Seo JS

AU - Park WY

DO - 10.1080/09553000802460123

LA - en

N1 - FEMU ID: 16546; EMF-Portal URL: https://www.emf-portal.org/en/article/16546

SP - 909-915

TI - Characterization of biological effect of 1763 MHz radiofrequency exposure on auditory hair cells

ER -

TY - JOUR

IS - 9

JA - Int J Radiat Biol

JO - International Journal of Radiation Biology

PY - 2008

SN - 0955-3002

VL - 84

AU - Huang TQ

AU - Lee MS

AU - Oh E

AU - Zhang BT

AU - Seo JS

AU - Park WY

DO - 10.1080/09553000802317760

LA - en

N1 - FEMU ID: 16424; EMF-Portal URL: https://www.emf-portal.org/en/article/16424

SP - 734-741

TI - Molecular responses of Jurkat T-cells to 1763 MHz radiofrequency radiation

ER -

TY - JOUR

IS - 2

JA - Radiat Res

JO - Radiation Research

PY - 2008

SN - 0033-7587

VL - 170

AU - Korenstein-Ilan A

AU - Barbul A

AU - Hasin P

AU - Eliran A

AU - Gover A

AU - Korenstein R

DO - 10.1667/RR0944.1

LA - en

N1 - FEMU ID: 16198; EMF-Portal URL: https://www.emf-portal.org/en/article/16198

SP - 224-234

TI - Terahertz radiation increases genomic instability in human lymphocytes

ER -

TY - JOUR

IS - 8

JO - Bioelectromagnetics

PY - 2008

SN - 0197-8462

VL - 29

AU - Schrader T

AU - Münter K

AU - Kleine-Ostmann T

AU - Schmid E

DO - 10.1002/bem.20428

LA - en

N1 - FEMU ID: 16004; EMF-Portal URL: https://www.emf-portal.org/en/article/16004

SP - 626-639

TI - Spindle disturbances in human-hamster hybrid (AL) cells induced by mobile communication frequency range signals

ER -

TY - JOUR

JA - Mol Vis

JO - Molecular Vision

PY - 2008

SN - 1090-0535

VL - 14

AU - Yao K

AU - Wu W

AU - Wang K

AU - Ni S

AU - Ye P

AU - Yu Y

AU - Ye J

AU - Sun L

LA - en

N1 - FEMU ID: 15998; EMF-Portal URL: https://www.emf-portal.org/en/article/15998

SP - 964-969

TI - Electromagnetic noise inhibits radiofrequency radiation-induced DNA damage and reactive oxygen species increase in human lens epithelial cells

UR - https://www.ncbi.nlm.nih.gov/pmc/articles/PMC2391079/pdf/mv-v14-964.pdf

ER -

TY - JOUR

IS - 5

JA - Radiat Res

JO - Radiation Research

PY - 2008

SN - 0033-7587

VL - 169

AU - Manti L

AU - Braselmann H

AU - Calabrese ML

AU - Massa R

AU - Pugliese M

AU - Scampoli P

AU - Sicignano G

AU - Grossi G

DO - 10.1667/RR1044.1

LA - en

N1 - FEMU ID: 15911; EMF-Portal URL: https://www.emf-portal.org/en/article/15911

SP - 575-583

TI - Effects of modulated microwave radiation at cellular telephone frequency (1.95 GHz) on X-ray-induced chromosome aberrations in human lymphocytes in vitro

ER -

TY - JOUR

IS - 3

JA - Radiat Res

JO - Radiation Research

PY - 2008

SN - 0033-7587

VL - 169

AU - Valbonesi P

AU - Franzellitti S

AU - Piano A

AU - Contin A

AU - Biondi C

AU - Fabbri E

DO - 10.1667/RR1061.1

LA - en

N1 - FEMU ID: 15699; EMF-Portal URL: https://www.emf-portal.org/en/article/15699

SP - 270-279

TI - Evaluation of HSP70 expression and DNA damage in cells of a human trophoblast cell line exposed to 1.8 GHz amplitude-modulated radiofrequency fields

ER -

TY - JOUR

IS - 6

JA - Int Arch Occup Environ Health

JO - International Archives of Occupational and Environmental Health

PY - 2008

SN - 0340-0131

VL - 81

AU - Schwarz C

AU - Kratochvil E

AU - Pilger A

AU - Kuster N

AU - Adlkofer F

AU - Rudiger HW

DO - 10.1007/s00420-008-0305-5

LA - en

N1 - FEMU ID: 15682; EMF-Portal URL: https://www.emf-portal.org/en/article/15682

SP - 755-767

TI - Radiofrequency electromagnetic fields (UMTS, 1,950 MHz) induce genotoxic effects in vitro in human fibroblasts but not in lymphocytes

ER -

TY - JOUR

IS - 3

JA - Environ Toxicol

JO - Environmental Toxicology

PY - 2008

SN - 1520-4081

VL - 23

AU - Kim JY

AU - Hong SY

AU - Lee YM

AU - Yu SA

AU - Koh WS

AU - Hong JR

AU - Son T

AU - Chang SK

AU - Lee M

DO - 10.1002/tox.20347

LA - en

N1 - FEMU ID: 15600; EMF-Portal URL: https://www.emf-portal.org/en/article/15600

SP - 319-327

TI - In vitro assessment of clastogenicity of mobile-phone radiation (835 MHz) using the alkaline comet assay and chromosomal aberration test

ER -

TY - JOUR

IS - 2

JA - Mutat Res Genet Toxicol Environ Mutagen

JO - Mutation Research - Genetic Toxicology and Environmental Mutagenesis

PY - 2008

VL - 650

AU - Yadav AS

AU - Sharma MK

DO - 10.1016/j.mrgentox.2007.11.005

LA - en

N1 - FEMU ID: 15588; EMF-Portal URL: https://www.emf-portal.org/en/article/15588

SP - 175-180

TI - Increased frequency of micronucleated exfoliated cells among humans exposed in vivo to mobile telephone radiations

ER -

TY - JOUR

IS - 1

JA - Radiat Res

JO - Radiation Research

PY - 2008

SN - 0033-7587

VL - 169

AU - Mazor R

AU - Korenstein-Ilan A

AU - Barbul A

AU - Eshet Y

AU - Shahadi A

AU - Jerby E

AU - Korenstein R

DO - 10.1667/RR0872.1

LA - en

N1 - FEMU ID: 15530; EMF-Portal URL: https://www.emf-portal.org/en/article/15530

SP - 28-37

TI - Increased levels of numerical chromosome aberrations after in vitro exposure of human peripheral blood lymphocytes to radiofrequency electromagnetic fields for 72 hours

ER -

TY - JOUR

IS - 3

JO - Bioelectromagnetics

PY - 2008

SN - 0197-8462

VL - 29

AU - Zeni O

AU - Schiavoni A

AU - Perrotta A

AU - Forigo D

AU - Deplano M

AU - Scarfi MR

DO - 10.1002/bem.20378

LA - en

N1 - FEMU ID: 15373; EMF-Portal URL: https://www.emf-portal.org/en/article/15373

SP - 177-184

TI - Evaluation of genotoxic effects in human leukocytes after in vitro exposure to 1950 MHz UMTS radiofrequency field

ER -

TY - JOUR

IS - 1-2

JA - Acupunct Electrother Res

JO - Acupuncture and Electro-Therapeutics Research

PY - 2007

SN - 0360-1293

VL - 32

AU - Syldona M

DO - 10.3727/036012907815844138

LA - en

N1 - FEMU ID: 15490; EMF-Portal URL: https://www.emf-portal.org/en/article/15490

SP - 1-14

TI - Reducing the in-vitro electromagnetic field effect of cellular phones on human DNA and the intensity of their emitted radiation

ER -

TY - JOUR

IS - 4

JA - Int J Radiat Biol

JO - International Journal of Radiation Biology

PY - 2007

SN - 0955-3002

VL - 83

AU - Juutilainen J

AU - Heikkinen P

AU - Soikkeli H

AU - Maki-Paakkanen J

DO - 10.1080/09553000601169800

LA - en

N1 - FEMU ID: 14790; EMF-Portal URL: https://www.emf-portal.org/en/article/14790

SP - 213-220

TI - Micronucleus frequency in erythrocytes of mice after long-term exposure to radiofrequency radiation

ER -

TY - JOUR

IS - 4

JA - Health Phys

JO - Health Physics

PY - 2007

SN - 0017-9078

VL - 92

AU - Zeni O

AU - Gallerano GP

AU - Perrotta A

AU - Romano M

AU - Sannino A

AU - Sarti M

AU - D'Arienzo M

AU - Doria A

AU - Giovenale E

AU - Lai A

AU - Messina G

AU - Scarfi MR

DO - 10.1097/01.HP.0000251248.23991.35

LA - en

N1 - FEMU ID: 14603; EMF-Portal URL: https://www.emf-portal.org/en/article/14603

SP - 349-357

TI - Cytogenetic Observations In Human Peripheral Blood Leukocytes Following In Vitro Exposure To THz Radiation: A Pilot Study

ER -

TY - JOUR

IS - 3

JO - Toxicology

PY - 2007

SN - 0300-483X

VL - 232

AU - Baohong W

AU - Lifen J

AU - Lanjuan L

AU - Jianlin L

AU - Deqiang L

AU - Wei Z

AU - Jiliang H

DO - 10.1016/j.tox.2007.01.019

LA - en

N1 - FEMU ID: 14600; EMF-Portal URL: https://www.emf-portal.org/en/article/14600

SP - 311-316

TI - Evaluating the combinative effects on human lymphocyte DNA damage induced by ultraviolet ray C plus 1.8 GHz microwaves using comet assay in vitro

ER -

TY - JOUR

IS - 1

JA - J Radiat Res

JO - Journal of Radiation Research

PY - 2007

SN - 0449-3060

VL - 48

AU - Koyama S

AU - Takashima Y

AU - Sakurai T

AU - Suzuki Y

AU - Taki M

AU - Miyakoshi J

DO - 10.1269/jrr.06085

LA - en

N1 - FEMU ID: 14407; EMF-Portal URL: https://www.emf-portal.org/en/article/14407

SP - 69-75

TI - Effects of 2.45 GHz electromagnetic fields with a wide range of SARs on bacterial and HPRT gene mutations

UR - https://www.jstage.jst.go.jp/article/jrr/48/1/48_1_69/_article

ER -

TY - JOUR

IS - 1-2

JA - Mutat Res Genet Toxicol Environ Mutagen

JO - Mutation Research - Genetic Toxicology and Environmental Mutagenesis

PY - 2007

VL - 626

AU - Speit G

AU - Schütz P

AU - Hoffmann H

DO - 10.1016/j.mrgentox.2006.08.003

LA - en

N1 - FEMU ID: 14202; EMF-Portal URL: https://www.emf-portal.org/en/article/14202

SP - 42-47

TI - Genotoxic effects of exposure to radiofrequency electromagnetic fields (RF-EMF) in cultured mammalian cells are not independently reproducible

ER -

TY - JOUR

IS - 1-2

JO - Mutation Research - Fundamental and Molecular Mechanism of Mutagenesis

PY - 2006

SN - 0027-5107

VL - 596

AU - Paulraj R

AU - Behari J

DO - 10.1016/j.mrfmmm.2005.12.006

LA - en

N1 - FEMU ID: 18097; EMF-Portal URL: https://www.emf-portal.org/en/article/18097

SP - 76-80

TI - Single strand DNA breaks in rat brain cells exposed to microwave radiation

ER -

TY - JOUR

IS - 4

JA - IEEE Trans Plasma Sci

JO - IEEE Transactions on Plasma Science

PY - 2006

SN - 0093-3813

VL - 34

AU - Sannino A

AU - Calabrese ML

AU - d'Ambrosio G

AU - Massa R

AU - Petraglia G

AU - Mita P

AU - Sarti M

AU - Scarfi MR

DO - 10.1109/TPS.2006.878379

LA - en

N1 - FEMU ID: 15411; EMF-Portal URL: https://www.emf-portal.org/en/article/15411

SP - 1441-1448

TI - Evaluation of Cytotoxic and Genotoxic Effects in Human Peripheral Blood Leukocytes Following Exposure to 1950-MHz Modulated Signal

ER -

TY - JOUR

IS - 1-2

JO - Mutation Research - Fundamental and Molecular Mechanism of Mutagenesis

PY - 2006

SN - 0027-5107

VL - 602

AU - Lixia S

AU - Yao K

AU - Kaijun W

AU - Deqiang L

AU - Huajun H

AU - Xiangwei G

AU - Baohong W

AU - Wei Z

AU - Jianling L

AU - Wei W

DO - 10.1016/j.mrfmmm.2006.08.010

LA - en

N1 - FEMU ID: 14262; EMF-Portal URL: https://www.emf-portal.org/en/article/14262

SP - 135-142

TI - Effects of 1.8 GHz radiofrequency field on DNA damage and expression of heat shock protein 70 in human lens epithelial cells

ER -

TY - JOUR

IS - 1

JA - Life Sci

JO - Life Sciences

PY - 2006

SN - 0024-3205

VL - 80

AU - Ferreira AR

AU - Knakievicz T

AU - Pasquali MA

AU - Gelain DP

AU - Dal-Pizzol F

AU - Fernandez CE

AU - de Salles AA

AU - Ferreira HB

AU - Moreira JC

DO - 10.1016/j.lfs.2006.08.018

LA - en

N1 - FEMU ID: 14184; EMF-Portal URL: https://www.emf-portal.org/en/article/14184

SP - 43-50

TI - Ultra high frequency-electromagnetic field irradiation during pregnancy leads to an increase in erythrocytes micronuclei incidence in rat offspring

ER -

TY - JOUR

IS - 3

JA - Radiat Res

JO - Radiation Research

PY - 2006

SN - 0033-7587

VL - 166

AU - Vijayalaxmi

DO - 10.1667/RR0643.1

LA - en

N1 - FEMU ID: 14138; EMF-Portal URL: https://www.emf-portal.org/en/article/14138

SP - 532-538

TI - Cytogenetic studies in human blood lymphocytes exposed in vitro to 2.45 GHz or 8.2 GHz radiofrequency radiation

ER -

TY - JOUR

IS - 5

JA - Int J Radiat Biol

JO - International Journal of Radiation Biology

PY - 2006

SN - 0955-3002

VL - 82

AU - Stronati L

AU - Testa A

AU - Moquet J

AU - Edwards A

AU - Cordelli E

AU - Villani P

AU - Marino C

AU - Fresegna AM

AU - Appolloni M

AU - Lloyd D

DO - 10.1080/09553000600739173

LA - en

N1 - FEMU ID: 13927; EMF-Portal URL: https://www.emf-portal.org/en/article/13927

SP - 339-346

TI - 935 MHz cellular phone radiation. An in vitro study of genotoxicity in human lymphocytes

ER -

TY - JOUR

IS - 6

JA - Radiat Res

JO - Radiation Research

PY - 2006

SN - 0033-7587

VL - 165

AU - Scarfi MR

AU - Fresegna AM

AU - Villani P

AU - Pinto R

AU - Marino C

AU - Sarti M

AU - Altavista P

AU - Sannino A

AU - Lovisolo GA

DO - 10.1667/RR3570.1

LA - en

N1 - FEMU ID: 13901; EMF-Portal URL: https://www.emf-portal.org/en/article/13901

SP - 655-663

TI - Exposure to radiofrequency radiation (900 MHz, GSM signal) does not affect micronucleus frequency and cell proliferation in human peripheral blood lymphocytes: an interlaboratory study

ER -

TY - JOUR

IS - 6

JA - Radiat Res

JO - Radiation Research

PY - 2006

SN - 0033-7587

VL - 165

AU - Qutob SS

AU - Chauhan V

AU - Bellier PV

AU - Yauk CL

AU - Douglas GR

AU - Berndt L

AU - Williams A

AU - Gajda GB

AU - Lemay E

AU - Thansandote A

AU - McNamee JP

DO - 10.1667/RR3561.1

LA - en

N1 - FEMU ID: 13899; EMF-Portal URL: https://www.emf-portal.org/en/article/13899

SP - 636-644

TI - Microarray gene expression profiling of a human glioblastoma cell line exposed in vitro to a 1.9 GHz pulse-modulated radiofrequency field

ER -

TY - JOUR

IS - 5

JA - Radiat Res

JO - Radiation Research

PY - 2006

SN - 0033-7587

VL - 165

AU - Verschaeve L

AU - Heikkinen P

AU - Verheyen G

AU - Van Gorp U

AU - Boonen F

AU - Vander Plaetse F

AU - Maes A

AU - Kumlin T

AU - Maki-Paakkanen J

AU - Puranen L

AU - Juutilainen J

DO - 10.1667/RR3559.1

LA - en

N1 - FEMU ID: 13792; EMF-Portal URL: https://www.emf-portal.org/en/article/13792

SP - 598-607

TI - Investigation of co-genotoxic effects of radiofrequency electromagnetic fields in vivo

ER -

TY - JOUR

IS - 4

JO - Bioelectromagnetics

PY - 2006

SN - 0197-8462

VL - 27

AU - Belyaev IY

AU - Koch CB

AU - Terenius O

AU - Röxstrom-Lindquist K

AU - Malmgren LO

AU - Sommer WH

AU - Salford LG

AU - Persson BR

DO - 10.1002/bem.20216

LA - en

N1 - FEMU ID: 13430; EMF-Portal URL: https://www.emf-portal.org/en/article/13430

SP - 295-306

TI - Exposure of rat brain to 915 MHz GSM microwaves induces changes in gene expression but not double stranded DNA breaks or effects on chromatin conformation

ER -

TY - JOUR

IS - 2

JO - Mutagenesis

PY - 2006

SN - 0267-8357

VL - 21

AU - Maes A

AU - Van Gorp U

AU - Verschaeve L

DO - 10.1093/mutage/gel008

LA - en

N1 - FEMU ID: 13387; EMF-Portal URL: https://www.emf-portal.org/en/article/13387

SP - 139-142

TI - Cytogenetic investigation of subjects professionally exposed to radiofrequency radiation

UR - https://academic.oup.com/mutage/article-pdf/21/2/139/3906515/gel008.pdf

ER -

TY - JOUR

IS - 3

JO - Bioelectromagnetics

PY - 2006

SN - 0197-8462

VL - 27

AU - Chemeris NK

AU - Gapeyev AB

AU - Sirota NP

AU - Gudkova OY

AU - Tankanag AV

AU - Konovalov IV

AU - Buzoverya ME

AU - Suvorov VG

AU - Logunov VA

DO - 10.1002/bem.20196

LA - en

N1 - FEMU ID: 12886; EMF-Portal URL: https://www.emf-portal.org/en/article/12886

SP - 197-203

TI - Lack of direct DNA damage in human blood leukocytes and lymphocytes after in vitro exposure to high power microwave pulses

ER -

TY - JOUR

IS - 1

JO - Bioelectromagnetics

PY - 2006

SN - 0197-8462

VL - 27

AU - Sakuma N

AU - Komatsubara Y

AU - Takeda H

AU - Hirose H

AU - Sekijima M

AU - Nojima T

AU - Miyakoshi J

DO - 10.1002/bem.20179

LA - en

N1 - FEMU ID: 12875; EMF-Portal URL: https://www.emf-portal.org/en/article/12875

SP - 51-57

TI - DNA strand breaks are not induced in human cells exposed to 2.1425 GHz band CW and W-CDMA modulated radiofrequency fields allocated to mobile radio base stations

ER -

TY - JOUR

JA - Phys Scr

JO - Physica Scripta

PY - 2005

SN - 0031-8949

VL - 118

AU - Trosic I

AU - Busljeta I

DO - 10.1238/Physica.Topical.118a00168

LA - en

N1 - FEMU ID: 19665; EMF-Portal URL: https://www.emf-portal.org/en/article/19665

SP - 168-170

TI - Frequency of micronucleated erythrocytes in rat bone marrow exposed to 2.45 GHz radiation

ER -

TY - JOUR

IS - 5

JA - Rev Sci Instrum

JO - Review of Scientific Instruments

PY - 2005

SN - 0034-6748

VL - 76

AU - Belloni F

AU - Nassisi V

AU - Alifano P

AU - Monaco C

AU - Tala A

AU - Tredici M

AU - Raino A

LA - en

N1 - FEMU ID: 16862; EMF-Portal URL: https://www.emf-portal.org/en/article/16862

SP - k.A

TI - A suitable plane transmission line at 900 MHz rf fields for E. coli DNA studies

ER -

TY - JOUR

IS - 4

JA - Int J Hum Genet

JO - International Journal of Human Genetics

PY - 2005

SN - 0972-3757

VL - 5

AU - Gandhi G

AU - Singh P

LA - en

N1 - FEMU ID: 16802; EMF-Portal URL: https://www.emf-portal.org/en/article/16802

SP - 259-265

TI - Cytogenetic damage in mobile phone users: preliminary data

UR - http://www.krepublishers.com/02-Journals/IJHG/IJHG-05-0-000-000-2005-Web/IJHG-05-4-225-288-2005-Abst-PDF/IJHG-05-4-259-265-2005-210-Gandhi-G/IJHG-05-4-259-265-2005-210-Gandhi-G.pdf

ER -

TY - JOUR

IS - 2

JA - Indian J Hum Genet

JO - Indian Journal of Human Genetics

PY - 2005

SN - 1998-362X

VL - 11

AU - Gandhi G

AU - Anita

DO - 10.4103/0971-6866.16810

LA - en

N1 - FEMU ID: 16801; EMF-Portal URL: https://www.emf-portal.org/en/article/16801

SP - 99-104

TI - Genetic damage in mobile phone users: some preliminary findings

UR - http://www.bioline.org.br/pdf?hg05022

ER -

TY - JOUR

IS - 1-2

JA - Mutat Res Genet Toxicol Environ Mutagen

JO - Mutation Research - Genetic Toxicology and Environmental Mutagenesis

PY - 2005

VL - 587

AU - Komatsubara Y

AU - Hirose H

AU - Sakurai T

AU - Koyama S

AU - Suzuki Y

AU - Taki M

AU - Miyakoshi J

DO - 10.1016/j.mrgentox.2005.08.010

LA - en

N1 - FEMU ID: 12641; EMF-Portal URL: https://www.emf-portal.org/en/article/12641

SP - 114-119

TI - Effect of high-frequency electromagnetic fields with a wide range of SARs on chromosomal aberrations in murine m5S cells

ER -

TY - JOUR

IS - 4

JA - Radiat Res

JO - Radiation Research

PY - 2005

SN - 0033-7587

VL - 164

AU - Gorlitz BD

AU - Muller M

AU - Ebert S

AU - Hecker H

AU - Kuster N

AU - Dasenbrock C

DO - 10.1667/rr3440.1

LA - en

N1 - FEMU ID: 12605; EMF-Portal URL: https://www.emf-portal.org/en/article/12605

SP - 431-439

TI - Effects of 1-week and 6-week exposure to GSM/DCS radiofrequency radiation on micronucleus formation in B6C3F1 mice

ER -

TY - JOUR

IS - 9

JA - Environ Health Perspect

JO - Environmental Health Perspectives

PY - 2005

SN - 0091-6765

VL - 113

AU - Markova E

AU - Hillert L

AU - Malmgren L

AU - Persson BR

AU - Belyaev IY

DO - 10.1289/ehp.7561

LA - en

N1 - FEMU ID: 12441; EMF-Portal URL: https://www.emf-portal.org/en/article/12441

SP - 1172-1177

TI - Microwaves from GSM mobile telephones affect 53BP1 and gamma-H2AX foci in human lymphocytes from hypersensitive and healthy persons

UR - https://www.ncbi.nlm.nih.gov/pmc/articles/PMC1280397/pdf/ehp0113-001172.pdf

ER -

TY - JOUR

IS - 12

JA - FASEB J

JO - The FASEB Journal

PY - 2005

SN - 0892-6638

VL - 19

AU - Nikolova T

AU - Czyz J

AU - Rolletschek A

AU - Blyszczuk P

AU - Fuchs J

AU - Jovtchev G

AU - Schuderer J

AU - Kuster N

AU - Wobus AM

DO - 10.1096/fj.04-3549fje

LA - en

N1 - FEMU ID: 12365; EMF-Portal URL: https://www.emf-portal.org/en/article/12365

SP - 1686-1688

TI - Electromagnetic fields affect transcript levels of apoptosis-related genes in embryonic stem cell-derived neural progenitor cells

ER -

TY - JOUR

IS - 1-2

JA - Mutat Res Genet Toxicol Environ Mutagen

JO - Mutation Research - Genetic Toxicology and Environmental Mutagenesis

PY - 2005

VL - 582

AU - Zotti-Martelli L

AU - Peccatori M

AU - Maggini V

AU - Ballardin M

AU - Barale R

DO - 10.1016/j.mrgentox.2004.12.014

LA - en

N1 - FEMU ID: 12152; EMF-Portal URL: https://www.emf-portal.org/en/article/12152

SP - 42-52

TI - Individual responsiveness to induction of micronuclei in human lymphocytes after exposure in vitro to 1800 MHz microwave radiation

ER -

TY - JOUR

IS - 1

JA - Electromagn Biol Med

JO - Electromagnetic Biology and Medicine

PY - 2005

SN - 1536-8386

VL - 24

AU - Lai H

AU - Singh NP

LA - en

N1 - FEMU ID: 12077; EMF-Portal URL: https://www.emf-portal.org/en/article/12077

SP - 23-29

TI - Interaction of Microwaves and a Temporally Incoherent Magnetic Field on Single and Double DNA Strand Breaks in Rat Brain Cells

ER -

TY - JOUR

IS - 1-2

JO - Mutation Research - Fundamental and Molecular Mechanism of Mutagenesis

PY - 2005

SN - 0027-5107

VL - 578

AU - Baohong W

AU - Jiliang H

AU - Lifen J

AU - Deqiang L

AU - Wei Z

AU - Jianlin L

AU - Hongping D

DO - 10.1016/j.mrfmmm.2005.05.001

LA - en

N1 - FEMU ID: 12063; EMF-Portal URL: https://www.emf-portal.org/en/article/12063

SP - 149-157

TI - Studying the synergistic damage effects induced by 1.8 GHz radiofrequency field radiation (RFR) with four chemical mutagens on human lymphocyte DNA using comet assay in vitro

ER -

TY - JOUR

IS - 3

JA - Int J Androl

JO - International Journal of Andrology

PY - 2005

SN - 0105-6263

VL - 28

AU - Aitken RJ

AU - Bennetts LE

AU - Sawyer D

AU - Wiklendt AM

AU - King BV

DO - 10.1111/j.1365-2605.2005.00531.x

LA - en

N1 - FEMU ID: 11992; EMF-Portal URL: https://www.emf-portal.org/en/article/11992

SP - 171-179

TI - Impact of radio frequency electromagnetic radiation on DNA integrity in the male germline

ER -

TY - JOUR

IS - 2

JA - Mutat Res Genet Toxicol Environ Mutagen

JO - Mutation Research - Genetic Toxicology and Environmental Mutagenesis

PY - 2005

VL - 583

AU - Diem E

AU - Schwarz C

AU - Adlkofer F

AU - Jahn O

AU - Rüdiger H

DO - 10.1016/j.mrgentox.2005.03.006

LA - en

N1 - FEMU ID: 11910; EMF-Portal URL: https://www.emf-portal.org/en/article/11910

SP - 178-183

TI - Non-thermal DNA breakage by mobile-phone radiation (1800 MHz) in human fibroblasts and in transformed GFSH-R17 rat granulosa cells in vitro

ER -

TY - JOUR

IS - 4

JO - Bioelectromagnetics

PY - 2005

SN - 0197-8462

VL - 26

AU - Zeni O

AU - Romano M

AU - Perrotta A

AU - Lioi MB

AU - Barbieri R

AU - d'Ambrosio G

AU - Massa R

AU - Scarfi MR

DO - 10.1002/bem.20078

LA - en

N1 - FEMU ID: 11836; EMF-Portal URL: https://www.emf-portal.org/en/article/11836

SP - 258-265

TI - Evaluation of genotoxic effects in human peripheral blood leukocytes following an acute in vitro exposure to 900 MHz radiofrequency fields

ER -

TY - JOUR

IS - 2

JA - Eur J Cancer Prev

JO - European Journal of Cancer Prevention

PY - 2005

SN - 0959-8278

VL - 14

AU - Chang SK

AU - Choi JS

AU - Gil HW

AU - Yang JO

AU - Lee EY

AU - Jeon YS

AU - Lee ZW

AU - Lee M

AU - Hong MY

AU - Ho Son T

AU - Hong SY

DO - 10.1097/00008469-200504000-00014

LA - en

N1 - FEMU ID: 11767; EMF-Portal URL: https://www.emf-portal.org/en/article/11767

SP - 175-179

TI - Genotoxicity evaluation of electromagnetic fields generated by 835-MHz mobile phone frequency band

ER -

TY - JOUR

IS - 3

JO - Bioelectromagnetics

PY - 2005

SN - 0197-8462

VL - 26

AU - Belyaev IY

AU - Hillert L

AU - Protopopova M

AU - Tamm C

AU - Malmgren LO

AU - Persson BR

AU - Selivanova G

AU - Harms-Ringdahl M

DO - 10.1002/bem.20103

LA - en

N1 - FEMU ID: 11713; EMF-Portal URL: https://www.emf-portal.org/en/article/11713

SP - 173-184

TI - 915 MHz microwaves and 50 Hz magnetic field affect chromatin conformation and 53BP1 foci in human lymphocytes from hypersensitive and healthy persons

ER -

TY - JOUR

IS - 3

JA - Genet Mol Biol

JO - Genetics and Molecular Biology

PY - 2004

SN - 1415-4757

VL - 27

AU - Figueiredo ABS

AU - Alves RN

AU - Ramalho AT

LA - en

N1 - FEMU ID: 14002; EMF-Portal URL: https://www.emf-portal.org/en/article/14002

SP - 460-466

TI - Cytogenetic analysis of the effects of 2.5 and 10.5 GHz microwaves on human lymphocytes

UR - http://www.scielo.br/pdf/gmb/v27n3/a24v27n3.pdf

ER -

TY - JOUR

IS - 4

JA - IEEE Trans Plasma Sci

JO - IEEE Transactions on Plasma Science

PY - 2004

SN - 0093-3813

VL - 32

AU - Sarimov R

AU - Malmgren LOG

AU - Markova E

AU - Persson BRR

AU - Belyaev IY

DO - 10.1109/TPS.2004.832613

LA - en

N1 - FEMU ID: 13460; EMF-Portal URL: https://www.emf-portal.org/en/article/13460

SP - 1600-1608

TI - Nonthermal GSM Microwaves Affect Chromatin Conformation in Human Lymphocytes Similar to Heat Shock

ER -

TY - JOUR

IS - 6

JA - Int J Hyg Environ Health

JO - International Journal of Hygiene and Environmental Health

PY - 2004

SN - 1438-4639

VL - 207

AU - Busljeta I

AU - Trosic I

AU - Milkovic-Kraus S

DO - 10.1078/1438-4639-00326

LA - en

N1 - FEMU ID: 11630; EMF-Portal URL: https://www.emf-portal.org/en/article/11630

SP - 549-554

TI - Erythropoietic changes in rats after 2.45 GHz nonthermal irradiation

ER -

TY - JOUR

IS - 5

JO - Mutagenesis

PY - 2004

SN - 0267-8357

VL - 19

AU - Trosic I

AU - Busljeta I

AU - Modlic B

DO - 10.1093/mutage/geh042

LA - en

N1 - FEMU ID: 11628; EMF-Portal URL: https://www.emf-portal.org/en/article/11628

SP - 361-364

TI - Investigation of the genotoxic effect of microwave irradiation in rat bone marrow cells: in vivo exposure

UR - https://academic.oup.com/mutage/article-pdf/19/5/361/4067612/geh042.pdf

ER -

TY - JOUR

JA - ScientificWorldJournal

JO - The Scientific World Journal

PY - 2004

SN - 1537-744X

VL - 4

AU - Demsia G

AU - Vlastos D

AU - Matthopoulos DP

DO - 10.1100/tsw.2004.178

LA - en

N1 - FEMU ID: 11395; EMF-Portal URL: https://www.emf-portal.org/en/article/11395

SP - 48-54

TI - Effect of 910-MHz electromagnetic field on rat bone marrow

UR - https://www.hindawi.com/journals/tswj/2004/591712/abs/

ER -

TY - JOUR

JA - ScientificWorldJournal

JO - The Scientific World Journal

PY - 2004

SN - 1537-744X

VL - 4

AU - Koyama S

AU - Isozumi Y

AU - Suzuki Y

AU - Taki M

AU - Miyakoshi J

DO - 10.1100/tsw.2004.176

LA - en

N1 - FEMU ID: 11393; EMF-Portal URL: https://www.emf-portal.org/en/article/11393

SP - 29-40

TI - Effects of 2.45-GHz electromagnetic fields with a wide range of SARs on micronucleus formation in CHO-K1 cells

UR - http://downloads.hindawi.com/journals/tswj/2004/743762.pdf

ER -

TY - JOUR

IS - 1

JA - Int J Radiat Biol

JO - International Journal of Radiation Biology

PY - 2004

SN - 0955-3002

VL - 80

AU - Lagroye I

AU - Anane R

AU - Wettring BA

AU - Moros EG

AU - Straube WL

AU - LaRegina MC

AU - Niehoff M

AU - Pickard WF

AU - Baty J

AU - Roti Roti JL

DO - 10.1080/09553000310001642911

LA - en

N1 - FEMU ID: 11161; EMF-Portal URL: https://www.emf-portal.org/en/article/11161

SP - 11-20

TI - Measurement of DNA damage after acute exposure to pulsed-wave 2450 MHz microwaves in rat brain cells by two alkaline comet assay methods

ER -

TY - JOUR

IS - 1-2

JA - Mutat Res Genet Toxicol Environ Mutagen

JO - Mutation Research - Genetic Toxicology and Environmental Mutagenesis

PY - 2004

VL - 558

AU - Chemeris NK

AU - Gapeyev AB

AU - Sirota NP

AU - Gudkova OY

AU - Kornienko NV

AU - Tankanag AV

AU - Konovalov IV

AU - Buzoverya ME

AU - Suvorov VG

AU - Logunov VA

DO - 10.1016/j.mrgentox.2003.10.017

LA - en

N1 - FEMU ID: 10650; EMF-Portal URL: https://www.emf-portal.org/en/article/10650

SP - 27-34

TI - DNA damage in frog erythrocytes after in vitro exposure to a high peak-power pulsed electromagnetic field

ER -

TY - JOUR

IS - 2

JA - Radiat Res

JO - Radiation Research

PY - 2004

SN - 0033-7587

VL - 161

AU - Hook GJ

AU - Zhang P

AU - Lagroye I

AU - Li L

AU - Higashikubo R

AU - Moros EG

AU - Straube WL

AU - Pickard WF

AU - Baty JD

AU - Roti Roti JL

DO - 10.1667/rr3127

LA - en

N1 - FEMU ID: 10635; EMF-Portal URL: https://www.emf-portal.org/en/article/10635

SP - 193-200

TI - Measurement of DNA damage and apoptosis in Molt-4 cells after in vitro exposure to radiofrequency radiation

ER -

TY - JOUR

IS - 2

JA - Radiat Res

JO - Radiation Research

PY - 2004

SN - 0033-7587

VL - 161

AU - Lagroye I

AU - Hook GJ

AU - Wettring BA

AU - Baty JD

AU - Moros EG

AU - Straube WL

AU - Roti Roti JL

DO - 10.1667/rr3122

LA - en

N1 - FEMU ID: 10634; EMF-Portal URL: https://www.emf-portal.org/en/article/10634

SP - 201-214

TI - Measurements of alkali-labile DNA damage and protein-DNA crosslinks after 2450 MHz microwave and low-dose gamma irradiation in vitro

ER -

TY - JOUR

IS - 3

JA - Radiat Res

JO - Radiation Research

PY - 2004

SN - 0033-7587

VL - 161

AU - Vijayalaxmi

AU - Logani MK

AU - Bhanushali A

AU - Ziskin MC

AU - Prihoda TJ

DO - 10.1667/rr3121

LA - en

N1 - FEMU ID: 10560; EMF-Portal URL: https://www.emf-portal.org/en/article/10560

SP - 341-345

TI - Micronuclei in peripheral blood and bone marrow cells of mice exposed to 42 GHz electromagnetic millimeter waves

ER -

TY - JOUR

IS - 2

JA - J Cell Physiol

JO - Journal of Cellular Physiology

PY - 2004

SN - 0021-9541

VL - 198

AU - Marinelli F

AU - La Sala D

AU - Cicciotti G

AU - Cattini L

AU - Trimarchi C

AU - Putti S

AU - Zamparelli A

AU - Giuliani L

AU - Tomassetti G

AU - Cinti C

DO - 10.1002/jcp.10425

LA - en

N1 - FEMU ID: 10381; EMF-Portal URL: https://www.emf-portal.org/en/article/10381

SP - 324-332

TI - Exposure to 900 MHz electromagnetic field induces an unbalance between pro-apoptotic and pro-survival signals in T-lymphoblastoid leukemia CCRF-CEM cells

ER -

TY - JOUR

IS - 2-3

JA - J Biol Phys

JO - Journal of Biological Physics

PY - 2003

SN - 0092-0606

VL - 29

AU - Scarfi MR

AU - Romano M

AU - Di Pietro R

AU - Zeni O

AU - Doria A

AU - Gallerano GP

AU - Giovenale E

AU - Messina G

AU - Lai A

AU - Campurra G

AU - Coniglio D

AU - Arienzo D

DO - 10.1023/A:1024440708943

LA - en

N1 - FEMU ID: 14927; EMF-Portal URL: https://www.emf-portal.org/en/article/14927

SP - 171-177

TI - THz Exposure of Whole Blood for the Study of Biological Effects on Human Lymphocytes

UR - https://www.ncbi.nlm.nih.gov/pmc/articles/PMC3456423/pdf/10867_2004_Article_5121580.pdf

ER -

TY - JOUR

IS - 9

JA - Int J Radiat Biol

JO - International Journal of Radiation Biology

PY - 2003

SN - 0955-3002

VL - 79

AU - Port M

AU - Abend M

AU - Romer B

AU - Van Beuningen D

DO - 10.1080/09553000310001606803

LA - en

N1 - FEMU ID: 10792; EMF-Portal URL: https://www.emf-portal.org/en/article/10792

SP - 701-708

TI - Influence of high-frequency electromagnetic fields on different modes of cell death and gene expression

ER -

TY - JOUR

IS - 2-3

JA - Electromagn Biol Med

JO - Electromagnetic Biology and Medicine

PY - 2003

SN - 1536-8386

VL - 22

AU - Gadhia PK

AU - Shah T

AU - Mistry A

AU - Pithawala M

AU - Tamakuvala D

DO - 10.1081/JBC-120024624

LA - en

N1 - FEMU ID: 10674; EMF-Portal URL: https://www.emf-portal.org/en/article/10674

SP - 149-159

TI - A Preliminary Study to Assess Possible Chromosomal Damage Among Users of Digital Mobile Phones

ER -

TY - JOUR

IS - 1-2

JA - Mutat Res Genet Toxicol Environ Mutagen

JO - Mutation Research - Genetic Toxicology and Environmental Mutagenesis

PY - 2003

VL - 542

AU - Stacey M

AU - Stickley J

AU - Fox P

AU - Statler V

AU - Schoenbach K

AU - Beebe SJ

AU - Buescher S

DO - 10.1016/j.mrgentox.2003.08.006

LA - en

N1 - FEMU ID: 10503; EMF-Portal URL: https://www.emf-portal.org/en/article/10503

SP - 65-75

TI - Differential effects in cells exposed to ultra-short, high intensity electric fields: cell survival, DNA damage, and cell cycle analysis

ER -

TY - JOUR

IS - 1-2

JA - Mutat Res Genet Toxicol Environ Mutagen

JO - Mutation Research - Genetic Toxicology and Environmental Mutagenesis

PY - 2003

VL - 541

AU - Koyama S

AU - Nakahara T

AU - Wake K

AU - Taki M

AU - Isozumi Y

AU - Miyakoshi J

DO - 10.1016/j.mrgentox.2003.07.009

LA - en

N1 - FEMU ID: 10341; EMF-Portal URL: https://www.emf-portal.org/en/article/10341

SP - 81-89

TI - Effects of high frequency electromagnetic fields on micronucleus formation in CHO-K1 cells

ER -

TY - JOUR

IS - 2

JA - Radiat Res

JO - Radiation Research

PY - 2003

SN - 0033-7587

VL - 160

AU - Zeni O

AU - Chiavoni AS

AU - Sannino A

AU - Antolini A

AU - Forigo D

AU - Bersani F

AU - Scarfi MR

DO - 10.1667/rr3014

LA - en

N1 - FEMU ID: 10036; EMF-Portal URL: https://www.emf-portal.org/en/article/10036

SP - 152-158

TI - Lack of genotoxic effects (micronucleus induction) in human lymphocytes exposed in vitro to 900 MHz electromagnetic fields

ER -

TY - JOUR

IS - 4

JA - Radiat Res

JO - Radiation Research

PY - 2003

SN - 0033-7587

VL - 159

AU - Vijayalaxmi

AU - Sasser LB

AU - Morris JE

AU - Wilson BW

AU - Anderson LE

DO - 10.1667/0033-7587(2003)159[0558:gpogwc]2.0.co;2

LA - en

N1 - FEMU ID: 9893; EMF-Portal URL: https://www.emf-portal.org/en/article/9893

SP - 558-564

TI - Genotoxic potential of 1.6 GHz wireless communication signal: in vivo two-year bioassay

UR - https://meridian.allenpress.com/radiation-research/article-pdf/159/4/558/2194600/0033-7587(2003)159%5b0558_gpogwc%5d2_0_co_2.pdf

ER -

TY - JOUR

IS - 5

JA - Radiat Res

JO - Radiation Research

PY - 2003

SN - 0033-7587

VL - 159

AU - McNamee JP

AU - Bellier PV

AU - Gajda GB

AU - Lavallee BF

AU - Marro L

AU - Lemay E

AU - Thansandote A

DO - 10.1667/0033-7587(2003)159[0693:nefgef]2.0.co;2

LA - en

N1 - FEMU ID: 9883; EMF-Portal URL: https://www.emf-portal.org/en/article/9883

SP - 693-697

TI - No evidence for genotoxic effects from 24 h exposure of human leukocytes to 1.9 GHz radiofrequency fields

ER -

TY - JOUR

IS - 2

JO - Bioelectromagnetics

PY - 2003

SN - 0197-8462

VL - 24

AU - Mashevich M

AU - Folkman D

AU - Kesar A

AU - Barbul A

AU - Korenstein R

AU - Jerby E

AU - Avivi L

DO - 10.1002/bem.10086

LA - en

N1 - FEMU ID: 9413; EMF-Portal URL: https://www.emf-portal.org/en/article/9413

SP - 82-90

TI - Exposure of human peripheral blood lymphocytes to electromagnetic fields associated with cellular phones leads to chromosomal instability

ER -

TY - JOUR

IS - 4

JA - IEEE Trans Plasma Sci

JO - IEEE Transactions on Plasma Science

PY - 2002

SN - 0093-3813

VL - 30

AU - Yu G

AU - Coln EA

AU - Schoenbach KH

AU - Gellermann M

AU - Fox P

AU - Rec L

AU - Beebe SJ

AU - Liu S

DO - 10.1109/TPS.2002.804179

LA - en

N1 - FEMU ID: 18244; EMF-Portal URL: https://www.emf-portal.org/en/article/18244

SP - 1489-1496

TI - A study on biological effects of low-intensity millimeter waves

ER -

TY - JOUR

IS - 1-2

JA - Mutat Res Genet Toxicol Environ Mutagen

JO - Mutation Research - Genetic Toxicology and Environmental Mutagenesis

PY - 2002

VL - 521

AU - Trosic I

AU - Busljeta I

AU - Kasuba V

AU - Rozgaj R

DO - 10.1016/s1383-5718(02)00214-0

LA - en

N1 - FEMU ID: 11629; EMF-Portal URL: https://www.emf-portal.org/en/article/11629

SP - 73-79

TI - Micronucleus induction after whole-body microwave irradiation of rats

ER -

TY - JOUR

IS - 1

JO - Biochimica et Biophysica Acta - General Subjects

PY - 2002

VL - 1572

AU - Shcheglov VS

AU - Alipov ED

AU - Belyaev IY

DO - 10.1016/s0304-4165(02)00283-0

LA - en

N1 - FEMU ID: 10302; EMF-Portal URL: https://www.emf-portal.org/en/article/10302

SP - 101-106

TI - Cell-to-cell communication in response of E. coli cells at different phases of growth to low-intensity microwaves

ER -

TY - JOUR

IS - 4

JA - Biomed Environ Sci

JO - Biomedical and Environmental Sciences

PY - 2002

SN - 0895-3988

VL - 15

AU - Zhang MB

AU - He JL

AU - Jin LF

AU - Lu DQ

LA - en

N1 - FEMU ID: 9988; EMF-Portal URL: https://www.emf-portal.org/en/article/9988

SP - 283-290

TI - Study of low-intensity 2450-MHz microwave exposure enhancing the genotoxic effects of mitomycin C using micronucleus test and comet assay in vitro

ER -

TY - JOUR

IS - 4

JA - Radiat Res

JO - Radiation Research

PY - 2002

SN - 0033-7587

VL - 158

AU - McNamee JP

AU - Bellier PV

AU - Gajda GB

AU - Miller SM

AU - Lemay EP

AU - Lavallee BF

AU - Marro L

AU - Thansandote A

DO - 10.1667/0033-7587(2002)158[0523:ddamii]2.0.co;2

LA - en

N1 - FEMU ID: 9741; EMF-Portal URL: https://www.emf-portal.org/en/article/9741

SP - 523-533

TI - DNA damage and micronucleus induction in human leukocytes after acute in vitro exposure to a 1.9 GHz continuous-wave radiofrequency field

ER -

TY - JOUR

IS - 4

JA - Radiat Res

JO - Radiation Research

PY - 2002

SN - 0033-7587

VL - 158

AU - McNamee JP

AU - Bellier PV

AU - Gajda GB

AU - Lavallee BF

AU - Lemay EP

AU - Marro L

AU - Thansandote A

DO - 10.1667/0033-7587(2002)158[0534:ddihla]2.0.co;2

LA - en

N1 - FEMU ID: 9740; EMF-Portal URL: https://www.emf-portal.org/en/article/9740

SP - 534-537

TI - DNA damage in human leukocytes after acute in vitro exposure to a 1.9 GHz pulse-modulated radiofrequency field

ER -

TY - JOUR

IS - 5

JA - Radiat Res

JO - Radiation Research

PY - 2002

SN - 0033-7587

VL - 157

AU - Bisht KS

AU - Moros EG

AU - Straube WL

AU - Baty JD

AU - Roti Roti JL

DO - 10.1667/0033-7587(2002)157[0506:teomfo]2.0.co;2

LA - en

N1 - FEMU ID: 8831; EMF-Portal URL: https://www.emf-portal.org/en/article/8831

SP - 506-515

TI - The effect of 835.62 MHz FDMA or 847.74 MHz CDMA modulated radiofrequency radiation on the induction of micronuclei in C3H 10T(1/2) cells

ER -

TY - JOUR

IS - 7

JA - Cancer Res

JO - Cancer Research

PY - 2002

SN - 0008-5472

VL - 62

AU - Takahashi S

AU - Inaguma S

AU - Cho YM

AU - Imaida K

AU - Wang J

AU - Fujiwara O

AU - Shirai T

LA - en

N1 - FEMU ID: 8665; EMF-Portal URL: https://www.emf-portal.org/en/article/8665

SP - 1956-1960

TI - Lack of mutation induction with exposure to 1.5 GHz electromagnetic near fields used for cellular phones in brains of Big Blue mice

UR - https://cancerres.aacrjournals.org/content/62/7/1956.full.pdf+html

ER -

TY - JOUR

IS - 2

JO - Bioelectromagnetics

PY - 2002

SN - 0197-8462

VL - 23

AU - Tice RR

AU - Hook GG

AU - Donner M

AU - McRee DI

AU - Guy AW

DO - 10.1002/bem.104

LA - en

N1 - FEMU ID: 8518; EMF-Portal URL: https://www.emf-portal.org/en/article/8518

SP - 113-126

TI - Genotoxicity of radiofrequency signals. I. Investigation of DNA damage and micronuclei induction in cultured human blood cells

ER -

TY - JOUR

IS - 1

JO - Bioelectromagnetics

PY - 2002

SN - 0197-8462

VL - 23

AU - d'Ambrosio G

AU - Massa R

AU - Scarfi MR

AU - Zeni O

DO - 10.1002/bem.93

LA - en

N1 - FEMU ID: 8130; EMF-Portal URL: https://www.emf-portal.org/en/article/8130

SP - 7-13

TI - Cytogenetic damage in human lymphocytes following GMSK phase modulated microwave exposure

ER -

TY - GEN

ET - 1

PB - Springer

PP - Boston

PY - 2001

SN - 9780306469015

T2 - Wireless Phones and Health II: State of the Science

AU - Hook GJ

DO - 10.1007/0-306-46901-4_11

LA - en

N1 - FEMU ID: 49553; EMF-Portal URL: https://www.emf-portal.org/en/article/49553

SP - 143-146

TI - Evaluation of potential genotoxicity of radiofrequency radiation technologies using the single cell gel electrophoresis and micronucleus assays

ER -

TY - JOUR

IS - 2

JA - Acta Med Okayama

JO - Acta Medica Okayama

PY - 2001

SN - 0386-300X

VL - 55

AU - Lalic H

AU - Lekic A

AU - Radosevic-Stasic B

DO - 10.18926/AMO/32005

LA - en

N1 - FEMU ID: 8555; EMF-Portal URL: https://www.emf-portal.org/en/article/8555

SP - 117-127

TI - Comparison of chromosome aberrations in peripheral blood lymphocytes from people occupationally exposed to ionizing and radiofrequency radiation

UR - http://www.lib.okayama-u.ac.jp/www/acta/pdf/55_2_117.pdf

ER -

TY - JOUR

IS - 3

JA - Radiat Res

JO - Radiation Research

PY - 2001

SN - 0033-7587

VL - 156

AU - Li L

AU - Bisht KS

AU - Lagroye I

AU - Zhang P

AU - Straube WL

AU - Moros EG

AU - Roti Roti JL

DO - 10.1667/0033-7587(2001)156[0328:moddim]2.0.co;2

LA - en

N1 - FEMU ID: 8548; EMF-Portal URL: https://www.emf-portal.org/en/article/8548

SP - 328-332

TI - Measurement of DNA damage in mammalian cells exposed in vitro to radiofrequency fields at SARs of 3-5 W/kg

ER -

TY - JOUR

IS - 6

JA - J Magn Reson Imaging

JO - Journal of Magnetic Resonance Imaging

PY - 2001

SN - 1053-1807

VL - 14

AU - Schreiber WG

AU - Teichmann EM

AU - Schiffer I

AU - Hast J

AU - Akbari W

AU - Georgi H

AU - Graf R

AU - Hehn M

AU - Spiebeta HW

AU - Thelen M

AU - Oesch F

AU - Hengstler JG

DO - 10.1002/jmri.10010

LA - en

N1 - FEMU ID: 7986; EMF-Portal URL: https://www.emf-portal.org/en/article/7986

SP - 779-788

TI - Lack of mutagenic and co-mutagenic effects of magnetic fields during magnetic resonance imaging

ER -

TY - JOUR

IS - 4

JA - Radiat Res

JO - Radiation Research

PY - 2001

SN - 0033-7587

VL - 156

AU - Vijayalaxmi

AU - Bisht KS

AU - Pickard WF

AU - Meltz ML

AU - Roti Roti JL

AU - Moros EG

DO - 10.1667/0033-7587(2001)156[0430:cdamfi]2.0.co;2

LA - en

N1 - FEMU ID: 7827; EMF-Portal URL: https://www.emf-portal.org/en/article/7827

SP - 430-433

TI - Chromosome damage and micronucleus formation in human blood lymphocytes exposed in vitro to radiofrequency radiation at a cellular telephone frequency (847.74 MHz, CDMA)

ER -

TY - JOUR

IS - 11

JA - Int J Radiat Biol

JO - International Journal of Radiation Biology

PY - 2001

SN - 0955-3002

VL - 77

AU - Vijayalaxmi

AU - Pickard WF

AU - Bisht KS

AU - Prihoda TJ

AU - Meltz ML

AU - LaRegina MC

AU - Roti Roti JL

AU - Straube WL

AU - Moros EG

DO - 10.1080/09553000110069100

LA - en

N1 - FEMU ID: 7826; EMF-Portal URL: https://www.emf-portal.org/en/article/7826

SP - 1109-1115

TI - Micronuclei in the peripheral blood and bone marrow cells of rats exposed to 2450 MHz radiofrequency radiation

ER -

TY - JOUR

IS - 5

JA - Radiat Res

JO - Radiation Research

PY - 2001

SN - 0033-7587

VL - 156

AU - Sykes PJ

AU - McCallum BD

AU - Bangay MJ

AU - Hooker AM

AU - Morley AA

DO - 10.1667/0033-7587(2001)156[0495:eoetmr]2.0.co;2

LA - en

N1 - FEMU ID: 7646; EMF-Portal URL: https://www.emf-portal.org/en/article/7646

SP - 495-502

TI - Effect of exposure to 900 MHz radiofrequency radiation on intrachromosomal recombination in pKZ1 mice

ER -

TY - JOUR

IS - 2

JO - Bioelectromagnetics

PY - 2001

SN - 0197-8462

VL - 22

AU - Maes A

AU - Collier M

AU - Verschaeve L

LA - en

N1 - FEMU ID: 5625; EMF-Portal URL: https://www.emf-portal.org/en/article/5625

SP - 91-96

TI - Cytogenetic effects of 900 MHz (GSM) microwaves on human lymphocytes

ER -

TY - JOUR

IS - 1

JA - Radiat Res

JO - Radiation Research

PY - 2001

SN - 0033-7587

VL - 155

AU - Vijayalaxmi

AU - Leal BZ

AU - Meltz ML

AU - Pickard WF

AU - Bisht KS

AU - Roti Roti JL

AU - Straube WL

AU - Moros EG

DO - 10.1667/0033-7587(2001)155[0113:csihbl]2.0.co;2

LA - en

N1 - FEMU ID: 5187; EMF-Portal URL: https://www.emf-portal.org/en/article/5187

SP - 113-121

TI - Cytogenetic studies in human blood lymphocytes exposed in vitro to radiofrequency radiation at a cellular telephone frequency (835.62 MHz, FDMA)

ER -

TY - JOUR

IS - 1

JA - Radiat Res

JO - Radiation Research

PY - 2001

SN - 0033-7587

VL - 155

AU - Roti Roti JL

AU - Malyapa RS

AU - Bisht KS

AU - Ahern EW

AU - Moros EG

AU - Pickard WF

AU - Straube WL

DO - 10.1667/0033-7587(2001)155[0239:nticca]2.0.co;2

LA - en

N1 - FEMU ID: 5186; EMF-Portal URL: https://www.emf-portal.org/en/article/5186

SP - 239-247

TI - Neoplastic transformation in C3H 10T(1/2) cells after exposure to 835.62 MHz FDMA and 847.74 MHz CDMA radiations

ER -

TY - JOUR

IS - 11

JA - Rofo

JO - RöFo: Fortschritte auf dem Gebiet der Röntgenstrahlen und der bildgebenden Verfahren

PY - 2000

SN - 1438-9010

VL - 172

AU - Teichmann EM

AU - Hengstler JG

AU - Schreiber WG

AU - Akbari W

AU - Georgi H

AU - Hehn M

AU - Schiffer I

AU - Oesch F

AU - Spiess HW

AU - Thelen M

DO - 10.1055/s-2000-8378

LA - de

N1 - FEMU ID: 5715; EMF-Portal URL: https://www.emf-portal.org/en/article/5715

SP - 934-939

TI - Untersuchung eines möglich mutagenen Potenzials von Magnetfeldern

ER -

TY - JOUR

IS - 1-2

JA - Mutat Res Genet Toxicol Environ Mutagen

JO - Mutation Research - Genetic Toxicology and Environmental Mutagenesis

PY - 2000

VL - 471

AU - Zotti-Martelli L

AU - Peccatori M

AU - Scarpato R

AU - Migliore L

DO - 10.1016/s1383-5718(00)00112-1

LA - en

N1 - FEMU ID: 5088; EMF-Portal URL: https://www.emf-portal.org/en/article/5088

SP - 51-58

TI - Induction of micronuclei in human lymphocytes exposed in vitro to microwave radiation

ER -

TY - JOUR

IS - 5

JA - Folia Biol

JO - Folia Biologica

PY - 2000

SN - 0015-5500

VL - 46

AU - Maes A

AU - Collier M

AU - Verschaeve L

LA - en

N1 - FEMU ID: 4988; EMF-Portal URL: https://www.emf-portal.org/en/article/4988

SP - 175-180

TI - Cytogenetic investigations on microwaves emitted by a 455.7 MHz car phone

ER -

TY - JOUR

IS - 4

JA - Radiat Res

JO - Radiation Research

PY - 2000

SN - 0033-7587

VL - 153

AU - Vijayalaxmi

AU - Leal BZ

AU - Szilagyi M

AU - Prihoda TJ

AU - Meltz ML

DO - 10.1667/0033-7587(2000)153[0479:pddihb]2.0.co;2

LA - en

N1 - FEMU ID: 4299; EMF-Portal URL: https://www.emf-portal.org/en/article/4299

SP - 479-486

TI - Primary DNA damage in human blood lymphocytes exposed in vitro to 2450 MHz radiofrequency radiation

ER -

TY - JOUR

IS - 7

JO - Bioelectromagnetics

PY - 2000

SN - 0197-8462

VL - 21

AU - Gos P

AU - Eicher B

AU - Kohli J

AU - Heyer WD

DO - 10.1002/1521-186x(200010)21:7<515::aid-bem5>3.0.co;2-k

LA - en

N1 - FEMU ID: 3935; EMF-Portal URL: https://www.emf-portal.org/en/article/3935

SP - 515-523

TI - No mutagenic or recombinogenic effects of mobile phone fields at 900 MHz detected in the yeast saccharomyces cerevisiae

ER -

TY - JOUR

IS - 13

JO - Chemosphere

PY - 1999

SN - 0045-6535

VL - 39

AU - Garaj-Vrhovac V

DO - 10.1016/s0045-6535(99)00139-3

LA - en

N1 - FEMU ID: 5642; EMF-Portal URL: https://www.emf-portal.org/en/article/5642

SP - 2301-2312

TI - Micronucleus assay and lymphocyte mitotic activity in risk assessment of occupational exposure to microwave radiation

ER -

TY - JOUR

IS - 1

JA - Int J Radiat Biol

JO - International Journal of Radiation Biology

PY - 1999

SN - 0955-3002

VL - 75

AU - Vijayalaxmi

AU - Seaman RL

AU - Belt ML

AU - Doyle JM

AU - Mathur SP

AU - Prihoda TJ

DO - 10.1080/095530099140870

LA - en

N1 - FEMU ID: 2207; EMF-Portal URL: https://www.emf-portal.org/en/article/2207

SP - 115-120

TI - Frequency of micronuclei in the blood and bone marrow cells of mice exposed to ultra-wideband electromagnetic radiation

ER -

TY - JOUR

IS - 2

JA - J Microw Power Electromagn Energy

JO - Journal of Microwave Power and Electromagnetic Energy

PY - 1998

SN - 0832-7823

VL - 33

AU - Kuchma T

DO - 10.1080/08327823.1998.11688363

LA - en

N1 - FEMU ID: 11053; EMF-Portal URL: https://www.emf-portal.org/en/article/11053

SP - 77-87

TI - Synergistic effect of microwave heating and hydrogen peroxide on inactivation of microorganisms

ER -

TY - JOUR

JO - Edition Wissenschaft

PY - 1998

VL - 14

AU - Antonopoulos A

AU - Obe G

AU - Brinkmann K

AU - Eisenbrandt H

AU - Grigat JP

AU - Elsner R

AU - Storbeck W

AU - Dehmel G

LA - de

N1 - FEMU ID: 9446; EMF-Portal URL: https://www.emf-portal.org/en/article/9446

SP - 3-13

TI - Der Einfluß von hochfrequenten elektromagnetischen Feldern auf den Zellzyklus und auf die Frequenz von Schwesterchromatidaustauschen: Analysen an menschlichen Lymphozyten in Kultur

UR - https://d-nb.info/974863599/34

ER -

TY - JOUR

IS - 1

JA - Bioelectrochem Bioenerg

JO - Bioelectrochemistry and Bioenergetics

PY - 1998

SN - 0302-4598

VL - 45

AU - Phillips JL

AU - Ivaschuk O

AU - Ishida-Jones T

AU - Jones RA

AU - Campbell-Beachler M

AU - Haggren W

LA - en

N1 - FEMU ID: 2112; EMF-Portal URL: https://www.emf-portal.org/en/article/2112

SP - 103-110

TI - DNA damage in Molt-4 T-lymphoblastoid cells exposed to cellular telephone radiofrequency fields in vitro

ER -

TY - JOUR

IS - 2

JO - Bioelectromagnetics

PY - 1998

SN - 0197-8462

VL - 19

AU - Pakhomova ON

AU - Belt ML

AU - Mathur SP

AU - Lee JC

AU - Akyel Y

DO - 10.1002/(sici)1521-186x(1998)19:2<128::aid-bem12>3.0.co;2-m

LA - en

N1 - FEMU ID: 2082; EMF-Portal URL: https://www.emf-portal.org/en/article/2082

SP - 128-130

TI - Ultra-wide band electromagnetic radiation does not affect UV-induced recombination and mutagenesis in yeast

ER -

TY - JOUR

IS - 6

JA - Radiat Res

JO - Radiation Research

PY - 1998

SN - 0033-7587

VL - 149

AU - Malyapa RS

AU - Ahern EW

AU - Bi C

AU - Straube WL

AU - LaRegina MC

AU - Pickard WF

AU - Roti Roti JL

LA - en

N1 - FEMU ID: 1374; EMF-Portal URL: https://www.emf-portal.org/en/article/1374

SP - 637-645

TI - DNA damage in rat brain cells after in vivo exposure to 2450 MHz electromagnetic radiation and various methods of euthanasia

ER -

TY - JOUR

IS - 6

JA - Wirel Netw

JO - Wireless Networks

PY - 1997

VL - 3

AU - Lai H

AU - Carino M

AU - Singh N

DO - 10.1023/A:1019154611749

LA - en

N1 - FEMU ID: 10681; EMF-Portal URL: https://www.emf-portal.org/en/article/10681

SP - 471-476

TI - Naltrexone blocks RFR-induced DNA double strand breaks in rat brain cells

UR - https://dl.acm.org/doi/pdf/10.1023/A%3A1019154611749

ER -

TY - JOUR

IS - 6

JA - Int J Radiat Biol

JO - International Journal of Radiation Biology

PY - 1997

SN - 0955-3002

VL - 72

AU - Vijayalaxmi

AU - Mohan N

AU - Meltz ML

AU - Wittler MA

DO - 10.1080/095530097142915

LA - en

N1 - FEMU ID: 2359; EMF-Portal URL: https://www.emf-portal.org/en/article/2359

SP - 751-757

TI - Proliferation and cytogenetic studies in human blood lymphocytes exposed in vitro to 2450 MHz radiofrequency radiation

ER -

TY - JOUR

IS - 2-3

JA - Mutat Res Genet Toxicol Environ Mutagen

JO - Mutation Research - Genetic Toxicology and Environmental Mutagenesis

PY - 1997

VL - 395

AU - Antonopoulos A

AU - Eisenbrandt H

AU - Obe G

DO - 10.1016/s1383-5718(97)00173-3

LA - en

N1 - FEMU ID: 2200; EMF-Portal URL: https://www.emf-portal.org/en/article/2200

SP - 209-214

TI - Effects of high-frequency electromagnetic fields on human lymphocytes in vitro

ER -

TY - JOUR

IS - 6

JA - Radiat Res

JO - Radiation Research

PY - 1997

SN - 0033-7587

VL - 148

AU - Malyapa RS

AU - Ahern EW

AU - Straube WL

AU - Moros EG

AU - Pickard WF

AU - Roti Roti JL

LA - en

N1 - FEMU ID: 2070; EMF-Portal URL: https://www.emf-portal.org/en/article/2070

SP - 608-617

TI - Measurement of DNA damage after exposure to 2450 MHz electromagnetic radiation

ER -

TY - JOUR

IS - 4

JA - Radiat Res

JO - Radiation Research

PY - 1997

SN - 0033-7587

VL - 147

AU - Vijayalaxmi

AU - Frei MR

AU - Dusch SJ

AU - Guel V

AU - Meltz ML

AU - Jauchem JR

LA - en

N1 - FEMU ID: 1413; EMF-Portal URL: https://www.emf-portal.org/en/article/1413

SP - 495-500

TI - Frequency of micronuclei in the peripheral blood and bone marrow of cancer-prone mice chronically exposed to 2450 MHz radiofrequency radiation

ER -

TY - JOUR

IS - 6

JO - Bioelectromagnetics

PY - 1997

SN - 0197-8462

VL - 18

AU - Lai H

AU - Singh NP

DO - 10.1002/(sici)1521-186x(1997)18:6<446::aid-bem7>3.0.co;2-2

LA - en

N1 - FEMU ID: 1257; EMF-Portal URL: https://www.emf-portal.org/en/article/1257

SP - 446-454

TI - Melatonin and a spin-trap compound block radiofrequency electromagnetic radiation-induced DNA strand breaks in rat brain cells

ER -

TY - JOUR

IS - 2

JA - Bioelectrochem Bioenerg

JO - Bioelectrochemistry and Bioenergetics

PY - 1997

SN - 0302-4598

VL - 43

AU - Pakhomova ON

AU - Pakhomov AG

AU - Akyel Y

DO - 10.1016/S0302-4598(96)05158-6

LA - en

N1 - FEMU ID: 963; EMF-Portal URL: https://www.emf-portal.org/en/article/963

SP - 227-232

TI - Effect of millimeter waves on UV-induced recombination and mutagenesis in yeast

ER -

TY - JOUR

IS - 1-2

JA - Mutat Res Genet Toxicol Environ Mutagen

JO - Mutation Research - Genetic Toxicology and Environmental Mutagenesis

PY - 1997

VL - 393

AU - Maes A

AU - Collier M

AU - Van Gorp U

AU - Vandoninck S

AU - Verschaeve L

DO - 10.1016/s1383-5718(97)00100-9

LA - en

N1 - FEMU ID: 948; EMF-Portal URL: https://www.emf-portal.org/en/article/948

SP - 151-156

TI - Cytogenetic effects of 935.2-MHz (GSM) microwaves alone and in combination with mitomycin C

ER -

TY - JOUR

IS - 6

JA - Radiat Res

JO - Radiation Research

PY - 1997

SN - 0033-7587

VL - 148

AU - Malyapa RS

AU - Ahern EW

AU - Straube WL

AU - Moros EG

AU - Pickard WF

AU - Roti Roti JL

LA - en

N1 - FEMU ID: 947; EMF-Portal URL: https://www.emf-portal.org/en/article/947

SP - 618-627

TI - Measurement of DNA damage after exposure to electromagnetic radiation in the cellular phone communication frequency band (835.62 and 847.74 MHz)

ER -

TY - JOUR

IS - 3

JO - Bioelectromagnetics

PY - 1997

SN - 0197-8462

VL - 18

AU - Ivaschuk OI

AU - Jones RA

AU - Ishida-Jones T

AU - Haggren W

AU - Adey WR

AU - Phillips JL

DO - 10.1002/(sici)1521-186x(1997)18:3<223::aid-bem4>3.0.co;2-4

LA - en

N1 - FEMU ID: 941; EMF-Portal URL: https://www.emf-portal.org/en/article/941

SP - 223-229

TI - Exposure of nerve growth factor-treated PC12 rat pheochromocytoma cells to a modulated radiofrequency field at 836.55 MHz: effects on c-jun and c-fos expression

ER -

TY - JOUR

IS - 3

JO - Bioelectromagnetics

PY - 1997

SN - 0197-8462

VL - 18

AU - Cain CD

AU - Thomas DL

AU - Adey WR

DO - 10.1002/(sici)1521-186x(1997)18:3<237::aid-bem6>3.0.co;2-3

LA - en

N1 - FEMU ID: 925; EMF-Portal URL: https://www.emf-portal.org/en/article/925

SP - 237-243

TI - Focus formation of C3H/10T1/2 cells and exposure to a 836.55 MHz modulated radiofrequency field

ER -

TY - JOUR

JO - Edition Wissenschaft

PY - 1996

VL - 4

AU - Eberle P

AU - Erdtmann-Vourliotis M

AU - Diener S

AU - Finke HG

AU - Löffelholz B

AU - Schnor A

AU - Schräder M

LA - de

N1 - FEMU ID: 9467; EMF-Portal URL: https://www.emf-portal.org/en/article/9467

SP - 2-15

TI - Zellproliferation, Schwesterchromatidaustausche, Chromosomenaberrationen, Mikrokerne und Mutationsrate des HGPRT-Locus

UR - https://d-nb.info/974863475/34

ER -

TY - JOUR

JO - Edition Wissenschaft

PY - 1996

VL - 8

AU - Hansen V

AU - Rüger W

LA - de

N1 - FEMU ID: 9463; EMF-Portal URL: https://www.emf-portal.org/en/article/9463

SP - 3-38

TI - Wirkung hochfrequenter elektromagnetischer Felder auf DNA, Proteine und DNA-Protein-Komplexe

UR - https://d-nb.info/974870188/34

ER -

TY - JOUR

IS - 1

JA - Sci Total Environ

JO - Science of the Total Evironment

PY - 1996

SN - 0048-9697

VL - 180

AU - Balode Z

DO - 10.1016/0048-9697(95)04923-1

LA - en

N1 - FEMU ID: 2132; EMF-Portal URL: https://www.emf-portal.org/en/article/2132

SP - 81-85

TI - Assessment of radio-frequency electromagnetic radiation by the micronucleus test in bovine peripheral erythrocytes

ER -

TY - JOUR

IS - 4

JA - Int J Radiat Biol

JO - International Journal of Radiation Biology

PY - 1996

SN - 0955-3002

VL - 69

AU - Lai H

AU - Singh NP

DO - 10.1080/095530096145814

LA - en

N1 - FEMU ID: 1389; EMF-Portal URL: https://www.emf-portal.org/en/article/1389

SP - 513-521

TI - Single- and double-strand DNA breaks in rat brain cells after acute exposure to radiofrequency electromagnetic radiation

ER -

TY - JOUR

IS - 2

JA - Electro Magnetobiol

JO - Electro- and Magnetobiology

PY - 1996

SN - 1061-9526

VL - 15

AU - Scarfi MR

AU - Lioi MB

AU - d'Ambrosio G

AU - Massa R

AU - Zeni O

AU - Di Pietto R

AU - Di Berardino D

DO - 10.3109/15368379609009826

LA - en

N1 - FEMU ID: 959; EMF-Portal URL: https://www.emf-portal.org/en/article/959

SP - 99-107

TI - Genotoxic effects of mitomycin-c and microwave radiation on bovine lymphocytes

ER -

TY - JOUR

IS - 1

JA - Environ Mol Mutagen

JO - Environmental and Molecular Mutagenesis

PY - 1996

SN - 0893-6692

VL - 28

AU - Maes A

AU - Collier M

AU - Slaets D

AU - Verschaeve L

DO - 10.1002/(SICI)1098-2280(1996)28:1<26::AID-EM6>3.0.CO;2-C

LA - en

N1 - FEMU ID: 934; EMF-Portal URL: https://www.emf-portal.org/en/article/934

SP - 26-30

TI - 954 MHz microwaves enhance the mutagenic properties of mitomycin C

ER -

TY - JOUR

IS - 3

JO - Bioelectromagnetics

PY - 1995

SN - 0197-8462

VL - 16

AU - Lai H

AU - Singh NP

DO - 10.1002/bem.2250160309

LA - en

N1 - FEMU ID: 1385; EMF-Portal URL: https://www.emf-portal.org/en/article/1385

SP - 207-210

TI - Acute low-intensity microwave exposure increases DNA single-strand breaks in rat brain cells

ER -

TY - JOUR

IS - 3

JA - Electro Magnetobiol

JO - Electro- and Magnetobiology

PY - 1995

SN - 1061-9526

VL - 14

AU - d'Ambrosio G

AU - Lioi MB

AU - Massa R

AU - Scarfi MR

AU - Zeni O

DO - 10.3109/15368379509030726

LA - en

N1 - FEMU ID: 905; EMF-Portal URL: https://www.emf-portal.org/en/article/905

SP - 157-164

TI - Genotoxic Effects of Amplitude-Modulated Microwaves on Human Lymphocytes Exposed in Vitro under Controlled Conditions

ER -

TY - JOUR

IS - 2

JA - Electro Magnetobiol

JO - Electro- and Magnetobiology

PY - 1995

SN - 1061-9526

VL - 14

AU - Maes A

AU - Collier M

AU - Slaets D

AU - Verschaeve L

LA - en

N1 - FEMU ID: 891; EMF-Portal URL: https://www.emf-portal.org/en/article/891

SP - 91-98

TI - Cytogenetic Effects of Microwaves from Mobile Communication Frequencies (954 MHz)

ER -

TY - JOUR

IS - 1

JA - Electro Magnetobiol

JO - Electro- and Magnetobiology

PY - 1994

SN - 1061-9526

VL - 13

AU - Belyaev IY

AU - Alipov YD

AU - Shcheglov VS

AU - Polunin VA

AU - Aizenberg OA

DO - 10.3109/15368379409030698

LA - en

N1 - FEMU ID: 8596; EMF-Portal URL: https://www.emf-portal.org/en/article/8596

SP - 53-66

TI - Cooperative response of Escherichia coli cells to the resonance effect of millimeter waves at super low intensity

ER -

TY - JOUR

IS - 1-2

JO - Mutation Research - Genetic Toxicology

PY - 1994

VL - 320

AU - Sarkar S

AU - Ali S

AU - Behari J

DO - 10.1016/0165-1218(94)90066-3

LA - en

N1 - FEMU ID: 1378; EMF-Portal URL: https://www.emf-portal.org/en/article/1378

SP - 141-147

TI - Effect of low power microwave on the mouse genome: a direct DNA analysis

ER -

TY - JOUR

IS - 1-2

JO - Mutation Research - Letters

PY - 1994

VL - 328

AU - Haider T

AU - Knasmueller S

AU - Kundi M

AU - Haider M

DO - 10.1016/0165-7992(94)90069-8

LA - en

N1 - FEMU ID: 873; EMF-Portal URL: https://www.emf-portal.org/en/article/873

SP - 65-68

TI - Clastogenic effects of radiofrequency radiations on chromosomes of Tradescantia

ER -

TY - JOUR

IS - 6

JA - Acta Radiol

JO - Acta Radiologica

PY - 1993

SN - 0284-1851

VL - 34

AU - Yamazaki E

AU - Matsubara S

AU - Yamada I

LA - en

N1 - FEMU ID: 22349; EMF-Portal URL: https://www.emf-portal.org/en/article/22349

SP - 607-611

TI - Effect of Gd-DTPA and/or magnetic field and radiofrequency exposure on sister chromatid exchange in human peripheral lymphocytes

ER -

TY - JOUR

IS - 1

JA - Electro Magnetobiol

JO - Electro- and Magnetobiology

PY - 1993

SN - 1061-9526

VL - 12

AU - Belyaev IY

AU - Alipov YD

AU - Polunin VA

AU - Shcheglov VS

DO - 10.3109/15368379309012861

LA - en

N1 - FEMU ID: 2116; EMF-Portal URL: https://www.emf-portal.org/en/article/2116

SP - 39-49

TI - Evidence for dependence of resonant frequency of millimeter wave interaction with Escherischia coli K12 cells on haploid genome length

ER -

TY - JOUR

JA - Bioelectrochem Bioenerg

JO - Bioelectrochemistry and Bioenergetics

PY - 1993

SN - 0302-4598

VL - 30

AU - Garaj-Vrhovac V

AU - Fucic A

DO - 10.1016/0302-4598(93)80091-8

LA - en

N1 - FEMU ID: 1874; EMF-Portal URL: https://www.emf-portal.org/en/article/1874

SP - 319-325

TI - The rate of elimination of chromosomal aberrations after accidental exposure to microwave radiation

ER -

TY - JOUR

IS - 6

JO - Bioelectromagnetics

PY - 1993

SN - 0197-8462

VL - 14

AU - Maes A

AU - Verschaeve L

AU - Arroyo A

AU - De Wagter C

AU - Vercruyssen L

DO - 10.1002/bem.2250140602

LA - en

N1 - FEMU ID: 889; EMF-Portal URL: https://www.emf-portal.org/en/article/889

SP - 495-501

TI - In vitro cytogenetic effects of 2450 MHz waves on human peripheral blood lymphocytes

ER -

TY - JOUR

IS - 7-8

JA - Z Naturforsch C

JO - Zeitschrift für Naturforschung C, Journal of Biosciences

PY - 1992

SN - 0341-0382

VL - 47

AU - Belyaev IY

AU - Alipov YD

AU - Shcheglov VS

AU - Lystsov VN

DO - 10.1515/znc-1992-7-822

LA - en

N1 - FEMU ID: 10177; EMF-Portal URL: https://www.emf-portal.org/en/article/10177

SP - 621-627

TI - Resonance effect of microwaves on the genome conformational state of E. coli cells

UR - https://www.degruyter.com/downloadpdf/j/znc.1992.47.issue-7-8/znc-1992-7-822/znc-1992-7-822.xml

ER -

TY - JOUR

IS - 2

JA - Electro Magnetobiol

JO - Electro- and Magnetobiology

PY - 1992

SN - 1061-9526

VL - 11

AU - Belyaev IY

AU - Alipov YD

AU - Shcheglov VS

DO - 10.3109/15368379209009820

LA - en

N1 - FEMU ID: 2115; EMF-Portal URL: https://www.emf-portal.org/en/article/2115

SP - 97-108

TI - Chromosome DNA as a target of resonant interaction between Escherichia coli cells and low-intensity millimeter waves

ER -

TY - JOUR

IS - 3

JO - Mutation Research - Letters

PY - 1992

VL - 281

AU - Garaj-Vrhovac V

AU - Fucic A

AU - Horvat D

DO - 10.1016/0165-7992(92)90006-4

LA - en

N1 - FEMU ID: 876; EMF-Portal URL: https://www.emf-portal.org/en/article/876

SP - 181-186

TI - The correlation between the frequency of micronuclei and specific chromosome aberrations in human lymphocytes exposed to microwave radiation in vitro

ER -

TY - JOUR

IS - 4

JO - Mutation Research - Letters

PY - 1992

VL - 282

AU - Fucic A

AU - Garaj-Vrhovac V

AU - Skara M

AU - Dimitrovic B

DO - 10.1016/0165-7992(92)90133-3

LA - en

N1 - FEMU ID: 875; EMF-Portal URL: https://www.emf-portal.org/en/article/875

SP - 265-271

TI - X-rays, microwaves and vinyl chloride monomer: their clastogenic and aneugenic activity, using the micronucleus assay on human lymphocytes

ER -

TY - JOUR

IS - 2

JA - Biochem Int

JO - Biochemistry International

PY - 1991

SN - 0158-5231

VL - 25

AU - Narasimhan V

AU - Huh WK

LA - en

N1 - FEMU ID: 8617; EMF-Portal URL: https://www.emf-portal.org/en/article/8617

SP - 363-370

TI - Altered restriction patterns of microwave irradiated lambda-phage DNA

ER -

TY - JOUR

IS - 5

JA - Med J Aust

JO - The Medical Journal of Australia

PY - 1991

SN - 0025-729X

VL - 155

AU - Garson OM

AU - McRobert TL

AU - Campbell LJ

AU - Hocking BA

AU - Gordon I

DO - 10.5694/j.1326-5377.1991.tb142282.x

LA - en

N1 - FEMU ID: 1407; EMF-Portal URL: https://www.emf-portal.org/en/article/1407

SP - 289-292

TI - A chromosomal study of workers with long-term exposure to radio-frequency radiation

ER -

TY - JOUR

IS - 3

JO - Mutation Research - Letters

PY - 1991

VL - 263

AU - Garaj-Vrhovac V

AU - Horvat D

AU - Koren Z

DO - 10.1016/0165-7992(91)90054-8

LA - en

N1 - FEMU ID: 877; EMF-Portal URL: https://www.emf-portal.org/en/article/877

SP - 143-149

TI - The relationship between colony-forming ability, chromosome aberrations and incidence of micronuclei in V79 Chinese hamster cells exposed to microwave radiation

ER -

TY - JOUR

IS - 4

JA - Period Biol

JO - Periodicum Biologorum

PY - 1990

SN - 0031-5362

VL - 92

AU - Garaj-Vrhovac V

AU - Fucic A

AU - Horvat D

LA - en

N1 - FEMU ID: 9585; EMF-Portal URL: https://www.emf-portal.org/en/article/9585

SP - 411-416

TI - Comparison of chromosome aberration and micronucleus induction in human lymphocytes after occupational exposure to vinyl chloride monomer and microwave radiation

ER -

TY - JOUR

IS - 3

JA - Radiat Res

JO - Radiation Research

PY - 1990

SN - 0033-7587

VL - 123

AU - Kerbacher JJ

AU - Meltz ML

AU - Erwin DN

LA - en

N1 - FEMU ID: 3403; EMF-Portal URL: https://www.emf-portal.org/en/article/3403

SP - 311-319

TI - Influence of radiofrequency radiation on chromosome aberrations in CHO cells and its interaction with DNA-damaging agents

ER -

TY - JOUR

IS - 2

JO - Bioelectromagnetics

PY - 1990

SN - 0197-8462

VL - 11

AU - Meltz ML

AU - Eagan P

AU - Erwin DN

DO - 10.1002/bem.2250110206

LA - en

N1 - FEMU ID: 888; EMF-Portal URL: https://www.emf-portal.org/en/article/888

SP - 149-157

TI - Proflavin and microwave radiation: absence of a mutagenic interaction

ER -

TY - JOUR

IS - 3

JO - Mutation Research - Letters

PY - 1990

VL - 243

AU - Garaj-Vrhovac V

AU - Horvat D

AU - Koren Z

DO - 10.1016/0165-7992(90)90028-i

LA - en

N1 - FEMU ID: 878; EMF-Portal URL: https://www.emf-portal.org/en/article/878

SP - 87-93

TI - The effect of microwave radiation on the cell genome

ER -

TY - JOUR

IS - 4

JA - Environ Mol Mutagen

JO - Environmental and Molecular Mutagenesis

PY - 1989

SN - 0893-6692

VL - 13

AU - Meltz ML

AU - Eagan P

AU - Erwin DN

DO - 10.1002/em.2850130404

LA - en

N1 - FEMU ID: 932; EMF-Portal URL: https://www.emf-portal.org/en/article/932

SP - 294-303

TI - Absence of mutagenic interaction between microwaves and mitomycin C in mammalian cells

ER -

TY - JOUR

IS - 6

JA - Int J Radiat Biol Relat Stud Phys Chem Med

JO - International Journal of Radiation Biology and Related Studies in Physics, Chemistry and Medicine

PY - 1988

SN - 0020-7616

VL - 53

AU - Saunders RD

AU - Kowalczuk CI

AU - Beechey CV

AU - Dunford R

DO - 10.1080/09553008814551341

LA - en

N1 - FEMU ID: 1408; EMF-Portal URL: https://www.emf-portal.org/en/article/1408

SP - 983-992

TI - Studies of the induction of dominant lethals and translocations in male mice after chronic exposure to microwave radiation

ER -

TY - JOUR

IS - 3

JA - Int J Biometeorol

JO - International Journal of Biometeorology

PY - 1987

SN - 0020-7128

VL - 31

AU - Levengood WC

DO - 10.1007/BF02188921

LA - en

N1 - FEMU ID: 2114; EMF-Portal URL: https://www.emf-portal.org/en/article/2114

SP - 185-190

TI - Non-disjunction mutations in Drosophila exposed to magnetic fields

ER -

TY - JOUR

IS - 2

JA - Radiat Res

JO - Radiation Research

PY - 1987

SN - 0033-7587

VL - 110

AU - Sagripanti JL

AU - Swicord ML

AU - Davis CC

DO - 10.2307/3576900

LA - en

N1 - FEMU ID: 2077; EMF-Portal URL: https://www.emf-portal.org/en/article/2077

SP - 219-231

TI - Microwave effects on plasmid DNA

ER -

TY - JOUR

IS - 4

JA - Environ Mutagen

JO - Environmental Mutagenesis

PY - 1987

SN - 0192-2521

VL - 9

AU - Ciaravino V

AU - Meltz ML

AU - Erwin DN

DO - 10.1002/em.2860090405

LA - en

N1 - FEMU ID: 2060; EMF-Portal URL: https://www.emf-portal.org/en/article/2060

SP - 393-399

TI - Effects of radiofrequency radiation and simultaneous exposure with mitomycin C on the frequency of sister chromatid exchanges in Chinese hamster ovary cells

ER -

TY - JOUR

IS - 1

JA - Int J Radiat Biol Relat Stud Phys Chem Med

JO - International Journal of Radiation Biology and Related Studies in Physics, Chemistry and Medicine

PY - 1986

SN - 0020-7616

VL - 50

AU - Sagripanti JL

AU - Swicord ML

DO - 10.1080/09553008614550431

LA - en

N1 - FEMU ID: 7147; EMF-Portal URL: https://www.emf-portal.org/en/article/7147

SP - 47-50

TI - DNA structural changes caused by microwave radiation

ER -

TY - JOUR

IS - 5

JA - Int J Radiat Biol Relat Stud Phys Chem Med

JO - International Journal of Radiation Biology and Related Studies in Physics, Chemistry and Medicine

PY - 1986

SN - 0020-7616

VL - 50

AU - Beechey CV

AU - Brooker D

AU - Kowalczuk CI

AU - Saunders RD

AU - Searle AG

DO - 10.1080/09553008614551321

LA - en

N1 - FEMU ID: 1388; EMF-Portal URL: https://www.emf-portal.org/en/article/1388

SP - 909-918

TI - Cytogenetic effects of microwave irradiation on male germ cells of the mouse

ER -

TY - JOUR

IS - 2

JO - Bioelectromagnetics

PY - 1986

SN - 0197-8462

VL - 7

AU - Lloyd DC

AU - Saunders RD

AU - Moquet JE

AU - Kowalczuk CI

DO - 10.1002/bem.2250070212

LA - en

N1 - FEMU ID: 893; EMF-Portal URL: https://www.emf-portal.org/en/article/893

SP - 235-237

TI - Absence of chromosomal damage in human lymphocytes exposed to microwave radiation with hyperthermia

ER -

TY - JOUR

IS - 1

JO - Radiology

PY - 1985

SN - 0033-8419

VL - 155

AU - Wolff S

AU - James TL

AU - Young GB

AU - Margulis AR

AU - Bodycote J

AU - Afzal V

DO - 10.1148/radiology.155.1.4038809

LA - en

N1 - FEMU ID: 22347; EMF-Portal URL: https://www.emf-portal.org/en/article/22347

SP - 163-165

TI - Magnetic resonance imaging: absence of in vitro cytogenetic damage

ER -

TY - JOUR

IS - 2-3

JO - Mutation Research - Genetic Toxicology

PY - 1985

VL - 157

AU - Marec F

AU - Ondracek J

AU - Brunnhofer V

DO - 10.1016/0165-1218(85)90112-0

LA - en

N1 - FEMU ID: 2121; EMF-Portal URL: https://www.emf-portal.org/en/article/2121

SP - 163-167

TI - The effect of repeated microwave irradiation on the frequency of sex-linked recessive lethal mutations in Drosophila melanogaster

ER -

TY - JOUR

IS - 6

JA - Int J Radiat Biol Relat Stud Phys Chem Med

JO - International Journal of Radiation Biology and Related Studies in Physics, Chemistry and Medicine

PY - 1985

SN - 0020-7616

VL - 48

AU - Dardalhon M

AU - Averbeck D

AU - Berteaud AJ

AU - Ravary V

DO - 10.1080/09553008514552111

LA - en

N1 - FEMU ID: 2043; EMF-Portal URL: https://www.emf-portal.org/en/article/2043

SP - 987-996

TI - Thermal aspects of biological effects of microwaves in Saccharomyces cerevisiae

ER -

TY - JOUR

IS - 1

JA - J Hered

JO - The Journal of Heredity

PY - 1985

SN - 0022-1503

VL - 76

AU - Manikowska-Czerska E

AU - Czerski P

AU - Leach WM

DO - 10.1093/oxfordjournals.jhered.a110027

LA - en

N1 - FEMU ID: 922; EMF-Portal URL: https://www.emf-portal.org/en/article/922

SP - 71-73

TI - Effects of 2.45 GHz microwaves on meiotic chromosomes of male CBA/CAY mice

ER -

TY - JOUR

IS - 2

JA - Int J Radiat Biol Relat Stud Phys Chem Med

JO - International Journal of Radiation Biology and Related Studies in Physics, Chemistry and Medicine

PY - 1984

SN - 0020-7616

VL - 46

AU - Lloyd DC

AU - Saunders RD

AU - Finnon P

AU - Kowalczuk CI

DO - 10.1080/09553008414551211

LA - en

N1 - FEMU ID: 13126; EMF-Portal URL: https://www.emf-portal.org/en/article/13126

SP - 135-141

TI - No clastogenic effect from in vitro microwave irradiation of G0 human lymphocytes

ER -

TY - JOUR

IS - 1

JO - Mutation Research - Letters

PY - 1982

VL - 103

AU - Goud SN

AU - Rani MV

AU - Reddy PP

AU - Reddi OS

AU - Rao MS

AU - Saxena VK

DO - 10.1016/0165-7992(82)90084-7

LA - en

N1 - FEMU ID: 2120; EMF-Portal URL: https://www.emf-portal.org/en/article/2120

SP - 39-42

TI - Genetic effects of microwave radiation in mice

ER -

TY - JOUR

IS - 4

JA - J Microw Power

JO - The Journal of Microwave Power

PY - 1982

SN - 0022-2739

VL - 17

AU - Dhahi SJ

AU - Habash RW

AU - Al-Hafid HT

DO - 10.1080/16070658.1982.11689289

LA - en

N1 - FEMU ID: 2106; EMF-Portal URL: https://www.emf-portal.org/en/article/2106

SP - 345-351

TI - Lack of mutagenic effects on conidia of Aspergillus amstelodami irradiated by 8.7175 GHz CW microwaves

ER -

TY - JOUR

IS - 2

JA - J Hered

JO - The Journal of Heredity

PY - 1982

SN - 0022-1503

VL - 73

AU - Yao KT

DO - 10.1093/oxfordjournals.jhered.a109596

LA - en

N1 - FEMU ID: 927; EMF-Portal URL: https://www.emf-portal.org/en/article/927

SP - 133-138

TI - Cytogenetic consequences of microwave irradiation on mammalian cells incubated in vitro

ER -

TY - JOUR

IS - 1

JA - Radiat Environ Biophys

JO - Radiation and Environmental Biophysics

PY - 1981

SN - 0301-634X

VL - 20

AU - Dardalhon M

AU - Averbeck D

AU - Berteaud AJ

DO - 10.1007/BF01323925

LA - en

N1 - FEMU ID: 2044; EMF-Portal URL: https://www.emf-portal.org/en/article/2044

SP - 37-51

TI - Studies on possible genetic effects of microwaves in procaryotic and eucaryotic cells

ER -

TY - JOUR

IS - 2

JA - Radiat Res

JO - Radiation Research

PY - 1981

SN - 0033-7587

VL - 85

AU - McRee DI

AU - MacNichols G

LA - en

N1 - FEMU ID: 1399; EMF-Portal URL: https://www.emf-portal.org/en/article/1399

SP - 340-348

TI - Incidence of sister chromatid exchange in bone marrow cells of the mouse following microwave exposure

ER -

TY - JOUR

IS - 3

JA - Radiat Res

JO - Radiation Research

PY - 1980

SN - 0033-7587

VL - 82

AU - Blevins RD

AU - Crenshaw Jr RC

AU - Hougland AE

AU - Clark CE

LA - en

N1 - FEMU ID: 9259; EMF-Portal URL: https://www.emf-portal.org/en/article/9259

SP - 511-517

TI - The effects of microwave radiation and heat on specific mutants of Salmonella typhimurium LT2

ER -

TY - JOUR

IS - 3

JO - Experientia

PY - 1979

SN - 0014-4754

VL - 35

AU - Manikowska E

AU - Luciani JM

AU - Servantie B

AU - Czerski P

AU - Obrenovitch J

AU - Stahl A

DO - 10.1007/BF01964370

LA - en

N1 - FEMU ID: 9731; EMF-Portal URL: https://www.emf-portal.org/en/article/9731

SP - 388-390

TI - Effects of 9.4 GHz microwave exposure on meiosis in mice

ER -

TY - JOUR

IS - 4

JA - J Microw Power

JO - The Journal of Microwave Power

PY - 1979

SN - 0022-2739

VL - 14

AU - Dardalhon M

AU - Averbeck D

AU - Berteaud AJ

DO - 10.1080/16070658.1979.11689165

LA - en

N1 - FEMU ID: 7859; EMF-Portal URL: https://www.emf-portal.org/en/article/7859

SP - 307-312

TI - Determination of a thermal equivalent of millimeter microwaves in living cells

ER -

TY - JOUR

IS - 3

JA - J Microw Power

JO - The Journal of Microwave Power

PY - 1979

SN - 0022-2739

VL - 14

AU - Dutta SK

AU - Nelson WH

AU - Blackman CF

AU - Brusick DJ

DO - 10.1080/16070658.1979.11689160

LA - en

N1 - FEMU ID: 5638; EMF-Portal URL: https://www.emf-portal.org/en/article/5638

SP - 275-280

TI - Lack of microbial genetic response to 2.45-GHz CW and 8.5- to 9.6-GHz pulsed microwaves

ER -

TY - JOUR

IS - 3

JO - Mutation Research - Genetic Toxicology

PY - 1979

VL - 68

AU - Hamnerius Y

AU - Olofsson H

AU - Rasmuson A

AU - Rasmuson B

DO - 10.1016/0165-1218(79)90153-8

LA - en

N1 - FEMU ID: 2042; EMF-Portal URL: https://www.emf-portal.org/en/article/2042

SP - 217-223

TI - A Negative Test for Mutagenic Action of Microwave Radiation in Drosophila melanogaster

ER -

TY - JOUR

IS - 1

JA - Can J Genet Cytol

JO - Canadian Journal of Genetics and Cytology

PY - 1978

SN - 0008-4093

VL - 20

AU - Alam MT

AU - Barthakur N

AU - Lambert NG

AU - Kasatiya SS

DO - 10.1139/g78-004

LA - en

N1 - FEMU ID: 933; EMF-Portal URL: https://www.emf-portal.org/en/article/933

SP - 23-30

TI - Cytological effects of microwave radiation in Chinese hamster cells in vitro

ER -

TY - JOUR

IS - 4

JA - J Hered

JO - The Journal of Heredity

PY - 1977

SN - 0022-1503

VL - 68

AU - Mittler S

DO - 10.1093/oxfordjournals.jhered.a108826

LA - en

N1 - FEMU ID: 1411; EMF-Portal URL: https://www.emf-portal.org/en/article/1411

SP - 257-258

TI - Failure of chronic exposure to nonthermal FM radio waves to mutate Drosophila

ER -

TY - JOUR

IS - 3

JA - Environ Res

JO - Environmental Research

PY - 1976

SN - 0013-9351

VL - 11

AU - Mittler S

DO - 10.1016/0013-9351(76)90094-3

LA - en

N1 - FEMU ID: 1410; EMF-Portal URL: https://www.emf-portal.org/en/article/1410

SP - 326-330

TI - Failure of 2- and 10-meter radio waves to induce genetic damage in Drosophila melanogaster

ER -

TY - JOUR

IS - 1

JA - Environ Lett

JO - Environmental Letters

PY - 1974

SN - 0013-9300

VL - 6

AU - Chen KM

AU - Samuel A

AU - Hoopingarner R

DO - 10.1080/00139307409437344

LA - en

N1 - FEMU ID: 7715; EMF-Portal URL: https://www.emf-portal.org/en/article/7715

SP - 37-46

TI - Chromosomal aberrations of living cells induced by microwave radiation

ER -

TY - JOUR

IS - 2

JA - Radiat Res

JO - Radiation Research

PY - 1973

SN - 0033-7587

VL - 56

AU - Hamrick PE

LA - en

N1 - FEMU ID: 2039; EMF-Portal URL: https://www.emf-portal.org/en/article/2039

SP - 400-404

TI - Letter: Thermal denaturation of DNA exposed to 2450 MHz CW microwave radiation

ER -
