# Supplementary material for: A scoping review and evidence map of radiofrequency field exposure and genotoxicity: assessing in vivo, in vitro, and epidemiological data
Source: Front Public Health. 2025 Jul 30;13:1613353. doi: 10.3389/fpubh.2025.1613353 (PMC12343714; doi:10.3389/fpubh.2025.1613353)
Supplement: Supplementary file 3 [file Data_Sheet_3.zip › Search data/EMF Portal Search - Micronuclei.docx]

EMF Portal search key words

The following terms were included: Mikronukleus, Mikrokern, MN, micronucleus, 小核, "micronuclei induction", "nuclear bud", "broken egg"

TY - JOUR

JA - Biomed Pharmacother

JO - Biomedicine & Pharmacotherapy

PY - 2022

SN - 0753-3322

VL - 155

AU - Zhang Y

AU - Li Q

AU - Wang C

AU - Zhu L

AU - Wang F

AU - Jiao W

AU - Zhuang X

AU - Xie F

AU - Du L

AU - Jin Y

DO - 10.1016/j.biopha.2022.113779

LA - en

N1 - FEMU ID: 50195; EMF-Portal URL: https://www.emf-portal.org/en/article/50195

SP - 113779

TI - Cinnarizine dissolving microneedles against microwave-induced brain injury

UR - https://www.sciencedirect.com/science/article/pii/S0753332222011684/pdfft?md5=f26c25755710d42ca38db2d0ae065622&pid=1-s2.0-S0753332222011684-main.pdf

ER -

TY - JOUR

IS - 2

JA - J Popul Ther Clin Pharmacol

JO - Journal of Population Therapeutics and Clinical Pharmacology

PY - 2022

SN - 1198-581X

VL - 29

AU - Mahmood MN

AU - Shaker AH

AU - Mohammed HE

DO - 10.47750/jptcp.2022.934

LA - en

N1 - FEMU ID: 47880; EMF-Portal URL: https://www.emf-portal.org/en/article/47880

SP - E79-E87

TI - Estimation of some antioxidants in people exposed to electromagnetic waves from Internet towers in Samarra

UR - https://www.jptcp.com/index.php/jptcp/article/view/934/895

ER -

TY - JOUR

IS - 1

JA - Saudi J Biol Sci

JO - Saudi Journal of Biological Sciences

PY - 2022

SN - 1319-562X

VL - 29

AU - Hasan I

AU - Jahan MR

AU - Islam MN

AU - Islam MR

DO - 10.1016/j.sjbs.2021.08.063

LA - en

N1 - FEMU ID: 46443; EMF-Portal URL: https://www.emf-portal.org/en/article/46443

SP - 102-110

TI - Effect of 2400 MHz mobile phone radiation exposure on the behavior and hippocampus morphology in Swiss mouse model

UR - https://www.sciencedirect.com/science/article/pii/S1319562X21007518/pdfft?md5=faa25562f821496cb73104fa28106e6f&pid=1-s2.0-S1319562X21007518-main.pdf

ER -

TY - JOUR

IS - 7

JA - Chin J Integr Med

JO - Chinese Journal of Integrative Medicine

PY - 2022

SN - 1672-0415

VL - 28

AU - Yin L

AU - Fan SJ

AU - Zhang MN

DO - 10.1007/s11655-021-3527-y

LA - en

N1 - FEMU ID: 46037; EMF-Portal URL: https://www.emf-portal.org/en/article/46037

SP - 620-626

TI - Protective Effects of Anthocyanins Extracted from Vaccinium Uliginosum on 661W Cells Against Microwave-Induced Retinal Damage

ER -

TY - JOUR

IS - 4

JA - J Int Oral Health

JO - Journal of International Oral Health

PY - 2021

SN - 0976-1799

VL - 13

AU - Thamilselvan S

AU - Behera A

AU - Nair SK

AU - Chandru CSL

AU - Krishnakumar M

AU - Ramani P

DO - 10.4103/JIOH.JIOH_358_20

LA - en

N1 - FEMU ID: 48844; EMF-Portal URL: https://www.emf-portal.org/en/article/48844

SP - 350-355

TI - Micronuclei analysis in people residing within 25 m of radiation-exposed areas around mobile towers in Chennai, India: An observational study

ER -

TY - JOUR

JA - Int J Prev Med

JO - International Journal of Preventive Medicine

PY - 2021

SN - 2008-7802

VL - 12

AU - Ghandehari M

AU - Sadri D

AU - Farhadi S

DO - 10.4103/ijpvm.IJPVM_489_19

LA - en

N1 - FEMU ID: 46095; EMF-Portal URL: https://www.emf-portal.org/en/article/46095

SP - 125

TI - Micronucleus Assay in Cell Phone Users: Importance of Oral Mucosa Screening

UR - https://www.ijpvmjournal.net/temp/IntJPrevMed121125-3231313_085833.pdf

ER -

TY - JOUR

JA - Front Public Health

JO - Frontiers in Public Health

PY - 2021

SN - 2296-2565

VL - 9

AU - Sieroń K

AU - Knapik K

AU - Onik G

AU - Romuk E

AU - Birkner E

AU - Kwiatek S

AU - Sieroń A

DO - 10.3389/fpubh.2021.710484

LA - en

N1 - FEMU ID: 45739; EMF-Portal URL: https://www.emf-portal.org/en/article/45739

SP - 710484

TI - Electromagnetic Fields Modify Redox Balance in the Rat Gastrointestinal Tract

UR - https://www.frontiersin.org/articles/10.3389/fpubh.2021.710484/pdf

ER -

TY - GEN

ET - 1

PB - IEEE

PY - 2020

SN - 9781728158679

T2 - 2020 IEEE MTT-S International Microwave Biomedical Conference (IMBioC), Toulouse, France

AU - Kolosnjaj-Tabi J

AU - Alberola G

AU - Augé S

AU - Tamra A

AU - Dubuc D

AU - Grenier K

AU - Rols MP

DO - 10.1109/IMBIoC47321.2020.9385011

LA - en

N1 - FEMU ID: 44633; EMF-Portal URL: https://www.emf-portal.org/en/article/44633

SP - 1-3

TI - Evaluation of Cell Membrane Effects After 3D Multicellular Spheroids RF Exposure

ER -

TY - JOUR

IS - 5

JA - Indian J Dent Res

JO - Indian Journal of Dental Research

PY - 2020

SN - 0970-9290

VL - 31

AU - Rashmi B

AU - Chinna SK

AU - Rodrigues C

AU - Anjaly D

AU - Bankur PK

AU - Kannaiyan K

DO - 10.4103/ijdr.IJDR_634_18

LA - en

N1 - FEMU ID: 44017; EMF-Portal URL: https://www.emf-portal.org/en/article/44017

SP - 734-737

TI - Occurrence of micronuclei in exfoliated buccal mucosal cells in mobile phone users: A case-control study

UR - https://www.ijdr.in/article.asp?issn=0970-9290;year=2020;volume=31;issue=5;spage=734;epage=737;aulast=Rashmi

ER -

TY - JOUR

IS - 5

JA - J Anim Physiol Anim Nutr (Berl)

JO - Journal of Animal Physiology and Animal Nutrition

PY - 2020

SN - 0931-2439

VL - 104

AU - Azimzadeh M

AU - Jelodar G

DO - 10.1111/jpn.13360

LA - en

N1 - FEMU ID: 42238; EMF-Portal URL: https://www.emf-portal.org/en/article/42238

SP - 1568-1574

TI - Trace elements homeostasis in brain exposed to 900 MHz RFW emitted from a BTS-antenna model and the protective role of vitamin E

ER -

TY - JOUR

IS - 2

JA - Environ Mol Mutagen

JO - Environmental and Molecular Mutagenesis

PY - 2020

SN - 0893-6692

VL - 61

AU - Smith-Roe SL

AU - Wyde ME

AU - Stout MD

AU - Winters JW

AU - Hobbs CA

AU - Shepard KG

AU - Green AS

AU - Kissling GE

AU - Shockley KR

AU - Tice RR

AU - Bucher JR

AU - Witt KL

DO - 10.1002/em.22343

LA - en

N1 - FEMU ID: 39949; EMF-Portal URL: https://www.emf-portal.org/en/article/39949

SP - 276-290

TI - Evaluation of the genotoxicity of cell phone radiofrequency radiation in male and female rats and mice following subchronic exposure

ER -

TY - JOUR

IS - 1

JA - IEEE J Electromagn RF Microw Med Biol

JO - IEEE Journal of Electromagnetics, RF and Microwaves in Medicine and Biology

PY - 2020

VL - 4

AU - Romeo S

AU - Sannino A

AU - Zeni O

AU - Angrisani L

AU - Massa R

AU - Scarfi MR

DO - 10.1109/JERM.2019.2918023

LA - en

N1 - FEMU ID: 38441; EMF-Portal URL: https://www.emf-portal.org/en/article/38441

SP - 17-23

TI - Effects of Radiofrequency Exposure and Co-Exposure on Human Lymphocytes: the Influence of Signal Modulation and Bandwidth

ER -

TY - JOUR

IS - 1

JA - Egypt J Rad Sci Applic

JO - Egyptian Journal of Radiation Sciences and Applications

PY - 2019

SN - 1110-0303

VL - 32

AU - Shedid SM

AU - El-Tawill GA

AU - Algeda FR

AU - El-Fatih NN

AU - Eltahawy NA

DO - 10.21608/EJRSA.2019.7814.1063

LA - en

N1 - FEMU ID: 46380; EMF-Portal URL: https://www.emf-portal.org/en/article/46380

SP - 51-60

TI - The Impact of 950MHz Electromagnetic Radiation on the Brain and Liver of Rats and the Role of Garlic Treatment

UR - https://ejrsa.journals.ekb.eg/article_31561_d52dbf6882859df535be3cb2874ce239.pdf

ER -

TY - JOUR

IS - 4

JA - Histol Histopathol

JO - Histology and Histopathology

PY - 2019

SN - 0213-3911

VL - 34

AU - Šimaiová V

AU - Almášiová V

AU - Holovská K

AU - Kisková T

AU - Horváthová F

AU - Ševčíková Z

AU - Tóth Š

AU - Raček A

AU - Račeková E

AU - Beňová K

AU - Dvořák P

AU - Cigánková V

DO - 10.14670/HH-18-049

LA - en

N1 - FEMU ID: 45874; EMF-Portal URL: https://www.emf-portal.org/en/article/45874

SP - 391-403

TI - The effect of 2.45 GHz non-ionizing radiation on the structure and ultrastructure of the testis in juvenile rats

ER -

TY - JOUR

IS - 4

JA - J Radiat Res

JO - Journal of Radiation Research

PY - 2019

SN - 0449-3060

VL - 60

AU - Koyama S

AU - Narita E

AU - Suzuki Y

AU - Shiina T

AU - Taki M

AU - Shinohara N

AU - Miyakoshi J

DO - 10.1093/jrr/rrz017

LA - en

N1 - FEMU ID: 38589; EMF-Portal URL: https://www.emf-portal.org/en/article/38589

SP - 417-423

TI - Long-term exposure to a 40-GHz electromagnetic field does not affect genotoxicity or heat shock protein expression in HCE-T or SRA01/04 cells

UR - https://academic.oup.com/jrr/article-pdf/60/4/417/28964321/rrz017.pdf

ER -

TY - JOUR

JA - Environ Res

JO - Environmental Research

PY - 2019

SN - 0013-9351

VL - 174

AU - Jooyan N

AU - Goliaei B

AU - Bigdeli B

AU - Faraji-Dana R

AU - Zamani A

AU - Entezami M

AU - Mortazavi SMJ

DO - 10.1016/j.envres.2019.03.063

LA - en

N1 - FEMU ID: 38182; EMF-Portal URL: https://www.emf-portal.org/en/article/38182

SP - 176-187

TI - Direct and indirect effects of exposure to 900 MHz GSM radiofrequency electromagnetic fields on CHO cell line: Evidence of bystander effect by non-ionizing radiation

ER -

TY - JOUR

IS - 2

JA - IEEE Trans Nanobioscience

JO - IEEE Transactions on Nanobioscience

PY - 2019

SN - 1536-1241

VL - 18

AU - Miyakoshi J

AU - Tonomura H

AU - Koyama S

AU - Narita E

AU - Shinohara N

DO - 10.1109/TNB.2019.2905491

LA - en

N1 - FEMU ID: 37795; EMF-Portal URL: https://www.emf-portal.org/en/article/37795

SP - 257-260

TI - Effects of Exposure to 5.8 GHz Electromagnetic Field on Micronucleus Formation, DNA Strand Breaks, and Heat Shock Protein Expressions in Cells Derived From Human Eye

ER -

TY - JOUR

IS - 3

JA - J Oral Maxillofac Pathol

JO - Journal of Oral and Maxillofacial Pathology: JOMFP

PY - 2018

SN - 0973-029X

VL - 22

AU - Vanishree M

AU - Manvikar V

AU - Rudraraju A

AU - Reddy KMP

AU - Kumar NHP

AU - Quadri SJM

DO - 10.4103/jomfp.JOMFP_201_18

LA - en

N1 - FEMU ID: 37117; EMF-Portal URL: https://www.emf-portal.org/en/article/37117

SP - 448

TI - Significance of micronuclei in buccal smears of mobile phone users: A comparative study

UR - http://www.jomfp.in/article.asp?issn=0973-029X;year=2018;volume=22;issue=3;spage=448;epage=448;aulast=Vanishree

ER -

TY - JOUR

IS - 1

JA - Health Phys

JO - Health Physics

PY - 2018

SN - 0017-9078

VL - 115

AU - Franchini V

AU - Regalbuto E

AU - De Amicis A

AU - De Sanctis S

AU - Di Cristofaro S

AU - Coluzzi E

AU - Marinaccio J

AU - Sgura A

AU - Ceccuzzi S

AU - Doria A

AU - Gallerano GP

AU - Giovenale E

AU - Ravera GL

AU - Bei R

AU - Benvenuto M

AU - Modesti A

AU - Masuelli L

AU - Lista F

DO - 10.1097/HP.0000000000000871

LA - en

N1 - FEMU ID: 35185; EMF-Portal URL: https://www.emf-portal.org/en/article/35185

SP - 126-139

TI - Genotoxic Effects in Human Fibroblasts Exposed to Microwave Radiation

ER -

TY - JOUR

IS - 6

JA - Environ Mol Mutagen

JO - Environmental and Molecular Mutagenesis

PY - 2018

SN - 0893-6692

VL - 59

AU - Franchini V

AU - De Sanctis S

AU - Marinaccio J

AU - De Amicis A

AU - Coluzzi E

AU - Di Cristofaro S

AU - Lista F

AU - Regalbuto E

AU - Doria A

AU - Giovenale E

AU - Gallerano GP

AU - Bei R

AU - Benvenuto M

AU - Masuelli L

AU - Udroiu I

AU - Sgura A

DO - 10.1002/em.22192

LA - en

N1 - FEMU ID: 34868; EMF-Portal URL: https://www.emf-portal.org/en/article/34868

SP - 476-487

TI - Study of the effects of 0.15 terahertz radiation on genome integrity of adult fibroblasts

UR - https://onlinelibrary.wiley.com/doi/epdf/10.1002/em.22192

ER -

TY - JOUR

IS - 10

JA - Int J Radiat Biol

JO - International Journal of Radiation Biology

PY - 2018

SN - 0955-3002

VL - 94

AU - Herrala M

AU - Mustafa E

AU - Naarala J

AU - Juutilainen J

DO - 10.1080/09553002.2018.1450534

LA - en

N1 - FEMU ID: 34733; EMF-Portal URL: https://www.emf-portal.org/en/article/34733

SP - 883-889

TI - Assessment of genotoxicity and genomic instability in rat primary astrocytes exposed to 872 MHz radiofrequency radiation and chemicals

ER -

TY - JOUR

IS - 1

JA - Pol J Environ Stud

JO - Polish Journal of Environmental Studies

PY - 2017

SN - 1230-1485

VL - 26

AU - Sieron-Stoltny K

AU - Pasek J

AU - Cieslar G

AU - Sieron A

DO - 10.15244/pjoes/64747

LA - en

N1 - FEMU ID: 46062; EMF-Portal URL: https://www.emf-portal.org/en/article/46062

SP - 279-285

TI - Influence of Electromagnetic Fields on Prooxidant/Antioxidant Balance in Rat Liver

UR - http://www.pjoes.com/pdf-64746-23951?filename=Increasing%20Area%20of.pdf

ER -

TY - JOUR

JA - Data Brief

JO - Data in Brief

PY - 2017

SN - 2352-3409

VL - 15

AU - de Oliveira FM

AU - Carmona AM

AU - Ladeira C

DO - 10.1016/j.dib.2017.09.048

LA - en

N1 - FEMU ID: 34141; EMF-Portal URL: https://www.emf-portal.org/en/article/34141

SP - 344-347

TI - Genotoxicity assessment data for exfoliated buccal cells exposed to mobile phone radiation

UR - https://www.sciencedirect.com/science/article/pii/S2352340917304791/pdfft?md5=cf92857ee630b0a186210e76b366ce6d&pid=1-s2.0-S2352340917304791-main.pdf

ER -

TY - JOUR

JA - Mutat Res Genet Toxicol Environ Mutagen

JO - Mutation Research - Genetic Toxicology and Environmental Mutagenesis

PY - 2017

VL - 822

AU - de Oliveira FM

AU - Carmona AM

AU - Ladeira C

DO - 10.1016/j.mrgentox.2017.08.001

LA - en

N1 - FEMU ID: 32942; EMF-Portal URL: https://www.emf-portal.org/en/article/32942

SP - 41-46

TI - Is mobile phone radiation genotoxic? An analysis of micronucleus frequency in exfoliated buccal cells

ER -

TY - JOUR

IS - 3

JA - Electromagn Biol Med

JO - Electromagnetic Biology and Medicine

PY - 2017

SN - 1536-8386

VL - 36

AU - Zothansiama

AU - Zosangzuali M

AU - Lalramdinpuii M

AU - Jagetia GC

DO - 10.1080/15368378.2017.1350584

LA - en

N1 - FEMU ID: 32665; EMF-Portal URL: https://www.emf-portal.org/en/article/32665

SP - 295-305

TI - Impact of radiofrequency radiation on DNA damage and antioxidants in peripheral blood lymphocytes of humans residing in the vicinity of mobile phone base stations

ER -

TY - JOUR

IS - 4

JA - Saudi J Biol Sci

JO - Saudi Journal of Biological Sciences

PY - 2017

SN - 1319-562X

VL - 24

AU - Qureshi ST

AU - Memon SA

AU - Abassi AR

AU - Sial MA

AU - Bughio FA

DO - 10.1016/j.sjbs.2016.02.011

LA - en

N1 - FEMU ID: 31864; EMF-Portal URL: https://www.emf-portal.org/en/article/31864

SP - 883-891

TI - Radiofrequency radiations induced genotoxic and carcinogenic effects on chickpea (Cicer arietinum L.) root tip cells

UR - https://www.sciencedirect.com/science/article/pii/S1319562X16000589/pdfft?md5=275ab70a0cf42609a2a27cd618810be3&pid=1-s2.0-S1319562X16000589-main.pdf

ER -

TY - JOUR

JA - Toxicol In Vitro

JO - Toxicology in Vitro

PY - 2017

SN - 0887-2333

VL - 40

AU - Al-Serori H

AU - Kundi M

AU - Ferk F

AU - Mišík M

AU - Nersesyan A

AU - Murbach M

AU - Lah TT

AU - Knasmüller S

DO - 10.1016/j.tiv.2017.01.012

LA - en

N1 - FEMU ID: 31101; EMF-Portal URL: https://www.emf-portal.org/en/article/31101

SP - 264-271

TI - Evaluation of the potential of mobile phone specific electromagnetic fields (UMTS) to produce micronuclei in human glioblastoma cell lines

ER -

TY - JOUR

IS - 4

JO - Bioelectromagnetics

PY - 2017

SN - 0197-8462

VL - 38

AU - Sannino A

AU - Zeni O

AU - Romeo S

AU - Massa R

AU - Scarfi MR

DO - 10.1002/bem.22034

LA - en

N1 - FEMU ID: 30897; EMF-Portal URL: https://www.emf-portal.org/en/article/30897

SP - 245-254

TI - Adverse and beneficial effects in Chinese hamster lung fibroblast cells following radiofrequency exposure

ER -

TY - JOUR

IS - 8

JA - Int J Environ Res Public Health

JO - International Journal of Environmental Research and Public Health

PY - 2016

SN - 1660-4601

VL - 13

AU - Koyama S

AU - Narita E

AU - Shimizu Y

AU - Suzuki Y

AU - Shiina T

AU - Taki M

AU - Shinohara N

AU - Miyakoshi J

DO - 10.3390/ijerph13080802

LA - en

N1 - FEMU ID: 32667; EMF-Portal URL: https://www.emf-portal.org/en/article/32667

SP - E802

TI - Effects of Long-Term Exposure to 60 GHz Millimeter-Wavelength Radiation on the Genotoxicity and Heat Shock Protein (Hsp) Expression of Cells Derived from Human Eye

UR - https://www.ncbi.nlm.nih.gov/pmc/articles/PMC4997488/pdf/ijerph-13-00802.pdf

ER -

TY - JOUR

IS - 7

JA - J Stroke Cerebrovasc Dis

JO - Journal of Stroke and Cerebrovascular Diseases

PY - 2016

SN - 1052-3057

VL - 25

AU - Fan Y

AU - Zhang C

AU - Li T

AU - Peng W

AU - Yin J

AU - Li X

AU - Kong Y

AU - Lan C

AU - Wang R

AU - Hu Z

DO - 10.1016/j.jstrokecerebrovasdis.2016.03.033

LA - en

N1 - FEMU ID: 31826; EMF-Portal URL: https://www.emf-portal.org/en/article/31826

SP - 1813-1822

TI - A New Approach of Short Wave Protection against Middle Cerebral Artery Occlusion/Reperfusion Injury via Attenuation of Golgi Apparatus Stress by Inhibition of Downregulation of Secretory Pathway Ca(2+)-ATPase Isoform 1 in Rats

ER -

TY - JOUR

IS - 8

JA - Int J Environ Res Public Health

JO - International Journal of Environmental Research and Public Health

PY - 2016

SN - 1660-4601

VL - 13

AU - Koyama S

AU - Narita E

AU - Shimizu Y

AU - Shiina T

AU - Taki M

AU - Shinohara N

AU - Miyakoshi J

DO - 10.3390/ijerph13080793

LA - en

N1 - FEMU ID: 30171; EMF-Portal URL: https://www.emf-portal.org/en/article/30171

SP - E793-

TI - Twenty four-hour exposure to a 0.12 THz electromagnetic field does not affect the genotoxicity, morphological changes, or expression of heat shock protein in HCE-T cells

UR - http://www.mdpi.com/1660-4601/13/8/793/pdf

ER -

TY - JOUR

IS - 3

JA - J Clin of Diagn Res

JO - Journal of Clinical and Diagnostic Research

PY - 2016

SN - 0973-709X

VL - 10

AU - Banerjee S

AU - Singh NN

AU - Sreedhar G

AU - Mukherjee S

DO - 10.7860/JCDR/2016/17592.7505

LA - en

N1 - FEMU ID: 29426; EMF-Portal URL: https://www.emf-portal.org/en/article/29426

SP - ZC82-ZC85

TI - Analysis of the Genotoxic Effects of Mobile Phone Radiation using Buccal Micronucleus Assay: A Comparative Evaluation

UR - https://www.ncbi.nlm.nih.gov/pmc/articles/PMC4843394/pdf/jcdr-10-ZC82.pdf

ER -

TY - JOUR

IS - 2

JO - Mutagenesis

PY - 2016

SN - 0267-8357

VL - 31

AU - Gustavino B

AU - Carboni G

AU - Petrillo R

AU - Paoluzzi G

AU - Santovetti E

AU - Rizzoni M

DO - 10.1093/mutage/gev071

LA - en

N1 - FEMU ID: 28074; EMF-Portal URL: https://www.emf-portal.org/en/article/28074

SP - 187-192

TI - Exposure to 915 MHz radiation induces micronuclei in Vicia faba root tips

UR - https://academic.oup.com/mutage/article-pdf/31/2/187/8178338/gev071.pdf

ER -

TY - JOUR

IS - 3

JA - Arch Environ Contam Toxicol

JO - Archives of Environmental Contamination and Toxicology

PY - 2016

SN - 0090-4341

VL - 70

AU - Gulati S

AU - Yadav A

AU - Kumar N

AU - Kanupriya

AU - Aggarwal NK

AU - Kumar R

AU - Gupta R

DO - 10.1007/s00244-015-0195-y

LA - en

N1 - FEMU ID: 27671; EMF-Portal URL: https://www.emf-portal.org/en/article/27671

SP - 615-625

TI - Effect of GSTM1 and GSTT1 polymorphisms on genetic damage in humans populations exposed to radiation from mobile towers

ER -

TY - JOUR

IS - 4

JA - Int J Hum Genet

JO - International Journal of Human Genetics

PY - 2015

SN - 0972-3757

VL - 15

AU - Gandhi G

AU - Singh P

AU - Kaur G

DO - 10.1080/09723757.2015.11886265

LA - en

N1 - FEMU ID: 47619; EMF-Portal URL: https://www.emf-portal.org/en/article/47619

SP - 173-182

TI - Perspectives Revisited - The Buccal Cytome Assay in Mobile Phone Users

UR - http://krepublishers.com/02-Journals/IJHG/IJHG-15-0-000-15-Web/IJHG-15-4-000-15-Abst-PDF/IJHG-15-4-173-15-606-Gandhi-G/IJHG-15-4-173-15-606-Gandhi-G-Tx[2].pdf

ER -

TY - JOUR

JA - Mutat Res Genet Toxicol Environ Mutagen

JO - Mutation Research - Genetic Toxicology and Environmental Mutagenesis

PY - 2015

VL - 793

AU - Amicis A

AU - Sanctis S

AU - Cristofaro SD

AU - Franchini V

AU - Lista F

AU - Regalbuto E

AU - Giovenale E

AU - Gallerano GP

AU - Nenzi P

AU - Bei R

AU - Fantini M

AU - Benvenuto M

AU - Masuelli L

AU - Coluzzi E

AU - Cicia C

AU - Sgura A

DO - 10.1016/j.mrgentox.2015.06.003

LA - en

N1 - FEMU ID: 28170; EMF-Portal URL: https://www.emf-portal.org/en/article/28170

SP - 150-160

TI - Biological effects of in vitro THz radiation exposure in human foetal fibroblasts

ER -

TY - JOUR

IS - 1

JA - Braz Oral Res

JO - Brazilian Oral Research

PY - 2015

SN - 1806-8324

VL - 29

AU - Daroit NB

AU - Visioli F

AU - Magnusson AS

AU - Vieira GR

AU - Rados PV

DO - 10.1590/1807-3107BOR-2015.vol29.0114

LA - en

N1 - FEMU ID: 28094; EMF-Portal URL: https://www.emf-portal.org/en/article/28094

SP - 1-8

TI - Cell phone radiation effects on cytogenetic abnormalities of oral mucosal cells

UR - http://www.scielo.br/pdf/bor/v29n1/1807-3107-bor-29-1-1807-3107BOR-2015vol290114.pdf

ER -

TY - JOUR

IS - 3

JA - Endocr Regul

JO - Endocrine Regulations

PY - 2015

SN - 1210-0668

VL - 49

AU - Gurbuz N

AU - Sirav B

AU - Kuzay D

AU - Ozer C

AU - Seyhan N

DO - 10.4149/endo_2015_03_126

LA - en

N1 - FEMU ID: 27666; EMF-Portal URL: https://www.emf-portal.org/en/article/27666

SP - 126-130

TI - Does radio frequency radiation induce micronuclei frequency in exfoliated bladder cells of diabetic rats?

ER -

TY - JOUR

IS - 4

JA - Int J Environ Res Public Health

JO - International Journal of Environmental Research and Public Health

PY - 2015

SN - 1660-4601

VL - 12

AU - Mizuno K

AU - Shinohara N

AU - Miyakoshi J

DO - 10.3390/ijerph120403853

LA - en

N1 - FEMU ID: 26850; EMF-Portal URL: https://www.emf-portal.org/en/article/26850

SP - 3853-3863

TI - In Vitro Evaluation of Genotoxic Effects under Magnetic Resonant Coupling Wireless Power Transfer

UR - http://www.mdpi.com/1660-4601/12/4/3853

ER -

TY - JOUR

IS - 2

JO - Bioelectromagnetics

PY - 2015

SN - 0197-8462

VL - 36

AU - Halgamuge MN

AU - Yak SK

AU - Eberhardt JL

DO - 10.1002/BEM.21890

LA - en

N1 - FEMU ID: 26476; EMF-Portal URL: https://www.emf-portal.org/en/article/26476

SP - 87-95

TI - Reduced growth of soybean seedlings after exposure to weak microwave radiation from GSM 900 mobile phone and base station

ER -

TY - GEN

ET - 1

PB - IEEE

PY - 2014

SN - 9781479956913

T2 - 2014 IEEE 5th Control and System Graduate Research Colloquium, Shah Alam, Malaysia

AU - Ma YP

AU - Haron MH

AU - Taib MN

AU - Jailani R

DO - 10.1109/ICSGRC.2014.6908738

LA - en

N1 - FEMU ID: 49747; EMF-Portal URL: https://www.emf-portal.org/en/article/49747

SP - 285-289

TI - Emotional identification during mobile RF Radiation

ER -

TY - JOUR

IS - 4

JO - Trace Elements and Electrolytes

PY - 2014

SN - 0946-2104

VL - 31

AU - Zhang Y

AU - An K

AU - Yin MS

AU - Yin LL

AU - Guo DM

AU - Cui X

DO - 10.5414/TEX01352

LA - en

N1 - FEMU ID: 31253; EMF-Portal URL: https://www.emf-portal.org/en/article/31253

SP - 174-180

TI - Effect of radiofrequency electromagnetic radiation emitted by mobile phone on trace elements levels in fetal rats

ER -

TY - JOUR

IS - 4

JA - Electromagn Biol Med

JO - Electromagnetic Biology and Medicine

PY - 2014

SN - 1536-8386

VL - 33

AU - Gurbuz N

AU - Sirav B

AU - Colbay M

AU - Yetkin I

AU - Seyhan N

DO - 10.3109/15368378.2013.831354

LA - en

N1 - FEMU ID: 23927; EMF-Portal URL: https://www.emf-portal.org/en/article/23927

SP - 296-301

TI - No genotoxic effect in exfoliated bladder cells of rat under the exposure of 1800 and 2100 MHz radio frequency radiation

ER -

TY - JOUR

IS - 2

JA - J Radiat Res

JO - Journal of Radiation Research

PY - 2014

SN - 0449-3060

VL - 55

AU - Kang KA

AU - Lee HC

AU - Lee JJ

AU - Hong MN

AU - Park MJ

AU - Lee YS

AU - Choi HD

AU - Kim N

AU - Ko YK

AU - Lee JS

DO - 10.1093/jrr/rrt116

LA - en

N1 - FEMU ID: 23673; EMF-Portal URL: https://www.emf-portal.org/en/article/23673

SP - 265-276

TI - Effects of combined radiofrequency radiation exposure on levels of reactive oxygen species in neuronal cells

UR - https://academic.oup.com/jrr/article-pdf/55/2/265/2803067/rrt116.pdf

ER -

TY - JOUR

IS - 2

JA - J Radiat Res

JO - Journal of Radiation Research

PY - 2014

SN - 0449-3060

VL - 55

AU - Sannino A

AU - Zeni O

AU - Romeo S

AU - Massa R

AU - Gialanella G

AU - Grossi G

AU - Manti L

AU - Vijayalaxmi

AU - Scarfi MR

DO - 10.1093/jrr/rrt106

LA - en

N1 - FEMU ID: 23403; EMF-Portal URL: https://www.emf-portal.org/en/article/23403

SP - 210-217

TI - Adaptive response in human blood lymphocytes exposed to non-ionizing radiofrequency fields: resistance to ionizing radiation-induced damage

UR - https://academic.oup.com/jrr/article-pdf/55/2/210/2797100/rrt106.pdf

ER -

TY - JOUR

IS - 2

JA - Cell Biochem Biophys

JO - Cell Biochemistry and Biophysics

PY - 2014

SN - 1085-9195

VL - 68

AU - Kesari KK

AU - Meena R

AU - Nirala J

AU - Kumar J

AU - Verma HN

DO - 10.1007/s12013-013-9715-4

LA - en

N1 - FEMU ID: 23361; EMF-Portal URL: https://www.emf-portal.org/en/article/23361

SP - 347-358

TI - Effect of 3G Cell Phone Exposure with Computer Controlled 2-D Stepper Motor on Non-thermal Activation of the hsp27/p38MAPK Stress Pathway in Rat Brain

ER -

TY - JOUR

IS - 2

JA - Electromagn Biol Med

JO - Electromagnetic Biology and Medicine

PY - 2014

SN - 1536-8386

VL - 33

AU - Souza LCM

AU - Cerqueira Ede M

AU - Meireles JR

DO - 10.3109/15368378.2013.783856

LA - en

N1 - FEMU ID: 22645; EMF-Portal URL: https://www.emf-portal.org/en/article/22645

SP - 98-102

TI - Assessment of nuclear abnormalities in exfoliated cells from the oral epithelium of mobile phone users

ER -

TY - JOUR

IS - 11

JO - Zahedan Journal of Research in Medical Sciences

PY - 2013

SN - 2383-2894

VL - 15

AU - Zahedifar Z

AU - Baharara J

LA - en

N1 - FEMU ID: 39150; EMF-Portal URL: https://www.emf-portal.org/en/article/39150

SP - 39-44

TI - Effect of Green Tea Extract in Reducing Genotoxic Injuries of Cell Phone Microwaves on Bone Marrow

UR - https://admin.kowsarpub.com/cdn/dl/28fbf5fa-6ccf-11e9-b7d0-8329d5be8d6a

ER -

TY - JOUR

IS - 2

JA - Mutat Res Genet Toxicol Environ Mutagen

JO - Mutation Research - Genetic Toxicology and Environmental Mutagenesis

PY - 2013

VL - 755

AU - Speit G

AU - Gminski R

AU - Tauber R

DO - 10.1016/j.mrgentox.2013.06.014

LA - en

N1 - FEMU ID: 22960; EMF-Portal URL: https://www.emf-portal.org/en/article/22960

SP - 163-166

TI - Genotoxic effects of exposure to radiofrequency electromagnetic fields (RF-EMF) in HL-60 cells are not reproducible

ER -

TY - JOUR

IS - 7

JO - Bioelectromagnetics

PY - 2013

SN - 0197-8462

VL - 34

AU - Vijayalaxmi

AU - Reddy AB

AU - McKenzie RJ

AU - McIntosh RL

AU - Prihoda TJ

AU - Wood AW

DO - 10.1002/bem.21798

LA - en

N1 - FEMU ID: 22648; EMF-Portal URL: https://www.emf-portal.org/en/article/22648

SP - 542-548

TI - Incidence of micronuclei in human peripheral blood lymphocytes exposed to modulated and unmodulated 2450 MHz radiofrequency fields

ER -

TY - JOUR

IS - 11

JA - Int J Radiat Biol

JO - International Journal of Radiation Biology

PY - 2013

SN - 0955-3002

VL - 89

AU - Atli Sekeroglu Z

AU - Akar A

AU - Sekeroglu V

DO - 10.3109/09553002.2013.809170

LA - en

N1 - FEMU ID: 22646; EMF-Portal URL: https://www.emf-portal.org/en/article/22646

SP - 985-992

TI - Evaluation of the cytogenotoxic damage in immature and mature rats exposed to 900 MHz radiofrequency electromagnetic fields

ER -

TY - JOUR

IS - 10

JA - Int J Radiat Biol

JO - International Journal of Radiation Biology

PY - 2013

SN - 0955-3002

VL - 89

AU - Szerencsi A

AU - Kubinyi G

AU - Valiczko E

AU - Juhasz P

AU - Rudas G

AU - Mester A

AU - Janossy G

AU - Bakos J

AU - Thuroczy G

DO - 10.3109/09553002.2013.804962

LA - en

N1 - FEMU ID: 22464; EMF-Portal URL: https://www.emf-portal.org/en/article/22464

SP - 870-876

TI - DNA Integrity of Human Leukocytes after Magnetic Resonance Imaging

ER -

TY - JOUR

IS - 2

JA - Radiat Res

JO - Radiation Research

PY - 2013

SN - 0033-7587

VL - 179

AU - Waldmann P

AU - Bohnenberger S

AU - Greinert R

AU - Hermann-Then B

AU - Heselich A

AU - Klug SJ

AU - Koenig J

AU - Kuhr K

AU - Kuster N

AU - Merker M

AU - Murbach M

AU - Pollet D

AU - Schadenboeck W

AU - Scheidemann-Wesp U

AU - Schwab B

AU - Volkmer B

AU - Weyer V

AU - Blettner M

DO - 10.1667/RR2914.1

LA - en

N1 - FEMU ID: 21641; EMF-Portal URL: https://www.emf-portal.org/en/article/21641

SP - 243-253

TI - Influence of GSM Signals on Human Peripheral Lymphocytes: Study of Genotoxicity

ER -

TY - JOUR

IS - 2

JA - Mutat Res Genet Toxicol Environ Mutagen

JO - Mutation Research - Genetic Toxicology and Environmental Mutagenesis

PY - 2013

VL - 751

AU - Jiang B

AU - Zong C

AU - Zhao H

AU - Ji Y

AU - Tong J

AU - Cao Y

DO - 10.1016/j.mrgentox.2012.12.003

LA - en

N1 - FEMU ID: 21616; EMF-Portal URL: https://www.emf-portal.org/en/article/21616

SP - 127-129

TI - Induction of adaptive response in mice exposed to 900MHz radiofrequency fields: application of micronucleus assay

ER -

TY - JOUR

IS - 3

JA - Int J Radiat Biol

JO - International Journal of Radiation Biology

PY - 2013

SN - 0955-3002

VL - 89

AU - Kumar S

AU - Behari J

AU - Sisodia R

DO - 10.3109/09553002.2013.741282

LA - en

N1 - FEMU ID: 21367; EMF-Portal URL: https://www.emf-portal.org/en/article/21367

SP - 147-154

TI - Influence of electromagnetic fields on reproductive system of male rats

ER -

TY - JOUR

IS - 1-2

JA - Mutat Res Genet Toxicol Environ Mutagen

JO - Mutation Research - Genetic Toxicology and Environmental Mutagenesis

PY - 2013

VL - 750

AU - Pesnya DS

AU - Romanovsky AV

DO - 10.1016/j.mrgentox.2012.08.010

LA - en

N1 - FEMU ID: 21320; EMF-Portal URL: https://www.emf-portal.org/en/article/21320

SP - 27-33

TI - Comparison of cytotoxic and genotoxic effects of plutonium-239 alpha particles and mobile phone GSM 900 radiation in the Allium cepa test

ER -

TY - JOUR

IS - 2

JA - J Appl Pharm Sci

JO - Journal of Applied Pharmaceutical Science

PY - 2012

SN - 2231-3354

VL - 2

AU - El-Abd SF

AU - Eltoweissy MY

LA - en

N1 - FEMU ID: 47219; EMF-Portal URL: https://www.emf-portal.org/en/article/47219

SP - 16-20

TI - Cytogenetic alterations in human lymphocyte culture following exposure to radiofrequency field of mobile phone

UR - https://www.japsonline.com/admin/php/uploads/372_pdf.pdf

ER -

TY - JOUR

IS - 9

JO - PLoS One

PY - 2012

SN - 1932-6203

VL - 7

AU - Hintzsche H

AU - Jastrow C

AU - Kleine-Ostmann T

AU - Karst U

AU - Schrader T

AU - Stopper H

DO - 10.1371/journal.pone.0046397

LA - en

N1 - FEMU ID: 21295; EMF-Portal URL: https://www.emf-portal.org/en/article/21295

SP - e46397

TI - Terahertz electromagnetic fields (0.106 THz) do not induce manifest genomic damage in vitro

UR - https://www.ncbi.nlm.nih.gov/pmc/articles/PMC3459899/pdf/pone.0046397.pdf

ER -

TY - JOUR

IS - 8

JA - Oral Dis

JO - Oral Diseases

PY - 2012

SN - 1354-523X

VL - 18

AU - Ros-Llor I

AU - Sanchez-Siles M

AU - Camacho-Alonso F

AU - Lopez-Jornet P

DO - 10.1111/j.1601-0825.2012.01946.x

LA - en

N1 - FEMU ID: 20877; EMF-Portal URL: https://www.emf-portal.org/en/article/20877

SP - 786-792

TI - Effect of mobile phones on micronucleus frequency in human exfoliated oral mucosal cells

ER -

TY - JOUR

IS - 7

JO - Bioelectromagnetics

PY - 2012

SN - 0197-8462

VL - 33

AU - Hong MN

AU - Kim BC

AU - Ko YG

AU - Lee YS

AU - Hong SC

AU - Kim T

AU - Pack JK

AU - Choi HD

AU - Kim N

AU - Lee JS

DO - 10.1002/bem.21731

LA - en

N1 - FEMU ID: 20693; EMF-Portal URL: https://www.emf-portal.org/en/article/20693

SP - 604-611

TI - Effects of 837 and 1950 MHz radiofrequency radiation exposure alone or combined on oxidative stress in MCF10A cells

ER -

TY - JOUR

IS - 1

JA - Mutat Res Genet Toxicol Environ Mutagen

JO - Mutation Research - Genetic Toxicology and Environmental Mutagenesis

PY - 2012

VL - 747

AU - Zeni O

AU - Sannino A

AU - Romeo S

AU - Massa R

AU - Sarti M

AU - Reddy AB

AU - Prihoda TJ

AU - Vijayalaxmi

AU - Scarfi MR

DO - 10.1016/j.mrgentox.2012.03.013

LA - en

N1 - FEMU ID: 20662; EMF-Portal URL: https://www.emf-portal.org/en/article/20662

SP - 29-35

TI - Induction of an adaptive response in human blood lymphocytes exposed to radiofrequency fields: Influence of the universal mobile telecommunication system (UMTS) signal and the specific absorption rate

ER -

TY - JOUR

IS - 2

JA - J Radiat Res

JO - Journal of Radiation Research

PY - 2012

SN - 0449-3060

VL - 53

AU - Kim HN

AU - Han NK

AU - Hong MN

AU - Chi SG

AU - Lee YS

AU - Kim T

AU - Pack JK

AU - Choi HD

AU - Kim N

AU - Lee JS

DO - 10.1269/jrr.11048

LA - en

N1 - FEMU ID: 20543; EMF-Portal URL: https://www.emf-portal.org/en/article/20543

SP - 176-183

TI - Analysis of the cellular stress response in MCF10A cells exposed to combined radio frequency radiation

UR - https://www.jstage.jst.go.jp/article/jrr/53/2/53_11048/_pdf

ER -

TY - JOUR

IS - 14

JA - J Agric Food Chem

JO - Journal of Agricultural and Food Chemistry

PY - 2012

SN - 0021-8561

VL - 60

AU - Pande R

AU - Mishra HN

AU - Singh MN

DO - 10.1021/jf204540n

LA - en

N1 - FEMU ID: 20386; EMF-Portal URL: https://www.emf-portal.org/en/article/20386

SP - 3809-3816

TI - Microwave drying for safe storage and improved nutritional quality of green gram seed (Vigna radiata)

ER -

TY - JOUR

IS - 4

JO - Mutagenesis

PY - 2012

SN - 0267-8357

VL - 27

AU - Hintzsche H

AU - Jastrow C

AU - Kleine-Ostmann T

AU - Schrader T

AU - Stopper H

DO - 10.1093/mutage/ges007

LA - en

N1 - FEMU ID: 20370; EMF-Portal URL: https://www.emf-portal.org/en/article/20370

SP - 477-483

TI - 900 MHz radiation does not induce micronucleus formation in different cell types

UR - https://academic.oup.com/mutage/article-pdf/27/4/477/3889278/ges007.pdf

ER -

TY - JOUR

JA - Ecotoxicol Environ Saf

JO - Ecotoxicology and Environmental Safety

PY - 2012

SN - 0147-6513

VL - 80

AU - Sekeroglu V

AU - Akar A

AU - Sekeroglu ZA

DO - 10.1016/j.ecoenv.2012.02.028

LA - en

N1 - FEMU ID: 20362; EMF-Portal URL: https://www.emf-portal.org/en/article/20362

SP - 140-144

TI - Cytotoxic and genotoxic effects of high-frequency electromagnetic fields (GSM 1800MHz) on immature and mature rats

ER -

TY - JOUR

IS - 1

JA - J Neurooncol

JO - Journal of Neuro-Oncology

PY - 2012

SN - 0167-594X

VL - 106

AU - Karaca E

AU - Durmaz B

AU - Aktug H

AU - Yildiz T

AU - Guducu C

AU - Irgi M

AU - Koksal MG

AU - Ozkinay F

AU - Gunduz C

AU - Cogulu O

DO - 10.1007/s11060-011-0644-z

LA - en

N1 - FEMU ID: 19410; EMF-Portal URL: https://www.emf-portal.org/en/article/19410

SP - 53-58

TI - The genotoxic effect of radiofrequency waves on mouse brain

ER -

TY - JOUR

IS - 4

JA - Electromagn Biol Med

JO - Electromagnetic Biology and Medicine

PY - 2011

SN - 1536-8386

VL - 30

AU - Zeng L

AU - Ji X

AU - Zhang Y

AU - Miao X

AU - Zou C

AU - Lang H

AU - Zhang J

AU - Li Y

AU - Wang X

AU - Qi H

AU - Ren D

AU - Guo G

DO - 10.3109/15368378.2011.587929

LA - en

N1 - FEMU ID: 19856; EMF-Portal URL: https://www.emf-portal.org/en/article/19856

SP - 205-218

TI - MnSOD expression inhibited by electromagnetic pulse radiation in the rat testis

ER -

TY - JOUR

IS - 9

JA - Int J Radiat Biol

JO - International Journal of Radiation Biology

PY - 2011

SN - 0955-3002

VL - 87

AU - Sannino A

AU - Zeni O

AU - Sarti M

AU - Romeo S

AU - Reddy SB

AU - Belisario MA

AU - Prihoda TJ

AU - Vijayalaxmi

AU - Scarfi MR

DO - 10.3109/09553002.2011.574779

LA - en

N1 - FEMU ID: 19265; EMF-Portal URL: https://www.emf-portal.org/en/article/19265

SP - 993-999

TI - Induction of adaptive response in human blood lymphocytes exposed to 900 MHz radiofrequency fields: Influence of cell cycle

ER -

TY - JOUR

IS - 4

JA - Appl Biochem Biotechnol

JO - Applied Biochemistry and Biotechnology

PY - 2011

SN - 0273-2289

VL - 164

AU - Kesari KK

AU - Kumar S

AU - Behari J

DO - 10.1007/s12010-010-9156-0

LA - en

N1 - FEMU ID: 18931; EMF-Portal URL: https://www.emf-portal.org/en/article/18931

SP - 546-559

TI - Effects of radiofrequency electromagnetic wave exposure from cellular phones on the reproductive pattern in male wistar rats

ER -

TY - JOUR

IS - 1

JA - Int J Hyg Environ Health

JO - International Journal of Hygiene and Environmental Health

PY - 2011

SN - 1438-4639

VL - 214

AU - Garaj-Vrhovac V

AU - Gajski G

AU - Pazanin S

AU - Sarolic A

AU - Domijan AM

AU - Flajs D

AU - Peraica M

DO - 10.1016/j.ijheh.2010.08.003

LA - en

N1 - FEMU ID: 18617; EMF-Portal URL: https://www.emf-portal.org/en/article/18617

SP - 59-65

TI - Assessment of cytogenetic damage and oxidative stress in personnel occupationally exposed to the pulsed microwave radiation of marine radar equipment

ER -

TY - JOUR

IS - 6

JA - Indian J Exp Biol

JO - Indian Journal of Experimental Biology

PY - 2010

SN - 0019-5189

VL - 48

AU - Kumar S

AU - Kesari KK

AU - Behari J

LA - en

N1 - FEMU ID: 18567; EMF-Portal URL: https://www.emf-portal.org/en/article/18567

SP - 586-592

TI - Evaluation of genotoxic effects in male Wistar rats following microwave exposure

UR - http://nopr.niscpr.res.in/bitstream/123456789/9081/1/IJEB%2048%286%29%20586-592.pdf

ER -

TY - JOUR

IS - 3

JA - Electromagn Biol Med

JO - Electromagnetic Biology and Medicine

PY - 2010

SN - 1536-8386

VL - 29

AU - Gurbuz N

AU - Sirav B

AU - Yuvaci HU

AU - Turhan N

AU - Coskun ZK

AU - Seyhan N

DO - 10.3109/15368378.2010.482498

LA - en

N1 - FEMU ID: 18549; EMF-Portal URL: https://www.emf-portal.org/en/article/18549

SP - 98-104

TI - Is There Any Possible Genotoxic Effect in Exfoliated Bladder Cells of Rat Under the Exposure of 1800 MHz GSM-Like Modulated Radio Frequency Radiation (RFR)?

ER -

TY - JOUR

IS - 2

JA - Genet Couns

JO - Genetic Counseling

PY - 2010

SN - 1015-8146

VL - 21

AU - Yildirim MS

AU - Yildirim A

AU - Zamani AG

AU - Okudan N

LA - en

N1 - FEMU ID: 18526; EMF-Portal URL: https://www.emf-portal.org/en/article/18526

SP - 243-251

TI - Effect of mobile phone station on micronucleus frequency and chromosomal aberrations in human blood cells

ER -

TY - JOUR

IS - 1

JA - Toxicol Lett

JO - Toxicology Letters

PY - 2010

SN - 0378-4274

VL - 193

AU - Hintzsche H

AU - Stopper H

DO - 10.1016/j.toxlet.2009.12.016

LA - en

N1 - FEMU ID: 17832; EMF-Portal URL: https://www.emf-portal.org/en/article/17832

SP - 124-130

TI - Micronucleus frequency in buccal mucosa cells of mobile phone users

ER -

TY - JOUR

IS - 6

JA - Radiat Res

JO - Radiation Research

PY - 2009

SN - 0033-7587

VL - 171

AU - Sannino A

AU - Di Costanzo G

AU - Brescia F

AU - Sarti M

AU - Zeni O

AU - Juutilainen J

AU - Scarfi MR

DO - 10.1667/RR1642.1

LA - en

N1 - FEMU ID: 17175; EMF-Portal URL: https://www.emf-portal.org/en/article/17175

SP - 743-751

TI - Human fibroblasts and 900 MHz radiofrequency radiation: evaluation of DNA damage after exposure and co-exposure to 3-chloro-4-(dichloromethyl)-5-hydroxy-2(5h)-furanone (MX)

ER -

TY - JOUR

IS - 6

JA - Radiat Res

JO - Radiation Research

PY - 2009

SN - 0033-7587

VL - 171

AU - Sannino A

AU - Sarti M

AU - Reddy SB

AU - Prihoda TJ

AU - Vijayalaxmi

AU - Scarfi MR

DO - 10.1667/RR1687.1

LA - en

N1 - FEMU ID: 17174; EMF-Portal URL: https://www.emf-portal.org/en/article/17174

SP - 735-742

TI - Induction of adaptive response in human blood lymphocytes exposed to radiofrequency radiation

ER -

TY - JOUR

IS - 5

JA - Int J Radiat Biol

JO - International Journal of Radiation Biology

PY - 2009

SN - 0955-3002

VL - 85

AU - Ziemann C

AU - Brockmeyer H

AU - Reddy SB

AU - Vijayalaxmi

AU - Prihoda TJ

AU - Kuster N

AU - Tillmann T

AU - Dasenbrock C

DO - 10.1080/09553000902818907

LA - en

N1 - FEMU ID: 17010; EMF-Portal URL: https://www.emf-portal.org/en/article/17010

SP - 454-464

TI - Absence of genotoxic potential of 902 MHz (GSM) and 1747 MHz (DCS) wireless communication signals: In vivo two-year bioassay in B6C3F1 mice

ER -

TY - GEN

ET - 1

PB - IEEE

PY - 2008

SN - 9781424428670

T2 - 2008 IEEE International RF and Microwave Conference, Kuala Lumpur, Malaysia

AU - Rusnani A

AU - Norhayati MN

AU - Siti Noraini S

AU - Marina M

DO - 10.1109/RFM.2008.4897448

LA - en

N1 - FEMU ID: 49987; EMF-Portal URL: https://www.emf-portal.org/en/article/49987

SP - 262-267

TI - Microwave radiation effect - a test on white mice

ER -

TY - JOUR

IS - 6

JA - Int Arch Occup Environ Health

JO - International Archives of Occupational and Environmental Health

PY - 2008

SN - 0340-0131

VL - 81

AU - Schwarz C

AU - Kratochvil E

AU - Pilger A

AU - Kuster N

AU - Adlkofer F

AU - Rudiger HW

DO - 10.1007/s00420-008-0305-5

LA - en

N1 - FEMU ID: 15682; EMF-Portal URL: https://www.emf-portal.org/en/article/15682

SP - 755-767

TI - Radiofrequency electromagnetic fields (UMTS, 1,950 MHz) induce genotoxic effects in vitro in human fibroblasts but not in lymphocytes

ER -

TY - JOUR

IS - 2

JA - Mutat Res Genet Toxicol Environ Mutagen

JO - Mutation Research - Genetic Toxicology and Environmental Mutagenesis

PY - 2008

VL - 650

AU - Yadav AS

AU - Sharma MK

DO - 10.1016/j.mrgentox.2007.11.005

LA - en

N1 - FEMU ID: 15588; EMF-Portal URL: https://www.emf-portal.org/en/article/15588

SP - 175-180

TI - Increased frequency of micronucleated exfoliated cells among humans exposed in vivo to mobile telephone radiations

ER -

TY - JOUR

IS - 3

JO - Bioelectromagnetics

PY - 2008

SN - 0197-8462

VL - 29

AU - Zeni O

AU - Schiavoni A

AU - Perrotta A

AU - Forigo D

AU - Deplano M

AU - Scarfi MR

DO - 10.1002/bem.20378

LA - en

N1 - FEMU ID: 15373; EMF-Portal URL: https://www.emf-portal.org/en/article/15373

SP - 177-184

TI - Evaluation of genotoxic effects in human leukocytes after in vitro exposure to 1950 MHz UMTS radiofrequency field

ER -

TY - GEN

ET - 1

PB - IEEE

PY - 2007

SN - 9781424413720

T2 - 2007 International Symposium on Electromagnetic Compatibility

AU - Ren DQ

AU - Yang WQ

AU - Zhao T

AU - Li XJ

AU - Zeng GY

AU - Zhang J

AU - Li YR

DO - 10.1109/ELMAGC.2007.4413535

LA - en

N1 - FEMU ID: 49696; EMF-Portal URL: https://www.emf-portal.org/en/article/49696

SP - 479-481

TI - Changes of microwave radiation on the level of trace elements in mice bone tissues

ER -

TY - JOUR

IS - 4

JA - Int J Radiat Biol

JO - International Journal of Radiation Biology

PY - 2007

SN - 0955-3002

VL - 83

AU - Juutilainen J

AU - Heikkinen P

AU - Soikkeli H

AU - Maki-Paakkanen J

DO - 10.1080/09553000601169800

LA - en

N1 - FEMU ID: 14790; EMF-Portal URL: https://www.emf-portal.org/en/article/14790

SP - 213-220

TI - Micronucleus frequency in erythrocytes of mice after long-term exposure to radiofrequency radiation

ER -

TY - JOUR

IS - 4

JA - Health Phys

JO - Health Physics

PY - 2007

SN - 0017-9078

VL - 92

AU - Zeni O

AU - Gallerano GP

AU - Perrotta A

AU - Romano M

AU - Sannino A

AU - Sarti M

AU - D'Arienzo M

AU - Doria A

AU - Giovenale E

AU - Lai A

AU - Messina G

AU - Scarfi MR

DO - 10.1097/01.HP.0000251248.23991.35

LA - en

N1 - FEMU ID: 14603; EMF-Portal URL: https://www.emf-portal.org/en/article/14603

SP - 349-357

TI - Cytogenetic Observations In Human Peripheral Blood Leukocytes Following In Vitro Exposure To THz Radiation: A Pilot Study

ER -

TY - JOUR

IS - 1-2

JA - Mutat Res Genet Toxicol Environ Mutagen

JO - Mutation Research - Genetic Toxicology and Environmental Mutagenesis

PY - 2007

VL - 626

AU - Speit G

AU - Schütz P

AU - Hoffmann H

DO - 10.1016/j.mrgentox.2006.08.003

LA - en

N1 - FEMU ID: 14202; EMF-Portal URL: https://www.emf-portal.org/en/article/14202

SP - 42-47

TI - Genotoxic effects of exposure to radiofrequency electromagnetic fields (RF-EMF) in cultured mammalian cells are not independently reproducible

ER -

TY - JOUR

IS - 6

JA - IEEE Trans Microw Theory Tech

JO - IEEE Transactions on Microwave Theory and Techniques

PY - 2006

SN - 0018-9480

VL - 54

AU - Zhadobov M

AU - Sauleau R

AU - Vie V

AU - Himd M

AU - Le Coq L

AU - Thouroude D

DO - 10.1109/TMTT.2006.875811

LA - en

N1 - FEMU ID: 16235; EMF-Portal URL: https://www.emf-portal.org/en/article/16235

SP - 2534-2542

TI - Interactions between 60-GHz millimeter waves and artificial biological membranes: dependence on radiation parameters

ER -

TY - JOUR

IS - 3

JA - Exp Toxicol Pathol

JO - Experimental and Toxicologic Pathology

PY - 2006

SN - 0940-2993

VL - 57

AU - Trosic I

AU - Busljeta I

DO - 10.1016/j.etp.2005.08.002

LA - en

N1 - FEMU ID: 15461; EMF-Portal URL: https://www.emf-portal.org/en/article/15461

SP - 247-251

TI - Erythropoietic dynamic equilibrium in rats maintained after microwave irradiation

ER -

TY - JOUR

IS - 1

JA - Life Sci

JO - Life Sciences

PY - 2006

SN - 0024-3205

VL - 80

AU - Ferreira AR

AU - Knakievicz T

AU - Pasquali MA

AU - Gelain DP

AU - Dal-Pizzol F

AU - Fernandez CE

AU - de Salles AA

AU - Ferreira HB

AU - Moreira JC

DO - 10.1016/j.lfs.2006.08.018

LA - en

N1 - FEMU ID: 14184; EMF-Portal URL: https://www.emf-portal.org/en/article/14184

SP - 43-50

TI - Ultra high frequency-electromagnetic field irradiation during pregnancy leads to an increase in erythrocytes micronuclei incidence in rat offspring

ER -

TY - JOUR

IS - 3

JA - Radiat Res

JO - Radiation Research

PY - 2006

SN - 0033-7587

VL - 166

AU - Vijayalaxmi

DO - 10.1667/RR0643.1

LA - en

N1 - FEMU ID: 14138; EMF-Portal URL: https://www.emf-portal.org/en/article/14138

SP - 532-538

TI - Cytogenetic studies in human blood lymphocytes exposed in vitro to 2.45 GHz or 8.2 GHz radiofrequency radiation

ER -

TY - JOUR

IS - 5

JA - Int J Radiat Biol

JO - International Journal of Radiation Biology

PY - 2006

SN - 0955-3002

VL - 82

AU - Stronati L

AU - Testa A

AU - Moquet J

AU - Edwards A

AU - Cordelli E

AU - Villani P

AU - Marino C

AU - Fresegna AM

AU - Appolloni M

AU - Lloyd D

DO - 10.1080/09553000600739173

LA - en

N1 - FEMU ID: 13927; EMF-Portal URL: https://www.emf-portal.org/en/article/13927

SP - 339-346

TI - 935 MHz cellular phone radiation. An in vitro study of genotoxicity in human lymphocytes

ER -

TY - JOUR

IS - 6

JA - Radiat Res

JO - Radiation Research

PY - 2006

SN - 0033-7587

VL - 165

AU - Scarfi MR

AU - Fresegna AM

AU - Villani P

AU - Pinto R

AU - Marino C

AU - Sarti M

AU - Altavista P

AU - Sannino A

AU - Lovisolo GA

DO - 10.1667/RR3570.1

LA - en

N1 - FEMU ID: 13901; EMF-Portal URL: https://www.emf-portal.org/en/article/13901

SP - 655-663

TI - Exposure to radiofrequency radiation (900 MHz, GSM signal) does not affect micronucleus frequency and cell proliferation in human peripheral blood lymphocytes: an interlaboratory study

ER -

TY - JOUR

IS - 5

JA - Radiat Res

JO - Radiation Research

PY - 2006

SN - 0033-7587

VL - 165

AU - Verschaeve L

AU - Heikkinen P

AU - Verheyen G

AU - Van Gorp U

AU - Boonen F

AU - Vander Plaetse F

AU - Maes A

AU - Kumlin T

AU - Maki-Paakkanen J

AU - Puranen L

AU - Juutilainen J

DO - 10.1667/RR3559.1

LA - en

N1 - FEMU ID: 13792; EMF-Portal URL: https://www.emf-portal.org/en/article/13792

SP - 598-607

TI - Investigation of co-genotoxic effects of radiofrequency electromagnetic fields in vivo

ER -

TY - JOUR

JA - Phys Scr

JO - Physica Scripta

PY - 2005

SN - 0031-8949

VL - 118

AU - Trosic I

AU - Busljeta I

DO - 10.1238/Physica.Topical.118a00168

LA - en

N1 - FEMU ID: 19665; EMF-Portal URL: https://www.emf-portal.org/en/article/19665

SP - 168-170

TI - Frequency of micronucleated erythrocytes in rat bone marrow exposed to 2.45 GHz radiation

ER -

TY - JOUR

IS - 4

JA - Int J Hum Genet

JO - International Journal of Human Genetics

PY - 2005

SN - 0972-3757

VL - 5

AU - Gandhi G

AU - Singh P

LA - en

N1 - FEMU ID: 16802; EMF-Portal URL: https://www.emf-portal.org/en/article/16802

SP - 259-265

TI - Cytogenetic damage in mobile phone users: preliminary data

UR - http://www.krepublishers.com/02-Journals/IJHG/IJHG-05-0-000-000-2005-Web/IJHG-05-4-225-288-2005-Abst-PDF/IJHG-05-4-259-265-2005-210-Gandhi-G/IJHG-05-4-259-265-2005-210-Gandhi-G.pdf

ER -

TY - JOUR

IS - 2

JA - Indian J Hum Genet

JO - Indian Journal of Human Genetics

PY - 2005

SN - 1998-362X

VL - 11

AU - Gandhi G

AU - Anita

DO - 10.4103/0971-6866.16810

LA - en

N1 - FEMU ID: 16801; EMF-Portal URL: https://www.emf-portal.org/en/article/16801

SP - 99-104

TI - Genetic damage in mobile phone users: some preliminary findings

UR - http://www.bioline.org.br/pdf?hg05022

ER -

TY - JOUR

IS - 3

JA - Electromagn Biol Med

JO - Electromagnetic Biology and Medicine

PY - 2005

SN - 1536-8386

VL - 24

AU - Vojtisek M

AU - Knotkova J

AU - Kasparova L

AU - Hornychova M

AU - Frantik M

AU - Svandova E

DO - 10.1080/15368370500381695

LA - en

N1 - FEMU ID: 13487; EMF-Portal URL: https://www.emf-portal.org/en/article/13487

SP - 355-358

TI - Potential Impact of Simulated Mobile Phone Radiation on Blood-Brain Barrier

ER -

TY - JOUR

IS - 1-2

JA - Mutat Res Genet Toxicol Environ Mutagen

JO - Mutation Research - Genetic Toxicology and Environmental Mutagenesis

PY - 2005

VL - 587

AU - Komatsubara Y

AU - Hirose H

AU - Sakurai T

AU - Koyama S

AU - Suzuki Y

AU - Taki M

AU - Miyakoshi J

DO - 10.1016/j.mrgentox.2005.08.010

LA - en

N1 - FEMU ID: 12641; EMF-Portal URL: https://www.emf-portal.org/en/article/12641

SP - 114-119

TI - Effect of high-frequency electromagnetic fields with a wide range of SARs on chromosomal aberrations in murine m5S cells

ER -

TY - JOUR

IS - 4

JA - Radiat Res

JO - Radiation Research

PY - 2005

SN - 0033-7587

VL - 164

AU - Gorlitz BD

AU - Muller M

AU - Ebert S

AU - Hecker H

AU - Kuster N

AU - Dasenbrock C

DO - 10.1667/rr3440.1

LA - en

N1 - FEMU ID: 12605; EMF-Portal URL: https://www.emf-portal.org/en/article/12605

SP - 431-439

TI - Effects of 1-week and 6-week exposure to GSM/DCS radiofrequency radiation on micronucleus formation in B6C3F1 mice

ER -

TY - JOUR

IS - 1-2

JA - Mutat Res Genet Toxicol Environ Mutagen

JO - Mutation Research - Genetic Toxicology and Environmental Mutagenesis

PY - 2005

VL - 582

AU - Zotti-Martelli L

AU - Peccatori M

AU - Maggini V

AU - Ballardin M

AU - Barale R

DO - 10.1016/j.mrgentox.2004.12.014

LA - en

N1 - FEMU ID: 12152; EMF-Portal URL: https://www.emf-portal.org/en/article/12152

SP - 42-52

TI - Individual responsiveness to induction of micronuclei in human lymphocytes after exposure in vitro to 1800 MHz microwave radiation

ER -

TY - JOUR

IS - 6

JA - Int J Hyg Environ Health

JO - International Journal of Hygiene and Environmental Health

PY - 2004

SN - 1438-4639

VL - 207

AU - Busljeta I

AU - Trosic I

AU - Milkovic-Kraus S

DO - 10.1078/1438-4639-00326

LA - en

N1 - FEMU ID: 11630; EMF-Portal URL: https://www.emf-portal.org/en/article/11630

SP - 549-554

TI - Erythropoietic changes in rats after 2.45 GHz nonthermal irradiation

ER -

TY - JOUR

IS - 5

JO - Mutagenesis

PY - 2004

SN - 0267-8357

VL - 19

AU - Trosic I

AU - Busljeta I

AU - Modlic B

DO - 10.1093/mutage/geh042

LA - en

N1 - FEMU ID: 11628; EMF-Portal URL: https://www.emf-portal.org/en/article/11628

SP - 361-364

TI - Investigation of the genotoxic effect of microwave irradiation in rat bone marrow cells: in vivo exposure

UR - https://academic.oup.com/mutage/article-pdf/19/5/361/4067612/geh042.pdf

ER -

TY - JOUR

JA - ScientificWorldJournal

JO - The Scientific World Journal

PY - 2004

SN - 1537-744X

VL - 4

AU - Demsia G

AU - Vlastos D

AU - Matthopoulos DP

DO - 10.1100/tsw.2004.178

LA - en

N1 - FEMU ID: 11395; EMF-Portal URL: https://www.emf-portal.org/en/article/11395

SP - 48-54

TI - Effect of 910-MHz electromagnetic field on rat bone marrow

UR - https://www.hindawi.com/journals/tswj/2004/591712/abs/

ER -

TY - JOUR

JA - ScientificWorldJournal

JO - The Scientific World Journal

PY - 2004

SN - 1537-744X

VL - 4

AU - Koyama S

AU - Isozumi Y

AU - Suzuki Y

AU - Taki M

AU - Miyakoshi J

DO - 10.1100/tsw.2004.176

LA - en

N1 - FEMU ID: 11393; EMF-Portal URL: https://www.emf-portal.org/en/article/11393

SP - 29-40

TI - Effects of 2.45-GHz electromagnetic fields with a wide range of SARs on micronucleus formation in CHO-K1 cells

UR - http://downloads.hindawi.com/journals/tswj/2004/743762.pdf

ER -

TY - JOUR

IS - 3

JA - Radiat Res

JO - Radiation Research

PY - 2004

SN - 0033-7587

VL - 161

AU - Vijayalaxmi

AU - Logani MK

AU - Bhanushali A

AU - Ziskin MC

AU - Prihoda TJ

DO - 10.1667/rr3121

LA - en

N1 - FEMU ID: 10560; EMF-Portal URL: https://www.emf-portal.org/en/article/10560

SP - 341-345

TI - Micronuclei in peripheral blood and bone marrow cells of mice exposed to 42 GHz electromagnetic millimeter waves

ER -

TY - JOUR

IS - 2-3

JA - J Biol Phys

JO - Journal of Biological Physics

PY - 2003

SN - 0092-0606

VL - 29

AU - Scarfi MR

AU - Romano M

AU - Di Pietro R

AU - Zeni O

AU - Doria A

AU - Gallerano GP

AU - Giovenale E

AU - Messina G

AU - Lai A

AU - Campurra G

AU - Coniglio D

AU - Arienzo D

DO - 10.1023/A:1024440708943

LA - en

N1 - FEMU ID: 14927; EMF-Portal URL: https://www.emf-portal.org/en/article/14927

SP - 171-177

TI - THz Exposure of Whole Blood for the Study of Biological Effects on Human Lymphocytes

UR - https://www.ncbi.nlm.nih.gov/pmc/articles/PMC3456423/pdf/10867_2004_Article_5121580.pdf

ER -

TY - JOUR

IS - 9

JA - Int J Radiat Biol

JO - International Journal of Radiation Biology

PY - 2003

SN - 0955-3002

VL - 79

AU - Port M

AU - Abend M

AU - Romer B

AU - Van Beuningen D

DO - 10.1080/09553000310001606803

LA - en

N1 - FEMU ID: 10792; EMF-Portal URL: https://www.emf-portal.org/en/article/10792

SP - 701-708

TI - Influence of high-frequency electromagnetic fields on different modes of cell death and gene expression

ER -

TY - JOUR

IS - 1-2

JA - Mutat Res Genet Toxicol Environ Mutagen

JO - Mutation Research - Genetic Toxicology and Environmental Mutagenesis

PY - 2003

VL - 541

AU - Koyama S

AU - Nakahara T

AU - Wake K

AU - Taki M

AU - Isozumi Y

AU - Miyakoshi J

DO - 10.1016/j.mrgentox.2003.07.009

LA - en

N1 - FEMU ID: 10341; EMF-Portal URL: https://www.emf-portal.org/en/article/10341

SP - 81-89

TI - Effects of high frequency electromagnetic fields on micronucleus formation in CHO-K1 cells

ER -

TY - JOUR

IS - 2

JA - Radiat Res

JO - Radiation Research

PY - 2003

SN - 0033-7587

VL - 160

AU - Zeni O

AU - Chiavoni AS

AU - Sannino A

AU - Antolini A

AU - Forigo D

AU - Bersani F

AU - Scarfi MR

DO - 10.1667/rr3014

LA - en

N1 - FEMU ID: 10036; EMF-Portal URL: https://www.emf-portal.org/en/article/10036

SP - 152-158

TI - Lack of genotoxic effects (micronucleus induction) in human lymphocytes exposed in vitro to 900 MHz electromagnetic fields

ER -

TY - JOUR

IS - 4

JA - Radiat Res

JO - Radiation Research

PY - 2003

SN - 0033-7587

VL - 159

AU - Vijayalaxmi

AU - Sasser LB

AU - Morris JE

AU - Wilson BW

AU - Anderson LE

DO - 10.1667/0033-7587(2003)159[0558:gpogwc]2.0.co;2

LA - en

N1 - FEMU ID: 9893; EMF-Portal URL: https://www.emf-portal.org/en/article/9893

SP - 558-564

TI - Genotoxic potential of 1.6 GHz wireless communication signal: in vivo two-year bioassay

UR - https://meridian.allenpress.com/radiation-research/article-pdf/159/4/558/2194600/0033-7587(2003)159%5b0558_gpogwc%5d2_0_co_2.pdf

ER -

TY - JOUR

IS - 5

JA - Radiat Res

JO - Radiation Research

PY - 2003

SN - 0033-7587

VL - 159

AU - McNamee JP

AU - Bellier PV

AU - Gajda GB

AU - Lavallee BF

AU - Marro L

AU - Lemay E

AU - Thansandote A

DO - 10.1667/0033-7587(2003)159[0693:nefgef]2.0.co;2

LA - en

N1 - FEMU ID: 9883; EMF-Portal URL: https://www.emf-portal.org/en/article/9883

SP - 693-697

TI - No evidence for genotoxic effects from 24 h exposure of human leukocytes to 1.9 GHz radiofrequency fields

ER -

TY - JOUR

IS - 1-2

JA - Mutat Res Genet Toxicol Environ Mutagen

JO - Mutation Research - Genetic Toxicology and Environmental Mutagenesis

PY - 2002

VL - 521

AU - Trosic I

AU - Busljeta I

AU - Kasuba V

AU - Rozgaj R

DO - 10.1016/s1383-5718(02)00214-0

LA - en

N1 - FEMU ID: 11629; EMF-Portal URL: https://www.emf-portal.org/en/article/11629

SP - 73-79

TI - Micronucleus induction after whole-body microwave irradiation of rats

ER -

TY - JOUR

IS - 4

JA - Biomed Environ Sci

JO - Biomedical and Environmental Sciences

PY - 2002

SN - 0895-3988

VL - 15

AU - Zhang MB

AU - He JL

AU - Jin LF

AU - Lu DQ

LA - en

N1 - FEMU ID: 9988; EMF-Portal URL: https://www.emf-portal.org/en/article/9988

SP - 283-290

TI - Study of low-intensity 2450-MHz microwave exposure enhancing the genotoxic effects of mitomycin C using micronucleus test and comet assay in vitro

ER -

TY - JOUR

IS - 4

JA - Radiat Res

JO - Radiation Research

PY - 2002

SN - 0033-7587

VL - 158

AU - McNamee JP

AU - Bellier PV

AU - Gajda GB

AU - Miller SM

AU - Lemay EP

AU - Lavallee BF

AU - Marro L

AU - Thansandote A

DO - 10.1667/0033-7587(2002)158[0523:ddamii]2.0.co;2

LA - en

N1 - FEMU ID: 9741; EMF-Portal URL: https://www.emf-portal.org/en/article/9741

SP - 523-533

TI - DNA damage and micronucleus induction in human leukocytes after acute in vitro exposure to a 1.9 GHz continuous-wave radiofrequency field

ER -

TY - JOUR

IS - 4

JA - Radiat Res

JO - Radiation Research

PY - 2002

SN - 0033-7587

VL - 158

AU - McNamee JP

AU - Bellier PV

AU - Gajda GB

AU - Lavallee BF

AU - Lemay EP

AU - Marro L

AU - Thansandote A

DO - 10.1667/0033-7587(2002)158[0534:ddihla]2.0.co;2

LA - en

N1 - FEMU ID: 9740; EMF-Portal URL: https://www.emf-portal.org/en/article/9740

SP - 534-537

TI - DNA damage in human leukocytes after acute in vitro exposure to a 1.9 GHz pulse-modulated radiofrequency field

ER -

TY - JOUR

IS - 5

JA - Radiat Res

JO - Radiation Research

PY - 2002

SN - 0033-7587

VL - 157

AU - Bisht KS

AU - Moros EG

AU - Straube WL

AU - Baty JD

AU - Roti Roti JL

DO - 10.1667/0033-7587(2002)157[0506:teomfo]2.0.co;2

LA - en

N1 - FEMU ID: 8831; EMF-Portal URL: https://www.emf-portal.org/en/article/8831

SP - 506-515

TI - The effect of 835.62 MHz FDMA or 847.74 MHz CDMA modulated radiofrequency radiation on the induction of micronuclei in C3H 10T(1/2) cells

ER -

TY - JOUR

IS - 2

JO - Bioelectromagnetics

PY - 2002

SN - 0197-8462

VL - 23

AU - Tice RR

AU - Hook GG

AU - Donner M

AU - McRee DI

AU - Guy AW

DO - 10.1002/bem.104

LA - en

N1 - FEMU ID: 8518; EMF-Portal URL: https://www.emf-portal.org/en/article/8518

SP - 113-126

TI - Genotoxicity of radiofrequency signals. I. Investigation of DNA damage and micronuclei induction in cultured human blood cells

ER -

TY - JOUR

IS - 1

JO - Bioelectromagnetics

PY - 2002

SN - 0197-8462

VL - 23

AU - d'Ambrosio G

AU - Massa R

AU - Scarfi MR

AU - Zeni O

DO - 10.1002/bem.93

LA - en

N1 - FEMU ID: 8130; EMF-Portal URL: https://www.emf-portal.org/en/article/8130

SP - 7-13

TI - Cytogenetic damage in human lymphocytes following GMSK phase modulated microwave exposure

ER -

TY - GEN

ET - 1

PB - Springer

PP - Boston

PY - 2001

SN - 9780306469015

T2 - Wireless Phones and Health II: State of the Science

AU - Hook GJ

DO - 10.1007/0-306-46901-4_11

LA - en

N1 - FEMU ID: 49553; EMF-Portal URL: https://www.emf-portal.org/en/article/49553

SP - 143-146

TI - Evaluation of potential genotoxicity of radiofrequency radiation technologies using the single cell gel electrophoresis and micronucleus assays

ER -

TY - JOUR

IS - 4

JA - Radiat Res

JO - Radiation Research

PY - 2001

SN - 0033-7587

VL - 156

AU - Vijayalaxmi

AU - Bisht KS

AU - Pickard WF

AU - Meltz ML

AU - Roti Roti JL

AU - Moros EG

DO - 10.1667/0033-7587(2001)156[0430:cdamfi]2.0.co;2

LA - en

N1 - FEMU ID: 7827; EMF-Portal URL: https://www.emf-portal.org/en/article/7827

SP - 430-433

TI - Chromosome damage and micronucleus formation in human blood lymphocytes exposed in vitro to radiofrequency radiation at a cellular telephone frequency (847.74 MHz, CDMA)

ER -

TY - JOUR

IS - 11

JA - Int J Radiat Biol

JO - International Journal of Radiation Biology

PY - 2001

SN - 0955-3002

VL - 77

AU - Vijayalaxmi

AU - Pickard WF

AU - Bisht KS

AU - Prihoda TJ

AU - Meltz ML

AU - LaRegina MC

AU - Roti Roti JL

AU - Straube WL

AU - Moros EG

DO - 10.1080/09553000110069100

LA - en

N1 - FEMU ID: 7826; EMF-Portal URL: https://www.emf-portal.org/en/article/7826

SP - 1109-1115

TI - Micronuclei in the peripheral blood and bone marrow cells of rats exposed to 2450 MHz radiofrequency radiation

ER -

TY - JOUR

IS - 1

JA - Radiat Res

JO - Radiation Research

PY - 2001

SN - 0033-7587

VL - 155

AU - Vijayalaxmi

AU - Leal BZ

AU - Meltz ML

AU - Pickard WF

AU - Bisht KS

AU - Roti Roti JL

AU - Straube WL

AU - Moros EG

DO - 10.1667/0033-7587(2001)155[0113:csihbl]2.0.co;2

LA - en

N1 - FEMU ID: 5187; EMF-Portal URL: https://www.emf-portal.org/en/article/5187

SP - 113-121

TI - Cytogenetic studies in human blood lymphocytes exposed in vitro to radiofrequency radiation at a cellular telephone frequency (835.62 MHz, FDMA)

ER -

TY - JOUR

IS - 1-2

JA - Mutat Res Genet Toxicol Environ Mutagen

JO - Mutation Research - Genetic Toxicology and Environmental Mutagenesis

PY - 2000

VL - 471

AU - Zotti-Martelli L

AU - Peccatori M

AU - Scarpato R

AU - Migliore L

DO - 10.1016/s1383-5718(00)00112-1

LA - en

N1 - FEMU ID: 5088; EMF-Portal URL: https://www.emf-portal.org/en/article/5088

SP - 51-58

TI - Induction of micronuclei in human lymphocytes exposed in vitro to microwave radiation

ER -

TY - JOUR

IS - 13

JO - Chemosphere

PY - 1999

SN - 0045-6535

VL - 39

AU - Garaj-Vrhovac V

DO - 10.1016/s0045-6535(99)00139-3

LA - en

N1 - FEMU ID: 5642; EMF-Portal URL: https://www.emf-portal.org/en/article/5642

SP - 2301-2312

TI - Micronucleus assay and lymphocyte mitotic activity in risk assessment of occupational exposure to microwave radiation

ER -

TY - JOUR

IS - 1

JA - Int J Radiat Biol

JO - International Journal of Radiation Biology

PY - 1999

SN - 0955-3002

VL - 75

AU - Vijayalaxmi

AU - Seaman RL

AU - Belt ML

AU - Doyle JM

AU - Mathur SP

AU - Prihoda TJ

DO - 10.1080/095530099140870

LA - en

N1 - FEMU ID: 2207; EMF-Portal URL: https://www.emf-portal.org/en/article/2207

SP - 115-120

TI - Frequency of micronuclei in the blood and bone marrow cells of mice exposed to ultra-wideband electromagnetic radiation

ER -

TY - JOUR

IS - 3

JA - Electro Magnetobiol

JO - Electro- and Magnetobiology

PY - 1997

SN - 1061-9526

VL - 16

AU - Pakhomova ON

AU - Belt MN

AU - Mathur SP

AU - Lee JC

AU - Akyel Y

DO - 10.3109/15368379709015652

LA - en

N1 - FEMU ID: 8582; EMF-Portal URL: https://www.emf-portal.org/en/article/8582

SP - 195-201

TI - Lack of genetic effects of ultra-wide band electromagnetic radiation in yeast

ER -

TY - JOUR

IS - 6

JA - Int J Radiat Biol

JO - International Journal of Radiation Biology

PY - 1997

SN - 0955-3002

VL - 72

AU - Vijayalaxmi

AU - Mohan N

AU - Meltz ML

AU - Wittler MA

DO - 10.1080/095530097142915

LA - en

N1 - FEMU ID: 2359; EMF-Portal URL: https://www.emf-portal.org/en/article/2359

SP - 751-757

TI - Proliferation and cytogenetic studies in human blood lymphocytes exposed in vitro to 2450 MHz radiofrequency radiation

ER -

TY - JOUR

IS - 4

JA - Radiat Res

JO - Radiation Research

PY - 1997

SN - 0033-7587

VL - 147

AU - Vijayalaxmi

AU - Frei MR

AU - Dusch SJ

AU - Guel V

AU - Meltz ML

AU - Jauchem JR

LA - en

N1 - FEMU ID: 1413; EMF-Portal URL: https://www.emf-portal.org/en/article/1413

SP - 495-500

TI - Frequency of micronuclei in the peripheral blood and bone marrow of cancer-prone mice chronically exposed to 2450 MHz radiofrequency radiation

ER -

TY - JOUR

JO - Edition Wissenschaft

PY - 1996

VL - 4

AU - Eberle P

AU - Erdtmann-Vourliotis M

AU - Diener S

AU - Finke HG

AU - Löffelholz B

AU - Schnor A

AU - Schräder M

LA - de

N1 - FEMU ID: 9467; EMF-Portal URL: https://www.emf-portal.org/en/article/9467

SP - 2-15

TI - Zellproliferation, Schwesterchromatidaustausche, Chromosomenaberrationen, Mikrokerne und Mutationsrate des HGPRT-Locus

UR - https://d-nb.info/974863475/34

ER -

TY - JOUR

IS - 1

JA - Sci Total Environ

JO - Science of the Total Evironment

PY - 1996

SN - 0048-9697

VL - 180

AU - Balode Z

DO - 10.1016/0048-9697(95)04923-1

LA - en

N1 - FEMU ID: 2132; EMF-Portal URL: https://www.emf-portal.org/en/article/2132

SP - 81-85

TI - Assessment of radio-frequency electromagnetic radiation by the micronucleus test in bovine peripheral erythrocytes

ER -

TY - JOUR

IS - 2

JA - Electro Magnetobiol

JO - Electro- and Magnetobiology

PY - 1996

SN - 1061-9526

VL - 15

AU - Scarfi MR

AU - Lioi MB

AU - d'Ambrosio G

AU - Massa R

AU - Zeni O

AU - Di Pietto R

AU - Di Berardino D

DO - 10.3109/15368379609009826

LA - en

N1 - FEMU ID: 959; EMF-Portal URL: https://www.emf-portal.org/en/article/959

SP - 99-107

TI - Genotoxic effects of mitomycin-c and microwave radiation on bovine lymphocytes

ER -

TY - JOUR

IS - 3

JA - Electro Magnetobiol

JO - Electro- and Magnetobiology

PY - 1995

SN - 1061-9526

VL - 14

AU - d'Ambrosio G

AU - Lioi MB

AU - Massa R

AU - Scarfi MR

AU - Zeni O

DO - 10.3109/15368379509030726

LA - en

N1 - FEMU ID: 905; EMF-Portal URL: https://www.emf-portal.org/en/article/905

SP - 157-164

TI - Genotoxic Effects of Amplitude-Modulated Microwaves on Human Lymphocytes Exposed in Vitro under Controlled Conditions

ER -

TY - JOUR

IS - 1-2

JO - Mutation Research - Letters

PY - 1994

VL - 328

AU - Haider T

AU - Knasmueller S

AU - Kundi M

AU - Haider M

DO - 10.1016/0165-7992(94)90069-8

LA - en

N1 - FEMU ID: 873; EMF-Portal URL: https://www.emf-portal.org/en/article/873

SP - 65-68

TI - Clastogenic effects of radiofrequency radiations on chromosomes of Tradescantia

ER -

TY - JOUR

IS - 6

JO - Bioelectromagnetics

PY - 1993

SN - 0197-8462

VL - 14

AU - Maes A

AU - Verschaeve L

AU - Arroyo A

AU - De Wagter C

AU - Vercruyssen L

DO - 10.1002/bem.2250140602

LA - en

N1 - FEMU ID: 889; EMF-Portal URL: https://www.emf-portal.org/en/article/889

SP - 495-501

TI - In vitro cytogenetic effects of 2450 MHz waves on human peripheral blood lymphocytes

ER -

TY - JOUR

IS - 3

JO - Mutation Research - Letters

PY - 1992

VL - 281

AU - Garaj-Vrhovac V

AU - Fucic A

AU - Horvat D

DO - 10.1016/0165-7992(92)90006-4

LA - en

N1 - FEMU ID: 876; EMF-Portal URL: https://www.emf-portal.org/en/article/876

SP - 181-186

TI - The correlation between the frequency of micronuclei and specific chromosome aberrations in human lymphocytes exposed to microwave radiation in vitro

ER -

TY - JOUR

IS - 4

JO - Mutation Research - Letters

PY - 1992

VL - 282

AU - Fucic A

AU - Garaj-Vrhovac V

AU - Skara M

AU - Dimitrovic B

DO - 10.1016/0165-7992(92)90133-3

LA - en

N1 - FEMU ID: 875; EMF-Portal URL: https://www.emf-portal.org/en/article/875

SP - 265-271

TI - X-rays, microwaves and vinyl chloride monomer: their clastogenic and aneugenic activity, using the micronucleus assay on human lymphocytes

ER -

TY - JOUR

IS - 3

JO - Mutation Research - Letters

PY - 1991

VL - 263

AU - Garaj-Vrhovac V

AU - Horvat D

AU - Koren Z

DO - 10.1016/0165-7992(91)90054-8

LA - en

N1 - FEMU ID: 877; EMF-Portal URL: https://www.emf-portal.org/en/article/877

SP - 143-149

TI - The relationship between colony-forming ability, chromosome aberrations and incidence of micronuclei in V79 Chinese hamster cells exposed to microwave radiation

ER -

TY - JOUR

IS - 4

JA - Period Biol

JO - Periodicum Biologorum

PY - 1990

SN - 0031-5362

VL - 92

AU - Garaj-Vrhovac V

AU - Fucic A

AU - Horvat D

LA - en

N1 - FEMU ID: 9585; EMF-Portal URL: https://www.emf-portal.org/en/article/9585

SP - 411-416

TI - Comparison of chromosome aberration and micronucleus induction in human lymphocytes after occupational exposure to vinyl chloride monomer and microwave radiation

ER -
