# Supplementary material for: A scoping review and evidence map of radiofrequency field exposure and genotoxicity: assessing in vivo, in vitro, and epidemiological data
Source: Front Public Health. 2025 Jul 30;13:1613353. doi: 10.3389/fpubh.2025.1613353 (PMC12343714; doi:10.3389/fpubh.2025.1613353)
Supplement: Supplementary file 3 [file Data_Sheet_3.zip › Search data/EMF Portal Search - Mutagen.docx]

EMF Portal search keywords

The following terms were included: Mutation, 突然変異, Mutagen, 突然変異原

TY - JOUR

IS - 3

JA - Int J Mol Sci

JO - International Journal of Molecular Sciences

PY - 2023

SN - 1422-0067

VL - 24

AU - Szilágyi Z

AU - Németh Z

AU - Bakos J

AU - Kubinyi G

AU - Necz PP

AU - Szabó E

AU - Thuróczy G

AU - Pinto R

AU - Selmaoui B

DO - 10.3390/ijms24032853

LA - en

N1 - FEMU ID: 49895; EMF-Portal URL: https://www.emf-portal.org/en/article/49895

SP - 2853

TI - Assessment of Inflammation in 3D Reconstructed Human Skin Exposed to Combined Exposure to Ultraviolet and Wi-Fi Radiation

UR - https://www.mdpi.com/1422-0067/24/3/2853/pdf?version=1675316287

ER -

TY - JOUR

IS - 1

JA - Environ Mol Mutagen

JO - Environmental and Molecular Mutagenesis

PY - 2022

SN - 0893-6692

VL - 63

AU - Zhou C

AU - Xiong L

AU - Zhou X

AU - Li L

AU - Yan Q

DO - 10.1002/em.22470

LA - en

N1 - FEMU ID: 46393; EMF-Portal URL: https://www.emf-portal.org/en/article/46393

SP - 29-36

TI - Transcriptome Profiling of Guinea Pig Skin Exposed to a High-Power Terahertz Source

UR - https://onlinelibrary.wiley.com/doi/epdf/10.1002/em.22470

ER -

TY - JOUR

IS - 5

JA - Turk J Biochem

JO - Turkish Journal of Biochemistry

PY - 2021

VL - 46

AU - Ozgur E

AU - Kayhan H

AU - Kismali G

AU - Senturk F

AU - Sensoz M

AU - Ozturk GG

AU - Sel T

DO - 10.1515/tjb-2020-0148

LA - en

N1 - FEMU ID: 45978; EMF-Portal URL: https://www.emf-portal.org/en/article/45978

SP - 525-532

TI - Effects of radiofrequency radiation on colorectal cancer cell proliferation and inflammation

UR - https://www.degruyter.com/document/doi/10.1515/tjb-2020-0148/pdf

ER -

TY - JOUR

IS - 2

JA - Electromagn Biol Med

JO - Electromagnetic Biology and Medicine

PY - 2021

SN - 1536-8386

VL - 40

AU - Gunes M

AU - Ates K

AU - Yalcin B

AU - Akkurt S

AU - Ozen S

AU - Kaya B

DO - 10.1080/15368378.2021.1878210

LA - en

N1 - FEMU ID: 44328; EMF-Portal URL: https://www.emf-portal.org/en/article/44328

SP - 254-263

TI - An Evaluation of the Genotoxic Effects of Electromagnetic Radiation at 900 MHz, 1800 MHz, and 2100 MHz Frequencies with a SMART Assay in Drosophila melanogaster

ER -

TY - JOUR

JA - Environ Pollut

JO - Environmental Pollution

PY - 2020

SN - 0269-7491

VL - 267

AU - Gulati S

AU - Kosik P

AU - Durdik M

AU - Skorvaga M

AU - Jakl L

AU - Markova E

AU - Belyaev I

DO - 10.1016/j.envpol.2020.115632

LA - en

N1 - FEMU ID: 43792; EMF-Portal URL: https://www.emf-portal.org/en/article/43792

SP - 115632

TI - Effects of different mobile phone UMTS signals on DNA, apoptosis and oxidative stress in human lymphocytes

ER -

TY - JOUR

IS - 2

JA - Environ Mol Mutagen

JO - Environmental and Molecular Mutagenesis

PY - 2020

SN - 0893-6692

VL - 61

AU - Smith-Roe SL

AU - Wyde ME

AU - Stout MD

AU - Winters JW

AU - Hobbs CA

AU - Shepard KG

AU - Green AS

AU - Kissling GE

AU - Shockley KR

AU - Tice RR

AU - Bucher JR

AU - Witt KL

DO - 10.1002/em.22343

LA - en

N1 - FEMU ID: 39949; EMF-Portal URL: https://www.emf-portal.org/en/article/39949

SP - 276-290

TI - Evaluation of the genotoxicity of cell phone radiofrequency radiation in male and female rats and mice following subchronic exposure

ER -

TY - JOUR

JA - Sci Rep

JO - Scientific Reports

PY - 2019

SN - 2045-2322

VL - 9

AU - Durdik M

AU - Kosik P

AU - Markova E

AU - Somsedikova A

AU - Gajdosechova B

AU - Nikitina E

AU - Horvathova E

AU - Kozics K

AU - Davis D

AU - Belyaev I

DO - 10.1038/s41598-019-52389-x

LA - en

N1 - FEMU ID: 40421; EMF-Portal URL: https://www.emf-portal.org/en/article/40421

SP - 16182

TI - Microwaves from mobile phone induce reactive oxygen species but not DNA damage, preleukemic fusion genes and apoptosis in hematopoietic stem/progenitor cells

UR - https://www.nature.com/articles/s41598-019-52389-x.pdf

ER -

TY - JOUR

IS - 4

JA - Exp Oncol

JO - Experimental Oncology

PY - 2018

SN - 1812-9269

VL - 40

AU - Yakymenko I

AU - Burlaka A

AU - Tsybulin I

AU - Brieieva I

AU - Buchynska L

AU - Tsehmistrenko I

AU - Chekhun F

LA - en

N1 - FEMU ID: 36998; EMF-Portal URL: https://www.emf-portal.org/en/article/36998

SP - 282-287

TI - Oxidative and mutagenic effects of low intensity GSM 1800 MHz microwave radiation

UR - https://exp-oncology.com.ua/wp/wp-content/uploads/2018/12/2458.pdf?upload=

ER -

TY - JOUR

IS - 2

JA - Int J Oncol

JO - International Journal of Oncology

PY - 2018

SN - 1019-6439

VL - 53

AU - Zhao YY

AU - Wu Q

AU - Wu ZB

AU - Zhang JJ

AU - Zhu LC

AU - Yang Y

AU - Ma SL

AU - Zhang SR

DO - 10.3892/ijo.2018.4439

LA - en

N1 - FEMU ID: 35311; EMF-Portal URL: https://www.emf-portal.org/en/article/35311

SP - 539-550

TI - Microwave hyperthermia promotes caspase‑3-dependent apoptosis and induces G2/M checkpoint arrest via the ATM pathway in non‑small cell lung cancer cells

UR - https://www.spandidos-publications.com/10.3892/ijo.2018.4439/download

ER -

TY - JOUR

IS - 6

JA - Anticancer Res

JO - Anticancer Research

PY - 2018

SN - 0250-7005

VL - 38

AU - Narvaez CJ

AU - Mall SK

AU - Fountain A

AU - Parr BA

AU - Chittur SV

AU - Kokorin BI

AU - Botsford SF

AU - Startari JF

DO - 10.21873/anticanres.12590

LA - en

N1 - FEMU ID: 35234; EMF-Portal URL: https://www.emf-portal.org/en/article/35234

SP - 3255-3266

TI - Specifically Targeted Electromagnetic Fields Arrest Proliferation of Glioblastoma Multiforme U-87 Cells in Culture

UR - https://ar.iiarjournals.org/content/anticanres/38/6/3255.full.pdf

ER -

TY - JOUR

IS - 6

JA - Environ Mol Mutagen

JO - Environmental and Molecular Mutagenesis

PY - 2018

SN - 0893-6692

VL - 59

AU - Franchini V

AU - De Sanctis S

AU - Marinaccio J

AU - De Amicis A

AU - Coluzzi E

AU - Di Cristofaro S

AU - Lista F

AU - Regalbuto E

AU - Doria A

AU - Giovenale E

AU - Gallerano GP

AU - Bei R

AU - Benvenuto M

AU - Masuelli L

AU - Udroiu I

AU - Sgura A

DO - 10.1002/em.22192

LA - en

N1 - FEMU ID: 34868; EMF-Portal URL: https://www.emf-portal.org/en/article/34868

SP - 476-487

TI - Study of the effects of 0.15 terahertz radiation on genome integrity of adult fibroblasts

UR - https://onlinelibrary.wiley.com/doi/epdf/10.1002/em.22192

ER -

TY - GEN

ET - 1

PB - IEEE

PP - Cancun, Mexico

PY - 2017

SN - 9781509060504

T2 - 2017 42nd International Conference on Infrared, Millimeter, and Terahertz Waves (IRMMW-THz), Cancun

AU - Hough CM

AU - Purschke DN

AU - Huang C

AU - Titova LV

AU - Kovalchuk O

AU - Warkentin BJ

AU - Hegmann FA

DO - 10.1109/IRMMW-THz.2017.8066865

LA - en

N1 - FEMU ID: 33630; EMF-Portal URL: https://www.emf-portal.org/en/article/33630

TI - Biological effects of intense THz pulses on human skin tissue models

ER -

TY - JOUR

JA - Mutat Res Genet Toxicol Environ Mutagen

JO - Mutation Research - Genetic Toxicology and Environmental Mutagenesis

PY - 2017

VL - 822

AU - de Oliveira FM

AU - Carmona AM

AU - Ladeira C

DO - 10.1016/j.mrgentox.2017.08.001

LA - en

N1 - FEMU ID: 32942; EMF-Portal URL: https://www.emf-portal.org/en/article/32942

SP - 41-46

TI - Is mobile phone radiation genotoxic? An analysis of micronucleus frequency in exfoliated buccal cells

ER -

TY - JOUR

JA - Mutat Res Genet Toxicol Environ Mutagen

JO - Mutation Research - Genetic Toxicology and Environmental Mutagenesis

PY - 2017

VL - 820

AU - He Q

AU - Zong L

AU - Sun Y

AU - Vijayalaxmi

AU - Prihoda TJ

AU - Tong J

AU - Cao Y

DO - 10.1016/j.mrgentox.2017.05.007

LA - en

N1 - FEMU ID: 32408; EMF-Portal URL: https://www.emf-portal.org/en/article/32408

SP - 19-25

TI - Adaptive response in mouse bone marrow stromal cells exposed to 900MHz radiofrequency fields: Impact of poly (ADP-ribose) polymerase (PARP)

ER -

TY - JOUR

IS - 6

JO - Bioelectromagnetics

PY - 2017

SN - 0197-8462

VL - 38

AU - Suzuki S

AU - Okutsu M

AU - Suganuma R

AU - Komiya H

AU - Nakatani-Enomoto S

AU - Kobayashi S

AU - Ugawa Y

AU - Tateno H

AU - Fujimori K

DO - 10.1002/bem.22063

LA - en

N1 - FEMU ID: 32274; EMF-Portal URL: https://www.emf-portal.org/en/article/32274

SP - 466-473

TI - Influence of radiofrequency-electromagnetic waves from 3rd-generation cellular phones on fertilization and embryo development in mice

ER -

TY - JOUR

JO - Mutation Research - Fundamental and Molecular Mechanism of Mutagenesis

PY - 2017

SN - 0027-5107

VL - 797-799

AU - Sun Y

AU - Zong L

AU - Gao Z

AU - Zhu S

AU - Tong J

AU - Cao Y

DO - 10.1016/j.mrfmmm.2017.03.001

LA - en

N1 - FEMU ID: 31583; EMF-Portal URL: https://www.emf-portal.org/en/article/31583

SP - 7-14

TI - Mitochondrial DNA damage and oxidative damage in HL-60 cells exposed to 900MHz radiofrequency fields

ER -

TY - JOUR

JA - Toxicol In Vitro

JO - Toxicology in Vitro

PY - 2017

SN - 0887-2333

VL - 40

AU - Al-Serori H

AU - Kundi M

AU - Ferk F

AU - Mišík M

AU - Nersesyan A

AU - Murbach M

AU - Lah TT

AU - Knasmüller S

DO - 10.1016/j.tiv.2017.01.012

LA - en

N1 - FEMU ID: 31101; EMF-Portal URL: https://www.emf-portal.org/en/article/31101

SP - 264-271

TI - Evaluation of the potential of mobile phone specific electromagnetic fields (UMTS) to produce micronuclei in human glioblastoma cell lines

ER -

TY - JOUR

IS - 4

JO - Bioelectromagnetics

PY - 2017

SN - 0197-8462

VL - 38

AU - Sannino A

AU - Zeni O

AU - Romeo S

AU - Massa R

AU - Scarfi MR

DO - 10.1002/bem.22034

LA - en

N1 - FEMU ID: 30897; EMF-Portal URL: https://www.emf-portal.org/en/article/30897

SP - 245-254

TI - Adverse and beneficial effects in Chinese hamster lung fibroblast cells following radiofrequency exposure

ER -

TY - JOUR

IS - 4

JA - Toxicol Ind Health

JO - Toxicology and Industrial Health

PY - 2017

SN - 0748-2337

VL - 33

AU - Pandey N

AU - Giri S

AU - Das S

AU - Upadhaya P

DO - 10.1177/0748233716671206

LA - en

N1 - FEMU ID: 30481; EMF-Portal URL: https://www.emf-portal.org/en/article/30481

SP - 373-384

TI - Radiofrequency radiation (900 MHz)-induced DNA damage and cell cycle arrest in testicular germ cells in swiss albino mice

ER -

TY - JOUR

JA - Sci Rep

JO - Scientific Reports

PY - 2016

SN - 2045-2322

VL - 6

AU - Sun C

AU - Wei X

AU - Fei Y

AU - Su L

AU - Zhao X

AU - Chen G

AU - Xu Z

DO - 10.1038/srep37423

LA - en

N1 - FEMU ID: 30619; EMF-Portal URL: https://www.emf-portal.org/en/article/30619

SP - 37423

TI - Mobile phone signal exposure triggers a hormesis-like effect in Atm+/+ and Atm-/- mouse embryonic fibroblasts

UR - http://www.nature.com/articles/srep37423.pdf

ER -

TY - JOUR

JA - J Chem Neuroanat

JO - Journal of Chemical Neuroanatomy

PY - 2016

SN - 0891-0618

VL - 78

AU - Hussein S

AU - El-Saba AA

AU - Galal MK

DO - 10.1016/j.jchemneu.2016.07.009

LA - en

N1 - FEMU ID: 30004; EMF-Portal URL: https://www.emf-portal.org/en/article/30004

SP - 10-19

TI - Biochemical and histological studies on adverse effects of mobile phone radiation on rat's brain

ER -

TY - JOUR

JA - Mutat Res Genet Toxicol Environ Mutagen

JO - Mutation Research - Genetic Toxicology and Environmental Mutagenesis

PY - 2016

VL - 803

AU - Sergeeva S

AU - Demidova E

AU - Sinitsyna O

AU - Goryachkovskaya T

AU - Bryanskaya A

AU - Semenov A

AU - Meshcheryakova I

AU - Dianov G

AU - Popik V

AU - Peltek S

DO - 10.1016/j.mrgentox.2016.05.005

LA - en

N1 - FEMU ID: 29619; EMF-Portal URL: https://www.emf-portal.org/en/article/29619

SP - 34-38

TI - 2.3THz radiation: Absence of genotoxicity/mutagenicity in Escherichia coli and Salmonella typhimurium

ER -

TY - JOUR

IS - 2

JA - Cell Biochem Biophys

JO - Cell Biochemistry and Biophysics

PY - 2016

SN - 1085-9195

VL - 74

AU - Kayhan H

AU - Esmekaya MA

AU - Saglam AS

AU - Tuysuz MZ

AU - Canseven AG

AU - Yagci AM

AU - Seyhan N

DO - 10.1007/s12013-016-0734-9

LA - en

N1 - FEMU ID: 29618; EMF-Portal URL: https://www.emf-portal.org/en/article/29618

SP - 99-107

TI - Does MW Radiation Affect Gene Expression, Apoptotic Level, and Cell Cycle Progression of Human SH-SY5Y Neuroblastoma Cells?

ER -

TY - JOUR

IS - 3

JA - J Clin of Diagn Res

JO - Journal of Clinical and Diagnostic Research

PY - 2016

SN - 0973-709X

VL - 10

AU - Banerjee S

AU - Singh NN

AU - Sreedhar G

AU - Mukherjee S

DO - 10.7860/JCDR/2016/17592.7505

LA - en

N1 - FEMU ID: 29426; EMF-Portal URL: https://www.emf-portal.org/en/article/29426

SP - ZC82-ZC85

TI - Analysis of the Genotoxic Effects of Mobile Phone Radiation using Buccal Micronucleus Assay: A Comparative Evaluation

UR - https://www.ncbi.nlm.nih.gov/pmc/articles/PMC4843394/pdf/jcdr-10-ZC82.pdf

ER -

TY - JOUR

IS - 1

JA - Oxid Antioxid Med Sci

JO - Oxidants and Antioxidants in Medical Science

PY - 2016

VL - 5

AU - Tsybulin O

AU - Sidorik E

AU - Kyrylenko S

AU - Yakymenko I

DO - 10.5455/oams.010216.or.092

LA - en

N1 - FEMU ID: 29343; EMF-Portal URL: https://www.emf-portal.org/en/article/29343

SP - 21-27

TI - Monochromatic red light of LED protects embryonic cells from oxidative stress caused by radiofrequency radiation

ER -

TY - JOUR

IS - 3

JA - Saudi J Biol Sci

JO - Saudi Journal of Biological Sciences

PY - 2016

SN - 1319-562X

VL - 23

AU - Shahin-Jafari A

AU - Bayat M

AU - Shahhosseiny MH

AU - Tajik P

AU - Roudbar-Mohammadi S

DO - 10.1016/j.sjbs.2015.05.001

LA - en

N1 - FEMU ID: 29289; EMF-Portal URL: https://www.emf-portal.org/en/article/29289

SP - 426-433

TI - Effect of long-term exposure to mobile phone radiation on alpha-Int1 gene sequence of Candida albicans

UR - https://www.sciencedirect.com/science/article/pii/S1319562X15001023/pdfft?md5=d115cdd86917cb8a9bcc6e2d5c318361&pid=1-s2.0-S1319562X15001023-main.pdf

ER -

TY - JOUR

JA - J Chem Neuroanat

JO - Journal of Chemical Neuroanatomy

PY - 2016

SN - 0891-0618

VL - 75

AU - Sahin D

AU - Ozgur E

AU - Guler G

AU - Tomruk A

AU - Unlu I

AU - Sepici-Dincel A

AU - Seyhan N

DO - 10.1016/j.jchemneu.2016.01.002

LA - en

N1 - FEMU ID: 28658; EMF-Portal URL: https://www.emf-portal.org/en/article/28658

SP - 94-98

TI - The 2100 MHz radiofrequency radiation of a 3G-mobile phone and the DNA oxidative damage in brain

ER -

TY - JOUR

IS - 2

JO - Mutagenesis

PY - 2016

SN - 0267-8357

VL - 31

AU - Gustavino B

AU - Carboni G

AU - Petrillo R

AU - Paoluzzi G

AU - Santovetti E

AU - Rizzoni M

DO - 10.1093/mutage/gev071

LA - en

N1 - FEMU ID: 28074; EMF-Portal URL: https://www.emf-portal.org/en/article/28074

SP - 187-192

TI - Exposure to 915 MHz radiation induces micronuclei in Vicia faba root tips

UR - https://academic.oup.com/mutage/article-pdf/31/2/187/8178338/gev071.pdf

ER -

TY - GEN

ET - 1

PB - IEEE

PY - 2015

SN - 9781424492718

T2 - 2015 37th Annual International Conference of the IEEE Engineering in Medicine and Biology Society (EMBC), Milan, Italy

AU - Moraitis N

AU - Christopoulou M

AU - Nikita KS

AU - Voulgaridou GP

AU - Anestopoulos I

AU - Panagiotidis MI

AU - Pappa A

DO - 10.1109/EMBC.2015.7318922

LA - en

N1 - FEMU ID: 28545; EMF-Portal URL: https://www.emf-portal.org/en/article/28545

SP - 2592-2595

TI - In-vitro assessment of Jurkat T-cells response to 1966 MHz electromagnetic fields in a GTEM cell

ER -

TY - JOUR

JA - Mutat Res Genet Toxicol Environ Mutagen

JO - Mutation Research - Genetic Toxicology and Environmental Mutagenesis

PY - 2015

VL - 793

AU - Amicis A

AU - Sanctis S

AU - Cristofaro SD

AU - Franchini V

AU - Lista F

AU - Regalbuto E

AU - Giovenale E

AU - Gallerano GP

AU - Nenzi P

AU - Bei R

AU - Fantini M

AU - Benvenuto M

AU - Masuelli L

AU - Coluzzi E

AU - Cicia C

AU - Sgura A

DO - 10.1016/j.mrgentox.2015.06.003

LA - en

N1 - FEMU ID: 28170; EMF-Portal URL: https://www.emf-portal.org/en/article/28170

SP - 150-160

TI - Biological effects of in vitro THz radiation exposure in human foetal fibroblasts

ER -

TY - JOUR

JA - Mutat Res Genet Toxicol Environ Mutagen

JO - Mutation Research - Genetic Toxicology and Environmental Mutagenesis

PY - 2015

VL - 792

AU - Zhu S

AU - Zhang J

AU - Liu C

AU - He Q

AU - Vijayalaxmi

AU - Prihoda TJ

AU - Tong J

AU - Cao Y

DO - 10.1016/j.mrgentox.2015.07.004

LA - en

N1 - FEMU ID: 28010; EMF-Portal URL: https://www.emf-portal.org/en/article/28010

SP - 53-57

TI - Dominant lethal mutation test in male mice exposed to 900MHz radiofrequency fields

ER -

TY - JOUR

IS - 3

JA - Cell Physiol Biochem

JO - Cellular Physiology and Biochemistry

PY - 2015

SN - 1015-8987

VL - 37

AU - Wang X

AU - Liu C

AU - Ma Q

AU - Feng W

AU - Yang L

AU - Lu Y

AU - Zhou Z

AU - Yu Z

AU - Li W

AU - Zhang L

DO - 10.1159/000430233

LA - en

N1 - FEMU ID: 27920; EMF-Portal URL: https://www.emf-portal.org/en/article/27920

SP - 1075-1088

TI - 8-oxoG DNA Glycosylase-1 Inhibition Sensitizes Neuro-2a Cells to Oxidative DNA Base Damage Induced by 900 MHz Radiofrequency Electromagnetic Radiation

UR - https://www.karger.com/Article/Pdf/430233

ER -

TY - JOUR

IS - 11

JA - Int J Radiat Biol

JO - International Journal of Radiation Biology

PY - 2015

SN - 0955-3002

VL - 91

AU - Furtado-Filho OV

AU - Borba JB

AU - Maraschin T

AU - Souza LM

AU - Henriques JA

AU - Moreira JC

AU - Saffi J

DO - 10.3109/09553002.2015.1083629

LA - en

N1 - FEMU ID: 27729; EMF-Portal URL: https://www.emf-portal.org/en/article/27729

SP - 891-897

TI - Effects of chronic exposure to 950 MHz ultra-high-frequency electromagnetic radiation on reactive oxygen species metabolism in the right and left cerebral cortex of young rats of different ages

ER -

TY - JOUR

IS - 1

JA - Int J Fertil Steril

JO - International Journal of Fertility & Sterility

PY - 2015

SN - 2008-0778

VL - 9

AU - Zalata A

AU - El-Samanoudy AZ

AU - Shaalan D

AU - El-Baiomy Y

AU - Mostafa T

DO - 10.22074/ijfs.2015.4217

LA - en

N1 - FEMU ID: 26969; EMF-Portal URL: https://www.emf-portal.org/en/article/26969

SP - 129-136

TI - In vitro effect of cell phone radiation on motility, DNA fragmentation and clusterin gene expression in human sperm

UR - https://www.ncbi.nlm.nih.gov/pmc/articles/PMC4410031/pdf/Int-J-Fertil-Steril-9-129.pdf

ER -

TY - JOUR

IS - 4

JA - Int J Environ Res Public Health

JO - International Journal of Environmental Research and Public Health

PY - 2015

SN - 1660-4601

VL - 12

AU - Mizuno K

AU - Shinohara N

AU - Miyakoshi J

DO - 10.3390/ijerph120403853

LA - en

N1 - FEMU ID: 26850; EMF-Portal URL: https://www.emf-portal.org/en/article/26850

SP - 3853-3863

TI - In Vitro Evaluation of Genotoxic Effects under Magnetic Resonant Coupling Wireless Power Transfer

UR - http://www.mdpi.com/1660-4601/12/4/3853

ER -

TY - JOUR

IS - 3

JA - Int J Toxicol

JO - International Journal of Toxicology

PY - 2015

SN - 1091-5818

VL - 34

AU - Deshmukh PS

AU - Nasare N

AU - Megha K

AU - Banerjee BD

AU - Ahmed RS

AU - Singh D

AU - Abegaonkar MP

AU - Tripathi AK

AU - Mediratta PK

DO - 10.1177/1091581815574348

LA - en

N1 - FEMU ID: 26650; EMF-Portal URL: https://www.emf-portal.org/en/article/26650

SP - 284-290

TI - Cognitive impairment and neurogenotoxic effects in rats exposed to low-intensity microwave radiation

ER -

TY - JOUR

IS - 3

JA - Radiat Res

JO - Radiation Research

PY - 2015

SN - 0033-7587

VL - 183

AU - Duan W

AU - Liu C

AU - Zhang L

AU - He M

AU - Xu S

AU - Chen C

AU - Pi H

AU - Gao P

AU - Zhang Y

AU - Zhong M

AU - Yu Z

AU - Zhou Z

DO - 10.1667/RR13851.1

LA - en

N1 - FEMU ID: 26541; EMF-Portal URL: https://www.emf-portal.org/en/article/26541

SP - 305-314

TI - Comparison of the genotoxic effects induced by 50 Hz extremely low-frequency electromagnetic fields and 1800 MHz radiofrequency electromagnetic fields in GC-2 cells

ER -

TY - JOUR

IS - 3

JA - Int J Radiat Biol

JO - International Journal of Radiation Biology

PY - 2015

SN - 0955-3002

VL - 91

AU - Zong C

AU - Ji Y

AU - He Q

AU - Zhu S

AU - Qin F

AU - Tong J

AU - Cao Y

DO - 10.3109/09553002.2014.980465

LA - en

N1 - FEMU ID: 25895; EMF-Portal URL: https://www.emf-portal.org/en/article/25895

SP - 270-276

TI - Adaptive response in mice exposed to 900 MHz radiofrequency fields: Bleomycin-induced DNA and oxidative damage/repair

ER -

TY - JOUR

IS - 1

JA - Electromagn Biol Med

JO - Electromagnetic Biology and Medicine

PY - 2015

SN - 1536-8386

VL - 34

AU - Hou Q

AU - Wang M

AU - Wu S

AU - Ma X

AU - An G

AU - Liu H

AU - Xie F

DO - 10.3109/15368378.2014.900507

LA - en

N1 - FEMU ID: 24552; EMF-Portal URL: https://www.emf-portal.org/en/article/24552

SP - 85-92

TI - Oxidative changes and apoptosis induced by 1800-MHz electromagnetic radiation in NIH/3T3 cells

ER -

TY - JOUR

IS - 3

JO - Rare Tumors

PY - 2014

SN - 2036-3605

VL - 6

AU - Akhavan-Sigari R

AU - Baf MM

AU - Ariabod V

AU - Rohde V

AU - Rahighi S

DO - 10.4081/rt.2014.5350

LA - en

N1 - FEMU ID: 25830; EMF-Portal URL: https://www.emf-portal.org/en/article/25830

SP - 5350

TI - Connection between Cell Phone use, p53 Gene Expression in Different Zones of Glioblastoma Multiforme and Survival Prognoses

UR - https://www.ncbi.nlm.nih.gov/pmc/articles/PMC4178273/pdf/rt-2014-3-5350.pdf

ER -

TY - JOUR

IS - 9

JA - Indian J Exp Biol

JO - Indian Journal of Experimental Biology

PY - 2014

SN - 0019-5189

VL - 52

AU - Kumar S

AU - Nirala JP

AU - Behari J

AU - Paulraj R

LA - en

N1 - FEMU ID: 25773; EMF-Portal URL: https://www.emf-portal.org/en/article/25773

SP - 890-897

TI - Effect of electromagnetic irradiation produced by 3G mobile phone on male rat reproductive system in a simulated scenario

UR - http://nopr.niscpr.res.in/bitstream/123456789/29335/1/IJEB%2052%289%29%20890-897.pdf

ER -

TY - JOUR

IS - 1

JA - Cent European J Urol

JO - Central European Journal of Urology

PY - 2014

SN - 2080-4806

VL - 67

AU - Gorpinchenko I

AU - Nikitin O

AU - Banyra O

AU - Shulyak A

DO - 10.5173/ceju.2014.01.art14

LA - en

N1 - FEMU ID: 25254; EMF-Portal URL: https://www.emf-portal.org/en/article/25254

SP - 65-71

TI - The influence of direct mobile phone radiation on sperm quality

UR - http://ceju.online/journal/2014/commenting-on-gorpinchenko-et-al-the-influence-of-direct-mobile-phone-radiation-on-sperm-439.php

ER -

TY - JOUR

IS - 4

JA - Electromagn Biol Med

JO - Electromagnetic Biology and Medicine

PY - 2014

SN - 1536-8386

VL - 33

AU - Gurbuz N

AU - Sirav B

AU - Colbay M

AU - Yetkin I

AU - Seyhan N

DO - 10.3109/15368378.2013.831354

LA - en

N1 - FEMU ID: 23927; EMF-Portal URL: https://www.emf-portal.org/en/article/23927

SP - 296-301

TI - No genotoxic effect in exfoliated bladder cells of rat under the exposure of 1800 and 2100 MHz radio frequency radiation

ER -

TY - JOUR

IS - 2

JA - J Radiat Res

JO - Journal of Radiation Research

PY - 2014

SN - 0449-3060

VL - 55

AU - Sannino A

AU - Zeni O

AU - Romeo S

AU - Massa R

AU - Gialanella G

AU - Grossi G

AU - Manti L

AU - Vijayalaxmi

AU - Scarfi MR

DO - 10.1093/jrr/rrt106

LA - en

N1 - FEMU ID: 23403; EMF-Portal URL: https://www.emf-portal.org/en/article/23403

SP - 210-217

TI - Adaptive response in human blood lymphocytes exposed to non-ionizing radiofrequency fields: resistance to ionizing radiation-induced damage

UR - https://academic.oup.com/jrr/article-pdf/55/2/210/2797100/rrt106.pdf

ER -

TY - JOUR

IS - 2

JA - Cell Biochem Biophys

JO - Cell Biochemistry and Biophysics

PY - 2014

SN - 1085-9195

VL - 68

AU - Kesari KK

AU - Meena R

AU - Nirala J

AU - Kumar J

AU - Verma HN

DO - 10.1007/s12013-013-9715-4

LA - en

N1 - FEMU ID: 23361; EMF-Portal URL: https://www.emf-portal.org/en/article/23361

SP - 347-358

TI - Effect of 3G Cell Phone Exposure with Computer Controlled 2-D Stepper Motor on Non-thermal Activation of the hsp27/p38MAPK Stress Pathway in Rat Brain

ER -

TY - JOUR

IS - 2

JA - Int J Radiat Biol

JO - International Journal of Radiation Biology

PY - 2014

SN - 0955-3002

VL - 90

AU - Furtado-Filho OV

AU - Borba JB

AU - Dallegrave A

AU - Pizzolato TM

AU - Henriques JA

AU - Moreira JC

AU - Saffi J

DO - 10.3109/09553002.2013.817697

LA - en

N1 - FEMU ID: 22969; EMF-Portal URL: https://www.emf-portal.org/en/article/22969

SP - 159-168

TI - Effect of 950 MHz UHF Electromagnetic radiation on biomarkers of oxidative damage, metabolism of UFA and antioxidants in the liver of young rats of different ages

ER -

TY - JOUR

IS - 2

JA - Electromagn Biol Med

JO - Electromagnetic Biology and Medicine

PY - 2014

SN - 1536-8386

VL - 33

AU - Souza LCM

AU - Cerqueira Ede M

AU - Meireles JR

DO - 10.3109/15368378.2013.783856

LA - en

N1 - FEMU ID: 22645; EMF-Portal URL: https://www.emf-portal.org/en/article/22645

SP - 98-102

TI - Assessment of nuclear abnormalities in exfoliated cells from the oral epithelium of mobile phone users

ER -

TY - JOUR

IS - 2

JA - Electromagn Biol Med

JO - Electromagnetic Biology and Medicine

PY - 2014

SN - 1536-8386

VL - 33

AU - Meena R

AU - Kumari K

AU - Kumar J

AU - Rajamani P

AU - Verma HN

AU - Kesari KK

DO - 10.3109/15368378.2013.781035

LA - en

N1 - FEMU ID: 22452; EMF-Portal URL: https://www.emf-portal.org/en/article/22452

SP - 81-91

TI - Therapeutic approaches of melatonin in microwave radiations-induced oxidative stress-mediated toxicity on male fertility pattern of Wistar rats

ER -

TY - JOUR

IS - 11

JA - J Endocrinol Invest

JO - Journal of Endocrinological Investigation

PY - 2013

SN - 0391-4097

VL - 36

AU - Rago R

AU - Salacone P

AU - Caponecchia L

AU - Sebastianelli A

AU - Marcucci I

AU - Calogero AE

AU - Condorelli R

AU - Vicari E

AU - Morgia G

AU - Favilla V

AU - Cimino S

AU - Arcoria AF

AU - La Vignera S

DO - 10.3275/8996

LA - en

N1 - FEMU ID: 25784; EMF-Portal URL: https://www.emf-portal.org/en/article/25784

SP - 970-974

TI - The semen quality of the mobile phone users

ER -

TY - JOUR

JA - Reprod Toxicol

JO - Reproductive Toxicology

PY - 2013

SN - 0890-6238

VL - 42

AU - Hanci H

AU - Odaci E

AU - Kaya H

AU - Aliyazicioglu Y

AU - Turan I

AU - Demir S

AU - Colakoglu S

DO - 10.1016/j.reprotox.2013.09.006

LA - en

N1 - FEMU ID: 23648; EMF-Portal URL: https://www.emf-portal.org/en/article/23648

SP - 203-209

TI - The effect of prenatal exposure to 900-megahertz electromagnetic field on the 21-old-day rat testicle

ER -

TY - JOUR

IS - 3

JA - Exp Oncol

JO - Experimental Oncology

PY - 2013

SN - 1812-9269

VL - 35

AU - Burlaka A

AU - Tsybulin O

AU - Sidorik E

AU - Lukin S

AU - Polishuk V

AU - Tsehmistrenko S

AU - Yakymenko I

LA - en

N1 - FEMU ID: 23628; EMF-Portal URL: https://www.emf-portal.org/en/article/23628

SP - 219-225

TI - Overproduction of free radical species in embryonal cells exposed to low intensity radiofrequency radiation

UR - https://exp-oncology.com.ua/article/6079

ER -

TY - JOUR

IS - 11

JA - Int J Radiat Biol

JO - International Journal of Radiation Biology

PY - 2013

SN - 0955-3002

VL - 89

AU - Liu C

AU - Gao P

AU - Xu SC

AU - Wang Y

AU - Chen CH

AU - He MD

AU - Yu ZP

AU - Zhang L

AU - Zhou Z

DO - 10.3109/09553002.2013.811309

LA - en

N1 - FEMU ID: 23360; EMF-Portal URL: https://www.emf-portal.org/en/article/23360

SP - 993-1001

TI - Mobile phone radiation induces mode-dependent DNA damage in a mouse spermatocyte-derived cell line: a protective role of melatonin

ER -

TY - JOUR

IS - 1

JA - Toxicol Int

JO - Toxicology International

PY - 2013

SN - 0971-6580

VL - 20

AU - Deshmukh PS

AU - Megha K

AU - Banerjee BD

AU - Ahmed RS

AU - Chandna S

AU - Abegaonkar MP

AU - Tripathi AK

DO - 10.4103/0971-6580.111549

LA - en

N1 - FEMU ID: 22984; EMF-Portal URL: https://www.emf-portal.org/en/article/22984

SP - 19-24

TI - Detection of Low Level Microwave Radiation Induced Deoxyribonucleic Acid Damage Vis-a-vis Genotoxicity in Brain of Fischer Rats

UR - https://www.ncbi.nlm.nih.gov/pmc/articles/PMC3702122/?report=printable

ER -

TY - JOUR

IS - 2

JA - Mutat Res Genet Toxicol Environ Mutagen

JO - Mutation Research - Genetic Toxicology and Environmental Mutagenesis

PY - 2013

VL - 755

AU - Speit G

AU - Gminski R

AU - Tauber R

DO - 10.1016/j.mrgentox.2013.06.014

LA - en

N1 - FEMU ID: 22960; EMF-Portal URL: https://www.emf-portal.org/en/article/22960

SP - 163-166

TI - Genotoxic effects of exposure to radiofrequency electromagnetic fields (RF-EMF) in HL-60 cells are not reproducible

ER -

TY - JOUR

IS - 7

JO - Bioelectromagnetics

PY - 2013

SN - 0197-8462

VL - 34

AU - Vijayalaxmi

AU - Reddy AB

AU - McKenzie RJ

AU - McIntosh RL

AU - Prihoda TJ

AU - Wood AW

DO - 10.1002/bem.21798

LA - en

N1 - FEMU ID: 22648; EMF-Portal URL: https://www.emf-portal.org/en/article/22648

SP - 542-548

TI - Incidence of micronuclei in human peripheral blood lymphocytes exposed to modulated and unmodulated 2450 MHz radiofrequency fields

ER -

TY - JOUR

IS - 11

JA - Int J Radiat Biol

JO - International Journal of Radiation Biology

PY - 2013

SN - 0955-3002

VL - 89

AU - Atli Sekeroglu Z

AU - Akar A

AU - Sekeroglu V

DO - 10.3109/09553002.2013.809170

LA - en

N1 - FEMU ID: 22646; EMF-Portal URL: https://www.emf-portal.org/en/article/22646

SP - 985-992

TI - Evaluation of the cytogenotoxic damage in immature and mature rats exposed to 900 MHz radiofrequency electromagnetic fields

ER -

TY - JOUR

IS - 9

JA - Int J Radiat Biol

JO - International Journal of Radiation Biology

PY - 2013

SN - 0955-3002

VL - 89

AU - Tsybulin O

AU - Sidorik E

AU - Brieieva O

AU - Buchynska L

AU - Kyrylenko S

AU - Henshel D

AU - Yakymenko I

DO - 10.3109/09553002.2013.791408

LA - en

N1 - FEMU ID: 22134; EMF-Portal URL: https://www.emf-portal.org/en/article/22134

SP - 756-763

TI - GSM 900 MHz cellular phone radiation can either stimulate or depress early embryogenesis in Japanese quails depending on the duration of exposure

ER -

TY - JOUR

IS - 1

JA - Gen Physiol Biophys

JO - General Physiology and Biophysics

PY - 2013

SN - 0231-5882

VL - 32

AU - Sokolovic D

AU - Djordjevic B

AU - Kocic G

AU - Veljkovic A

AU - Marinkovic M

AU - Basic J

AU - Jevtovic-Stoimenov T

AU - Stanojkovic Z

AU - Sokolovic DM

AU - Pavlovic V

AU - Djindjic B

AU - Krstic D

DO - 10.4149/gpb_2013002

LA - en

N1 - FEMU ID: 21991; EMF-Portal URL: https://www.emf-portal.org/en/article/21991

SP - 79-90

TI - Melatonin protects rat thymus against oxidative stress caused by exposure to microwaves and modulates proliferation/apoptosis of thymocytes

ER -

TY - JOUR

JA - Ecotoxicol Environ Saf

JO - Ecotoxicology and Environmental Safety

PY - 2013

SN - 0147-6513

VL - 90

AU - Tkalec M

AU - Stambuk A

AU - Srut M

AU - Malaric K

AU - Klobucar GI

DO - 10.1016/j.ecoenv.2012.12.005

LA - en

N1 - FEMU ID: 21702; EMF-Portal URL: https://www.emf-portal.org/en/article/21702

SP - 7-12

TI - Oxidative and genotoxic effects of 900 MHz electromagnetic fields in the earthworm Eisenia fetida

ER -

TY - JOUR

IS - 1

JO - PLoS One

PY - 2013

SN - 1932-6203

VL - 8

AU - Xu S

AU - Chen G

AU - Chen C

AU - Sun C

AU - Zhang D

AU - Murbach M

AU - Kuster N

AU - Zeng Q

AU - Xu Z

DO - 10.1371/journal.pone.0054906

LA - en

N1 - FEMU ID: 21701; EMF-Portal URL: https://www.emf-portal.org/en/article/21701

SP - e54906

TI - Cell Type-Dependent Induction of DNA Damage by 1800 MHz Radiofrequency Electromagnetic Fields Does Not Result in Significant Cellular Dysfunctions

UR - http://journals.plos.org/plosone/article?id=10.1371/journal.pone.0054906

ER -

TY - JOUR

IS - 1

JA - Toxicol Lett

JO - Toxicology Letters

PY - 2013

SN - 0378-4274

VL - 218

AU - Liu C

AU - Duan W

AU - Xu S

AU - Chen C

AU - He M

AU - Zhang L

AU - Yu Z

AU - Zhou Z

DO - 10.1016/j.toxlet.2013.01.003

LA - en

N1 - FEMU ID: 21674; EMF-Portal URL: https://www.emf-portal.org/en/article/21674

SP - 2-9

TI - Exposure to 1800 MHz radiofrequency electromagnetic radiation induces oxidative DNA base damage in a mouse spermatocyte-derived cell line

ER -

TY - JOUR

IS - 2

JA - Radiat Res

JO - Radiation Research

PY - 2013

SN - 0033-7587

VL - 179

AU - Waldmann P

AU - Bohnenberger S

AU - Greinert R

AU - Hermann-Then B

AU - Heselich A

AU - Klug SJ

AU - Koenig J

AU - Kuhr K

AU - Kuster N

AU - Merker M

AU - Murbach M

AU - Pollet D

AU - Schadenboeck W

AU - Scheidemann-Wesp U

AU - Schwab B

AU - Volkmer B

AU - Weyer V

AU - Blettner M

DO - 10.1667/RR2914.1

LA - en

N1 - FEMU ID: 21641; EMF-Portal URL: https://www.emf-portal.org/en/article/21641

SP - 243-253

TI - Influence of GSM Signals on Human Peripheral Lymphocytes: Study of Genotoxicity

ER -

TY - JOUR

IS - 2

JA - Mutat Res Genet Toxicol Environ Mutagen

JO - Mutation Research - Genetic Toxicology and Environmental Mutagenesis

PY - 2013

VL - 751

AU - Jiang B

AU - Zong C

AU - Zhao H

AU - Ji Y

AU - Tong J

AU - Cao Y

DO - 10.1016/j.mrgentox.2012.12.003

LA - en

N1 - FEMU ID: 21616; EMF-Portal URL: https://www.emf-portal.org/en/article/21616

SP - 127-129

TI - Induction of adaptive response in mice exposed to 900MHz radiofrequency fields: application of micronucleus assay

ER -

TY - JOUR

JA - Behav Brain Res

JO - Behavioural Brain Research

PY - 2013

SN - 0166-4328

VL - 240

AU - Banaceur S

AU - Banasr S

AU - Sakly M

AU - Abdelmelek H

DO - 10.1016/j.bbr.2012.11.021

LA - en

N1 - FEMU ID: 21511; EMF-Portal URL: https://www.emf-portal.org/en/article/21511

SP - 197-201

TI - Whole body exposure to 2.4 GHz WIFI signals: Effects on cognitive impairment in adult triple transgenic mouse models of Alzheimer's disease (3xTg-AD)

ER -

TY - JOUR

JA - Ecotoxicol Environ Saf

JO - Ecotoxicology and Environmental Safety

PY - 2013

SN - 0147-6513

VL - 88

AU - Hekmat A

AU - Saboury AA

AU - Moosavi-Movahedi AA

DO - 10.1016/j.ecoenv.2012.10.016

LA - en

N1 - FEMU ID: 21475; EMF-Portal URL: https://www.emf-portal.org/en/article/21475

SP - 35-41

TI - The toxic effects of mobile phone radiofrequency (940 MHz) on the structure of calf thymus DNA

ER -

TY - JOUR

IS - 3

JA - Int J Radiat Biol

JO - International Journal of Radiation Biology

PY - 2013

SN - 0955-3002

VL - 89

AU - Kumar S

AU - Behari J

AU - Sisodia R

DO - 10.3109/09553002.2013.741282

LA - en

N1 - FEMU ID: 21367; EMF-Portal URL: https://www.emf-portal.org/en/article/21367

SP - 147-154

TI - Influence of electromagnetic fields on reproductive system of male rats

ER -

TY - JOUR

IS - 1-2

JA - Mutat Res Genet Toxicol Environ Mutagen

JO - Mutation Research - Genetic Toxicology and Environmental Mutagenesis

PY - 2013

VL - 750

AU - Pesnya DS

AU - Romanovsky AV

DO - 10.1016/j.mrgentox.2012.08.010

LA - en

N1 - FEMU ID: 21320; EMF-Portal URL: https://www.emf-portal.org/en/article/21320

SP - 27-33

TI - Comparison of cytotoxic and genotoxic effects of plutonium-239 alpha particles and mobile phone GSM 900 radiation in the Allium cepa test

ER -

TY - JOUR

IS - 1

JO - Bioelectromagnetics

PY - 2013

SN - 0197-8462

VL - 34

AU - Bourthoumieu S

AU - Magnaudeix A

AU - Terro F

AU - Leveque P

AU - Collin A

AU - Yardin C

DO - 10.1002/bem.21744

LA - en

N1 - FEMU ID: 20967; EMF-Portal URL: https://www.emf-portal.org/en/article/20967

SP - 52-60

TI - Study of p53 expression and post-transcriptional modifications after GSM-900 radiofrequency exposure of human amniotic cells

ER -

TY - JOUR

IS - 2

JA - J Pediatr Urol

JO - Journal of Pediatric Urology

PY - 2013

SN - 1477-5131

VL - 9

AU - Atasoy HI

AU - Gunal MY

AU - Atasoy P

AU - Elgun S

AU - Bugdayci G

DO - 10.1016/j.jpurol.2012.02.015

LA - en

N1 - FEMU ID: 20439; EMF-Portal URL: https://www.emf-portal.org/en/article/20439

SP - 223-229

TI - Immunohistopathologic demonstration of deleterious effects on growing rat testes of radiofrequency waves emitted from conventional Wi-Fi devices

ER -

TY - JOUR

IS - 4

JA - Nepal Med Coll J

JO - Nepal Medical College Journal

PY - 2012

SN - 2676-1319

VL - 14

AU - Ingole IV

AU - Ghosh SK

LA - en

N1 - FEMU ID: 49582; EMF-Portal URL: https://www.emf-portal.org/en/article/49582

SP - 337-341

TI - Effect of exposure to radio frequency radiation emitted by cell phone on the developing dorsal root ganglion of chick embryo: a light microscopic study

UR - https://nmcth.edu/images/gallery/Original%20Articles/KM56PIV%20Ingole.pdf

ER -

TY - JOUR

JA - Oxid Med Cell Longev

JO - Oxidative Medicine and Cellular Longevity

PY - 2012

SN - 1942-0994

AU - Lu YS

AU - Huang BT

AU - Huang YX

DO - 10.1155/2012/740280

LA - en

N1 - FEMU ID: 20976; EMF-Portal URL: https://www.emf-portal.org/en/article/20976

SP - 740280

TI - Reactive Oxygen Species Formation and Apoptosis in Human Peripheral Blood Mononuclear Cell Induced by 900 MHz Mobile Phone Radiation

UR - https://www.hindawi.com/journals/oximed/2012/740280/

ER -

TY - JOUR

IS - 1

JA - Mutat Res Genet Toxicol Environ Mutagen

JO - Mutation Research - Genetic Toxicology and Environmental Mutagenesis

PY - 2012

VL - 747

AU - Zeni O

AU - Sannino A

AU - Romeo S

AU - Massa R

AU - Sarti M

AU - Reddy AB

AU - Prihoda TJ

AU - Vijayalaxmi

AU - Scarfi MR

DO - 10.1016/j.mrgentox.2012.03.013

LA - en

N1 - FEMU ID: 20662; EMF-Portal URL: https://www.emf-portal.org/en/article/20662

SP - 29-35

TI - Induction of an adaptive response in human blood lymphocytes exposed to radiofrequency fields: Influence of the universal mobile telecommunication system (UMTS) signal and the specific absorption rate

ER -

TY - JOUR

IS - 4

JO - Mutagenesis

PY - 2012

SN - 0267-8357

VL - 27

AU - Hintzsche H

AU - Jastrow C

AU - Kleine-Ostmann T

AU - Schrader T

AU - Stopper H

DO - 10.1093/mutage/ges007

LA - en

N1 - FEMU ID: 20370; EMF-Portal URL: https://www.emf-portal.org/en/article/20370

SP - 477-483

TI - 900 MHz radiation does not induce micronucleus formation in different cell types

UR - https://academic.oup.com/mutage/article-pdf/27/4/477/3889278/ges007.pdf

ER -

TY - JOUR

JA - Ecotoxicol Environ Saf

JO - Ecotoxicology and Environmental Safety

PY - 2012

SN - 0147-6513

VL - 80

AU - Sekeroglu V

AU - Akar A

AU - Sekeroglu ZA

DO - 10.1016/j.ecoenv.2012.02.028

LA - en

N1 - FEMU ID: 20362; EMF-Portal URL: https://www.emf-portal.org/en/article/20362

SP - 140-144

TI - Cytotoxic and genotoxic effects of high-frequency electromagnetic fields (GSM 1800MHz) on immature and mature rats

ER -

TY - JOUR

IS - 2

JO - PLoS One

PY - 2012

SN - 1932-6203

VL - 7

AU - Jiang B

AU - Nie J

AU - Zhou Z

AU - Zhang J

AU - Tong J

AU - Cao Y

DO - 10.1371/journal.pone.0032040

LA - en

N1 - FEMU ID: 20338; EMF-Portal URL: https://www.emf-portal.org/en/article/20338

SP - e32040

TI - Adaptive Response in Mice Exposed to 900 MHz Radiofrequency Fields: Primary DNA Damage

UR - http://journals.plos.org/plosone/article?id=10.1371/journal.pone.0032040

ER -

TY - JOUR

IS - 2

JA - Cell Biochem Biophys

JO - Cell Biochemistry and Biophysics

PY - 2012

SN - 1085-9195

VL - 63

AU - Panagopoulos DJ

DO - 10.1007/s12013-012-9347-0

LA - en

N1 - FEMU ID: 20327; EMF-Portal URL: https://www.emf-portal.org/en/article/20327

SP - 121-132

TI - Effect of microwave exposure on the ovarian development of Drosophila melanogaster

ER -

TY - JOUR

IS - 6

JO - Bioelectromagnetics

PY - 2012

SN - 0197-8462

VL - 33

AU - Zeni O

AU - Sannino A

AU - Sarti M

AU - Romeo S

AU - Massa R

AU - Scarfi MR

DO - 10.1002/bem.21712

LA - en

N1 - FEMU ID: 20288; EMF-Portal URL: https://www.emf-portal.org/en/article/20288

SP - 497-507

TI - Radiofrequency radiation at 1950 MHz (UMTS) does not affect key cellular endpoints in neuron-like PC12 cells

ER -

TY - JOUR

IS - 5

JA - Int J Radiat Biol

JO - International Journal of Radiation Biology

PY - 2012

SN - 0955-3002

VL - 88

AU - Cam ST

AU - Seyhan N

DO - 10.3109/09553002.2012.666005

LA - en

N1 - FEMU ID: 20278; EMF-Portal URL: https://www.emf-portal.org/en/article/20278

SP - 420-424

TI - Single-strand DNA breaks in human hair root cells exposed to mobile phone radiation

ER -

TY - JOUR

IS - 7

JA - Hum Exp Toxicol

JO - Human & Experimental Toxicology

PY - 2012

SN - 0960-3271

VL - 31

AU - Khalil AM

AU - Gagaa MH

AU - Alshamali AM

DO - 10.1177/0960327111433184

LA - en

N1 - FEMU ID: 20105; EMF-Portal URL: https://www.emf-portal.org/en/article/20105

SP - 734-740

TI - 8-Oxo-7, 8-dihydro-2'-deoxyguanosine as a biomarker of DNA damage by mobile phone radiation

ER -

TY - JOUR

IS - 4

JA - Int J Radiat Biol

JO - International Journal of Radiation Biology

PY - 2012

SN - 0955-3002

VL - 88

AU - Guler G

AU - Tomruk A

AU - Ozgur E

AU - Sahin D

AU - Sepici A

AU - Altan N

AU - Seyhan N

DO - 10.3109/09553002.2012.646349

LA - en

N1 - FEMU ID: 19950; EMF-Portal URL: https://www.emf-portal.org/en/article/19950

SP - 367-373

TI - The effect of radiofrequency radiation on DNA and lipid damage in female and male infant rabbits

ER -

TY - JOUR

IS - 2

JA - Br J Cancer

JO - British Journal of Cancer

PY - 2012

SN - 0007-0920

VL - 106

AU - Zimmerman JW

AU - Pennison MJ

AU - Brezovich I

AU - Yi N

AU - Yang CT

AU - Ramaker R

AU - Absher D

AU - Myers RM

AU - Kuster N

AU - Costa FP

AU - Barbault A

AU - Pasche B

DO - 10.1038/bjc.2011.523

LA - en

N1 - FEMU ID: 19937; EMF-Portal URL: https://www.emf-portal.org/en/article/19937

SP - 307-313

TI - Cancer cell proliferation is inhibited by specific modulation frequencies

UR - https://stopsmartmetersirvine.files.wordpress.com/2011/12/zimmerman-et-al-advance-online-publication-1201111.pdf

ER -

TY - JOUR

IS - 1

JA - Fertil Steril

JO - Fertility and Sterility

PY - 2012

SN - 0015-0282

VL - 97

AU - Avendano C

AU - Mata A

AU - Sanchez Sarmiento CA

AU - Doncel GF

DO - 10.1016/j.fertnstert.2011.10.012

LA - en

N1 - FEMU ID: 19930; EMF-Portal URL: https://www.emf-portal.org/en/article/19930

SP - 39-45.e2

TI - Use of laptop computers connected to internet through Wi-Fi decreases human sperm motility and increases sperm DNA fragmentation

ER -

TY - JOUR

IS - 1

JA - J Neurooncol

JO - Journal of Neuro-Oncology

PY - 2012

SN - 0167-594X

VL - 106

AU - Karaca E

AU - Durmaz B

AU - Aktug H

AU - Yildiz T

AU - Guducu C

AU - Irgi M

AU - Koksal MG

AU - Ozkinay F

AU - Gunduz C

AU - Cogulu O

DO - 10.1007/s11060-011-0644-z

LA - en

N1 - FEMU ID: 19410; EMF-Portal URL: https://www.emf-portal.org/en/article/19410

SP - 53-58

TI - The genotoxic effect of radiofrequency waves on mouse brain

ER -

TY - JOUR

IS - 4

JA - Coll Antropol

JO - Collegium Antropologicum

PY - 2011

SN - 0350-6134

VL - 35

AU - Trosic I

AU - Pavicic I

AU - Milkovic-Kraus S

AU - Mladinic M

AU - Zeljezic D

LA - en

N1 - FEMU ID: 20359; EMF-Portal URL: https://www.emf-portal.org/en/article/20359

SP - 1259-1264

TI - Effect of electromagnetic radiofrequency radiation on the rats' brain, liver and kidney cells measured by comet assay

UR - https://hrcak.srce.hr/file/112380

ER -

TY - JOUR

JA - World Acad Sci Eng Technol

JO - World Academy of Science, Engineering and Technology

PY - 2011

VL - 76

AU - Khalil AM

AU - Alshamali AM

AU - Gagaa MH

LA - en

N1 - FEMU ID: 20110; EMF-Portal URL: https://www.emf-portal.org/en/article/20110

SP - 657-622

TI - Detection of oxidative stress induced by mobile phone radiation in tissues of mice using 8-oxo-7, 8-dihydro-20-deoxyguanosine as a biomarker

UR - http://publications.waset.org/7054/pdf

ER -

TY - JOUR

IS - 4

JA - Electromagn Biol Med

JO - Electromagnetic Biology and Medicine

PY - 2011

SN - 1536-8386

VL - 30

AU - Zeng L

AU - Ji X

AU - Zhang Y

AU - Miao X

AU - Zou C

AU - Lang H

AU - Zhang J

AU - Li Y

AU - Wang X

AU - Qi H

AU - Ren D

AU - Guo G

DO - 10.3109/15368378.2011.587929

LA - en

N1 - FEMU ID: 19856; EMF-Portal URL: https://www.emf-portal.org/en/article/19856

SP - 205-218

TI - MnSOD expression inhibited by electromagnetic pulse radiation in the rat testis

ER -

TY - JOUR

JA - Sci Total Environ

JO - Science of the Total Evironment

PY - 2011

SN - 0048-9697

VL - 410

AU - Esmekaya MA

AU - Aytekin E

AU - Ozgur E

AU - Güler G

AU - Ergun MA

AU - Omeroglu S

AU - Seyhan N

DO - 10.1016/j.scitotenv.2011.09.036

LA - en

N1 - FEMU ID: 19800; EMF-Portal URL: https://www.emf-portal.org/en/article/19800

SP - 59-64

TI - Mutagenic and morphologic impacts of 1.8 GHz radiofrequency radiation on human peripheral blood lymphocytes (hPBLs) and possible protective role of pre-treatment with Ginkgo biloba (EGb 761)

ER -

TY - JOUR

JA - Progr Electromagn Res B (PIER B)

JO - Progress in Electromagnetics Research B

PY - 2011

SN - 1937-6472

VL - 29

AU - Chaturvedi CM

AU - Singh VP

AU - Singh P

AU - Basu P

AU - Singaravel M

AU - Shukla RK

AU - Dhawan A

AU - Pati AK

AU - Gangwar RK

AU - Singh SP

DO - 10.2528/PIERB11011205

LA - en

N1 - FEMU ID: 19608; EMF-Portal URL: https://www.emf-portal.org/en/article/19608

SP - 23-42

TI - 2.45 GHz (CW) microwave irradiation alters circadian organization, spatial memory, DNA structure in the brain cells and blood cell counts of male mice, Mus musculus

UR - https://www.jpier.org/ac_api/download.php?id=11011205

ER -

TY - JOUR

JO - Mutation Research - Fundamental and Molecular Mechanism of Mutagenesis

PY - 2011

SN - 0027-5107

VL - 716

AU - Ballardin M

AU - Tusa I

AU - Fontana N

AU - Monorchio A

AU - Pelletti C

AU - Rogovich A

AU - Barale R

AU - Scarpato R

DO - 10.1016/j.mrfmmm.2011.07.009

LA - en

N1 - FEMU ID: 19499; EMF-Portal URL: https://www.emf-portal.org/en/article/19499

SP - 1-9

TI - Non-thermal effects of 2.45GHz microwaves on spindle assembly, mitotic cells and viability of Chinese hamster V-79 cells

ER -

TY - JOUR

IS - 9

JA - Int J Radiat Biol

JO - International Journal of Radiation Biology

PY - 2011

SN - 0955-3002

VL - 87

AU - Sannino A

AU - Zeni O

AU - Sarti M

AU - Romeo S

AU - Reddy SB

AU - Belisario MA

AU - Prihoda TJ

AU - Vijayalaxmi

AU - Scarfi MR

DO - 10.3109/09553002.2011.574779

LA - en

N1 - FEMU ID: 19265; EMF-Portal URL: https://www.emf-portal.org/en/article/19265

SP - 993-999

TI - Induction of adaptive response in human blood lymphocytes exposed to 900 MHz radiofrequency fields: Influence of cell cycle

ER -

TY - JOUR

JO - Neuroscience

PY - 2011

SN - 0306-4522

VL - 185

AU - Dragicevic N

AU - Bradshaw PC

AU - Mamcarz M

AU - Lin X

AU - Wang L

AU - Cao C

AU - Arendash GW

DO - 10.1016/j.neuroscience.2011.04.012

LA - en

N1 - FEMU ID: 19218; EMF-Portal URL: https://www.emf-portal.org/en/article/19218

SP - 135-149

TI - Long-term electromagnetic field treatment enhances brain mitochondrial function of both Alzheimer's transgenic mice and normal mice: a mechanism for electromagnetic field-induced cognitive benefit?

ER -

TY - JOUR

IS - 5

JA - Radiat Res

JO - Radiation Research

PY - 2011

SN - 0033-7587

VL - 175

AU - Hintzsche H

AU - Jastrow C

AU - Kleine-Ostmann T

AU - Stopper H

AU - Schmid E

AU - Schrader T

DO - 10.1667/RR2406.1

LA - en

N1 - FEMU ID: 19081; EMF-Portal URL: https://www.emf-portal.org/en/article/19081

SP - 569-574

TI - Terahertz radiation induces spindle disturbances in human-hamster hybrid cells

ER -

TY - JOUR

IS - 4

JA - Int J Radiat Biol

JO - International Journal of Radiation Biology

PY - 2011

SN - 0955-3002

VL - 87

AU - Bourthoumieu S

AU - Terro F

AU - Leveque P

AU - Collin A

AU - Joubert V

AU - Yardin C

DO - 10.3109/09553002.2011.542543

LA - en

N1 - FEMU ID: 18946; EMF-Portal URL: https://www.emf-portal.org/en/article/18946

SP - 400-408

TI - Aneuploidy studies in human cells exposed in vitro to GSM-900 MHz radiofrequency radiation using FISH

ER -

TY - JOUR

IS - 4

JA - Appl Biochem Biotechnol

JO - Applied Biochemistry and Biotechnology

PY - 2011

SN - 0273-2289

VL - 164

AU - Kesari KK

AU - Kumar S

AU - Behari J

DO - 10.1007/s12010-010-9156-0

LA - en

N1 - FEMU ID: 18931; EMF-Portal URL: https://www.emf-portal.org/en/article/18931

SP - 546-559

TI - Effects of radiofrequency electromagnetic wave exposure from cellular phones on the reproductive pattern in male wistar rats

ER -

TY - JOUR

IS - 4

JO - Bioelectromagnetics

PY - 2011

SN - 0197-8462

VL - 32

AU - Schrader T

AU - Kleine-Ostmann T

AU - Münter K

AU - Jastrow C

AU - Schmid E

DO - 10.1002/bem.20634

LA - en

N1 - FEMU ID: 18854; EMF-Portal URL: https://www.emf-portal.org/en/article/18854

SP - 291-301

TI - Spindle disturbances in human-hamster hybrid (A(L)) cells induced by the electrical component of the mobile communication frequency range signal

ER -

TY - JOUR

IS - 2

JA - Int J Radiat Biol

JO - International Journal of Radiation Biology

PY - 2011

SN - 0955-3002

VL - 87

AU - Kumar G

AU - Wood AW

AU - Anderson V

AU - McIntosh RL

AU - Chen YY

AU - McKenzie RJ

DO - 10.3109/09553002.2010.518212

LA - en

N1 - FEMU ID: 18740; EMF-Portal URL: https://www.emf-portal.org/en/article/18740

SP - 231-240

TI - Evaluation of hematopoietic system effects after in vitro radiofrequency radiation exposure in rats

ER -

TY - JOUR

IS - 1

JA - Int J Hyg Environ Health

JO - International Journal of Hygiene and Environmental Health

PY - 2011

SN - 1438-4639

VL - 214

AU - Garaj-Vrhovac V

AU - Gajski G

AU - Pazanin S

AU - Sarolic A

AU - Domijan AM

AU - Flajs D

AU - Peraica M

DO - 10.1016/j.ijheh.2010.08.003

LA - en

N1 - FEMU ID: 18617; EMF-Portal URL: https://www.emf-portal.org/en/article/18617

SP - 59-65

TI - Assessment of cytogenetic damage and oxidative stress in personnel occupationally exposed to the pulsed microwave radiation of marine radar equipment

ER -

TY - JOUR

IS - 6

JA - Toxicol Environ Chem

JO - Toxicological and Environmental Chemistry

PY - 2010

SN - 0092-9867

VL - 92

AU - Kesari KK

AU - Behari J

DO - 10.1080/02772240903233637

LA - en

N1 - FEMU ID: 28577; EMF-Portal URL: https://www.emf-portal.org/en/article/28577

SP - 1135-1147

TI - Effects of microwave at 2.45 GHz radiations on reproductive system of male rats

ER -

TY - JOUR

IS - 9-10

JA - CR physique

JO - Comptes Rendus Physique

PY - 2010

SN - 1631-0705

VL - 11

AU - Perrin A

AU - Freire M

AU - Bachelet C

AU - Collin A

AU - Leveque P

AU - Pla S

AU - Debouzy JC

DO - 10.1016/j.crhy.2010.10.006

LA - en

N1 - FEMU ID: 19405; EMF-Portal URL: https://www.emf-portal.org/en/article/19405

SP - 613-621

TI - Evaluation of the co-genotoxic effects of 1800 MHz GSM radiofrequency exposure and a chemical mutagen in cultured human cells

ER -

TY - JOUR

IS - 10

JA - J Zhejiang Univ Sci B

JO - Journal of Zhejiang University Science B

PY - 2010

SN - 1673-1581

VL - 11

AU - Shckorbatov YG

AU - Pasiuga VN

AU - Goncharuk EI

AU - Petrenko TP

AU - Grabina VA

AU - Kolchigin NN

AU - Ivanchenko DD

AU - Bykov VN

AU - Dumin OM

DO - 10.1631/jzus.B1000051

LA - en

N1 - FEMU ID: 18648; EMF-Portal URL: https://www.emf-portal.org/en/article/18648

SP - 801-805

TI - Effects of differently polarized microwave radiation on the microscopic structure of the nuclei in human fibroblasts

UR - https://www.ncbi.nlm.nih.gov/pmc/articles/PMC2950243/pdf/JZUSB11-0801.pdf

ER -

TY - JOUR

IS - 6

JA - Radiat Res

JO - Radiation Research

PY - 2010

SN - 0033-7587

VL - 174

AU - Bourthoumieu S

AU - Joubert V

AU - Marin B

AU - Collin A

AU - Leveque P

AU - Terro F

AU - Yardin C

LA - en

N1 - FEMU ID: 18635; EMF-Portal URL: https://www.emf-portal.org/en/article/18635

SP - 712-718

TI - Cytogenetic Studies in Human Cells Exposed In Vitro to GSM-900 MHz Radiofrequency Radiation Using R-Banded Karyotyping

ER -

TY - JOUR

IS - 6

JA - Indian J Exp Biol

JO - Indian Journal of Experimental Biology

PY - 2010

SN - 0019-5189

VL - 48

AU - Kumar S

AU - Kesari KK

AU - Behari J

LA - en

N1 - FEMU ID: 18567; EMF-Portal URL: https://www.emf-portal.org/en/article/18567

SP - 586-592

TI - Evaluation of genotoxic effects in male Wistar rats following microwave exposure

UR - http://nopr.niscpr.res.in/bitstream/123456789/9081/1/IJEB%2048%286%29%20586-592.pdf

ER -

TY - JOUR

IS - 3

JA - Electromagn Biol Med

JO - Electromagnetic Biology and Medicine

PY - 2010

SN - 1536-8386

VL - 29

AU - Gurbuz N

AU - Sirav B

AU - Yuvaci HU

AU - Turhan N

AU - Coskun ZK

AU - Seyhan N

DO - 10.3109/15368378.2010.482498

LA - en

N1 - FEMU ID: 18549; EMF-Portal URL: https://www.emf-portal.org/en/article/18549

SP - 98-104

TI - Is There Any Possible Genotoxic Effect in Exfoliated Bladder Cells of Rat Under the Exposure of 1800 MHz GSM-Like Modulated Radio Frequency Radiation (RFR)?

ER -

TY - JOUR

IS - 2

JA - Genet Couns

JO - Genetic Counseling

PY - 2010

SN - 1015-8146

VL - 21

AU - Yildirim MS

AU - Yildirim A

AU - Zamani AG

AU - Okudan N

LA - en

N1 - FEMU ID: 18526; EMF-Portal URL: https://www.emf-portal.org/en/article/18526

SP - 243-251

TI - Effect of mobile phone station on micronucleus frequency and chromosomal aberrations in human blood cells

ER -

TY - JOUR

IS - 1-2

JA - Mutat Res Genet Toxicol Environ Mutagen

JO - Mutation Research - Genetic Toxicology and Environmental Mutagenesis

PY - 2010

VL - 700

AU - Chavdoula ED

AU - Panagopoulos DJ

AU - Margaritis LH

DO - 10.1016/j.mrgentox.2010.05.008

LA - en

N1 - FEMU ID: 18222; EMF-Portal URL: https://www.emf-portal.org/en/article/18222

SP - 51-61

TI - Comparison of biological effects between continuous and intermittent exposure to GSM-900-MHz mobile phone radiation: Detection of apoptotic cell-death features

ER -

TY - JOUR

IS - 6

JO - Bioelectromagnetics

PY - 2010

SN - 0197-8462

VL - 31

AU - Luukkonen J

AU - Juutilainen J

AU - Naarala J

DO - 10.1002/bem.20580

LA - en

N1 - FEMU ID: 18142; EMF-Portal URL: https://www.emf-portal.org/en/article/18142

SP - 417-424

TI - Combined effects of 872 MHz radiofrequency radiation and ferrous chloride on reactive oxygen species production and DNA damage in human SH-SY5Y neuroblastoma cells

ER -

TY - JOUR

IS - 1-2

JA - Mutat Res Genet Toxicol Environ Mutagen

JO - Mutation Research - Genetic Toxicology and Environmental Mutagenesis

PY - 2010

VL - 699

AU - Panagopoulos DJ

AU - Margaritis LH

DO - 10.1016/j.mrgentox.2010.04.010

LA - en

N1 - FEMU ID: 18120; EMF-Portal URL: https://www.emf-portal.org/en/article/18120

SP - 17-22

TI - The effect of exposure duration on the biological activity of mobile telephony radiation

ER -

TY - JOUR

IS - 1

JA - Gen Physiol Biophys

JO - General Physiology and Biophysics

PY - 2010

SN - 0231-5882

VL - 29

AU - Güler G

AU - Tomruk A

AU - Ozgur E

AU - Seyhan N

DO - 10.4149/gpb_2010_01_59

LA - en

N1 - FEMU ID: 18104; EMF-Portal URL: https://www.emf-portal.org/en/article/18104

SP - 59-66

TI - The effect of radiofrequency radiation on DNA and lipid damage in non-pregnant and pregnant rabbits and their newborns

ER -

TY - JOUR

IS - 4

JA - Int J Radiat Biol

JO - International Journal of Radiation Biology

PY - 2010

SN - 0955-3002

VL - 86

AU - Kesari KK

AU - Behari J

AU - Kumar S

DO - 10.3109/09553000903564059

LA - en

N1 - FEMU ID: 18089; EMF-Portal URL: https://www.emf-portal.org/en/article/18089

SP - 334-343

TI - Mutagenic response of 2.45 GHz radiation exposure on rat brain

ER -

TY - JOUR

IS - 1

JA - Neurosci Lett

JO - Neuroscience Letters

PY - 2010

SN - 0304-3940

VL - 473

AU - Campisi A

AU - Gulino M

AU - Acquaviva R

AU - Bellia P

AU - Raciti G

AU - Grasso R

AU - Musumeci F

AU - Vanella A

AU - Triglia A

DO - 10.1016/j.neulet.2010.02.018

LA - en

N1 - FEMU ID: 17968; EMF-Portal URL: https://www.emf-portal.org/en/article/17968

SP - 52-55

TI - Reactive oxygen species levels and DNA fragmentation on astrocytes in primary culture after acute exposure to low intensity microwave electromagnetic field

ER -

TY - JOUR

IS - 3

JA - Environ Health Perspect

JO - Environmental Health Perspectives

PY - 2010

SN - 0091-6765

VL - 118

AU - Belyaev I

AU - Markova E

AU - Malmgren L

DO - 10.1289/ehp.0900781

LA - en

N1 - FEMU ID: 17858; EMF-Portal URL: https://www.emf-portal.org/en/article/17858

SP - 394-399

TI - Microwaves from Mobile Phones Inhibit 53BP1 Focus Formation in Human Stem Cells Stronger than in Differentiated Cells: Possible Mechanistic Link to Cancer Risk

UR - https://www.ncbi.nlm.nih.gov/pmc/articles/PMC2854769/pdf/ehp-118-394.pdf

ER -

TY - JOUR

IS - 1

JA - Toxicol Lett

JO - Toxicology Letters

PY - 2010

SN - 0378-4274

VL - 193

AU - Hintzsche H

AU - Stopper H

DO - 10.1016/j.toxlet.2009.12.016

LA - en

N1 - FEMU ID: 17832; EMF-Portal URL: https://www.emf-portal.org/en/article/17832

SP - 124-130

TI - Micronucleus frequency in buccal mucosa cells of mobile phone users

ER -

TY - JOUR

JA - Brain Res

JO - Brain Research

PY - 2010

SN - 0006-8993

VL - 1311

AU - Xu S

AU - Zhou Z

AU - Zhang L

AU - Yu Z

AU - Zhang W

AU - Wang Y

AU - Wang X

AU - Li M

AU - Chen Y

AU - Chen C

AU - He M

AU - Zhang G

AU - Zhong M

DO - 10.1016/j.brainres.2009.10.062

LA - en

N1 - FEMU ID: 17674; EMF-Portal URL: https://www.emf-portal.org/en/article/17674

SP - 189-196

TI - Exposure to 1800 MHz radiofrequency radiation induces oxidative damage to mitochondrial DNA in primary cultured neurons

ER -

TY - JOUR

IS - 1

JA - Cell Biochem Biophys

JO - Cell Biochemistry and Biophysics

PY - 2010

SN - 1085-9195

VL - 56

AU - Tomruk A

AU - Güler G

AU - Dincel AS

DO - 10.1007/s12013-009-9068-1

LA - en

N1 - FEMU ID: 17640; EMF-Portal URL: https://www.emf-portal.org/en/article/17640

SP - 39-47

TI - The influence of 1800 MHz GSM-like signals on hepatic oxidative DNA and lipid damage in nonpregnant, pregnant, and newly born rabbits

ER -

TY - JOUR

IS - 1-2

JA - Mutat Res Genet Toxicol Environ Mutagen

JO - Mutation Research - Genetic Toxicology and Environmental Mutagenesis

PY - 2010

VL - 695

AU - Zhijian C

AU - Xiaoxue L

AU - Yezhen L

AU - Shijie C

AU - Lifen J

AU - Jianlin L

AU - Deqiang L

AU - Jiliang H

DO - 10.1016/j.mrgentox.2009.10.001

LA - en

N1 - FEMU ID: 17623; EMF-Portal URL: https://www.emf-portal.org/en/article/17623

SP - 16-21

TI - Impact of 1.8-GHz radiofrequency radiation (RFR) on DNA damage and repair induced by doxorubicin in human B-cell lymphoblastoid cells

ER -

TY - JOUR

IS - 1-2

JO - Mutation Research - Fundamental and Molecular Mechanism of Mutagenesis

PY - 2010

SN - 0027-5107

VL - 683

AU - Franzellitti S

AU - Valbonesi P

AU - Ciancaglini N

AU - Biondi C

AU - Contin A

AU - Bersani F

AU - Fabbri E

DO - 10.1016/j.mrfmmm.2009.10.004

LA - en

N1 - FEMU ID: 17612; EMF-Portal URL: https://www.emf-portal.org/en/article/17612

SP - 35-42

TI - Transient DNA damage induced by high-frequency electromagnetic fields (GSM 1.8GHz) in the human trophoblast HTR-8/SVneo cell line evaluated with the alkaline comet assay

ER -

TY - JOUR

IS - 11

JA - Anticancer Res

JO - Anticancer Research

PY - 2009

SN - 0250-7005

VL - 29

AU - Hansteen IL

AU - Clausen KO

AU - Haugan V

AU - Svendsen M

AU - Svendsen MV

AU - Eriksen JG

AU - Skiaker R

AU - Hauger E

AU - Lageide L

AU - Vistnes AI

AU - Kure EH

LA - en

N1 - FEMU ID: 17824; EMF-Portal URL: https://www.emf-portal.org/en/article/17824

SP - 4323-4330

TI - Cytogenetic effects of exposure to 2.3 GHz radiofrequency radiation on human lymphocytes in vitro

UR - http://ar.iiarjournals.org/content/29/11/4323.full.pdf+html

ER -

TY - JOUR

IS - 2

JA - Int J Toxicol

JO - International Journal of Toxicology

PY - 2009

SN - 1091-5818

VL - 28

AU - Gajski G

AU - Garaj-Vrhovac V

DO - 10.1177/1091581809335051

LA - en

N1 - FEMU ID: 17452; EMF-Portal URL: https://www.emf-portal.org/en/article/17452

SP - 88-98

TI - Radioprotective effects of honeybee venom (Apis mellifera) against 915-MHz microwave radiation-induced DNA damage in wistar rat lymphocytes: in vitro study

ER -

TY - JOUR

IS - 8

JA - Anticancer Res

JO - Anticancer Research

PY - 2009

SN - 0250-7005

VL - 29

AU - Hansteen IL

AU - Lageide L

AU - Clausen KO

AU - Haugan V

AU - Svendsen M

AU - Eriksen JG

AU - Skiaker R

AU - Hauger E

AU - Vistnes AI

AU - Kure EH

LA - en

N1 - FEMU ID: 17426; EMF-Portal URL: https://www.emf-portal.org/en/article/17426

SP - 2885-2892

TI - Cytogenetic effects of 18.0 and 16.5 GHz microwave radiation on human lymphocytes in vitro

UR - http://ar.iiarjournals.org/content/29/8/2885.full.pdf+html

ER -

TY - JOUR

IS - 7

JO - PLoS One

PY - 2009

SN - 1932-6203

VL - 4

AU - De Iuliis GN

AU - Newey RJ

AU - King BV

AU - Aitken RJ

DO - 10.1371/journal.pone.0006446

LA - en

N1 - FEMU ID: 17394; EMF-Portal URL: https://www.emf-portal.org/en/article/17394

SP - e6446

TI - Mobile phone radiation induces reactive oxygen species production and DNA damage in human spermatozoa in vitro

UR - https://journals.plos.org/plosone/article/file?id=10.1371/journal.pone.0006446&type=printable

ER -

TY - JOUR

IS - 1-2

JA - Mutat Res Genet Toxicol Environ Mutagen

JO - Mutation Research - Genetic Toxicology and Environmental Mutagenesis

PY - 2009

VL - 677

AU - Zhijian C

AU - Xiaoxue L

AU - Yezhen L

AU - Deqiang L

AU - Shijie C

AU - Lifen J

AU - Jianlin L

AU - Jiliang H

DO - 10.1016/j.mrgentox.2009.05.015

LA - en

N1 - FEMU ID: 17178; EMF-Portal URL: https://www.emf-portal.org/en/article/17178

SP - 100-104

TI - Influence of 1.8-GHz (GSM) radiofrequency radiation (RFR) on DNA damage and repair induced by X-rays in human leukocytes in vitro

ER -

TY - JOUR

IS - 6

JA - Radiat Res

JO - Radiation Research

PY - 2009

SN - 0033-7587

VL - 171

AU - Sannino A

AU - Di Costanzo G

AU - Brescia F

AU - Sarti M

AU - Zeni O

AU - Juutilainen J

AU - Scarfi MR

DO - 10.1667/RR1642.1

LA - en

N1 - FEMU ID: 17175; EMF-Portal URL: https://www.emf-portal.org/en/article/17175

SP - 743-751

TI - Human fibroblasts and 900 MHz radiofrequency radiation: evaluation of DNA damage after exposure and co-exposure to 3-chloro-4-(dichloromethyl)-5-hydroxy-2(5h)-furanone (MX)

ER -

TY - JOUR

IS - 6

JA - Radiat Res

JO - Radiation Research

PY - 2009

SN - 0033-7587

VL - 171

AU - Sannino A

AU - Sarti M

AU - Reddy SB

AU - Prihoda TJ

AU - Vijayalaxmi

AU - Scarfi MR

DO - 10.1667/RR1687.1

LA - en

N1 - FEMU ID: 17174; EMF-Portal URL: https://www.emf-portal.org/en/article/17174

SP - 735-742

TI - Induction of adaptive response in human blood lymphocytes exposed to radiofrequency radiation

ER -

TY - JOUR

IS - 4

JA - Int J Radiat Biol

JO - International Journal of Radiation Biology

PY - 2009

SN - 0955-3002

VL - 85

AU - Shckorbatov YG

AU - Pasiuga VN

AU - Kolchigin NN

AU - Grabina VA

AU - Batrakov DO

AU - Kalashnikov VV

AU - Ivanchenko DD

AU - Bykov VN

DO - 10.1080/09553000902781113

LA - en

N1 - FEMU ID: 17050; EMF-Portal URL: https://www.emf-portal.org/en/article/17050

SP - 322-329

TI - The influence of differently polarised microwave radiation on chromatin in human cells

ER -

TY - JOUR

IS - 5

JA - Int J Radiat Biol

JO - International Journal of Radiation Biology

PY - 2009

SN - 0955-3002

VL - 85

AU - Ziemann C

AU - Brockmeyer H

AU - Reddy SB

AU - Vijayalaxmi

AU - Prihoda TJ

AU - Kuster N

AU - Tillmann T

AU - Dasenbrock C

DO - 10.1080/09553000902818907

LA - en

N1 - FEMU ID: 17010; EMF-Portal URL: https://www.emf-portal.org/en/article/17010

SP - 454-464

TI - Absence of genotoxic potential of 902 MHz (GSM) and 1747 MHz (DCS) wireless communication signals: In vivo two-year bioassay in B6C3F1 mice

ER -

TY - JOUR

IS - 1-2

JO - Mutation Research - Fundamental and Molecular Mechanism of Mutagenesis

PY - 2009

SN - 0027-5107

VL - 662

AU - Luukkonen J

AU - Hakulinen P

AU - Maki-Paakkanen J

AU - Juutilainen J

AU - Naarala J

DO - 10.1016/j.mrfmmm.2008.12.005

LA - en

N1 - FEMU ID: 16700; EMF-Portal URL: https://www.emf-portal.org/en/article/16700

SP - 54-58

TI - Enhancement of chemically induced reactive oxygen species production and DNA damage in human SH-SY5Y neuroblastoma cells by 872 MHz radiofrequency radiation

ER -

TY - JOUR

IS - 1

JA - Appl Biochem Biotechnol

JO - Applied Biochemistry and Biotechnology

PY - 2009

SN - 0273-2289

VL - 158

AU - Kesari KK

AU - Behari J

DO - 10.1007/s12010-008-8469-8

LA - en

N1 - FEMU ID: 16653; EMF-Portal URL: https://www.emf-portal.org/en/article/16653

SP - 126-139

TI - Fifty-gigahertz microwave exposure effect of radiations on rat brain

ER -

TY - JOUR

IS - 2

JA - Mutat Res Genet Toxicol Environ Mutagen

JO - Mutation Research - Genetic Toxicology and Environmental Mutagenesis

PY - 2009

VL - 672

AU - Tkalec M

AU - Malaric K

AU - Pavlica M

AU - Pevalek-Kozlina B

AU - Vidakovic-Cifrek Z

DO - 10.1016/j.mrgentox.2008.09.022

LA - en

N1 - FEMU ID: 16578; EMF-Portal URL: https://www.emf-portal.org/en/article/16578

SP - 76-81

TI - Effects of radiofrequency electromagnetic fields on seed germination and root meristematic cells of Allium cepa L

ER -

TY - JOUR

IS - 2

JO - Bioelectromagnetics

PY - 2009

SN - 0197-8462

VL - 30

AU - Belyaev IY

AU - Markova E

AU - Hillert L

AU - Malmgren LO

AU - Persson BR

DO - 10.1002/bem.20445

LA - en

N1 - FEMU ID: 16451; EMF-Portal URL: https://www.emf-portal.org/en/article/16451

SP - 129-141

TI - Microwaves from UMTS/GSM mobile phones induce long-lasting inhibition of 53BP1/gamma-H2AX DNA repair foci in human lymphocytes

ER -

TY - JOUR

IS - 4

JA - Fertil Steril

JO - Fertility and Sterility

PY - 2009

SN - 0015-0282

VL - 92

AU - Agarwal A

AU - Desai NR

AU - Makker K

AU - Varghese A

AU - Mouradi R

AU - Sabanegh E

AU - Sharma R

DO - 10.1016/j.fertnstert.2008.08.022

LA - en

N1 - FEMU ID: 16397; EMF-Portal URL: https://www.emf-portal.org/en/article/16397

SP - 1318-1325

TI - Effects of radiofrequency electromagnetic waves (RF-EMW) from cellular phones on human ejaculated semen: an in vitro pilot study

ER -

TY - JOUR

IS - 1

JA - Gene Ther

JO - Gene Therapy

PY - 2009

SN - 0969-7128

VL - 16

AU - Doran TJ

AU - Lu PJ

AU - Vanier GS

AU - Collins MJ

AU - Wu B

AU - Lu QL

DO - 10.1038/gt.2008.144

LA - en

N1 - FEMU ID: 16347; EMF-Portal URL: https://www.emf-portal.org/en/article/16347

SP - 119-126

TI - Microwave irradiation enhances gene and oligonucleotide delivery and induces effective exon skipping in myoblasts

UR - https://www.nature.com/articles/gt2008144.pdf

ER -

TY - JOUR

IS - 4

JA - Electromagn Biol Med

JO - Electromagnetic Biology and Medicine

PY - 2008

SN - 1536-8386

VL - 27

AU - Tiwari R

AU - Lakshmi NK

AU - Surender V

AU - Rajesh AD

AU - Bhargava SC

AU - Ahuja YR

DO - 10.1080/15368370802473554

LA - en

N1 - FEMU ID: 16591; EMF-Portal URL: https://www.emf-portal.org/en/article/16591

SP - 418-425

TI - Combinative Exposure Effect of Radio Frequency Signals from CDMA Mobile Phones and Aphidicolin on DNA Integrity

ER -

TY - JOUR

IS - 11

JA - Int J Radiat Biol

JO - International Journal of Radiation Biology

PY - 2008

SN - 0955-3002

VL - 84

AU - Huang TQ

AU - Lee MS

AU - Oh EH

AU - Kalinec F

AU - Zhang BT

AU - Seo JS

AU - Park WY

DO - 10.1080/09553000802460123

LA - en

N1 - FEMU ID: 16546; EMF-Portal URL: https://www.emf-portal.org/en/article/16546

SP - 909-915

TI - Characterization of biological effect of 1763 MHz radiofrequency exposure on auditory hair cells

ER -

TY - JOUR

IS - 9

JA - Int J Radiat Biol

JO - International Journal of Radiation Biology

PY - 2008

SN - 0955-3002

VL - 84

AU - Huang TQ

AU - Lee MS

AU - Oh E

AU - Zhang BT

AU - Seo JS

AU - Park WY

DO - 10.1080/09553000802317760

LA - en

N1 - FEMU ID: 16424; EMF-Portal URL: https://www.emf-portal.org/en/article/16424

SP - 734-741

TI - Molecular responses of Jurkat T-cells to 1763 MHz radiofrequency radiation

ER -

TY - JOUR

IS - 2

JA - Radiat Res

JO - Radiation Research

PY - 2008

SN - 0033-7587

VL - 170

AU - Korenstein-Ilan A

AU - Barbul A

AU - Hasin P

AU - Eliran A

AU - Gover A

AU - Korenstein R

DO - 10.1667/RR0944.1

LA - en

N1 - FEMU ID: 16198; EMF-Portal URL: https://www.emf-portal.org/en/article/16198

SP - 224-234

TI - Terahertz radiation increases genomic instability in human lymphocytes

ER -

TY - JOUR

IS - 8

JO - Bioelectromagnetics

PY - 2008

SN - 0197-8462

VL - 29

AU - Schrader T

AU - Münter K

AU - Kleine-Ostmann T

AU - Schmid E

DO - 10.1002/bem.20428

LA - en

N1 - FEMU ID: 16004; EMF-Portal URL: https://www.emf-portal.org/en/article/16004

SP - 626-639

TI - Spindle disturbances in human-hamster hybrid (AL) cells induced by mobile communication frequency range signals

ER -

TY - JOUR

JA - Mol Vis

JO - Molecular Vision

PY - 2008

SN - 1090-0535

VL - 14

AU - Yao K

AU - Wu W

AU - Wang K

AU - Ni S

AU - Ye P

AU - Yu Y

AU - Ye J

AU - Sun L

LA - en

N1 - FEMU ID: 15998; EMF-Portal URL: https://www.emf-portal.org/en/article/15998

SP - 964-969

TI - Electromagnetic noise inhibits radiofrequency radiation-induced DNA damage and reactive oxygen species increase in human lens epithelial cells

UR - https://www.ncbi.nlm.nih.gov/pmc/articles/PMC2391079/pdf/mv-v14-964.pdf

ER -

TY - JOUR

IS - 5

JA - Radiat Res

JO - Radiation Research

PY - 2008

SN - 0033-7587

VL - 169

AU - Manti L

AU - Braselmann H

AU - Calabrese ML

AU - Massa R

AU - Pugliese M

AU - Scampoli P

AU - Sicignano G

AU - Grossi G

DO - 10.1667/RR1044.1

LA - en

N1 - FEMU ID: 15911; EMF-Portal URL: https://www.emf-portal.org/en/article/15911

SP - 575-583

TI - Effects of modulated microwave radiation at cellular telephone frequency (1.95 GHz) on X-ray-induced chromosome aberrations in human lymphocytes in vitro

ER -

TY - JOUR

IS - 3

JA - Radiat Res

JO - Radiation Research

PY - 2008

SN - 0033-7587

VL - 169

AU - Valbonesi P

AU - Franzellitti S

AU - Piano A

AU - Contin A

AU - Biondi C

AU - Fabbri E

DO - 10.1667/RR1061.1

LA - en

N1 - FEMU ID: 15699; EMF-Portal URL: https://www.emf-portal.org/en/article/15699

SP - 270-279

TI - Evaluation of HSP70 expression and DNA damage in cells of a human trophoblast cell line exposed to 1.8 GHz amplitude-modulated radiofrequency fields

ER -

TY - JOUR

IS - 6

JA - Int Arch Occup Environ Health

JO - International Archives of Occupational and Environmental Health

PY - 2008

SN - 0340-0131

VL - 81

AU - Schwarz C

AU - Kratochvil E

AU - Pilger A

AU - Kuster N

AU - Adlkofer F

AU - Rudiger HW

DO - 10.1007/s00420-008-0305-5

LA - en

N1 - FEMU ID: 15682; EMF-Portal URL: https://www.emf-portal.org/en/article/15682

SP - 755-767

TI - Radiofrequency electromagnetic fields (UMTS, 1,950 MHz) induce genotoxic effects in vitro in human fibroblasts but not in lymphocytes

ER -

TY - JOUR

IS - 3

JA - Environ Toxicol

JO - Environmental Toxicology

PY - 2008

SN - 1520-4081

VL - 23

AU - Kim JY

AU - Hong SY

AU - Lee YM

AU - Yu SA

AU - Koh WS

AU - Hong JR

AU - Son T

AU - Chang SK

AU - Lee M

DO - 10.1002/tox.20347

LA - en

N1 - FEMU ID: 15600; EMF-Portal URL: https://www.emf-portal.org/en/article/15600

SP - 319-327

TI - In vitro assessment of clastogenicity of mobile-phone radiation (835 MHz) using the alkaline comet assay and chromosomal aberration test

ER -

TY - JOUR

IS - 2

JA - Mutat Res Genet Toxicol Environ Mutagen

JO - Mutation Research - Genetic Toxicology and Environmental Mutagenesis

PY - 2008

VL - 650

AU - Yadav AS

AU - Sharma MK

DO - 10.1016/j.mrgentox.2007.11.005

LA - en

N1 - FEMU ID: 15588; EMF-Portal URL: https://www.emf-portal.org/en/article/15588

SP - 175-180

TI - Increased frequency of micronucleated exfoliated cells among humans exposed in vivo to mobile telephone radiations

ER -

TY - JOUR

IS - 1

JA - Radiat Res

JO - Radiation Research

PY - 2008

SN - 0033-7587

VL - 169

AU - Mazor R

AU - Korenstein-Ilan A

AU - Barbul A

AU - Eshet Y

AU - Shahadi A

AU - Jerby E

AU - Korenstein R

DO - 10.1667/RR0872.1

LA - en

N1 - FEMU ID: 15530; EMF-Portal URL: https://www.emf-portal.org/en/article/15530

SP - 28-37

TI - Increased levels of numerical chromosome aberrations after in vitro exposure of human peripheral blood lymphocytes to radiofrequency electromagnetic fields for 72 hours

ER -

TY - JOUR

IS - 1

JA - Neurosci Lett

JO - Neuroscience Letters

PY - 2008

SN - 0304-3940

VL - 432

AU - Inoue S

AU - Motoda H

AU - Koike Y

AU - Kawamura K

AU - Hiragami F

AU - Kano Y

DO - 10.1016/j.neulet.2007.12.002

LA - en

N1 - FEMU ID: 15527; EMF-Portal URL: https://www.emf-portal.org/en/article/15527

SP - 35-39

TI - Microwave irradiation induces neurite outgrowth in PC12m3 cells via the p38 mitogen-activated protein kinase pathway

ER -

TY - JOUR

IS - 3

JO - Bioelectromagnetics

PY - 2008

SN - 0197-8462

VL - 29

AU - Zeni O

AU - Schiavoni A

AU - Perrotta A

AU - Forigo D

AU - Deplano M

AU - Scarfi MR

DO - 10.1002/bem.20378

LA - en

N1 - FEMU ID: 15373; EMF-Portal URL: https://www.emf-portal.org/en/article/15373

SP - 177-184

TI - Evaluation of genotoxic effects in human leukocytes after in vitro exposure to 1950 MHz UMTS radiofrequency field

ER -

TY - JOUR

IS - 1-2

JA - Mutat Res Genet Toxicol Environ Mutagen

JO - Mutation Research - Genetic Toxicology and Environmental Mutagenesis

PY - 2008

VL - 649

AU - Hruby R

AU - Neubauer G

AU - Kuster N

AU - Frauscher M

DO - 10.1016/j.mrgentox.2007.07.016

LA - en

N1 - FEMU ID: 15331; EMF-Portal URL: https://www.emf-portal.org/en/article/15331

SP - 34-44

TI - Study on potential effects of "902-MHz GSM-type Wireless Communication Signals" on DMBA-induced mammary tumours in Sprague-Dawley rats

ER -

TY - JOUR

IS - 3

JA - Neuroquantology

JO - NeuroQuantology: An Interdisciplinary Journal of Neuroscience and Quantum Physics

PY - 2007

SN - 1303-5150

VL - 5

AU - Barcal J

AU - Vozeh F

DO - 10.14704/nq.2007.5.3.138

LA - en

N1 - FEMU ID: 17209; EMF-Portal URL: https://www.emf-portal.org/en/article/17209

SP - 292-303

TI - Effect of whole-body exposure to high-frequency electromagnetic field on the brain cortical and hippocampal activity in mouse experimental model

UR - https://www.researchgate.net/publication/287860546_Effect_of_Whole-Body_Exposure_to_High-Frequency_Electromagnetic_Field_on_the_Brain_Cortical_and_Hippocampal_Activity_in_Mouse_Experimental_Model

ER -

TY - JOUR

IS - 1-2

JA - Acupunct Electrother Res

JO - Acupuncture and Electro-Therapeutics Research

PY - 2007

SN - 0360-1293

VL - 32

AU - Syldona M

DO - 10.3727/036012907815844138

LA - en

N1 - FEMU ID: 15490; EMF-Portal URL: https://www.emf-portal.org/en/article/15490

SP - 1-14

TI - Reducing the in-vitro electromagnetic field effect of cellular phones on human DNA and the intensity of their emitted radiation

ER -

TY - JOUR

IS - 4

JA - Fertil Steril

JO - Fertility and Sterility

PY - 2007

SN - 0015-0282

VL - 88

AU - Yan JG

AU - Agresti M

AU - Bruce T

AU - Yan YH

AU - Granlund A

AU - Matloub HS

DO - 10.1016/j.fertnstert.2006.12.022

LA - en

N1 - FEMU ID: 14886; EMF-Portal URL: https://www.emf-portal.org/en/article/14886

SP - 957-964

TI - Effects of cellular phone emissions on sperm motility in rats

ER -

TY - JOUR

IS - 4

JA - Int J Radiat Biol

JO - International Journal of Radiation Biology

PY - 2007

SN - 0955-3002

VL - 83

AU - Juutilainen J

AU - Heikkinen P

AU - Soikkeli H

AU - Maki-Paakkanen J

DO - 10.1080/09553000601169800

LA - en

N1 - FEMU ID: 14790; EMF-Portal URL: https://www.emf-portal.org/en/article/14790

SP - 213-220

TI - Micronucleus frequency in erythrocytes of mice after long-term exposure to radiofrequency radiation

ER -

TY - JOUR

IS - 3

JO - Toxicology

PY - 2007

SN - 0300-483X

VL - 232

AU - Baohong W

AU - Lifen J

AU - Lanjuan L

AU - Jianlin L

AU - Deqiang L

AU - Wei Z

AU - Jiliang H

DO - 10.1016/j.tox.2007.01.019

LA - en

N1 - FEMU ID: 14600; EMF-Portal URL: https://www.emf-portal.org/en/article/14600

SP - 311-316

TI - Evaluating the combinative effects on human lymphocyte DNA damage induced by ultraviolet ray C plus 1.8 GHz microwaves using comet assay in vitro

ER -

TY - JOUR

IS - 3

JA - Radiat Res

JO - Radiation Research

PY - 2007

SN - 0033-7587

VL - 167

AU - Zeni O

AU - Di Pietro R

AU - d'Ambrosio G

AU - Massa R

AU - Capri M

AU - Naarala J

AU - Juutilainen J

AU - Scarfi MR

DO - 10.1667/RR0595.1

LA - en

N1 - FEMU ID: 14552; EMF-Portal URL: https://www.emf-portal.org/en/article/14552

SP - 306-311

TI - Formation of reactive oxygen species in L929 cells after exposure to 900 MHz RF radiation with and without co-exposure to 3-chloro-4-(dichloromethyl)-5-hydroxy-2(5H)-furanone

ER -

TY - JOUR

IS - 1

JA - J Radiat Res

JO - Journal of Radiation Research

PY - 2007

SN - 0449-3060

VL - 48

AU - Koyama S

AU - Takashima Y

AU - Sakurai T

AU - Suzuki Y

AU - Taki M

AU - Miyakoshi J

DO - 10.1269/jrr.06085

LA - en

N1 - FEMU ID: 14407; EMF-Portal URL: https://www.emf-portal.org/en/article/14407

SP - 69-75

TI - Effects of 2.45 GHz electromagnetic fields with a wide range of SARs on bacterial and HPRT gene mutations

UR - https://www.jstage.jst.go.jp/article/jrr/48/1/48_1_69/_article

ER -

TY - JOUR

IS - 1-2

JA - Mutat Res Genet Toxicol Environ Mutagen

JO - Mutation Research - Genetic Toxicology and Environmental Mutagenesis

PY - 2007

VL - 626

AU - Panagopoulos DJ

AU - Chavdoula ED

AU - Nezis IP

AU - Margaritis LH

DO - 10.1016/j.mrgentox.2006.08.008

LA - en

N1 - FEMU ID: 14278; EMF-Portal URL: https://www.emf-portal.org/en/article/14278

SP - 69-78

TI - Cell death induced by GSM 900-MHz and DCS 1800-MHz mobile telephony radiation

ER -

TY - JOUR

IS - 1-2

JA - Mutat Res Genet Toxicol Environ Mutagen

JO - Mutation Research - Genetic Toxicology and Environmental Mutagenesis

PY - 2007

VL - 626

AU - Speit G

AU - Schütz P

AU - Hoffmann H

DO - 10.1016/j.mrgentox.2006.08.003

LA - en

N1 - FEMU ID: 14202; EMF-Portal URL: https://www.emf-portal.org/en/article/14202

SP - 42-47

TI - Genotoxic effects of exposure to radiofrequency electromagnetic fields (RF-EMF) in cultured mammalian cells are not independently reproducible

ER -

TY - JOUR

IS - 2

JA - Def Sci J

JO - Defence Science Journal

PY - 2006

SN - 0011-748X

VL - 56

AU - Sarkar S

AU - Kumari B

AU - Ali S

DO - 10.14429/dsj.56.1883

LA - en

N1 - FEMU ID: 50157; EMF-Portal URL: https://www.emf-portal.org/en/article/50157

SP - 199-208

TI - Effect of radiofrequency electromagnetic field on human DNA

UR - https://publications.drdo.gov.in/ojs/index.php/dsj/article/view/1883/925

ER -

TY - JOUR

IS - 1-2

JO - Mutation Research - Fundamental and Molecular Mechanism of Mutagenesis

PY - 2006

SN - 0027-5107

VL - 596

AU - Paulraj R

AU - Behari J

DO - 10.1016/j.mrfmmm.2005.12.006

LA - en

N1 - FEMU ID: 18097; EMF-Portal URL: https://www.emf-portal.org/en/article/18097

SP - 76-80

TI - Single strand DNA breaks in rat brain cells exposed to microwave radiation

ER -

TY - JOUR

IS - 4

JA - IEEE Trans Plasma Sci

JO - IEEE Transactions on Plasma Science

PY - 2006

SN - 0093-3813

VL - 34

AU - Sannino A

AU - Calabrese ML

AU - d'Ambrosio G

AU - Massa R

AU - Petraglia G

AU - Mita P

AU - Sarti M

AU - Scarfi MR

DO - 10.1109/TPS.2006.878379

LA - en

N1 - FEMU ID: 15411; EMF-Portal URL: https://www.emf-portal.org/en/article/15411

SP - 1441-1448

TI - Evaluation of Cytotoxic and Genotoxic Effects in Human Peripheral Blood Leukocytes Following Exposure to 1950-MHz Modulated Signal

ER -

TY - JOUR

IS - 2

JA - Bull Exp Biol Med

JO - Bulletin of Experimental Biology and Medicine

PY - 2006

SN - 0007-4888

VL - 142

AU - Subbotina TI

AU - Tereshkina OV

AU - Khadartsev AA

AU - Yashin AA

DO - 10.1007/s10517-006-0324-8

LA - en

N1 - FEMU ID: 14609; EMF-Portal URL: https://www.emf-portal.org/en/article/14609

SP - 189-190

TI - Effect of low-intensity extremely high frequency radiation on reproductive function in wistar rats

ER -

TY - JOUR

IS - 1-2

JO - Mutation Research - Fundamental and Molecular Mechanism of Mutagenesis

PY - 2006

SN - 0027-5107

VL - 602

AU - Lixia S

AU - Yao K

AU - Kaijun W

AU - Deqiang L

AU - Huajun H

AU - Xiangwei G

AU - Baohong W

AU - Wei Z

AU - Jianling L

AU - Wei W

DO - 10.1016/j.mrfmmm.2006.08.010

LA - en

N1 - FEMU ID: 14262; EMF-Portal URL: https://www.emf-portal.org/en/article/14262

SP - 135-142

TI - Effects of 1.8 GHz radiofrequency field on DNA damage and expression of heat shock protein 70 in human lens epithelial cells

ER -

TY - JOUR

IS - 1

JA - Life Sci

JO - Life Sciences

PY - 2006

SN - 0024-3205

VL - 80

AU - Ferreira AR

AU - Knakievicz T

AU - Pasquali MA

AU - Gelain DP

AU - Dal-Pizzol F

AU - Fernandez CE

AU - de Salles AA

AU - Ferreira HB

AU - Moreira JC

DO - 10.1016/j.lfs.2006.08.018

LA - en

N1 - FEMU ID: 14184; EMF-Portal URL: https://www.emf-portal.org/en/article/14184

SP - 43-50

TI - Ultra high frequency-electromagnetic field irradiation during pregnancy leads to an increase in erythrocytes micronuclei incidence in rat offspring

ER -

TY - JOUR

IS - 3

JA - Radiat Res

JO - Radiation Research

PY - 2006

SN - 0033-7587

VL - 166

AU - Vijayalaxmi

DO - 10.1667/RR0643.1

LA - en

N1 - FEMU ID: 14138; EMF-Portal URL: https://www.emf-portal.org/en/article/14138

SP - 532-538

TI - Cytogenetic studies in human blood lymphocytes exposed in vitro to 2.45 GHz or 8.2 GHz radiofrequency radiation

ER -

TY - JOUR

IS - 2

JA - Radiat Res

JO - Radiation Research

PY - 2006

SN - 0033-7587

VL - 166

AU - Heikkinen P

AU - Ernst H

AU - Huuskonen H

AU - Komulainen H

AU - Kumlin T

AU - Maki-Paakkanen J

AU - Puranen L

AU - Juutilainen J

DO - 10.1667/RR3588.1

LA - en

N1 - FEMU ID: 14055; EMF-Portal URL: https://www.emf-portal.org/en/article/14055

SP - 397-408

TI - No effects of radiofrequency radiation on 3-chloro-4-(dichloromethyl)-5-hydroxy-2(5H)-furanone-induced tumorigenesis in female Wistar rats

ER -

TY - JOUR

IS - 5

JA - Int J Radiat Biol

JO - International Journal of Radiation Biology

PY - 2006

SN - 0955-3002

VL - 82

AU - Stronati L

AU - Testa A

AU - Moquet J

AU - Edwards A

AU - Cordelli E

AU - Villani P

AU - Marino C

AU - Fresegna AM

AU - Appolloni M

AU - Lloyd D

DO - 10.1080/09553000600739173

LA - en

N1 - FEMU ID: 13927; EMF-Portal URL: https://www.emf-portal.org/en/article/13927

SP - 339-346

TI - 935 MHz cellular phone radiation. An in vitro study of genotoxicity in human lymphocytes

ER -

TY - JOUR

IS - 6

JA - Radiat Res

JO - Radiation Research

PY - 2006

SN - 0033-7587

VL - 165

AU - Scarfi MR

AU - Fresegna AM

AU - Villani P

AU - Pinto R

AU - Marino C

AU - Sarti M

AU - Altavista P

AU - Sannino A

AU - Lovisolo GA

DO - 10.1667/RR3570.1

LA - en

N1 - FEMU ID: 13901; EMF-Portal URL: https://www.emf-portal.org/en/article/13901

SP - 655-663

TI - Exposure to radiofrequency radiation (900 MHz, GSM signal) does not affect micronucleus frequency and cell proliferation in human peripheral blood lymphocytes: an interlaboratory study

ER -

TY - JOUR

IS - 6

JA - Radiat Res

JO - Radiation Research

PY - 2006

SN - 0033-7587

VL - 165

AU - Qutob SS

AU - Chauhan V

AU - Bellier PV

AU - Yauk CL

AU - Douglas GR

AU - Berndt L

AU - Williams A

AU - Gajda GB

AU - Lemay E

AU - Thansandote A

AU - McNamee JP

DO - 10.1667/RR3561.1

LA - en

N1 - FEMU ID: 13899; EMF-Portal URL: https://www.emf-portal.org/en/article/13899

SP - 636-644

TI - Microarray gene expression profiling of a human glioblastoma cell line exposed in vitro to a 1.9 GHz pulse-modulated radiofrequency field

ER -

TY - JOUR

IS - 5

JA - Radiat Res

JO - Radiation Research

PY - 2006

SN - 0033-7587

VL - 165

AU - Verschaeve L

AU - Heikkinen P

AU - Verheyen G

AU - Van Gorp U

AU - Boonen F

AU - Vander Plaetse F

AU - Maes A

AU - Kumlin T

AU - Maki-Paakkanen J

AU - Puranen L

AU - Juutilainen J

DO - 10.1667/RR3559.1

LA - en

N1 - FEMU ID: 13792; EMF-Portal URL: https://www.emf-portal.org/en/article/13792

SP - 598-607

TI - Investigation of co-genotoxic effects of radiofrequency electromagnetic fields in vivo

ER -

TY - JOUR

IS - 4

JO - Bioelectromagnetics

PY - 2006

SN - 0197-8462

VL - 27

AU - Belyaev IY

AU - Koch CB

AU - Terenius O

AU - Röxstrom-Lindquist K

AU - Malmgren LO

AU - Sommer WH

AU - Salford LG

AU - Persson BR

DO - 10.1002/bem.20216

LA - en

N1 - FEMU ID: 13430; EMF-Portal URL: https://www.emf-portal.org/en/article/13430

SP - 295-306

TI - Exposure of rat brain to 915 MHz GSM microwaves induces changes in gene expression but not double stranded DNA breaks or effects on chromatin conformation

ER -

TY - JOUR

IS - 2

JO - Mutagenesis

PY - 2006

SN - 0267-8357

VL - 21

AU - Maes A

AU - Van Gorp U

AU - Verschaeve L

DO - 10.1093/mutage/gel008

LA - en

N1 - FEMU ID: 13387; EMF-Portal URL: https://www.emf-portal.org/en/article/13387

SP - 139-142

TI - Cytogenetic investigation of subjects professionally exposed to radiofrequency radiation

UR - https://academic.oup.com/mutage/article-pdf/21/2/139/3906515/gel008.pdf

ER -

TY - JOUR

IS - 3

JO - Bioelectromagnetics

PY - 2006

SN - 0197-8462

VL - 27

AU - Chemeris NK

AU - Gapeyev AB

AU - Sirota NP

AU - Gudkova OY

AU - Tankanag AV

AU - Konovalov IV

AU - Buzoverya ME

AU - Suvorov VG

AU - Logunov VA

DO - 10.1002/bem.20196

LA - en

N1 - FEMU ID: 12886; EMF-Portal URL: https://www.emf-portal.org/en/article/12886

SP - 197-203

TI - Lack of direct DNA damage in human blood leukocytes and lymphocytes after in vitro exposure to high power microwave pulses

ER -

TY - JOUR

IS - 1

JO - Bioelectromagnetics

PY - 2006

SN - 0197-8462

VL - 27

AU - Sakuma N

AU - Komatsubara Y

AU - Takeda H

AU - Hirose H

AU - Sekijima M

AU - Nojima T

AU - Miyakoshi J

DO - 10.1002/bem.20179

LA - en

N1 - FEMU ID: 12875; EMF-Portal URL: https://www.emf-portal.org/en/article/12875

SP - 51-57

TI - DNA strand breaks are not induced in human cells exposed to 2.1425 GHz band CW and W-CDMA modulated radiofrequency fields allocated to mobile radio base stations

ER -

TY - JOUR

IS - 5

JA - Rev Sci Instrum

JO - Review of Scientific Instruments

PY - 2005

SN - 0034-6748

VL - 76

AU - Belloni F

AU - Nassisi V

AU - Alifano P

AU - Monaco C

AU - Tala A

AU - Tredici M

AU - Raino A

LA - en

N1 - FEMU ID: 16862; EMF-Portal URL: https://www.emf-portal.org/en/article/16862

SP - k.A

TI - A suitable plane transmission line at 900 MHz rf fields for E. coli DNA studies

ER -

TY - JOUR

IS - 4

JA - Int J Hum Genet

JO - International Journal of Human Genetics

PY - 2005

SN - 0972-3757

VL - 5

AU - Gandhi G

AU - Singh P

LA - en

N1 - FEMU ID: 16802; EMF-Portal URL: https://www.emf-portal.org/en/article/16802

SP - 259-265

TI - Cytogenetic damage in mobile phone users: preliminary data

UR - http://www.krepublishers.com/02-Journals/IJHG/IJHG-05-0-000-000-2005-Web/IJHG-05-4-225-288-2005-Abst-PDF/IJHG-05-4-259-265-2005-210-Gandhi-G/IJHG-05-4-259-265-2005-210-Gandhi-G.pdf

ER -

TY - JOUR

IS - 2

JA - Indian J Hum Genet

JO - Indian Journal of Human Genetics

PY - 2005

SN - 1998-362X

VL - 11

AU - Gandhi G

AU - Anita

DO - 10.4103/0971-6866.16810

LA - en

N1 - FEMU ID: 16801; EMF-Portal URL: https://www.emf-portal.org/en/article/16801

SP - 99-104

TI - Genetic damage in mobile phone users: some preliminary findings

UR - http://www.bioline.org.br/pdf?hg05022

ER -

TY - JOUR

IS - 12

JA - Int J Radiat Biol

JO - International Journal of Radiation Biology

PY - 2005

SN - 0955-3002

VL - 81

AU - Huang TQ

AU - Lee JS

AU - Kim TH

AU - Pack JK

AU - Jang JJ

AU - Seo JS

DO - 10.1080/09553000600568093

LA - en

N1 - FEMU ID: 13546; EMF-Portal URL: https://www.emf-portal.org/en/article/13546

SP - 861-867

TI - Effect of radiofrequency radiation exposure on mouse skin tumorigenesis initiated by 7,12-dimethybenz[alpha]anthracene

ER -

TY - JOUR

IS - 1-2

JA - Mutat Res Genet Toxicol Environ Mutagen

JO - Mutation Research - Genetic Toxicology and Environmental Mutagenesis

PY - 2005

VL - 587

AU - Komatsubara Y

AU - Hirose H

AU - Sakurai T

AU - Koyama S

AU - Suzuki Y

AU - Taki M

AU - Miyakoshi J

DO - 10.1016/j.mrgentox.2005.08.010

LA - en

N1 - FEMU ID: 12641; EMF-Portal URL: https://www.emf-portal.org/en/article/12641

SP - 114-119

TI - Effect of high-frequency electromagnetic fields with a wide range of SARs on chromosomal aberrations in murine m5S cells

ER -

TY - JOUR

IS - 4

JA - Radiat Res

JO - Radiation Research

PY - 2005

SN - 0033-7587

VL - 164

AU - Gorlitz BD

AU - Muller M

AU - Ebert S

AU - Hecker H

AU - Kuster N

AU - Dasenbrock C

DO - 10.1667/rr3440.1

LA - en

N1 - FEMU ID: 12605; EMF-Portal URL: https://www.emf-portal.org/en/article/12605

SP - 431-439

TI - Effects of 1-week and 6-week exposure to GSM/DCS radiofrequency radiation on micronucleus formation in B6C3F1 mice

ER -

TY - JOUR

IS - 9

JA - Environ Health Perspect

JO - Environmental Health Perspectives

PY - 2005

SN - 0091-6765

VL - 113

AU - Markova E

AU - Hillert L

AU - Malmgren L

AU - Persson BR

AU - Belyaev IY

DO - 10.1289/ehp.7561

LA - en

N1 - FEMU ID: 12441; EMF-Portal URL: https://www.emf-portal.org/en/article/12441

SP - 1172-1177

TI - Microwaves from GSM mobile telephones affect 53BP1 and gamma-H2AX foci in human lymphocytes from hypersensitive and healthy persons

UR - https://www.ncbi.nlm.nih.gov/pmc/articles/PMC1280397/pdf/ehp0113-001172.pdf

ER -

TY - JOUR

IS - 12

JA - FASEB J

JO - The FASEB Journal

PY - 2005

SN - 0892-6638

VL - 19

AU - Nikolova T

AU - Czyz J

AU - Rolletschek A

AU - Blyszczuk P

AU - Fuchs J

AU - Jovtchev G

AU - Schuderer J

AU - Kuster N

AU - Wobus AM

DO - 10.1096/fj.04-3549fje

LA - en

N1 - FEMU ID: 12365; EMF-Portal URL: https://www.emf-portal.org/en/article/12365

SP - 1686-1688

TI - Electromagnetic fields affect transcript levels of apoptosis-related genes in embryonic stem cell-derived neural progenitor cells

ER -

TY - JOUR

IS - 2

JA - Bull Exp Biol Med

JO - Bulletin of Experimental Biology and Medicine

PY - 2005

SN - 0007-4888

VL - 139

AU - Ivanov VB

AU - Subbotina TI

AU - Khadartsev AA

AU - Yashin MA

AU - Yashin AA

DO - 10.1007/s10517-005-0259-5

LA - en

N1 - FEMU ID: 12271; EMF-Portal URL: https://www.emf-portal.org/en/article/12271

SP - 241-244

TI - Exposure to low-intensive superhigh frequency electromagnetic field as a factor of carcinogenesis in experimental animals

ER -

TY - JOUR

IS - 1

JA - Prague Med Rep

JO - Prague Medical Report

PY - 2005

SN - 1214-6994

VL - 106

AU - Barcal J

AU - Cendelin J

AU - Vozeh F

AU - Zalud V

LA - en

N1 - FEMU ID: 12229; EMF-Portal URL: https://www.emf-portal.org/en/article/12229

SP - 91-100

TI - Effect of whole-body exposure to high-frequency electromagnetic field on the brain electrogeny in neurodefective and healthy mice

UR - https://pmr.cuni.cz/Data/files/PragueMedicalReport/PMR%2005-01%20Barcal.pdf

ER -

TY - JOUR

IS - 1-2

JA - Mutat Res Genet Toxicol Environ Mutagen

JO - Mutation Research - Genetic Toxicology and Environmental Mutagenesis

PY - 2005

VL - 582

AU - Zotti-Martelli L

AU - Peccatori M

AU - Maggini V

AU - Ballardin M

AU - Barale R

DO - 10.1016/j.mrgentox.2004.12.014

LA - en

N1 - FEMU ID: 12152; EMF-Portal URL: https://www.emf-portal.org/en/article/12152

SP - 42-52

TI - Individual responsiveness to induction of micronuclei in human lymphocytes after exposure in vitro to 1800 MHz microwave radiation

ER -

TY - JOUR

IS - 1

JA - Electromagn Biol Med

JO - Electromagnetic Biology and Medicine

PY - 2005

SN - 1536-8386

VL - 24

AU - Lai H

AU - Singh NP

LA - en

N1 - FEMU ID: 12077; EMF-Portal URL: https://www.emf-portal.org/en/article/12077

SP - 23-29

TI - Interaction of Microwaves and a Temporally Incoherent Magnetic Field on Single and Double DNA Strand Breaks in Rat Brain Cells

ER -

TY - JOUR

IS - 1-2

JO - Mutation Research - Fundamental and Molecular Mechanism of Mutagenesis

PY - 2005

SN - 0027-5107

VL - 578

AU - Baohong W

AU - Jiliang H

AU - Lifen J

AU - Deqiang L

AU - Wei Z

AU - Jianlin L

AU - Hongping D

DO - 10.1016/j.mrfmmm.2005.05.001

LA - en

N1 - FEMU ID: 12063; EMF-Portal URL: https://www.emf-portal.org/en/article/12063

SP - 149-157

TI - Studying the synergistic damage effects induced by 1.8 GHz radiofrequency field radiation (RFR) with four chemical mutagens on human lymphocyte DNA using comet assay in vitro

ER -

TY - JOUR

IS - 3

JA - Int J Androl

JO - International Journal of Andrology

PY - 2005

SN - 0105-6263

VL - 28

AU - Aitken RJ

AU - Bennetts LE

AU - Sawyer D

AU - Wiklendt AM

AU - King BV

DO - 10.1111/j.1365-2605.2005.00531.x

LA - en

N1 - FEMU ID: 11992; EMF-Portal URL: https://www.emf-portal.org/en/article/11992

SP - 171-179

TI - Impact of radio frequency electromagnetic radiation on DNA integrity in the male germline

ER -

TY - JOUR

IS - 2

JA - Mutat Res Genet Toxicol Environ Mutagen

JO - Mutation Research - Genetic Toxicology and Environmental Mutagenesis

PY - 2005

VL - 583

AU - Diem E

AU - Schwarz C

AU - Adlkofer F

AU - Jahn O

AU - Rüdiger H

DO - 10.1016/j.mrgentox.2005.03.006

LA - en

N1 - FEMU ID: 11910; EMF-Portal URL: https://www.emf-portal.org/en/article/11910

SP - 178-183

TI - Non-thermal DNA breakage by mobile-phone radiation (1800 MHz) in human fibroblasts and in transformed GFSH-R17 rat granulosa cells in vitro

ER -

TY - JOUR

IS - 4

JO - Bioelectromagnetics

PY - 2005

SN - 0197-8462

VL - 26

AU - Zeni O

AU - Romano M

AU - Perrotta A

AU - Lioi MB

AU - Barbieri R

AU - d'Ambrosio G

AU - Massa R

AU - Scarfi MR

DO - 10.1002/bem.20078

LA - en

N1 - FEMU ID: 11836; EMF-Portal URL: https://www.emf-portal.org/en/article/11836

SP - 258-265

TI - Evaluation of genotoxic effects in human peripheral blood leukocytes following an acute in vitro exposure to 900 MHz radiofrequency fields

ER -

TY - JOUR

IS - 2

JA - Eur J Cancer Prev

JO - European Journal of Cancer Prevention

PY - 2005

SN - 0959-8278

VL - 14

AU - Chang SK

AU - Choi JS

AU - Gil HW

AU - Yang JO

AU - Lee EY

AU - Jeon YS

AU - Lee ZW

AU - Lee M

AU - Hong MY

AU - Ho Son T

AU - Hong SY

DO - 10.1097/00008469-200504000-00014

LA - en

N1 - FEMU ID: 11767; EMF-Portal URL: https://www.emf-portal.org/en/article/11767

SP - 175-179

TI - Genotoxicity evaluation of electromagnetic fields generated by 835-MHz mobile phone frequency band

ER -

TY - JOUR

IS - 3

JO - Bioelectromagnetics

PY - 2005

SN - 0197-8462

VL - 26

AU - Belyaev IY

AU - Hillert L

AU - Protopopova M

AU - Tamm C

AU - Malmgren LO

AU - Persson BR

AU - Selivanova G

AU - Harms-Ringdahl M

DO - 10.1002/bem.20103

LA - en

N1 - FEMU ID: 11713; EMF-Portal URL: https://www.emf-portal.org/en/article/11713

SP - 173-184

TI - 915 MHz microwaves and 50 Hz magnetic field affect chromatin conformation and 53BP1 foci in human lymphocytes from hypersensitive and healthy persons

ER -

TY - JOUR

IS - 4

JA - IEEE Trans Plasma Sci

JO - IEEE Transactions on Plasma Science

PY - 2004

SN - 0093-3813

VL - 32

AU - Sarimov R

AU - Malmgren LOG

AU - Markova E

AU - Persson BRR

AU - Belyaev IY

DO - 10.1109/TPS.2004.832613

LA - en

N1 - FEMU ID: 13460; EMF-Portal URL: https://www.emf-portal.org/en/article/13460

SP - 1600-1608

TI - Nonthermal GSM Microwaves Affect Chromatin Conformation in Human Lymphocytes Similar to Heat Shock

ER -

TY - JOUR

IS - 6

JA - Int J Hyg Environ Health

JO - International Journal of Hygiene and Environmental Health

PY - 2004

SN - 1438-4639

VL - 207

AU - Busljeta I

AU - Trosic I

AU - Milkovic-Kraus S

DO - 10.1078/1438-4639-00326

LA - en

N1 - FEMU ID: 11630; EMF-Portal URL: https://www.emf-portal.org/en/article/11630

SP - 549-554

TI - Erythropoietic changes in rats after 2.45 GHz nonthermal irradiation

ER -

TY - JOUR

IS - 5

JO - Mutagenesis

PY - 2004

SN - 0267-8357

VL - 19

AU - Trosic I

AU - Busljeta I

AU - Modlic B

DO - 10.1093/mutage/geh042

LA - en

N1 - FEMU ID: 11628; EMF-Portal URL: https://www.emf-portal.org/en/article/11628

SP - 361-364

TI - Investigation of the genotoxic effect of microwave irradiation in rat bone marrow cells: in vivo exposure

UR - https://academic.oup.com/mutage/article-pdf/19/5/361/4067612/geh042.pdf

ER -

TY - JOUR

JA - ScientificWorldJournal

JO - The Scientific World Journal

PY - 2004

SN - 1537-744X

VL - 4

AU - Koyama S

AU - Isozumi Y

AU - Suzuki Y

AU - Taki M

AU - Miyakoshi J

DO - 10.1100/tsw.2004.176

LA - en

N1 - FEMU ID: 11393; EMF-Portal URL: https://www.emf-portal.org/en/article/11393

SP - 29-40

TI - Effects of 2.45-GHz electromagnetic fields with a wide range of SARs on micronucleus formation in CHO-K1 cells

UR - http://downloads.hindawi.com/journals/tswj/2004/743762.pdf

ER -

TY - JOUR

IS - 1-2

JO - Mutation Research - Fundamental and Molecular Mechanism of Mutagenesis

PY - 2004

SN - 0027-5107

VL - 545

AU - Paulraj R

AU - Behari J

DO - 10.1016/s0027-5107(03)00113-1

LA - en

N1 - FEMU ID: 11325; EMF-Portal URL: https://www.emf-portal.org/en/article/11325

SP - 127-130

TI - Radio frequency radiation effects on protein kinase C activity in rats' brain

ER -

TY - JOUR

IS - 1

JA - Int J Radiat Biol

JO - International Journal of Radiation Biology

PY - 2004

SN - 0955-3002

VL - 80

AU - Lagroye I

AU - Anane R

AU - Wettring BA

AU - Moros EG

AU - Straube WL

AU - LaRegina MC

AU - Niehoff M

AU - Pickard WF

AU - Baty J

AU - Roti Roti JL

DO - 10.1080/09553000310001642911

LA - en

N1 - FEMU ID: 11161; EMF-Portal URL: https://www.emf-portal.org/en/article/11161

SP - 11-20

TI - Measurement of DNA damage after acute exposure to pulsed-wave 2450 MHz microwaves in rat brain cells by two alkaline comet assay methods

ER -

TY - JOUR

IS - 1-2

JA - Mutat Res Genet Toxicol Environ Mutagen

JO - Mutation Research - Genetic Toxicology and Environmental Mutagenesis

PY - 2004

VL - 558

AU - Chemeris NK

AU - Gapeyev AB

AU - Sirota NP

AU - Gudkova OY

AU - Kornienko NV

AU - Tankanag AV

AU - Konovalov IV

AU - Buzoverya ME

AU - Suvorov VG

AU - Logunov VA

DO - 10.1016/j.mrgentox.2003.10.017

LA - en

N1 - FEMU ID: 10650; EMF-Portal URL: https://www.emf-portal.org/en/article/10650

SP - 27-34

TI - DNA damage in frog erythrocytes after in vitro exposure to a high peak-power pulsed electromagnetic field

ER -

TY - JOUR

IS - 2

JA - Radiat Res

JO - Radiation Research

PY - 2004

SN - 0033-7587

VL - 161

AU - Hook GJ

AU - Zhang P

AU - Lagroye I

AU - Li L

AU - Higashikubo R

AU - Moros EG

AU - Straube WL

AU - Pickard WF

AU - Baty JD

AU - Roti Roti JL

DO - 10.1667/rr3127

LA - en

N1 - FEMU ID: 10635; EMF-Portal URL: https://www.emf-portal.org/en/article/10635

SP - 193-200

TI - Measurement of DNA damage and apoptosis in Molt-4 cells after in vitro exposure to radiofrequency radiation

ER -

TY - JOUR

IS - 2

JA - Radiat Res

JO - Radiation Research

PY - 2004

SN - 0033-7587

VL - 161

AU - Lagroye I

AU - Hook GJ

AU - Wettring BA

AU - Baty JD

AU - Moros EG

AU - Straube WL

AU - Roti Roti JL

DO - 10.1667/rr3122

LA - en

N1 - FEMU ID: 10634; EMF-Portal URL: https://www.emf-portal.org/en/article/10634

SP - 201-214

TI - Measurements of alkali-labile DNA damage and protein-DNA crosslinks after 2450 MHz microwave and low-dose gamma irradiation in vitro

ER -

TY - JOUR

IS - 3

JA - Radiat Res

JO - Radiation Research

PY - 2004

SN - 0033-7587

VL - 161

AU - Vijayalaxmi

AU - Logani MK

AU - Bhanushali A

AU - Ziskin MC

AU - Prihoda TJ

DO - 10.1667/rr3121

LA - en

N1 - FEMU ID: 10560; EMF-Portal URL: https://www.emf-portal.org/en/article/10560

SP - 341-345

TI - Micronuclei in peripheral blood and bone marrow cells of mice exposed to 42 GHz electromagnetic millimeter waves

ER -

TY - JOUR

IS - 2

JO - Bioelectromagnetics

PY - 2004

SN - 0197-8462

VL - 25

AU - Markkanen A

AU - Penttinen P

AU - Naarala J

AU - Pelkonen J

AU - Sihvonen AP

AU - Juutilainen J

DO - 10.1002/bem.10167

LA - en

N1 - FEMU ID: 10487; EMF-Portal URL: https://www.emf-portal.org/en/article/10487

SP - 127-133

TI - Apoptosis induced by ultraviolet radiation is enhanced by amplitude modulated radiofrequency radiation in mutant yeast cells

ER -

TY - JOUR

IS - 2

JA - J Cell Physiol

JO - Journal of Cellular Physiology

PY - 2004

SN - 0021-9541

VL - 198

AU - Marinelli F

AU - La Sala D

AU - Cicciotti G

AU - Cattini L

AU - Trimarchi C

AU - Putti S

AU - Zamparelli A

AU - Giuliani L

AU - Tomassetti G

AU - Cinti C

DO - 10.1002/jcp.10425

LA - en

N1 - FEMU ID: 10381; EMF-Portal URL: https://www.emf-portal.org/en/article/10381

SP - 324-332

TI - Exposure to 900 MHz electromagnetic field induces an unbalance between pro-apoptotic and pro-survival signals in T-lymphoblastoid leukemia CCRF-CEM cells

ER -

TY - JOUR

IS - 9

JA - Int J Radiat Biol

JO - International Journal of Radiation Biology

PY - 2003

SN - 0955-3002

VL - 79

AU - Port M

AU - Abend M

AU - Romer B

AU - Van Beuningen D

DO - 10.1080/09553000310001606803

LA - en

N1 - FEMU ID: 10792; EMF-Portal URL: https://www.emf-portal.org/en/article/10792

SP - 701-708

TI - Influence of high-frequency electromagnetic fields on different modes of cell death and gene expression

ER -

TY - JOUR

IS - 2-3

JA - Electromagn Biol Med

JO - Electromagnetic Biology and Medicine

PY - 2003

SN - 1536-8386

VL - 22

AU - Gadhia PK

AU - Shah T

AU - Mistry A

AU - Pithawala M

AU - Tamakuvala D

DO - 10.1081/JBC-120024624

LA - en

N1 - FEMU ID: 10674; EMF-Portal URL: https://www.emf-portal.org/en/article/10674

SP - 149-159

TI - A Preliminary Study to Assess Possible Chromosomal Damage Among Users of Digital Mobile Phones

ER -

TY - JOUR

IS - 1-2

JA - Mutat Res Genet Toxicol Environ Mutagen

JO - Mutation Research - Genetic Toxicology and Environmental Mutagenesis

PY - 2003

VL - 542

AU - Stacey M

AU - Stickley J

AU - Fox P

AU - Statler V

AU - Schoenbach K

AU - Beebe SJ

AU - Buescher S

DO - 10.1016/j.mrgentox.2003.08.006

LA - en

N1 - FEMU ID: 10503; EMF-Portal URL: https://www.emf-portal.org/en/article/10503

SP - 65-75

TI - Differential effects in cells exposed to ultra-short, high intensity electric fields: cell survival, DNA damage, and cell cycle analysis

ER -

TY - JOUR

IS - 1-2

JA - Mutat Res Genet Toxicol Environ Mutagen

JO - Mutation Research - Genetic Toxicology and Environmental Mutagenesis

PY - 2003

VL - 541

AU - Koyama S

AU - Nakahara T

AU - Wake K

AU - Taki M

AU - Isozumi Y

AU - Miyakoshi J

DO - 10.1016/j.mrgentox.2003.07.009

LA - en

N1 - FEMU ID: 10341; EMF-Portal URL: https://www.emf-portal.org/en/article/10341

SP - 81-89

TI - Effects of high frequency electromagnetic fields on micronucleus formation in CHO-K1 cells

ER -

TY - JOUR

IS - 2

JA - Radiat Res

JO - Radiation Research

PY - 2003

SN - 0033-7587

VL - 160

AU - Zeni O

AU - Chiavoni AS

AU - Sannino A

AU - Antolini A

AU - Forigo D

AU - Bersani F

AU - Scarfi MR

DO - 10.1667/rr3014

LA - en

N1 - FEMU ID: 10036; EMF-Portal URL: https://www.emf-portal.org/en/article/10036

SP - 152-158

TI - Lack of genotoxic effects (micronucleus induction) in human lymphocytes exposed in vitro to 900 MHz electromagnetic fields

ER -

TY - JOUR

IS - 4

JA - Radiat Res

JO - Radiation Research

PY - 2003

SN - 0033-7587

VL - 159

AU - Vijayalaxmi

AU - Sasser LB

AU - Morris JE

AU - Wilson BW

AU - Anderson LE

DO - 10.1667/0033-7587(2003)159[0558:gpogwc]2.0.co;2

LA - en

N1 - FEMU ID: 9893; EMF-Portal URL: https://www.emf-portal.org/en/article/9893

SP - 558-564

TI - Genotoxic potential of 1.6 GHz wireless communication signal: in vivo two-year bioassay

UR - https://meridian.allenpress.com/radiation-research/article-pdf/159/4/558/2194600/0033-7587(2003)159%5b0558_gpogwc%5d2_0_co_2.pdf

ER -

TY - JOUR

IS - 5

JA - Radiat Res

JO - Radiation Research

PY - 2003

SN - 0033-7587

VL - 159

AU - McNamee JP

AU - Bellier PV

AU - Gajda GB

AU - Lavallee BF

AU - Marro L

AU - Lemay E

AU - Thansandote A

DO - 10.1667/0033-7587(2003)159[0693:nefgef]2.0.co;2

LA - en

N1 - FEMU ID: 9883; EMF-Portal URL: https://www.emf-portal.org/en/article/9883

SP - 693-697

TI - No evidence for genotoxic effects from 24 h exposure of human leukocytes to 1.9 GHz radiofrequency fields

ER -

TY - JOUR

IS - 2

JO - Bioelectromagnetics

PY - 2003

SN - 0197-8462

VL - 24

AU - Mashevich M

AU - Folkman D

AU - Kesar A

AU - Barbul A

AU - Korenstein R

AU - Jerby E

AU - Avivi L

DO - 10.1002/bem.10086

LA - en

N1 - FEMU ID: 9413; EMF-Portal URL: https://www.emf-portal.org/en/article/9413

SP - 82-90

TI - Exposure of human peripheral blood lymphocytes to electromagnetic fields associated with cellular phones leads to chromosomal instability

ER -

TY - JOUR

IS - 4

JA - IEEE Trans Plasma Sci

JO - IEEE Transactions on Plasma Science

PY - 2002

SN - 0093-3813

VL - 30

AU - Yu G

AU - Coln EA

AU - Schoenbach KH

AU - Gellermann M

AU - Fox P

AU - Rec L

AU - Beebe SJ

AU - Liu S

DO - 10.1109/TPS.2002.804179

LA - en

N1 - FEMU ID: 18244; EMF-Portal URL: https://www.emf-portal.org/en/article/18244

SP - 1489-1496

TI - A study on biological effects of low-intensity millimeter waves

ER -

TY - JOUR

IS - 1-2

JA - Mutat Res Genet Toxicol Environ Mutagen

JO - Mutation Research - Genetic Toxicology and Environmental Mutagenesis

PY - 2002

VL - 521

AU - Trosic I

AU - Busljeta I

AU - Kasuba V

AU - Rozgaj R

DO - 10.1016/s1383-5718(02)00214-0

LA - en

N1 - FEMU ID: 11629; EMF-Portal URL: https://www.emf-portal.org/en/article/11629

SP - 73-79

TI - Micronucleus induction after whole-body microwave irradiation of rats

ER -

TY - JOUR

IS - 1

JO - Biochimica et Biophysica Acta - General Subjects

PY - 2002

VL - 1572

AU - Shcheglov VS

AU - Alipov ED

AU - Belyaev IY

DO - 10.1016/s0304-4165(02)00283-0

LA - en

N1 - FEMU ID: 10302; EMF-Portal URL: https://www.emf-portal.org/en/article/10302

SP - 101-106

TI - Cell-to-cell communication in response of E. coli cells at different phases of growth to low-intensity microwaves

ER -

TY - JOUR

IS - 4

JA - Biomed Environ Sci

JO - Biomedical and Environmental Sciences

PY - 2002

SN - 0895-3988

VL - 15

AU - Zhang MB

AU - He JL

AU - Jin LF

AU - Lu DQ

LA - en

N1 - FEMU ID: 9988; EMF-Portal URL: https://www.emf-portal.org/en/article/9988

SP - 283-290

TI - Study of low-intensity 2450-MHz microwave exposure enhancing the genotoxic effects of mitomycin C using micronucleus test and comet assay in vitro

ER -

TY - JOUR

IS - 4

JA - Radiat Res

JO - Radiation Research

PY - 2002

SN - 0033-7587

VL - 158

AU - McNamee JP

AU - Bellier PV

AU - Gajda GB

AU - Miller SM

AU - Lemay EP

AU - Lavallee BF

AU - Marro L

AU - Thansandote A

DO - 10.1667/0033-7587(2002)158[0523:ddamii]2.0.co;2

LA - en

N1 - FEMU ID: 9741; EMF-Portal URL: https://www.emf-portal.org/en/article/9741

SP - 523-533

TI - DNA damage and micronucleus induction in human leukocytes after acute in vitro exposure to a 1.9 GHz continuous-wave radiofrequency field

ER -

TY - JOUR

IS - 4

JA - Radiat Res

JO - Radiation Research

PY - 2002

SN - 0033-7587

VL - 158

AU - McNamee JP

AU - Bellier PV

AU - Gajda GB

AU - Lavallee BF

AU - Lemay EP

AU - Marro L

AU - Thansandote A

DO - 10.1667/0033-7587(2002)158[0534:ddihla]2.0.co;2

LA - en

N1 - FEMU ID: 9740; EMF-Portal URL: https://www.emf-portal.org/en/article/9740

SP - 534-537

TI - DNA damage in human leukocytes after acute in vitro exposure to a 1.9 GHz pulse-modulated radiofrequency field

ER -

TY - JOUR

IS - 5

JA - Radiat Res

JO - Radiation Research

PY - 2002

SN - 0033-7587

VL - 157

AU - Bisht KS

AU - Moros EG

AU - Straube WL

AU - Baty JD

AU - Roti Roti JL

DO - 10.1667/0033-7587(2002)157[0506:teomfo]2.0.co;2

LA - en

N1 - FEMU ID: 8831; EMF-Portal URL: https://www.emf-portal.org/en/article/8831

SP - 506-515

TI - The effect of 835.62 MHz FDMA or 847.74 MHz CDMA modulated radiofrequency radiation on the induction of micronuclei in C3H 10T(1/2) cells

ER -

TY - JOUR

IS - 7

JA - Cancer Res

JO - Cancer Research

PY - 2002

SN - 0008-5472

VL - 62

AU - Takahashi S

AU - Inaguma S

AU - Cho YM

AU - Imaida K

AU - Wang J

AU - Fujiwara O

AU - Shirai T

LA - en

N1 - FEMU ID: 8665; EMF-Portal URL: https://www.emf-portal.org/en/article/8665

SP - 1956-1960

TI - Lack of mutation induction with exposure to 1.5 GHz electromagnetic near fields used for cellular phones in brains of Big Blue mice

UR - https://cancerres.aacrjournals.org/content/62/7/1956.full.pdf+html

ER -

TY - JOUR

IS - 2

JO - Bioelectromagnetics

PY - 2002

SN - 0197-8462

VL - 23

AU - Tice RR

AU - Hook GG

AU - Donner M

AU - McRee DI

AU - Guy AW

DO - 10.1002/bem.104

LA - en

N1 - FEMU ID: 8518; EMF-Portal URL: https://www.emf-portal.org/en/article/8518

SP - 113-126

TI - Genotoxicity of radiofrequency signals. I. Investigation of DNA damage and micronuclei induction in cultured human blood cells

ER -

TY - JOUR

IS - 1

JO - Bioelectromagnetics

PY - 2002

SN - 0197-8462

VL - 23

AU - d'Ambrosio G

AU - Massa R

AU - Scarfi MR

AU - Zeni O

DO - 10.1002/bem.93

LA - en

N1 - FEMU ID: 8130; EMF-Portal URL: https://www.emf-portal.org/en/article/8130

SP - 7-13

TI - Cytogenetic damage in human lymphocytes following GMSK phase modulated microwave exposure

ER -

TY - JOUR

IS - 2

JA - Acta Med Okayama

JO - Acta Medica Okayama

PY - 2001

SN - 0386-300X

VL - 55

AU - Lalic H

AU - Lekic A

AU - Radosevic-Stasic B

DO - 10.18926/AMO/32005

LA - en

N1 - FEMU ID: 8555; EMF-Portal URL: https://www.emf-portal.org/en/article/8555

SP - 117-127

TI - Comparison of chromosome aberrations in peripheral blood lymphocytes from people occupationally exposed to ionizing and radiofrequency radiation

UR - http://www.lib.okayama-u.ac.jp/www/acta/pdf/55_2_117.pdf

ER -

TY - JOUR

IS - 3

JA - Radiat Res

JO - Radiation Research

PY - 2001

SN - 0033-7587

VL - 156

AU - Li L

AU - Bisht KS

AU - Lagroye I

AU - Zhang P

AU - Straube WL

AU - Moros EG

AU - Roti Roti JL

DO - 10.1667/0033-7587(2001)156[0328:moddim]2.0.co;2

LA - en

N1 - FEMU ID: 8548; EMF-Portal URL: https://www.emf-portal.org/en/article/8548

SP - 328-332

TI - Measurement of DNA damage in mammalian cells exposed in vitro to radiofrequency fields at SARs of 3-5 W/kg

ER -

TY - JOUR

IS - 6

JA - J Magn Reson Imaging

JO - Journal of Magnetic Resonance Imaging

PY - 2001

SN - 1053-1807

VL - 14

AU - Schreiber WG

AU - Teichmann EM

AU - Schiffer I

AU - Hast J

AU - Akbari W

AU - Georgi H

AU - Graf R

AU - Hehn M

AU - Spiebeta HW

AU - Thelen M

AU - Oesch F

AU - Hengstler JG

DO - 10.1002/jmri.10010

LA - en

N1 - FEMU ID: 7986; EMF-Portal URL: https://www.emf-portal.org/en/article/7986

SP - 779-788

TI - Lack of mutagenic and co-mutagenic effects of magnetic fields during magnetic resonance imaging

ER -

TY - JOUR

IS - 4

JA - Radiat Res

JO - Radiation Research

PY - 2001

SN - 0033-7587

VL - 156

AU - Vijayalaxmi

AU - Bisht KS

AU - Pickard WF

AU - Meltz ML

AU - Roti Roti JL

AU - Moros EG

DO - 10.1667/0033-7587(2001)156[0430:cdamfi]2.0.co;2

LA - en

N1 - FEMU ID: 7827; EMF-Portal URL: https://www.emf-portal.org/en/article/7827

SP - 430-433

TI - Chromosome damage and micronucleus formation in human blood lymphocytes exposed in vitro to radiofrequency radiation at a cellular telephone frequency (847.74 MHz, CDMA)

ER -

TY - JOUR

IS - 11

JA - Int J Radiat Biol

JO - International Journal of Radiation Biology

PY - 2001

SN - 0955-3002

VL - 77

AU - Vijayalaxmi

AU - Pickard WF

AU - Bisht KS

AU - Prihoda TJ

AU - Meltz ML

AU - LaRegina MC

AU - Roti Roti JL

AU - Straube WL

AU - Moros EG

DO - 10.1080/09553000110069100

LA - en

N1 - FEMU ID: 7826; EMF-Portal URL: https://www.emf-portal.org/en/article/7826

SP - 1109-1115

TI - Micronuclei in the peripheral blood and bone marrow cells of rats exposed to 2450 MHz radiofrequency radiation

ER -

TY - JOUR

IS - 5

JA - Radiat Res

JO - Radiation Research

PY - 2001

SN - 0033-7587

VL - 156

AU - Sykes PJ

AU - McCallum BD

AU - Bangay MJ

AU - Hooker AM

AU - Morley AA

DO - 10.1667/0033-7587(2001)156[0495:eoetmr]2.0.co;2

LA - en

N1 - FEMU ID: 7646; EMF-Portal URL: https://www.emf-portal.org/en/article/7646

SP - 495-502

TI - Effect of exposure to 900 MHz radiofrequency radiation on intrachromosomal recombination in pKZ1 mice

ER -

TY - JOUR

IS - 10

JO - Carcinogenesis

PY - 2001

SN - 0143-3334

VL - 22

AU - Mason PA

AU - Walters TJ

AU - DiGiovanni J

AU - Beason CW

AU - Jauchem JR

AU - Dick Jr EJ

AU - Mahajan K

AU - Dusch SJ

AU - Shields BA

AU - Merritt JH

AU - Murphy MR

AU - Ryan KL

DO -

LA - en

N1 - FEMU ID: 7468; EMF-Portal URL: https://www.emf-portal.org/en/article/7468

SP - 1701-1708

TI - Lack of effect of 94 GHz radio frequency radiation exposure in an animal model of skin carcinogenesis

UR - https://academic.oup.com/carcin/article-pdf/22/10/1701/9418014/0221701.pdf

ER -

TY - JOUR

IS - 2

JO - Bioelectromagnetics

PY - 2001

SN - 0197-8462

VL - 22

AU - Maes A

AU - Collier M

AU - Verschaeve L

LA - en

N1 - FEMU ID: 5625; EMF-Portal URL: https://www.emf-portal.org/en/article/5625

SP - 91-96

TI - Cytogenetic effects of 900 MHz (GSM) microwaves on human lymphocytes

ER -

TY - JOUR

IS - 1

JA - Radiat Res

JO - Radiation Research

PY - 2001

SN - 0033-7587

VL - 155

AU - Vijayalaxmi

AU - Leal BZ

AU - Meltz ML

AU - Pickard WF

AU - Bisht KS

AU - Roti Roti JL

AU - Straube WL

AU - Moros EG

DO - 10.1667/0033-7587(2001)155[0113:csihbl]2.0.co;2

LA - en

N1 - FEMU ID: 5187; EMF-Portal URL: https://www.emf-portal.org/en/article/5187

SP - 113-121

TI - Cytogenetic studies in human blood lymphocytes exposed in vitro to radiofrequency radiation at a cellular telephone frequency (835.62 MHz, FDMA)

ER -

TY - JOUR

IS - 11

JA - Rofo

JO - RöFo: Fortschritte auf dem Gebiet der Röntgenstrahlen und der bildgebenden Verfahren

PY - 2000

SN - 1438-9010

VL - 172

AU - Teichmann EM

AU - Hengstler JG

AU - Schreiber WG

AU - Akbari W

AU - Georgi H

AU - Hehn M

AU - Schiffer I

AU - Oesch F

AU - Spiess HW

AU - Thelen M

DO - 10.1055/s-2000-8378

LA - de

N1 - FEMU ID: 5715; EMF-Portal URL: https://www.emf-portal.org/en/article/5715

SP - 934-939

TI - Untersuchung eines möglich mutagenen Potenzials von Magnetfeldern

ER -

TY - JOUR

IS - 1-2

JA - Mutat Res Genet Toxicol Environ Mutagen

JO - Mutation Research - Genetic Toxicology and Environmental Mutagenesis

PY - 2000

VL - 471

AU - Zotti-Martelli L

AU - Peccatori M

AU - Scarpato R

AU - Migliore L

DO - 10.1016/s1383-5718(00)00112-1

LA - en

N1 - FEMU ID: 5088; EMF-Portal URL: https://www.emf-portal.org/en/article/5088

SP - 51-58

TI - Induction of micronuclei in human lymphocytes exposed in vitro to microwave radiation

ER -

TY - JOUR

IS - 5

JA - Folia Biol

JO - Folia Biologica

PY - 2000

SN - 0015-5500

VL - 46

AU - Maes A

AU - Collier M

AU - Verschaeve L

LA - en

N1 - FEMU ID: 4988; EMF-Portal URL: https://www.emf-portal.org/en/article/4988

SP - 175-180

TI - Cytogenetic investigations on microwaves emitted by a 455.7 MHz car phone

ER -

TY - JOUR

IS - 4

JA - Radiat Res

JO - Radiation Research

PY - 2000

SN - 0033-7587

VL - 153

AU - Vijayalaxmi

AU - Leal BZ

AU - Szilagyi M

AU - Prihoda TJ

AU - Meltz ML

DO - 10.1667/0033-7587(2000)153[0479:pddihb]2.0.co;2

LA - en

N1 - FEMU ID: 4299; EMF-Portal URL: https://www.emf-portal.org/en/article/4299

SP - 479-486

TI - Primary DNA damage in human blood lymphocytes exposed in vitro to 2450 MHz radiofrequency radiation

ER -

TY - JOUR

IS - 7

JO - Bioelectromagnetics

PY - 2000

SN - 0197-8462

VL - 21

AU - Gos P

AU - Eicher B

AU - Kohli J

AU - Heyer WD

DO - 10.1002/1521-186x(200010)21:7<515::aid-bem5>3.0.co;2-k

LA - en

N1 - FEMU ID: 3935; EMF-Portal URL: https://www.emf-portal.org/en/article/3935

SP - 515-523

TI - No mutagenic or recombinogenic effects of mobile phone fields at 900 MHz detected in the yeast saccharomyces cerevisiae

ER -

TY - JOUR

IS - 13

JO - Chemosphere

PY - 1999

SN - 0045-6535

VL - 39

AU - Garaj-Vrhovac V

DO - 10.1016/s0045-6535(99)00139-3

LA - en

N1 - FEMU ID: 5642; EMF-Portal URL: https://www.emf-portal.org/en/article/5642

SP - 2301-2312

TI - Micronucleus assay and lymphocyte mitotic activity in risk assessment of occupational exposure to microwave radiation

ER -

TY - JOUR

IS - 2

JA - J Microw Power Electromagn Energy

JO - Journal of Microwave Power and Electromagnetic Energy

PY - 1998

SN - 0832-7823

VL - 33

AU - Kuchma T

DO - 10.1080/08327823.1998.11688363

LA - en

N1 - FEMU ID: 11053; EMF-Portal URL: https://www.emf-portal.org/en/article/11053

SP - 77-87

TI - Synergistic effect of microwave heating and hydrogen peroxide on inactivation of microorganisms

ER -

TY - JOUR

JO - Edition Wissenschaft

PY - 1998

VL - 14

AU - Antonopoulos A

AU - Obe G

AU - Brinkmann K

AU - Eisenbrandt H

AU - Grigat JP

AU - Elsner R

AU - Storbeck W

AU - Dehmel G

LA - de

N1 - FEMU ID: 9446; EMF-Portal URL: https://www.emf-portal.org/en/article/9446

SP - 3-13

TI - Der Einfluß von hochfrequenten elektromagnetischen Feldern auf den Zellzyklus und auf die Frequenz von Schwesterchromatidaustauschen: Analysen an menschlichen Lymphozyten in Kultur

UR - https://d-nb.info/974863599/34

ER -

TY - JOUR

IS - 1

JO - Mutation Research - Fundamental and Molecular Mechanism of Mutagenesis

PY - 1998

SN - 0027-5107

VL - 399

AU - Daniells C

AU - Duce I

AU - Thomas D

AU - Sewell P

AU - Tattersall J

AU - de Pomerai DI

DO - 10.1016/s0027-5107(97)00266-2

LA - en

N1 - FEMU ID: 4467; EMF-Portal URL: https://www.emf-portal.org/en/article/4467

SP - 55-64

TI - Transgenic nematodes as biomonitors of microwave-induced stress

ER -

TY - JOUR

IS - 1

JA - Bioelectrochem Bioenerg

JO - Bioelectrochemistry and Bioenergetics

PY - 1998

SN - 0302-4598

VL - 45

AU - Phillips JL

AU - Ivaschuk O

AU - Ishida-Jones T

AU - Jones RA

AU - Campbell-Beachler M

AU - Haggren W

LA - en

N1 - FEMU ID: 2112; EMF-Portal URL: https://www.emf-portal.org/en/article/2112

SP - 103-110

TI - DNA damage in Molt-4 T-lymphoblastoid cells exposed to cellular telephone radiofrequency fields in vitro

ER -

TY - JOUR

IS - 2

JO - Bioelectromagnetics

PY - 1998

SN - 0197-8462

VL - 19

AU - Pakhomova ON

AU - Belt ML

AU - Mathur SP

AU - Lee JC

AU - Akyel Y

DO - 10.1002/(sici)1521-186x(1998)19:2<128::aid-bem12>3.0.co;2-m

LA - en

N1 - FEMU ID: 2082; EMF-Portal URL: https://www.emf-portal.org/en/article/2082

SP - 128-130

TI - Ultra-wide band electromagnetic radiation does not affect UV-induced recombination and mutagenesis in yeast

ER -

TY - JOUR

IS - 6

JA - Radiat Res

JO - Radiation Research

PY - 1998

SN - 0033-7587

VL - 149

AU - Malyapa RS

AU - Ahern EW

AU - Bi C

AU - Straube WL

AU - LaRegina MC

AU - Pickard WF

AU - Roti Roti JL

LA - en

N1 - FEMU ID: 1374; EMF-Portal URL: https://www.emf-portal.org/en/article/1374

SP - 637-645

TI - DNA damage in rat brain cells after in vivo exposure to 2450 MHz electromagnetic radiation and various methods of euthanasia

ER -

TY - JOUR

IS - 6

JA - Wirel Netw

JO - Wireless Networks

PY - 1997

VL - 3

AU - Lai H

AU - Carino M

AU - Singh N

DO - 10.1023/A:1019154611749

LA - en

N1 - FEMU ID: 10681; EMF-Portal URL: https://www.emf-portal.org/en/article/10681

SP - 471-476

TI - Naltrexone blocks RFR-induced DNA double strand breaks in rat brain cells

UR - https://dl.acm.org/doi/pdf/10.1023/A%3A1019154611749

ER -

TY - JOUR

IS - 3

JA - Electro Magnetobiol

JO - Electro- and Magnetobiology

PY - 1997

SN - 1061-9526

VL - 16

AU - Pakhomova ON

AU - Belt MN

AU - Mathur SP

AU - Lee JC

AU - Akyel Y

DO - 10.3109/15368379709015652

LA - en

N1 - FEMU ID: 8582; EMF-Portal URL: https://www.emf-portal.org/en/article/8582

SP - 195-201

TI - Lack of genetic effects of ultra-wide band electromagnetic radiation in yeast

ER -

TY - JOUR

IS - 6

JA - Int J Radiat Biol

JO - International Journal of Radiation Biology

PY - 1997

SN - 0955-3002

VL - 72

AU - Vijayalaxmi

AU - Mohan N

AU - Meltz ML

AU - Wittler MA

DO - 10.1080/095530097142915

LA - en

N1 - FEMU ID: 2359; EMF-Portal URL: https://www.emf-portal.org/en/article/2359

SP - 751-757

TI - Proliferation and cytogenetic studies in human blood lymphocytes exposed in vitro to 2450 MHz radiofrequency radiation

ER -

TY - JOUR

IS - 2-3

JA - Mutat Res Genet Toxicol Environ Mutagen

JO - Mutation Research - Genetic Toxicology and Environmental Mutagenesis

PY - 1997

VL - 395

AU - Antonopoulos A

AU - Eisenbrandt H

AU - Obe G

DO - 10.1016/s1383-5718(97)00173-3

LA - en

N1 - FEMU ID: 2200; EMF-Portal URL: https://www.emf-portal.org/en/article/2200

SP - 209-214

TI - Effects of high-frequency electromagnetic fields on human lymphocytes in vitro

ER -

TY - JOUR

IS - 6

JA - Radiat Res

JO - Radiation Research

PY - 1997

SN - 0033-7587

VL - 148

AU - Malyapa RS

AU - Ahern EW

AU - Straube WL

AU - Moros EG

AU - Pickard WF

AU - Roti Roti JL

LA - en

N1 - FEMU ID: 2070; EMF-Portal URL: https://www.emf-portal.org/en/article/2070

SP - 608-617

TI - Measurement of DNA damage after exposure to 2450 MHz electromagnetic radiation

ER -

TY - JOUR

IS - 4

JA - Radiat Res

JO - Radiation Research

PY - 1997

SN - 0033-7587

VL - 147

AU - Vijayalaxmi

AU - Frei MR

AU - Dusch SJ

AU - Guel V

AU - Meltz ML

AU - Jauchem JR

LA - en

N1 - FEMU ID: 1413; EMF-Portal URL: https://www.emf-portal.org/en/article/1413

SP - 495-500

TI - Frequency of micronuclei in the peripheral blood and bone marrow of cancer-prone mice chronically exposed to 2450 MHz radiofrequency radiation

ER -

TY - JOUR

IS - 6

JO - Bioelectromagnetics

PY - 1997

SN - 0197-8462

VL - 18

AU - Lai H

AU - Singh NP

DO - 10.1002/(sici)1521-186x(1997)18:6<446::aid-bem7>3.0.co;2-2

LA - en

N1 - FEMU ID: 1257; EMF-Portal URL: https://www.emf-portal.org/en/article/1257

SP - 446-454

TI - Melatonin and a spin-trap compound block radiofrequency electromagnetic radiation-induced DNA strand breaks in rat brain cells

ER -

TY - JOUR

IS - 2

JA - Bioelectrochem Bioenerg

JO - Bioelectrochemistry and Bioenergetics

PY - 1997

SN - 0302-4598

VL - 43

AU - Pakhomova ON

AU - Pakhomov AG

AU - Akyel Y

DO - 10.1016/S0302-4598(96)05158-6

LA - en

N1 - FEMU ID: 963; EMF-Portal URL: https://www.emf-portal.org/en/article/963

SP - 227-232

TI - Effect of millimeter waves on UV-induced recombination and mutagenesis in yeast

ER -

TY - JOUR

IS - 1-2

JA - Mutat Res Genet Toxicol Environ Mutagen

JO - Mutation Research - Genetic Toxicology and Environmental Mutagenesis

PY - 1997

VL - 393

AU - Maes A

AU - Collier M

AU - Van Gorp U

AU - Vandoninck S

AU - Verschaeve L

DO - 10.1016/s1383-5718(97)00100-9

LA - en

N1 - FEMU ID: 948; EMF-Portal URL: https://www.emf-portal.org/en/article/948

SP - 151-156

TI - Cytogenetic effects of 935.2-MHz (GSM) microwaves alone and in combination with mitomycin C

ER -

TY - JOUR

IS - 6

JA - Radiat Res

JO - Radiation Research

PY - 1997

SN - 0033-7587

VL - 148

AU - Malyapa RS

AU - Ahern EW

AU - Straube WL

AU - Moros EG

AU - Pickard WF

AU - Roti Roti JL

LA - en

N1 - FEMU ID: 947; EMF-Portal URL: https://www.emf-portal.org/en/article/947

SP - 618-627

TI - Measurement of DNA damage after exposure to electromagnetic radiation in the cellular phone communication frequency band (835.62 and 847.74 MHz)

ER -

TY - JOUR

IS - 3

JO - Bioelectromagnetics

PY - 1997

SN - 0197-8462

VL - 18

AU - Cain CD

AU - Thomas DL

AU - Adey WR

DO - 10.1002/(sici)1521-186x(1997)18:3<237::aid-bem6>3.0.co;2-3

LA - en

N1 - FEMU ID: 925; EMF-Portal URL: https://www.emf-portal.org/en/article/925

SP - 237-243

TI - Focus formation of C3H/10T1/2 cells and exposure to a 836.55 MHz modulated radiofrequency field

ER -

TY - JOUR

JO - Edition Wissenschaft

PY - 1996

VL - 4

AU - Eberle P

AU - Erdtmann-Vourliotis M

AU - Diener S

AU - Finke HG

AU - Löffelholz B

AU - Schnor A

AU - Schräder M

LA - de

N1 - FEMU ID: 9467; EMF-Portal URL: https://www.emf-portal.org/en/article/9467

SP - 2-15

TI - Zellproliferation, Schwesterchromatidaustausche, Chromosomenaberrationen, Mikrokerne und Mutationsrate des HGPRT-Locus

UR - https://d-nb.info/974863475/34

ER -

TY - JOUR

JO - Edition Wissenschaft

PY - 1996

VL - 8

AU - Hansen V

AU - Rüger W

LA - de

N1 - FEMU ID: 9463; EMF-Portal URL: https://www.emf-portal.org/en/article/9463

SP - 3-38

TI - Wirkung hochfrequenter elektromagnetischer Felder auf DNA, Proteine und DNA-Protein-Komplexe

UR - https://d-nb.info/974870188/34

ER -

TY - JOUR

IS - 1

JA - Sci Total Environ

JO - Science of the Total Evironment

PY - 1996

SN - 0048-9697

VL - 180

AU - Balode Z

DO - 10.1016/0048-9697(95)04923-1

LA - en

N1 - FEMU ID: 2132; EMF-Portal URL: https://www.emf-portal.org/en/article/2132

SP - 81-85

TI - Assessment of radio-frequency electromagnetic radiation by the micronucleus test in bovine peripheral erythrocytes

ER -

TY - JOUR

IS - 4

JA - Int J Radiat Biol

JO - International Journal of Radiation Biology

PY - 1996

SN - 0955-3002

VL - 69

AU - Lai H

AU - Singh NP

DO - 10.1080/095530096145814

LA - en

N1 - FEMU ID: 1389; EMF-Portal URL: https://www.emf-portal.org/en/article/1389

SP - 513-521

TI - Single- and double-strand DNA breaks in rat brain cells after acute exposure to radiofrequency electromagnetic radiation

ER -

TY - JOUR

IS - 2

JA - Electro Magnetobiol

JO - Electro- and Magnetobiology

PY - 1996

SN - 1061-9526

VL - 15

AU - Scarfi MR

AU - Lioi MB

AU - d'Ambrosio G

AU - Massa R

AU - Zeni O

AU - Di Pietto R

AU - Di Berardino D

DO - 10.3109/15368379609009826

LA - en

N1 - FEMU ID: 959; EMF-Portal URL: https://www.emf-portal.org/en/article/959

SP - 99-107

TI - Genotoxic effects of mitomycin-c and microwave radiation on bovine lymphocytes

ER -

TY - JOUR

IS - 1

JA - Environ Mol Mutagen

JO - Environmental and Molecular Mutagenesis

PY - 1996

SN - 0893-6692

VL - 28

AU - Maes A

AU - Collier M

AU - Slaets D

AU - Verschaeve L

DO - 10.1002/(SICI)1098-2280(1996)28:1<26::AID-EM6>3.0.CO;2-C

LA - en

N1 - FEMU ID: 934; EMF-Portal URL: https://www.emf-portal.org/en/article/934

SP - 26-30

TI - 954 MHz microwaves enhance the mutagenic properties of mitomycin C

ER -

TY - JOUR

IS - 3

JO - Bioelectromagnetics

PY - 1995

SN - 0197-8462

VL - 16

AU - Lai H

AU - Singh NP

DO - 10.1002/bem.2250160309

LA - en

N1 - FEMU ID: 1385; EMF-Portal URL: https://www.emf-portal.org/en/article/1385

SP - 207-210

TI - Acute low-intensity microwave exposure increases DNA single-strand breaks in rat brain cells

ER -

TY - JOUR

IS - 3

JA - Electro Magnetobiol

JO - Electro- and Magnetobiology

PY - 1995

SN - 1061-9526

VL - 14

AU - d'Ambrosio G

AU - Lioi MB

AU - Massa R

AU - Scarfi MR

AU - Zeni O

DO - 10.3109/15368379509030726

LA - en

N1 - FEMU ID: 905; EMF-Portal URL: https://www.emf-portal.org/en/article/905

SP - 157-164

TI - Genotoxic Effects of Amplitude-Modulated Microwaves on Human Lymphocytes Exposed in Vitro under Controlled Conditions

ER -

TY - JOUR

IS - 2

JA - Electro Magnetobiol

JO - Electro- and Magnetobiology

PY - 1995

SN - 1061-9526

VL - 14

AU - Maes A

AU - Collier M

AU - Slaets D

AU - Verschaeve L

LA - en

N1 - FEMU ID: 891; EMF-Portal URL: https://www.emf-portal.org/en/article/891

SP - 91-98

TI - Cytogenetic Effects of Microwaves from Mobile Communication Frequencies (954 MHz)

ER -

TY - JOUR

IS - 1

JA - Electro Magnetobiol

JO - Electro- and Magnetobiology

PY - 1994

SN - 1061-9526

VL - 13

AU - Belyaev IY

AU - Alipov YD

AU - Shcheglov VS

AU - Polunin VA

AU - Aizenberg OA

DO - 10.3109/15368379409030698

LA - en

N1 - FEMU ID: 8596; EMF-Portal URL: https://www.emf-portal.org/en/article/8596

SP - 53-66

TI - Cooperative response of Escherichia coli cells to the resonance effect of millimeter waves at super low intensity

ER -

TY - JOUR

IS - 1-2

JO - Mutation Research - Genetic Toxicology

PY - 1994

VL - 320

AU - Sarkar S

AU - Ali S

AU - Behari J

DO - 10.1016/0165-1218(94)90066-3

LA - en

N1 - FEMU ID: 1378; EMF-Portal URL: https://www.emf-portal.org/en/article/1378

SP - 141-147

TI - Effect of low power microwave on the mouse genome: a direct DNA analysis

ER -

TY - JOUR

IS - 1-2

JO - Mutation Research - Letters

PY - 1994

VL - 328

AU - Haider T

AU - Knasmueller S

AU - Kundi M

AU - Haider M

DO - 10.1016/0165-7992(94)90069-8

LA - en

N1 - FEMU ID: 873; EMF-Portal URL: https://www.emf-portal.org/en/article/873

SP - 65-68

TI - Clastogenic effects of radiofrequency radiations on chromosomes of Tradescantia

ER -

TY - JOUR

IS - 1

JA - Electro Magnetobiol

JO - Electro- and Magnetobiology

PY - 1993

SN - 1061-9526

VL - 12

AU - Belyaev IY

AU - Alipov YD

AU - Polunin VA

AU - Shcheglov VS

DO - 10.3109/15368379309012861

LA - en

N1 - FEMU ID: 2116; EMF-Portal URL: https://www.emf-portal.org/en/article/2116

SP - 39-49

TI - Evidence for dependence of resonant frequency of millimeter wave interaction with Escherischia coli K12 cells on haploid genome length

ER -

TY - JOUR

JA - Bioelectrochem Bioenerg

JO - Bioelectrochemistry and Bioenergetics

PY - 1993

SN - 0302-4598

VL - 30

AU - Garaj-Vrhovac V

AU - Fucic A

DO - 10.1016/0302-4598(93)80091-8

LA - en

N1 - FEMU ID: 1874; EMF-Portal URL: https://www.emf-portal.org/en/article/1874

SP - 319-325

TI - The rate of elimination of chromosomal aberrations after accidental exposure to microwave radiation

ER -

TY - JOUR

IS - 6

JO - Bioelectromagnetics

PY - 1993

SN - 0197-8462

VL - 14

AU - Maes A

AU - Verschaeve L

AU - Arroyo A

AU - De Wagter C

AU - Vercruyssen L

DO - 10.1002/bem.2250140602

LA - en

N1 - FEMU ID: 889; EMF-Portal URL: https://www.emf-portal.org/en/article/889

SP - 495-501

TI - In vitro cytogenetic effects of 2450 MHz waves on human peripheral blood lymphocytes

ER -

TY - JOUR

IS - 7-8

JA - Z Naturforsch C

JO - Zeitschrift für Naturforschung C, Journal of Biosciences

PY - 1992

SN - 0341-0382

VL - 47

AU - Belyaev IY

AU - Alipov YD

AU - Shcheglov VS

AU - Lystsov VN

DO - 10.1515/znc-1992-7-822

LA - en

N1 - FEMU ID: 10177; EMF-Portal URL: https://www.emf-portal.org/en/article/10177

SP - 621-627

TI - Resonance effect of microwaves on the genome conformational state of E. coli cells

UR - https://www.degruyter.com/downloadpdf/j/znc.1992.47.issue-7-8/znc-1992-7-822/znc-1992-7-822.xml

ER -

TY - JOUR

IS - 2

JA - Electro Magnetobiol

JO - Electro- and Magnetobiology

PY - 1992

SN - 1061-9526

VL - 11

AU - Belyaev IY

AU - Alipov YD

AU - Shcheglov VS

DO - 10.3109/15368379209009820

LA - en

N1 - FEMU ID: 2115; EMF-Portal URL: https://www.emf-portal.org/en/article/2115

SP - 97-108

TI - Chromosome DNA as a target of resonant interaction between Escherichia coli cells and low-intensity millimeter waves

ER -

TY - JOUR

IS - 3

JO - Mutation Research - Letters

PY - 1992

VL - 281

AU - Garaj-Vrhovac V

AU - Fucic A

AU - Horvat D

DO - 10.1016/0165-7992(92)90006-4

LA - en

N1 - FEMU ID: 876; EMF-Portal URL: https://www.emf-portal.org/en/article/876

SP - 181-186

TI - The correlation between the frequency of micronuclei and specific chromosome aberrations in human lymphocytes exposed to microwave radiation in vitro

ER -

TY - JOUR

IS - 4

JO - Mutation Research - Letters

PY - 1992

VL - 282

AU - Fucic A

AU - Garaj-Vrhovac V

AU - Skara M

AU - Dimitrovic B

DO - 10.1016/0165-7992(92)90133-3

LA - en

N1 - FEMU ID: 875; EMF-Portal URL: https://www.emf-portal.org/en/article/875

SP - 265-271

TI - X-rays, microwaves and vinyl chloride monomer: their clastogenic and aneugenic activity, using the micronucleus assay on human lymphocytes

ER -

TY - JOUR

IS - 2

JA - Biochem Int

JO - Biochemistry International

PY - 1991

SN - 0158-5231

VL - 25

AU - Narasimhan V

AU - Huh WK

LA - en

N1 - FEMU ID: 8617; EMF-Portal URL: https://www.emf-portal.org/en/article/8617

SP - 363-370

TI - Altered restriction patterns of microwave irradiated lambda-phage DNA

ER -

TY - JOUR

IS - 5

JA - Med J Aust

JO - The Medical Journal of Australia

PY - 1991

SN - 0025-729X

VL - 155

AU - Garson OM

AU - McRobert TL

AU - Campbell LJ

AU - Hocking BA

AU - Gordon I

DO - 10.5694/j.1326-5377.1991.tb142282.x

LA - en

N1 - FEMU ID: 1407; EMF-Portal URL: https://www.emf-portal.org/en/article/1407

SP - 289-292

TI - A chromosomal study of workers with long-term exposure to radio-frequency radiation

ER -

TY - JOUR

IS - 3

JO - Mutation Research - Letters

PY - 1991

VL - 263

AU - Garaj-Vrhovac V

AU - Horvat D

AU - Koren Z

DO - 10.1016/0165-7992(91)90054-8

LA - en

N1 - FEMU ID: 877; EMF-Portal URL: https://www.emf-portal.org/en/article/877

SP - 143-149

TI - The relationship between colony-forming ability, chromosome aberrations and incidence of micronuclei in V79 Chinese hamster cells exposed to microwave radiation

ER -

TY - JOUR

IS - 4

JA - Period Biol

JO - Periodicum Biologorum

PY - 1990

SN - 0031-5362

VL - 92

AU - Garaj-Vrhovac V

AU - Fucic A

AU - Horvat D

LA - en

N1 - FEMU ID: 9585; EMF-Portal URL: https://www.emf-portal.org/en/article/9585

SP - 411-416

TI - Comparison of chromosome aberration and micronucleus induction in human lymphocytes after occupational exposure to vinyl chloride monomer and microwave radiation

ER -

TY - JOUR

IS - 3

JA - Radiat Res

JO - Radiation Research

PY - 1990

SN - 0033-7587

VL - 123

AU - Kerbacher JJ

AU - Meltz ML

AU - Erwin DN

LA - en

N1 - FEMU ID: 3403; EMF-Portal URL: https://www.emf-portal.org/en/article/3403

SP - 311-319

TI - Influence of radiofrequency radiation on chromosome aberrations in CHO cells and its interaction with DNA-damaging agents

ER -

TY - JOUR

IS - 2

JO - Bioelectromagnetics

PY - 1990

SN - 0197-8462

VL - 11

AU - Meltz ML

AU - Eagan P

AU - Erwin DN

DO - 10.1002/bem.2250110206

LA - en

N1 - FEMU ID: 888; EMF-Portal URL: https://www.emf-portal.org/en/article/888

SP - 149-157

TI - Proflavin and microwave radiation: absence of a mutagenic interaction

ER -

TY - JOUR

IS - 3

JO - Mutation Research - Letters

PY - 1990

VL - 243

AU - Garaj-Vrhovac V

AU - Horvat D

AU - Koren Z

DO - 10.1016/0165-7992(90)90028-i

LA - en

N1 - FEMU ID: 878; EMF-Portal URL: https://www.emf-portal.org/en/article/878

SP - 87-93

TI - The effect of microwave radiation on the cell genome

ER -

TY - JOUR

IS - 4

JA - Environ Mol Mutagen

JO - Environmental and Molecular Mutagenesis

PY - 1989

SN - 0893-6692

VL - 13

AU - Meltz ML

AU - Eagan P

AU - Erwin DN

DO - 10.1002/em.2850130404

LA - en

N1 - FEMU ID: 932; EMF-Portal URL: https://www.emf-portal.org/en/article/932

SP - 294-303

TI - Absence of mutagenic interaction between microwaves and mitomycin C in mammalian cells

ER -

TY - JOUR

IS - 6

JA - Int J Radiat Biol Relat Stud Phys Chem Med

JO - International Journal of Radiation Biology and Related Studies in Physics, Chemistry and Medicine

PY - 1988

SN - 0020-7616

VL - 53

AU - Saunders RD

AU - Kowalczuk CI

AU - Beechey CV

AU - Dunford R

DO - 10.1080/09553008814551341

LA - en

N1 - FEMU ID: 1408; EMF-Portal URL: https://www.emf-portal.org/en/article/1408

SP - 983-992

TI - Studies of the induction of dominant lethals and translocations in male mice after chronic exposure to microwave radiation

ER -

TY - JOUR

IS - 3

JA - Int J Biometeorol

JO - International Journal of Biometeorology

PY - 1987

SN - 0020-7128

VL - 31

AU - Levengood WC

DO - 10.1007/BF02188921

LA - en

N1 - FEMU ID: 2114; EMF-Portal URL: https://www.emf-portal.org/en/article/2114

SP - 185-190

TI - Non-disjunction mutations in Drosophila exposed to magnetic fields

ER -

TY - JOUR

IS - 2

JA - Radiat Res

JO - Radiation Research

PY - 1987

SN - 0033-7587

VL - 110

AU - Sagripanti JL

AU - Swicord ML

AU - Davis CC

DO - 10.2307/3576900

LA - en

N1 - FEMU ID: 2077; EMF-Portal URL: https://www.emf-portal.org/en/article/2077

SP - 219-231

TI - Microwave effects on plasmid DNA

ER -

TY - JOUR

IS - 4

JA - Environ Mutagen

JO - Environmental Mutagenesis

PY - 1987

SN - 0192-2521

VL - 9

AU - Ciaravino V

AU - Meltz ML

AU - Erwin DN

DO - 10.1002/em.2860090405

LA - en

N1 - FEMU ID: 2060; EMF-Portal URL: https://www.emf-portal.org/en/article/2060

SP - 393-399

TI - Effects of radiofrequency radiation and simultaneous exposure with mitomycin C on the frequency of sister chromatid exchanges in Chinese hamster ovary cells

ER -

TY - JOUR

IS - 1

JA - Int J Radiat Biol Relat Stud Phys Chem Med

JO - International Journal of Radiation Biology and Related Studies in Physics, Chemistry and Medicine

PY - 1986

SN - 0020-7616

VL - 50

AU - Sagripanti JL

AU - Swicord ML

DO - 10.1080/09553008614550431

LA - en

N1 - FEMU ID: 7147; EMF-Portal URL: https://www.emf-portal.org/en/article/7147

SP - 47-50

TI - DNA structural changes caused by microwave radiation

ER -

TY - JOUR

IS - 5

JA - Int J Radiat Biol Relat Stud Phys Chem Med

JO - International Journal of Radiation Biology and Related Studies in Physics, Chemistry and Medicine

PY - 1986

SN - 0020-7616

VL - 50

AU - Beechey CV

AU - Brooker D

AU - Kowalczuk CI

AU - Saunders RD

AU - Searle AG

DO - 10.1080/09553008614551321

LA - en

N1 - FEMU ID: 1388; EMF-Portal URL: https://www.emf-portal.org/en/article/1388

SP - 909-918

TI - Cytogenetic effects of microwave irradiation on male germ cells of the mouse

ER -

TY - JOUR

IS - 2

JO - Bioelectromagnetics

PY - 1986

SN - 0197-8462

VL - 7

AU - Lloyd DC

AU - Saunders RD

AU - Moquet JE

AU - Kowalczuk CI

DO - 10.1002/bem.2250070212

LA - en

N1 - FEMU ID: 893; EMF-Portal URL: https://www.emf-portal.org/en/article/893

SP - 235-237

TI - Absence of chromosomal damage in human lymphocytes exposed to microwave radiation with hyperthermia

ER -

TY - JOUR

IS - 2-3

JO - Mutation Research - Genetic Toxicology

PY - 1985

VL - 157

AU - Marec F

AU - Ondracek J

AU - Brunnhofer V

DO - 10.1016/0165-1218(85)90112-0

LA - en

N1 - FEMU ID: 2121; EMF-Portal URL: https://www.emf-portal.org/en/article/2121

SP - 163-167

TI - The effect of repeated microwave irradiation on the frequency of sex-linked recessive lethal mutations in Drosophila melanogaster

ER -

TY - JOUR

IS - 6

JA - Int J Radiat Biol Relat Stud Phys Chem Med

JO - International Journal of Radiation Biology and Related Studies in Physics, Chemistry and Medicine

PY - 1985

SN - 0020-7616

VL - 48

AU - Dardalhon M

AU - Averbeck D

AU - Berteaud AJ

AU - Ravary V

DO - 10.1080/09553008514552111

LA - en

N1 - FEMU ID: 2043; EMF-Portal URL: https://www.emf-portal.org/en/article/2043

SP - 987-996

TI - Thermal aspects of biological effects of microwaves in Saccharomyces cerevisiae

ER -

TY - JOUR

IS - 1

JA - J Hered

JO - The Journal of Heredity

PY - 1985

SN - 0022-1503

VL - 76

AU - Manikowska-Czerska E

AU - Czerski P

AU - Leach WM

DO - 10.1093/oxfordjournals.jhered.a110027

LA - en

N1 - FEMU ID: 922; EMF-Portal URL: https://www.emf-portal.org/en/article/922

SP - 71-73

TI - Effects of 2.45 GHz microwaves on meiotic chromosomes of male CBA/CAY mice

ER -

TY - JOUR

IS - 2

JA - Int J Radiat Biol Relat Stud Phys Chem Med

JO - International Journal of Radiation Biology and Related Studies in Physics, Chemistry and Medicine

PY - 1984

SN - 0020-7616

VL - 46

AU - Lloyd DC

AU - Saunders RD

AU - Finnon P

AU - Kowalczuk CI

DO - 10.1080/09553008414551211

LA - en

N1 - FEMU ID: 13126; EMF-Portal URL: https://www.emf-portal.org/en/article/13126

SP - 135-141

TI - No clastogenic effect from in vitro microwave irradiation of G0 human lymphocytes

ER -

TY - JOUR

IS - 3-4

JO - Mutation Research - Genetic Toxicology

PY - 1983

VL - 117

AU - Saunders RD

AU - Darby SC

AU - Kowalczuk CI

LA - en

N1 - FEMU ID: 10386; EMF-Portal URL: https://www.emf-portal.org/en/article/10386

SP - 345-356

TI - Dominant lethal studies in male mice after exposure to 2.45 GHz microwave radiation

ER -

TY - JOUR

IS - 1

JO - Hereditas

PY - 1983

SN - 0018-0661

VL - 98

AU - Anderstam B

AU - Hamnerius Y

AU - Hussain S

AU - Ehrenberg L

DO - 10.1111/j.1601-5223.1983.tb00575.x

LA - en

N1 - FEMU ID: 2691; EMF-Portal URL: https://www.emf-portal.org/en/article/2691

SP - 11-32

TI - Studies of possible genetic effects in bacteria of high frequency electromagnetic fields

UR - http://onlinelibrary.wiley.com/doi/10.1111/j.1601-5223.1983.tb00575.x/epdf

ER -

TY - JOUR

IS - 2

JO - Mutation Research - Letters

PY - 1983

VL - 122

AU - Kowalczuk CI

AU - Saunders RD

AU - Stapleton HR

DO - 10.1016/0165-7992(83)90054-4

LA - en

N1 - FEMU ID: 2034; EMF-Portal URL: https://www.emf-portal.org/en/article/2034

SP - 155-161

TI - Sperm count and sperm abnormality in male mice after exposure to 2.45 GHz microwave radiation

ER -

TY - JOUR

IS - 1

JO - Mutation Research - Letters

PY - 1982

VL - 103

AU - Goud SN

AU - Rani MV

AU - Reddy PP

AU - Reddi OS

AU - Rao MS

AU - Saxena VK

DO - 10.1016/0165-7992(82)90084-7

LA - en

N1 - FEMU ID: 2120; EMF-Portal URL: https://www.emf-portal.org/en/article/2120

SP - 39-42

TI - Genetic effects of microwave radiation in mice

ER -

TY - JOUR

IS - 4

JA - J Microw Power

JO - The Journal of Microwave Power

PY - 1982

SN - 0022-2739

VL - 17

AU - Dhahi SJ

AU - Habash RW

AU - Al-Hafid HT

DO - 10.1080/16070658.1982.11689289

LA - en

N1 - FEMU ID: 2106; EMF-Portal URL: https://www.emf-portal.org/en/article/2106

SP - 345-351

TI - Lack of mutagenic effects on conidia of Aspergillus amstelodami irradiated by 8.7175 GHz CW microwaves

ER -

TY - JOUR

IS - 2

JA - J Hered

JO - The Journal of Heredity

PY - 1982

SN - 0022-1503

VL - 73

AU - Yao KT

DO - 10.1093/oxfordjournals.jhered.a109596

LA - en

N1 - FEMU ID: 927; EMF-Portal URL: https://www.emf-portal.org/en/article/927

SP - 133-138

TI - Cytogenetic consequences of microwave irradiation on mammalian cells incubated in vitro

ER -

TY - JOUR

IS - 1

JA - Radiat Environ Biophys

JO - Radiation and Environmental Biophysics

PY - 1981

SN - 0301-634X

VL - 20

AU - Dardalhon M

AU - Averbeck D

AU - Berteaud AJ

DO - 10.1007/BF01323925

LA - en

N1 - FEMU ID: 2044; EMF-Portal URL: https://www.emf-portal.org/en/article/2044

SP - 37-51

TI - Studies on possible genetic effects of microwaves in procaryotic and eucaryotic cells

ER -

TY - JOUR

IS - 2

JA - Radiat Res

JO - Radiation Research

PY - 1981

SN - 0033-7587

VL - 85

AU - McRee DI

AU - MacNichols G

LA - en

N1 - FEMU ID: 1399; EMF-Portal URL: https://www.emf-portal.org/en/article/1399

SP - 340-348

TI - Incidence of sister chromatid exchange in bone marrow cells of the mouse following microwave exposure

ER -

TY - JOUR

IS - 2

JA - J Microw Power

JO - The Journal of Microwave Power

PY - 1980

SN - 0022-2739

VL - 15

AU - Mezykowski T

AU - Bal J

AU - Debiec H

AU - Kwarecki K

LA - en

N1 - FEMU ID: 9751; EMF-Portal URL: https://www.emf-portal.org/en/article/9751

SP - 75-80

TI - Response of Aspergillus nidulans and Physarum polycephalum to microwave irradiation

ER -

TY - JOUR

IS - 3

JA - Radiat Res

JO - Radiation Research

PY - 1980

SN - 0033-7587

VL - 82

AU - Blevins RD

AU - Crenshaw Jr RC

AU - Hougland AE

AU - Clark CE

LA - en

N1 - FEMU ID: 9259; EMF-Portal URL: https://www.emf-portal.org/en/article/9259

SP - 511-517

TI - The effects of microwave radiation and heat on specific mutants of Salmonella typhimurium LT2

ER -

TY - JOUR

IS - 5-6

JA - J Environ Pathol Toxicol

JO - Journal of Environmental Pathology and Toxicology

PY - 1980

SN - 0146-4779

VL - 3

AU - Dutta SK

AU - Nelson WH

AU - Blackman CF

AU - Brusick DJ

LA - en

N1 - FEMU ID: 5670; EMF-Portal URL: https://www.emf-portal.org/en/article/5670

SP - 195-206

TI - Cellular effects in microbial tester strains caused by exposure to microwaves or elevated temperatures

ER -

TY - JOUR

IS - 1

JO - Bioelectromagnetics

PY - 1980

SN - 0197-8462

VL - 1

AU - Berman E

AU - Carter HB

AU - House D

DO - 10.1002/bem.2250010107

LA - en

N1 - FEMU ID: 2022; EMF-Portal URL: https://www.emf-portal.org/en/article/2022

SP - 65-76

TI - Tests of mutagenesis and reproduction in male rats exposed to 2,450-MHz (CW) microwaves

ER -

TY - JOUR

IS - 3

JO - Experientia

PY - 1979

SN - 0014-4754

VL - 35

AU - Manikowska E

AU - Luciani JM

AU - Servantie B

AU - Czerski P

AU - Obrenovitch J

AU - Stahl A

DO - 10.1007/BF01964370

LA - en

N1 - FEMU ID: 9731; EMF-Portal URL: https://www.emf-portal.org/en/article/9731

SP - 388-390

TI - Effects of 9.4 GHz microwave exposure on meiosis in mice

ER -

TY - JOUR

IS - 4

JA - J Microw Power

JO - The Journal of Microwave Power

PY - 1979

SN - 0022-2739

VL - 14

AU - Dardalhon M

AU - Averbeck D

AU - Berteaud AJ

DO - 10.1080/16070658.1979.11689165

LA - en

N1 - FEMU ID: 7859; EMF-Portal URL: https://www.emf-portal.org/en/article/7859

SP - 307-312

TI - Determination of a thermal equivalent of millimeter microwaves in living cells

ER -

TY - JOUR

IS - 3

JA - J Microw Power

JO - The Journal of Microwave Power

PY - 1979

SN - 0022-2739

VL - 14

AU - Dutta SK

AU - Nelson WH

AU - Blackman CF

AU - Brusick DJ

DO - 10.1080/16070658.1979.11689160

LA - en

N1 - FEMU ID: 5638; EMF-Portal URL: https://www.emf-portal.org/en/article/5638

SP - 275-280

TI - Lack of microbial genetic response to 2.45-GHz CW and 8.5- to 9.6-GHz pulsed microwaves

ER -

TY - JOUR

IS - 3

JO - Mutation Research - Genetic Toxicology

PY - 1979

VL - 68

AU - Hamnerius Y

AU - Olofsson H

AU - Rasmuson A

AU - Rasmuson B

DO - 10.1016/0165-1218(79)90153-8

LA - en

N1 - FEMU ID: 2042; EMF-Portal URL: https://www.emf-portal.org/en/article/2042

SP - 217-223

TI - A Negative Test for Mutagenic Action of Microwave Radiation in Drosophila melanogaster

ER -

TY - JOUR

IS - 1

JA - Can J Genet Cytol

JO - Canadian Journal of Genetics and Cytology

PY - 1978

SN - 0008-4093

VL - 20

AU - Alam MT

AU - Barthakur N

AU - Lambert NG

AU - Kasatiya SS

DO - 10.1139/g78-004

LA - en

N1 - FEMU ID: 933; EMF-Portal URL: https://www.emf-portal.org/en/article/933

SP - 23-30

TI - Cytological effects of microwave radiation in Chinese hamster cells in vitro

ER -

TY - JOUR

IS - 4

JA - J Hered

JO - The Journal of Heredity

PY - 1977

SN - 0022-1503

VL - 68

AU - Mittler S

DO - 10.1093/oxfordjournals.jhered.a108826

LA - en

N1 - FEMU ID: 1411; EMF-Portal URL: https://www.emf-portal.org/en/article/1411

SP - 257-258

TI - Failure of chronic exposure to nonthermal FM radio waves to mutate Drosophila

ER -

TY - JOUR

IS - 3

JA - Environ Res

JO - Environmental Research

PY - 1976

SN - 0013-9351

VL - 11

AU - Mittler S

DO - 10.1016/0013-9351(76)90094-3

LA - en

N1 - FEMU ID: 1410; EMF-Portal URL: https://www.emf-portal.org/en/article/1410

SP - 326-330

TI - Failure of 2- and 10-meter radio waves to induce genetic damage in Drosophila melanogaster

ER -

TY - JOUR

IS - 1

JA - Environ Lett

JO - Environmental Letters

PY - 1974

SN - 0013-9300

VL - 6

AU - Chen KM

AU - Samuel A

AU - Hoopingarner R

DO - 10.1080/00139307409437344

LA - en

N1 - FEMU ID: 7715; EMF-Portal URL: https://www.emf-portal.org/en/article/7715

SP - 37-46

TI - Chromosomal aberrations of living cells induced by microwave radiation

ER -

TY - JOUR

IS - 2

JA - Radiat Res

JO - Radiation Research

PY - 1973

SN - 0033-7587

VL - 56

AU - Hamrick PE

LA - en

N1 - FEMU ID: 2039; EMF-Portal URL: https://www.emf-portal.org/en/article/2039

SP - 400-404

TI - Letter: Thermal denaturation of DNA exposed to 2450 MHz CW microwave radiation

ER -
