# Supplementary material for: A scoping review and evidence map of radiofrequency field exposure and genotoxicity: assessing in vivo, in vitro, and epidemiological data
Source: Front Public Health. 2025 Jul 30;13:1613353. doi: 10.3389/fpubh.2025.1613353 (PMC12343714; doi:10.3389/fpubh.2025.1613353)
Supplement: Supplementary file 3 [file Data_Sheet_3.zip › Search data/EMF portal search - OS.docx]

EMF search keywords

8-Oxo-dG, 8-oxoG, 8-Hydroxy-2-Desoxyguanosin, 8-OHdG, 8-hydroxy-2-deoxyguanosine, "DNA base damage", "DNA base oxidisation"

TY - JOUR

IS - 3

JA - J Family Reprod Health

JO - Journal of Family & Reproductive Health

PY - 2021

SN - 1735-9392

VL - 15

AU - Moghadasi N

AU - Alimohammadi I

AU - Safari Variani A

AU - Ashtarinezhad A

DO - 10.18502/jfrh.v15i3.7134

LA - en

N1 - FEMU ID: 45935; EMF-Portal URL: https://www.emf-portal.org/en/article/45935

SP - 172-178

TI - The Effect of Mobile Radiation on the Oxidative Stress Biomarkers in Pregnant Mice

UR - https://jfrh.tums.ac.ir/index.php/jfrh/article/view/1634/568

ER -

TY - JOUR

IS - 1

JO - Bioelectromagnetics

PY - 2021

SN - 0197-8462

VL - 42

AU - Alkis ME

AU - Akdag MZ

AU - Dasdag S

DO - 10.1002/bem.22315

LA - en

N1 - FEMU ID: 43930; EMF-Portal URL: https://www.emf-portal.org/en/article/43930

SP - 76-85

TI - Effects of Low-Intensity Microwave Radiation on Oxidant-Antioxidant Parameters and DNA Damage in the Liver of Rats

ER -

TY - JOUR

IS - 1

JA - Biotechnol Biotechnol Equip

JO - Biotechnology & Biotechnological Equipment

PY - 2020

SN - 1310-2818

VL - 34

AU - Bektas H

AU - Dasdag S

AU - Bektas MS

DO - 10.1080/13102818.2020.1725639

LA - en

N1 - FEMU ID: 43422; EMF-Portal URL: https://www.emf-portal.org/en/article/43422

SP - 154-162

TI - Comparison of effects of 2.4 GHz Wi-Fi and mobile phone exposure on human placenta and cord blood

UR - https://www.tandfonline.com/doi/abs/10.1080/13102818.2020.1725639?needAccess=true#aHR0cHM6Ly93d3cudGFuZGZvbmxpbmUuY29tL2RvaS9wZGYvMTAuMTA4MC8xMzEwMjgxOC4yMDIwLjE3MjU2Mzk/bmVlZEFjY2Vzcz10cnVlQEBAMA==

ER -

TY - JOUR

IS - 1

JA - Biotechnol Biotechnol Equip

JO - Biotechnology & Biotechnological Equipment

PY - 2019

SN - 1310-2818

VL - 33

AU - Alkis ME

AU - Akdag MZ

AU - Dasdag S

AU - Yegin K

AU - Akpolat V

DO - 10.1080/13102818.2019.1696702

LA - en

N1 - FEMU ID: 43430; EMF-Portal URL: https://www.emf-portal.org/en/article/43430

SP - 1733-1740

TI - Single-strand DNA breaks and oxidative changes in rat testes exposed to radiofrequency radiation emitted from cellular phones

UR - https://www.tandfonline.com/doi/abs/10.1080/13102818.2019.1696702?needAccess=true#aHR0cHM6Ly93d3cudGFuZGZvbmxpbmUuY29tL2RvaS9wZGYvMTAuMTA4MC8xMzEwMjgxOC4yMDE5LjE2OTY3MDI/bmVlZEFjY2Vzcz10cnVlQEBAMA==

ER -

TY - JOUR

IS - 6

JA - Biotech Histochem

JO - Biotechnic & Histochemistry

PY - 2019

SN - 1052-0295

VL - 94

AU - Okatan DÖ

AU - Kulaber A

AU - Kerimoglu G

AU - Odaci E

DO - 10.1080/10520295.2019.1580767

LA - en

N1 - FEMU ID: 38102; EMF-Portal URL: https://www.emf-portal.org/en/article/38102

SP - 420-428

TI - Altered morphology and biochemistry of the female rat liver following 900 megahertz electromagnetic field exposure during mid to late adolescence

ER -

TY - JOUR

IS - 1

JA - Electromagn Biol Med

JO - Electromagnetic Biology and Medicine

PY - 2019

SN - 1536-8386

VL - 38

AU - Alkis ME

AU - Bilgin HM

AU - Akpolat V

AU - Dasdag S

AU - Yegin K

AU - Yavas MC

AU - Akdag MZ

DO - 10.1080/15368378.2019.1567526

LA - en

N1 - FEMU ID: 37161; EMF-Portal URL: https://www.emf-portal.org/en/article/37161

SP - 32-47

TI - Effect of 900-, 1800-, and 2100-MHz radiofrequency radiation on DNA and oxidative stress in brain

ER -

TY - JOUR

IS - 4

JA - Chin Med J

JO - Chinese Medical Journal

PY - 2018

SN - 0366-6999

VL - 131

AU - Ding SS

AU - Sun P

AU - Zhang Z

AU - Liu X

AU - Tian H

AU - Huo YW

AU - Wang LR

AU - Han Y

AU - Xing JP

DO - 10.4103/0366-6999.225045

LA - en

N1 - FEMU ID: 49146; EMF-Portal URL: https://www.emf-portal.org/en/article/49146

SP - 402-412

TI - Moderate Dose of Trolox Preventing the Deleterious Effects of Wi-Fi Radiation on Spermatozoa In vitro through Reduction of Oxidative Stress Damage

UR - https://journals.lww.com/cmj/Fulltext/2018/02200/Moderate_Dose_of_Trolox_Preventing_the_Deleterious.4.aspx

ER -

TY - JOUR

IS - 3

JA - Int J Clin Exp Med

JO - International Journal of Clinical and Experimental Medicine

PY - 2018

SN - 1940-5901

VL - 11

AU - Ding SS

AU - Sun P

AU - Tian H

AU - Huo YW

AU - Wang LR

AU - Han Y

AU - Zhang Z

AU - Liu X

AU - Xing JP

LA - en

N1 - FEMU ID: 49145; EMF-Portal URL: https://www.emf-portal.org/en/article/49145

SP - 2821-2830

TI - Association between daily exposure to electromagnetic radiation from 4G smartphone and 2.45-GHz wi-fi and oxidative damage to semen of males attending a genetics clinic: a primary study

UR - https://e-century.us/files/ijcem/11/3/ijcem0063001.pdf

ER -

TY - JOUR

IS - 4

JA - Exp Oncol

JO - Experimental Oncology

PY - 2018

SN - 1812-9269

VL - 40

AU - Yakymenko I

AU - Burlaka A

AU - Tsybulin I

AU - Brieieva I

AU - Buchynska L

AU - Tsehmistrenko I

AU - Chekhun F

LA - en

N1 - FEMU ID: 36998; EMF-Portal URL: https://www.emf-portal.org/en/article/36998

SP - 282-287

TI - Oxidative and mutagenic effects of low intensity GSM 1800 MHz microwave radiation

UR - https://exp-oncology.com.ua/wp/wp-content/uploads/2018/12/2458.pdf?upload=

ER -

TY - JOUR

JA - Front Public Health

JO - Frontiers in Public Health

PY - 2018

SN - 2296-2565

VL - 6

AU - Houston BJ

AU - Nixon B

AU - King BV

AU - Aitken RJ

AU - De Iuliis GN

DO - 10.3389/fpubh.2018.00270

LA - en

N1 - FEMU ID: 36249; EMF-Portal URL: https://www.emf-portal.org/en/article/36249

SP - 270

TI - Probing the Origins of 1,800 MHz Radio Frequency Electromagnetic Radiation Induced Damage in Mouse Immortalized Germ Cells and Spermatozoa in vitro

UR - https://www.frontiersin.org/articles/10.3389/fpubh.2018.00270/pdf

ER -

TY - JOUR

IS - 7

JA - Int J Mol Sci

JO - International Journal of Molecular Sciences

PY - 2018

SN - 1422-0067

VL - 19

AU - Jeong YJ

AU - Son Y

AU - Han NK

AU - Choi HD

AU - Pack JK

AU - Kim N

AU - Lee YS

AU - Lee HJ

DO - 10.3390/ijms19072103

LA - en

N1 - FEMU ID: 35546; EMF-Portal URL: https://www.emf-portal.org/en/article/35546

TI - Impact of Long-Term RF-EMF on Oxidative Stress and Neuroinflammation in Aging Brains of C57BL/6 Mice

UR - http://www.mdpi.com/1422-0067/19/7/2103/pdf

ER -

TY - JOUR

JO - Mutation Research - Fundamental and Molecular Mechanism of Mutagenesis

PY - 2017

SN - 0027-5107

VL - 797-799

AU - Sun Y

AU - Zong L

AU - Gao Z

AU - Zhu S

AU - Tong J

AU - Cao Y

DO - 10.1016/j.mrfmmm.2017.03.001

LA - en

N1 - FEMU ID: 31583; EMF-Portal URL: https://www.emf-portal.org/en/article/31583

SP - 7-14

TI - Mitochondrial DNA damage and oxidative damage in HL-60 cells exposed to 900MHz radiofrequency fields

ER -

TY - JOUR

IS - 6

JO - Bioelectromagnetics

PY - 2016

SN - 0197-8462

VL - 37

AU - Nakatani-Enomoto S

AU - Okutsu M

AU - Suzuki S

AU - Suganuma R

AU - Groiss SJ

AU - Kadowaki S

AU - Enomoto H

AU - Fujimori K

AU - Ugawa Y

DO - 10.1002/bem.21985

LA - en

N1 - FEMU ID: 29652; EMF-Portal URL: https://www.emf-portal.org/en/article/29652

SP - 373-381

TI - Effects of 1950 MHz W-CDMA-like signal on human spermatozoa

ER -

TY - JOUR

JA - J Chem Neuroanat

JO - Journal of Chemical Neuroanatomy

PY - 2016

SN - 0891-0618

VL - 75

AU - Sahin D

AU - Ozgur E

AU - Guler G

AU - Tomruk A

AU - Unlu I

AU - Sepici-Dincel A

AU - Seyhan N

DO - 10.1016/j.jchemneu.2016.01.002

LA - en

N1 - FEMU ID: 28658; EMF-Portal URL: https://www.emf-portal.org/en/article/28658

SP - 94-98

TI - The 2100 MHz radiofrequency radiation of a 3G-mobile phone and the DNA oxidative damage in brain

ER -

TY - JOUR

JA - J Chem Neuroanat

JO - Journal of Chemical Neuroanatomy

PY - 2016

SN - 0891-0618

VL - 75

AU - Guler G

AU - Ozgur E

AU - Keles H

AU - Tomruk A

AU - Vural SA

AU - Seyhan N

DO - 10.1016/j.jchemneu.2015.10.006

LA - en

N1 - FEMU ID: 28169; EMF-Portal URL: https://www.emf-portal.org/en/article/28169

SP - 128-133

TI - Neurodegenerative changes and apoptosis induced by intrauterine and extrauterine exposure of radiofrequency radiation

ER -

TY - JOUR

IS - 3

JA - Cell Physiol Biochem

JO - Cellular Physiology and Biochemistry

PY - 2015

SN - 1015-8987

VL - 37

AU - Wang X

AU - Liu C

AU - Ma Q

AU - Feng W

AU - Yang L

AU - Lu Y

AU - Zhou Z

AU - Yu Z

AU - Li W

AU - Zhang L

DO - 10.1159/000430233

LA - en

N1 - FEMU ID: 27920; EMF-Portal URL: https://www.emf-portal.org/en/article/27920

SP - 1075-1088

TI - 8-oxoG DNA Glycosylase-1 Inhibition Sensitizes Neuro-2a Cells to Oxidative DNA Base Damage Induced by 900 MHz Radiofrequency Electromagnetic Radiation

UR - https://www.karger.com/Article/Pdf/430233

ER -

TY - JOUR

IS - 3

JA - Radiat Res

JO - Radiation Research

PY - 2015

SN - 0033-7587

VL - 183

AU - Duan W

AU - Liu C

AU - Zhang L

AU - He M

AU - Xu S

AU - Chen C

AU - Pi H

AU - Gao P

AU - Zhang Y

AU - Zhong M

AU - Yu Z

AU - Zhou Z

DO - 10.1667/RR13851.1

LA - en

N1 - FEMU ID: 26541; EMF-Portal URL: https://www.emf-portal.org/en/article/26541

SP - 305-314

TI - Comparison of the genotoxic effects induced by 50 Hz extremely low-frequency electromagnetic fields and 1800 MHz radiofrequency electromagnetic fields in GC-2 cells

ER -

TY - JOUR

IS - 10

JA - Int J Radiat Biol

JO - International Journal of Radiation Biology

PY - 2014

SN - 0955-3002

VL - 90

AU - Gürler HS

AU - Bilgici B

AU - Akar AK

AU - Tomak L

AU - Bedir A

DO - 10.3109/09553002.2014.922717

LA - en

N1 - FEMU ID: 24955; EMF-Portal URL: https://www.emf-portal.org/en/article/24955

SP - 892-896

TI - Increased DNA oxidation (8-OHdG) and protein oxidation (AOPP) by Low level electromagnetic field (2.45 GHz) in rat brain and protective effect of garlic

ER -

TY - JOUR

IS - 2

JA - Electromagn Biol Med

JO - Electromagnetic Biology and Medicine

PY - 2014

SN - 1536-8386

VL - 33

AU - Khalil AM

AU - Abu Khadra KM

AU - Aljaberi AM

AU - Gagaa MH

AU - Issa HS

DO - 10.3109/15368378.2013.783855

LA - en

N1 - FEMU ID: 22875; EMF-Portal URL: https://www.emf-portal.org/en/article/22875

SP - 92-97

TI - Assessment of oxidant/antioxidant status in saliva of cell phone users

ER -

TY - JOUR

JA - Reprod Toxicol

JO - Reproductive Toxicology

PY - 2013

SN - 0890-6238

VL - 42

AU - Hanci H

AU - Odaci E

AU - Kaya H

AU - Aliyazicioglu Y

AU - Turan I

AU - Demir S

AU - Colakoglu S

DO - 10.1016/j.reprotox.2013.09.006

LA - en

N1 - FEMU ID: 23648; EMF-Portal URL: https://www.emf-portal.org/en/article/23648

SP - 203-209

TI - The effect of prenatal exposure to 900-megahertz electromagnetic field on the 21-old-day rat testicle

ER -

TY - JOUR

IS - 3

JA - Exp Oncol

JO - Experimental Oncology

PY - 2013

SN - 1812-9269

VL - 35

AU - Burlaka A

AU - Tsybulin O

AU - Sidorik E

AU - Lukin S

AU - Polishuk V

AU - Tsehmistrenko S

AU - Yakymenko I

LA - en

N1 - FEMU ID: 23628; EMF-Portal URL: https://www.emf-portal.org/en/article/23628

SP - 219-225

TI - Overproduction of free radical species in embryonal cells exposed to low intensity radiofrequency radiation

UR - https://exp-oncology.com.ua/article/6079

ER -

TY - JOUR

IS - 1

JA - Toxicol Lett

JO - Toxicology Letters

PY - 2013

SN - 0378-4274

VL - 218

AU - Liu C

AU - Duan W

AU - Xu S

AU - Chen C

AU - He M

AU - Zhang L

AU - Yu Z

AU - Zhou Z

DO - 10.1016/j.toxlet.2013.01.003

LA - en

N1 - FEMU ID: 21674; EMF-Portal URL: https://www.emf-portal.org/en/article/21674

SP - 2-9

TI - Exposure to 1800 MHz radiofrequency electromagnetic radiation induces oxidative DNA base damage in a mouse spermatocyte-derived cell line

ER -

TY - JOUR

IS - 2

JA - J Pediatr Urol

JO - Journal of Pediatric Urology

PY - 2013

SN - 1477-5131

VL - 9

AU - Atasoy HI

AU - Gunal MY

AU - Atasoy P

AU - Elgun S

AU - Bugdayci G

DO - 10.1016/j.jpurol.2012.02.015

LA - en

N1 - FEMU ID: 20439; EMF-Portal URL: https://www.emf-portal.org/en/article/20439

SP - 223-229

TI - Immunohistopathologic demonstration of deleterious effects on growing rat testes of radiofrequency waves emitted from conventional Wi-Fi devices

ER -

TY - JOUR

IS - 7

JA - Hum Exp Toxicol

JO - Human & Experimental Toxicology

PY - 2012

SN - 0960-3271

VL - 31

AU - Khalil AM

AU - Gagaa MH

AU - Alshamali AM

DO - 10.1177/0960327111433184

LA - en

N1 - FEMU ID: 20105; EMF-Portal URL: https://www.emf-portal.org/en/article/20105

SP - 734-740

TI - 8-Oxo-7, 8-dihydro-2'-deoxyguanosine as a biomarker of DNA damage by mobile phone radiation

ER -

TY - JOUR

IS - 4

JA - Int J Radiat Biol

JO - International Journal of Radiation Biology

PY - 2012

SN - 0955-3002

VL - 88

AU - Guler G

AU - Tomruk A

AU - Ozgur E

AU - Sahin D

AU - Sepici A

AU - Altan N

AU - Seyhan N

DO - 10.3109/09553002.2012.646349

LA - en

N1 - FEMU ID: 19950; EMF-Portal URL: https://www.emf-portal.org/en/article/19950

SP - 367-373

TI - The effect of radiofrequency radiation on DNA and lipid damage in female and male infant rabbits

ER -

TY - JOUR

JA - World Acad Sci Eng Technol

JO - World Academy of Science, Engineering and Technology

PY - 2011

VL - 76

AU - Khalil AM

AU - Alshamali AM

AU - Gagaa MH

LA - en

N1 - FEMU ID: 20110; EMF-Portal URL: https://www.emf-portal.org/en/article/20110

SP - 657-622

TI - Detection of oxidative stress induced by mobile phone radiation in tissues of mice using 8-oxo-7, 8-dihydro-20-deoxyguanosine as a biomarker

UR - http://publications.waset.org/7054/pdf

ER -

TY - JOUR

IS - 1

JA - Gen Physiol Biophys

JO - General Physiology and Biophysics

PY - 2010

SN - 0231-5882

VL - 29

AU - Güler G

AU - Tomruk A

AU - Ozgur E

AU - Seyhan N

DO - 10.4149/gpb_2010_01_59

LA - en

N1 - FEMU ID: 18104; EMF-Portal URL: https://www.emf-portal.org/en/article/18104

SP - 59-66

TI - The effect of radiofrequency radiation on DNA and lipid damage in non-pregnant and pregnant rabbits and their newborns

ER -

TY - JOUR

JA - Brain Res

JO - Brain Research

PY - 2010

SN - 0006-8993

VL - 1311

AU - Xu S

AU - Zhou Z

AU - Zhang L

AU - Yu Z

AU - Zhang W

AU - Wang Y

AU - Wang X

AU - Li M

AU - Chen Y

AU - Chen C

AU - He M

AU - Zhang G

AU - Zhong M

DO - 10.1016/j.brainres.2009.10.062

LA - en

N1 - FEMU ID: 17674; EMF-Portal URL: https://www.emf-portal.org/en/article/17674

SP - 189-196

TI - Exposure to 1800 MHz radiofrequency radiation induces oxidative damage to mitochondrial DNA in primary cultured neurons

ER -

TY - JOUR

IS - 1

JA - Cell Biochem Biophys

JO - Cell Biochemistry and Biophysics

PY - 2010

SN - 1085-9195

VL - 56

AU - Tomruk A

AU - Güler G

AU - Dincel AS

DO - 10.1007/s12013-009-9068-1

LA - en

N1 - FEMU ID: 17640; EMF-Portal URL: https://www.emf-portal.org/en/article/17640

SP - 39-47

TI - The influence of 1800 MHz GSM-like signals on hepatic oxidative DNA and lipid damage in nonpregnant, pregnant, and newly born rabbits

ER -

TY - JOUR

IS - 7

JO - PLoS One

PY - 2009

SN - 1932-6203

VL - 4

AU - De Iuliis GN

AU - Newey RJ

AU - King BV

AU - Aitken RJ

DO - 10.1371/journal.pone.0006446

LA - en

N1 - FEMU ID: 17394; EMF-Portal URL: https://www.emf-portal.org/en/article/17394

SP - e6446

TI - Mobile phone radiation induces reactive oxygen species production and DNA damage in human spermatozoa in vitro

UR - https://journals.plos.org/plosone/article/file?id=10.1371/journal.pone.0006446&type=printable

ER -
