# Supplementary material for: A scoping review and evidence map of radiofrequency field exposure and genotoxicity: assessing in vivo, in vitro, and epidemiological data
Source: Front Public Health. 2025 Jul 30;13:1613353. doi: 10.3389/fpubh.2025.1613353 (PMC12343714; doi:10.3389/fpubh.2025.1613353)
Supplement: Supplementary file 3 [file Data_Sheet_3.zip › Search data/EMF Portal Search - ploidy.docx]

EMF Portal search keywords

The following terms were included: aneuploidy, Aneuploidie, 異数体, polyploid, 倍数体

TY - JOUR

IS - 1

JA - Health Phys

JO - Health Physics

PY - 2018

SN - 0017-9078

VL - 115

AU - Franchini V

AU - Regalbuto E

AU - De Amicis A

AU - De Sanctis S

AU - Di Cristofaro S

AU - Coluzzi E

AU - Marinaccio J

AU - Sgura A

AU - Ceccuzzi S

AU - Doria A

AU - Gallerano GP

AU - Giovenale E

AU - Ravera GL

AU - Bei R

AU - Benvenuto M

AU - Modesti A

AU - Masuelli L

AU - Lista F

DO - 10.1097/HP.0000000000000871

LA - en

N1 - FEMU ID: 35185; EMF-Portal URL: https://www.emf-portal.org/en/article/35185

SP - 126-139

TI - Genotoxic Effects in Human Fibroblasts Exposed to Microwave Radiation

ER -

TY - JOUR

IS - 6

JA - Environ Mol Mutagen

JO - Environmental and Molecular Mutagenesis

PY - 2018

SN - 0893-6692

VL - 59

AU - Franchini V

AU - De Sanctis S

AU - Marinaccio J

AU - De Amicis A

AU - Coluzzi E

AU - Di Cristofaro S

AU - Lista F

AU - Regalbuto E

AU - Doria A

AU - Giovenale E

AU - Gallerano GP

AU - Bei R

AU - Benvenuto M

AU - Masuelli L

AU - Udroiu I

AU - Sgura A

DO - 10.1002/em.22192

LA - en

N1 - FEMU ID: 34868; EMF-Portal URL: https://www.emf-portal.org/en/article/34868

SP - 476-487

TI - Study of the effects of 0.15 terahertz radiation on genome integrity of adult fibroblasts

UR - https://onlinelibrary.wiley.com/doi/epdf/10.1002/em.22192

ER -

TY - JOUR

JA - Mutat Res Genet Toxicol Environ Mutagen

JO - Mutation Research - Genetic Toxicology and Environmental Mutagenesis

PY - 2015

VL - 793

AU - Amicis A

AU - Sanctis S

AU - Cristofaro SD

AU - Franchini V

AU - Lista F

AU - Regalbuto E

AU - Giovenale E

AU - Gallerano GP

AU - Nenzi P

AU - Bei R

AU - Fantini M

AU - Benvenuto M

AU - Masuelli L

AU - Coluzzi E

AU - Cicia C

AU - Sgura A

DO - 10.1016/j.mrgentox.2015.06.003

LA - en

N1 - FEMU ID: 28170; EMF-Portal URL: https://www.emf-portal.org/en/article/28170

SP - 150-160

TI - Biological effects of in vitro THz radiation exposure in human foetal fibroblasts

ER -

TY - JOUR

IS - 1-2

JA - Mutat Res Genet Toxicol Environ Mutagen

JO - Mutation Research - Genetic Toxicology and Environmental Mutagenesis

PY - 2013

VL - 750

AU - Pesnya DS

AU - Romanovsky AV

DO - 10.1016/j.mrgentox.2012.08.010

LA - en

N1 - FEMU ID: 21320; EMF-Portal URL: https://www.emf-portal.org/en/article/21320

SP - 27-33

TI - Comparison of cytotoxic and genotoxic effects of plutonium-239 alpha particles and mobile phone GSM 900 radiation in the Allium cepa test

ER -

TY - JOUR

IS - 5

JA - Radiat Res

JO - Radiation Research

PY - 2011

SN - 0033-7587

VL - 175

AU - Hintzsche H

AU - Jastrow C

AU - Kleine-Ostmann T

AU - Stopper H

AU - Schmid E

AU - Schrader T

DO - 10.1667/RR2406.1

LA - en

N1 - FEMU ID: 19081; EMF-Portal URL: https://www.emf-portal.org/en/article/19081

SP - 569-574

TI - Terahertz radiation induces spindle disturbances in human-hamster hybrid cells

ER -

TY - JOUR

IS - 4

JA - Int J Radiat Biol

JO - International Journal of Radiation Biology

PY - 2011

SN - 0955-3002

VL - 87

AU - Bourthoumieu S

AU - Terro F

AU - Leveque P

AU - Collin A

AU - Joubert V

AU - Yardin C

DO - 10.3109/09553002.2011.542543

LA - en

N1 - FEMU ID: 18946; EMF-Portal URL: https://www.emf-portal.org/en/article/18946

SP - 400-408

TI - Aneuploidy studies in human cells exposed in vitro to GSM-900 MHz radiofrequency radiation using FISH

ER -

TY - JOUR

IS - 6

JA - Radiat Res

JO - Radiation Research

PY - 2010

SN - 0033-7587

VL - 174

AU - Bourthoumieu S

AU - Joubert V

AU - Marin B

AU - Collin A

AU - Leveque P

AU - Terro F

AU - Yardin C

LA - en

N1 - FEMU ID: 18635; EMF-Portal URL: https://www.emf-portal.org/en/article/18635

SP - 712-718

TI - Cytogenetic Studies in Human Cells Exposed In Vitro to GSM-900 MHz Radiofrequency Radiation Using R-Banded Karyotyping

ER -

TY - JOUR

IS - 2

JA - Radiat Res

JO - Radiation Research

PY - 2008

SN - 0033-7587

VL - 170

AU - Korenstein-Ilan A

AU - Barbul A

AU - Hasin P

AU - Eliran A

AU - Gover A

AU - Korenstein R

DO - 10.1667/RR0944.1

LA - en

N1 - FEMU ID: 16198; EMF-Portal URL: https://www.emf-portal.org/en/article/16198

SP - 224-234

TI - Terahertz radiation increases genomic instability in human lymphocytes

ER -

TY - JOUR

IS - 1

JA - Radiat Res

JO - Radiation Research

PY - 2008

SN - 0033-7587

VL - 169

AU - Mazor R

AU - Korenstein-Ilan A

AU - Barbul A

AU - Eshet Y

AU - Shahadi A

AU - Jerby E

AU - Korenstein R

DO - 10.1667/RR0872.1

LA - en

N1 - FEMU ID: 15530; EMF-Portal URL: https://www.emf-portal.org/en/article/15530

SP - 28-37

TI - Increased levels of numerical chromosome aberrations after in vitro exposure of human peripheral blood lymphocytes to radiofrequency electromagnetic fields for 72 hours

ER -

TY - JOUR

IS - 2

JO - Bioelectromagnetics

PY - 2003

SN - 0197-8462

VL - 24

AU - Mashevich M

AU - Folkman D

AU - Kesar A

AU - Barbul A

AU - Korenstein R

AU - Jerby E

AU - Avivi L

DO - 10.1002/bem.10086

LA - en

N1 - FEMU ID: 9413; EMF-Portal URL: https://www.emf-portal.org/en/article/9413

SP - 82-90

TI - Exposure of human peripheral blood lymphocytes to electromagnetic fields associated with cellular phones leads to chromosomal instability

ER -

TY - JOUR

IS - 2

JO - Cytologia

PY - 2001

SN - 0011-4545

VL - 66

AU - Othman EO

AU - Aly MS

AU - El Nahas SM

DO - 10.1508/cytologia.66.117

LA - en

N1 - FEMU ID: 36938; EMF-Portal URL: https://www.emf-portal.org/en/article/36938

SP - 117-125

TI - Aneuploidy in workers occupationally exposed to electromagnetic field detected by FISH

UR - https://www.jstage.jst.go.jp/article/cytologia1929/66/2/66_2_117/_pdf/-char/en

ER -

TY - JOUR

IS - 4

JO - Mutation Research - Letters

PY - 1992

VL - 282

AU - Fucic A

AU - Garaj-Vrhovac V

AU - Skara M

AU - Dimitrovic B

DO - 10.1016/0165-7992(92)90133-3

LA - en

N1 - FEMU ID: 875; EMF-Portal URL: https://www.emf-portal.org/en/article/875

SP - 265-271

TI - X-rays, microwaves and vinyl chloride monomer: their clastogenic and aneugenic activity, using the micronucleus assay on human lymphocytes

ER -

TY - JOUR

IS - 3

JA - J Natl Cancer Inst

JO - Journal of the National Cancer Institute

PY - 1983

SN - 0027-8874

VL - 70

AU - Banerjee R

AU - Goldfeder A

AU - Mitra J

LA - en

N1 - FEMU ID: 9033; EMF-Portal URL: https://www.emf-portal.org/en/article/9033

SP - 517-521

TI - Sister chromatid exchanges and chromosome aberrations induced by radiosensitizing agents in bone marrow cells of treated tumor-bearing mice

ER -

TY - JOUR

IS - 2

JA - J Hered

JO - The Journal of Heredity

PY - 1982

SN - 0022-1503

VL - 73

AU - Yao KT

DO - 10.1093/oxfordjournals.jhered.a109596

LA - en

N1 - FEMU ID: 927; EMF-Portal URL: https://www.emf-portal.org/en/article/927

SP - 133-138

TI - Cytogenetic consequences of microwave irradiation on mammalian cells incubated in vitro

ER -

TY - JOUR

IS - 3

JO - Experientia

PY - 1979

SN - 0014-4754

VL - 35

AU - Manikowska E

AU - Luciani JM

AU - Servantie B

AU - Czerski P

AU - Obrenovitch J

AU - Stahl A

DO - 10.1007/BF01964370

LA - en

N1 - FEMU ID: 9731; EMF-Portal URL: https://www.emf-portal.org/en/article/9731

SP - 388-390

TI - Effects of 9.4 GHz microwave exposure on meiosis in mice

ER -
