# Supplementary material for: A scoping review and evidence map of radiofrequency field exposure and genotoxicity: assessing in vivo, in vitro, and epidemiological data
Source: Front Public Health. 2025 Jul 30;13:1613353. doi: 10.3389/fpubh.2025.1613353 (PMC12343714; doi:10.3389/fpubh.2025.1613353)
Supplement: Supplementary file 3 [file Data_Sheet_3.zip › Search data/EMF Portal Search - Sister.docx]

EMF Portal search key words

The following terms were included: “Sister Chromatid Exchange”

TY - JOUR

IS - 2

JA - Radiat Res

JO - Radiation Research

PY - 2013

SN - 0033-7587

VL - 179

AU - Waldmann P

AU - Bohnenberger S

AU - Greinert R

AU - Hermann-Then B

AU - Heselich A

AU - Klug SJ

AU - Koenig J

AU - Kuhr K

AU - Kuster N

AU - Merker M

AU - Murbach M

AU - Pollet D

AU - Schadenboeck W

AU - Scheidemann-Wesp U

AU - Schwab B

AU - Volkmer B

AU - Weyer V

AU - Blettner M

DO - 10.1667/RR2914.1

LA - en

N1 - FEMU ID: 21641; EMF-Portal URL: https://www.emf-portal.org/en/article/21641

SP - 243-253

TI - Influence of GSM Signals on Human Peripheral Lymphocytes: Study of Genotoxicity

ER -

TY - JOUR

JA - Sci Total Environ

JO - Science of the Total Evironment

PY - 2011

SN - 0048-9697

VL - 410

AU - Esmekaya MA

AU - Aytekin E

AU - Ozgur E

AU - Güler G

AU - Ergun MA

AU - Omeroglu S

AU - Seyhan N

DO - 10.1016/j.scitotenv.2011.09.036

LA - en

N1 - FEMU ID: 19800; EMF-Portal URL: https://www.emf-portal.org/en/article/19800

SP - 59-64

TI - Mutagenic and morphologic impacts of 1.8 GHz radiofrequency radiation on human peripheral blood lymphocytes (hPBLs) and possible protective role of pre-treatment with Ginkgo biloba (EGb 761)

ER -

TY - JOUR

IS - 5

JA - Int J Radiat Biol

JO - International Journal of Radiation Biology

PY - 2006

SN - 0955-3002

VL - 82

AU - Stronati L

AU - Testa A

AU - Moquet J

AU - Edwards A

AU - Cordelli E

AU - Villani P

AU - Marino C

AU - Fresegna AM

AU - Appolloni M

AU - Lloyd D

DO - 10.1080/09553000600739173

LA - en

N1 - FEMU ID: 13927; EMF-Portal URL: https://www.emf-portal.org/en/article/13927

SP - 339-346

TI - 935 MHz cellular phone radiation. An in vitro study of genotoxicity in human lymphocytes

ER -

TY - JOUR

IS - 2

JO - Mutagenesis

PY - 2006

SN - 0267-8357

VL - 21

AU - Maes A

AU - Van Gorp U

AU - Verschaeve L

DO - 10.1093/mutage/gel008

LA - en

N1 - FEMU ID: 13387; EMF-Portal URL: https://www.emf-portal.org/en/article/13387

SP - 139-142

TI - Cytogenetic investigation of subjects professionally exposed to radiofrequency radiation

UR - https://academic.oup.com/mutage/article-pdf/21/2/139/3906515/gel008.pdf

ER -

TY - JOUR

IS - 12

JA - FASEB J

JO - The FASEB Journal

PY - 2005

SN - 0892-6638

VL - 19

AU - Nikolova T

AU - Czyz J

AU - Rolletschek A

AU - Blyszczuk P

AU - Fuchs J

AU - Jovtchev G

AU - Schuderer J

AU - Kuster N

AU - Wobus AM

DO - 10.1096/fj.04-3549fje

LA - en

N1 - FEMU ID: 12365; EMF-Portal URL: https://www.emf-portal.org/en/article/12365

SP - 1686-1688

TI - Electromagnetic fields affect transcript levels of apoptosis-related genes in embryonic stem cell-derived neural progenitor cells

ER -

TY - JOUR

IS - 4

JO - Bioelectromagnetics

PY - 2005

SN - 0197-8462

VL - 26

AU - Zeni O

AU - Romano M

AU - Perrotta A

AU - Lioi MB

AU - Barbieri R

AU - d'Ambrosio G

AU - Massa R

AU - Scarfi MR

DO - 10.1002/bem.20078

LA - en

N1 - FEMU ID: 11836; EMF-Portal URL: https://www.emf-portal.org/en/article/11836

SP - 258-265

TI - Evaluation of genotoxic effects in human peripheral blood leukocytes following an acute in vitro exposure to 900 MHz radiofrequency fields

ER -

TY - JOUR

IS - 2-3

JA - Electromagn Biol Med

JO - Electromagnetic Biology and Medicine

PY - 2003

SN - 1536-8386

VL - 22

AU - Gadhia PK

AU - Shah T

AU - Mistry A

AU - Pithawala M

AU - Tamakuvala D

DO - 10.1081/JBC-120024624

LA - en

N1 - FEMU ID: 10674; EMF-Portal URL: https://www.emf-portal.org/en/article/10674

SP - 149-159

TI - A Preliminary Study to Assess Possible Chromosomal Damage Among Users of Digital Mobile Phones

ER -

TY - JOUR

IS - 2

JO - Bioelectromagnetics

PY - 2001

SN - 0197-8462

VL - 22

AU - Maes A

AU - Collier M

AU - Verschaeve L

LA - en

N1 - FEMU ID: 5625; EMF-Portal URL: https://www.emf-portal.org/en/article/5625

SP - 91-96

TI - Cytogenetic effects of 900 MHz (GSM) microwaves on human lymphocytes

ER -

TY - JOUR

IS - 5

JA - Folia Biol

JO - Folia Biologica

PY - 2000

SN - 0015-5500

VL - 46

AU - Maes A

AU - Collier M

AU - Verschaeve L

LA - en

N1 - FEMU ID: 4988; EMF-Portal URL: https://www.emf-portal.org/en/article/4988

SP - 175-180

TI - Cytogenetic investigations on microwaves emitted by a 455.7 MHz car phone

ER -

TY - JOUR

IS - 13

JO - Chemosphere

PY - 1999

SN - 0045-6535

VL - 39

AU - Garaj-Vrhovac V

DO - 10.1016/s0045-6535(99)00139-3

LA - en

N1 - FEMU ID: 5642; EMF-Portal URL: https://www.emf-portal.org/en/article/5642

SP - 2301-2312

TI - Micronucleus assay and lymphocyte mitotic activity in risk assessment of occupational exposure to microwave radiation

ER -

TY - JOUR

JO - Edition Wissenschaft

PY - 1998

VL - 14

AU - Antonopoulos A

AU - Obe G

AU - Brinkmann K

AU - Eisenbrandt H

AU - Grigat JP

AU - Elsner R

AU - Storbeck W

AU - Dehmel G

LA - de

N1 - FEMU ID: 9446; EMF-Portal URL: https://www.emf-portal.org/en/article/9446

SP - 3-13

TI - Der Einfluß von hochfrequenten elektromagnetischen Feldern auf den Zellzyklus und auf die Frequenz von Schwesterchromatidaustauschen: Analysen an menschlichen Lymphozyten in Kultur

UR - https://d-nb.info/974863599/34

ER -

TY - JOUR

IS - 2-3

JA - Mutat Res Genet Toxicol Environ Mutagen

JO - Mutation Research - Genetic Toxicology and Environmental Mutagenesis

PY - 1997

VL - 395

AU - Antonopoulos A

AU - Eisenbrandt H

AU - Obe G

DO - 10.1016/s1383-5718(97)00173-3

LA - en

N1 - FEMU ID: 2200; EMF-Portal URL: https://www.emf-portal.org/en/article/2200

SP - 209-214

TI - Effects of high-frequency electromagnetic fields on human lymphocytes in vitro

ER -

TY - JOUR

IS - 1-2

JA - Mutat Res Genet Toxicol Environ Mutagen

JO - Mutation Research - Genetic Toxicology and Environmental Mutagenesis

PY - 1997

VL - 393

AU - Maes A

AU - Collier M

AU - Van Gorp U

AU - Vandoninck S

AU - Verschaeve L

DO - 10.1016/s1383-5718(97)00100-9

LA - en

N1 - FEMU ID: 948; EMF-Portal URL: https://www.emf-portal.org/en/article/948

SP - 151-156

TI - Cytogenetic effects of 935.2-MHz (GSM) microwaves alone and in combination with mitomycin C

ER -

TY - JOUR

JO - Edition Wissenschaft

PY - 1996

VL - 4

AU - Eberle P

AU - Erdtmann-Vourliotis M

AU - Diener S

AU - Finke HG

AU - Löffelholz B

AU - Schnor A

AU - Schräder M

LA - de

N1 - FEMU ID: 9467; EMF-Portal URL: https://www.emf-portal.org/en/article/9467

SP - 2-15

TI - Zellproliferation, Schwesterchromatidaustausche, Chromosomenaberrationen, Mikrokerne und Mutationsrate des HGPRT-Locus

UR - https://d-nb.info/974863475/34

ER -

TY - JOUR

IS - 1

JA - Environ Mol Mutagen

JO - Environmental and Molecular Mutagenesis

PY - 1996

SN - 0893-6692

VL - 28

AU - Maes A

AU - Collier M

AU - Slaets D

AU - Verschaeve L

DO - 10.1002/(SICI)1098-2280(1996)28:1<26::AID-EM6>3.0.CO;2-C

LA - en

N1 - FEMU ID: 934; EMF-Portal URL: https://www.emf-portal.org/en/article/934

SP - 26-30

TI - 954 MHz microwaves enhance the mutagenic properties of mitomycin C

ER -

TY - JOUR

IS - 6

JA - Acta Radiol

JO - Acta Radiologica

PY - 1993

SN - 0284-1851

VL - 34

AU - Yamazaki E

AU - Matsubara S

AU - Yamada I

LA - en

N1 - FEMU ID: 22349; EMF-Portal URL: https://www.emf-portal.org/en/article/22349

SP - 607-611

TI - Effect of Gd-DTPA and/or magnetic field and radiofrequency exposure on sister chromatid exchange in human peripheral lymphocytes

ER -

TY - JOUR

IS - 6

JO - Bioelectromagnetics

PY - 1993

SN - 0197-8462

VL - 14

AU - Maes A

AU - Verschaeve L

AU - Arroyo A

AU - De Wagter C

AU - Vercruyssen L

DO - 10.1002/bem.2250140602

LA - en

N1 - FEMU ID: 889; EMF-Portal URL: https://www.emf-portal.org/en/article/889

SP - 495-501

TI - In vitro cytogenetic effects of 2450 MHz waves on human peripheral blood lymphocytes

ER -

TY - JOUR

IS - 4

JO - Mutation Research - Letters

PY - 1992

VL - 282

AU - Fucic A

AU - Garaj-Vrhovac V

AU - Skara M

AU - Dimitrovic B

DO - 10.1016/0165-7992(92)90133-3

LA - en

N1 - FEMU ID: 875; EMF-Portal URL: https://www.emf-portal.org/en/article/875

SP - 265-271

TI - X-rays, microwaves and vinyl chloride monomer: their clastogenic and aneugenic activity, using the micronucleus assay on human lymphocytes

ER -

TY - JOUR

IS - 5

JO - Bioelectromagnetics

PY - 1991

SN - 0197-8462

VL - 12

AU - Ciaravino V

AU - Meltz ML

AU - Erwin DN

DO - 10.1002/bem.2250120504

LA - en

N1 - FEMU ID: 1770; EMF-Portal URL: https://www.emf-portal.org/en/article/1770

SP - 289-298

TI - Absence of a synergistic effect between moderate-power radio-frequency electromagnetic radiation and adriamycin on cell-cycle progression and sister-chromatid exchange

ER -

TY - JOUR

IS - 2

JO - Bioelectromagnetics

PY - 1990

SN - 0197-8462

VL - 11

AU - Meltz ML

AU - Eagan P

AU - Erwin DN

DO - 10.1002/bem.2250110206

LA - en

N1 - FEMU ID: 888; EMF-Portal URL: https://www.emf-portal.org/en/article/888

SP - 149-157

TI - Proflavin and microwave radiation: absence of a mutagenic interaction

ER -

TY - JOUR

IS - 4

JA - Environ Mutagen

JO - Environmental Mutagenesis

PY - 1987

SN - 0192-2521

VL - 9

AU - Ciaravino V

AU - Meltz ML

AU - Erwin DN

DO - 10.1002/em.2860090405

LA - en

N1 - FEMU ID: 2060; EMF-Portal URL: https://www.emf-portal.org/en/article/2060

SP - 393-399

TI - Effects of radiofrequency radiation and simultaneous exposure with mitomycin C on the frequency of sister chromatid exchanges in Chinese hamster ovary cells

ER -

TY - JOUR

IS - 2

JO - Bioelectromagnetics

PY - 1986

SN - 0197-8462

VL - 7

AU - Lloyd DC

AU - Saunders RD

AU - Moquet JE

AU - Kowalczuk CI

DO - 10.1002/bem.2250070212

LA - en

N1 - FEMU ID: 893; EMF-Portal URL: https://www.emf-portal.org/en/article/893

SP - 235-237

TI - Absence of chromosomal damage in human lymphocytes exposed to microwave radiation with hyperthermia

ER -

TY - JOUR

IS - 1

JO - Radiology

PY - 1985

SN - 0033-8419

VL - 155

AU - Wolff S

AU - James TL

AU - Young GB

AU - Margulis AR

AU - Bodycote J

AU - Afzal V

DO - 10.1148/radiology.155.1.4038809

LA - en

N1 - FEMU ID: 22347; EMF-Portal URL: https://www.emf-portal.org/en/article/22347

SP - 163-165

TI - Magnetic resonance imaging: absence of in vitro cytogenetic damage

ER -

TY - JOUR

IS - 2

JA - Int J Radiat Biol Relat Stud Phys Chem Med

JO - International Journal of Radiation Biology and Related Studies in Physics, Chemistry and Medicine

PY - 1984

SN - 0020-7616

VL - 46

AU - Lloyd DC

AU - Saunders RD

AU - Finnon P

AU - Kowalczuk CI

DO - 10.1080/09553008414551211

LA - en

N1 - FEMU ID: 13126; EMF-Portal URL: https://www.emf-portal.org/en/article/13126

SP - 135-141

TI - No clastogenic effect from in vitro microwave irradiation of G0 human lymphocytes

ER -

TY - JOUR

IS - 3

JA - J Natl Cancer Inst

JO - Journal of the National Cancer Institute

PY - 1983

SN - 0027-8874

VL - 70

AU - Banerjee R

AU - Goldfeder A

AU - Mitra J

LA - en

N1 - FEMU ID: 9033; EMF-Portal URL: https://www.emf-portal.org/en/article/9033

SP - 517-521

TI - Sister chromatid exchanges and chromosome aberrations induced by radiosensitizing agents in bone marrow cells of treated tumor-bearing mice

ER -

TY - JOUR

IS - 2

JA - Radiat Res

JO - Radiation Research

PY - 1981

SN - 0033-7587

VL - 85

AU - McRee DI

AU - MacNichols G

LA - en

N1 - FEMU ID: 1399; EMF-Portal URL: https://www.emf-portal.org/en/article/1399

SP - 340-348

TI - Incidence of sister chromatid exchange in bone marrow cells of the mouse following microwave exposure

ER -
