# Supplementary material for: A scoping review and evidence map of radiofrequency field exposure and genotoxicity: assessing in vivo, in vitro, and epidemiological data
Source: Front Public Health. 2025 Jul 30;13:1613353. doi: 10.3389/fpubh.2025.1613353 (PMC12343714; doi:10.3389/fpubh.2025.1613353)
Supplement: Supplementary file 3 [file Data_Sheet_3.zip › Search data/Radiofrequency Search.docx]

Pubmed Search Keywords

Search 1 Pubmed 15/04/2023

| Search: **((((((radiofrequency) OR (radiowave)) OR (microwave)) OR ("millimeter wave")) OR (“RF-EMR”)) OR (“RF-EMF”)) OR (mmWave)**  "radiofrequencies"[All Fields] OR "radiofrequency"[All Fields] OR "radiofrequent"[All Fields] OR ("microwavable"[All Fields] OR "microwaveable"[All Fields] OR "microwaved"[All Fields] OR "microwaves"[MeSH Terms] OR "microwaves"[All Fields] OR "microwave"[All Fields] OR "microwaving"[All Fields]) OR (("millimeter"[All Fields] OR "millimeters"[All Fields] OR "millimetre"[All Fields] OR "millimetres"[All Fields] OR "millimetric"[All Fields]) AND "wave"[All Fields]) OR ("rf"[Journal] OR "rf"[All Fields]) OR "RF-EMR"[All Fields] OR "RF-EMF"[All Fields] OR ("resour fem res"[Journal] OR "rfr"[All Fields]) OR "MWR"[All Fields] OR "MMW"[All Fields] OR "mmWave"[All Fields]  **Translations**  **radiofrequency:** "radiofrequencies"[All Fields] OR "radiofrequency"[All Fields] OR "radiofrequent"[All Fields]  **microwave:** "microwavable"[All Fields] OR "microwave's"[All Fields] OR "microwaveable"[All Fields] OR "microwaved"[All Fields] OR "microwaves"[MeSH Terms] OR "microwaves"[All Fields] OR "microwave"[All Fields] OR "microwaving"[All Fields]  **millimeter:** "millimeter"[All Fields] OR "millimeters"[All Fields] OR "millimetre"[All Fields] OR "millimetres"[All Fields] OR "millimetric"[All Fields]  **RF:** "RF"[Journal:__jid100927983] OR "rf"[All Fields]  **RFR:** "Resour Fem Res"[Journal:__jid100969513] OR "rfr"[All Fields] |  |
| --- | --- |

99,420 Records Identified

Search 2 – Pubmed 15/04/2023

Search: **(((((((((((((((((((((((((((((((((((((((("DNA damage") OR ("DNA break")) OR ("DNA double strand break")) OR ("DNA single strand break")) OR ("DNA fragmentation")) OR ("DNA SSB")) OR ("DNA DSB")) OR (micronuclei)) OR ("micronuclei induction")) OR ("chromosome aberration")) OR ("nuclear bud")) OR ("sister chromatid exchange")) OR ("dicentric rings")) OR ("acentric ring")) OR ("chromatid break")) OR ("chromosome break")) OR "chromosome deletion")) OR ("chromosome inversion")) OR ("chromosome exchange")) OR (aneuploidy)) OR (polyploidy)) OR ("chromosome translocation")) OR ("chromosome insertion")) OR (Ring chromosome)) OR (“8-Oxo-dG”)) OR (“8-oxoG”)) OR (“8-oxo-G”)) OR (“8-Oxoguanine”)) OR (“8-OH-dG”)) OR ("DNA crosslinking")) OR ("DNA base damage")) OR ("DNA base oxidation")) OR ("oxidative base damage")) OR ("point mutation")) OR ("DNA adduct")) OR ("chromosome fragment")) OR ("broken egg")) OR ("DNA mutation")) OR (mutagen)) OR (genotoxic)**

440,532 Records Identified

Search 3 - #1 AND #2

679 Records Identified

Search 4 - #3 NOT “COOK”

645 Records Identified

Search 5 Pubmed (final)

(((("radiofrequencies"[All Fields] OR "radiofrequency"[All Fields] OR "radiofrequent"[All Fields] OR ("radio waves"[MeSH Terms] OR ("radio"[All Fields] AND "waves"[All Fields]) OR "radio waves"[All Fields] OR "radiowave"[All Fields] OR "radiowaves"[All Fields]) OR ("microwavable"[All Fields] OR "microwaveable"[All Fields] OR "microwaved"[All Fields] OR "microwaves"[MeSH Terms] OR "microwaves"[All Fields] OR "microwave"[All Fields] OR "microwaving"[All Fields]) OR "millimeter wave"[All Fields] OR "RF-EMR"[All Fields] OR "RF-EMF"[All Fields] OR "mmWave"[All Fields]) AND ("DNA damage"[All Fields] OR "DNA break"[All Fields] OR "DNA double strand break"[All Fields] OR "DNA single strand break"[All Fields] OR "DNA fragmentation"[All Fields] OR "DNA SSB"[All Fields] OR "DNA DSB"[All Fields] OR "micronuclei"[All Fields] OR "micronuclei induction"[All Fields] OR "chromosome aberration"[All Fields] OR "nuclear bud"[All Fields] OR "sister chromatid exchange"[All Fields] OR "dicentric rings"[All Fields] OR "acentric ring"[All Fields] OR "chromatid break"[All Fields] OR "chromosome break"[All Fields] OR "chromosome deletion"[All Fields] OR "chromosome inversion"[All Fields] OR "chromosome exchange"[All Fields] OR ("aneuploidy"[MeSH Terms] OR "aneuploidy"[All Fields] OR "aneuploidies"[All Fields]) OR ("polyploidy"[MeSH Terms] OR "polyploidy"[All Fields] OR "polyploidies"[All Fields]) OR "chromosome translocation"[All Fields] OR "chromosome insertion"[All Fields] OR ("ring chromosomes"[MeSH Terms] OR ("ring"[All Fields] AND "chromosomes"[All Fields]) OR "ring chromosomes"[All Fields] OR ("ring"[All Fields] AND "chromosome"[All Fields]) OR "ring chromosome"[All Fields]) OR ("8 hydroxy 2 deoxyguanosine"[MeSH Terms] OR "8 hydroxy 2 deoxyguanosine"[All Fields] OR "8 oxo dg"[All Fields]) OR "8-oxoG"[All Fields] OR "8-oxo-G"[All Fields] OR ("8 hydroxyguanine"[Supplementary Concept] OR "8 hydroxyguanine"[All Fields] OR "8 oxoguanine"[All Fields]) OR ("8 hydroxy 2 deoxyguanosine"[MeSH Terms] OR "8 hydroxy 2 deoxyguanosine"[All Fields] OR "8 oh dg"[All Fields]) OR "DNA crosslinking"[All Fields] OR "DNA base damage"[All Fields] OR "DNA base oxidation"[All Fields] OR "oxidative base damage"[All Fields] OR "point mutation"[All Fields] OR "DNA adduct"[All Fields] OR "chromosome fragment"[All Fields] OR "broken egg"[All Fields] OR "DNA mutation"[All Fields] OR ("mutagenes"[All Fields] OR "mutagenic"[All Fields] OR "mutagenicities"[All Fields] OR "mutagenicity"[All Fields] OR "mutagenity"[All Fields] OR "mutagenization"[All Fields] OR "mutagenize"[All Fields] OR "mutagenized"[All Fields] OR "mutagenizing"[All Fields] OR "mutagens"[Pharmacological Action] OR "mutagens"[MeSH Terms] OR "mutagens"[All Fields] OR "mutagen"[All Fields]) OR ("genotoxic"[All Fields] OR "genotoxical"[All Fields] OR "genotoxically"[All Fields] OR "genotoxicities"[All Fields] OR "genotoxicity"[All Fields] OR "genotoxics"[All Fields] OR "genotoxity"[All Fields]))) NOT ("ablate"[All Fields] OR "ablated"[All Fields] OR "ablates"[All Fields] OR "ablating"[All Fields] OR "ablation"[All Fields] OR "ablational"[All Fields] OR "ablations"[All Fields])) NOT ("diathermy"[MeSH Terms] OR "diathermy"[All Fields] OR "diathermies"[All Fields])) NOT ("review"[Publication Type] OR "review literature as topic"[MeSH Terms] OR "review"[All Fields])

**Translations**

**radiofrequency:** "radiofrequencies"[All Fields] OR "radiofrequency"[All Fields] OR "radiofrequent"[All Fields]

**radiowave:** "radio waves"[MeSH Terms] OR ("radio"[All Fields] AND "waves"[All Fields]) OR "radio waves"[All Fields] OR "radiowave"[All Fields] OR "radiowaves"[All Fields]

**microwave:** "microwavable"[All Fields] OR "microwave's"[All Fields] OR "microwaveable"[All Fields] OR "microwaved"[All Fields] OR "microwaves"[MeSH Terms] OR "microwaves"[All Fields] OR "microwave"[All Fields] OR "microwaving"[All Fields]

**aneuploidy:** "aneuploidy"[MeSH Terms] OR "aneuploidy"[All Fields] OR "aneuploidies"[All Fields]

**polyploidy:** "polyploidy"[MeSH Terms] OR "polyploidy"[All Fields] OR "polyploidies"[All Fields]

**Ring chromosome:** "ring chromosomes"[MeSH Terms] OR ("ring"[All Fields] AND "chromosomes"[All Fields]) OR "ring chromosomes"[All Fields] OR ("ring"[All Fields] AND "chromosome"[All Fields]) OR "ring chromosome"[All Fields]

**8-Oxo-dG:** "8-hydroxy-2'-deoxyguanosine"[MeSH Terms] OR "8-hydroxy-2'-deoxyguanosine"[All Fields] OR "8 oxo dg"[All Fields]

**8-Oxoguanine:** "8-hydroxyguanine"[Supplementary Concept] OR "8-hydroxyguanine"[All Fields] OR "8 oxoguanine"[All Fields]

**8-OH-dG:** "8-hydroxy-2'-deoxyguanosine"[MeSH Terms] OR "8-hydroxy-2'-deoxyguanosine"[All Fields] OR "8 oh dg"[All Fields]

**mutagen:** "mutagenes"[All Fields] OR "mutagenic"[All Fields] OR "mutagenicities"[All Fields] OR "mutagenicity"[All Fields] OR "mutagenity"[All Fields] OR "mutagenization"[All Fields] OR "mutagenize"[All Fields] OR "mutagenized"[All Fields] OR "mutagenizing"[All Fields] OR "mutagens"[Pharmacological Action] OR "mutagens"[MeSH Terms] OR "mutagens"[All Fields] OR "mutagen"[All Fields]

**genotoxic:** "genotoxic"[All Fields] OR "genotoxical"[All Fields] OR "genotoxically"[All Fields] OR "genotoxicities"[All Fields] OR "genotoxicity"[All Fields] OR "genotoxics"[All Fields] OR "genotoxity"[All Fields]

**ablation:** "ablate"[All Fields] OR "ablated"[All Fields] OR "ablates"[All Fields] OR "ablating"[All Fields] OR "ablation"[All Fields] OR "ablational"[All Fields] OR "ablations"[All Fields]

**diathermy:** "diathermy"[MeSH Terms] OR "diathermy"[All Fields] OR "diathermies"[All Fields]

**review:** "review"[Publication Type] OR "review literature as topic"[MeSH Terms] OR "review"[All Fields]

567 Records Identified

SCOPUS Search Keywords

Search 1 keywords

## {radiofrequency} OR "radiowave" OR {microwave} OR "millimeter wave" OR "RFR" OR "RF-EMR" OR "RF-EMF" OR "mmWave"

583,509 Records Identified

Search 2 keywords

## "DNA damage" OR "DNA break" OR "DNA double strand break" OR "DNA single strand break" OR "DNA fragmentation" OR "micronuclei" OR {micronuclei induction} OR "chromosome aberration" OR {nuclear bud} OR {sister chromatid exchange} OR {dicentric ring} OR {acentric ring} OR {chromatid break} OR {chromosome break} OR "chromosome deletion" OR "chromosome inversion" OR "chromosome exchange" OR "aneuploidy" OR "polyploidy" OR "chromosome translocation" OR "chromosome insertion" OR "ring chromosome" OR {8-Oxo-dG} OR {8-oxoG} OR {8-oxo-G} OR {8-Oxoguanine} OR {8-OH-dG} OR {DNA crosslinking} OR "DNA base damage" OR "DNA base oxidation" OR "oxidative base damage" OR "point mutation" OR "DNA adduct" OR "chromosome fragment" OR {broken egg} OR "DNA mutation" OR "mutagen" OR "genotoxic"

557,282 Records Identified

Search 3 - #1 AND #2

1,098 Records Identified

Search 4 - #3 AND NOT

"ablation" OR "diathermy" OR "review" OR "integrated circuit" OR "extract*" OR "cook" OR "cooking" OR "synthesis"

645 Records Identified

IEEE Search Keywords

Search #1 keywords

## “radiofrequency” OR "radiowave" OR “microwave” OR "millimeter wave" OR "RFR" OR "RF-EMR" OR "RF-EMF" OR "mmWave"

380,825 Records Identified

Search #2 keywords

"DNA damage" OR "DNA break" OR "DNA double strand break" OR "DNA single strand break" OR "DNA fragmentation" OR "micronuclei" OR "chromosome aberration" OR "nuclear bud" OR "sister chromatid exchange" OR "dicentric ring" OR "acentric ring" OR "chromatid break" OR "chromosome break" OR "ploidy" OR "DNA base oxidation" OR "oxidative base damage" OR "chromosome fragment" OR "broken egg" OR "mutation" OR "mutagen" OR "genotoxic"

10,847 Records Identified

Search 3 - #1 AND #2

121 Records Identified

Science Direct (keyword limits apply) Search Keywords

Search 1 keywords

("radiofrequency" OR "radiowave" OR "microwave" OR "millimeter wave")

476,579 Records Identified

Search 2 keywords

"DNA damage" OR "DNA fragmentation" or "DNA breaks" or "DNA base damage" OR "micronucle" OR "chromosome aberration" OR "DNA oxidation"

58,364 Records Identified

Search 3 keywords

("radiofrequency" OR "radiowave" OR "microwave" OR "millimeter wave") AND ("DNA damage" OR "DNA fragmentation" or "DNA breaks" or "DNA base damage" OR "micronucle" OR "chromosome aberration" OR "DNA oxidation")

628 Records Identified

Search 4 - #3

Include Articles Only [tick box checked]

378 Records Identified

Web of Science Search Keywords

Search 1 keywords

"DNA damage" OR "DNA break" OR "DNA double strand break" OR "DNA single strand break" OR "DNA fragmentation" OR "DNA SSB" OR "DNA DSB" OR micronuclei OR "chromosome aberration" OR "nuclear bud" OR "sister chromatid exchange" OR "dicentric rings" OR "acentric ring" OR "chromatid break" OR "chromosome break" OR "chromosome deletion" OR "chromosome inversion" OR "chromosome exchange" OR aneuploidy OR polyploidy OR "chromosome translocation" OR "chromosome insertion" OR Ring chromosome OR “8-Oxo-dG” OR “8-oxoG” OR “8-oxo-G” OR “8-Oxoguanine” OR “8-OH-dG” OR "DNA crosslinking" OR "DNA base damage" OR "DNA base oxidation" OR "oxidative base damage" OR "point mutation" OR "DNA adduct" OR "chromosome fragment" OR "broken egg" OR "DNA mutation" OR mutagen OR genotoxic

298,298 Records Identified

Search 2 keywords

radiofrequency OR radiowave OR microwave OR "millimeter wave" OR “RF-EMR” OR “RF-EMF” OR mmWave

594,596 Records Identified

Search 3 - #1 AND #2

830 Records Identified

Search 4 - #3 AND

NOT cooking or ablation or diathermy or “integrated circuit" or extraction or synthesis or cook or extract

612 Records Identified

Search 5 - #4 AND

Not book chapters

603 Records Identified

EMF Portal – has key word limits. It was necessary to create separate search criteria for each type of DNA damage. The output of each search result was saved to a uniquely named text file. Manually copied all text records from each file to a common MS Excel spreadsheet. Used Excel sort feature on merged result to identify and remove duplicates.

476 unique papers identified as potential candidates. The individual search keywords applied for each type of DNA damage are detailed below.

EMF-Portal Search Keywords

Search 1 keywords

8-Oxo-dG, 8-oxoG, 8-Hydroxy-2-Desoxyguanosin, 8-OHdG, 8-hydroxy-2-deoxyguanosine, "DNA base damage", "DNA base oxidisation"

30 Records Identified

Search 2 keywords

aneuploidy, Aneuploidie, 異数体, polyploid, 倍数体

15 Records Identified

Search 3 keywords

"DNA damage", "chromosome aberration", Chromosomenaberration, Chromosomenmutation, Aberration, 染色体異常, Mikronukleus, Mikrokern, MN, micronucleus, 小核, "sister chromatid exchange", Schwesterchromatid-Austausch, SCE, 姉妹染色分体交換, "DNA breaks", "double strand break", "single strand break", "DNA fragmentation", "DNA base damage"

416 Records Identified

Search 4 keywords

Genotoxizität, genotoxicity, 遺伝毒性

359 Records Identified

Search 5 keywords

Mikronukleus, Mikrokern, MN, micronucleus, 小核, "micronuclei induction", "nuclear bud", "broken egg"

137 Records Identified

Search 6 keywords

“DNA Damage”, "DNA breaks", "double strand break", "single strand break", "DNA fragmentation", "DNA base damage"

281 Records Identified

Search 7 keywords

“Sister Chromatid Exchange”

26 Records Identified

Search 8 keywords

"chromosome aberration", Chromosomenaberration, Chromosomenmutation, Aberration, 染色体異常, "chromosome exchange", "chromosome inversion", Chromosomeninversion, Inversion, 染色体逆位, 逆位, "chromosome deletion", Chromosomendeletion, 染色体欠失, "chromosome break", Chromosomenbruch, 染色体切断, "chromatid break", "dicentric chromosome", "dizentrisches Chromosom", 二動原体染色体, "acentric chromosome", "ring chromosome", Ringchromosom, 環状染色体

93 Records Identified

Search 9 keywords

Mutation, 突然変異, Mutagen, 突然変異原

310 Records Identified
