# Supplementary material for: A scoping review and evidence map of radiofrequency field exposure and genotoxicity: assessing in vivo, in vitro, and epidemiological data
Source: Front Public Health. 2025 Jul 30;13:1613353. doi: 10.3389/fpubh.2025.1613353 (PMC12343714; doi:10.3389/fpubh.2025.1613353)
Supplement: Supplementary file 5 [file Data_Sheet_5.pdf]

Notes

Trends: A trend was identified when the study found 10% or more DNA damage for the RF-EMF exposed compared with the sham or control sample. Trends occurred in either positive or negative directions. Results were classified as a “mixed trend” when a study involved multiple experiments with both positive and negative trends. All trends were classified as non-significant effects. Additionally, studies showing trends or protective effects were all grouped under the “no effect” category i.e., no significant DNA damage, when calculating balance of evidence data.

Supp_Table 1: Types of DNA damage and assays to detect them

DNA Damage	Assays
**DNA Breaks**<br>- **Double Strand**	• Comet Assay – Single Cell Gel Electrophoresis (SCGE)<br>• DNA Ladder – Agarose Gel Electrophoresis (AGE)<br>• Terminal deoxynucleotidyl transferase dUTP Nick-End Labelling (TUNEL) Assay<br>• Anomalous Viscosity Time Dependence (AVTD)<br>• Fluorescence Microscopy–γ-H2AX<br>• Change in p53 expression detected by immunohistochemistry
**DNA Breaks**<br>- **Single Strand**	• Comet Assay<br>• Terminal deoxynucleotidyl transferase dUTP Nick-End Labelling (TUNEL) Assay<br>• Anomalous Viscosity Time Dependence (AVTD)
**DNA Base Damage**	• Formamidopyrimidine DNA Glycosylase (Fpg) modified Comet Assay<br>• 8-hydroxy-2’-deoxyguanosine (8-OHdG) Assay<br>• 8-oxoguanine (8-oxoG) Antibody Assay<br>• HPLC-MS (High-Performance Liquid Chromatography-Mass Spectrometry)<br>• Reverse Phase High-Performance Liquid Chromatography (HPLC-EC)<br>• ELISA (Enzyme-Linked Immunosorbent Assay)<br>• Polymerase Chain Reaction (PCR) for point mutations<br>• Random Amplified Polymorphic DNA (RAPD) Methodology
**DNA Conformational Change**	• Anomalous Viscosity Time Dependence (AVTD)<br>• Dynamic Light Scattering (DLS)<br>• Nuclear Magnetic Resonance (NMR) Spectroscopy<br>• Raman spectroscopy<br>• UV–vis spectroscopy<br>• Electron Microscopy Observations
**Chromosome Aberrations (Structural)**	• Squash method combined with Feulgen techniques Chromosome Aberrations Assay – Giemsa Stain<br>• Chromosome Aberrations Assay – Acetic Orcein Stain<br>• Chromosomal Aberrations Assay – Acetocarmine Stain<br>• Chromosome Aberrations Assay – Toluidine Blue Stain<br>• Hematoxylin and Eosin Stain<br>• Hoechst 33342 (H342) Stain<br>• Confocal Laser Scanning Microscopy with DAPI stain<br>• R-banded Karyotyping<br>• Sperm Chromatin Structure Assay (SCSA)
**Chromosome Aberrations (Numerical)**	• Fluorescence in situ hybridisation (FISH)<br>• DNA ploidy analysis<br>• Flow Cytometry/ Fluorescence-Activated Cell Sorting (FACS)

**Micronuclei Induction**	• Micronucleus Test/Cytokinesis Block Micronucleus (CBMN) Test – Giemsa Stain<br>• Micronucleus Test - Propidium Iodide Stain<br>• Micronucleus Test - Feulgen Reaction Stain<br>• Micronucleus Test - Acridine Orange Stain<br>• Micronucleus Test - DAPI (4,6-diamidino -2-phenylindole dihydrochloride) Stain<br>• Flow Cytometric Micronuclei Scoring<br>• Micronucleus Assay with anti-Kinetochore Antibody (CREST)<br>• Microscope - Hematoxylin and Eosin stain<br>• Squash method combined with Feulgen techniques
**Sister Chromatid Exchange**	• Sister Chromatid Exchange (SCE) Test<br>• Fluorescence plus Giemsa (FPG) staining<br>• Cytogenic Analysis - Feulgen stain
**Nuclear Bud/Broken Eggs**	• Cytokinesis Block Micronucleus Test<br>• Acetic Orcein Stain
**Spindle Disturbances**	• Spindle Disturbance Assay - 2% Acetic Orcein
**Multiple Mutations**	• Dominant Lethal Assay<br>• Polymerase Chain Reaction (PCR)<br>• Randomly Amplified Polymorphic DNA (RAPD)<br>• Tetrazolium Overlay Technique<br>• Petite Mutations<br>• AMES Test<br>• Mutant Frequencies<br>• Pig-Alpha mutation test<br>• Wing Spot Test<br>• Thymidine Kinase Locus Assay<br>• Somatic Mutation and Recombination Test (SMART) Assay

Examples of Comet Assay study parameter differences include:

- Duration times for lysis and temperatures at which it is conducted (ice-cold, room temperature, 37°c);
- pH (8 to 10, 13 and >13), which is important for detecting different types of DNA damage (DS vs SS DNA breaks);
- buffer formulations;
- electrophoresis voltages;
- electrophoresis run times;
- temperatures used for electrophoresis;
- number of neutralisation steps;
- staining methods;
- methods of analysing comets and recording results.

All of these parameters can influence the sensitivity of the comet assay to detect DNA breaks.

1. Research overview

Location details

Research has been conducted all over the world with USA, China, India, Italy, Japan and Turkey being the dominant countries in this research space. See next page for map.

Supp_Table 2: Number of articles by location and findings – heat map applied based on % of "Significant Effects"

Country	Significant Effect	%	No Significant Effect	%	Total Papers
Argentina	1	100.0	0	0.0	1
Australia	4	44.4	5	55.6	9
Austria	4	80.0	1	20.0	5
Belgium	4	50.0	4	50.0	8
Brazil	5	55.6	4	44.4	9
Canada	1	16.7	5	83.3	6
China	28	60.9	18	39.1	46
Croatia	22	91.7	2	8.3	24
Czechoslovakia	0	0.0	1	100.0	1
Egypt	12	92.3	1	7.7	13
Finland	0	0.0	5	100.0	5
France	2	13.3	13	86.7	15
Germany	6	30	14	70	20
Great Britain	1	14.3	6	85.7	7
Greece	12	100.0	0	0.0	12
Hungary	4	80.0	1	20.0	5
India	50	94.3	3	5.7	53
Iran	11	73	4	27	15
Iraq	1	50.0	1	50.0	2
Israel	4	66.7	2	33.3	6
Italy	20	42.6	27	57.4	47
Japan	12	41	17	59	36
Jordan	1	100.0	0	0.0	1
Latvia	1	100.0	0	0.0	1
Malaysia	4	100.0	0	0.0	4
Netherlands	1	100.0	0	0.0	1
Nigeria	4	100.0	0	0.0	4
Norway	1	50.0	1	50.0	2
Pakistan	1	100.0	0	0.0	1
Poland	3	75.0	1	25.0	4
Portugal	0	0.0	1	100.0	1
Romania	4	100.0	0	0.0	4
Russia	3	42.9	4	57.1	7
Saudi Arabia	3	50.0	3	50.0	6
Serbia	2	100.0	0	0.0	2
Slovakia	1	25.0	3	75.0	4
South Africa	1	50.0	1	50.0	2
South Korea	4	26.7	11	73.3	15
Spain	0	0.0	1	100.0	1
Sweden	4	50.0	4	50.0	8
Switzerland	1	50.0	1	50.0	2
Taiwan	1	100.0	0	0.0	1
Turkey	27	77	8	23	35
Ukraine	6	100.0	0	0.0	6
USA	28	45.2	34	54.8	62

Publication History

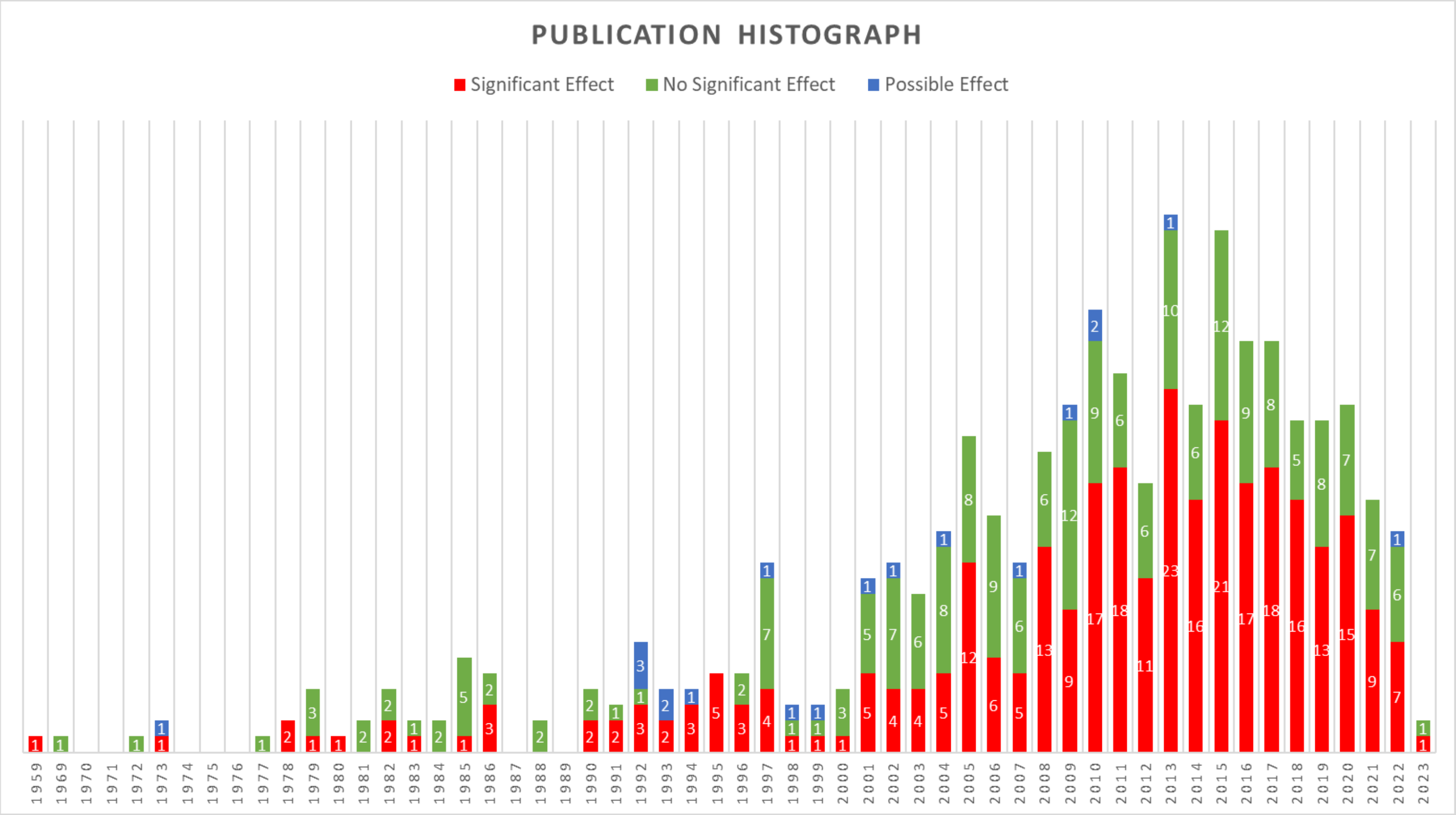

Supp_Figure 1: Publication Histogram

2. Organism and cell types

Mammals

All papers

Supp_Table 3: RF-EMF DNA damage studies on organisms – heat map colour coding applied to "# Papers" as well as separately for "% Significant damage" for each experiment type

Organism	Human Studies			Rat Studies		Mice Studies		Other Mammal Studies			All Mammalian Studies		
Finding / Study Type	In Vitro	In Vivo	Epidemiological	In Vitro	In Vivo	In Vitro	In Vivo	In Vitro	In Vivo	Epidemiological	In Vitro	In Vivo	Epidemiological
Significant DNA damage	67	5	39	12	65	12	24	19	10	1	110	104	40
No-Significant damage	102	1	12	9	15	20	21	8	3	0	139	40	12
Protective Effect	1	0	0	0	1	1	0	0	0	0	2	1	0
Total # Papers	170	6	51	21	81	33	45	27	13	1	251	145	52
% Significant damage	39	83	76	57	80	36	53	70	77	100	44	72	77
% No-Significant damage	60	17	24	43	19	61	47	30	23	0	55	27	23
% Protective Effect	1	0	0	0	1	3	0	0	0	0	1	1	0
Balance of Evidence (All Studies)	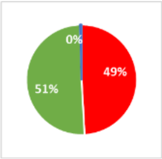			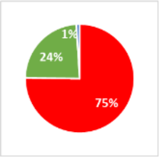		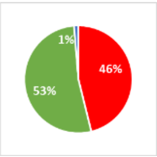		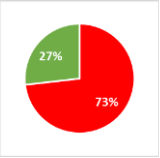			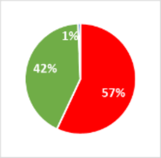		

Other organisms

Supp_Table 4: RF-EMF DNA damage studies on organisms – heat map colour coding applied to “# Papers” as well as separately for “% Significant damage” for each experiment type

Organism	Microbes, Plasmids		Plants		Snails		Worms		Insects		Birds		Amphibians	
Finding / Study Type	In Vitro	In Vivo	In Vitro	In Vivo	In Vitro	In Vivo	In Vitro	In Vivo	In Vitro	In Vivo	In Vitro	In Vivo	In Vitro	In Vivo
Significant DNA damage	16		1	18		0		3		15		6	2	
No-Significant damage	17		0	0		1		0		6		0	0	
Total # Papers	33		1	18		1		3		21		6	2	
% Significant damage	48		100	100		0		100		71		100	100	
% No-Significant damage	52		0	0		100		0		29		0	0	
Balance of Evidence (All Studies)	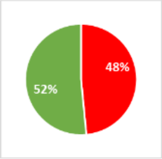		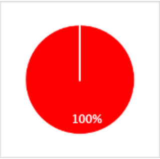		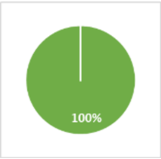		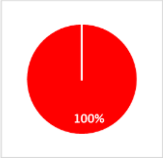		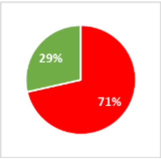		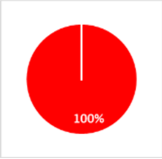		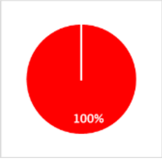	

Cells

Supp_Table 5: RF-EMF DNA damage studies on cells – heat map colour coding applied to "# Papers" as well as separately for "% Significant damage" for each experiment type

Cell Type	Lymphocytes			Leukocytes			Erythrocytes (Blood)			Erythrocytes (Bone)		Bone Marrow		Brain Neurons (Neoplasms)		Brain Neurons		Buccal Mucosa			Embryonic	
Finding / Study Type	In Vitro	In Vivo	Epidemiological	In Vitro	In Vivo	Epidemiological	In Vitro	In Vivo	Epidemiological	In Vitro	In Vivo	In Vitro	In Vivo	In Vitro	In Vivo	In Vitro	In Vivo	In Vitro	In Vivo	Epidemiological	In Vitro	In Vivo
Significant DNA damage	25	3	17	1	2	3	2	11	1		8	0	3	4		6	35	0		15	4	7
No-Significant damage	43	4	3	12	2	0	0	7	0		7	4	3	8		3	10	1		7	8	1
Total # Papers	68	7	20	13	4	3	2	18	1		15	4	6	12		9	45	1		22	12	8
% Significant damage	37	43	85	8	50	100	100	61	100		53	0	50	33		67	78	0		68	33	88
% No-Significant damage	63	57	15	92	50	0	0	39	0		47	100	50	67		33	22	100		32	67	12
Balance of Evidence (All Studies)	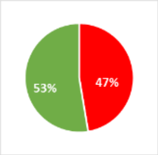			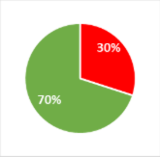			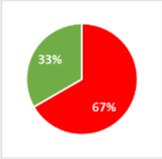			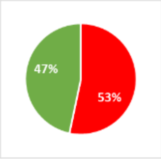		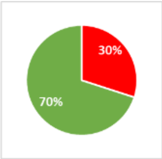		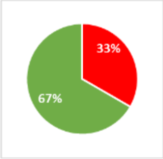		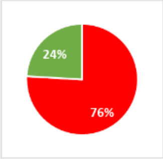		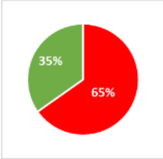			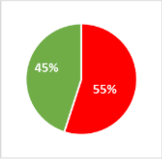	

Supp_Table 6: RF-EMF DNA damage studies on cells – heat map colour coding applied to "# Papers" as well as separately for "% Significant damage" for each experiment type

Cell Type	Eye (Lens/Cornea)		Liver		Lung		Ovary		Skin		Spermatozoa			Testicle		Larvae	
Finding / Study Type	In Vitro	In Vivo	In Vitro	In Vivo	In Vitro	In Vivo	In Vitro	In Vivo	In Vitro	In Vivo	In Vitro	In Vivo	Epidemiological	In Vitro	In Vivo	In Vitro	In Vivo
Significant DNA damage	2	2	2	7	7	2	5	11	5	0	9	7	4	4	20		3
No-Significant damage	8	0	2	5	7	0	4	0	7	3	4	1	2	2	4		1
Total # Papers	10	2	4	12	14	2	9	11	12	3	13	8	6	6	24		4
% Significant damage	20	100	50	58	50	100	56	100	50	0	69	78	67	67	83		75
% No-Significant damage	80	0	50	42	50	0	44	0	50	100	31	22	33	33	17		25
Balance of Evidence (All Studies)	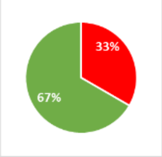		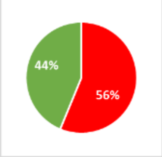		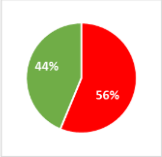		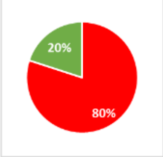		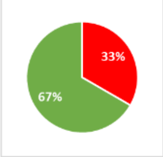		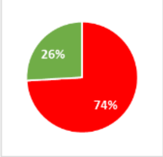			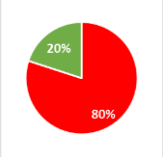		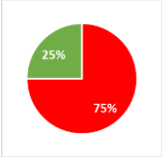	

3. Study type and type of damage type

All papers

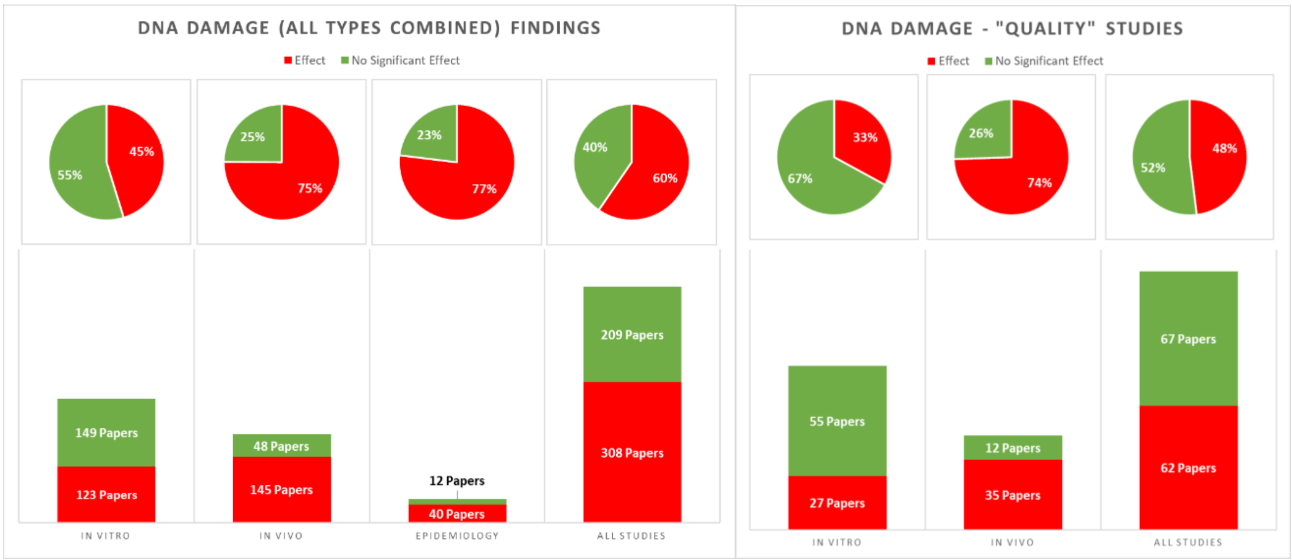

Supp_Figure 2: (A) Balance of evidence summary for all studies (B) Balance of evidence for quality studies

DNA damage by type (All Studies)

Supp_Table 7: Balance of evidence for all types of DNA damage (all studies) – heat map colour coding applied to "# Papers" as well as separately for "% Significant damage" for each experiment type

Genotoxic Endpoint	DNA Breaks/ Fragmentation			DNA Base Damage			Chromosome Aberrations			Micronuclei			Sister Chromatid Exchange			Mutations		
Finding / Study Type	In Vitro	In Vivo	Epidemiological	In Vitro	In Vivo	Epidemiological	In Vitro	In Vivo	Epidemiological	In Vitro	In Vivo	Epidemiological	In Vitro	In Vivo	Epidemiological	In Vitro	In Vivo	Epidemiological
Significant DNA damage	65	84	17	12	20	4	29	25	10	24	30	21	2	0	1	8	10	
No-Significant damage	98	16	3	1	5	0	25	9	3	42	14	9	17	3	2	12	12	
Total # papers	163	100	20	13	25	4	54	34	13	66	44	30	19	3	3	20	22	
% Significant damage	40	84	85	92	75	100	54	74	77	36	68	70	11	0	33	40	45	
% No-Significant damage	60	16	15	8	25	0	46	26	23	64	32	30	89	100	67	60	55	
Balance of Evidence (All Studies)	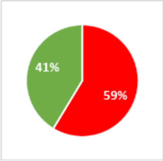			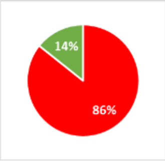			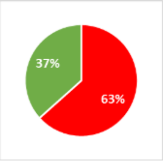			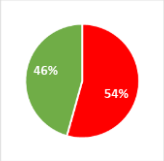			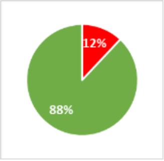			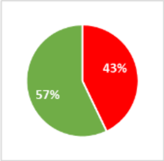		

DNA damage by type ("Quality" Studies)

Supp_Table 8: Balance of evidence for all types of DNA damage (quality studies) – heat map colour coding applied to "# Papers" as well as separately for "% Significant damage" for each experiment type

Genotoxic Endpoint	DNA Damage/ Fragmentation		DNA Base Damage		Chromosome Aberrations		Micronuclei Induction		Sister Chromatid Exchange		Mutation	
Finding / Study Type	In Vitro	In Vivo	In Vitro	In Vivo	In Vitro	In Vivo	In Vitro	In Vivo	In Vitro	In Vivo	In Vitro	In Vivo
Significant DNA damage	14	23	5	4	5	3	3	8	1		1	0
No-Significant damage	41	4	1	2	9	1	19	5	4		1	2
Total # papers	55	27	6	6	14	4	22	13	5		2	2
% Significant damage	25	85	83	67	36	75	14	62	20		50	0
% No-Significant damage	75	15	27	33	64	25	86	38	40		50	100
Balance of Evidence (All Studies)	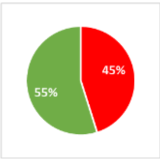		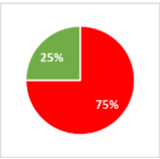		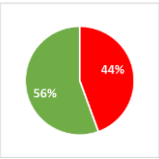		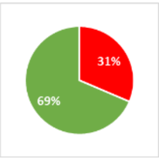		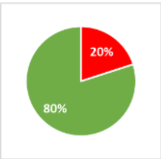		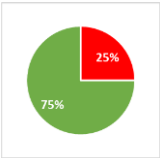	

Signs of Potential DNA damage (All Papers)

Supp_Table 9: Potential DNA damage map (all studies)

Potential Genotoxic Endpoint	DNA Conformational Change			Apoptosis		
Finding / Study Type	In Vitro	In Vivo	Epidemiological	In Vitro	In Vivo	Epidemiological
Significant effects	33	8	2	25	41	1
No-Significant effects	2	1	0	33	9	3
Total # papers	35	9	2	58	50	4
% Significant effects	94	89	100	43	82	25
% No-Significant effects	6	11	0	57	18	75
Balance of Evidence (All Studies)	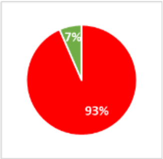			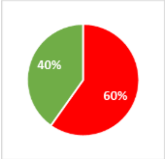		

Potential DNA damage (“Quality” Studies)

Supp_Table 10: Potential DNA damage map (quality studies)

Potential Genotoxic Endpoint	DNA Conformational Change		Apoptosis	
Finding / Study Type	In Vitro	In Vivo	In Vitro	In Vivo
Significant effects	7	1	5	8
No-Significant effects	0	0	9	4
Total # papers	7	1	14	12
% Significant effects	100	100	36	67
% No-Significant effects	0	0	64	33
Balance of Evidence (All Studies)	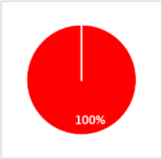		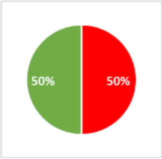	

Synergistic effects

Supp_Table 11: Synergistic, combinative and protective effects with other genotoxic agents

**Adaptive/Protective Response**	DNA Breaks/Fragmentation (# Studies)	DNA Conformation (# Studies)	Chromosome Breaks (# Studies)	Micronuclei (# Studies)
Gamma Rays	3			1
X-Rays	2		1	1
Ultraviolet Radiation (UV)	1			
Menadione (MD)	4			
Mitomycin C (MMC)			1	5
Bleomycin (BLM)	2			
**Synergistic Effect**	DNA Breaks/ Fragmentation (# Studies)	DNA Conformation (# Studies)	Chromosome Breaks (# Studies)	Micronuclei (# Studies)
Gamma Rays	1		2	
X-Rays			1	1
Ultraviolet Radiation (UV)	1			
Menadione (MD)	2			
Mitomycin C (MMC)	2			
4-Nitroquinoline 1-oxide (4NQO)	2			
Methyl methane sulfonate (MMS)				1
Cyclophosphamide (CPA)	1			
Copper Oxide	1			
3-chloro-4-(dichloromethyl)-5-hydroxy-2(5h)-furanone (MX)	1			
Hydrogen Peroxide		1		

4. Exposure Signal Characteristics

Frequency

Frequency Map

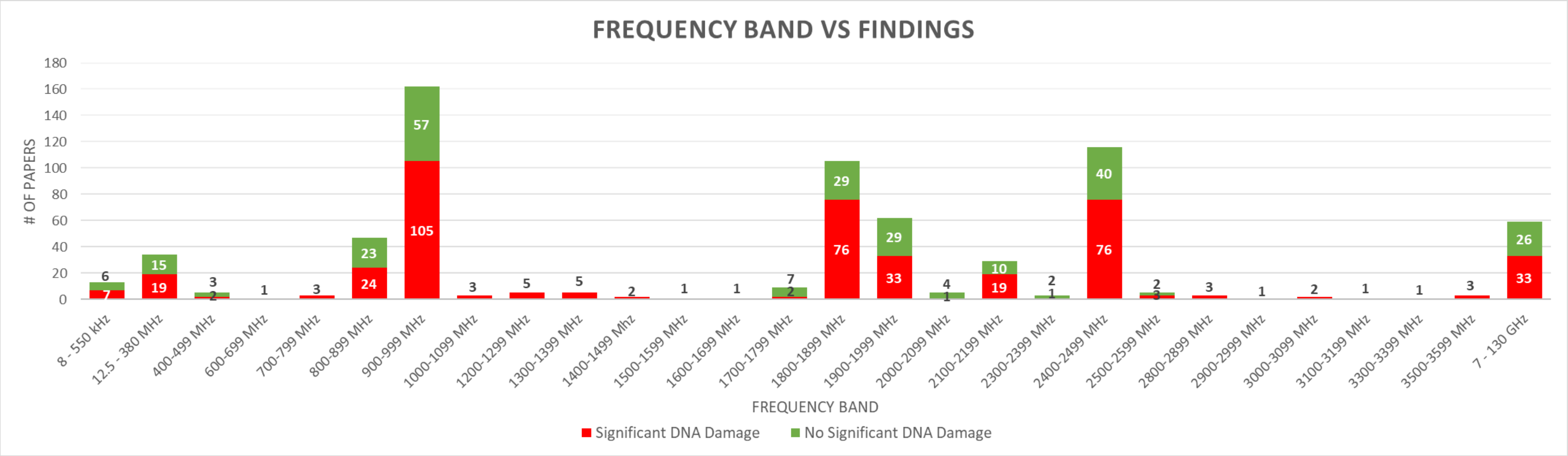

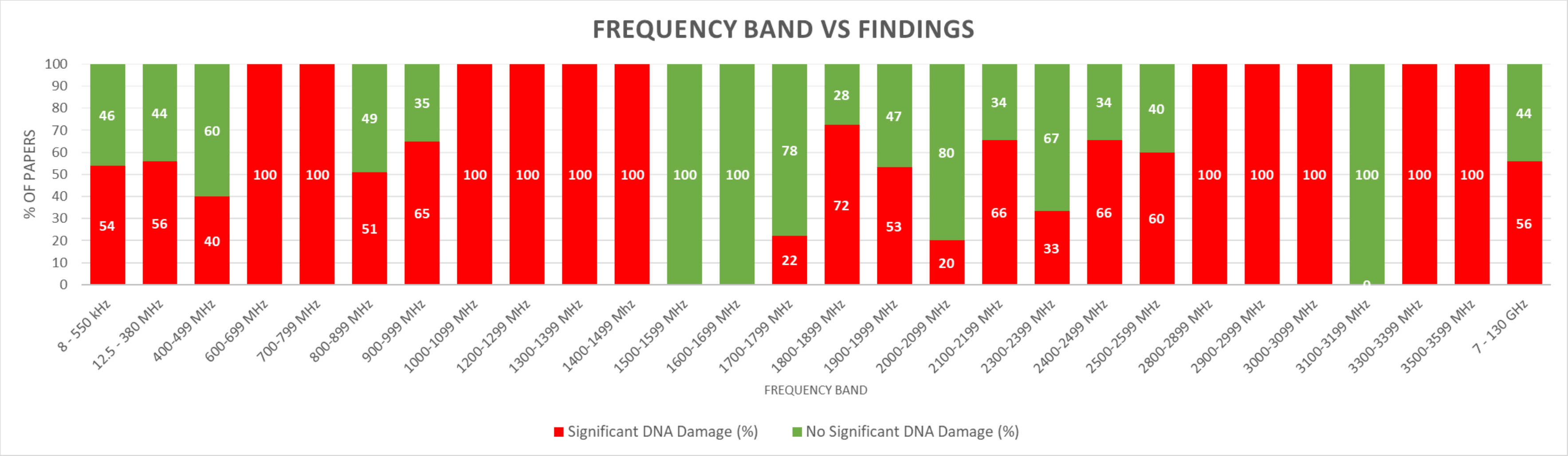

Supp_Figure 3: DNA damage vs Frequency band map

Duration

Exposure Time Map (All Studies)

Supp_Table 12: Exposure time map (all studies) – heat map colour coding applied to “# Papers” as well as separately for “% Significant damage” for each experiment type

Exposure Duration	Acute (<1 hr)			Short (1 - 24 hrs)			Medium (>24hrs - 6 months)			Long (> 6 months)		
Finding / Study Type	In Vitro	In Vivo	Epidemiological	In Vitro	In Vivo	Epidemiological	In Vitro	In Vivo	Epidemiological	In Vitro	In Vivo	Epidemiological
Significant DNA damage	72	33		62	43		11	63	0	1	13	39
No-Significant damage	51	11		111	27		12	21	1	0	5	12
Total # papers	123	44		173	70		23	84	1	1	18	51
% Significant damage	59	75		36	61		48	75	0	100	72	76
% No-Significant damage	41	25		64	39		52	25	100	0	28	24
Balance of Evidence (All Studies)	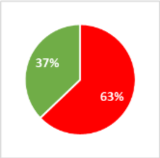			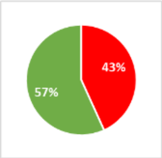			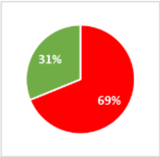			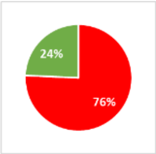		

Duration by damage type

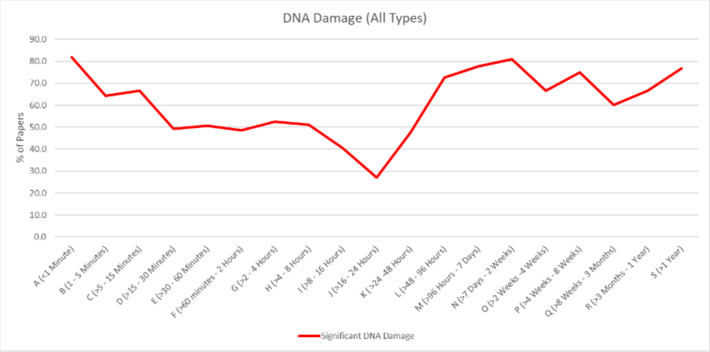

Supp_Figure 4A: DNA damage (All types) balance of evidence

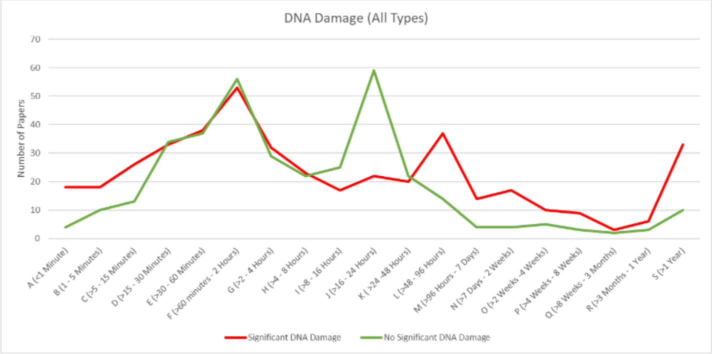

Supp_Figure 4B: DNA damage (All types) - number of papers

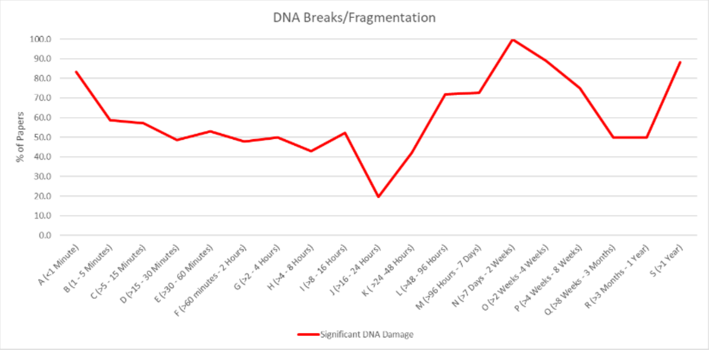

Supp_Figure 5A: DNA breaks balance of evidence

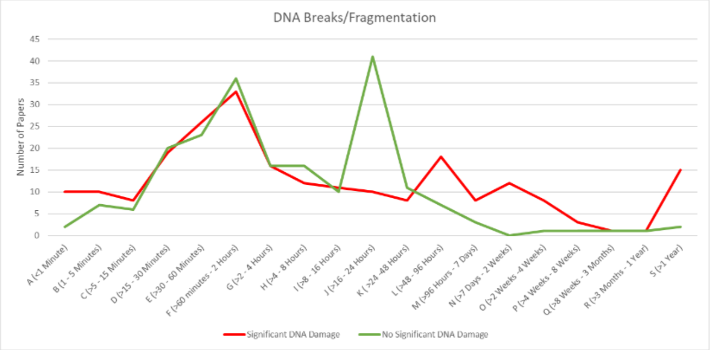

Supp_Figure 5B: DNA breaks - number of papers

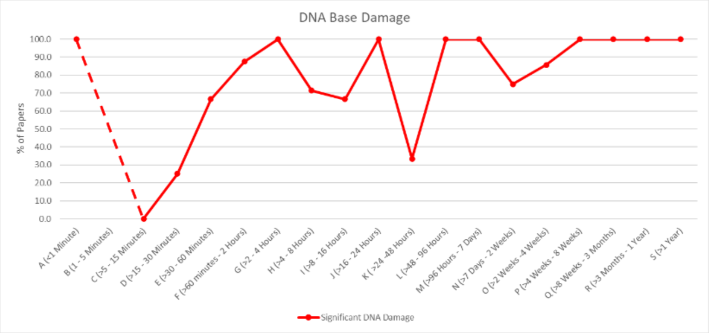

Supp_Figure 6A: DNA base damage balance of evidence

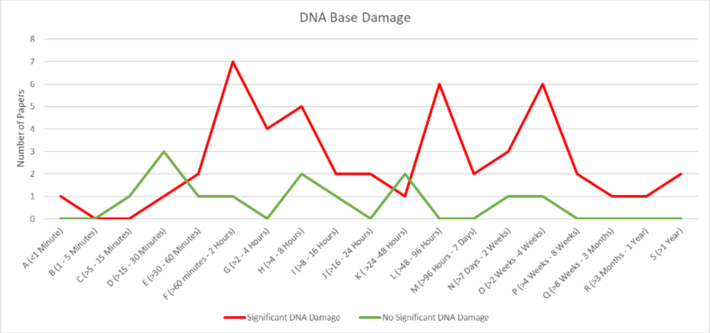

Supp_Figure 6B: DNA base damage - number of papers

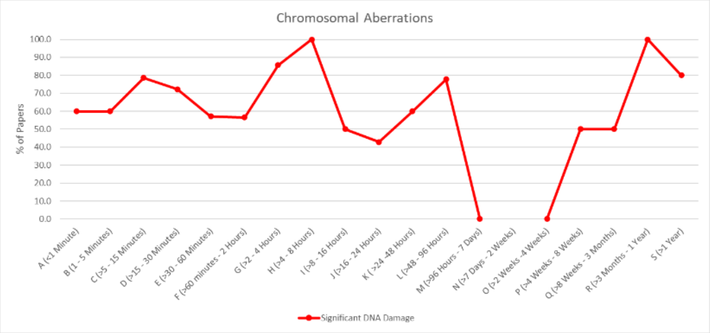

Supp_Figure 7A: Chromosome aberr. balance of evidence

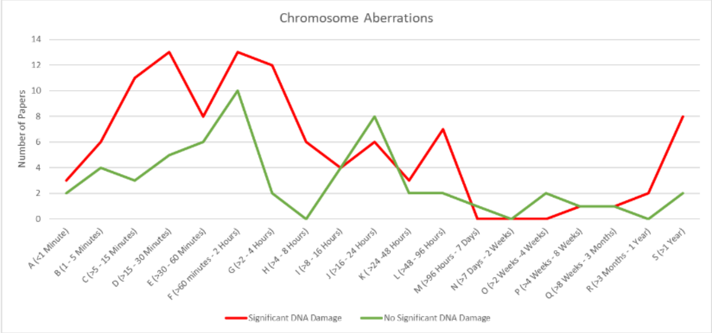

Supp_Figure 7B: Chromosome aberr. - number of papers

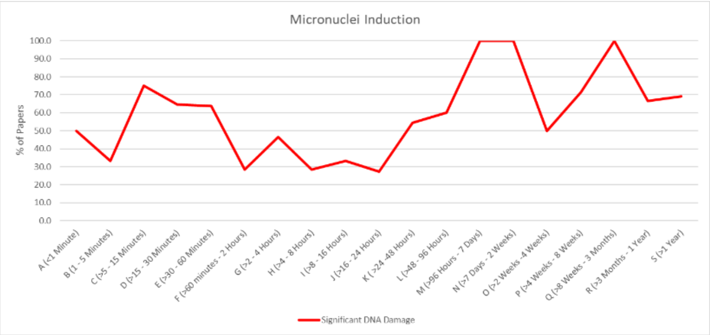

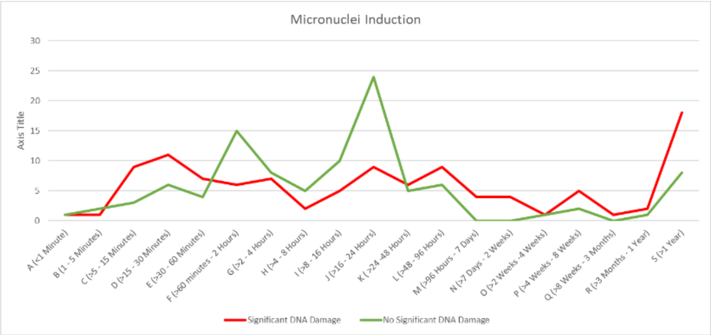

Supp_Figure 8A: Micronuclei balance of evidence

Supp_Figure 8B: Micronuclei - number of papers

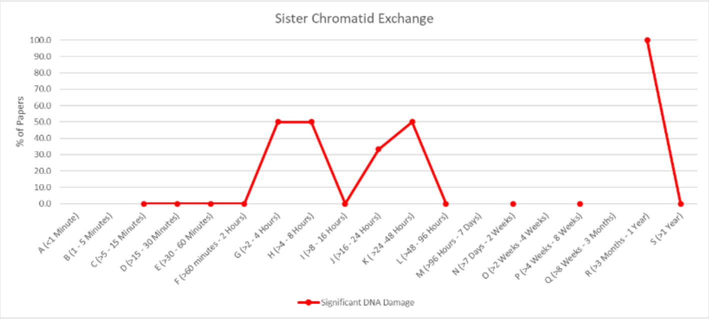

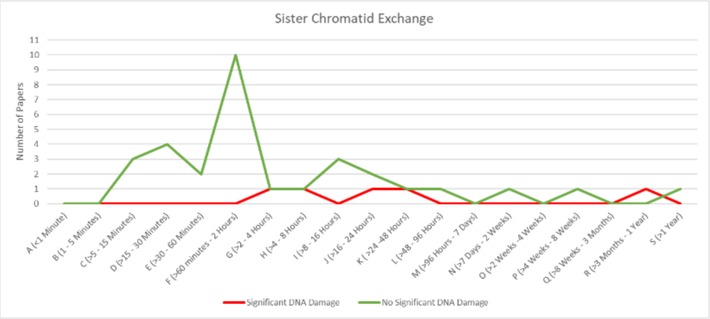

Supp_Figure 9A: Sister chromatid balance of evidence

Supp_Figure9B: Sister chromatid - number of papers

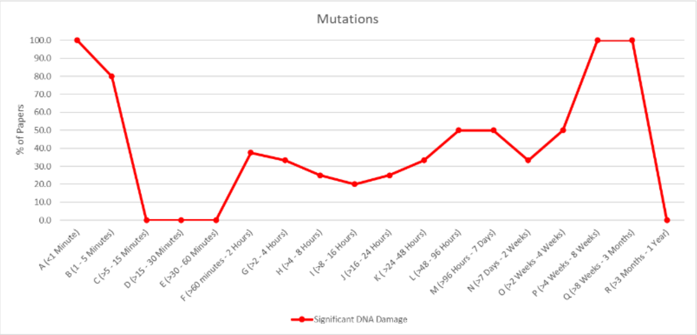

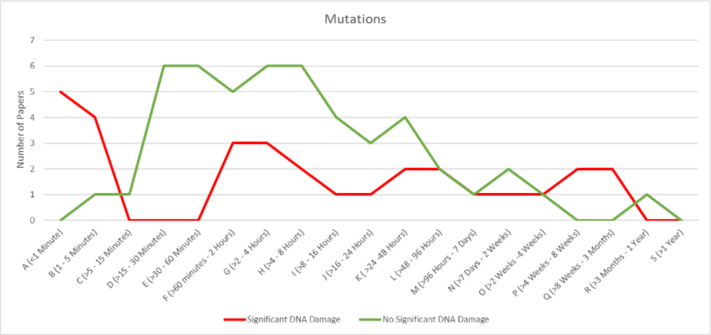

Supp_Figure 10A: Mutations balance of evidence

Supp_Figure 10B: Mutations - number of papers

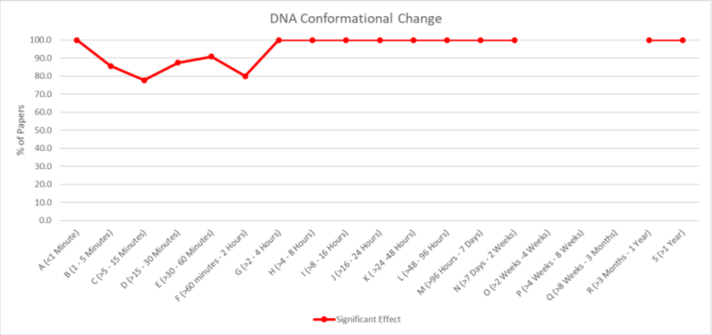

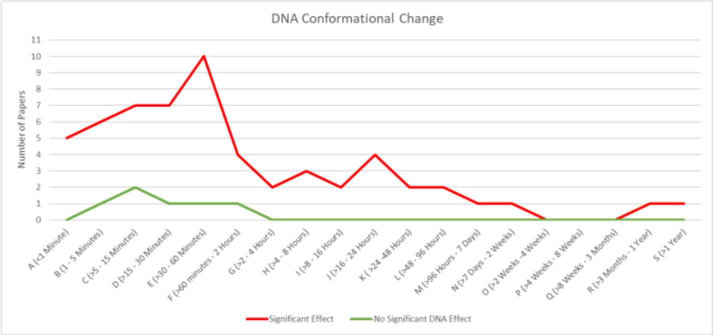

Supp_Figure 11A: DNA conformation balance of evidence

Supp_Figure 11B: DNA conformation - number of papers

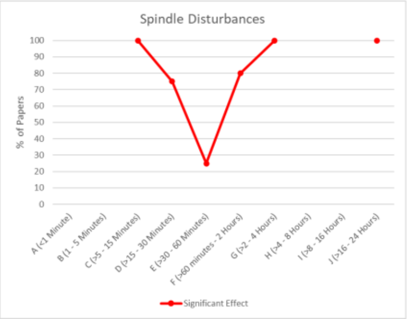

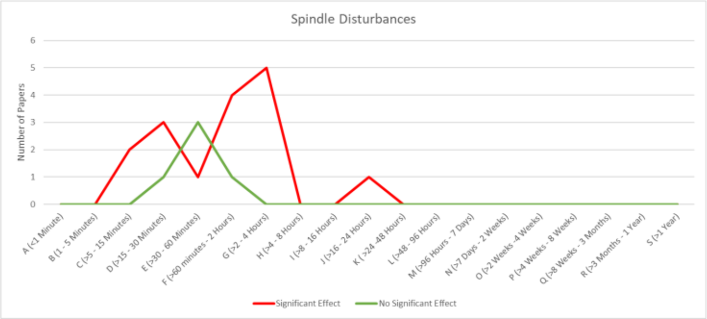

Supp_Figure 12A: Spindles dist. balance of evidence

Supp_Figure 12B: Spindle dist. - number of papers

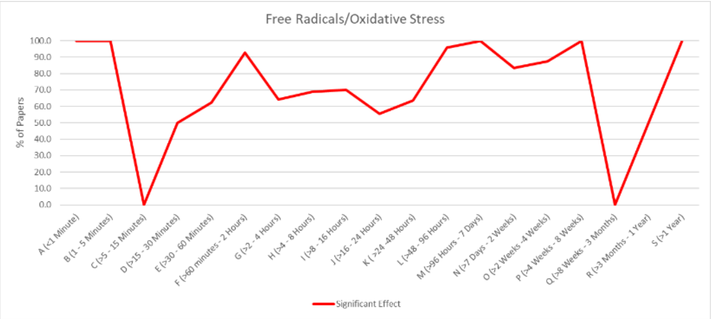

Supp_Figure 13A: Free radicals balance of evidence

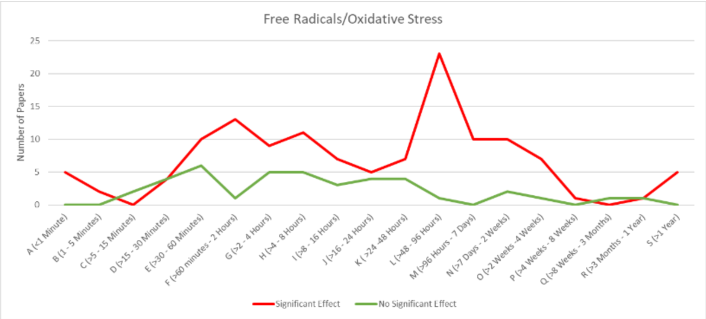

Supp_Figure 13B: Free radicals - number of papers

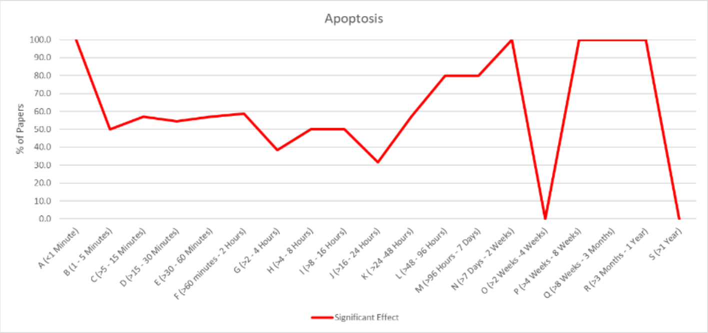

Supp_Figure 14A: Apoptosis balance of evidence

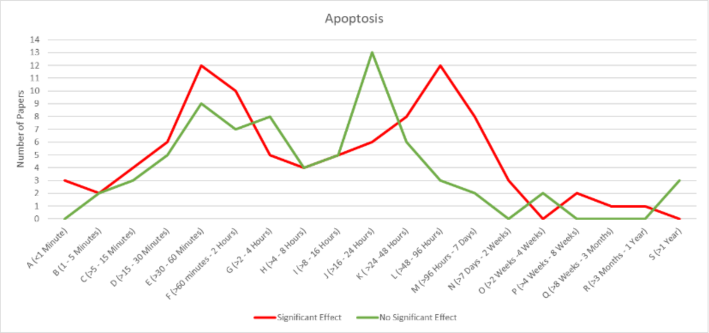

Supp_Figure 14B: Apoptosis - number of papers

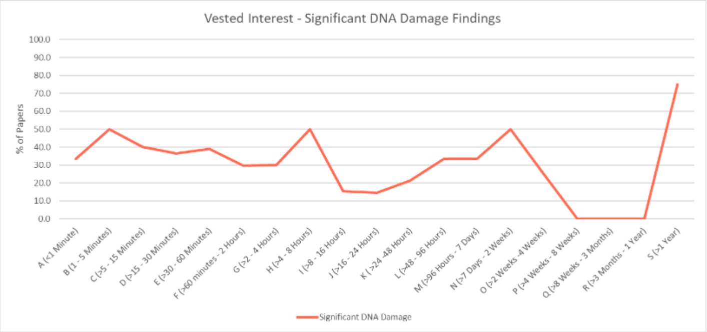

Supp_Figure 15A: DNA damage (V.I.) – balance of evidence

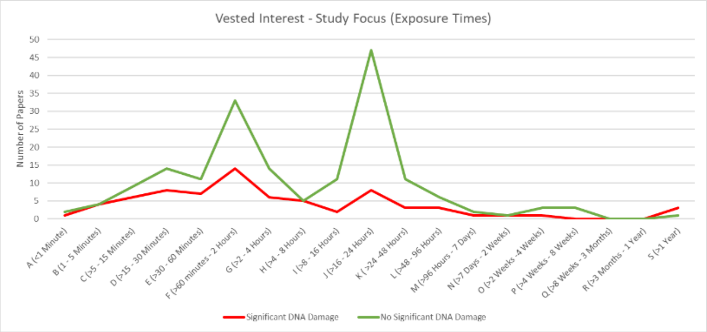

Supp_Figure 15B: DNA damage (V.I.) - number of papers

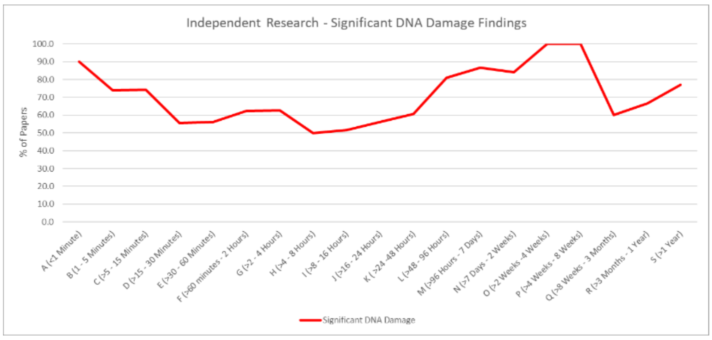

Supp_Figure 16A: DNA damage (Ind.) – balance of evidence

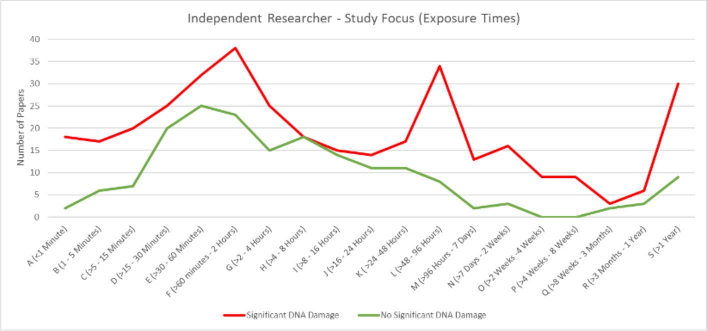

Supp_Figure 16B: DNA damage (Ind.) - number of papers

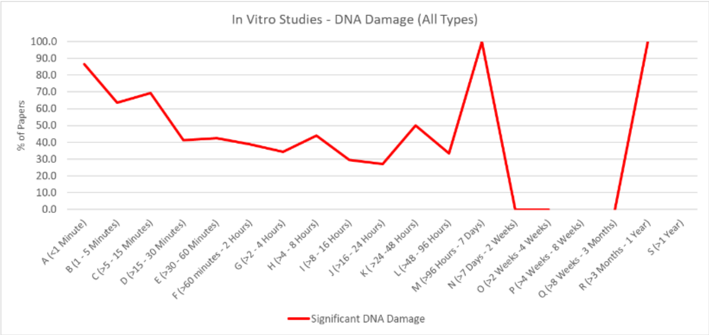

Supp_Figure 17A: DNA damage - *In vitro* balance of evidence

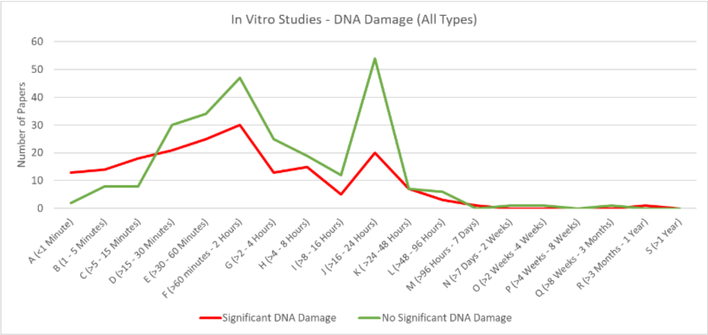

Supp_Figure 17B: DNA damage - *In vitro* studies (# papers)

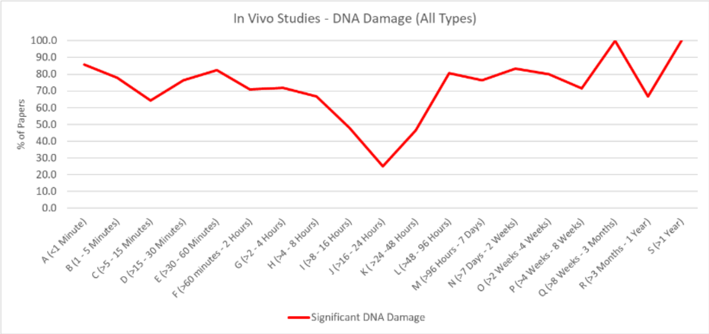

Supp_Figure 18A: DNA damage - *In vivo* balance of evidence

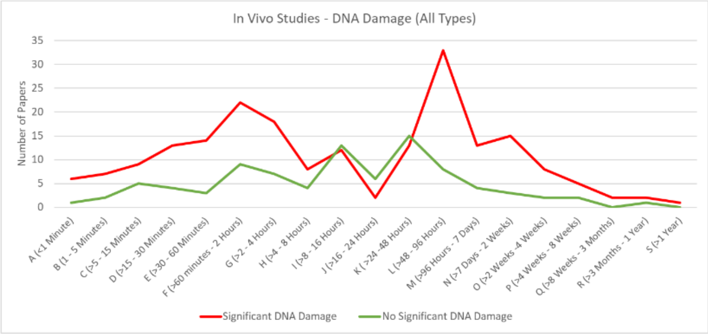

Supp_Figure 18B: DNA damage - *In vivo* studies (# papers)

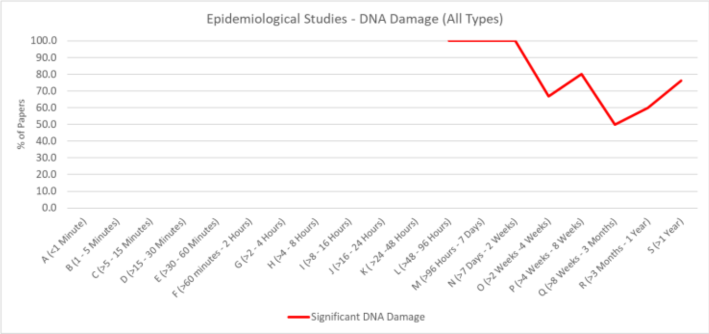

Supp_Figure 19A: DNA damage - *In vivo* balance of evidence

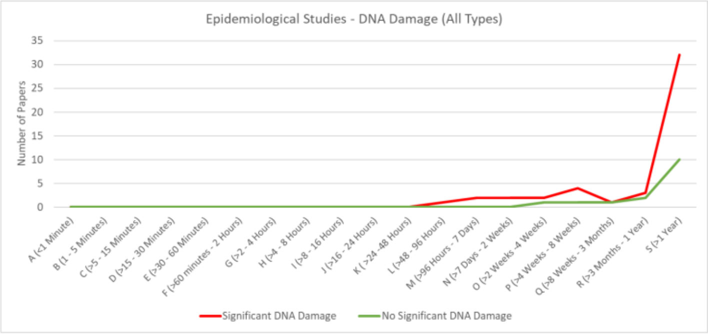

Supp_Figure 19B: DNA damage - *In vivo* studies (# papers)

Intensity

Supp_Table 13: Exposure intensity (all studies) – heat map colour coding applied to "# Papers" as well as separately for "% Significant damage" for each experiment type

Exposure Intensity	≤ ICNIRP Occupational Limits			> ICNIRP Occupational Limits			Extremely Low			Very Low			Low/Non Thermal			Medium			High			Very High			Extremely High		
Finding / Study Type	In Vitro	In Vivo	Epidemiological	In Vitro	In Vivo	Epidemiological	In Vitro	In Vivo	Epidemiological	In Vitro	In Vivo	Epidemiological	In Vitro	In Vivo	Epidemiological	In Vitro	In Vivo	Epidemiological	In Vitro	In Vivo	Epidemiological	In Vitro	In Vivo	Epidemiological	In Vitro	In Vivo	Epidemiological
Significant DNA damage	86	129	40	39	11		2	12	3	6	8	5	24	67	16	40	54	31	19	13	6	11	6	5	44	17	
No-Significant damage	133	39	12	30	12		1	3	0	2	5	0	42	15	2	77	21	12	31	6	1	36	10	0	34	10	
Total # Papers	219	168	52	69	23		3	15	3	8	13	5	66	82	18	117	75	43	50	19	7	47	16	5	78	27	
% Significant damage	39	77	78	57	50		67	80	100	75	62	100	36	82	89	34	72	72	38	68	86	23	38	100	56	63	
% No-Significant damage	61	23	22	43	50		33	20	0	25	38	0	64	18	11	66	28	28	62	32	14	77	62	0	44	37	
Balance of Evidence (All Studies)	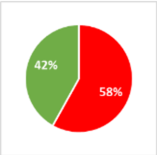			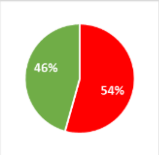			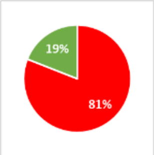			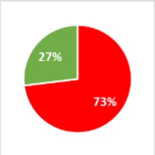			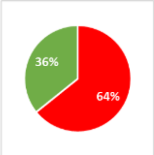			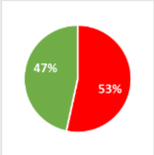			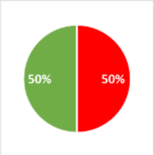			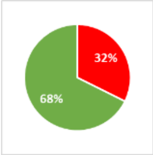			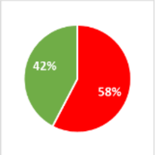		

DNA Damage vs Intensity

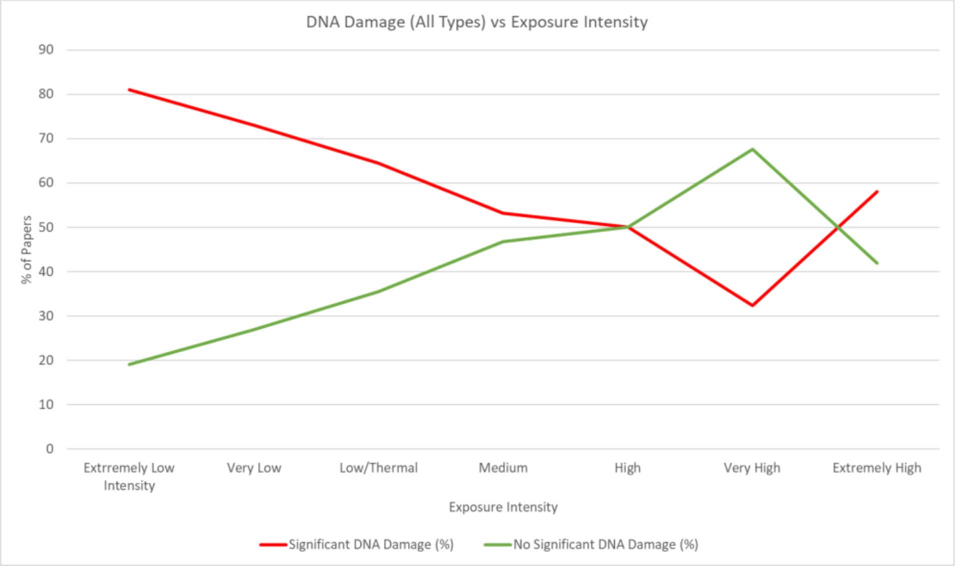

Supp_Figure 20A: Percentage of papers showing DNA damage across exposure intensity

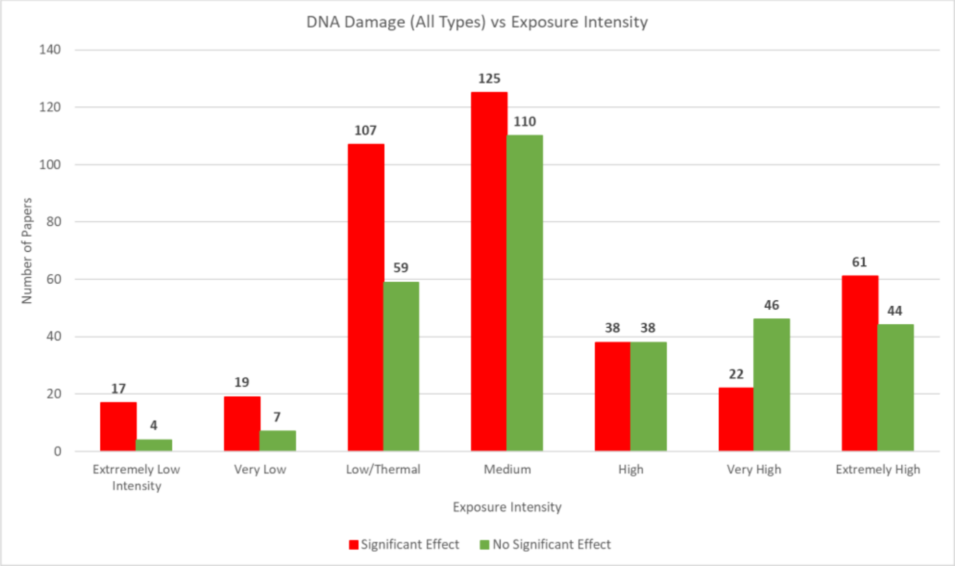

Supp_Figure 20B: Number of papers showing DNA damage across exposure intensity

Intensity by duration

Supp_Table 14: DNA damage from exposure intensity vs duration (all studies) – heat map colour coding applied to “# Papers” as well as separately for “% Significant damage” for each experiment type

Exposure Intensity	Extremely Low				Very Low				Low/Non thermal				Medium				High				Very High				Extremely High			
Finding / Exposure Duration	Acute	Short	Medium	Long	Acute	Short	Medium	Long	Acute	Short	Medium	Long	Acute	Short	Medium	Long	Acute	Short	Medium	Long	Acute	Short	Medium	Long	Acute	Short	Medium	Long
Significant DNA damage	0	4	9	4	4	11	6	5	21	42	34	18	27	47	27	37	11	13	9	7	5	8	4	6	47	12	1	1
No-Significant damage	1	3	1	0	2	3	1	1	15	42	10	5	22	71	18	16	8	32	6	2	19	29	2	1	18	26	4	0
Protective Effect	0	0	0	0	0	0	1	0	1	1	0	0	0	0	0	0	0	0	1	0	1	0	0	0	0	0	0	0
Total # Papers	1	7	10	4	6	14	8	6	37	85	44	23	49	118	45	53	19	45	16	9	25	37	6	7	65	38	5	1
% Significant damage	0	57	90	100	67	79	75	83	57	49.5	77	78	55	40	60	70	61	29	56	78	20	22	67	86	72	32	25	100
% No-Significant damage	100	43	10	0	33	21	12.5	17	41	49.5	23	22	45	60	40	30	39	71	38	22	76	78	33	14	27	68	75	0
% Protective Effect	0	0	0	0	0	0	12.5	0	2	1	0	0	0	0	0	0	0	0	6	0	4	0	0	0	0	0	0	0
	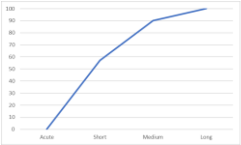				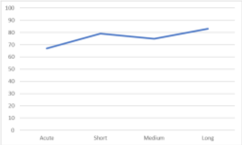				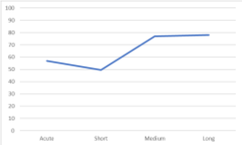				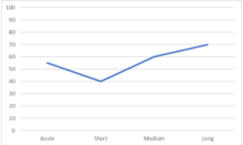				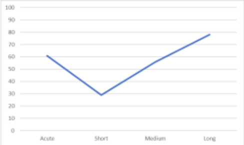				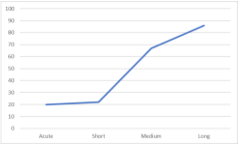				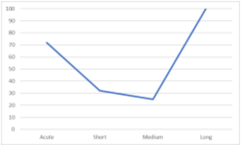			

Telecommunication Transmission protocols

Modulation Map (Experimental Studies)

Supp_Table 15: DNA damage vs signal modulation – heat map colour coding applied to “# Papers” as well as separately for “% Significant damage” for each experiment type

Modulation	GSM		GSM-Talk		GSM DTX		GSM-217		GSM-Basic		CDMA		UMTS-WCDMA		TDMA		Wi-Fi	
Finding / Study Type	Simulated Signal	Real Signal	Simulated Signal	Real Signal	Simulated Signal	Real Signal	Simulated Signal	Real Signal	Simulated Signal	Real Signal	Simulated Signal	Real Signal	Simulated Signal	Real Signal	Simulated Signal	Real Signal	Simulated Signal	Real Signal
Significant DNA damage	47	32	10	10	0	1	22	6	4	0	5	1	5	7	3	1	2	9
No-Significant damage	43	6	5	1	2	0	22	0	5	1	10	1	24	1	4	0	3	3
Total # papers	90	38	15	11	2	1	44	6	9	1	15	2	29	8	7	1	5	12
% Significant damage	52	84	67	91	0	100	50	100	44	0	33	50	17	88	43	100	33	75
% No-Significant damage	48	16	33	9	100	0	50	0	56	100	67	50	83	12	57	0	67	25
Balance of Evidence (All Studies)	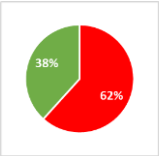		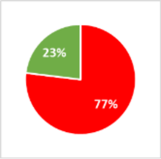		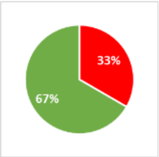		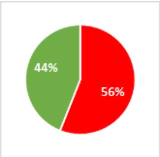		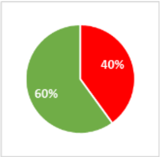		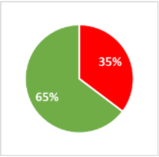		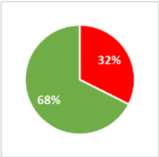		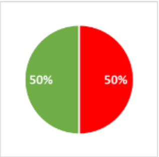		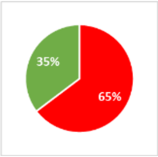	

Signal Generator vs Real Transmitters

Supp_Table 16: DNA damage vs signal source – heat map colour coding applied to “# Papers” as well as separately for “% Significant damage” for each experiment type

Exposure Source	Signal Generator		Real Device/Transmitter		
Finding / Study Type	In Vitro	In Vivo	In Vitro	In Vivo	Epidemiological
Significant DNA damage	86	82	37	54	40
No-Significant damage	135	38	13	10	12
Total # papers	221	120	50	64	52
% Significant damage	39	68	74	84	77
% No-Significant damage	61	32	26	16	23
Balance of Evidence (All Studies)	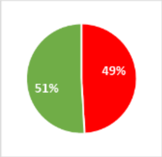		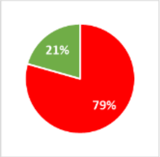		

Exposure Regime

Supp_Table 17: DNA damage vs exposure regime (all studies) – heat map colour coding applied to “# Papers” as well as separately for “% Significant damage” for each experiment type

Exposure Regime	Pulsed Wave			Continuous Wave			Single Exposure			Multiple Exposures			Continuous Exposure			Intermittent Exposure			Variable Exposure		
Finding / Study Type	In Vitro	In Vivo	Epidemiological	In Vitro	In Vivo	Epidemiological	In Vitro	In Vivo	Epidemiological	In Vitro	In Vivo	Epidemiological	In Vitro	In Vivo	Epidemiological	In Vitro	In Vivo	Epidemiological	In Vitro	In Vivo	Epidemiological
Significant DNA damage	61	65	34	57	65	2	113	59		11	85	39	93	109		21	6	10	13	31	29
No-Significant damage	101	22	10	59	26	2	141	20		9	29	12	126	41		19	4	0	7	3	13
Total # papers	162	87	44	116	91	4	254	79		18	114	51	219	150		40	10	10	20	34	42
% Significant damage	38	75	77	49	71	50	44	75		55	75	76	42	73		55	60	100	65	91	69
% No-Significant damage	62	25	23	51	29	50	56	25		45	25	24	58	27		45	40	0	35	9	31
Balance of Evidence (All Studies)	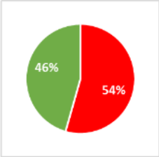			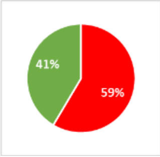			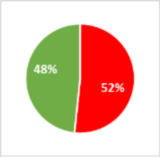			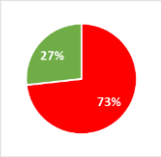			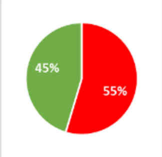			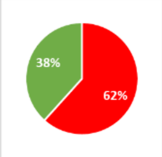			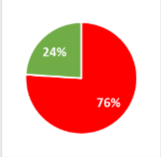		

5. Mechanisms

Potential Mechanisms (All Studies)

Supp_Table 18: Potential mechanisms map (all studies)

Relevant Biological Endpoint	Oxidative Stress			Heat Shock Proteins			Spindle Disturbances		
Finding / Study Type	In Vitro	In Vivo	Epidemiological	In Vitro	In Vivo	Epidemiological	In Vitro	In Vivo	Epidemiological
	31	60	7	7	6		5	5	
No-Significant effects	13	7	0	12	3		0	0	
Total # papers	44	67	7	19	9		5	5	
% Significant effects	70	90	100	37	67		100	100	
% No-Significant effects	30	10	0	63	33		0	0	
Balance of Evidence	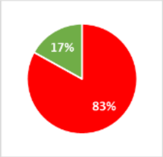			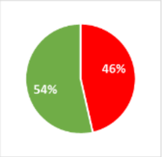			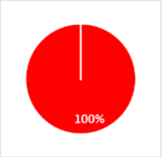		

Potential Mechanisms ("Quality" Studies)

Supp_Table 19: Potential mechanisms map (quality studies)

Potential Genotoxic Endpoint	Oxidative Stress		Heat Shock Proteins		Spindle Disturbances	
Finding / Study Type	In Vitro	In Vivo	In Vitro	In Vivo	In Vitro	In Vivo
Significant effects	8	13	2	2	2	
No-Significant effects	6	4	1	0	0	
Total # papers	14	17	3	2	2	
% Significant effects	57	76	67	100	100	
% No-Significant effects	43	24	33	0	0	
Balance of Evidence (All Studies)	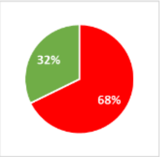		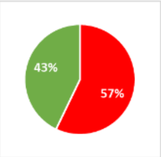		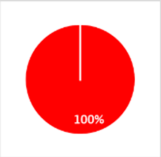	

Damage type and mechanism correspondences

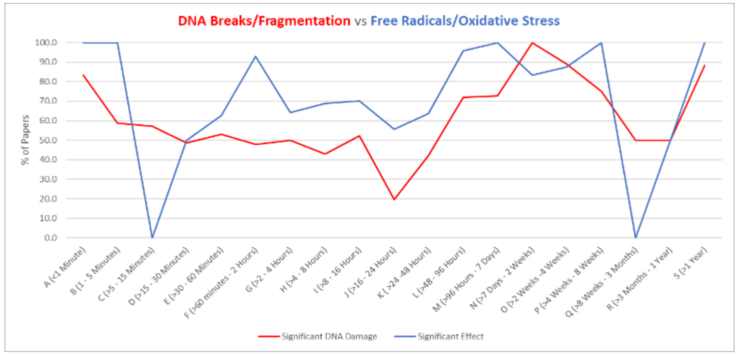

Supp_Figure 21:DNA breaks vs OS over time map

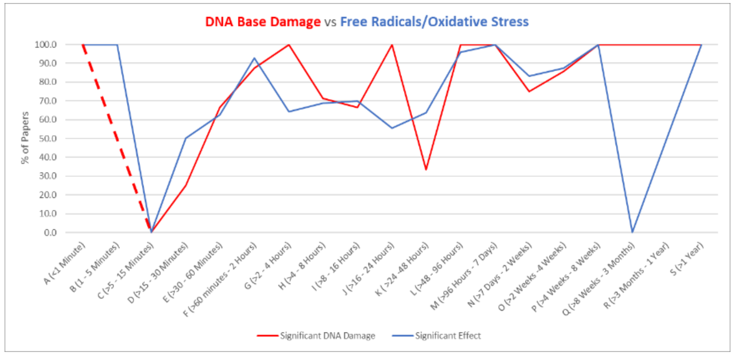

Supp_Figure 22:DNA base damage vs OS over time Map

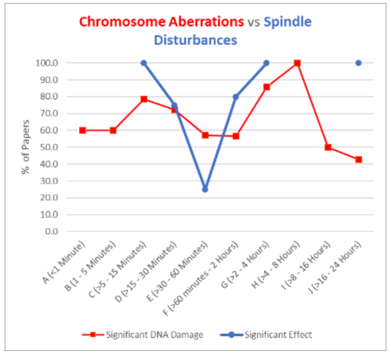

Supp_Figure 23:DNA breaks vs spindle disturbances over time map

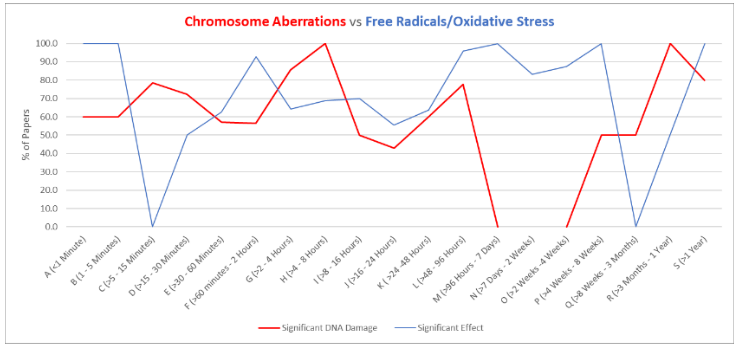

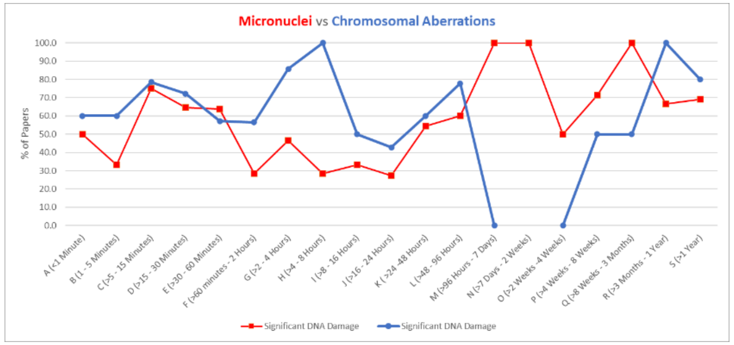

Supp_Figure 24: Chromosome aberrations vs OS over time map Supp_Figure 25: Micronuclei vs chromosome aberrations over time map

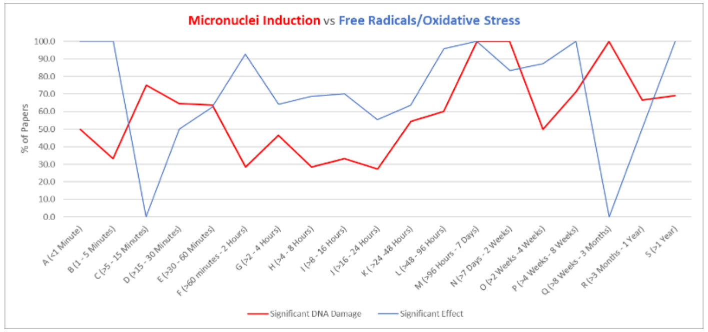

Supp_Figure 26: Micronuclei vs OS over time map

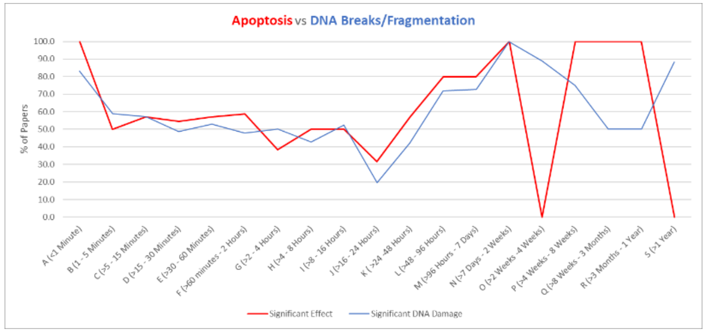

Supp_Figure 27: Apoptosis vs DNA breaks over time map

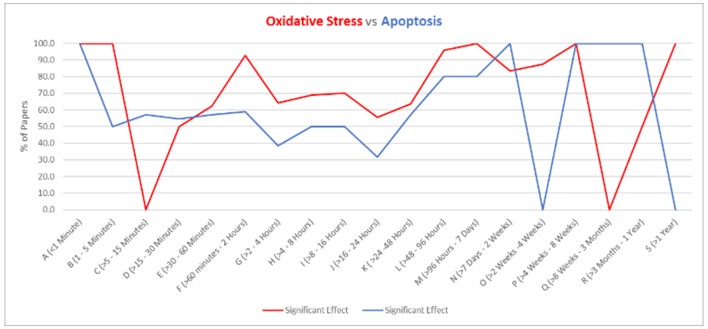

Supp_Figure 28: OS vs apoptosis over time map

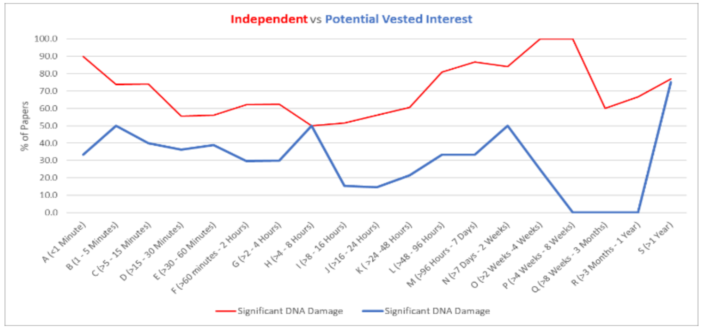

Supp_Figure 29: Vested interest vs independent research (DNA damage over time)

Oxidative Stress VS DNA Damage

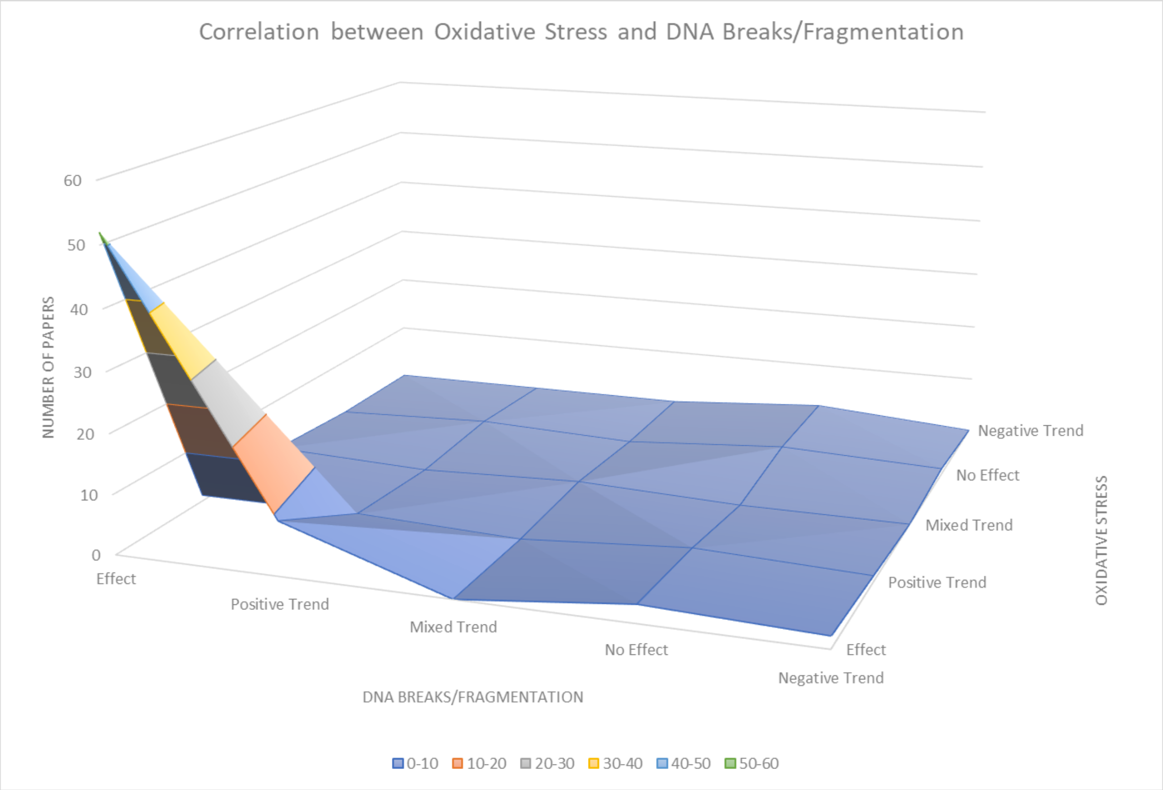

Supp_Figure 30: Relationship between DNA breaks and free radicals/oxidative stress

Heat Shock Proteins

Supp_Table 20: Heat shock protein expression vs exposure intensity – heat map colour coding applied to "# Papers" as well as separately for "% Significant damage" for each experiment type

Exposure Intensity	Extremely Low		Very Low		Low/Non Thermal		Medium		High		Very High		Extremely High	
Finding / Study Type	In Vitro	In Vivo	In Vitro	In Vivo	In Vitro	In Vivo	In Vitro	In Vivo	In Vitro	In Vivo	In Vitro	In Vivo	In Vitro	In Vivo
Significant Effect		3	1	1	2	1	2		0	1			2	1
Non-Significant Effect		1	1	0	2	1	3		1	1			3	0
Total # Papers		4	2	1	4	2	5		1	2			5	1
% Significant Effect		75	50	100	50	50	40		0	50			40	100
% No-Significant Effect		25	50	0	50	50	60		100	50			60	0
Balance of Evidence (All Studies)	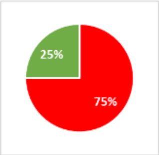		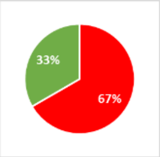		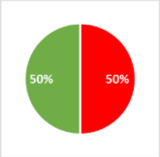		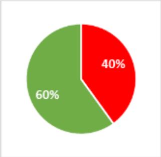		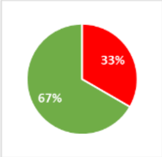				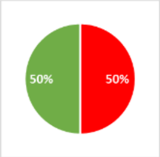	

6. Quality criteria

Blinding vs non-blinding, sufficient dosimetry vs poor dosimetry and sham vs control only (experimental studies)

Supp_Table 21: Blinding, dosimetry, sham, control and combined balance of evidence

Experiment Condition	Blinding		No Blinding		Sufficient Dosimetry		Poor Dosimetry		Exposed vs Sham		Exposed vs Control Only		Waveform Not Specified		Waveform Specified		Blinding+ Sham + Dosimetry	
Finding / Study Type	In Vitro	In Vivo	In Vitro	In Vivo	In Vitro	In Vivo	In Vitro	In Vivo	In Vitro	In Vivo	In Vitro	In Vivo	In Vitro	In Vivo	In Vitro	In Vivo	In Vitro	In Vivo
Significant DNA damage	40	45	83	100	80	103	42	42	59	81	63	62	14	20	109	125	30	42
No-Significant damage	75	21	74	27	112	31	37	17	108	33	39	15	30	4	137	45	66	11
Total # papers	115	66	157	127	192	134	79	59	167	114	102	77	44	24	246	170	96	53
% Significant damage	35	68	53	79	42	77	53	71	35	71	62	81	32	83	44	73.5	30	79
% No-Significant damage	65	32	47	21	58	23	47	29	65	29	38	19	68	17	56	27.5	70	21
Balance of Evidence (All Studies)	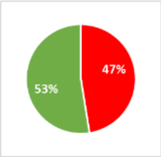		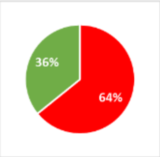		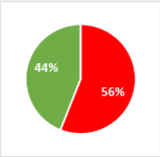		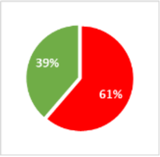		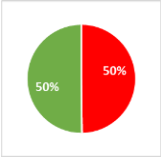		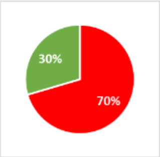		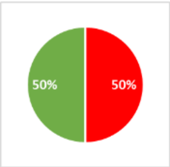		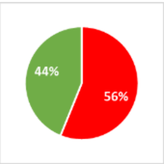		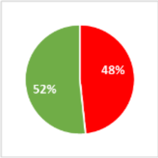	

Quality vs All experimental papers (intensity mix)

Supp_Table 22: Quality vs All experimental papers (intensity mix)

	Extremely Low	Very Low	Low	Medium	High	Very High	Extremely High
Experimental Papers (All)	18	21	151	194	69	63	102
Quality Papers	7	4	51	82	24	19	10
All Experimental Studies % Breakdown	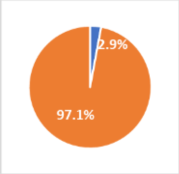 (a)	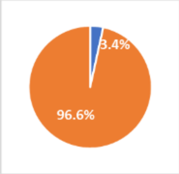 (c)	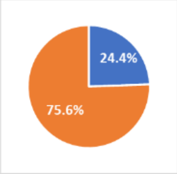 (e)	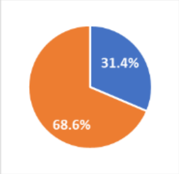 (g)	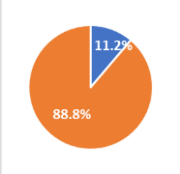 (i)	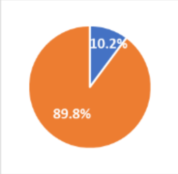 (k)	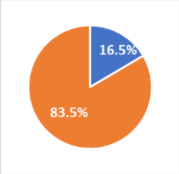 (m)
Quality Studies % Breakdown	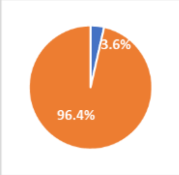 (b)	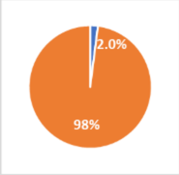 (d)	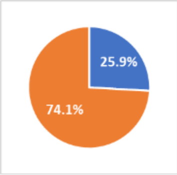 (f)	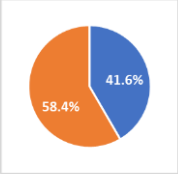 (h)	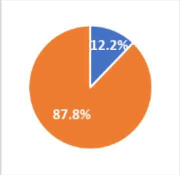 (j)	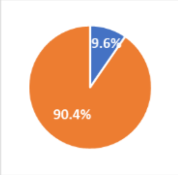 (l)	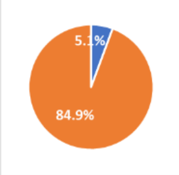 (n)

Quality vs All Experimental Papers (Duration Mix)

Supp_Table 23: Quality vs All experimental papers (duration mix)

	Acute	Short	Medium	Long
Experimental Papers (All)	164	242	107	20
Quality Papers	34	78	27	7
All Experimental Studies % Breakdown	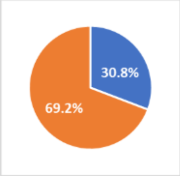 (a)	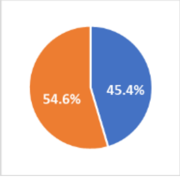 (c)	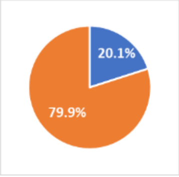 (e)	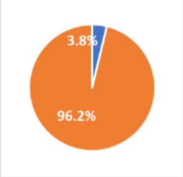 (g)
Quality Studies % Breakdown	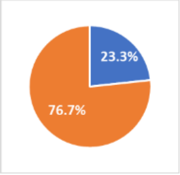 (b)	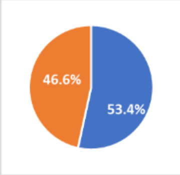 (d)	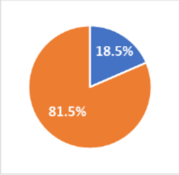 (f)	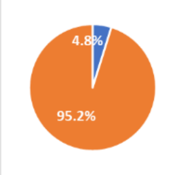 (h)

Other Important Experimental Parameter Differences (All Experimental Studies vs “Quality” Studies)

Supp_Table 24: Experimental parameter differences (All experimental studies vs “Quality” studies)

	In vitro	In vivo	Real Signal	Signal Generator	Single Exposure	Multiple Exposures	Primary Cell	Cell Line
Experimental Papers (All)	290	192	113	362	344	132	357	120
Quality Papers	84	48	14	119	94	38	93	41
All Experimental Studies %	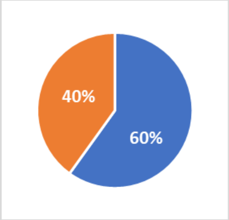<br>(a)		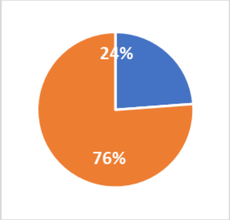<br>(c)		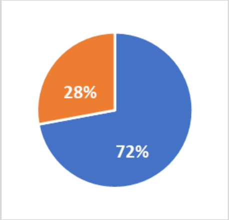<br>(e)		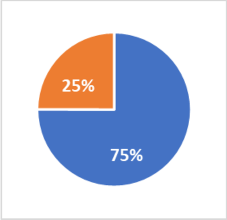<br>(g)	
Quality Studies %	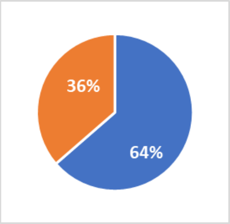<br>(b)		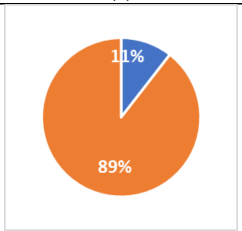<br>(d)		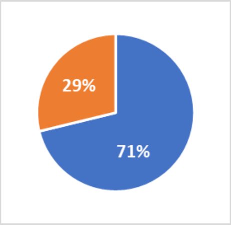<br>(f)		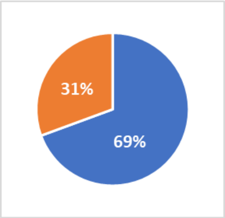<br>(h)	

Effects of quality attributes on DNA damage findings

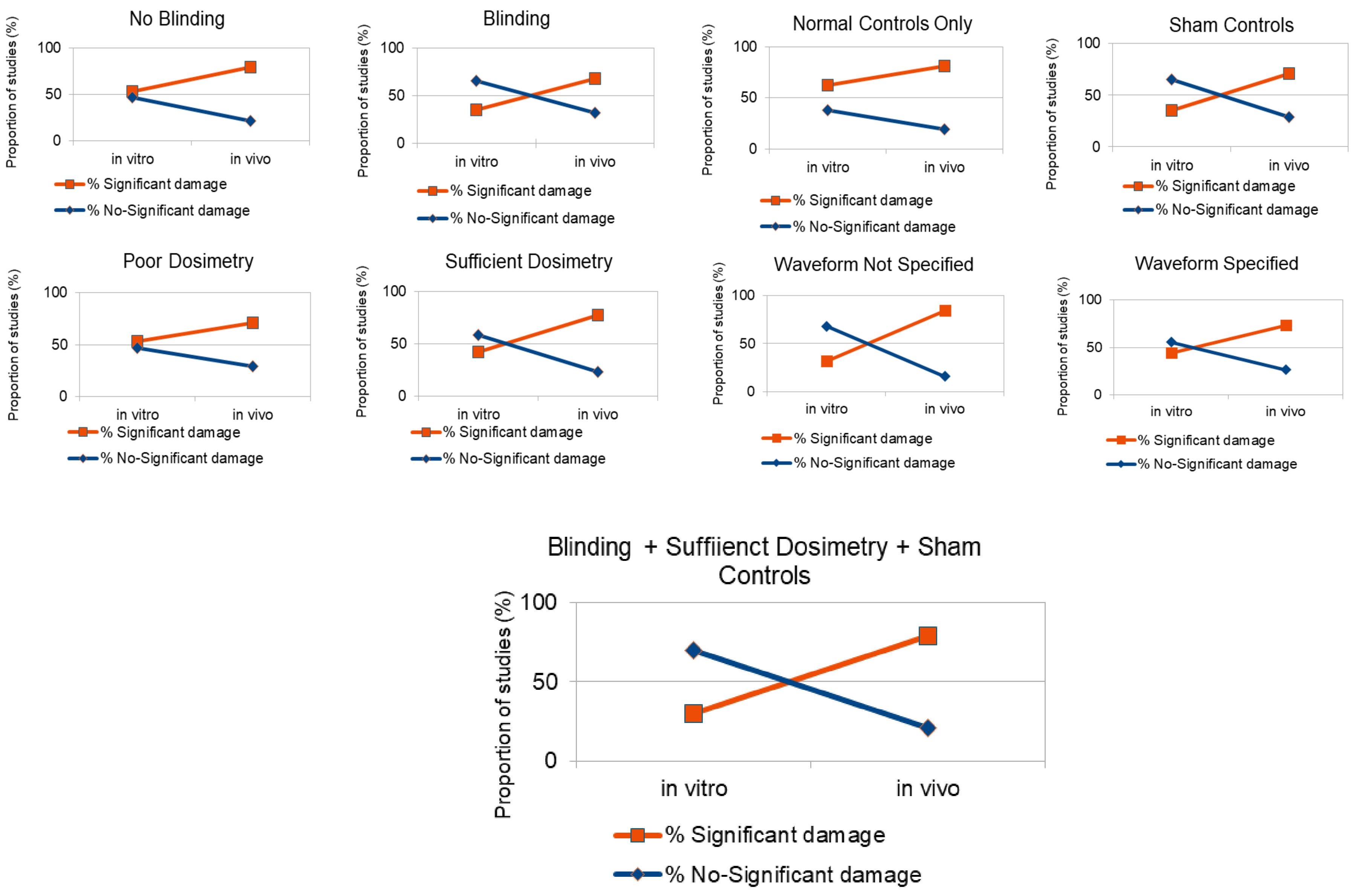

Supp_Figure 31: Quality Attributes on DNA damage outcomes

7. Risk of bias

Funding vs Findings

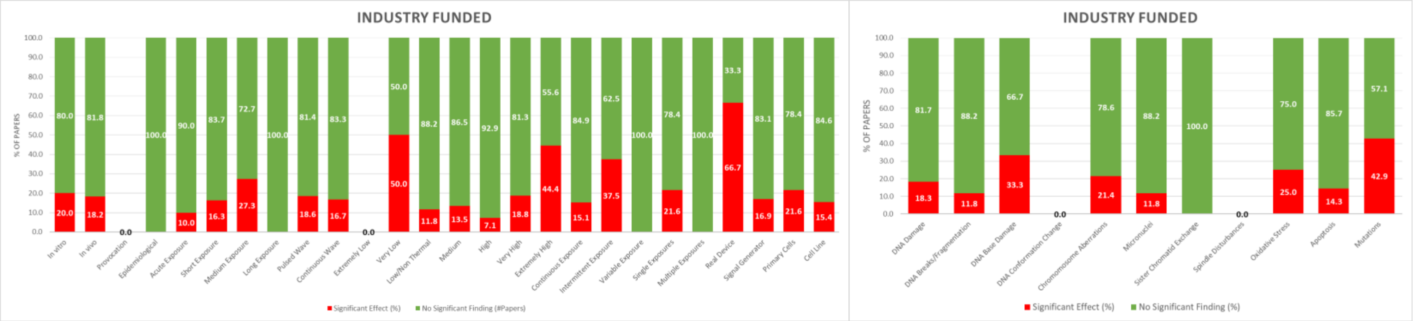

Supp_Figure 32: (a) Industry funding experimental parameters (% papers) (b) DNA damage findings (% papers)

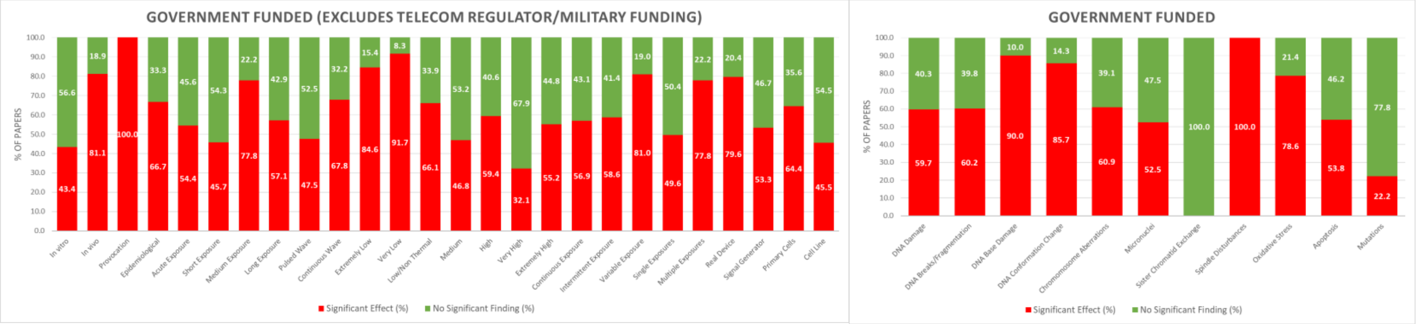

Supp_Figure 33: (a) Government funding experimental parameters (% papers) (b) DNA damage findings (% papers)

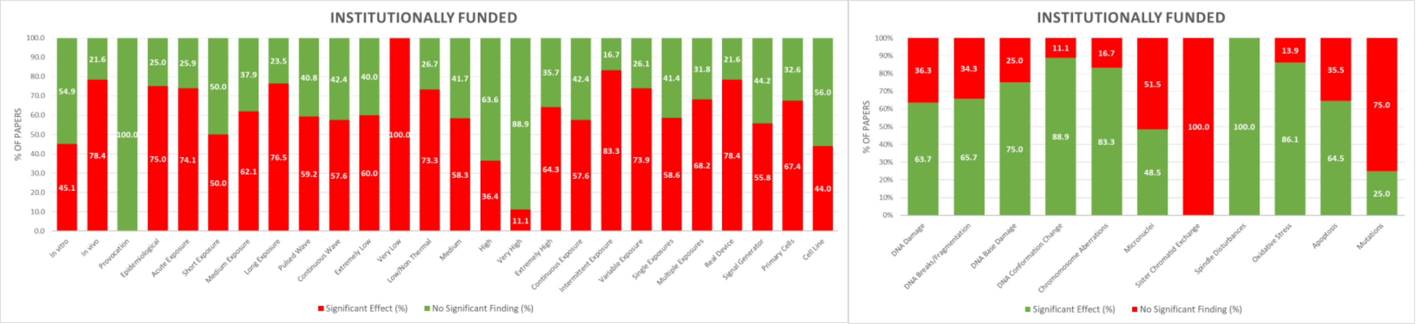

Supp_Figure 34: (a) Institutional funding experimental parameters (% papers) (b) DNA damage findings (% papers)

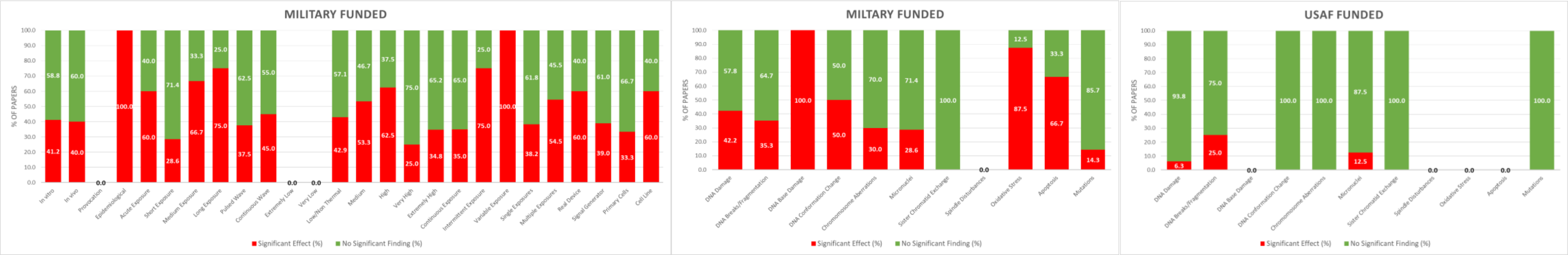

Supp_Figure 35: (a) Military funding experimental parameters (% papers) (b) DNA damage findings (% papers) (c) USAF funded DNA damage findings (% papers)

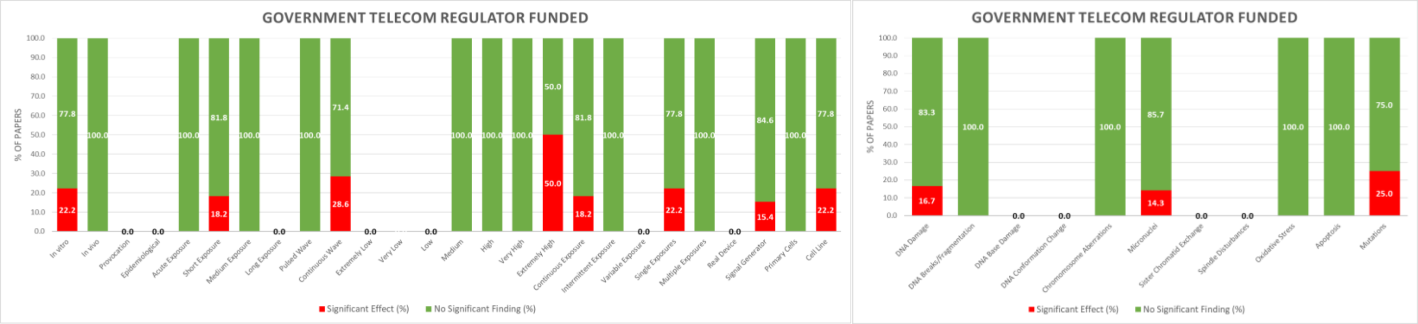

Supp_Figure 36: (a) Government telecom funding experimental parameters (% papers) (b) DNA damage findings (% papers)

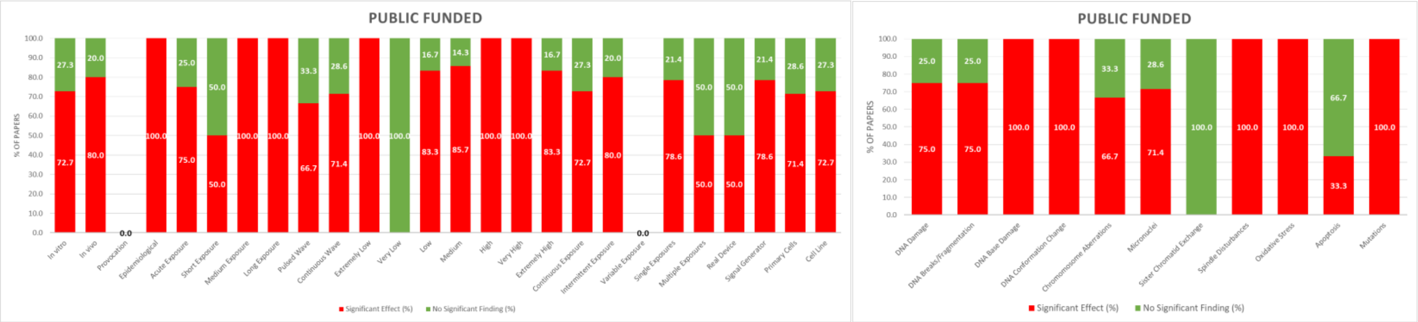

Supp_Figure 37: (a) Public funding experimental parameters (% papers) (b) DNA damage findings (% papers)

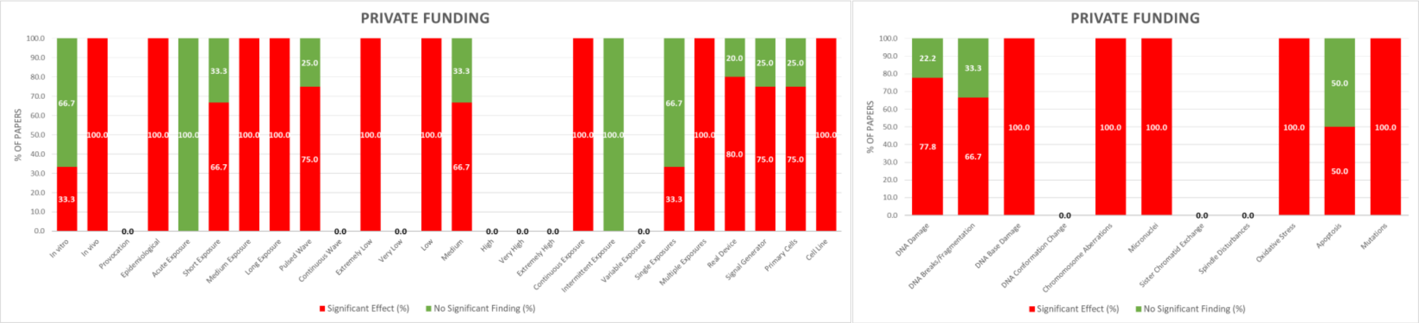

Supp_Figure 38: (a) Private funding experimental parameters (% papers) (b) DNA damage findings (% papers)

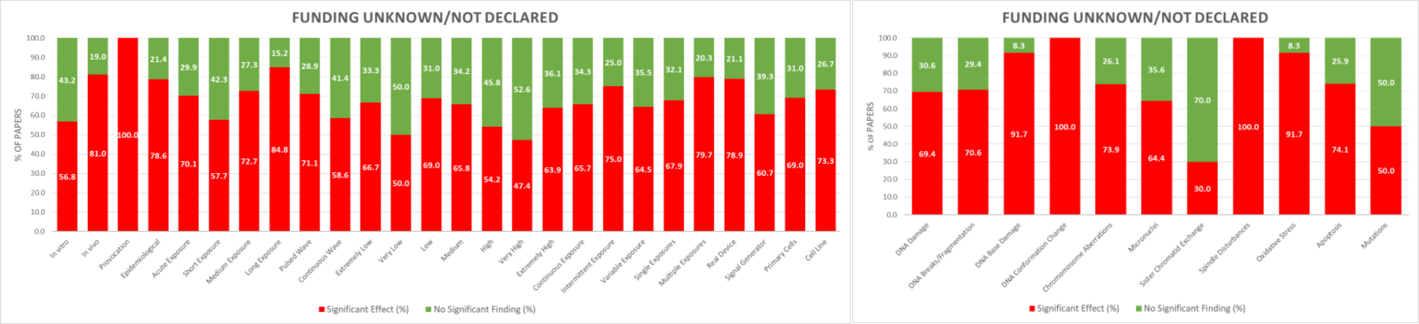

Supp_Figure 39: (a) Unknown funding experimental parameters (% papers) (b) DNA damage findings (% papers)

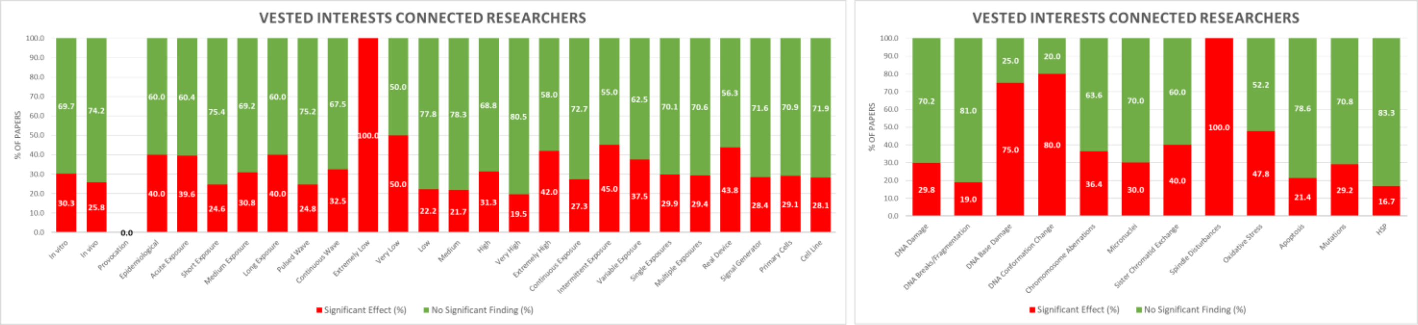

Supp_Figure 40: (a) Vested interest research experimental parameters (% papers) (b) DNA damage findings (% papers)

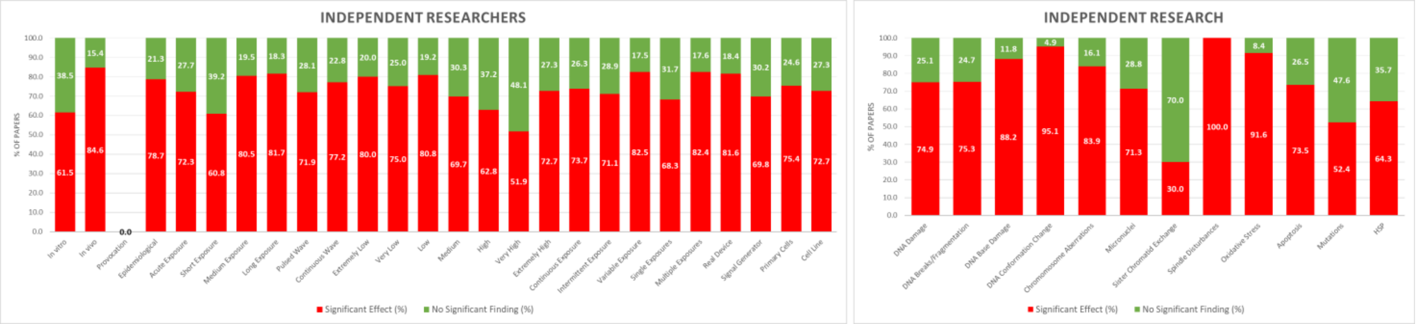

Supp_Figure 41: (a) Independent research experimental parameters (% papers) (b) DNA damage findings (% papers)

Funding vs Experimental Parameters and Findings

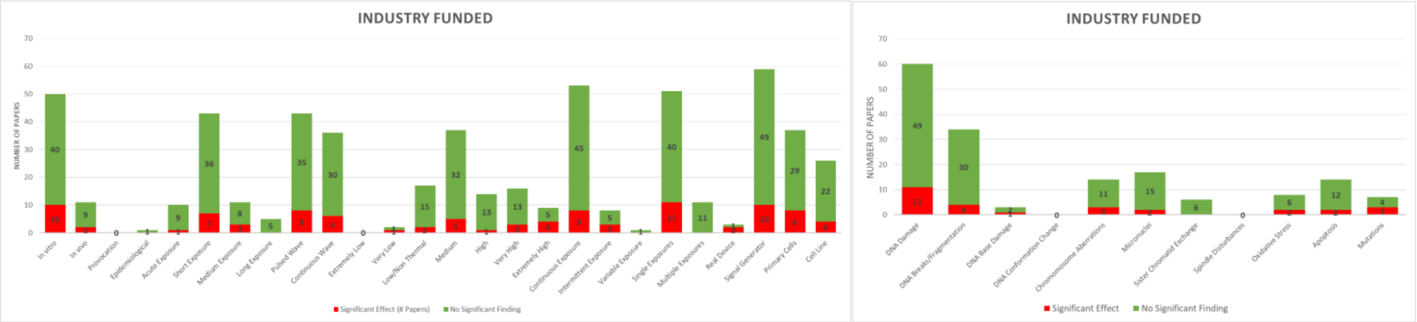

Supp_Figure 42: (a) Industry funding experimental parameters (# papers) (b) DNA damage findings (# papers)

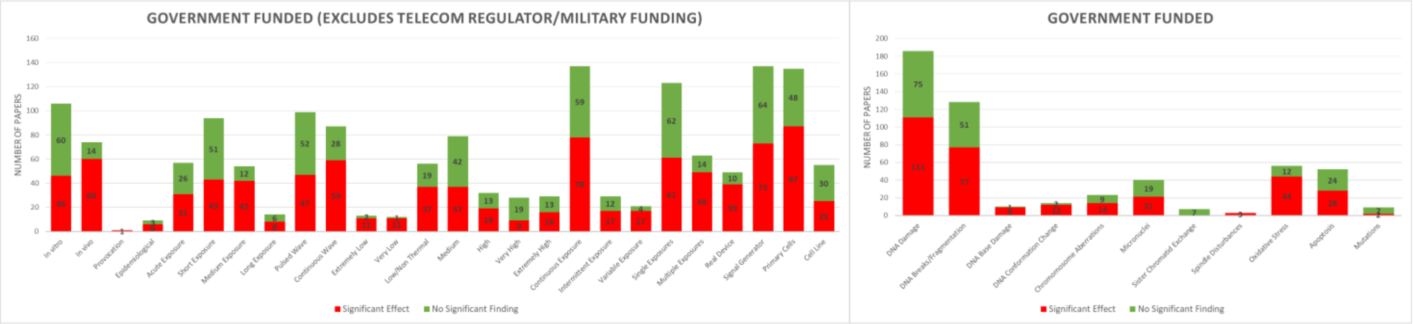

Supp_Figure 43: (a) Government funding experimental parameters (# papers) (b) DNA damage findings (# papers)

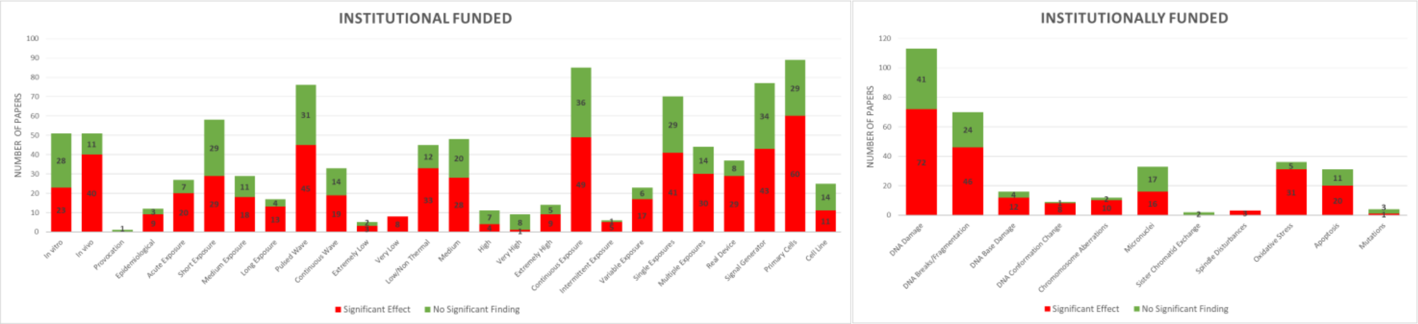

Supp_Figure 44: (a) Institutional funding experimental parameters (# papers) (b) DNA damage findings (# papers)

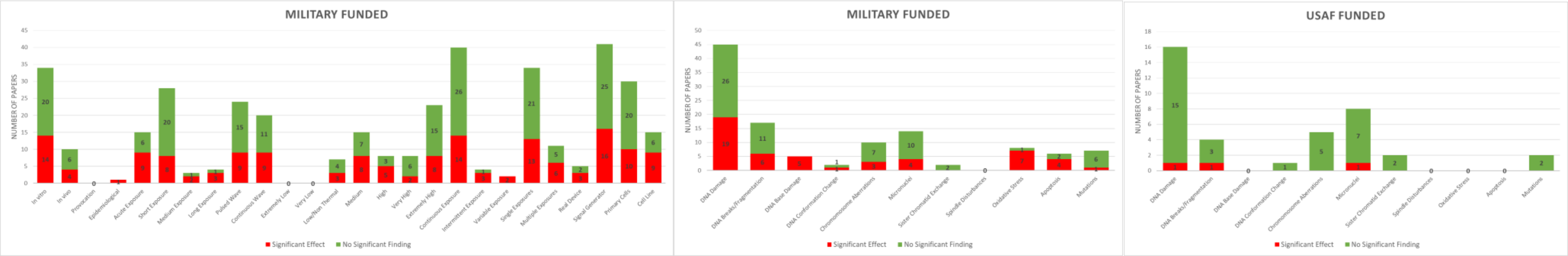

Supp_Figure 45: (a) Military funding experimental parameters (# papers) (b) DNA damage findings (# papers) (c) USAF funded DNA damage findings (# papers)

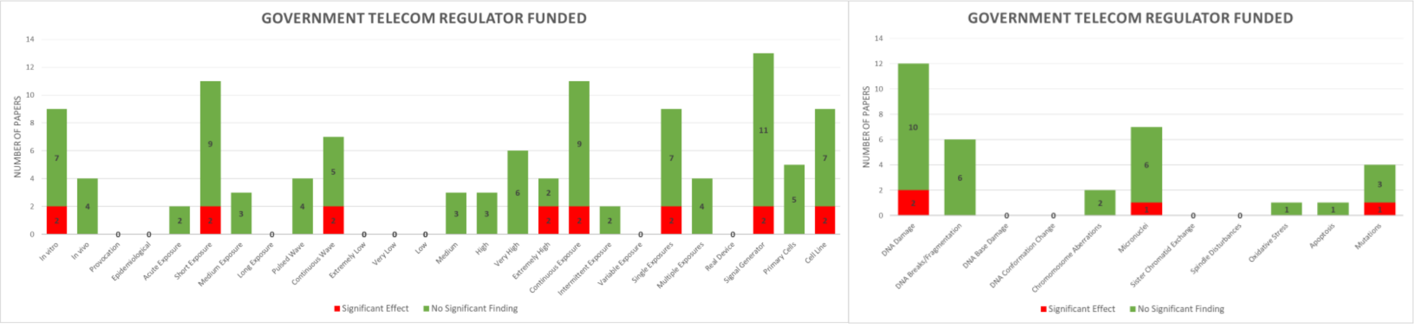

Supp_Figure 46: (a) Government telecom funding experimental parameters (# papers) (b) DNA damage findings (# papers)

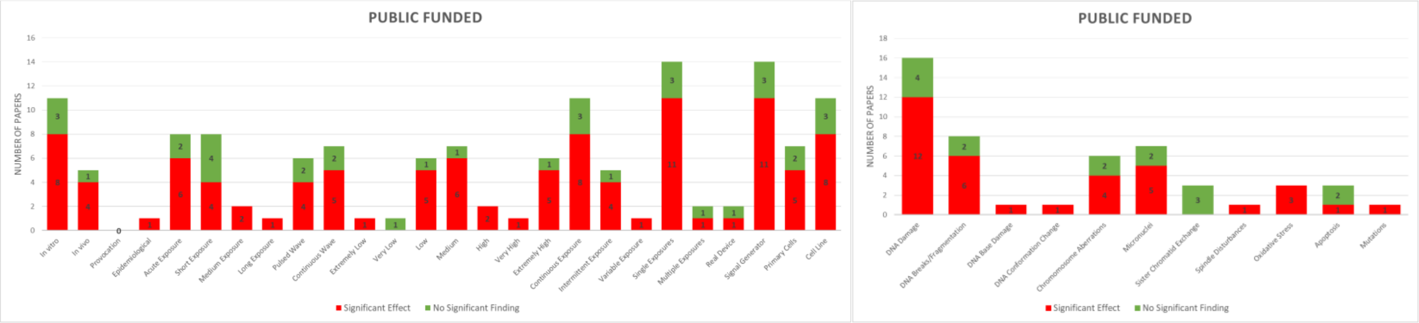

Supp_Figure 47: (a) Public funding experimental parameters (# papers) (b) DNA damage findings (# papers)

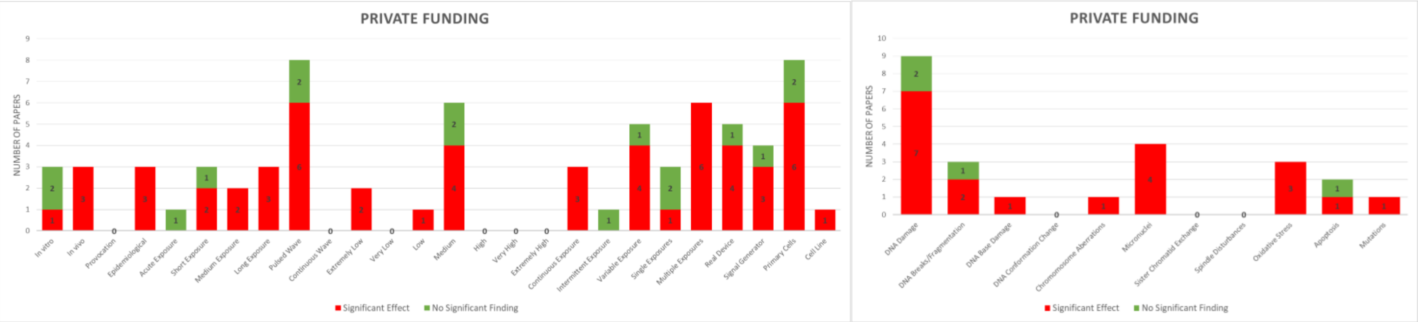

Supp_Figure 48: (a) Private funding experimental parameters (# papers) (b) DNA damage findings (# papers)

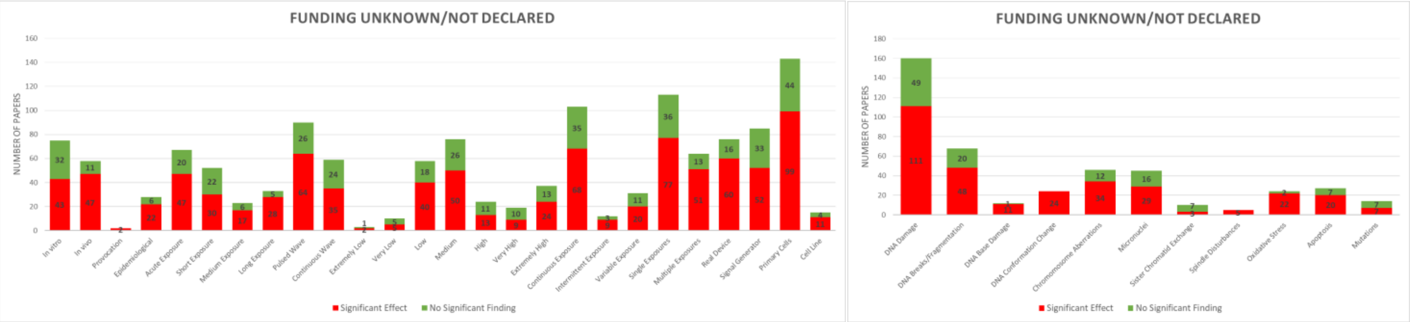

Supp_Figure 49: (a) Unknown funding experimental parameters (# papers) (b) DNA damage findings (# papers)

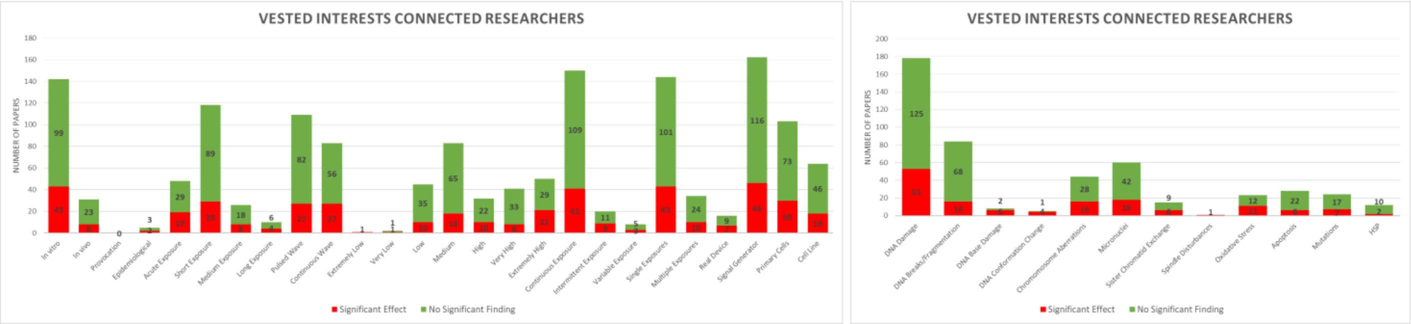

Supp_Figure 50: (a) Vested interests experimental parameters (# papers) (b) DNA damage findings (# papers)

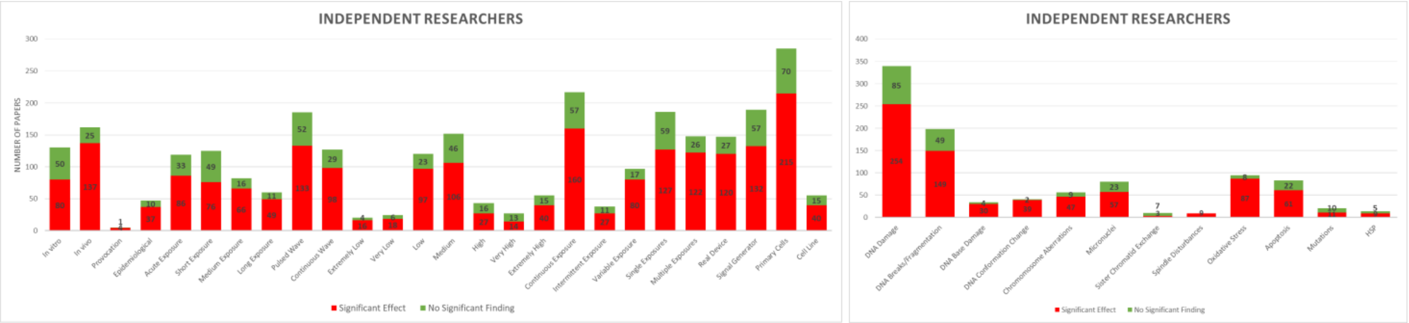

Supp_Figure 51: (a) Independent research experimental parameters (# papers) (b) DNA damage findings (# papers)

Funding Summary (All Papers)

Supp_Table 25: DNA damage vs funding source (all papers) – heat map colour coding applied to "# Papers" as well as separately for "% Significant damage" for each experiment type

Funding Source	Industry			Telecom Regulator			Military			Government (excl. Military/ Regulator)			Private			Public			Institutional			Unknown/Not Declared			USAF	
Finding / Study Type	In Vitro	In Vivo	Epidemiological	In Vitro	In Vivo	Epidemiological	In Vitro	In Vivo	Epidemiological	In Vitro	In Vivo	Epidemiological	In Vitro	In Vivo	Epidemiological	In Vitro	In Vivo	Epidemiological	In Vitro	In Vivo	Epidemiological	In Vitro	In Vivo	Epidemiological	In Vitro	In Vivo
Significant DNA damage	10	2	0	2	0		14	4	1	46	60	6	1	3	3	8	4	1	23	40	9	43	47	22	1	1
No-Significant damage	40	9	1	7	4		20	6	0	60	14	3	2	0	0	3	1	0	28	11	3	32	11	6	12	3
Total # Papers	50	11	1	9	4		34	10	1	106	74	9	3	3	3	11	5	1	51	51	12	75	58	28	13	4
% Significant damage	20	18	0	22	0		41	40	100	43	81	67	33	100	100	73	80	100	45	78	75	57	81	79	8	25
% No-Significant damage	80	82	100	78	100		59	60	0	57	19	33	67	0	0	27	20	0	55	22	25	43	19	21	92	75
Balance of Evidence (All Studies)	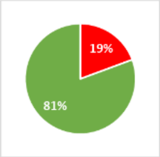			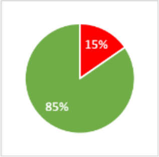			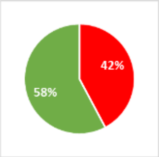			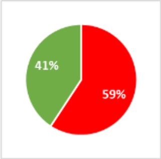			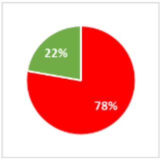			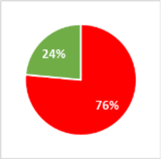			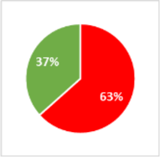			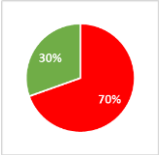			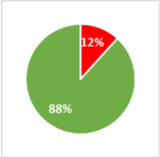	

Funding Source “Quality” Papers

Supp_Table 26: DNA damage vs funding source (quality papers) – heat map colour coding applied to “# Papers” as well as separately for “% Significant damage” for each experiment type

Funding Source	Industry		Telecom Regulator		Military		Government (excl. Military/ Regulator)		Private		Public		Institutional		Unknown/Not Declared	
Finding / Study Type	In Vitro	In Vivo	In Vitro	In Vivo	In Vitro	In Vivo	In Vitro	In Vivo	In Vitro	In Vivo	In Vitro	In Vivo	In Vitro	In Vivo	In Vitro	In Vivo
Significant DNA damage	2	1	1	0	6	1	13	22	0		3		3	8	6	6
No-Significant damage	16	6	0	1	6	1	21	6	1		1		6	2	14	1
Total # Papers	18	7	1	1	12	2	34	28	1		4		9	10	20	7
% Significant damage	11	14	100	0	50	50	38	79	0		75		33	80	30	86
% No-Significant damage	89	86	0	100	50	50	62	21	100		25		77	20	68	14
Balance of Evidence (All Quality Studies)	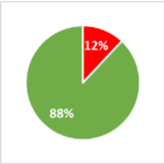		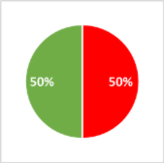		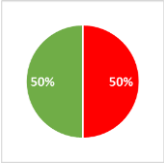		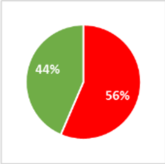		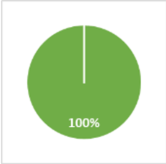		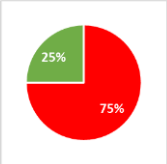		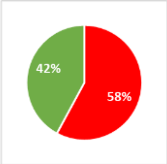		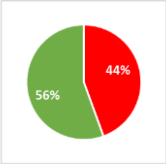	

Overall results plus vested interests

DNA Damage Outcomes for Vested Interests vs Independent Research

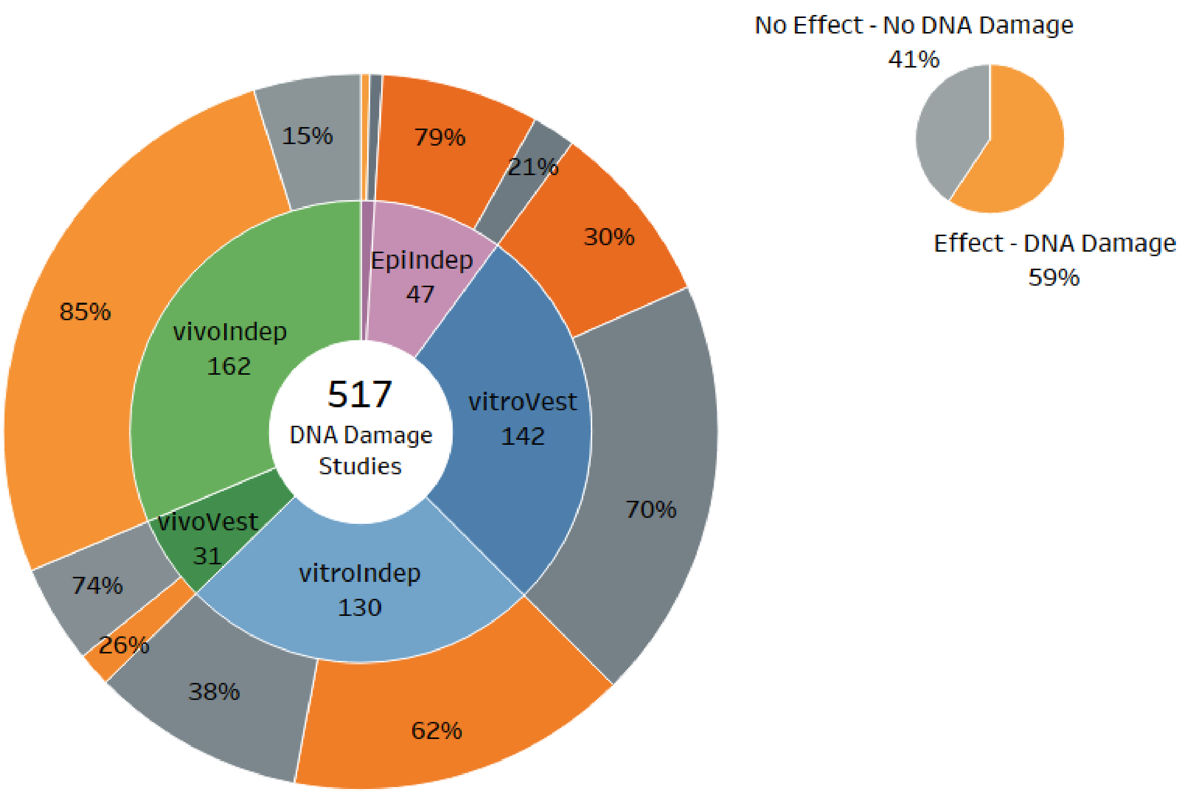

Supp_Figure 52: Vested interest vs independent research focus and findings

Author Potential COI

Supp_Table 27: DNA damage vs funding source (all papers) – heat map colour coding applied to "# Papers" as well as separately for "% Significant damage" for each experiment type

Author Affiliations (Past/Present)	Potential Vested Interests (All Studies)		Potential Vested Interests (Quality Studies)		Independent (All Studies)		Independent (Quality Studies)	
Finding / Study Type	In Vitro	In Vivo	In Vitro	In Vivo	In Vitro	In Vivo	In Vitro	In Vivo
Significant DNA damage	43	8	14	3	80	137	13	32
No-Significant damage	99	23	42	9	50	25	14	3
Total # Papers	142	31	56	12	130	162	27	35
% Significant damage	30	26	25	25	62	85	48	91
% No-Significant damage	70	74	75	75	38	15	52	9
Balance of Evidence (All Studies)	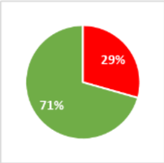		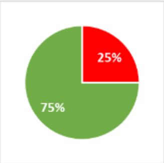		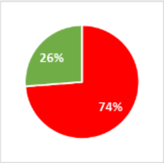		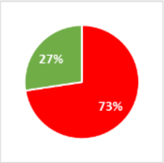	

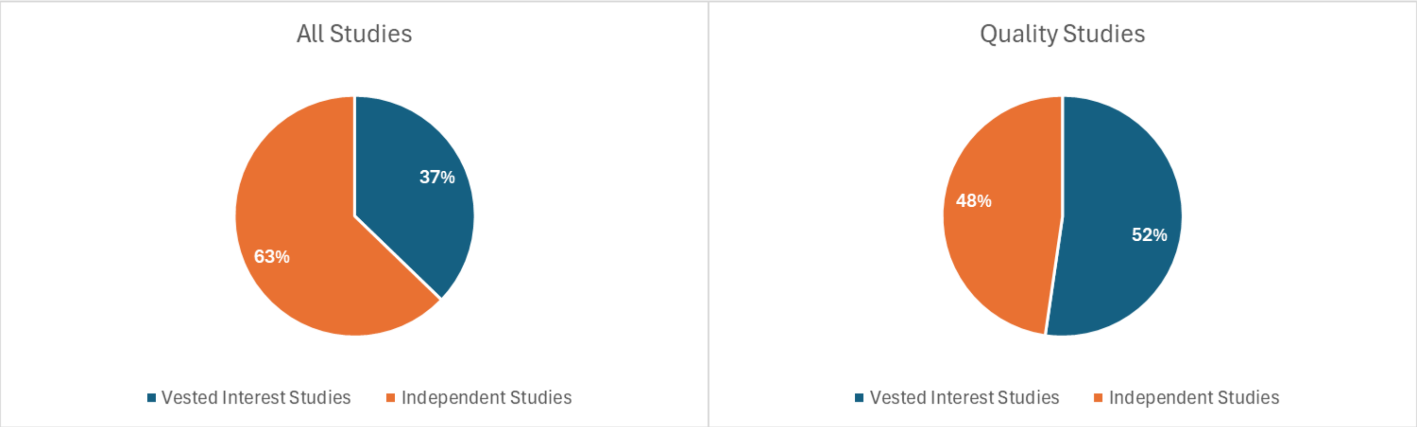

Supp_Figure 53: Distribution of Independent vs. Vested Interest Studies: All Studies vs. Quality Subset

Top Authors by findings

Top Authors by findings (X/Y/Z papers) where X represents the number of papers that either received funding from, or included co-authors from Industry, Telecom Regulator or Military, Y represent papers where funding was not declared, and Z represents total papers published

Supp_Table 28: Top Authors by findings and past funding

Top 10 No Effect Authors/Co Authors	Top 10 Effect Authors/Co Authors	Top 10 No Effect First Authors	Top 10 Effect First Authors
1. Maria Scarfi (3/6/19 Papers)	1. Vera Garaj-Vrhovac (1/5/11 Papers)	1. Vijayalaxmi (10/0/10 Papers)	1. Vera Garaj-Vrhovac (1/4/9 Papers)
2. Vijayalaxmi (11/3/18 Papers)	2. Jitendra Behari (1/0/9 Papers)	2. Anna Sannino (0/0/8 Papers)	2. Dimitris Panagopoulos (0/4/7 Papers)
3. Olga Zeni (3/4/16 Papers)	3. Kavindra K. Kesari (0/1/9 Papers)	3. Olga Zeni (2/2/7 Papers)	3. Henry Lai (1/0/5 Papers)
4. Anna Sannino (3/5/16 Papers)	4. Igor Y. Belyaev (0/3/9 Papers)	4. Anne-Marie Maes (1/3/4 Papers)	4. Kavindra K. Kesari (0/0/5 Papers)
5. Joseph Roti Roti (12/0/12 Papers)	5. Dimitris Panagopoulos (0/4/8 Papers)	5. Shin Koyama (4/0/4 Papers)	5.Gursatej Gandhi (0/4/5 Papers)
6. Thomas Prihoda (5/3/12 Papers)	6. Nesrin Seyhan (0/6/8 Papers)	6. James McNamee (0/3/3 Papers)	6.Ivancica Trosic (0/2/4 Papers)
7. William Straube (11/0/11 Papers)	7. Suleyman Dasdag (0/3/7 Papers)	7. Robert Malyapa (3/0/3 Papers)	7. Mihaela Răcuciu (0/2/4 Papers)
8. Eduardo Moros (10/0/10 Papers)	8. Mirta Tkalec (0/4/7 Papers)	8. Isabelle Lagroye (3/0/3 Papers)	8. Sanjay Kumar (0/0/4 Papers)
9. Stephanie Romeo (0/3/10 Papers)	9. Elcin Ozgur (0/5/7 Papers)	9. Ahmad Khalil (0/0/3 Papers)	9. Igor Belyaev (0/3/4 Papers)
10. Martin Meltz (10/0/10 Papers)	10. Sanjay Kumar (0/0/6 Papers)	10. Henning Hintzsche (0/1/3 Papers)	10. Mirta Tkalec (0/2/3 Papers)

Network Charts

No DNA Damage Effect Author Relationships

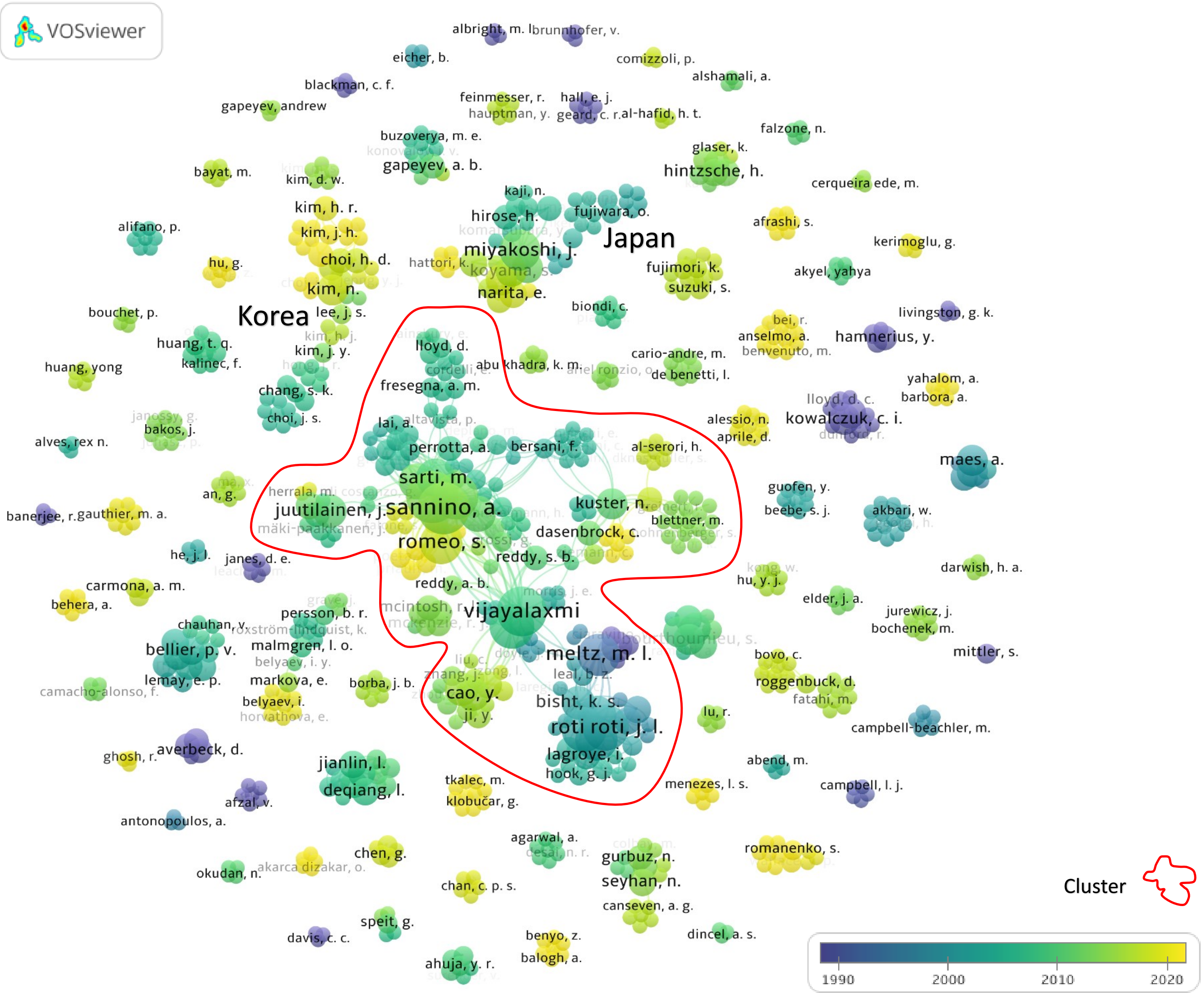

Supp_Figure 54: Bibliographic relationships for no DNA damage effect authors

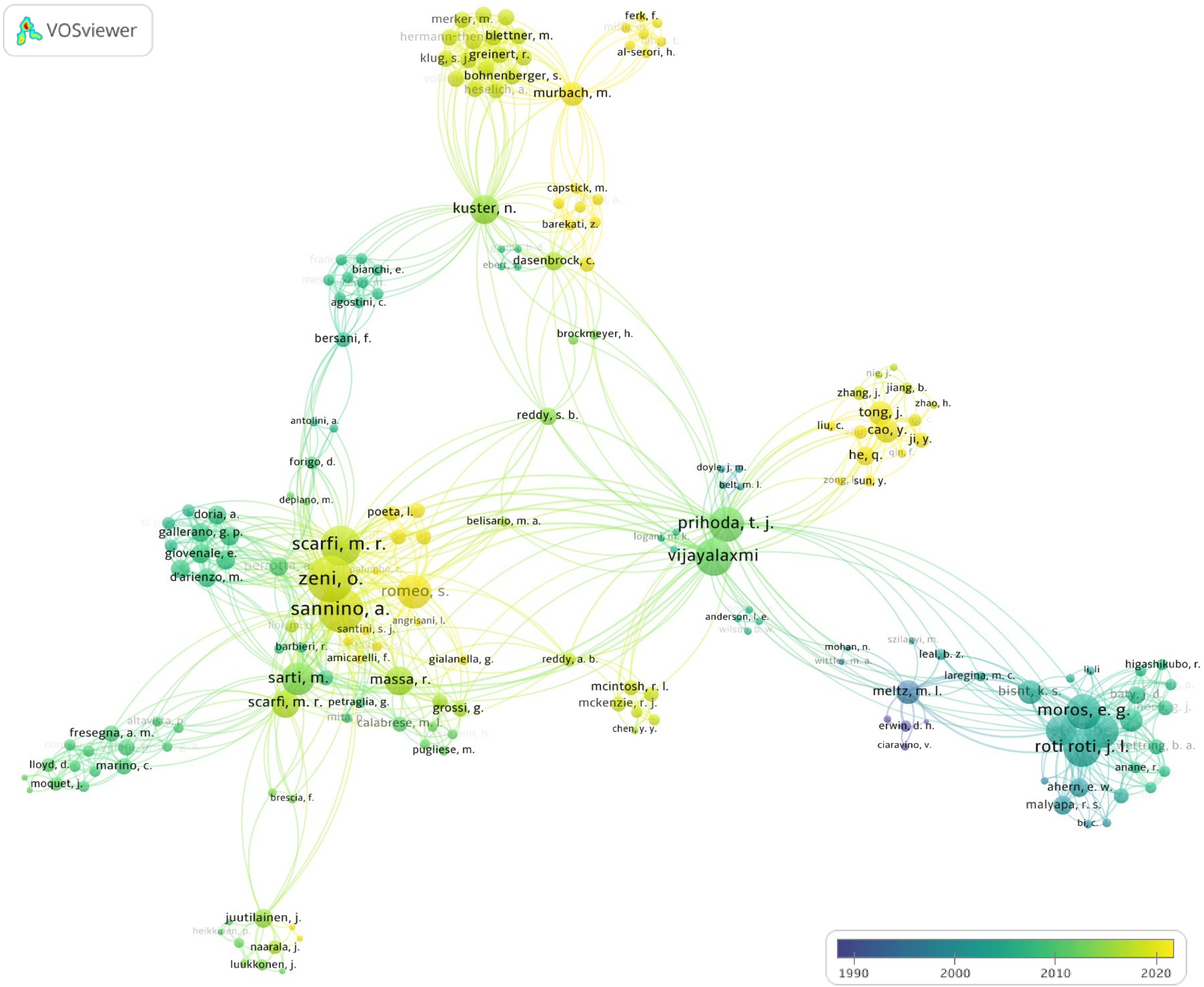

Supp_Figure 55: Bibliographic relationships for no DNA damage effect authors (Cluster)

DNA Damage Effect Authors Relationships

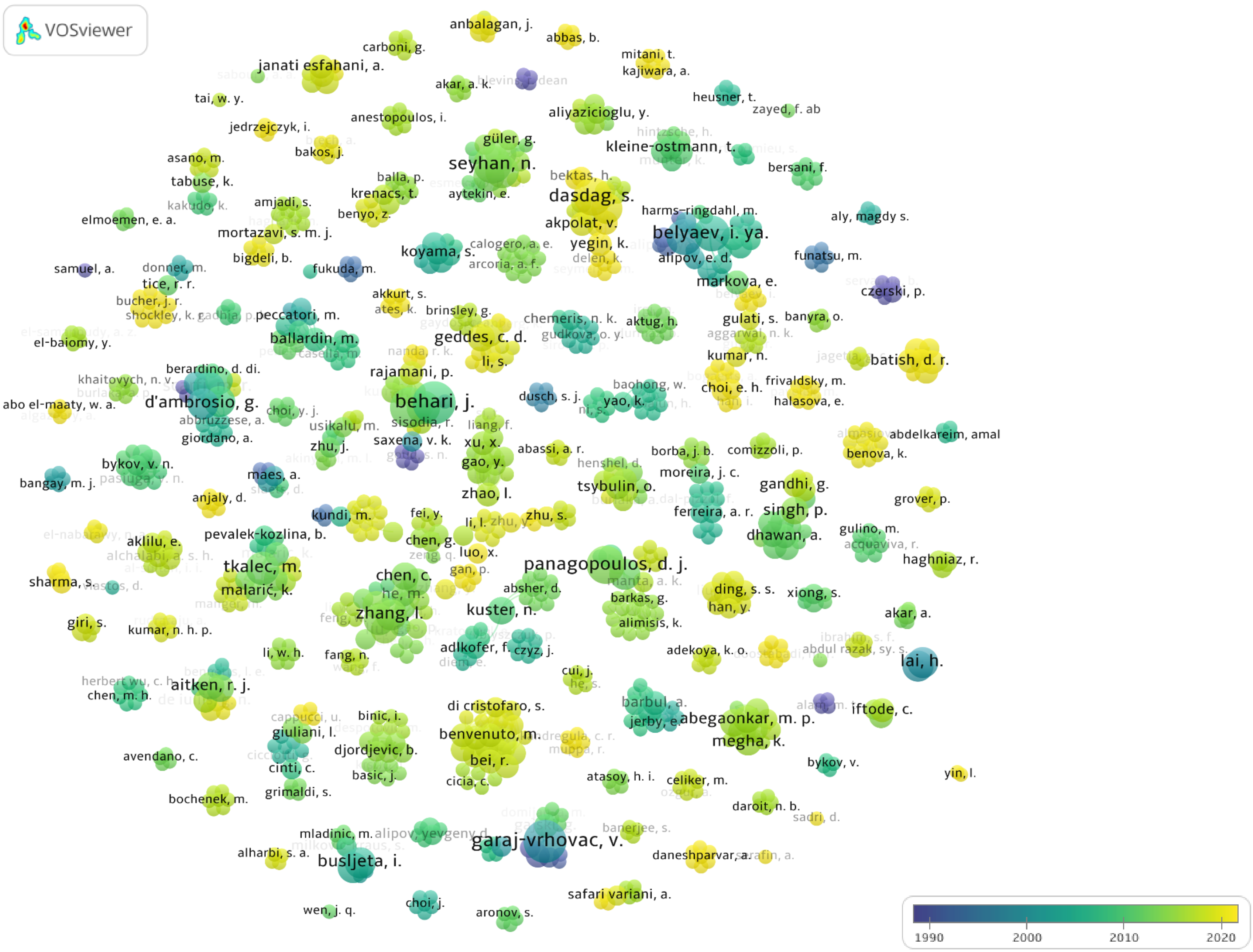

Supp_Figure 56: Bibliographic relationships for DNA damage effect authors

Journal Risk of Bias

Supp_Table 29: Top 10 Journals used for publishing (Effect studies vs No Effect studies)

Top 10 No Effect Journals	Top 10 Effect Journals	Top 10 Journals used by vested interests
1. Radiation Research (32 Papers)	1. Bioelectromagnetics (16 Papers)	1. Radiation Research (31 Papers)
2. Bioelectromagnetics (22 Papers)	2. International Journal of Radiation Biology (15 Papers)	2. International Journal of Radiation Biology (22 Papers)
3. International Journal of Molecular Sciences (22 Papers)	3. Electromagnetic Biology and Medicine (13 Papers)	3. Bioelectromagnetics (21 Papers)
4. Mutation Research - Genetic Toxicology and Environmental Mutagenesis (12 Papers)	4. Mutation Research - Genetic Toxicology and Environmental Mutagenesis (13 Papers)	4. Mutation Research/Genetic Toxicology and Environmental Mutagenesis (10 Papers)
5. Electromagnetic Biology and Medicine (5 Papers)	5. Radiation Research (8 Papers)	5. International Journal of Environmental Research and Public Health (4 Papers)
6. International Journal of Molecular Sciences (5 Papers)	6. PLoS ONE (7 Papers)	6. International Journal of Molecular Sciences (4 Papers)
7. Mutation Research (4 Papers)	7. Mutation Research Letters (5 Papers)	7. Journal of Radiation Research (4 Papers)
8. International Journal of Environmental Research and Public Health (4 Papers)	8. General Physiology and Biophysics (5 Papers)	8. Electro- and Magnetobiology (4 Papers)
9. The Journal of Microwave Power (3 Papers)	9. Mutation Research/Fundamental and Molecular Mechanisms of Mutagenesis (4 Papers)	9. Mutation Research (3 Papers)
10. Journal of Radiation Research (3 Papers)	10. Scientific Reports (4 Papers)	10. Environmental and Molecular Mutagenesis (3 Papers)
